# A Software Tool for Estimation of Burden of Infectious Diseases in Europe Using Incidence-Based Disability Adjusted Life Years

**DOI:** 10.1371/journal.pone.0170662

**Published:** 2017-01-20

**Authors:** Edoardo Colzani, Alessandro Cassini, Daniel Lewandowski, Marie-Josee J. Mangen, Dietrich Plass, Scott A. McDonald, Alies van Lier, Juanita A. Haagsma, Guido Maringhini, Alessandro Pini, Piotr Kramarz, Mirjam E. Kretzschmar

**Affiliations:** 1 European Centre for Disease Prevention and Control (ECDC), Solna, Sweden; 2 Julius Centre for Health Sciences and Primary Care, University Medical Centre Utrecht, Utrecht, The Netherlands; 3 NextPage Software Daniel Lewandowski, Zielona Gora, Poland; 4 Centre for Infectious Disease Control, National Institute for Public Health and the Environment (RIVM), Bilthoven, the Netherlands; 5 Section Exposure Assessment and Environmental Health Indicators, German Environment Agency, Berlin, Germany; 6 Department of Global Health, Institute for Health Metrics and Evaluation (IHME), University of Washington, Seattle, Washington, United States of America; 7 Department of Public Health, Erasmus Medical Center, Erasmus University Rotterdam, Rotterdam, the Netherlands; New York City Department of Health and Mental Hygiene, UNITED STATES

## Abstract

The burden of disease framework facilitates the assessment of the health impact of diseases through the use of summary measures of population health such as Disability-Adjusted Life Years (DALYs). However, calculating, interpreting and communicating the results of studies using this methodology poses a challenge. The aim of the Burden of Communicable Disease in Europe (BCoDE) project is to summarize the impact of communicable disease in the European Union and European Economic Area Member States (EU/EEA MS). To meet this goal, a user-friendly software tool (BCoDE toolkit), was developed. This stand-alone application, written in C++, is open-access and freely available for download from the website of the European Centre for Disease Prevention and Control (ECDC). With the BCoDE toolkit, one can calculate DALYs by simply entering the age group- and sex-specific number of cases for one or more of selected sets of 32 communicable diseases (CDs) and 6 healthcare associated infections (HAIs). Disease progression models (i.e., outcome trees) for these communicable diseases were created following a thorough literature review of their disease progression pathway. The BCoDE toolkit runs Monte Carlo simulations of the input parameters and provides disease-specific results, including 95% uncertainty intervals, and permits comparisons between the different disease models entered. Results can be displayed as mean and median overall DALYs, DALYs per 100,000 population, and DALYs related to mortality vs. disability. Visualization options summarize complex epidemiological data, with the goal of improving communication and knowledge transfer for decision-making.

## Introduction

Summary measures of population health (SMPH) are composite indicators that facilitate comprehensive and comparable quantitative assessments of health-related phenomena. Several measures have been developed for this purpose based on different assumptions, models and parameters [1–4]. One of the most common and prominent SMPH is the disability-adjusted life years (DALY), which has been largely used for global comparisons of the overall impact of diseases, injuries and risk factors [5, 6]. DALY is a composite metric quantifying the health losses measured in years by adding the number of years of life lost due to disability (YLD) and the number of years of life lost due to premature death (YLL).

A principal goal of the Burden of Communicable Disease in Europe (BCoDE) project is to provide the European Union and European Economic Area Member States (EU/EEA MS) with a tool to estimate the impact of communicable diseases on population health expressed in DALYs. The main objectives are to promote evidence-based methods in epidemiology, to facilitate planning and prioritization related to public health decision making, to identify gaps in surveillance data availability and quality, and to provide a comprehensive framework for communicating complex information to decision-makers.

The use and interpretation of DALY estimates is often challenging due to the complexity of a composite health indicator and due to the underlying assumptions made. Also, calculation of the DALYs can be quite computationally intensive and time consuming since DALYs have typically been calculated using multiple and complex tools such as spreadsheets, macros, complemented with ad-hoc add-on software (such as @Risk). The BCoDE project addresses these issues by creating a flexible and user-friendly software (the BCoDE toolkit, available from http://ecdc.europa.eu/en/healthtopics/burden_of_communicable_diseases/Pages/Tool.aspx#sthash.9GmX1e3Q.dpuf) able to estimate DALYs for several communicable diseases, and to provide clear and understandable results for public health professionals and policy makers [7].

In this paper we describe the technical and computational characteristics of the BCoDE toolkit and how this represents an important step forward by providing a consistent computational framework across diseases and populations.

## Design and Implementation

The BCoDE toolkit is a stand-alone Microsoft Windows^®^ 32-bit desktop application written in C++ using Qt C++ framework version 4.8.7. The main factors taken into consideration when designing the tool were: simple deployment, performance and customization capabilities. For this purpose, a client-side architecture has been chosen. The dual objectives of execution speed with a small memory footprint led to choice of a compiled language (C++) rather than a scripting language (JavaScript, R, etc.) for the core of the toolkit. The BCoDE toolkit runs on both 32- and 64-bit editions of Windows XP and later (so XP, Vista, 7, 8, 10) and the minimal requirements in terms of hardware are low: 1 GB of RAM, 1GHz CPU. No other operating system is currently supported (e.g., Android, OS X, Linux). The supported platform has been limited to Windows as the most widely spread operating system used by the potential clients in order to maximize the resources for features development and minimize maintenance burden. Additionally, sticking to the proven Win32 architecture maximally expanded the supported versions of Windows operating system (.Net, for instance, is a relatively new technology and requires an additional external software installed: .Net runtime). The toolkit is equipped with all the necessary external libraries included in the download package. The only dependency is the presence of Microsoft Excel OLE objects in the system which are usually installed together with the Excel application. This dependency is due to the fact that the majority of input data for the toolkit are stored in Microsoft Excel files format (Excel 97–2003 workbooks with extension “.xls”) which are used for loading and saving files. The remaining external inputs to the toolkit are disease reports which are stored as HTML files and are rendered in the interface without any additional dependencies. The prerequisites for compilation of the toolkit are:

1. C++ compiler;
2. Qt 4.8.7 framework;
3. Boost C++ libraries;
4. Visual Leak Detector.

All other prerequisites are distributed with the toolkit, which itself does not require any administrative rights.

The application is a hybrid of a desktop C++ application and a web application. All core functionality and computationally intensive tasks, including generating Monte-Carlo samples and post-processing results, are managed by the calculation engine implemented in C++. This gives an overall efficiency and speed to the calculations. The implementation follows the object-oriented paradigm. However, the graphical user interface is implemented in a web browser component embedded in the application. This feature, on the other hand, gives substantial possibilities for customizability. This is particularly valuable in the implementation of a dynamic walkthrough, visualization of outcome trees with interactive elements and output results tables and charts with complex formatting.

The toolkit is essentially a web page connected by a thin software layer to the C++ calculation engine handling data exchange between both worlds. The interface utilizes JavaScript as the programming language to implement handling of user actions and display of results. For that reason the software makes use of open-access external C++ libraries such as Boost, Marray, muParser, as well as JavaScript libraries jQuery, SlickGrid, jsPlumb, Flot and Trip.js.

The BCoDE toolkit does not include any scripting capability in the sense that it does not provide an API for use by external programs. The current version indeed limits the interoperability possibilities as project resources were allocated rather to areas determining the core functionality of the toolkit, and the scripting capability has not been seen as one. However, it should be stressed that the BCoDE toolkit implements a very generic calculation engine that is not restricted to any specific disease model. This single engine handles all implemented disease models. A specification of a disease model has been developed and each implemented model must be provided to the tool in accordance with this specification. This gives a possibility of extending the tool with new models in a relatively straightforward way and ongoing work is oriented on making this process even easier in the future versions. An additional advantage is the fact that only a single calculation engine must be tested and maintained, whereas multiple disease models are supported.

The license GNU General Public License v.3, under which the toolkit is released, was determined by ECDC. The various libraries and frameworks used internally vary in licensing, but have been on purpose selected to be quite permissive so that they can be used even in commercial, closed-source applications. This gives all range of possibilities for the licensing of the BCoDE toolkit, starting from more permissive (less restrictions) licence (e.g., MIT) to very restrictive (e.g., GPL). From the algorithmic point of view the toolkit executes calculations in the order defined by the outcome tree, starting from infection and traversing down the tree structure with the directions of transitions. Each entity in the outcome tree is able to retrieve inputs from the preceding entity, process this input data and expose outputs for the consecutive entity which will be picked up for further processing. Data exchanged between model components is organized into 2-dimensional matrix objects implemented using Marray library, a runtime-flexible multidimensional array. Similarly, all calculations are performed cell-wise at matrix level thanks to the implementation of arithmetic operators directly in the Marray library. This simplifies expressions as the usage of loops is limited.

Another aspect of the calculation engine is the need to deal with the optional stochastic nature of the disease models. Any input, or part of it, can be specified as a random variable or an expression including a random component. The toolkit gives an option to select the following probability distribution to draw samples from: Uniform, Pert, Beta, Gamma, LogNormal. Their sample generators are all based on the Boost random number generator with Mersenne Twister pseudorandom number generator as the generator of the underlying uniform sample. The standard implementation, MT19933, using 32-bit word length is utilized. In order to appropriately express uncertainty in the model outputs given the random nature of its inputs, Monte Carlo simulation methods are used. First, a representative sample of the inputs is generated from their respective probability distributions independently of each other. The sample size defaults to 1000, but can be overridden by the user. The algorithm repeats traversing the outcome tree in order to calculate outcomes for every value in the input sample. Sample size of the inputs also determines the number of repetitions. Eventually, the output sample is processed and various statistics, like mean value and 2.5, 50 and 97.5 percentiles, are computed and presented in the interface.

In order to provide a certain degree of freedom in setting the inputs, the toolkit includes a fast mathematical formula parser, muParser library. The user specifies mathematical expressions as an input value, rather than a constant or one of predefined values. The standard set of operators provided by the muParser library is very broad and was extended with a set of extra expressions for representing random number generators. The user, for instance, can specify the following expression as an input: “2 + sin(RandUniform(0, 1))”. The toolkit will then generate a value that is a sinus of random value sampled from a uniform distribution on interval [0, 1] and increased by 2. We found the performance of this expression parser to be very good and rarely a single full model run time on a moderately performing computer system (Intel Core2 Duo class) exceeds one second.

All calculations are executed with double-precision floating-point format used for variables.

## Results

### Disease models

Disease models (outcome trees) were created through a comprehensive literature review with the aim to describe the disease progression pathway of 32 selected communicable diseases and six healthcare-associated infections included in the project (Table 1, see S1 Document for details on the selection process) [7, 8]. Additional outcome trees were created whenever sex-specific or congenital/acquired forms of the disease lead to different health outcomes.

**Table 1:** Diseases and healthcare-associated infections included in the BCoDE toolkit for DALYs calculation.

**Diseases**
Campylobacteriosis
Chlamydia
Congenital Toxoplasmosis
Cryptosporidiosis
Diphtheria
Giardiasis
Gonococcal infections
Hepatitis A
Hepatitis B
Hepatitis C
HIV/AIDS
Infection with STEC/VTEC
Influenza
Invasive *Haemophilus influenza* disease
Invasive meningococcal disease
Invasive pneumococcal infections
Legionnaires`disease
Listeriosis
Measles
Mumps
Pertussis
Poliomyelitis
Q fever
Rabies
Rubella
Salmonellosis
Shigellosis
Syphilis
Tetanus
Tick-borne encephalitis
Tuberculosis
Variant Creutzfeldt-Jakob disease
**Healthcare-associated infections**
Healthcare-associated *Clostridium difficile* infection (HA CDI)
Healthcare-associated pneumonia (HAP)
Healthcare-associated neonatal sepsis
Healthcare-associated primary bloodstream infection (HA primary BSI)
Healthcare-associated surgical site infection (HA SSI)
Healthcare-associated urinary tract infection (HA UTI)

**Fig 1:**
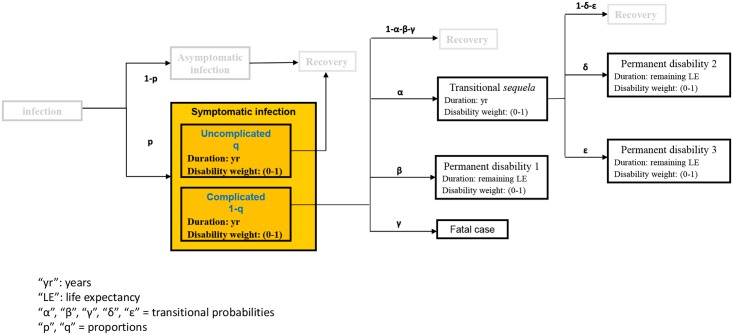
A disease outcome tree linking infection and all sequelae.

The outcome trees are shown in the BCoDE toolkit as graphical representations of disease progression. Detailed information on the modelling process are provided in S2 Document available from Mangen et al. [9]. In short, each box represents a distinct health outcome with a specific disability weight and a specific duration [10] (Fig 1).

Each arrow connecting various health outcomes is assigned a specific transition probability (probability for a case to move from one health outcome to the next, indicated by a Greek letter in Fig 1). The origin of each outcome tree is the infection (shown as a round box), and the entry point where the input data are entered, referring to the number of incident symptomatic cases and to the starting point of the modelling process, is shown in yellow. The end points in each outcome tree include death, recovery (R), and permanent disability. A health outcome can be further subdivided into health states according to different degrees of severity (see S2 Document). Each of these sub-boxes (health states) is assigned a specific disability weight, duration and proportion of cases that develop the health state out of the total number of cases that develop the overreaching health outcome.

Transition probabilities, durations of health outcomes, proportion of cases assigned to a certain health state within each health outcome (e.g., proportion of severe cases of acute symptomatic infection over the total number of acute symptomatic cases) were derived from literature reviews (a separate review of the literature was carried out for each of the 32 diseases and 6 healthcare-associated infections included in the tool) followed by rounds of disease experts’ consultations in order to validate the proposed parameters’ values.

Disease models have then been programmed in C++ and extensively tested by researchers with expertise in modelling disease progression. For application to specific diseases in different populations we conducted a pilot study covering four infectious diseases (salmonellosis, influenza, measles and hepatitis B) in four countries (Estonia, Germany, Italy, The Netherlands). Results were compared per disease and per health state for all four countries [11]. After the pilot study led to satisfactory results for these countries, a national disease burden estimation was performed with a pilot version of the toolkit for all 32 infectious diseases [12].

Details of the outcome trees are shown in specific tabs within the BCoDE toolkit [7]. The outcome trees represented in the toolkit are interactive. Users can explore all parameters by clicking on boxes and arrows. Information on all disease model parameters and how default values were specifically obtained for each communicable disease is included in the disease reports which describe references and rationale behind all parameters chosen. The disease reports are available in text form for each disease as an integral part of the BCoDE toolkit (disease report tab, next to the outcome tree tab) and in the S3 Document. The disease models and default parameters implemented in the present version of the BCoDE toolkit represent the result of a thorough reviewing and validation process that was performed over a time period of around 5 years involving disease-specific experts from many countries and institutions.

However, since disease progression can be different in different populations (due to e.g. health care system, access to health care, social economic factors) and since default values within the toolkit outcome trees were chosen with the aim to reflect a European average, the software application enables the user to choose other than default parameter values and to include uncertainty in those parameters into the analysis. All disease model parameters can be thus edited by the user (see below).

The user become then responsible for the correctness of the assumptions made and of the validation. In fact, although we see this flexibility as a great advantage of this tool for carrying out tailored research, simulation exercises, scenario analyses and more accurate national and regional disease burden estimations taking into account geographical specificities and other peculiarities of different populations, we also want to emphasize the importance for the user of always choosing parameters based on sound clinical and epidemiological evidence for the validity of the burden estimation.

The transition probabilities are of two types: lifetime transition probabilities (LTP) indicating a transition probability that applies once to all cases exiting the health outcome; and annual transition probabilities (ATP) which apply cyclically (i.e., annually) to all cases in the health outcome for the duration of the latter.

Results of BCoDE do not incorporate age-weighting, and are optionally shown with and without time discounting. The disability weights were obtained from European studies, involving more than 30,000 citizens, that applied elicitation methods (i.e. pairwise comparison) with the same methodology used by the Global Burden of Disease 2015 study but with disability weights tailored for the European population [13–15].

All parameter values can be specified according to 5-year age-group and/or sex if specific information is available. Moreover, each value can be entered as a constant or as an interval according to different distributions (Uniform, Pert, Beta, Gamma, LogNormal) by choosing the preferred option from a drop-down menu at the top of each input table.

### BCoDE toolkit interface

**Fig 2:**
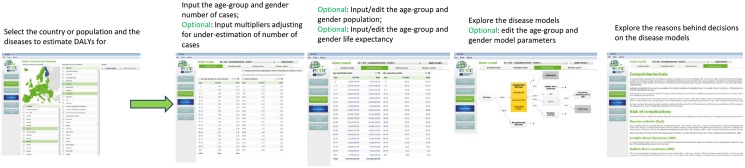
BCoDE toolkit: step-wise approach to disease model creation and data input.

The user interface consists of various menu options on the left hand-side of the screen that link to different pages: “Tutorial”, “Create models”, “Edit model data”, “Run models”, “View detailed results” and “View aggregated results”. The tutorial guides the user through the BCoDE toolkit and is available in a static (pop-up window) and in a dynamic version with walk-through functionalities. The user selects the disease model(s) and the population from the “Create models” page. Multiple selections are possible for all the listed diseases and all the EU/EEA MS or custom populations. This permits estimation of the burden of the same disease across populations, or the burden of different diseases within the same population. Its application could be, for example, the estimation of the burden of several diseases within one EU/EEA MS as well as the ranking of the same disease across different population groups. Fig 2 shows more information on available input interfaces.

On the “Edit model” page, incidence data can be entered manually and all parameters can be viewed and edited. Data import from Microsoft Excel^™^ tables is also enabled. Four additional tabs are included on the page: “Case data”, “Population data”, “Outcome tree”, and “Disease report”. The most important input, the number of age-group and sex-specific annual symptomatic cases for each selected disease, is entered in the “Incidence data” tab. Data are entered as number of cases stratified by age-group and sex. Using this tab, adjustment for under-estimation of the annual cases of symptomatic disease can be made, and a similar age- and sex-specific table can be populated with either a single or a combination of two multipliers, as described elsewhere [16]. Moreover, each value can be entered as a constant or as parameters of an assumed [stochastic] distributional form by choosing the preferred option from a drop-down menu at the top of each input table. The user can move between disease models through a drop-down menu at the top of the screen. The “population data” tab shows the Eurostat 2014’s sex- and age-specific population distribution. This tab also shows the standard life expectancy table required for DALY calculation [17]. Both tables can be modified by the user. The “Outcome tree” tab presents the selected disease models. When the natural history is different between males and females (e.g., chlamydia infection) and/or between acquired and congenital syndromes (e.g., syphilis), more than one outcome tree is shown. The models are interactive and detailed information presented in the tables appears as pop-up windows when clicking on the transition probabilities or on the health outcomes. All default values can be edited and saved. The “Disease report” page shows detailed information on the selected disease model.

If edited, disease models can be saved and stored to be loaded again whenever necessary using the top-left hand corner menu under “File”. Age-weighting is not implemented in the toolkit and time discounting is optional and can be set by the user. Computation in the application consists of Monte Carlo simulations of disease progression models. Therefore, once all the necessary information is entered in the “Edit model data” page, the user will be able to choose the number of iterations (default number is 1,000), and if time discounting should be applied, the desired discount rate needs to be specified. This approach ensures that all uncertainty ranges included in the disease model parameters and incidence data are taken into account. Hence, results are provided with 95% uncertainty intervals (UI).

### Outputs

In addition to a possibility of viewing the calculation outputs at outcome tree and disease model level, the user can explore results per individual sex/age category, outcome tree element (health state, transition), and type of *sequela*. The interface has been designed to give the user the option to view the output at various levels of aggregation, thus providing both a “birds-eye view” of the overall results, and a “zoomed-in” view of a specific health outcomes. It was a deliberate design choice of not exporting the actually generated sample as it is a vast data set even for a relatively simple disease model. A single unit of computation can be considered a 3D tensor (sex category x age category x sample size) occupying by default 39000 numeric cells (2 x 19 x 1000). Each health state requires three such tensors for storing its internal parameters (severity/duration/proportion) and two for outputs (number of cases, burden). Combining this with the model wide parameters (number of cases, underestimation, age distribution, life expectancy) and transition parameters would result in a substantial output dataset.

**Fig 3:**
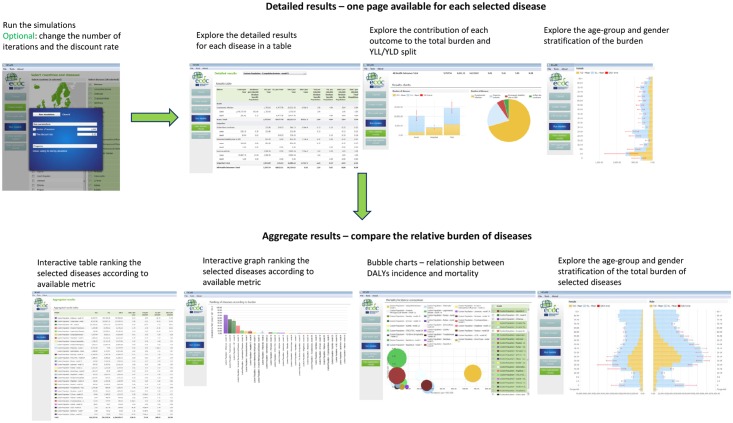
BCoDE toolkit: options for visualization of the outputs.

The BCoDE toolkit computes disease-specific results displayed as DALYs calculated for the selected set of disease models, and based on the incidence values entered by the user. Once the models are run, the results are presented as “Detailed results” separately for each disease model, and as “Aggregated results” comparing and ranking the various disease models according to their burden in DALYs (Fig 3).

The “Detailed results” page shows a summary table providing total and health outcome-specific results. This table shows the estimated number of cases and of deaths, incidence per 100,000 population, years of life lost due to disability (YLD), years of life lost due to premature death (YLL), DALYs, and DALYs per case as well as YLD per 100,000 population, YLL per 100,000 population, and DALYs per 100,000 population. DALYs per case represent the average burden of a disease experienced by each infected symptomatic case and it can be interpreted as a measure of average severity apart from those disease models where some of the burden comes also from asymptomatic infections (e.g., hepatitis B). The table is followed by a bar chart presenting results with the relative contribution of acute disease and *sequelae*, and a pie chart showing the contribution of the different health outcomes to the total burden. Finally, a series of interactive tables and bar charts complete the detailed description of the burden of each disease. Averages, medians and age- and sex-specific 2.5 and 97.5 percentiles are displayed for DALYs, DALY per case, DALY per 100,000 total population and DALY per 100,000 stratum specific population.

The final page “Aggregated results” compares the results of the selected disease models. An interactive table and a bar chart enables the user to rank the burden of disease by a selected metric from the ones listed above. Moreover, bubble charts are shown comparing different disease models, in which the size of each bubble corresponds to the magnitude of the burden of disease expressed in DALYs per 100,000 population. In the first graph the x-axis represents the estimated incidence per 100,000 of symptomatic disease, while the y-axis represents the estimated mortality per 100,000 population calculated through the disease model. The second graph differs only by showing DALYs per case on the y-axis. The “Aggregate results” page also shows interactive age-group and sex-stratified tables and bar charts similar to those described for the “Detailed results” page for the aggregate results of all diseases.

All results are printable and exportable in portable document format (PDF) and in Microsoft Excel^™^.

### The BCoDE toolkit in practice

The BCoDE project employs an incidence- and pathogen-based DALY approach based on disease-specific models (outcome trees) [9,11,18]. For the estimation of healthcare-associated infections (HAIs), a syndromic approach was used based on different infection sites [19].

The BCoDE study summarized the 2009–2013 comparative impact of 31 infectious diseases [20,21] and the 2011/2012 impact of 6 HAIs [19] (Table 1) in DALYs across EU/EEA MS.

Within the BCoDE 2009–2013 study, the default starting point for the estimation of incidence, which is a necessary prerequisite of the underlying disease-specific models, was generally the cases notified to The European Surveillance System (TESSy) [22]. The assessment of how much notified cases underestimate the total number of symptomatic cases in the population was carried out through extensive literature review of previous studies addressing this specific issue, with a particular focus on studies carried out in EU/EEA MS [16].

The BCoDE toolkit has also been used for the assessment of an intervention comparing the burden of measles in EU/EEA Member States compared to the measles vaccination coverage [23].

## Availability and Future Directions

The BCoDE toolkit is a user-friendly application to calculate DALYs for 32 communicable diseases and six HAIs. The interface and the layout are meant to facilitate utilization by public health professionals who are not necessarily familiar with the burden of disease methodology, and to enable more effective communication of ranking of infectious diseases in terms of disease burden within and across different populations. Moreover, this tool provides a standardized method for estimation of burden of infectious disease expressed in DALYs in different settings. Since January 2016, it is available for download as a stand-alone software application, available from the ECDC`s website: http://ecdc.europa.eu/en/healthtopics/burden_of_communicable_diseases/Pages/Tool.aspx.

The BCoDE toolkit may be used by interested professionals from academia and EU/EEA national health institutes enabling the estimation of the burden of communicable diseases [24]. Until now (December 2016), the ECDC webpage hosting the link for downloading the BCoDE toolkit has had nearly 2,800 potential downloads. Furthermore, 44 public health experts from national public health institutes not limited to EU/EEA countries, as well as from academia, hospitals and international organizations, have downloaded the toolkit and subscribed to a newsletter reminding users when updates are available.

National experts will be able to estimate national and subnational burden of communicable diseases, or more generally to introduce DALYs to their epidemiological research. For example, the Centre for Infectious Disease Control at the National Institute for Public Health and the Environment in the Netherlands (RIVM) has estimated the national burden of infectious diseases by adapting the BCoDE toolkit models to the Dutch epidemiological situation, using the BCoDE toolkit to calculate DALYs and communicating the results through the visualization options of the software [12]. Depending on their access to and understanding of local availability and quality of data, experts may be able to enter the most accurate incidence and outcome tree data for the setting under study. In the quantification of the actual occurrence of a symptomatic infection, national experts may have more detailed information on potential changes in the sensitivity of the data sources used due to contingent situation, such as outbreaks or changes in the surveillance system or laboratory testing, and may take this information into account when adjusting for underestimation. Notably, the BCoDE toolkit has been included in an European Food Safety Authority’s (EFSA) Scientific Opinion on risk ranking tools and was recommended for use by European experts when developing risk ranking of biological hazards [25].

As with all epidemiological analyses, it is crucial to keep in mind that the quality of the output depends on the quality of the input. For many communicable diseases the notification data are not aimed at capturing the exact overall number of incident cases of disease; therefore alternative methods for obtaining incidence data (e.g., via modelling) or alternative data sources (e.g., from serological studies) should be considered. These decisions have to be made by the user who therefore needs to have an in-depth understanding of the quality of the disease incidence data available for the population of interest. Additionally, the incidence-based approach estimates the present and future burden based on the yearly incidence entered in the models. The starting point of the models is the new cases of symptomatic disease, hence, people affected with a chronic condition caused by a past infection do not contribute to the burden, ultimately underestimating the results. A planned improvement to the BCoDE toolkit is the optional inclusion of prevalence-based methodology for estimating the burden of these diseases.

Default disease model parameters were chosen from a literature review carried out with a European-wide perspective, rather than a national one, however, all parameters are modifiable by the user. Registered users will be informed about all updates of the software, which will be published regularly on the ECDC website. Conditional on funding, we are also planning to perform regular literature reviews on all disease models and parameters to keep the default disease models in the software up to date. An additional planned improvement will allow the user to create novel outcome trees (i.e., boxes and sub-boxes for health outcomes and states, and arrows for transitional probabilities). Related to this, an open access library where users can share new models is foreseen.

In conclusion, the BCoDE toolkit is a user-friendly software for estimating the burden of communicable diseases. The toolkit facilitates communication between data analysts and users through multiple visualization options, ultimately fostering its value in health policy communication. The use of the Toolkit could hopefully stimulate further improvements in epidemiological data availability and quality. Planned enhancement in the BCoDE methodology and toolkit should contribute to more effective and evidence-driven health policy decision-making.

## Supporting Information

S1 Document Criteria used when selecting pathogens/diseases. (PDF)

S2 Document Defining health states in a pathogen and incidence-based DALY approach. (PDF)

S3 Document Results from BCoDE 2015 study, Disease models—Outcome trees. (PDF)
