# Supplementary material for: A Software Tool for Estimation of Burden of Infectious Diseases in Europe Using Incidence-Based Disability Adjusted Life Years
Source: PLoS One. 2017 Jan 20;12(1):e0170662. doi: 10.1371/journal.pone.0170662 (PMC5249178; doi:10.1371/journal.pone.0170662)
Supplement: S1 Document — (PDF) [file pone.0170662.s001.pdf]

Annex 1. Criteria used when selecting pathogens/diseases

The master list is based on the objectives set out in the BCoDE Call for Proposal and includes:

- Forty-nine communicable diseases and special health issues specified under Decision 2119/98/EC with amendments.
- Other diseases and conditions which may result from infections, including infection-associated cancers. Based on a strong recommendation by the experts assembled at the BCoDE expert workshop in March 2010, the following conditions will be included in this category: end-stage liver disease or primary liver cancer related to hepatitis B virus (HBV), or hepatitis C virus (HCV) infection; cervical cancer related to human papilloma virus infection and gastric cancer related to *Helicobacter pylori* infection.
- Other conditions were added following discussions with ECDC Disease-Specific Programmes (DSP) and include diseases such as leishmaniasis, Lyme disease, tick-borne encephalitis and viral haemorrhagic fevers (including Crimean-Congo haemorrhagic fever, diseases caused by hantaviruses, dengue, Rift Valley fever, diseases caused by the Marburg filovirus and chikungunya).

Exclusion criteria:

1. Expert consensus (based on expert consideration of published evidence) that the burden of a disease/special health issue is negligible, mostly due to very low incidence, defined as equal to or lower than 1/100 000 per year.
2. Extreme methodological and practical difficulties foreseen in estimating the burden due to:
   - Lack of data and very low probability of data generation over the next five years
   - Serious methodological difficulties foreseen, for example in defining a disease and/or special health condition
   - Lack of disability weights for the most important health states of outcome trees, and absence of similar alternatives.

Exemptions:

If Criterion 1 for exclusion is met but:

- the negligible burden is believed to be low due to vaccination programmes with high coverage, or
- there is an outbreak potential based on history or theoretical considerations,

then the disease/special health issue will be kept on the final list and an attempt made to estimate the burden in the absence of intervention (e.g. by using the prevented fraction estimates in the case of interventions and mathematical modelling in the case of outbreak potential).

Process for defining the final list:

- The master list will be reviewed by the ad hoc working group of the ECDC Advisory Forum.
- Decisions will be based on a simple majority of responses from the group.
- The working group will start by considering the methodological issues (Criterion 2 above, Column A in the example table below). If the majority of responses relating to the feasibility of burden estimation is negative, the disease/health issue will be dropped from the list.
- The resulting shortlist will be assessed against the incidence (Criterion B: Above very low relative incidence i.e. ≥1/100 000 per year). Diseases that score a majority of "No" answers under Criterion B need to have a majority of "Yes" under Criterion C (Outbreak potential) or D (Vaccine-preventable with widely used vaccine) in order to be kept on the list.
- The final list will then be discussed with the Chief Scientist of ECDC and the management team of the project consortium.

Where necessary, experts can add or remove other diseases, by producing thorough justification which must be approved by the Chief Scientist of ECDC.

Table 1. Decision tool to facilitate selection of diseases/health conditions

Diseases/health issues		Criterion A	Criterion B	Criterion C	Criterion D
Respiratory tract infections		Feasibility based on data availability or methodological issues<br>Y/N	Above very low relative incidence (i.e. ≥1/100,000 per year)<br>Y/N	Outbreak potential?<br>Y/N	Vaccine-preventable with widely used vaccine?<br>Y/N
1	Seasonal influenza				
2	Avian influenza				
3	Legionellosis				
4	Tuberculosis				
5	etc.				
