# Supplementary material for: A Software Tool for Estimation of Burden of Infectious Diseases in Europe Using Incidence-Based Disability Adjusted Life Years
Source: PLoS One. 2017 Jan 20;12(1):e0170662. doi: 10.1371/journal.pone.0170662 (PMC5249178; doi:10.1371/journal.pone.0170662)
Supplement: S2 Document — (PDF) [file pone.0170662.s002.pdf]

# Annex 2.

# Defining health states in a pathogen and incidence-based DALY approach

## 1. Assessment of health outcomes

*Definition of the term health outcome and health state*

An infectious disease may have diverse manifestations. Manifestations may be local (e.g. abscess or encephalitis) or systemic (e.g. flu, sepsis, malaria). The severity of the manifestations differs from very mild to extremely severe. The clinical manifestations and symptom severity depend on e.g. pathogen virulence, host immunity, time since infection and initiation of therapy. Ideally, the range of clinical manifestations of infectious diseases and the continuum of symptom severity allows the identification of a small set of well-defined categories in order to calculate YLD. These well-defined categories are termed "sequelae" in the GBD-study [1]. The GBD use of the term "sequela" covers the traditional clinical meaning and a broad categorization of health outcomes into severity levels for particular impairments. Our aim is to derive categories that can be used as a basis for the calculation of YLD. We distinguish between categories that describe qualitatively different disease manifestations (so-called "health outcomes"; e.g. gastroenteritis versus Guillain-Barré syndrome versus reactive arthritis in figure 1) and categories that are similar in clinical manifestation but with a different severity level and duration (so-called "health states"; e.g. mild versus severe gastroenteritis in figure 1).

Figure 1. Outcome tree of *Campylobacter* spp.

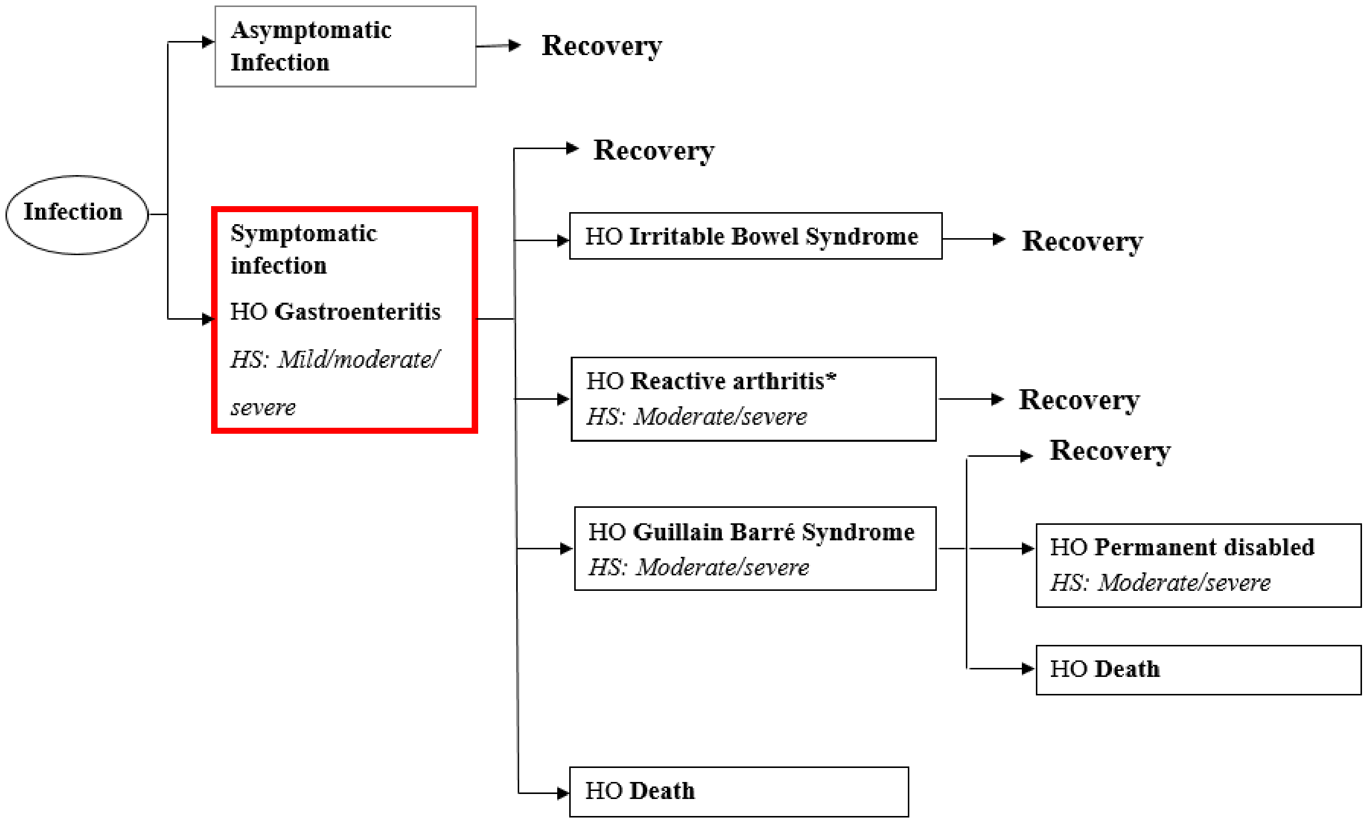


HO = health outcome, HS = health state

*Horizontal and vertical disaggregation*

To clarify the distinction between health outcomes and health states, we introduce the concepts of horizontal and vertical disaggregation. By "vertical" disaggregation we mean a discretization of the continuum of severity into a small number of categories. Every individual of an infected population is mapped into exactly one severity category. In case of infectious diseases, the severity level of the health state may differ, but the International Classification of Diseases (ICD)-code is the same and also the case definitions used by e.g. European Centre for Disease Prevention and Control (ECDC) or Centers of Disease Control and Prevention (CDC) do not distinguish between severity categories. Figure 2 illustrates the concept of vertical disaggregation. In this figure A1 and A2 represent the same health outcome (e.g.

gastroenteritis), but a different severity level (i.e. health state) in different individuals (e.g. mild, moderate or severe gastroenteritis).

Figure 2. Illustration of the concept of vertical disaggregation. The health-related quality of life (HRQoL) is plotted against the time since infection. A1 and A2 represent different manifestations of the same health outcome in different individuals (e.g. mild and severe gastroenteritis).

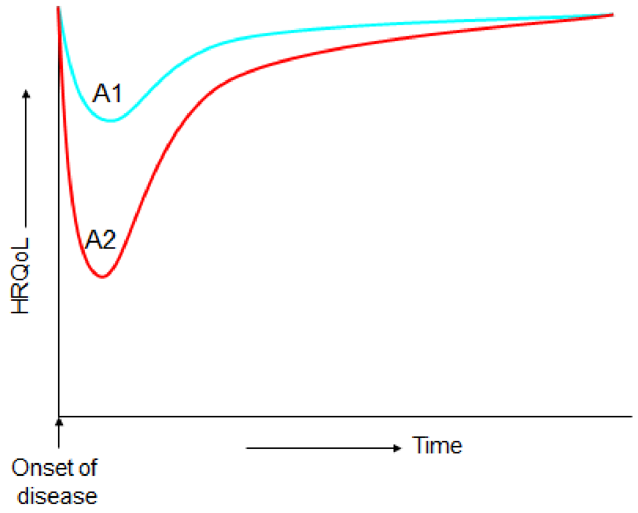


By "horizontal" disaggregation we describe the situation that clinical manifestations occurring sequentially in time can be distinguished and categorized into different health outcomes. Horizontal disaggregation is required if the effects of a disease manifests itself in separate consecutive stages in one and the same person. These health outcomes are clinically different conditions or diseases and can be distinguished by different ICD codes (e.g. Campylobacter infection where a person first experiences gastroenteritis followed by Guillain-Barré syndrome). For different health outcomes, different diagnostic methods may be required and different treatments may be needed. Figure 3 illustrates the concept of horizontal disaggregation. In this figure A and B represent two consecutive health outcomes occurring in one and the same person.

Figure 3. Illustration of the concept of horizontal disaggregation. The health-related quality of life (HRQoL) is plotted against the time since infection. The change of clinical manifestation over time is shown for one individual, who develops first health outcome A (e.g. gastroenteritis) and later health outcome B (e.g. Guillain-Barré syndrome) as a sequela of an infection event.

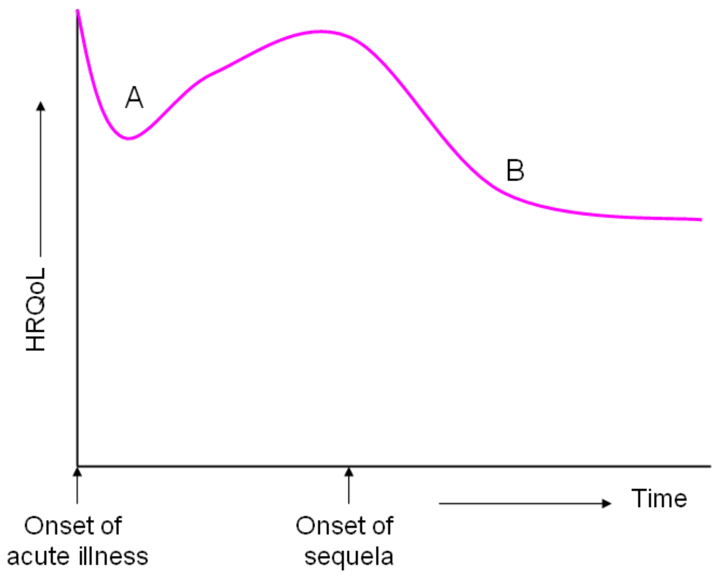


Using the concepts of horizontal and vertical disaggregation we can make the following distinction of health outcomes:

*Uncomplicated case (case definition of illness)*

An uncomplicated acute illness of an infection with a typical manifestation as described a standardized case definition (e.g. influenza; salmonellosis), which is normally the first health outcome after infection, can be seen as an "uncomplicated case" of that health outcome. The majority of cases of infectious disease are in general uncomplicated cases.

*Complicated case*

The pathogen that causes the typical acute infection then causes atypical disease symptoms that may be much more severe than the uncomplicated course of infection (e.g. sepsis due to Salmonella infection). Such infections may have a longer duration and an increased severity and thus would require a different disability weight than uncomplicated infections. Also, the case-fatality ratio may be higher. This distinction is then described as vertical disaggregation. The health state A1 in Figure 2 may represent an uncomplicated case, whereas A2 represents a complicated case of an infection.

*Superinfection*

A superinfection is a co-infection with another pathogen than the one causing the primary infection. The second infection develops either together with the first infection or soon thereafter (e.g. influenza followed by pneumonia). The episode of acute infection is considered one single episode that is slightly longer and more severe than an “uncomplicated” infection. Again health state A1 in Figure 2 may represent an uncomplicated case, whereas A2 represents a case with a co-infection.

*Sequelae*

A sequela is a pathological condition resulting from a prior infection in one and the same person and is characterized by ICD codes different from the initial acute disease. Immune-mediated diseases such as reactive arthritis and Guillain-Barré Syndrome which are triggered by a primary infection with a pathogen are considered sequelae. Health outcome B in Figure 3 may represent such a sequela, where health outcome A represents the prior infection (i.e. gastroenteritis). Failure of organs resulting from a chronic infection with the pathogen will also be considered as a sequela. When constructing an outcome tree it is essential to identify and define all possible sequelae of that are causally related to an infection, including long-term sequelae. According to Mitchell et al.[2] the strength of evidence for a causal relationship can

be classified in different categories. We include only sequelae for which sufficient evidence for a causal relationship or an association exists to avoid overestimation of the burden of infectious diseases.

References


1. Mathers, C.D., et al., *National Burden of Diseases Studies: A Practical Guide. Edition 2.0*. 2001, WHO Global Program on Evidence for Health Policy: Geneva.
2. Mitchell, A.E., L.B. Sivitz, and R.E. Black, *Gulf war and health. Volume 5: Infectious Diseases.* 2007, Washington DC: The National Academies Press; 2007.: The National Academies Press.
