# Supplementary material for: A Software Tool for Estimation of Burden of Infectious Diseases in Europe Using Incidence-Based Disability Adjusted Life Years
Source: PLoS One. 2017 Jan 20;12(1):e0170662. doi: 10.1371/journal.pone.0170662 (PMC5249178; doi:10.1371/journal.pone.0170662)
Supplement: S3 Document — (PDF) [file pone.0170662.s003.pdf]

Burden of Communicable Diseases in Europe Project

Office of the Chief Scientist Unit

Supplementary Information 3
Results from BCoDE 2015 Study

Disease models – Outcome trees

Campylobacteriosis

Acute gastroenteritis associated with Campylobacter infections in humans is in most cases self-limiting after a few days to weeks, but for some patients the disease may be fatal. When available, information on duration of illness mainly relates to cases having requested medical help. These cases are often the most severe cases of longer duration. For example, the overall mean duration of illness due to Campylobacter infection observed in the GP[*] case control component of the IID study in England and Wales was 9.34 days, whereas it was only 6.52 days for all cases observed in the community component (Adak, 2002). About 47% of the community component cases would have visited their GP (Food Standards Agency, 2000). Based on the IID study, it is has been assumed that gastroenteritis caused by campylobacteriosis would last 3.22 days (no medical help), 9.72 days (visiting GP) and 14.39 days (hospitalised) (Mangen, 2004; Mangen, 2005). In our current model we chose to apply 3.22–9.72 days for all uncomplicated cases and 14.39 days for the complicated ones.

Bacteraemia is highlighted in many reports as a possible extra-intestinal complication of campylobacteriosis. For example, Skirrow et al. (Skirrow, 1993) estimated a bacteraemia incidence of 1.5 per 1 000 reported campylobacteriosis cases, whereas Ternhag et al. (Ternhag, 2008) reported an absolute risk of bacteraemia/sepsis of 0.02% for laboratory-confirmed campylobacteriosis cases.

Assuming that GP visits represent an indication of moderate diarrhoea and that the proportion of hospitalised cases represents severe diarrhoea, we divided cases into the following groups: uncomplicated (mild diarrhoea) 75.5%, complicated (GP, moderate diarrhoea) 23.5% and complicated hospitalised (severe diarrhoea) cases 1% (Kemmeren, 2006; Kwong, 2012; redistributing to total 100%).

Estimates of campylobacteriosis case fatality proportions range from 0.001% to 0.05%: 0.05% (Mead, 1999), 0.024% of all foodborne campylobacteriosis cases in the IID study (Adak, 2002), 2–6% of the hospitalised cases (Buzby, 1996; corresponding to 0.012–0.036% of all cases, considering that 0.6% of cases are hospitalised according to Mangen et al. 2004), 1.3 fatal cases per year, corresponding to 0.001% of the estimated 123 000 Campylobacter cases (Cressey & Lake, 2007), 0.038% of all symptomatic cases (Mangen, 2004).

We chose to estimate the overall case fatality proportion as being within the range 0.001–0.05% and assumed a different age-group distribution of this risk based on the age-group distribution of reported deaths to TESSy between 2009 and 2013 (see Table 3). This table is based on all TESSy notified cases from EU Member States except Bulgaria, reporting only aggregate data, Greece, Portugal and Liechtenstein which do not report.

Risk of complications

Reactive arthritis (ReA), irritable bowel syndrome (IBS) (but not inflammatory bowel disease due to lack of confirmation of a biological link and limited evidence) and Guillain-Barré syndrome (GBS) may be associated with campylobacteriosis.

Reactive arthritis (ReA)

ReA is a significant long-term sequelae following campylobacteriosis (Keat, 1983; Johnsen, 1983; Hannu, 2002). A retrospective study carried out in Finland found that 7.4% (45/609) of laboratory-confirmed campylobacteriosis cases fulfilled the criteria for ReA (Hannu, 2002), which is similar to that found by another study: 8.1% (3/37) (Johnsen, 1983). A further study reported a 2.6% (9 of 350) frequency of ReA in patients contacting a municipal health centre following an outbreak of C. jejuni (Hannu, 2004) and 16% of laboratory-confirmed cases self-reported having had ReA (Locht & Krogfeld, 2002), although self-reporting might be prone to overestimation (Hannu, 2002). Other studies including clinical testing report a 2.8% and a 2.4% risk of developing rheumatological symptoms (Rees, 2004; Kosunen, 1980). In order to account for the large uncertainty, the risk of developing ReA from all symptomatic cases is 1.7% (0.73–4.4%) (Kemmeren, 2006).

Little is known about the duration of ReA; the average duration is between 1.5 months derived from Hannu et al. (Hannu, 2005) and 222 days (Kemmeren, 2006).

Irritable Bowel Syndrome (IBS)

In a recent literature review, 8.8% (7.2–10.4%) of symptomatic campylobacteriosis symptomatic cases were considered at risk of developing IBS, irrespective of age and gender; the duration was set to five years (Haagsma, 2010). However, the causality is largely debated and the impact of concurrent factors significant. Therefore, IBS is not considered as part of the campylobacteriosis outcome tree in our study.

Guillain-Barré syndrome (GBS)

GBS is a neurological disease frequently preceded by an acute infectious illness, mainly upper respiratory infections and gastrointestinal infections. The functional status of patients with GBS is scored on a seven-point disability scale (F-score), ranking from 0 (healthy) to 6 (death). GBS-patients with an F-score at nadir of < 3 (able to walk unaided at nadir) are considered to be mildly affected. GBS patients with an F-score of ≥ 3 (unable to walk unaided at nadir) are considered to be severely affected (van Koningsveld, 2001). Paralysis from GBS is generally reversible over time, but some patients are bedridden for life and others die prematurely.

Incidence is estimated at 0.8–2.0 or 0.4–4 cases per 100 000 persons year (van Koningsveld, 2001; Mc Grogan, 2009; Hughes & Rees, 1997) in Europe and North America. A systematic review of the literature and metanalysis estimated an age-specific GBS rate per 100 000 person years of exp[-12.0771 + 0.01813(age in years)] × 100 000 (Sejvar, 2011).

Studies show that 14–36% of GBS patients previously had a Campylobacter infection (Jacobs, 1998); 33–50% of GBS patients had increased levels of Campylobacter spp. (Mishu, 1993). A more recent systematic literature review estimated that 31% of the 2 502 GBS cases studied were attributable to Campylobacter infection (Poropatich, 2010).

Research has found that about 0.022% of laboratory-confirmed campylobacteriosis cases would develop GBS (13/57,425) (Ternhag, 2008), resulting for all symptomatic cases in a 0.0015% risk of developing GBS; in Sweden one GBS case per 3 285 Campylobacter jejuni infections (95% C.I.: 1.729 – 7.210) resulting in a risk of 0.03% (McCarthy & Giesecke, 2001); in the USA one per 1 058 campylobacteriosis cases (0.09% risk; Allos, 1997). Studies estimating the burden of campylobacteriosis assumed a 0.075% and 0.023% risk of developing GBS (Mangen, 2004 and 2005; Cressy & Lake, 2007). Given the large diversity found in the literature, the risk of developing GBS following a symptomatic Campylobacter infection is set to 0.0015–0.09%.

Males were more commonly affected by GBS in almost all studies (Sedano, 1994; Hughes & Rees, 1997; Nachamkin, 1998; Nagpal, 1999; van Koningsveld, 2000; Sejvar, 2011). However, these differences might be based on environmental factors as well as biological factors (van Koningsveld, 2000) and therefore it is difficult to speculate about the origin of this gender difference and the cause and determinants of GBS and therefore we do not distinguish in risk between genders.

Havelaar et al. (Havelaar 2000 a,b) estimated the proportion of mild and severe GBS cases after Campylobacter infections to be 17% and 83%, respectively. Age plays a role (van Koningsveld et al., 2000; Sejvar et al., 2011), we therefore assume that the age-group-specific distribution of the risk of developing a mild GBS is 17% and a severe GBS is 83% – see Table 4 and 5 (Havelaar 2000a, b). A total of 69% of mild GBS cases are under the age of 50, whereas for severe GBS cases this is only 48%.

The clinical course of GBS is highly variable. Very limited information is available for mildly affected patients. About 50% of the patients recover fully after six months, and the others have an F-score of 1. Most will recover after one year and the remainder will only suffer from minor symptoms (Havelaar, 2000a). We therefore assumed that mild cases will recover fully after one year.

There is a high heterogeneity among the severely-affected GBS patients: 60% of patients are reported to have an F-score of 4 when hospitalised, and approximately 20% of the patients had an F-score of 5 at nadir (Van der Meché, 1992). All patients recovered from intensive care, but after six months, 17% of them still had an F-score of 3 or 4. In a follow-up study the residual symptoms were evaluated up to six years after onset (Bernsen, 1997): only 25% recovered fully, whereas 44% of patients continued to suffer from minor symptoms (F-score=1) and 31% had functional limitations (F-score 2-4). Given that there had been no significant improvement since the acute phase, we assume that 17–31% of severely affected GBS patients would have permanent sequelae; this risk is distributed by age groups, see Table 6 (Havelaar 2000 a;b).

The case fatality rate for GBS ranges from 2–5% (Havelaar, 2000a) to 3.4% in a retrospective study (Van Koningsveld, 2000). However, generally only the severe cases are at risk of dying, therefore the risk is only estimated for these cases (CFR/83% severe cases x 100): 4.1% (2.41–6.02). The case fatality rate is age-dependent (Havelaar, 2000a) and strictly linked to the risk of developing permanent disabilities due to GBS; therefore, we apply the same age-group distribution as the risk of dying, see Table 6).

Model input summary

Table 1. Transition probabilities used in the outcome tree

Health outcome<br>(Health state)	Distribution of health states in health outcome	Transition probability	Source/assumption
Symptomatic infection<br>(Uncomplicated)<br>(Complicated, GP)<br>(Complicated, hosp)	 76%<br>23%<br>1%		Kemmeren, 2006; Kwong, 2012
Fatal cases following symptomatic infection		0.001–0.05%<br>Age dep. Table 3	Adak, 2002; Cressey & Lake, 2007; Mangen, 2005; Mead, 1999; TESSy 2009-2013
Reactive arthritis		1.7% (0.73–4.4%)	Kemmeren, 2006

Guillain-Barré syndrome<br>(Mild)<br><br>(Severe)	 <br>17%<br>Age dep. Table 4<br><br>83%<br>Age dep. Table 5	0.0015–0.09%	Allos, 1987; Ternhag, 2008; Havelaar 2000a, b
Fatal cases following severe GBS		4.1% (2.41–6.02%)<br>Age dep. Table 6	Koningsveld, 2001; Havelaar, 2000a<br>Assuming only severe cases are fatal
Permanent disability following GBS		17–31%<br>Age dep. Table 6	Havelaar, 2000a, b<br>Assuming only severe cases

Table 2. Disability weights and duration

Health outcome (Health state)	Disability Weight (DW) (Haagsma, 2015)		Duration	
	DW	Label	In years	Source/assumption

Symptomatic infection<br><br>(Uncomplicated)<br>(Complicated, GP)<br>(Complicated, hosp)	 <br><br>0.073 (0.061–0.092)<br>0.149 (0.12–0.182)<br>0.239 (0.202–0.285)	 <br><br>Diarrhoea, mild<br>Diarrhoea, moderate<br>Diarrhoea, severe	 <br><br>0.009<br>0.027<br>0.039	Food Standard Agency, 2000; Mangen, 2004, 2005
Reactive arthritis	0.344 (0.3-0.391)	Musculoskeletal problems, generalized, moderate	0.131-0.608	Hannu, 2002; Kemmeren, 2006
Guillain-Barré syndrome<br>(Mild)<br>(Severe)	 <br>0.053 (0.042-0.064)<br><br>0.520 (0.465-0.581)	 <br>Motor impairment, moderate<br><br>Spinal cord lesion at neck level (treated)	 <br>1<br>1	Havelaar, 2000a, b
Permanent disability following GBS	0.421 (0.377-0.477)	Motor impairment, severe	Remaining life expectancy	Van der Meché, 1992; Bernsen, 1997

Table 3. Age-group distribution of the case fatality rate (0.001–0.05%)

Age groups	%
0	0.54
1-4	1.09
5-9	3.26
10-14	1.63
15-19	0.54
20-24	4.35
25-29	5.98
30-34	1.63
35-39	3.26
40-44	3.80
45-49	3.80
50-54	5.43

55-59	5.98
60-64	5.98
65-69	8.15
70-74	6.52
75-79	11.96
80-84	11.96
>85	14.13
All ages	100.00

Table 4. Age distribution mild GBS

Age	%
0	0.63
01-04	5.02
05-09	2.51
10-14	1.25
15-19	6.27
20-24	6.90

25-29	10.04
30-34	9.41
35-39	9.41
40-44	8.78
45-49	8.78
50-54	5.17
55-59	4.82
60-64	4.13
65-69	5.51
70-74	5.17
75-79	4.13
80-84	0.69
85+	1.38
Total	100

Table 5. Age distribution – severe GBS

Age	%
0	0.44
01-04	3.49
05-09	1.75
10-14	0.87
15-19	4.36
20-24	4.80
25-29	6.98
30-34	6.55
35-39	6.55
40-44	6.11
45-49	6.11
50-54	8.67
55-59	8.09
60-64	6.93
65-69	9.24
70-74	8.67
75-79	6.93

80-84	1.16
85+	2.31

Total	100

Table 6. Age distribution permanent GBS and case fatality rate

Age	%
0	0.00
01-04	0.00
05-09	0.00
10-14	0.00
15-19	0.00
20-24	1.56
25-29	1.56
30-34	1.56
35-39	1.56
40-44	2.08
45-49	2.08
50-54	2.08
55-59	6.25
60-64	6.25
65-69	6.25
70-74	18.75
75-79	25.00
80-84	18.75
85+	6.25
Total	100.00

References

Adak GK, Long SM, O'Brien SJ (2002). Trends in indigenous foodborne disease and deaths, England and Wales: 1992 to 2000. Gut 2002. 51:832- 841.

Allos BM. (1997). Association between Campylobacter infection and Guillain-Barré syndrome. J Infect Dis 1997.176(Suppl 2): S125-S128.

Bernsen RA, Jacobs HM, de Jager AE, van der Meché FGA (1997). Residual health status after Guillain-Barré syndrome. J. Neurol Neurosurg Psychiatry 1997. 62:637-640.

Buzby JC, Roberts T, Lin JCT, MacDonald JM (1996). Bacterial Foodborne Disease – Health care costs & Productivity losses. Washington, USA: Economic Research service, USDA. Agricultural Economic Report No 741.

Cressey P J and Lake R (2007). Risk ranking: Estimates of the burden of foodborne disease for New Zealand. Institute of Environmental Science & Research Limited; Christchurch Science Center.

Food Standards Agency (2000). A Report of the Study of Infectious Intestinal Disease in England. Food Standards Agency London: HMSO.

Haagsma JA, Havelaar AH, Janssen BM, Bonsel GJ. Disability Adjusted Life Years and minimal disease: application of a preference-based relevance criterion to rank enteric pathogens. Popul Health Metr 2008;6:7.

Haagsma JA, van der Zanden BP, Tariq L, van Pelt W, van Duynhoven YTHP, Havelaar AH (2009). Disease burden and costs of selected foodborne pathogens in the Netherlands, 2006. Bilthoven: National Institute of Public Health and Environment. RIVM rapport 330331001. Available online at: www.rivm.nl/bibliotheek/rapporten/330331001.pdf

Haagsma JA, Siersema PD, De Wit NJ, Havelaar AH. Disease burden of post-infectious irritable bowel syndrome in the Netherlands. Epidemiol Infect 2010.138(11):1650-6.

Haagsma JA, Maertens de Noordhout C, Polinder S, Vos T, Havelaar AH, Cassini A, Devleesschauwer B, Kretzschmar ME, Speybroeck N, Salomon JA. Assessing disability weights based on the responses of 30,660 people from four European countries. Population Health Metrics 2015; 13: 10

Hannu TL, Mattila H, Rautelin P, Pelkonen P, Lahdenne A, Siitonen M et al. Campylobacter-triggered reactive arthritis: a population-based study. Rheumatology 2002. 41(3):312-318.

Hannu TL, Kauppi M, Tuomala M, Laaksonen I, Klemets P, Kuusi M. Reactive arthritis following an outbreak of Campylobacter jejuni infection. J Rheumatol 2004. 31(3):528-30.

Havelaar AH, de Wit MAS, van Koningsveld R, van Kempen E (2000a). Health burden in the Netherlands due to infection with thermophilic Campylobacter spp. Epidemiol Infect 2000. 125(3):505-22.

Havelaar AH, de Wit MAS, van Koningsveld R (2000b). Health burden in the Netherlands (1990-1995) due to infection with thermophilic Campylobacter species. Bilthoven, the Netherlands: National Institute for Public Health and the Environment. Report no. 284550004.

Havelaar AH, Haagsma JA, Mangen MJJ, Kemmeren JM, Verhoef LPB, Vijgen SMC, et al. (2012a). Disease burden of foodborne pathogens in the Netherlands, 2009. International Journal of Microbiology in press.

Havelaar AH, Ivarsson S, Löfdahl M, Nauta MJ (2012b). Estimating the true incidence of campylobacteriosis and salmonellosis in the EU. Submitted.

Helms M, Simonsen J, Molbak K. Foodborne bacterial infection and hospitalization: a registry-based study. Clin Infect Dis 2006.42(4):498-506.

Hughes RAC and Rees JH (1997). Clinical and epidemiologic features of Guillain-Barré syndrome. Journal of infectious diseases 1997. 176 (suppl 2): S92-S98.

Jacobs BC, Rothbarth PH, van der Meché FG, Herbrink P, Schmitz PI, de Klerk MA, et al. (1998). The spectrum of antecedent infections in Guillain-Barré syndrome: a case-control study. Neurology 1998. 51(4):1110-1115.

Jess T, Simonsen J, Nielsen NM, Jørgensen KT, Bager P, Ethelberg S et al. (2011). Enteric Salmonella or Campylobacter infections and the risk of inflammatory bowel disease. Gut 2011;60(3):318-324.

Johnsen K, Ostensen M, Melbeye AC, Melby K (1983). HLA-B27-negative arthritis related to Campylobacter jejuni enteritis in three children and two adults. Acta Med Scand 1983. 214(2): 165-168.

Kemmeren JM, Mangen MJJ, van Duynhoven YTHP, Havelaar AH. (2006). Priority setting of foodborne pathogens: disease burden and costs of selected enteric pathogens. Bilthoven: National Institute of Public Health and Environment: RIVM rapport 330080001. Available online at: www.rivm.nl/bibliotheek/rapporten/330080001.pdf

Keat A. (1983). Reiter's syndrome and reactive arthritis in perspective. N Engl J Med 1983. 309(26):1606-15.

Kosunen TU, Kauranen O, Martio J, Pitkanen T, Ponka A, Hortling L, et al. (1980). Reactive arthritis after campylobacter jejuni enteritis in patients with HLA-B27. Lancet 1980. 1 (8181):1312-3.

Kwong JC, Ratnasingham S, Campitelli MA, Daneman N, Deeks SL, et al. (2012) The Impact of Infection on Population Health: Results of the Ontario Burden of Infectious Diseases Study. PLoS ONE 7(9): e44103. doi:10.1371/journal.pone.0044103

Lake RJ, Cressey PJ, Campbell DM, Oakley E (2010). Risk ranking for foodborne microbial hazards in New Zealand: burden of disease estimates. Risk Analysis 2010. 30(5):743-52.

Locht H, Krogfelt KA (2002). Comparison of rheumatological and gastrointestinal symptoms after infection with Campylobacter jejuni/coli and enterotoxigenic Escherichia coli. Ann Rheum Dis 2002.61(5): 448-52.

Mangen MJJ, Havelaar AH, de Wit GA. (2004). Campylobacteriosis and sequelae in the Netherlands - Estimating the disease burden and the cost-of-illness. Bilthoven: National Institute for Public Health and the Environment; Report nr. 250911004. Available online at: www.rivm.nl/bibliotheek/rapporten/250911004.pdf

Mangen MJJ, Havelaar AH, Bernsen RAJAM, van Koningsveld R, de Wit GA (2005). The costs of human Campylobacter infections and sequelae in the Netherlands: A DALY and cost-of-illness approach. 2005. Acta Agriculturae Scandinavica Section C Food Economics 2005. 2(1):35-51.

McCarthy N, Giesecke J (2001). Incidence of Guillain-Barre syndrome following infection with Campylobacter jejuni. Am J Epidemiol 2001. 153 (6): 610-614.

McGrogan A, Madle GC, Seaman HE, de Vries CS (2009). The epidemiology of Guillain-Barré syndrome worldwide. A systematic literature review. Neuroepidemiology 2009. 32(2):150-163.

Mead PS, Slutsker L, Dietz V, McCaig LF, Bresee JF, Shapiro C, et al. (1999). Food-related illness and death in the United States. Emerg Infect Dis 1999.5(5): 607-625.

Melse JM, Kramers PGN (1998). Berekening van de ziektelast in Nederland. Achtergronddocument bij VTV-1997; deel III, hoofdstuk 7. Bilthoven; Report nr. 431501028.

Mishu B, Ilyas AA, Koski CL, Vriesendorp F, Cook SD, Mithen FA, et al. (1993). Serological evidence of previous Campylobacter jejuni infection in patients with the Guillain-Barré Syndrome. Ann Intern Med 1993. 118(12): 947-953.

Nachamkin I, Allos BM, Ho T. (1998). Campylobacter species and Guillain-Barré syndrome. Clin Microbiol Rev 1998. 11(3): 555-567.

Nagpal S, Benstead T, Shumak K, Rock G, Brown M, Anderson DR (1999). Treatment of Guillain-Barré syndrome: a cost-effectiveness analysis. J. Clin Apheresis 1999. 14(3):107-113.

Poropatich KO, Walker CL, Black RE (2010). Quantifying the association between Campylobacter infection and Guillain-Barré syndrome: a systematic review. J. Health Popul Nutr; 2010. 28(6):545-52.

Sedano MJ, Calleja J, Canga E, Berciano J (1994). Guillain-Barré syndrome in Cantabria, Spain. An epidemiological and clinical study. Acta Neurol Scand 89(4): 287-292.

Sejvar JJ, Baughman AL, Wise M, Morgan OW (2011). Population incidence of Guillain-Barré syndrome: a systematic review and meta-analysis. Neuroepidemiology; 2011 36(2):123-133.

Skirrow MB, Jones DM, Sutcliffe E, Benjamin J (1993). Campylobacter bacteraemia in England and Wales, 191-91. Epidemiology and Infection 110 (3): 49-57.

Tam CC, Rodrigues IC, Viviani L, Dodds JP, Evans MR, Hunter PR et al. Longitudinal study of infectious intestinal disease in the UK (IID2 study): incidence in the community and presenting to general practice. Gut. 2012 Jan;61(1):69-77

Ternhag A, Törner A, Svensson A, Ekdahl K, Giesecke J (2008). Short- and long-term effects of bacterial gastrointestinal infections. Emerg Infect Dis 2008.14 (1):143-8.

Van der Meché FGA, Schmitz PIM and Dutch Guillain-Barré Study Group (1992). A randomized trial comparing intravenous immune globulin and plasma exchange in Guillain-Barré syndrome.N Engl. J. Med. 1992 326:1123-1129.

Van Koningsveld R, van Doorn PA, Schmitz PIM, Ang CW, van der Meché FGA (2000). Mild forms of Guillain-Barré syndrome in an epidemiological survey in the Netherlands. Neurology 54: 620-625.

Van Koningsveld R. (2001). Epidemiological and clinical aspects of the Guillain-Barré syndrome. Rotterdam, the Netherlands: Erasmus University Rotterdam. PhD-Thesis.

WHO (2004). Global Burden of Disease 2004 Update: Disability weights for diseases and conditions. Geneva: WHO. Available online at: http://www.who.int/healthinfo/global_burden_disease/GBD2004_DisabilityWeights.pdf

Yu DTY, Thomson GTD (1994). Clinical, epidemiological and pathogenic aspects of reactive arthritis. Food-Microbiology 1994. 11(2):97-108.

Chlamydia

Chlamydia trachomatis is a bacterium that causes a sexually transmitted infection (STI). WHO estimates a global annual incidence of about 90 million cases. Chlamydia trachomatis affects both women and men and can cause severe harm to the reproductive system of women. Additionally, children born to infected mothers are at high risk of developing severe complications (e.g. ophthalmia neonatorum, pneumonia). C. trachomatis has various serovars with different transmission modes and consequences. Serovars A, B, Ba and C, often transmitted by close eye- to-eye contact, cause ocular trachoma and are responsible for about 7–9 million cases of blindness (Stamm, 2005). Serovars D–K, responsible for genital infections, are associated with various adverse health outcomes in both men and women (Carey & Beagley, 2010). Serovars L1, L2 and L3 cause Lymphogranuloma venerum, a systemic STI mainly observed in the high-risk group of men having sex with men (MSM) (Martin- Iguacel, 2010). For the current outcome trees only serovars D–K responsible for genital infection are taken into consideration.

C. trachomatis mostly affects the young and sexually-active population with a female-male sex ratio of 1:0.7 (in tested individuals) (ECDC, 2014a). The genito-urinary infections present different disease patterns in the female and male hosts.

The asymptomatic infection poses serious threats to the health of the population as asymptomatic carriers represent a pool for new infections, and asymptomatic infections are associated with the risk of developing severe sequelae.

Rates of asymptomatic cases reported in literature vary widely. More than 50% of the infections due to C. trachomatis in males do not produce any symptoms or present a mild symptomatic illness (van de Laar & Morre, 2007). In a study of male army recruits, 85.6% of men testing positive for Chlamydia reported no symptoms (Cecil, 2001). Comparable rates were also reported by McKay and colleagues, with 88% of infected men being asymptomatic (McKay, 2003). Long-term sequelae due to chronic asymptomatic infections in men are still under discussion, but the pool of asymptomatic C. trachomatis carriers poses a serious threat to women's health due to continuous transmission and re-infection. Gaydos and Quinn refer to a percentage of asymptomatic male cases above 50%, in line with the above-mentioned estimates (Gaydos & Quinn, 2012).

Genital infections in women may present with short-term acute symptoms of cervicitis and urethritis (Stamm, 2005). Women also face a high number of asymptomatic infections. In total, 70–90% of all female and 50–88% of all male chlamydial infections do not present any symptoms (Stamm, 2005; Gaydos, 1998; Kalwij, 2010). Quinn and colleagues noted that around 79% of women with a Chlamydia infection attending a STI clinic were asymptomatic (Quinn, 1996). Clinical textbooks report a range of 70–90% of female cases being asymptomatic (Stamm, 2005; Gaydos & Quinn, 2012).

For the model we decided to use a range of 70–90% for the asymptomatic proportion (Stamm, 2005; Gaydos & Quinn, 2012) for female and 50– 88% for male cases (Stamm, 2005; Gaydos, 1998; Kalwij, 2010).

Health outcomes associated with chlamydial infection

Genital infection in men

Urethritis: with an incubation period of 7–14 days, urethritis causes symptoms of dysuria and urethral discharge (Stamm, 2005). We selected a range of 12–50% of infected men to represent symptomatic cases developing non-gonococcal urethritis (NGU) (Carey & Beagley, 2010; McKay, 2003).

Epididymitis: epididymitis is an acute inflammation of the epididymis (Carey & Beagley, 2010). The symptoms are oligospermia during the acute phase, swollen epididymis (and/or testicles), and dysuria. Fever and chills may occur in some cases. The association between epididymitis and future (in)fertility is an ongoing debate in research with no clear indication (Stamm, 2005).

Proctitis and proctocolitis: this clinical picture is most common in the MSM community. The classic symptoms are rectal pruritus, -pain and - bleeding. Fever often accompanies the initial proctitis and proctocolitis (Stamm, 2005; Carey & Beagley, 2010). This health outcome was not considered in the model due to lack of information.

Reactive arthritis: a further clinical picture is sexually-acquired reactive arthritis occurring as an acute aseptic arthritis or presenting as Reiter's syndrome. Reiter's syndrome includes symptoms of arthritis, conjunctivitis, urethritis and skin lesions (Stamm, 2005; Keat, 1983).

Genital infection in men can also include chronic pelvic pain. However, due to lack of information we decided not to include it in the model (Haggerty, 2010).

Genital infection in women

Urethritis/cervicitis

The acute form of C. trachomatis infection in women is urethritis and/or cervicitis. The majority of cases of both urethritis and cervicitis are asymptomatic, but can lead to severe sequelae (Low, 2007).

Pelvic inflammatory disease (PID)

Both symptomatic and asymptomatic infections can lead to serious consequences. Pelvic inflammatory disease is a commonly reported health outcome of a chlamydial infection. The literature shows very heterogeneous patterns regarding the transition probabilities from acute infection to PID. Carey and Beagley state that 12–50% of women infected with C. trachomatis develop PID (Carey & Beagley, 2010). In other literature the risk of PID after lower genital tract infection with Chlamydia varied from 0 to 30% (Risser & Risser, 2007) and from 0 to 72% (Boeke, 2005). Cates and Wasserheit reported that 40% of women with an untreated C. trachomatis infection develop PID (Cates & Wasserheit, 1991). Van Valkengoed and colleagues reported that complications of Chlamydia trachomatis infections are overestimated in the literature. They found five Cost Effectiveness Analyses (CEA) using decision trees to estimate the effect of screening programmes (Van Valkengoed, 2004). In these studies the estimates of the probability of developing PID after infection varied from 25 to 80%. ECDC has undertaken a systematic literature review and found a risk of developing PID from chlamydial infections of 9% (4–19%) (ECDC, 2014b).

Acute PID with pelvic pain, lasting for about 15 days, and silent PID with no or mild symptoms can cause severe long-term sequelae (Carey & Beagley, 2010; Westrom, 1980).

The estimated risk of tubal infertility as a sequelae of PID varies between 10–20% (Carey & Beagley, 2010; Lan, 1995; Land, 2010). Land and colleagues estimated the risk of tubal infertility after asymptomatic Chlamydia infection to be around 0.07% (Land, 2010). The risk of tubal infertility was found to be dependent on the course of infection (mild vs. severe) and the frequencies of re-infection (e.g. after three episodes of PID the risk is five-fold compared to a single episode.) ECDC's systematic review found that 16% of women with PID will develop infertility (ECDC, 2014b), which applies to women of reproductive age.

In total, 7–9% of pregnant women develop ectopic pregnancy after PID (Lan, 1995). Around 15% of women with previous PID develop chronic pelvic pain (Rogstad, 2008). Tubo-ovarian abscesses (tubal pathology) incur a risk of 7–16% for women who have previously had PID (Kottmann, 1995). The risk of cervical neoplasia is still under debate due to the fact that most cervical neoplasia are due to human papilloma virus (HPV) (Stamm, 2005).

Based on registration data from Amsterdam it was estimated that 0.07% and 0.02% of women exposed to chlamydia infection develop ectopic pregnancy and tubal factor infertility, respectively (Van Valkengoed, 2004).

Perinatal infections

Perinatal chlamydia may complicate as conjunctivitis (ophthalmia neonatorum) and neonatal pneumonia. We considered the ONBoID study for the input parameters which estimated that 15% of cases would develop ophthalmia neonatorum and 16% neonatal pneumonia (Kwong 2012). Assuming that in EU/EEA Member States all notified cases will have had symptoms, we used the same proportion: 48.39% are affected by ophthalmia and 51.61% will present pneumonia.

Outcome-tree parameters

Male outcome tree

For the male outcome tree a minimum of 50% and maximum of 88% was estimated as the percentage of asymptomatic cases (Carey & Beagley, 2010; McKay, 2003). The probability of developing epididymitis from symptomatic infections (10%) was taken from the World Health Organization STD Burden of Disease Study by Gerbase and colleagues (Gerbase, 2000). For asymptomatic infections a probability of 1–4% was taken from the cost effectiveness analysis of Welte and colleagues (Welte, 2001). Data on sexually acquired reactive arthritis (1% of symptomatic urethritis) and the resulting Reiters' syndrome (33% of reactive arthritis) were taken from a clinical text book (Stamm, 2005).

Female outcome tree

For the percentage of asymptomatic cases a range of 70–90% was included in the model (Stamm, 2005; Gaydos & Quinn, 2012; Gaydos, 1998; Kalwij, 2010; Stamm, 1999).

For the development of PID, estimates are included from the systematic review conducted by ECDC for the minimum (4%) (Van Valkengoed, 2004), maximum (19%) and most likely values (9%) (ECDC, 2014b).

Although ectopic pregnancy and tubal infertility normally are a consequence of PID, experts considered that evidence relating these outcomes directly to the chlamydia infection was stronger than that relating it to PID (mainly because of the heterogeneous definition of PID). The probability of developing ectopic pregnancy and tubal infertility after chlamydia infection is set to 0.07% and 0.02% respectively (Van Valkengoed, 2004). The probability of dying due to ectopic pregnancy was set to 0.038%, based on the study from Goldner (Goldner, 1993).

The risk of moving from PID to chronic pelvic pain was set at 18–75% and from PID to tubo-ovarian abscess at 0.8% (ECDC, 2014b; Ness, 2002, Soper 2010).

We decided to set the case fatality proportion for abscesses that have not ruptured to zero. Current mortality proportions for patients with ruptured abscesses are not reported in the literature; data from the 1960s suggested a mortality proportion ranging from 1.7 to 3.7 percent (Pedowitz, 2004; Paik, 2006). Due to the fact that these figures come from old studies and that diagnostics and treatment have significantly improved, we decided not to include the risk of dying from tubo-ovarian abscess.

Model input summary

Table 1. Transition probabilities and distributions used in the outcome tree

Health outcome (Health state)	Distribution of health states within health outcome	Transition probability	Source/assumption
Men			
Symptomatic infection		12–50%	Carey & Beagley, 2010; McKay, 2003
Epididymitis following symptomatic infection		10%	Gerbase, 2000
Reactive arthritis		1%	Stamm, 2005
(Mild)	67%		Stamm, 2005
(Severe)	33%		Stamm, 2005
Epididymitis following asymptomatic infection		1–4%	Gerbase, 2000; Welte, 2001

Women<br>Symptomatic infection		10–30%	Stamm, 1999; Stamm, 2005; Gaydos & Quinn, 2012; Gaydos, 1998; Kalwij, 2010
Pelvic inflammatory disease (PID)		9% (4–19%)	ECDC, 2014b
Tubo-ovarian abscess from PID		0.8%	Ness, 2002
Chronic pelvic pain after PID		18–75%	ECDC, 2014b; Soper 2010
Ectopic pregnancy		0.07%<br>Age dep. See Table 4	van Valkengoed, 2004<br>Female reproductive age 15-49
Tubal Infertility		0.02%<br>Age dep. See Table 4	Land, 2010; ECDC, 2014b<br>Female reproductive age 15-49
Fatal cases following ectopic pregnancy		0.038%	Goldner, 1993
Perinatal			
Symptomatic infection<br>(Neonatal pneumonia) (Ophthalmia neonatorum)	48.39%<br>51.61%		Kwong, 2012<br>Assuming that all reported cases have symptoms, we used the same proportion

Table 2. Disability weights and duration

Health outcome (Health state)	Disability Weight (DW) (Haagsma, 2015)		Duration	
	DW	ECDC European Disability Weight Project (2014)	In years	Source

Men				
Symptomatic infection	0.007 (0.005–0.01)	Infectious disease, acute episode, mild	0.02	Trojian, 2009
Epididymitis	0.176 (0.143–0.208)	Epididymo-orchitis	0.04	Murray, 1996
Reactive arthritis<br>(Mild)<br><br>(Severe)	 0.344 (0.3-0.391)<br><br>0.518 (0.457–0.576)	 Musculoskeletal problems, generalised, moderate<br>Musculoskeletal problems, generalised, severe	 0.13–0.28<br><br>0.41	 Özgül, 2006; Hannu, 2002<br><br>Miehle, 2003
Women				
Symptomatic infection	0.007 (0.005–0.01)	Infectious disease, acute episode, mild	0.03	Murray, 1996
Pelvic inflammatory disease (PID)	0.018–0.310	Abdominopelvic problem, mild to severe	0.04	Westrom, 1980
Tubo-ovarian abscess	0.31 (0.262–0.355)	Abdominopelvic problem, severe	0.01	Goharkhay, 2007; Teisala, 1990
Chronic pelvic pain	0.018–0.123	Abdominopelvic problem, mild to moderate	2.8	Sharma, 2011
Ectopic pregnancy	0.31 (0.262–0.355)	Abdominopelvic problem, severe	0.08	Murray, 1996
Tubal infertility	0.007 (0.005–0.01)	Infertility, secondary	See Table 3	Female reproductive age 15-49 (See Table 4)

Perinatal				
Neonatal pneumonia	0.125 (0.104–0.152)	Infectious disease, acute episode, severe	0.038	Zar, 2005<br><br>Assuming two weeks of treatment
Ophthalmia neonatorum	0.015 (0.011–0.019)	Conjunctivitis without corneal scar	0.038	American Academy of Pediatrics, 2012.<br><br>Assuming two weeks of treatment

Table 3. Duration of tubal infertility (female outcome tree)

Age	Duration in years
15–19	32
20–24	27
25–29	22
30–34	17
35–39	12
40–44	7
45–49	2

Table 4. Age-group risk (only reproductive age)

Age	%
0–14	0
15–49	100
≥50	0

References

American Academy of Pediatrics. Chlamydia trachomatis. In: Pickering LK, editor. Red book: 2012. Report of the Committee on Infectious Diseases. Elk Grove Village, IL: American Academy of Pediatrics; 2012. pp. 276–81.

Boeke AJ, van Bergen JE, Morre SA, van Everdingen JJ: [The risk of pelvic inflammatory disease associated with urogenital infection with Chlamydia trachomatis; literature review]. Ned Tijdschr Geneeskd 2005, 149:878-884.

Carey AJ, Beagley KW: Chlamydia trachomatis, a hidden epidemic: effects on female reproduction and options for treatment. Am J Reprod Immunol 2010, 63:576-586.

Cates W Jr. Wasserheit JN: Genital chlamydial infections: epidemiology and reproductive sequelae. Am J Obstet Gynecol 1991, 164:1771-1781.

Cecil JA, Howell MR, Tawes JJ, Gaydos JC, McKee KT, Quinn TC, et al. Features of Chlamydia trachomatis and Neisseria gonorrhoeae Infection in Male Army Recruits. Journal of Infectious Diseases 2001, 184:1216-1219.

European Centre for Disease Prevention and Control. Sexually transmitted infections in Europe 2012: ECDC; 2014a.

European Centre for Disease Prevention and Control. Chlamydia control in Europe: literature review. Stockholm: ECDC; 2014b.

Gaydos CA, Howell MR, Pare B, Clark KL, Ellis DA, Hendrix RM, et al. Chlamydia trachomatis Infections in Female Military Recruits. New England Journal of Medicine 1998, 339:739-744.

Gaydos CA, Quinn TC. Chlamydial infections. In Harrison's Principles of Internal Medicine. Volume 2. 18th Edition. Edited by Longo D, Fauci A, Kasper D, Hauser S, Jameson J, Loscalzo J. New York: McGraw Hill; 2012: 1421-1432

Gerbase A, Stein C, Levison J, Htun Y: Global burden of sexually transmitted diseases (excluding HIV) in the year 2000. In: Global burden of sexually transmitted diseases (excluding HIV) in the year 2000 (Ed.). World Health Organization; 2000.

Goldner TE, Lawson HW, Xia Z, Atrash HK. Surveillance for ectopic pregnancy—United States, 1970–1989. MMWR CDC Surveill Summ. 1993;42:73–85.

Goharkhay N, Verma U, Maggiorotto F: Comparison of CT- or ultrasound-guided drainage with concomitant intravenous antibiotics vs. intravenous antibiotics alone in the management of tubo-ovarian abscesses. Ultrasound Obstet Gynecol 2007, 29:65-69.

Haggerty CL, Gottlieb SL, Taylor BD, Low N, Xu F, Ness RB. Risk of sequelae after Chlamydia trachomatis genital infection in women. J Infect Dis 2010, 201 Suppl 2:S134-155.

Haagsma JA, Maertens de Noordhout C, Polinder S, Vos T, Havelaar AH, Cassini A, Devleesschauwer B, Kretzschmar ME, Speybroeck N, Salomon JA. Assessing disability weights based on the responses of 30,660 people from four European countries. Population Health Metrics 2015; 13: 10

Hammerschlag MR, Chandler JW, Alexander ER, English M, Koutsky L. Longitudinal studies on chlamydial infections in the first year of life. The Pediatric Infectious Disease Journal 1982, 1:395-401.

Hammerschlag MR. Chlamydial infections. J Pediatr 1989, 114:727-734.

Hannu TL, Mattila H, Rautelin P, Pelkonen P, Lahdenne A, Siitonen M et al. (2002) Campylobacter-triggered reactive arthritis: a population- based study. Rheumatology 41(3): 312-318.

Kalwij S, Macintosh M, Baraitser P. Screening and treatment of Chlamydia trachomatis infections. BMJ 2010, 340.

Keat A, Thomas BJ, Taylor-Robinson D. Chlamydial infection in the aetiology of arthritis. Br Med Bull 1983, 39:168-174.

Kottmann LM. Pelvic inflammatory disease: clinical overview. J Obstet Gynecol Neonatal Nurs 1995, 24:759-767.

Kwong JC, Ratnasingham S, Campitelli MA, Daneman N, Deeks SL, et al. (2012) The Impact of Infection on Population Health: Results of the Ontario Burden of Infectious Diseases Study. PLoS ONE 7(9): e44103. doi:10.1371/journal.pone.0044103

Lan J, van den Brule AJ, Hemrika DJ, Risse EK, Walboomers JM, Schipper ME, et al. Chlamydia trachomatis and ectopic pregnancy: retrospective analysis of salpingectomy specimens, endometrial biopsies, and cervical smears. J Clin Pathol 1995, 48:815-819.

Land JA, Van Bergen JE, Morre SA, Postma MJ. Epidemiology of Chlamydia trachomatis infection in women and the cost-effectiveness of screening. Hum Reprod Update 2010, 16:189-204.

Low N, McCarthy A, Macleod J, Salisbury C, Campbell R, Roberts TE, et al. Epidemiological, social, diagnostic and economic evaluation of population screening for genital chlamydial infection. Health Technol Assess 2007, 11:iii-iv, ix-xii, 1-165.

Martin-Iguacel R, Llibre JM, Nielsen H, Heras E, Matas L, Lugo R, et al. Lymphogranuloma venereum proctocolitis: a silent endemic disease in men who have sex with men in industrialised countries. Eur J Clin Microbiol Infect Dis 2010, 29:917-925.

McKay L, Clery H, Carrick-Anderson K, Hollis S, Scott G. Genital Chlamydia trachomatis infection in a subgroup of young men in the UK. Lancet 2003, 361:1792.

Miehle W. Medikamentöse Therapie rheumatischer Erkrankungen. Stuttgart: Georg Thieme Verlag; 2003.

Murray CJL, Lopez AD. The Global Burden of Disease - A comprehensive assessment of mortality and disability from diseases, injuries and risk factors in 1990 and projected to 2020. Cambridge: Harvard School of Public Health on behalf of the World Health Organization and the World Bank; 1996.

Ness RB, Soper DE, Holley RL, Peipert J, Randall H, Sweet RL, Sondheimer SJ, Hendrix SL, Amortegui A, Trucco G, Songer T, Lave JR, Hillier SL, Bass DC, Kelsey SF (2002) Effectiveness of inpatient and outpatient treatment strategies for women with pelvic inflammatory disease: results from the Pelvic Inflammatory Disease Evaluation and Clinical Health (PEACH) Randomized Trial. Am J Obstet Gynecol 186: 929-937.

Özgül A, et al. (2006). Clinical presentations of chlamydial and non-chlamydial reactive arthritis. Rheumatology International 26(10): 879-885.

Paik CK, Waetjen LE, Xing G, et al. Hospitalizations for pelvic inflammatory disease and tuboovarian abscess. Obstet Gynecol 2006; 107:611.

Pedowitz P, Bloomfield Rd. Ruptured adnexal abcess (tuboovarian) with generalized peritonitis. Am J Obstet Gynecol 1964; 88:721.

Peipert JF. Clinical practice. Genital chlamydial infections. N Engl J Med 2003, 349:2424-2430.

Quinn TC, Gaydos C, Shepherd M, Bobo L, Hook EW, Viscidi R, et al. Epidemiological and microbiological correlates of Chlamydia trachomatis infection in sexual partnerships. JAMA: Journal of the American Medical Association 1996, 276:1737-1742.

Risser WL, Risser JM. The incidence of pelvic inflammatory disease in untreated women infected with Chlamydia trachomatis: a structured review. Int J STD AIDS 2007, 18:727-731.

Rogstad K. Complications in the female and their management. In: International handbook of Chlamydia. 3rd Edition. Edited by Moss T. Haslemere, UK: Alden Press; 2008: 111-121

Salomon JA, Haagsma JA, Davis A, Maertens de Noordhout C, Polinder S, Havelaar AH, et al. Disability weights for the Global Burden of Disease 2013 study. The Lancet Global Health. [Accepted, in press.]

Sharma D, Dahiya K, Duhan N, Bansal R. Diagnostic laparoscopy in chronic pelvic pain. Arch Gynecol Obstet 2011, 283:295-297.

Soper DE. Pelvic inflammatory disease. Obstetrics and Gynecology, vol. 116, no. 2, part 1, pp. 419–428, 2010.

Stamm WE, Jones RB, Batteiger BE. Chlamydia trachomatis (Trachoma, Prinatal Infections, Lymphogranuloma Venerum and other genital Infections). In: Principles and practice of infectious diseases. Volume 2, 6th Edition. Edited by Mandel GL, Bennet JE, Dolin R. Pennsylvania: Elsevier Inc.; 2005: 2239-2255

Stamm WE. Chlamydia trachomatis infections of the adult. In: Sexually transmitted diseases. 3rd Edition. Edited by Holmes KK, Sparling PF, Mardh PA. New York: McGraw Hill; 1999:407–422

Trojian TH, Lishnak TS, Heiman D. Epididymitis and orchitis: an overview. Am Fam Physician 2009, 79:583-587.

Teisala K, Heinonen PK, Punnonen R. Transvaginal ultrasound in the diagnosis and treatment of tubo-ovarian abscess. Br J Obstet Gynaecol 1990, 97:178-180.

van de Laar MJ, Morre SA. Chlamydia: a major challenge for public health. Euro Surveill 2007, 12:E1-2.

van Valkengoed IG, Morre SA, van den Brule AJ, Meijer CJ, Bouter LM, Boeke AJ. Overestimation of complication rates in evaluations of Chlamydia trachomatis screening programmes--implications for cost-effectiveness analyses. Int J Epidemiol 2004, 33:416-425.

Welte R, Jager H, Postma MJ. Cost-effectiveness of screening for genital Chlamydia trachomatis. Expert Rev Pharmacoecon Outcomes Res 2001, 1:145-156.

Westrom L. Incidence, prevalence, and trends of acute pelvic inflammatory disease and its consequences in industrialized countries. Am J Obstet Gynecol 1980, 138:880-892.

Zar HJ. Neonatal chlamydial infections: prevention and treatment. Pediatric Drugs, vol. 7, no. 2, pp. 103–110, 2005

Cryptosporidiosis

Acute gastroenteritis associated with cryptosporidiosis in humans is in most cases self-limiting and symptoms disappear within a few days or weeks, but in very small number of cases the disease can be fatal.

We assumed that only a small proportion of cases (0.150%) experience the disease as more severe and complicated (Vijgen, 2007).

The average duration of the uncomplicated, mild disease is 3.5 days and 7–18.4 days for the complicated form (Vijgen, 2007).

The case fatality proportion was found to be 0.0042% (Vijgen, 2007), in line with 0.005% found in other studies (Mead, 1999). Mortality from acute gastroenteritis was assumed to be age-dependent and was redistributed according to the age-group-distributed cryptosporidiosis and giardiasis case fatality proportion reported to TESSy between 2009 and 2013 (see Table 3). This table is based on all TESSy notified cases from EEA Member States except Bulgaria, Poland (reporting only aggregate data), Austria, Czech Republic, Iceland, Luxembourg, Malta, Norway, Romania, Slovenia and Slovakia (because the very low incidence reported seems to indicate low sensitivity of the surveillance system).

Cryptosporidiosis can become chronic in immunocompromised persons, especially those with AIDS (Caccio and Pozio, 2006; Call, 2000; Pozio, 1997). However, several studies showed that AIDS-related cryptosporidiosis can be cured following successful antiretroviral therapy (Miao, 2000; Maggi, 2000; Foudraine, 1998).

Model input summary

Table 1. Transition probabilities and distributions used in the outcome tree

Health outcome<br><br>(Health state)	Distribution of health states<br><br>in health outcome	Transition probability	Source/assumption
Symptomatic infection			Vijgen, 2007
Uncomplicated)	99.85%		
Complicated)	0.15%		
Fatal cases following symptomatic<br><br>infection		0.0042%<br><br>Age dependent (Table 3)	Vijgen, 2007;<br><br>TESSy 2009–2013

Table 2. Disability weights and duration

Health outcome<br><br>(Health state)	Disability Weight (DW) (Haagsma, 2015)			Duration	

	DW	Label	In years	Source
Symptomatic infection				Vijgen, 2007
(Moderate)	0.073 (0.061–0.092)	Diarrhoea, mild	0.01	
(Severe)	0.239 (0.202–0.285)	Diarrhoea, severe	0.019–0.05	

Table 3. Age-group redistribution of case fatality proportion due to cryptosporidiosis (0.0042%)

Age groups	%
0	12.50
1–4	6.25
5–9	6.25
10–14	0.00
15–19	0.00
20–24	0.00
25–29	0.00
30–34	6.25

35–39	0.00
40–44	0.00
45–49	6.25
50–54	12.50
55–59	6.25
60–64	6.25
65–69	6.25
70–74	6.25
75–79	18.75
80–84	6.25
>85	0.00
All ages	100.00

References

Caccio SM, Pozio E (2006). Advances in the epidemiology, diagnosis and treatment of cryptosporidiosis. Expert Rev Anti Infect Ther. 4(3):429- 443.

Call SA, Heudebert G, Saag M, Wilcox CM (2000). The changing etiology of chronic diarrhea in HIV-infected patients with CD4 cell counts less than 200 cells/mm3. Am J Gastroenterol , 95(11):3142-3146.

Foudraine NA, Weverling GJ, van Gool T, Roos MT, de Wolf F, Koopmans PP, et al. (1998). Improvement of chronic diarrhoea in patients with advanced HIV-1 infection during potent antiretroviral therapy. Aids, 12(1):35-41.

Haagsma JA, Maertens de Noordhout C, Polinder S, Vos T, Havelaar AH, Cassini A, Devleesschauwer B, Kretzschmar ME, Speybroeck N, Salomon JA. Assessing disability weights based on the responses of 30,660 people from four European countries. Population Health Metrics 2015; 13: 10

Maggi P, Larocca AM, Quarto M, Serio G, Brandonisio O, Angarano G, et al. (2000) Effect of antiretroviral therapy on cryptosporidiosis and microsporidiosis in patients infected with human immunodeficiency virus type 1. Eur J Clin Microbiol Infect Dis, 19(3):213-217.

Mead PS, Slutsker L, Dietz V, McCaig LF, Bresee JS, Shapiro C, et al. (1999) Food-related illness and death in the United States. Emerg Infect Dis, 5(5):607-625.

Miao YM, Awad-El-Kariem FM, Franzen C, Ellis DS, Muller A, Counihan HM, et al. (2000) Eradication of cryptosporidia and microsporidia following successful antiretroviral therapy. J Acquir Immune Defic Syndr, 25(2):124-129.

Pozio E, Rezza G, Boschini A, Pezzotti P, Tamburrini A, Rossi P, et al. (1997) Clinical cryptosporidiosis and human immunodeficiency virus (HIV)- induced immunosuppression: findings from a longitudinal study of HIV-positive and HIV-negative former injection drug users. J Infect Dis, 176(4):969-975.

Vijgen SMC, Mangen MJM, Kortbeek LM, Van Duynhoven YTHP, Havelaar AH (2007). Disease burden and related costs of cryptosporidiosis and giardiasis in the Netherlands. 58 pp. Bilthoven.

Diphtheria

Thanks to vaccination, respiratory diphtheria has almost disappeared from many European countries. In total, 85% of patients suffer from subclinical disease or turn into asymptomatic carriers (Vitek, 1998) and only an estimated 15% of infections lead to a symptomatic case. The duration of acute illness was based on the Ontario Burden of Infectious Disease Study [AC1] ('the Ontario Study') [SW2] and set at 12 days (Kwong, 2012).

Risk of complications

Systemic toxicity (a toxic form of the disease with swelling of the neck) occurs in 8.1% of all diphtheria patients and may lead to complications such as myocarditis, neuropathies and renal failure (Rakhmanova, 1996). The more frequent complications of acute illness are myocarditis and polyneuropathies/nerve palsies. Other complications, such as sepsis, septic arthritis, pneumonia, otitis media, splenic and hepatic abscesses and rhinitis, were not included in the outcome tree because they are either extremely rare or mild.

Our model is based on the assumption that 8.1% of symptomatic patients would have a complicated form of the disease (Rakhmanova, 1996).

Permanent disability following myocarditis (arrhythmias)

Assuming that myocarditis represents 66.6% of the complicated diphtheria cases (Jayashree, 2006) and that 0.25% (Mandell, 1999) of these will develop permanent conduction defects (arrhythmias), the transition probability of patients with complications developing permanent cardiac disability is 0.17%.

Case fatality ratio

The US Centers for Disease Control and Prevention (US CDC) have reported a case-fatality proportion (CFP) of 5–10% for diphtheria, with higher death rates (up to 20%) among persons under five and over 40 years. The case fatality proportion has changed very little over the last 50 years (CDC, 2009).

In the model, the CFP associated with uncomplicated disease is 1% and with complicated disease 25.7% (Rakhmanova, 1996).

Model input summary

Table 1. Transition probabilities used in the outcome tree

Health outcome (Health state)	Distribution of health states in health outcome	Transition probability	Source/assumption
Symptomatic infection<br>(Uncomplicated)<br>(Complicated)	 91.9%<br>8.1%		Rakhmanova, 1996
Permanent disability (arrhythmias) following complicated symptomatic infection		0.17%	Jayashree, 2006; Mandell, 1999

Fatal cases following uncomplicated symptomatic infection		1%	Rakhmanova, 1996
Fatal cases following complicated symptomatic infection		25.7%	Rakhmanova, 1996

Table 2. Disability weights and duration

Health outcome (Health state)	Disability Weight (DW) (Haagsma, 2015)		Duration	
	DW	Label	In years	Source
Symptomatic infection (Uncomplicated) (Complicated)	0.051 (0.039-0.06) 0.125 (0.104-0.152)	Infectious disease, acute episode, moderate Infectious disease, acute episode, severe	0.003	Kwong 2012
Permanent disability (arrhythmias) following complicated symptomatic infection	0.295 (0.258-0.343)	Cardiac conduction disorders and cardiac dysrhythmias	Remaining life expectancy	

References

Centers for Disease Control and Prevention. Epidemiology and Prevention of Vaccine-Preventable Diseases. Atkinson W, Wolfe S, Hamborsky J, McIntyre L, eds. 11th ed. Washington DC: Public Health Foundation, 2009.

Demirci CS, Abuhammour W. Diphtheria. Available from: http://emedicine.medscape.com/article/963334-clinical#showall Accessed: 1 September 2011.

Hadfield TL, McEvoy P, Polotsky Y, Tzinserling VA, Yakovlev AA. The pathology of diphtheria. J Infect Dis. 2000;181(SUPPL. 1):S116-20

Haagsma JA, Maertens de Noordhout C, Polinder S, Vos T, Havelaar AH, Cassini A, Devleesschauwer B, Kretzschmar ME, Speybroeck N, Salomon JA. Assessing disability weights based on the responses of 30,660 people from four European countries. Population Health Metrics 2015; 13: 10

Jayashree M, Shruthi N, Singhi S. Predictors of outcome in patients with diphtheria receiving intensive care. Indian Pediatr. 2006;43(2):155-60.

Kwong JC, Ratnasingham S, Campitelli MA, Daneman N, Deeks SL, et al. (2012) The Impact of Infection on Population Health: Results of the Ontario Burden of Infectious Diseases Study. PLoS ONE 7(9): e44103. doi:10.1371/journal.pone.0044103

Lodha R, Dash NR, Kapil A, Kabra SK. Diphtheria in urban slums in north India. Lancet, 2000 Jan 15;355(9199):204. PMID: 10675127

Mandell GL, Bennet JE, Dolin R. Mandell, Douglas and Bennett's principles and practice of infectious disease 5th edition. Churchill Livingstone, 1999.

Merck Manual available at http://www.merckmanuals.com/professional/infectious_diseases/gram-positive_bacilli/diphtheria.html?qt=diphtheria&alt=sh#v1005972 (last update May 2013)

Rakhmanova AG, Nosikova E, Tanasijchuk T, Saikku J. Diphtheria Outbreak in St. Petersburg: Clinical Characteristics of 1,860 Adult Patients. Scand J Infect Dis 1996; 28: 37-40.

Solders G, Nennesmo I, Perssont A, Diphtheritic neuropathy, an analysis based on muscle and nerve biopsy and repeated neurophysiological and autonomic function tests. Journal of Neurology, Neurosurgery, and Psychiatry 1989;52:876-880

Vitek CR, Wharton M. Diphtheria in the former soviet union: Reemergence of a pandemic disease. Emerging Infectious Diseases. 1998;4(4):539-50.

WHO (2004). Global Burden of Disease 2004 Update: Disability weights for diseases and conditions. Geneva: WHO. Available online at: http://www.who.int/healthinfo/global_burden_disease/

GBD2004_DisabilityWeights.pdf

Giardiasis

Acute gastroenteritis associated with giardia in humans is in most cases self-limiting within a few weeks (Wolfe, 2000). Vijgen et al. (Vijgen, 2007) assumed for their disease burden estimates a mean duration of 10 days for gastroenteritis cases not requiring medical help or requiring a visit to the doctor. Severe hospitalised gastroenteritis cases were assumed to last for 30 days.

We assumed that the proportion of more severe cases requiring hospitalisation would be 0.265% (360 cases requiring hospitalisation out of an estimated 136 000 incident cases) (Vijgen, 2007). Moreover, the study presents an age-specific risk of hospitalisation which we applied to the 'severe' health state of the symptomatic infection outcome (see Table 3).

The Dutch Association of Parasitology is not aware of fatal cases of giardia (Vijgen, 2007). Additionally, studies by Adak et al. (Adak, 2002) and Levy et al. (Levy, 1998) have not reported fatal cases.

However, a small number of deaths associated with giardiasis were reported to TESSy: nine cases between 2009 and 2013, resulting in 0.014% of notified cases. The CFP is applied to all symptomatic cases and re-distributed according to the age-group observed deaths for giardiasis and cryptosporidiosis notified between 2009 and 2013 from all Member States, with the exception of Denmark, France, Greece, Italy, Liechtenstein, the Netherlands and Portugal, because they do not report (see Table 4). Data from Bulgaria and Poland were also excluded because they only report aggregate data. It is important to note that the CFP will increase in case multipliers adjusting for under-estimation are applied to the incidence inputted in the toolkit and this should be taken into account.

Risk of complications

Apart from Irritable Bowel Syndrome (IBS) as a possible sequela of giardia, no other sequelae could be identified. However, given the fact that few studies expressed a statistical link between IBS and giardia (1–2%) (Nygard, 2006; Hanevik, 2009; Haagsma, 2010), IBS was not included as a possible complication.

Model input summary

Table 1. Transition probabilities used in the outcome tree

Health outcome (Health state)	Distribution of health states in health outcome	Transition probability	Source/assumption
Symptomatic infection<br>(Uncomplicated)<br>(Complicated)	99.735%<br>0.265%<br>Age dep. (Table 3)		
Fatal cases following		0.014%	TESSy 2009-2013

symptomatic infection		Age dependent (Table 4)	

Table 2. Disability weights and duration

Health outcome (Health state)	Disability Weight (DW) (Haagsma, 2015)		Duration	
	DW	Label	In years	Source/assumption
Symptomatic infection				Vijgen, 2007
(Moderate)	0.149 (0.12–0.182)	Diarrhoea, moderate	0.027	
(Severe)	0.239 (0.202-0.285)	Diarrhoea, severe	0.082	

Table 3. Age distribution of severe cases

Age class	%
0–4	27
5–9	27
10–14	3
15–64	34
≥65	8

Table 4. Age-group redistribution of CFR (applied only to complicated cases)

Age groups	%
0	12.50
1–4	6.25
5–9	6.25
10–14	0.00
15–19	0.00
20–24	0.00
25–29	0.00
30–34	6.25
35–39	0.00
40–44	0.00
45–49	6.25
50–54	12.50
55–59	6.25
60–64	6.25
65–69	6.25
70–74	6.25
75–79	18.75
80–84	6.25
>85	0.00
All ages	100.00

References

Adak GK, Long SM, O'Brien SJ. Trends in indigenous foodborne disease and deaths, England and Wales: 1992 to 2000. Gut, 2002. 51(6):832-841.

Food Standards Agency. A Report of the Study of Infectious Intestinal Disease in England. Food Standards Agency London, 2000. HMSO.

Haagsma JA, Siersema PD, De Wit NJ, Havelaar AH. Disease burden of post-infectious irritable bowel syndrome in the Netherlands. Epidemiol Infect 2010. 138(11):1650-6.

Haagsma JA, Maertens de Noordhout C, Polinder S, Vos T, Havelaar AH, Cassini A, Devleesschauwer B, Kretzschmar ME, Speybroeck N, Salomon JA. Assessing disability weights based on the responses of 30,660 people from four European countries. Population Health Metrics 2015; 13: 10

Hanevik K, Dizdar V, Langeland N, Hausken T. Development of functional gastrointestinal disorders after Giardia lamblia infection. BMC Gastroenterol, 2009. 9:27.

Lengerich EJ, Addiss DG, Juranek DD. Severe giardiasis in the United States. Clin Infect Dis, 1994.18(5):760-763.

Levy DA, Bens MS, Craun GF, Calderon RL, Herwaldt BL. Surveillance for waterborne-disease outbreaks – United States, 1995–96. MMWR CDC Surveill Summ, 1998. 47(5):1-34.

Nygard K, Schimmer B, Sobstad O, Walde A, Tveit I, Langeland N. A large community outbreak of waterborne giardiasis-delayed detection in a non-endemic urban area. BMC Public Health, 2006. 6:141.

Vijgen SMC, Mangen MJM, Kortbeek LM, Van Duynhoven YTHP, Havelaar AH. Disease burden and related costs of cryptosporidiosis and giardiasis in the Netherlands. 58 pp. Bilthoven. 2007. National Institute of Public Health and the Environment.

Wolfe MS. Giardiasis. Clin Microbiol Rev, 1992. 5(1):93-100.

Gonorrhoea

Gonorrhoea is the second most commonly reported sexually transmitted disease (STD) in the United States of America (Skolnik & Neil, 2008). Neisseria gonorrhoeae is almost exclusively transmitted by sexual contact and perinatally (from mother to child during labour) (Handsfield & Sparling, 2005). The bacteria affect the mucous membranes of the urethra and the cervix. Less frequently, mucous membranes of the rectum, oropharynx and conjunctivae are also involved during infection. N. gonorrhoeae primarily infects columnar and cuboidal epithelium. Gonorrhoeal infections in women may lead to pelvic inflammatory disease (PID) and may be a cause of female infertility. Further complications resulting from infection with N. gonorrhoeae are epididymitis, ophthalmitis, ectopic pregnancy and disseminated gonococcal infection (DGI). Untreated infections mostly resolve spontaneously over time (several weeks or months) but can lead to serious sequelae associated with adverse effects on health. Even though the duration of disease is hard to estimate, mean duration is assumed to be several days for men and less than two weeks for women. The incubation period is short and re-infection is common (Handsfield & Sparling, 2005).

The true number of gonorrhoea cases is largely affected by under-estimation due to high percentages of asymptomatic cases and diagnosed cases not being reported to the surveillance system. It was estimated that the true number of new infections is twice as high as the reported number (CDC, 2002). Brunham and Embree reported that gonorrhoea is posing serious threats in Africa, Latin America, Asia and eastern Europe (Brunham & Embree, 1992). In 2008, WHO estimated that there were around 46.8 million cases of STDs in the European Region, with 3.4 million cases being due to N. gonorrhoeae (WHO, 2012).

About 40–80% of women are asymptomatically infected (De Maio & Zenilman, 1998; Nelson, 2007). For men symptomatic rates of up to 95- 99% were observed for genital infection (De Maio & Zenilman, 1998; Nelson, 2007; Stamm, 2005).

Health outcomes and health states associated with gonococcal infection

Infection with N. gonorrhoeae results in different clinical pictures in women, men and infants. In our study, we only considered disease models which reflect genital infection; pharyngeal and rectal infections are not considered to be the cause of significant short or long-term sequelae and therefore do not contribute to the burden of gonorrhoea.

Infections in men

An uncomplicated infection presents as an acute urethritis, infection in the pharynx or rectum are likely to be asymptomatic. In 2013, 36% of reported gonorrhoea cases were detected at these sites. In most cases (95–99%) the disease has a symptomatic course with typical signs of dysuria and urethral discharge (De Maio & Zenilman, 1998; Nelson, 2007; Stamm, 2005). In a few cases the infection remains asymptomatic and is neither recognised nor diagnosed (Sherrard, 1996). These infections pose a serious problem as they provide a pool of further transmissible infections. In most cases gonococcal urethritis resolves spontaneously over several weeks but may also trigger sequelae (Handsfield & Sparling, 2005).

The most common sequela of gonococcal infections in men is the acute epididymitis (Stamm, 2005; Trojian, 2009). The symptoms associated with epididymitis are oligospermia during the acute phase, swollen epididymis (and/or testicles), and dysuria. The association between epididymitis and future infertility is an ongoing debate in research with no clear evidence (Stamm, 2005). Uncommon complications are penile oedema, penile lymphangitis, periurethral abscess, acute prostatitis, seminal vasculitis and Tyson's or Cowper's gland infections (Handsfield & Sparling, 2005). Due to their rare occurrence they are not considered in the outcome tree.

Infections in women

Uncomplicated infections in women mostly affect the endocervix and N. gonorrhoeae are also recovered from the urethra, rectum or occasionally from the periurethral (Skene's) glands and the ducts of Bartholin's glands. Many women with gonococcal infections only develop minor symptoms or are entirely asymptomatic and thus do not seek medical advice and are consequently not reported to the surveillance system.

A major complication resulting in remarkable disease burden is pelvic inflammatory disease (PID) (Handsfield & Sparling, 2005; De Maio & Zenilman, 1998). Studies report 10–40% of infected women developing PID (Handsfield, 1974; McCormack, 1977; Westrom, 1980; Westrom, 1992). In a cost effectiveness analysis, Bernstein and colleagues estimated a base case scenario of 30% (range 10–40%) of infected women developing PID (Bernstein, 2006). Women with

PID have an increased risk of developing infertility in the future (Handsfield & Sparling, 2005; De Maio & Zenilman, 1998; Westrom, 1980; Westrom, 1992; Ross, 2002). The study of Weström (1992) and colleagues reported a 10% probability of infected women developing tubal infertility. The risk of infertility is linked to number and severity of PID episodes. Ross reported 15–20% and 50–80% of infected women developing tubal infertility after one and three or more PID episodes, respectively. PID itself is also a cause of further (long-term) sequelae such as chronic pelvic pain, ectopic pregnancy and perihepatitis. Pelvic pain occurs in 20% of cases and ectopic pregnancy in 9.1% of PID cases (Handsfield & Sparling, 2005; Westrom, 1980). Infections with N. gonorrhoeae during pregnancy can result in spontaneous abortion, premature labour, early rupture of fetal membranes and perinatal infant mortality (Handsfield & Sparling, 2005). The cost effectiveness study by Bernstein and colleagues estimated transition probabilities from PID to chronic pelvic pain, ectopic pregnancy and tubal factor infertility of 18% (range 15–30), 7.8% (range 7.8–9.1%), and 15% (range 9–18%), respectively (Bernstein, 2006).

Sequelae reported for both sexes

As a result of bacteraemic dissemination, disseminated gonococcal infection (DGI) can occur in 0.5–3% of people infected with N. gonorrhoeae. This may cause infective arthritis and also be the cause of endocarditis and meningitis in very rare cases (Holmes, 2007).

Gonococcal infections in infants

Infants born to infected mothers can suffer from gonococcal conjunctivitis (ophthalmia neonatorum). Gonococcal conjunctivitis affects 30–35% of children born to infected mothers and is a major problem in many developing countries causing blindness (De Maio & Zenilman, 1998; Nelson, 2007). Ophthalmia neonatorum can lead to corneal scars, resulting in low-vision or complete blindness. Effective treatment is available which has led to very low numbers of sequelae resulting from ophthalmia neonatorum in the developed world (Darling, 2010; Schaller & Klauss, 2001). Consequently, we did not consider corneal-scar-related ‘low-vision’ or ‘blindness’ in our model.

Infected infants may have a low birth weight; some studies relate low birth weight to gonococcal infections (15% from Gerbase, 2000), however the attribution of this condition to the infection is extremely difficult in a developed country setting. Therefore, we decided to discard this relationship.

Case fatality proportion

Fatal cases resulting from gonococcal infections are extremely rare and mainly result from endocarditis, meningitis and DGI. Estimating the mortality of PID is complicated due to the lack of standardised case definitions, inconsistent reporting practices and unclear aetiology (percentage of fatal cases attributable to gonococcal PID) (De Meaio & Zenilman, 1998).

Outcome tree parameters

Male outcome-tree

The proportion of infections in men who develop symptoms is set at 95–99% (De Maio & Zenilman, 1998; Trojian, 2009, Nelson, 2007). The probability of developing DGI (which is part of the initial symptomatic phase of the disease) is set at 0.5–3% (Holmes, 2007), whereas the probability of developing epididymitis is set to 3% (1–5%) (Bernstein, 2006). Debate is currently ongoing as to whether asymptomatic cases also develop epididymitis, however, due to lack of a proven association, this was not taken into account.

Female outcome-tree

Information on the proportion of symptomatic (20–60%) and asymptomatic (40–80%) gonococcal infections were taken from reviews, clinical text books and a study conducted by Weström (Handsfield & Sparling, 2005; De Maio & Zenilman, 1998; Nelson, 2007; Westrom, 1992). Information on PID as a major sequela were obtained from reviews, clinical text books and a cost effectiveness analysis which provided an estimate that 30% (10–40%) of women were symptomatically infected (Bernstein, 2006). The probabilities of developing an ectopic pregnancy (7.8-9.1%), chronic pelvic pain (18%, range 15–30%) or tubal infertility (15%, range 9–18%) were taken from Bernstein`s cost-effectiveness study (Bernstein, 2006). Case fatality proportions from ectopic pregnancies were estimated at 0.038% (Goldner, 1993). The probability of developing a tubo-ovarian abscess is set at 0.8% (Ness, 2002). However, diagnosis and treatment have significantly improved it was therefore decided not to include a case fatality event for tubo-ovarian abscess.

Congenital outcome-tree

The burden studies on STDs by Gerbase and colleagues and Nelson et al. report 30–35% of cases developing ophthalmia neonatorum (Nelson, 2007; Gerbase, 2000).

Assuming that in EU/EEA Member States all notified cases will have had symptoms, in our model all cases of symptomatic infant gonococcal infections manifest as ophthalmia neonatorum and will represent the only health state included in the model.

Model input summary

Table 1. Transition probabilities and distributions used in the outcome tree

Health outcome	Distribution of health states in health	Transition probability	Source/assumption

(health state)	outcome		
Men			
Symptomatic infection<br>(Urethritis)<br>- Uncomplicated<br>- Complicated	 <br><br>97–99.5%<br>0.5–3%	 95–99%	 De Maio & Zenilman, 1998; Nelson, 2007; Stamm, 2005<br>Holmes, 2007
Epididymitis from symptomatic		3% (1–5%)	Bernstein, 2006
Women			
Symptomatic infection<br>(Cervicitis)<br>- Uncomplicated<br>- Complicated	 <br><br>97–99.5%<br>0.5–3%	 20–60%	 Handsfield & Sparling, 2005; De Maio & Zenilman, 1998; Nelson, 2007;<br>Westrom, 1992;<br>Holmes, 2007
Pelvic Inflammatory Disease (PID) from symptomatic and asymptomatic		30% (10–40%)	Bernstein, 2006
Ectopic pregnancy		7.8–9.1%<br>Age dep.<br>See Table 4	Bernstein, 2006<br>Female reproductive age 15-49
Tubo-ovarian abscess		0.8%	Ness, 2002
Chronic pelvic pain syndrome		18% (15–30%)	Bernstein, 2006

Tubal infertility		15% (9–18%)<br>Age dep.<br>See Table 4	Bernstein, 2006<br>Female reproductive age 15-49
Fatal cases due to ectopic pregnancy		0.038%	Goldner, 1993
Congenital			
Symptomatic infection<br>(Ophthalmia neonatorum)		100%	

Table 2. Disability weights and duration

Health outcome<br>(health state)	Disability Weight (DW) (Haagsma, 2015)		Duration	
	DW	Label	In years	Source
Men				
Uncomplicated<br>Complicated	0.051 (0.039-0.06)<br>0.125 (0.104-0.152)	Infectious disease, acute episode, moderate<br>Infectious disease, acute episode, severe	0.02<br>0.02	Trojian, 2009<br>Trojian, 2009
Epididymitis	0.176 (0.143-0.208)	Epididymo-orchitis	0.08	Trojian, 2009
Women				
Uncomplicated<br>Complicated	0.051 (0.039-0.06)<br>0.125 (0.104-0.152)	Infectious disease, acute episode, moderate<br>Infectious disease, acute episode, severe	0.03<br>0.03	Murray, 1996<br>Murray, 1996
Pelvic Inflammatory Disease (PID)	0.123 (0.1-0.15)	Abdominopelvic problem, moderate	0.07	De Maio & Zenilman, 1998
Tubo-ovarian abscess	0.31 (0.262-0.355)	Abdominopelvic problem, severe	0.01	Goharkhay, 2007; Teisala, 1990
Chronic pelvic pain	0.123 (0.1-0.15)	Abdominopelvic problem, moderate	2.8	Sharma, 2011
Ectopic pregnancy	0.31 (0.262-0.355)	Abdominopelvic problem, severe	0.08	Murray, 1996
Tubal infertility	0.007 (0.005-0.01)	Infertility, secondary	See Table 3	Female reproductive age 15–49 years<br>See Table 4

Congenital				
Symptomatic infection (Ophthalmia neonatorum)	0.015 (0.011-0.019)	Conjunctivitis without corneal scar	0.038	American Academy of Pediatrics, 2012. Assuming 2 weeks of treatment

Table 3. Duration of tubal infertility

Age	Duration in years
15–19	32
20–24	27
25–29	22
30–34	17
35–39	12

40–44	7
45–49	2

Table 4. Age group risk (only reproductive age)

Age	%
0–14	0
15–49	100
≥50	0

References

Adelman D, Casale T, Corren J. Manual of Allergy and Immunology. Philadelphia, USA: Lippincott, Williams and Wilkins; 2002.

American Academy of Pediatrics. Chlamydia trachomatis. In: Pickering LK, editor. Red book: 2012. Report of the Committee on Infectious Diseases. Elk Grove Village, IL: American Academy of Pediatrics; 2012:276–81.

American Social Health Association. Sexually Transmitted Diseases in America: How Many Cases and at What Cost? In: Sexually Transmitted Disease in America: How Many Cases and at What Cost? (Ed.). City; 1998:1-27.

Barr J, Danielsson D. Septic gonococcal dermatitis. Br Med J 1971, 1:482-485.

Bernstein KT, Mehta SD, Rompalo AM, Erbelding EJ. Cost-effectiveness of screening strategies for gonorrhea among females in private sector care. Obstet Gynecol 2006, 107:813-821.

Brunham R, Embree J. Sexually transmitted diseases: Current and future dimensions of the problem of the Third World. In: Reproductive Tract Infections Global Impact on Priorities for Women's Reproductive Health. Edited by Germanie A, Holmes K, Piot P, Wasserheit N. New York: Plenum Press; 1992: 35-38

Centers for Disease Control and Prevention. Sexually Transmitted Disease Surveillance, 2002. Atlanta: US Department of Health and Human Services; 2003.

Darling EK, McDonald H. A Meta-analysis of the Efficacy of Ocular Prophylactic Agents Used for the Prevention of Gonococcal and Chlamydial Ophthalmia Neonatorum. Journal of Midwifery & Women's Health 2010, 55:319-327.

DeMaio J, Zenilman J. Gonococcal Infections. In: Bacterial Infections of Humans: Epidemiology and Control. Edited by Evans A, Brachman P. New York: Plenum Medical Book Company; 1998: 285-304

Gerbase A, Stein C, Levison J, Htun Y. Global burden of sexually transmitted diseases (excluding HIV) in the year 2000. In: Global burden of sexually transmitted diseases (excluding HIV) in the year 2000 (Ed.) City: World Health Organization; 2000.

Goharkhay N, Verma U, Maggiorotto F. Comparison of CT- or ultrasound-guided drainage with concomitant intravenous antibiotics vs. intravenous antibiotics alone in the management of tubo-ovarian abscesses. Ultrasound Obstet Gynecol 2007, 29:65-69.

Goldner TE, Lawson HW, Xia Z, Atrash HK. Surveillance for ectopic pregnancy—United States, 1970–1989. MMWR CDC Surveill Summ. 1993;42:73–85.

Haagsma JA, Maertens de Noordhout C, Polinder S, Vos T, Havelaar AH, Cassini A, Devleesschauwer B, Kretzschmar ME, Speybroeck N, Salomon JA. Assessing disability weights based on the responses of 30,660 people from four European countries. Population Health Metrics 2015; 13: 10

Handsfield HH, Lipman TO, Harnisch JP, Tronca E, Holmes KK. Asymptomatic gonorrhea in men. Diagnosis, natural course, prevalence and significance. N Engl J Med 1974, 290:117-123.

Handsfield HH, Sparling RF. Neisseria gonorrhoeae. In Principles and Practice of Infectious Diseases. Volume Volume 2; 6th Edition. Edited by Mandel GL, Bennet JE, Dolin R. Peensylvania: Elsevier Inc.; 2005: 2514-2529

Holmes KK et al. Sexually Transmitted Diseases. 4th Edition 2007. McGraw-Hill Companies: New York. ISBN: 978-0071417488

Marra F, Marra CA, Patrick DM. Cost effectiveness analysis of azithromycin and doxycycline for Chlamydia trachomatis infection in women: A Canadian perspective. Can J Infect Dis 1997, 8:202-208.

McCormack WM, Stumacher RJ, Johnson K, Donner A. Clinical spectrum of gonococcal infection in women. Lancet 1977, 1:1182-1185.

Murray CJL, Lopez AD, World Health Organization, World Bank, Harvard School of Public Health. Global health statistics: a compendium of incidence, prevalence and mortality estimates for over 200 conditions. Geneva: World Health Organization; 1996.

Murray CJL, Lopez AD. The Global Burden of Disease - A comprehensive assessment of mortality and disability from diseases, injuries, and risk factors in 1990 and projected to 2020. Cambridge: Harvard School of Public Health on behalf of the World Health Organization and the World Bank; 1996.

Nelson AL. Gonorrheal Infections. In: Sexually Transmitted Infections - Current Clinical Practice. Edited by Nelson AL, Woodward J, Wysocki S: Humana Press; 2007: 153-182

Ness RB, Soper DE, Holley RL, Peipert J, Randall H, Sweet RL, et al. Effectiveness of inpatient and outpatient treatment strategies for women with pelvic inflammatory disease: results from the Pelvic Inflammatory Disease Evaluation and Clinical Health (PEACH) Randomized Trial. Am J Obstet Gynecol 2002. 186: 929-937.

Newman LM, Moran JS, Workowski KA. Update on the management of gonorrhea in adults in the United States. Clin Infect Dis 2007, 44 Suppl 3:S84-101.

Ross JD. An update on pelvic inflammatory disease. Sex Transm Infect 2002, 78:18-19.

Rowley J, Berkely S (1998). Sexually Transmitted Diseases. In: Health Dimensions of Sex and Reproduction. The Global Burden of Disease and Injuries Series, Volume 3. Harvard University Press, Cambridge.

Schaller UC, Klauss V. Is Crede's prophylaxis for ophthalmia neonatorum still valid? Bull World Health Organ 2001, 79:262-263.

Sharma D, Dahiya K, Duhan N, Bansal R. Diagnostic laparoscopy in chronic pelvic pain. Arch Gynecol Obstet 2011, 283:295-297.

Sharp JT. Editorial: Gonococcal infections and arthritis. Arthritis Rheum 1974, 17:511-512.

Sherrard J, Barlow D. Gonorrhoea in men: clinical and diagnostic aspects. Genitourin Med. 1996;72:422–6.

Skolnik NS, Neil S. Sexually Transmitted Diseases. In: Essential Infectious Disease Topics in Primary Care - Current Clinical Practice. Edited by Albert RH: Humana Press; 2008: 105-166

Spencer SE, Bash MC. Extragenital manifestations of Neisseria gonorrhoeae. Curr Infect Dis Rep 2006, 8:132-138.

Stamm WE, Jones RB, Batteiger BE. Chlamydia trachomatis (Trachoma, prinatal infections, Lymphogranuloma venerum and other genital infections). In Principles and Practice of Infectious Diseases. Volume 2, 6th Edition. Edited by Mandel GL, Bennet JE, Dolin R. Pennsylvania: Elsevier Inc.; 2005: 2239-2255

Teisala K, Heinonen PK, Punnonen R. Transvaginal ultrasound in the diagnosis and treatment of tubo-ovarian abscess. Br J Obstet Gynaecol 1990, 97:178-180.

Trojian TH, Lishnak TS, Heiman D. Epididymitis and orchitis: an overview. Am Fam Physician 2009, 79:583-587.

Westrom L, Joesoef R, Reynolds G, Hagdu A, Thompson SE. Pelvic inflammatory disease and fertility. A cohort study of 1,844 women with laparoscopically verified disease and 657 control women with normal laparoscopic results. Sex Transm Dis 1992, 19:185-192.

Westrom L. Incidence, prevalence, and trends of acute pelvic inflammatory disease and its consequences in industrialized countries. Am J Obstet Gynecol 1980, 138:880-892.

Wise CM, Morris CR, Wasilauskas BL, Salzer WL. Gonococcal arthritis in an era of increasing penicillin resistance. Presentations and outcomes in 41 recent cases (1985-1991). Arch Intern Med 1994, 154:2690-2695.

World Health Organization. Global incidence and prevalence of selected curable sexually transmitted infections - 2008. Geneva: World Health Organization; 2012.

Hepatitis A

Hepatitis A virus (HAV) infections range from asymptomatic health state to fulminant hepatitis (Jeong & Lee, 2010). Hepatitis A symptomatic infections depend strongly on the age: approximately 30% of infected children develop symptoms (Jeong & Lee, 2010; Ciocca, 2000), whereas, according to literature, this is 70–80% for adults (Jeong & Lee, 2010; Ciocca, 2000; Cuthbert, 2001). The manifestation of HAV infection in young children generally includes mild flu-like, but anicteric symptoms (Gingrich, 1983), whereas in adults frequently reported symptoms are jaundice, dark urine, fatigue, loss of appetite, abdominal pain and light-coloured stool lasting for several weeks (Koff, 1992).

Not only severity, also duration is related to the age of the patient. Symptoms in young children last for one to two weeks (Gingrich, 1983). According to Koff, around 80% of adults are ill for up to eight weeks (Koff, 1992). Haagsma et al. assumed that symptomatic HAV cases not requiring medical help would have symptoms for 14 days, and symptomatic HAV cases requiring any kind of medical help would have symptoms for 30 days (Haagsma, 2009). Havelaar et al. assumed that hospitalised HAV cases would have symptoms for up to 0.3 years(Haavelar, 2012). According to the US Centers for Disease Control and Prevention, clinical illness usually does not last longer than two months, although 10–15% of persons have prolonged or relapsing signs of symptoms for up to six months (CDC, 2012).

The case fatality proportions are reported to be 0.1% (Mead, 1999), 1% of hospitalised HAV cases (Arteaga Rodriguez, 2010) and 0.3% (Bauch, 2007; Fiore, 2004).

Fatal cases occur mainly in elderly people (Bauch, 2007; Jacobs, 2004; Jacobs, 2000). In the following table we have summarised the rates of mortality attributable to HAV as used in various cost-effectiveness analyses (Bauch, 2007; Jacobs, 2004; Jacobs, 2000).

Table 1. Deaths among symptomatic patients per 10 000 stratified for age classes

Age classes (in years)		Sources	
	Bauch 2007	Jacobs 2004	Jacobs 2000
	30	-	
5-14	18	-	
15-19	18	-	18 (6-30)
20-29	18	18	18 (6-30)
30-39	21	21	21 (10-32)
40-49	59	36	36 (23-49)

50-59	59	81	81 (70-92)
60-69	272	149	149 (146-152)
70-79	272	283	283 (154-310)
>80	272	283	385 (356-414)

We chose to consider the overall case fatality proportion to be within the range 0.1–0.3% and assumed a different age-group distribution of this risk based on the age-group distribution of fatal cases reported to TESSy between 2009 and 2013 (see Table 4). This table is based on all TESSy notified cases from EU Member States except Bulgaria, Lithuania, Latvia and Poland, because they report only aggregate data, and Liechtenstein which does not report.

Risk of complications

Fulminant hepatitis is a rare complication of hepatitis (Jeong & Lee, 2010). According to Bauch et al. (Bauch, 2007), the probability of fulminant infection in hospitalised HAV cases is 0.011%. Jacobs et al. (Jacobs, 2004) assumed that the probability of liver transplantation would be 0.02% for symptomatic HAV cases in 25 to 29-year olds, increasing slightly with age to 0.08% for symptomatic HAV cases in 70-year olds. According to Jeong and Lee (Jeong & Lee, 2010), a liver transplantation may be necessary, however HAV-related fulminant hepatitis does resolve spontaneously on a more frequent basis than fulminant hepatitis of other aetiologies. Given the low incidence, and the resulting negligible burden, fulminant hepatitis was not considered as a separate health outcome in the current study.

In a current review (Jeong & Lee, 2010), rare atypical clinical manifestations and extra-hepatic manifestations are listed. Atypical clinical manifestations occasionally reported are: relapsing hepatitis, prolonged cholestasis, and complicated cases with acute kidney injury. Rarely reported extra-hepatic manifestations are autoimmune haemolytic anaemia, aplastic anaemia, pure red cell aplasia, pleural or pericardial effusion, acute reactive arthritis, acute pancreatitis, acalculous cholecystitis, mononeuritis, and Guillain-Barré syndrome. None of these manifestations were considered in a recent disease burden study (Havelaar, 2012), nor in cost-effectiveness studies evaluating HAV vaccination programmes (Bauch, 2007; Jacobs, 2000, 2004).

Model input summary

Table 2. Transition probabilities used in the outcome tree

Health outcome<br><br>(Health state)	Distribution of health states in health outcome	Transition probability	Source/assumption
Fatal cases		0.1–0.3%.<br><br>Age-dependent (Table 4)	Mead 1999, Bauch 2007, Fiore 2004

Table 3. Disability weights and duration

Health outcome (Health state)	Disability Weight (DW) (Haagsma, 2015)		Duration	
	DW	Label	In years	Source/assumption
Symptomatic infection	0.125 (0.104–0.152)	Infectious disease, acute episode, severe	0–9 years: 0.019–0.038 ≥ 10 years: 0.082 (0.038–0.5).<br><br>See Table 5.	CDC 2012; Haagsma 2009, age-dependent

Table 4. Age-group redistribution of case fatality proportion (0.1–0.3%)

Age groups	%
0	0.00
1-4	0.00
5-9	0.00
10-14	0.00
15-19	0.00
20-24	10.00
25-29	0.00
30-34	0.00
35-39	0.00

40-44	10.00
45-49	0.00
50-54	10.00
55-59	20.00
60-64	10.00
65-69	0.00
70-74	20.00
75-79	20.00
80-84	0.00
>85	0.00
All ages	100.00

Table 5. Duration of symptomatic disease by age group

Age	%
0-9	0.019–0.038
$\geq$ 10	0.082 (0.038–0.5)

References

Allen CJ, Ferson MJ. Notification of infectious diseases by general practitioners: a quantitative and qualitative study. Med J Aust 2000, 172:325- 328.

Arteaga-Rodriguez A, Carrasco-Garrido P, de Andres AL, de Miguel AG, Santos J, Jimenez-Garcia R. Changes in the epidemiology of hepatitis A in Spain (2005-2008): trends of acute hepatitis A hospitalizations, comorbidities, and costs associated with the hospitalization. Eur J Gastroenterol Hepatol 2010, 22:1284-1289.

Bauch CT, Anonychuk AM, Pham BZ, Gilca V, Duval B, Krahn MD. Cost-utility of universal hepatitis A vaccination in Canada. Vaccine 2007, 25:8536-8548.

Centers for Disease Control and Prevention. Epidemiology and Prevention of Vaccine-Preventable Diseases. Atkinson W, Wolfe S, Hamborsky J, eds. 12th ed. Washington DC: Public Health Foundation, 2012.

Ciocca M. Clinical course and consequences of hepatitis A infection. Vaccine 2000, 18 Suppl 1:S71-74.

Cuthbert JA: Hepatitis A: old and new. Clin Microbiol Rev 2001, 14:38-58.

Fiore AE. Hepatitis A transmitted by food. Clin Infect Dis 2004, 38:705-715.

Gingrich GA, Hadler SC, Elder HA, Ash KO. Serologic investigation of an outbreak of hepatitis A in a rural day-care center. Am J Public Health 1983, 73:1190-1193.

Haagsma JA, Janssen MF, Bonsel GJ. Comparing generic and disease specific health state valuations by a laymen panel. In EuroQol proceedings; Oslo, Norway. 2005

Haagsma JA, van der Zanden BP, Tariq L, van Pelt W, van Duynhoven YTHP, Havelaar AH. Disease burden and costs of selected foodborne pathogens in the Netherlands, 2006. vol. RIVM rapport 330331001. Bilthoven: National Institute of Public Health and Environment; 2009.

Haagsma JA, Maertens de Noordhout C, Polinder S, Vos T, Havelaar AH, Cassini A, Devleesschauwer B, Kretzschmar ME, Speybroeck N, Salomon JA. Assessing disability weights based on the responses of 30,660 people from four European countries. Population Health Metrics 2015; 13: 10

Havelaar AH, Haagsma J, Mangen MJJ, Kemmeren JM, Verhoef L, Vijgen SMC, et al. Disease burden of foodborne pathogens in the Netherlands, 2009. International Journal of Food Microbiology 2012 156:231-238.

Havelaar AH, Melse JM. Quantifying public health risks in the WHO Guidelines for Drinking-Water Quality. Bilthoven, the Netherlands: National Institute for Public Health and the Environment; 2003.

Jacobs RJ, Gibson GA, Meyerhoff AS: Cost-effectiveness of hepatitis A-B vaccine versus hepatitis B vaccine for healthcare and public safety workers in the western United States. Infect Control Hosp Epidemiol 2004, 25:563-569.

Jacobs RJ, Margolis HS, Coleman PJ. The cost-effectiveness of adolescent hepatitis A vaccination in states with the highest disease rates. Arch Pediatr Adolesc Med 2000, 154:763-770.

Jeong SH, Lee HS. Hepatitis A: clinical manifestations and management. Intervirology 2010, 53:15-19.

Koff RS. Clinical manifestations and diagnosis of hepatitis A virus infection. Vaccine 1992, 10 Suppl 1:S15-17.

Matin N, Grant A, Granerod J, Crowcroft N. Hepatitis A surveillance in England--how many cases are not reported and does it really matter? Epidemiol Infect 2006, 134:1299-1302.

Mead PS, Slutsker L, Dietz V, McCaig LF, Bresee JS, Shapiro C, et al. Food-related illness and death in the United States. Emerg Infect Dis 1999, 5:607-625.

Van de Laar MJW, Wijgergangs LM, Rijlaarsdam J, de Wit A. Vaccineren tegen hepatitis A: een verkenning van kosten. Infectieziektebulletin 2000, 11:262-266.

WHO. Global Burden of Disease 2004 Update: Disability weights for diseases and conditions. Geneva: WHO; 2004.

Hepatitis B

Hepatitis B is caused by the hepatitis B virus (HBV) which affects the liver and can cause both acute and chronic infections. Many patients present no symptoms during the initial infection.

The following estimates have been calculated for the proportion of infected individuals who develop symptoms:

- 30–50% of those adults infected develop acute icteric hepatitis (McMahon et al. 1985)

- Over 90 percent of perinatal HBV infections are asymptomatic, while the typical manifestations of acute hepatitis are noted in 5–15 percent of newly-infected young children (1–5 years of age) and in 33–50 percent of older children, adolescents, and adults (Shepard et al. 2006).

We therefore assumed that the range for the symptomatic proportion of new infections was age-dependent (see Table 1). The duration of acute illness has been estimated at six weeks (Kwong, 2012).

Chronicity rate

There is much evidence of age-related variation in the development rate for chronic HBV infection after acute infection. For example:

- The likelihood of developing chronic HBV infection is higher in individuals infected perinatally (90%) or during childhood (20–30%), when the immune system is thought to be immature, compared with immunocompetent subjects infected during adulthood (<1%)' (Fattovich, 2008)

- The overall chronicity rate for HBV has been estimated at 5–10%, although it is higher in those who were infected perinatally (90%) or during childhood (20%) (Yim & Lok, 2005)

- More than 90% of infected infants, 25–50% of children infected between and 5 years, and 6–10% of acutely infected older children and adults develop chronic infection (Shepard et al. 2006)

- About 30% of children aged 1–5 years and 5% of adults develop chronic hepatitis B infection (Pungpadong et al. 2007).

- Nearly all persons infected perinatally and up to 50% of children infected between the ages of 1–5 years develop chronic hepatitis (NIH, 2008)

- 5% of adults with acute infection develop chronic hepatitis B (Wilt et al. 2008)

- 5-10% of adult patients do not clear the virus and either progress to become asymptomatic carriers or develop chronic hepatitis (WHO 2002)

- The chronicity rate is approximately 90% for infants in the first year of life, 30% for children infected between the ages of 1 and 4 years and <5% for healthy adults (Edmunds et al. 1993).

In the model, we adopted the age-dependent chronicity rates reported above by Fattovich et al. presented in the results of a systematic review of the literature (2008).

The duration of the chronic carrier stage varies according to the presence or absence of active viral replication, estimated at 4.5 years in the case of active viral replication and 33.24 years in the case of no active replication (Stouthard, 1997). Information on the proportion of chronic hepatitis cases with active viral replication to those without active replication is not available and we chose to set the duration as uncertain, between 4.5 and 33.24 years.

Risk of complications

Fulminant liver failure

Fulminant liver failure occurs in approximately 0.5 to 1.0% of adults with reported acute hepatitis B but rarely in infants and children (Pappas, 1995; Hoofnagle et al. 1995). In the model we specified a range (0.5–1.0%) for this transition probability for all age groups as we were unable to locate specific values for infants and children. However, we modelled the age-specific probability of the case fatality rate based on the observed rates, hence a zero probability of children dying of acute hepatitis (see Table 5).

The case fatality rate (CFR) among patients who develop fulminant liver failure is approximately 20–33% (Bernua et al. 1986; Wai et al. 2005) and this figure was chosen for our model. There were no recent specific European studies stating the frequency and impact of orthotopic liver transplantation (OLT) (Steinmuller et al. 2002) and new antiviral medications (Eisenbach, 2006).

The duration of fulminant liver failure, estimated based on the time from onset of symptoms to encephalopathy, is one to 56 days (Trey and Davidson 1970).

Compensated cirrhosis (CC)

According to Chu (2000), on average, 2.1% of people with chronic HBV infection develop compensated cirrhosis annually. This does not take into account variations due to other effects such as alcohol consumption, diabetes and obesity (in the BCoDE toolkit the yearly rate refers to an Annual Transition Probability, ATP, as opposed to the Lifetime Transition Probability, LTP). However, it is important to consider that individuals who have a severe acute exacerbation complicated by subacute hepatic failure or who have recurrent episodes of acute exacerbations with bridging hepatic necrosis are more likely to develop cirrhosis (Chu, 2000)

Decompensated cirrhosis (DC)

According to a systematic review undertaken by D'Amico et al. (2006. The review undertaken by Fattovich et al. (2008) estimated an annual probability of 3–4% for Europe which we chose for our model.

The 20–57% case fatality rate for DC was estimated based on the review by D`Amico et al. (20% from the first of two DC stages, characterised by ascites with or without non-bleeding esophageal varices; 57% from the second of two DC stages, characterised by bleeding varices, with or without ascites).

The duration of DC is based on the average waiting time for liver transplants in EU countries which publish their data online (UK and Spain): between 124 and 142 days (NHS, 2014; Matesanz 2009).

Hepatocellular carcinoma (HCC)

The annual rate of developing HCC is 0.1% in asymptomatic HBsAg individuals, and between 0.3 and 1% in patients with chronic hepatitis B, but this rate increases to 2–10% in patients with compensated cirrhosis (Fattovich, 2008; Yim & Lok, 2005; Pungpadong, 2007; Chu, 2000; D'Amico, 2006). Chu and Liaw (2006) and Fattovich (2008) estimated the CC to HCC transition probability to range between 1.5 and 2.2%/year for Europe.

For the model, we adopted Fattovich`s (2008) estimate stemming from an extensive systematic literature review of 0.3% (0.12–0.41) per year to develop HCC from chronic hepatitis B infection and 2.2% (1.71–2.71) per year for the development of HCC from compensated cirrhosis.

In a European setting, Shepherd`s (2006) cost-effectiveness analysis set the annual case fatality rate for HCC to 56%, while Kanwal (2005) set it to 43.3% (20–60). We chose the latter range for our model as it includes Shepherd`s assumption.

Model input summary

Table 1. Transition probabilities and distributions used in the outcome tree

Health outcome<br>(Health state)	Distribution of health states within health outcome	Transition probability	Source/assumption
Symptomatic infection		10–50%<br>See Table 3	Age-dependent<br>McMahon, 1985; Shepard, 2006
Chronic hepatitis		1–90%<br>See Table 4	Age-dependent<br>Fattovich, 2008
Fulminant liver failure		0.5–1%	Pappas, 1995; Hoofnagle et al. 1995
Fatal cases due to liver failure		20-33.3%<br>See Table 5	Bernau et al. 1986 ; Wait et al. 2005<br>Assuming different age-specific probabilities based on observed mortality

Compensated cirrhosis		2.1%/year	Chu, 2000 (ATP)
Decompensated cirrhosis		3-4%/year	Fattovich, 2008 (ATP)
HCC, following<br>- Chronic hepatitis<br>- Compensated cirrhosis		0.3% (0.12–0.41)/year<br>2.2% (1.71–2.71)/year	Fattovich, 2008 (ATP<br>Fattovich, 2008 (ATP
CFR, following:<br>- DC<br>- HCC		20-57%/year<br>43.3% (20-60)/year	D'Amico, 2006 (ATP)<br>Kanwal, 2005 (ATP)

Table 2. Disability weights and duration

Health outcome (Health state)	Disability Weight (DW) (Haagsma, 2015)		Duration	
	DW	Source: ECDC European Disability Weight Project (2014)	In years	Source/assumption
Symptomatic infection	0.051 (0.039–0.06)	Infectious disease, acute episode, moderate	0.115	Kwong 2012
Fulminant liver failure	0.515 (0.459–0.572)	Terminal phase, with medication (for cancers, end-stage kidney/liver disease)	0.003–0.153	Trey, 1970
Chronic hepatitis	0.07 (0.057–0.088)	Generic, uncomplicated disease: worry and daily medication	4.5–33.24	Stouthard, 1997

				Assuming uncertainty between proportion with active replication and without
Compensated cirrhosis	0.07 (0.057–0.088)	Generic uncomplicated disease: worry and daily medication	6-10.4<br><br>See Table 6	Murray, 1996<br><br>Age and gender specific
Decompensated cirrhosis	0.163 (0.136–0.194)	Decompensated cirrhosis of the liver	0.34–039	Assuming average waiting time before liver transplantation in the UK and Spain (NHS and Matesanz 2009)
Hepatocellular carcinoma	0.265 (0.222–0.303)	Cancer, diagnosis and primary therapy	0.72–4.48<br><br>See Table 7	Murray, 1996<br><br>Age and gender specific

Table 3. Hepatitis B infected developing symptoms

Age group	Symptomatic hepatitis B
0	10%
1–4	5–15%
5–80+	30–50%

Table 4. Hepatitis B infected developing chronic hepatitis

Age group	Chronic hepatitis B
0	90%
1–4	20–30%
5–80+	1%

Table 5. CFR age distribution for acute hepatitis observed in Estonia, Germany and the Netherlands 2005–2007

Age groups	CFR
0	0.00

1-4	0.00
5-9	0.10
10-14	0.00
15-19	0.00
20-24	0.14
25-29	0.30
30-34	0.53
35-39	1.27
40-44	1.75
45-49	4.56
50-54	5.81
55-59	5.83
60-64	7.90
65-69	11.86
70-74	11.97
75-79	19.77
80-84	15.67
>85	12.54
All ages	100

Table 6. Duration of compensated cirrhosis

	F	M
0–4	10.4	10.3
5–14	10.4	10.4
15–44	10.2	10
45–59	9.3	8.8
60+	6.5	6

Table 7. Duration of HCC

Age group	Duration (years)	
	F	M
0–14	4.48	4.11
15–44	1.45	2.92
45–59	1.91	2.88
60+	0.72	1.56

References

Alter MJ, Mares A, Hadler SC, Maynard JE. The effect of underreporting on the apparent incidence and epidemiology of acute viral hepatitis. Am J Epidemiol. 1987;125(1):133-9.

Bernuau J, Goudeau A, Poynard T. Multivariate analysis of prognostic factors in fulminant hepatitis B. Hepatology, vol.6, no. 4, pp. 648–651, 1986

Bernuau J, Rueff B, Benhamou JP. Fulminant and subfulminant liver failure: definitions and causes. Seminars in Liver Disease, vol. 6, no. 2, pp. 97–106, 1986

Christensen PB, et al. Outbreak of Hepatitis B among injecting drug users in Denmark. J Clinical Virology 2001;22(1):133-141.

Chu CM. (2000), Natural history of chronic hepatitis B virus infection in adults with emphasis on the occurrence of cirrhosis and hepatocellular carcinoma. Journal of Gastroenterology and Hepatology, 15: E25–E30.

D'Amico G, Garcia-Tsao G, Pagliaro L. Natural history and prognostic indicators of survival in cirrhosis: a systematic review of 118 studies. Journal of Hepatology 2006;44(1):217-31.

Edmunds WJ, Medley GF, Nokes DJ, Hall AJ, Whittle HC. The influence of age on the development of the hepatitis B carrier state. Proceedings Biological Sciences, 1993;253(1337):197-201.

Elhouhari HM, Tamimi TIAR, Carey WD. Hepatitis B virus infection: Understanding its epidemiology, course, and diagnosis. Cleveland Clinical Journal of Medicine 2008;75:881-889.

Eisenbach C, Sauer P, Mehrabi A, Stremmel W, Encke J. Prevention of hepatitis B virus recurrence after liver transplantation. Clin Transplant 2006; 20 Suppl 17: 111–116

Fattovich G, Bortolotti F, Donato F. Natural history of chronic hepatitis B: special emphasis on disease progression and prognostic factors. [Review] [143 refs]. Journal of Hepatology. 2008 20071204 [Epub ahead of print];48(2):335-52.

Garfein RS, Vlahov D, Galai N, et al. Viral infections in short-term injection drug users: the prevalence of the hepatitis C, hepatitis B, human immunodeficiency, and human T-lymphotropic viruses. Am J Public Health 1996;86:655–661.

Greten TF, Papendorf F, Bleck JS, Kirchhoff T, Wohlberedt T, Kubicka S, et al. Survival rate in patients with hepatocellular carcinoma: a retrospective analysis of 389 patients. British Journal of Cancer, 2005 May 23;92(10):1862-8.

Haagsma JA, Maertens de Noordhout C, Polinder S, Vos T, Havelaar AH, Cassini A, Devleesschauwer B, Kretzschmar ME, Speybroeck N, Salomon JA. Assessing disability weights based on the responses of 30,660 people from four European countries. Population Health Metrics 2015; 13: 10

Hoofnagle JH, Carithers RL Jr, Shapiro C, Ascher N. Fulminant hepatic failure: summary of a workshop. Hepatology 1995;21:240-252.

Hoofnagle JH, Doo E, Liang TJ, Fleischer R, Lok ASF. Management of Hepatitis B: Summary of a Clinical Research Workshop. Hepatology 2007;45:1056-1075.

Kanwal F, Gralnek IM, Martin P et al. Treatment alternatives for chronic hepatitis B viral infection: a cost-effectiveness analysis. Annals of Internal Medicine. 2005;142:821-31.

Kim WR, Brown RS, Terrault NA, El-Serag H. Burden of liver disease in the United States: summary of a workshop. Hepatology 2002; 36: 227-242

Kwong JC, Ratnasingham S, Campitelli MA, Daneman N, Deeks SL, et al. (2012) The Impact of Infection on Population Health: Results of the Ontario Burden of Infectious Diseases Study. PLoS ONE 7(9): e44103. doi:10.1371/journal.pone.0044103

Levine OS, Vlahov D, Nelson KE. Epidemiology of hepatitis B virus infections among injecting drug users: seroprevalence, risk factors, and viral interactions. Epidemiol Rev 1994;16:418–36

Levine OS, Vlahov D, Koehler J, et al. Seroepidemiology of hepatitis B virus in a population of injecting drug users. Association with drug injection patterns. Am J Epidemiol 1995;142:331–41.

Liaw YF (2009) Natural history of chronic hepatitis B virus infection and long-term outcome under treatment. Liver international Vol. 29; pp 100- 107.

Lok ASF, McMahon (2007) Chronic Hepatitis B. Hepatology Vol. 45; pp 507 - 539

Matesanzemail R, de la Rosa G. Liver transplantation: The Spanish experience. Digestive and Liver Disease Supplements. Volume 3, Issue 4 , Pages 75-81, December 2009.

McMahon BJ, Alward WL, Hall DB, Heyward WL, Bender TR, Francis DP, et al. Acute hepatitis B virus infection: relation of age to the clinical expression of disease and subsequent development of the carrier state. The Journal of Infectious Diseases, 1985 Apr;151(4):599-603.

McMahon BJ (2005). Epidemiology and natural history of Hepatitis B. Seminars in liver disease. Vol. 25. Supplement 1.

Murray CJL, Lopez AD. World Health Organization, World Bank, Harvard School of Public Health. Global health statistics: a compendium of incidence, prevalence and mortality estimates for over 200 conditions. Geneva: World Health Organization; 1996.

Murray CJL, Lopez AD. The Global Burden of Disease - A comprehensive assessment of mortality and disability from diseases, injuries, and risk factors in 1990 and projected to 2020. Cambridge: Harvard School of Public Health on behalf of the World Health Organization and the World Bank; 1996.

National Health System (NHS), UK. h p://www.nhs.uk/condi‸ons/Liver-transplant/Pages/Introduc‸on.aspx. Website visited on 31 July 2014

National Institutes of Health (2008) NIH Consensus Development Conference Statement on Management of Hepatitis B. NIH Consensus and State-of-the-Science Statements Volume 25, Number 2. October 20–22, 2008

Pungpapong S, Kim WR, Poterucha JJ. Natural history of hepatitis B virus infection: an update for clinicians. Mayo Clin Proc 2007; 82:967–975.

Shepard CW, Simard EP, Finelli L, Fiore AE, Bell BP (2006) Hepatitis B virus infection: Epidemiology and vaccination. Epidemiological Reviews; Vol. 28; pp 112-125

Shepherd J, Jones J, Takeda A et al. Adefovir dipivoxil and pegylated interferon alfa-2a for the treatment of chronic hepatitis B: a systematic review and economic evaluation. [Review] [136 refs]. Health Technology Assessment (Winchester, England). 2006;10(28):iii-iiv.

Stouthard M , Essink-Bot M, Bonsel G, Barendregt J, Kramers P. 1997. Disability weights for diseases in the Netherlands. Rotterdam, Department of Public Health, Erasmus University.

Trey C, Davidson CS. The management of fulminant hepatic failure. In: Popper H, Schaffner F, eds. Progress in liver disease. Volume 3. New York: Grune & Stratton, 1970:282-98

Wai CT, Fontana RJ, Polson J, Hussain M, Shakil AO, Han SH, et al. Clinical outcome and virological characteristics of hepatitis B-related acute liver failure in the United States. J Viral Hepat 2005; 12: 192-198

Wilt TJ, Shamliyan T, Shaukat A, Taylor BC, MacDonald R, Yuan J-M, et al. (2008) . Management of Chronic Hepatitis B. Evidence Report/Technology Assessment No. 174. (Prepared by the Minnesota Evidence-based Practice Center under Contract No. 290-02-0009.) AHRQ Publication No. 09-E002. Rockville, MD. Agency for Healthcare Research and Quality. October 2008.

WHO (2002) Hepatitis B; WHO/CDS/CSR/LYO/2002.2:Hepatitis B

WHO (2001) Introduction of Hepatitis B vaccine into childhood immunization services

Yim HJ, Lok ASF. Natural history of chronic hepatitis B virus infection: what we knew in 1981 and what we know in 2005. Hepatology 2006; 43:pp 173–S181.

Hepatitis C

A total of 20–30% of newly infected individuals develop clinical symptoms of acute hepatitis (e.g. anorexia, abdominal discomfort, nausea, vomiting and jaundice) within 2–24 weeks of exposure (CDC, 2011; Wasmuth, 2010; World Health Organization, 2002). In persons who do develop symptoms of acute hepatitis, the illness lasts between two and 12 weeks (Wasmuth, 2010).

In the model, it was assumed that 20–30% of newly infected individuals develop clinical symptoms of acute hepatitis (CDC, 2011).

Rate of developing chronic hepatitis C

Acute hepatitis C develops into chronic infection in 75.6% (67.3–84.9) of all symptomatic and asymptomatic cases over 20 years old, with the infection resolving in the remaining proportion (Alter & Seef, 1994). The chronicity rate is known to be lower in younger individuals. A recent review of the literature by Alter et al. (2000), has estimated that the rate of spontaneous recovery is 29–45% in those aged under 20 years and this was used for the disease model (chronicity rate: 55–69%).

In the early stages of chronic infection there is a small chance of spontaneous remission. The rate of remission of chronic hepatitis C was set at 0.31 (0.26–0.36)% per year in accordance with the findings of Micallef et al. (2006) (in the Burden of Communicable Diseases in Europe toolkit a yearly rate refers to an Annual Transition Probability, ATP, as opposed to the Lifetime Transition Probability, LTP).

In the absence of spontaneous remission or successful antiviral therapy, chronic infections may progress from mild to moderate hepatitis to liver cirrhosis, with a risk of developing life-threatening sequelae such as decompensated liver disease and hepatocellular carcinoma. Progression to severe liver disease can take 20–40 years. However, progression, which is non-linear, is strongly influenced by cofactors including alcohol intake, HIV or HBV coinfection, gender (male) and an older age at infection (Alberti, 2005; Alter & Seeff, 2000; Freeman, 2003; Lauer & Walker, 2001; Poynard, 2001; Thein, 2008).

Given emerging knowledge of the disease, the most appropriate approach to simulating the progression from chronic infection to cirrhosis would be to specify a model with five health stages, representing the METAVIR fibrosis stages F0–F4, linked by multivariate risk functions. A further possibility could be to represent mild and moderate pre-cirrhotic disease stages. However, for the sake of simplicity and in the context of a burden of disease study in which the objective is to compare a broad spectrum of diseases, a single, chronic hepatitis health outcome was applied.

Risk of complications

Compensated cirrhosis (CC)

The risk of HCV-infected persons developing cirrhosis within 20–30 years is estimated in most studies to be within the range of between 5 and 20%, although some studies give estimates of up to 50% (CDC, 2011; Freeman 2001; Freeman 2003; Lauer & Walker, 2001; Poynard, 1997; Poynard, 2001; Thein, 2008; Wasmuth, 2010). Thein et al. predicted via meta-analysis an average 20-year cirrhosis risk of 16% (95% CI: 14%-19%), and a 30-year risk of 41% (95% CI: 36%–45%), which underlines that the progression to cirrhosis is not a linear process (Thein et al. 2008).

The annual risk of progressing to compensated cirrhosis was calculated based on the transitional probabilities between the five METAVIR stages of fibrosis, as estimated by Thein et al. (2008), using random-effect meta-analysis applied to non-clinical studies only. The point estimate for the risk of developing compensated cirrhosis from chronic hepatitis, calculated as the inverse of the summed durations in the first four METAVIR stages (each duration in turn was estimated as 1/probability of leaving the METAVIR stage), was 1.9% per year. The disability duration was calculated at 36.5 years; this is the average time taken for 50% of those with chronic hepatitis to exit the compartment: $1- \exp(-0.019 * 36.5) = 0.5$.

Decompensated cirrhosis (DC)

HCV-associated cirrhosis leads to liver failure and death in about 20–25% of cirrhotic cases. The annual risk of compensated cirrhosis progressing to the decompensated stage (characterised by ascites, bleeding oesophagal varices, or jaundice) is estimated to be 3.9–7% (D'Amico, 2006; Fattovich, 1997; Grieve, 2006; Poynard, 1997; Wasmuth, 2010). In the model, hepatic decompensation was assumed to occur with an annual risk of 3.9 to 12.9 (Dienstag, 2011).

Without transplantation the prognosis is poor. The five-year survival rate with decompensated liver cirrhosis is roughly 50% (Planas, 2004). One report based on a small study population (n=65) estimated the annual mortality rate at 12.9% (Fattovich et al. 1997), but higher values were reported in the systematic review by D'Amico et al. (2006) (20% 1-year mortality from the first stage of DC; 55% from the second DC stage, which is indicated by bleeding varices with or without ascites). The estimated annual risk of death from DC was set to within a range of 13– 38.5% (Fattovich, 1997; Grieve, 2006; D'Amico, 2006); the upper bound was calculated as the mean of the rates for the two DC stages reported by D'Amico et al (2006).

Duration of DC is based on average waiting time for liver transplant in the UK and in Spain which are represented as an average duration (142 days, NHS and 124 days, Matesanz 2009).

Hepatocellular carcinoma (HCC)

In contrast to hepatitis B, development of primary liver cancer, or hepatocellular carcinoma (HCC), is rare in patients with chronic hepatitis C who do not have cirrhosis (Lauer & Walker, 2001; Spengler, 2010; Wasmuth, 2010; WHO, 2002). Once cirrhosis is established, the risk of hepatocellular carcinoma is estimated to be 1–4% per year (Fattovich et al. 1997; Lauer & Walker, 2001). Studies modelling the natural course of hepatitis C have assumed annual risks of around 1.5% (Grieve et al. 2006; Siebert et al. 2003).

HCC is an outcome that can occur after either the compensated or decompensated cirrhosis stages. The annual risk of developing HCC following either CC or DC was set to 3%, based on the estimate by D'Amico et al. (2006).

Studies modelling the natural course of hepatitis C have assumed annual case fatality rates (CFR) due to liver cancer ranging widely from 43– 86% (Grieve, 2006; Siebert, 2003; Wong, 2000). This variation might be a consequence of stage and treatment-specific survival rates, and other underlying conditions including alcohol consumption, diabetes or obesity, where the higher estimate is used to simulate a situation without early diagnosis and effective treatment. In the model, this CFR is set to 48.9%/year, based on the 1-year survival rate (Kwong, 2012).

Other complications

Fulminant hepatic failure due to acute HCV infection is considered to be very rare (CDC, 2011; Lauer & Walker, 2001; Wasmuth, 2010; World Health Organization, 2002) except in cases of HBV coinfection (Chu, 1999). Fulminant liver failure and death was reported to occur in approximately 0.1% (2/1536) of adults with reported (notified) acute hepatitis C (Bianco, 2003). Due to this condition being extremely rare, no health outcome was specified in the outcome tree.

Model input summary

Table 1. Transition probabilities and distributions used in the outcome tree

Health outcome<br>(Health state)	Distribution of health states in health outcome	Transition probability	Source/assumption
Symptomatic infection	20–30%		CDC, 2011
Chronic hepatitis		> 19yr: 75.6% (67.3–84.9)<br>< 20yr: 55–69%	Alter, 1994; Alter, 2000<br>Age dependent
Remission from chronic hepatitis		0.31 (0.26–0.36)%/year	Micallef, 2006 (ATP)
Compensated cirrhosis		1.9%/year	Modelled from Thein, 2008 (ATP)
Decompensated cirrhosis		3.9–12.9%/year	Dienstag, 2011 (ATP)
HCC, following<br>- Compensated cirrhosis<br>- Decompensated cirrhosis		3.0%/year<br>3.0%/year	D'Amico, 2006 (ATP)<br>D'Amico, 2006 (ATP)
CFR, following:<br>- Decompensated cirrhosis<br>- Hepatocellular carcinoma		13–38.5%/year<br>48.9%/year	Fattovich, 1997; Grieve 2006;<br>D’Amico 2006 (ATP)<br>Kwong, 2012 (ATP)

Table 2. Disability weights and duration

Health outcome (Health state)	Disability Weight (DW) (Haagsma, 2015)		Duration	
	DW	Label	In years	Source
Symptomatic infection	0.051 (0.039–0.06)	Infectious disease, acute episode, moderate	0.038–0.23	CDC, 2011; Wasmuth, 2010; World Health Organization, 2002
Chronic hepatitis	0.07 (0.057–0.088)	Generic uncomplicated disease: worry and daily medication	36.5	Modelled from Thein, 2008
Compensated cirrhosis	0.07 (0.057–0.088)	Generic uncomplicated disease: worry and daily medication	6-10.4<br>See table 3	Murray, 1996<br>Age and gender specific
Decompensated cirrhosis	0.163 (0.136–0.194)	Decompensated cirrhosis of the liver	0.34–039	Assuming average waiting time before liver transplantation in UK and Spain (NHS and Matesanz 2009)
Hepatocellular carcinoma	0.265 (0.222–0.303)	Cancer, diagnosis and primary therapy	0.72–4.48<br>See table 4	Murray, 1996<br>Age and gender specific

Table 3. Duration of compensated cirrhosis

Age group	Duration (years)	
	F	M

0-4	10.4	10.3
5-14	10.4	10.4
15-44	10.2	10
45-59	9.3	8.8
60+	6.5	6

Table 4. Duration of hepatocellular carcinoma

Age group	Duration (years)	
	F	M
0-14	4.48	4.11
15-44	1.45	2.92
45-59	1.91	2.88
60+	0.72	1.56

References

Alberti A, Vario A, Ferrari A, Pistis R. (2005). Review article: chronic hepatitis C--natural history and cofactors. Aliment Pharmacol Ther 22 Suppl 2, 74-78.

Alter MJ, Kruszon-Moran D, Nainan OV, McQuillan GM, Gao F, Moyer LA, et al. The prevalence of hepatitis C virus infection in the United States, 1988 through 1994. Engl J Med. 1999 Aug 19; 341(8):556-62.

Alter HJ, Seeff LB. (2000). Recovery, persistence, and sequelae in hepatitis C virus infection: a perspective on long-term outcome. Semin Liver Dis 20, 17-35.

Bianco E, Stroffolini T, Spada E, Szklo A, Marzolini F, Ragni P, et al. (2003). Case fatality rate of acute viral hepatitis in Italy: 1995-2000. An update. Dig Liver Dis 35, 404-408.

Centers for Disease Control and Prevention (CDC). Hepatitis C: Information for Health Professionals. 2011. Available at: http://www.cdc.gov/hepatitis/HCV/index.htm.

Chu CM, Yeh CT, Liaw YF. Fulminant hepatic failure in acute hepatitis C: increased risk in chronic carriers of hepatitis B virus. Gut 1999;45:613- 617.

Crofts N, Jolley D, Kaldor J, et al. Epidemiology of hepatitis C virus infection among injecting drug users in Australia. J Epidemiol Community Health 1997;1:692–697.

D'Amico G, Garcia-Tsao G, Pagliaro L. Natural history and prognostic indicators of survival in cirrhosis: a systematic review of 118 studies. J Hepatol. 2006;44(1):217-31.

Dienstag JL, Ghany MG, Morgan TR, Di Bisceglie AM, Bonkovsky HL, Kim HY, et al. and the HALT-C Trial Group: A prospective study of the rate of progression in compensated, histologically advanced chronic hepatitis C. Hepatology 2011, 54:396–405

ECDC. Surveillance and prevention of hepatitis B and C in Europe. Stockholm: ECDC 2010.

Fattovich G, Giustina G, Degos F, Tremolada F, Diodati G, Almasio P, et al. (1997). Morbidity and mortality in compensated cirrhosis type C: a retrospective follow-up study of 384 patients. Gastroenterology 112, 463-472.

Freeman AJ, Dore GJ, Law MG, Thorpe M, Von Overbeck J, Lloyd AR et al. Estimating progression to cirrhosis in chronic hepatitis C virus infection. Hepatology 2001;34:809-816.

Freeman AJ, Law MG, Kaldor JM, and Dore GJ. Predicting progression to cirrhosis in chronic hepatitis C virus infection. J Viral Hepat 2003;10:285-293.

Grieve R, Roberts J, Wright M, Sweeting M, DeAngelis D, Rosenberg W, et al. Cost effectiveness of interferon alpha or peginterferon alpha with ribavirin for histologically mild chronic hepatitis C. Gut 2006;55:1332-1338.

Hagan H, Snyder N, Hough E, Yu T, McKeirnan S, Boase J, et al. Case-reporting of acute hepatitis B and C among injection drug users. J Urban Health 2002;79(4):579-85.

Haagsma JA, Maertens de Noordhout C, Polinder S, Vos T, Havelaar AH, Cassini A, Devleesschauwer B, Kretzschmar ME, Speybroeck N, Salomon JA. Assessing disability weights based on the responses of 30,660 people from four European countries. Population Health Metrics 2015; 13: 10

Kwong JC, Ratnasingham S, Campitelli MA, Daneman N, Deeks SL, et al. (2012) The Impact of Infection on Population Health: Results of the Ontario Burden of Infectious Diseases Study. PLoS ONE 7(9): e44103. doi:10.1371/journal.pone.0044103

Lauer GM, Walker BD. Hepatitis C virus infection. N Engl J Med. 2001;345:41-52.

Mann AG, Ramsay ME, Brant LJ, Balogun MA, Costella A, Harris HE. Diagnoses of, and deaths from, severe liver disease due to hepatitis C in England between 2000 and 2005 estimated using multiple data sources. Epidemiol Infect. 2009;137(4):513-8.

Matesanzemail R, de la Rosa G. Liver transplantation: The Spanish experience. Digestive and Liver Disease Supplements. Volume 3, Issue 4 , Pages 75-81, December 2009.

McDonald SA, Hutchinson SJ, Mills PR, et al. Diagnosis of hepatitis C virus infection in Scotland's injecting drug user population. Epidemiol. Infect 2010;138:393–402.

Micallef JM, Kaldor JM, Dore GJ (2006) Spontaneous viral clearance following acute hepatitis C infection: a systematic review of longitudinal studies. J Viral Hepat 13: 34–41.

Murray CJL, Lopez AD, World Health Organization., World Bank., Harvard School of Public Health.: Global health statistics: a compendium of incidence, prevalence and mortality estimates for over 200 conditions. Geneva: World Health Organization; 1996.

Murray CJL, Lopez AD: The Global Burden of Disease - A comprehensive assessment of mortality and disability from diseases, injuries, and risk factors in 1990 and projected to 2020. Cambridge: Harvard School of Public Health on behalf of the World Health Organization and the World Bank; 1996.

National Health System (NHS), UK. Available at: http://www.nhs.uk/conditions/Liver-transplant/Pages/Introduction.aspx(accessed 31 July 2014)

Okuda K, Ohtsuki T, Obata H, Tomimatsu M, Okazaki N, Hasegawa H. et al. (1985). Natural history of hepatocellular carcinoma and prognosis in relation to treatment. Study of 850 patients. Cancer 1985;56:918-928.

Planas R, Balleste B, Alvarez MA, Rivera M, Montoliu S, Galeras JA, et al. Natural history of decompensated hepatitis C virus-related cirrhosis. A study of 200 patients. J Hepatol. 2004;40823-830.

Poynard T, Bedossa P, Opolon P. Natural history of liver fibrosis progression in patients with chronic hepatitis C. The OBSVIRC, METAVIR, CLINIVIR, and DOSVIRC groups. Lancet 1997;349:825-832.

Poynard T, Ratziu V, Charlotte F, Goodman Z, McHutchison J, Albrecht J. Rates and risk factors of liver fibrosis progression in patients with chronic hepatitis c. J Hepatol. 2001;34:730-739.

Siebert U, Sroczynski G, Rossol S, Wasem J, Ravens-Sieberer U, Kurth BM, et al. (2003). Cost effectiveness of peginterferon alpha-2b plus ribavirin versus interferon alpha-2b plus ribavirin for initial treatment of chronic hepatitis C. Gut 52, 425-432.

Spengler U. (2010). Hepatocellular Carcinoma - Diagnosis, Prognosis & Therapy. In Hepatology - A Clinical Textbook. S. Mauss, T. Berg, J. Rockstroh, C. Sarrazin, and H. Wedemeyer, eds. (Düsseldorf, Flying Publisher).

Strauss R, Fülöp G, Pfeifer C. Hepatitis C in Austria 1993–2000: reporting bias distort HCV epidemiology in Austria. Euro Surveill. 2003;8(5):pii=412.

Thein HH, Yi Q, Dore GJ, Krahn MD. (2008). Estimation of stage-specific fibrosis progression rates in chronic hepatitis C virus infection: a meta- analysis and meta-regression. Hepatology 48, 418-431.

Wasmuth, J. (2010). Hepatitis C - Epidemiology, transmission and natural history. In Hepatology - A Clinical Textbook. S. Mauss, T. Berg, J. Rockstroh, C. Sarrazin, and H. Wedemeyer, eds. (Düsseldorf, Flying Publisher).

Wong JB, McQuillan GM, McHutchison JG, Poynard T. (2000). Estimating future hepatitis C morbidity, mortality, and costs in the United States. Am J Public Health 90, 1562-1569.

World Health Organization (2002). Hepatitis C. Available at: http://www.who.int/csr/disease/hepatitis/whocdscsrlyo2003/en/index.html. Accessed: February 2011.

Yousuf M, Nakano Y, Tanaka E, Sodeyama T, Kiyosawa K. Persistence of viremia in patients with type-C chronic hepatitis during long-term follow-up. Scand J Gastroenterol. 1992;27:812-816.

HIV

Acquired Immunodeficiency Syndrome (AIDS) is the most severe outcome of an untreated HIV infection. AIDS presents with severe opportunistic infections, malignancies, neurological complications or other HIV-induced disease conditions (Del Rio & Curran, 2005). After infection with HIV, individuals may remain asymptomatic or develop Acute Retroviral Syndrome (ARS) (Del Rio & Curran, 2005). ARS occurs in 50–66% of all recently infected cases (Sterling & Chaisson, 2005). Due to mild and non-specific flu-like symptoms many people do not seek medical advice, and thus are not diagnosed and treated and proceed to a latent stage where they may remain asymptomatic for years before subsequently developing AIDS.

Within the EU, it is estimated that around 8–45% of all HIV infections are undiagnosed and therefore not reported to the health authorities (ECDC, 2014). The overall duration is difficult to estimate because since introduction of Anti-Retroviral Therapy (ART) HIV is increasingly observed as being a chronic disease and individuals receiving treatment have a similar life expectancy to the rest of the population in Europe (Bhaskaran, 2008). Persistent asymptomatic HIV infection is estimated to be on average 17.2 years for long-term non-progressors (Herida, 2006).

Health outcomes/states associated with HIV-infection

HIV is associated with a heterogeneous set of health outcomes/states. In most cases, certain health outcomes/states are caused by subsequent infections with a secondary or tertiary pathogen. HIV compromises the immune status of an individual and thus increases the risk of further additional pathogens causing severe sequelae.

For our study, we considered that in Europe development of AIDS is significantly limited through ART.

HIV/AIDS is a complicated, multi-faceted and systemic disease and for reasons of feasibility, we developed a simplified model which does not differentiate between the CD4 count stages of the disease at the point of diagnosis, even though this is known to affect mortality (Aghaizu, 2013). Moreover, the current model does not take into account transmitted drug resistance, or the issue of co-morbidity (HIV–HCV or HIV–TB) and the consequent need for a specific therapeutic pathway. We assumed that all diagnosed cases are offered treatment and we applied a certain burden to the disease (e.g. side effects).

HIV infection-related deaths are associated with the development of an acquired immunodeficiency syndrome (AIDS) which, after a prolonged latent period, eventually enables opportunistic infections to develop which are generally the cause of death. Therefore, the nature of AIDS itself consists of comorbidities introducing the issue of attributable cause of death. However, we assumed that the severity of the co-infection and the precipitation to death would not have occurred without the primary HIV infection and deaths were therefore attributed entirely to the initial HIV infection. We also did not include the burden associated with HIV-related malignancies or complications linked to long-term antiretroviral therapy (e.g. cardiovascular disease).

Outcome-tree parameters

The main input is 'persistent HIV infection' and this is subdivided according to the speed of progression (Qu, 2008). In general, 5–15% of all patients are rapid progressors (RP) and are at risk of developing AIDS within 2–5 years (Qu, 2008). Another 5–15% are long-term non- progressors (LNP) with, on average 17.2 years duration of development (Qu, 2008; Sterling & Chaisson, 2005). The remainder (70–90%) are typical progressors (TP) with an average duration of 8–10 years (Qu, 2008).

The risk of developing early symptomatic AIDS is set at between 4.5% and 7% (Grinsztejn, 2014: 40 observed cases out of 886 in the group with early ART initiation versus 61 out of 877 in the delayed group).

Terminal AIDS has a duration of one month (Kwong, 2010) and the risk of developing terminal AIDS from early symptomatic AIDS is set at 32.09% as this was the case fatality proportion estimated for AIDS in a recent study (Serraino, 2010).

Model input summary

Table 1. Transition probabilities and distributions used in the outcome tree

Health outcome (Health state)	Distribution of health states in health outcome	Transition probability	Source/assumption
Persistent HIV infection			No cure available
(Rapid progressors)	5–15%		Qu, 2008
(Typical progressors)	70–90%		Qu, 2008
(Long-term non-progressors)	5–15%		Qu, 2008; Herida, 2006
AIDS early symptomatic		4.5–7%	Grinsztejn, 2014
AIDS terminal phase		32.09%	Serraino, 2010
CFR from AIDS		100%	

Table 2. Disability weights and duration

	Disability Weight (DW) (Haagsma, 2015)		Duration

Health outcome<br>(Health state)	DW	Label	In years	Source
Persistent HIV infection	0.108<br>(0.089-0.132)	HIV/AIDS cases, receiving ARV treatment		
(Rapid progressors)			2-5	Qu, 2008
(Typical progressors)			8-10	Qu, 2008
(Long-term non-progressors)			17.2	Qu, 2008; Herida, 2006
Permanent ARV treatment	0.108<br>(0.089-0.132)	HIV/AIDS cases, receiving ARV treatment	Remaining life expectancy	Assuming ARV treatment has optimal effectiveness and good compliance
AIDS early symptomatic	0.351<br>(0.299–0.394)	HIV cases, symptomatic, pre-AIDS	5.36	Herida, 2006
AIDS terminal phase	0.574<br>(0.518–0.635)	AIDS cases, not receiving ARV treatment	0.08	Kwong, 2010

References

Aghaizu A, Brown AE, Nardone A, Gill ON, Delpech VC & contributors. HIV in the United Kingdom 2013 - Report: data to end 2012. November 2013. Public Health England, London. Available at: https://www.gov.uk/government/uploads/system/uploads/attachment_data/file/326601/HIV_annual_report_2013.pdf

Barre-Sinoussi F, Chermann JC, Rey F, Nugeyre MT, Chamaret S, Gruest J, et al. Isolation of a T-lymphotropic retrovirus from a patient at risk for acquired immune deficiency syndrome (AIDS). Science 1983, 220:868-871.

Bhaskaran K, Hamouda O, Sannes M, Boufassa F, Johnson AM, Lambert PC, et al. for the CASCADE Collaboration. Changes in the Risk of Death After HIV Seroconversion Compared With Mortality in the General Population. JAMA. 2008;300(1):51-59. doi:10.1001/jama.300.1.51.

Cameron DW, Simonsen JN, D'Costa LJ, Ronald AR, Maitha GM, Gakinya MN, et al. Female to male transmission of human immunodeficiency virus type 1: risk factors for seroconversion in men. Lancet 1989, 2:403-407.

Cleghorn FR, Reits Jr. MS, Popovic M, Gallo RC. Human Immunodeficiency Viruses. In Principles and Practice of Infectious Diseases. Volume 2. Edited by Mandell GL, Bennet JE, Dolin R. Pennsylvania: Elsevier Inc.; 2005: 2119-2132

HIV/AIDS. Available at: http://www.who.int/topics/hiv_aids/en/

Del Rio C, Curran JW. Epidemiology and Prevention of Acquired Immunodeficiency Virus Infection. In Principles and Practice of Infectious Diseases. Volume 1. Edited by Mandell GL, Bennet JE, Dolin R. Pennsylvania: Elsevier Inc.; 2005: 1477-1506

European Centre for Disease Prevention and Control. Monitoring implementation of the Dublin Declaration on Partnership to Fight HIV/AIDS in Europe and Central Asia: 2014 progress report, in press.

Gallo RC, Sarin PS, Gelmann EP, Robert-Guroff M, Richardson E, Kalyanaraman VS, et al. Isolation of human T-cell leukemia virus in acquired immune deficiency syndrome (AIDS). Science 1983, 220:865-867.

Grinsztejn B, Hosseinipour M, Ribaudo H, Havlir D, Cohen M, et al. (2014). Effects of Early versus Delayed Initiation of Antiretroviral Therapy on HIV Clinical Outcomes: Results from the HPTN 052. The Lancet Infectious Diseases, Volume 14, Issue 4, April 2014, Pages 281-290

Haagsma JA, Maertens de Noordhout C, Polinder S, Vos T, Havelaar AH, Cassini A, Devleesschauwer B, Kretzschmar ME, Speybroeck N, Salomon JA. Assessing disability weights based on the responses of 30,660 people from four European countries. Population Health Metrics 2015; 13: 10

Herida M, Larsen C, Lot F, Laporte A, Desenclos JC, Hamers FF. Cost-effectiveness of HIV post-exposure prophylaxis in France. AIDS 2006, 20:1753-1761.

Kwong JC, Crowcroft NS, Campitelli MA, Ratanasingham S, Daneman N, Deeks SL, et al. Ontario Burden of Infectious Disease Study. In: Ontario Burden of Infectious Disease Study (ed.), Vol. OAHPP/ECES Report. City: Ontario Agency for Health Protection and Promotion and the Institute for Clinical Evaluative Sciences; 2010.

Laga M, Manoka A, Kivuvu M, Malele B, Tuliza M, Nzila N,et al. Non-ulcerative sexually transmitted diseases as risk factors for HIV-1 transmission in women: results from a cohort study. AIDS 1993, 7:95-102.

Merson MH, Piot P. Global Perspectives on Human Immunodeficiency Virus Infection and Acquired Immunodeficiency Syndrome. In Principles and Practice of Infectious Diseases. Volume 1. Edited by Mandell GL, Bennet JE, Dolin R. Pennsylvania: Elsevier Inc.; 2005: 1456-1506

Qu W, Robinson M, Zhang FJ. Factors influencing the natural history of HIV-1 infection. Chin Med J (Engl) 2008, 121:2613-2621.

Serraino D, Bruzzone S, Zucchetta A, Suligoi B, De Paoli A, Pennazza S, et al. Elevated risks of death for diabetes mellitus and cardiovascular diseases in Italian AIDS cases. AIDS Research and Therapy 2010, 7(11)

Sterling TR, Chaisson RE: General Clinical Manifestations of Human Immunodeficiency Virus Infection (Including the Acute Retroviral Syndrome and Oral, Cutaneous, Renal, Ocular and Cardia Diseases). In Principles and Practice of Infectious Diseases. Volume 1. Edited by Mandell GL, Bennet JE, Dolin R. Pennsylvania: Elsevier Inc.; 2005: 1547-1574

Influenza

In most cases influenza infection in humans is uncomplicated and self-limiting within a few days or weeks, but for some patients the disease is fatal. Approximately one third of influenza infections are mild or asymptomatic, to the extent that infected persons do not even see a doctor (Hayward, 2010; Hayward, 2014). Our model assumes a mean duration of five days (Nicholson, 2003).

Wielders et al. (2010) included four different outcomes and their long-term sequelae following acute illness. These were pneumonia, otitis media, acute respiratory distress syndrome (ARDS) and sepsis. The frequency of other post-infectious complications following an influenza infection is low and these were therefore disregarded in the current study. From a clinical perspective, the acute manifestations of the disease often occur in concomitance as complicated cases

Based on information derived from the General Practice Research Database (GPRD), Meier et al. (2000) estimated the number of patients consulting a doctor with symptoms of influenza-like illness (ILI) who developed complications. The percentages were based on subjects who had at least one clinical diagnosis of influenza or influenza-like-illness (ILI) recorded in the GPRD between 1991 and 1996. In addition to the wide range of national case definitions, estimated consultation rates will also vary among countries due to differences in consultation behaviour, estimation procedure (estimation of incidence, given that many surveillance systems are based on sentinel reporting), vaccination coverage (although vaccination has a limited impact on the number of consultations) and obligatory doctor visits for absence from work or school (Harbers, 2005; Meijer et al., 2006). Therefore, doctor consultations were not considered to be indicative of acute complicated influenza disease.

Given very little specific information on the ratio of complicated/uncomplicated acute disease, no distinction was made between these and the variability was accounted for by including all possible manifestations in the disability weight (mild, moderate and severe): 0.051 (0.007–0.125).

Case fatality ratio

Research has shown that clinicians often attribute influenza-related deaths to a pre-existing underlying condition rather than to influenza (Zucs et al., 2005). Therefore, it is difficult to identify true mortality due to influenza only. Distinguishing further between mortality due to influenza with or without complications such as cardiac problems or pneumonia is even more difficult. Therefore in the current study only one category of death was considered, encompassing all causes which, in the model, occur shortly after infection.

For the Netherlands, it was estimated that during the period 1967–1989 the overall impact of influenza on mortality was greater than registered mortality by a factor of 3.6 (Sprenger et al., 1993). Using this multiplication factor for more recent data may overestimate the number of deaths due to influenza, because in many Member States today vaccination coverage is considerably higher than in the period 1967– 1989. In the study by Sprenger et al. almost half of the non-registered influenza deaths were registered as deaths from heart disease, approximately 25% from lung disease and approximately 30% from other diseases (Sprenger et al., 1993). Recently, time series analysis has also been used to estimate mortality attributable to influenza and other respiratory pathogens (van den Wijngaard et al., 2010).

In about 0.1% of all influenza cases the disease will be fatal (Flu.gov, 2012). This includes both uncomplicated and complicated influenza cases.

Approximately 90% of persons with influenza as cause of death were aged ≥ 65 years (Webster, 2013). Therefore, given that the case fatality proportion for influenza is age-dependent, we modelled the age-specific risk according to the observed mortality data in Estonia, Germany and the Netherlands (see Table 3) (CBS, 2009).

Risk of complications

The most vulnerable populations in terms of complications following influenza are children aged under one 1 year and adults over 65 years, pregnant woman, and people of any age with comorbid illnesses (Rothberg et al., 2008).

The most common complications of influenza are secondary bacterial infections, especially otitis media and pneumonia (van Steenbergen, et al., 2006). It is estimated that 0.65% of influenza cases develop otitis media and 0.36% pneumonia (Meier et al., 2000). Secondary

bacterial pneumonia most often complicate the condition 4–14 days after primary seasonal influenza infection (Rothberg et al., 2008). Neurological complications such as encephalopathy (Reye's syndrome), encephalomyelitis, transverse myelitis, aseptic meningitis, focal neurological disorders, and Guillain-Barré syndrome most often appear in small children (Rothberg et al., 2010). The incidence of neurological complications among <5 years was estimated to be 4 per 100 000 (Newland, 2007).

Wielders et al. (2010) assumed that about 1.23% of all influenza cases develop pneumonia. Earlier, van Lier et al. (2007) assumed that this fraction was 0.36%. In most cases the disease will be self-limiting within a few days, and only in a few cases will it be fatal. According to Murray et al. (1996) long-term outcomes of pneumonia in developed countries are very rare and can be disregarded when estimating disease burden.

Wielders et al. (2010) assumed that 0.65% of influenza cases will develop otitis media as a complication of influenza. Most affected persons will fully recover, but 0.006% of otitis media cases will develop deafness as a life-long disability (Murray, 1996). Given the very low risk, we considered this complication as negligible.

A few cases will develop sepsis during an influenza infection, estimated at 0.0097% of all cases (Wielders, 2010). In some cases the disease will be fatal but again, since there was no detailed information available on the percentage, we assumed that fatal cases would be included in the death estimate related to influenza. Long-term disability was estimated to occur in 82% of patients surviving sepsis (Korosec Jagodic, 2006). However, given the fact that sepsis is caused by bacteria giving rise to super-infections possibly related to other factors, the long-term sequelae of sepsis are not considered to be part of the burden of influenza infections.

Acute respiratory distress syndrome (ARDS) and life-long disability

Following Wielders et al. (2010), we assumed that 0.023% of influenza cases will develop ARDS as a complication of influenza. We assumed that the risk of developing ARDS changes according to age (Manzano, 2005). Wielder's study, however, does not consider cases <15 years and in order to account for these, we also included a study on younger populations (Zimmerman, 2009). We combined the ARDS incidence from the two studies, added them together and estimated the age-group risk of developing ARDS (see Table 4).

In a few cases the disease will be fatal. However, having no detailed information on the specific risk, we assumed that fatal cases would be included in the death estimate related to influenza. Around 30–55% (Hopkins, 1999; Mikkelsen, 2012) of patients surviving ARDS will have developed disabilities related to cognitive impairments at one year follow-up. Therefore, in our model, we estimated that 0.007–0.013% of all symptomatic influenza cases will develop cognitive sequelae assumed to be permanent.

Model input summary

Table 1. Transition probabilities used in the outcome tree

Health outcome (Health state)	Distribution of health states in health outcome	Transition probability	Source/assumption
Permanent disability due to ARDS		0.007–0.013% Age dep. (Table 4)	Wielders, 2010; Manzano, 2005; Hopkins, 1999; Mikkelsen, 2012
Fatal cases		0.10% Age dep. (Table 3)	Flu.gov, 2012; observed cases

Table 2. Disability weights and duration

Health outcome (Health state)	Disability Weight (DW) (Haagsma, 2015)		Duration	
	DW	Label	In years	Source
Symptomatic infection	0.051 (0.007–0.125)	Infectious disease, acute episode, from mild to severe	0.014	Nicholson, 2003
Permanent disability due to ARDS	0.056 (0.044–0.067)	Motor plus cognitive impairments, mild	Remaining life expectancy	Hopkins, 1999; Mikkelsen, 2012

Table 3. Age group distribution of 0.1% risk of fatal cases

Age	%
0	0.58
01-04	0.51
05-09	0.24
10-14	0.27
15-19	0.24

20-24	0.33
25-29	0.31
30-34	0.33
35-39	0.75
40-44	1.15
45-49	1.56
50-54	1.53
55-59	2.21
60-64	3.23
65-69	4.54
70-74	5.22
75-79	11.42
80-84	18.72

85+	46.85

Source: based on all reported fatal influenza cases in Estonia, Germany and the Netherlands for the years 2005–2007.

Table 4. Age group distribution of 0.007–0.013% risk of developing ARDS

Age	%
0-14	7.21
15-29	2.59
30-44	7.66
45-59	12.17
60-74	28.73
≥75	41.63

References

CBS (Statistics Netherlands). Causes of death, 2009. Available at: http://statline.cbs.nl/StatWeb/

U.S. Department of Health & Human Services, Flu.gov (US). Community strategy for pandemic influenza mitigation. 2007 Feb.www.flu.gov/professional/community/mitigation.html [cited 12 March 2010]

Haagsma JA, Maertens de Noordhout C, Polinder S, Vos T, Havelaar AH, Cassini A, Devleesschauwer B, Kretzschmar ME, Speybroeck N, Salomon JA. Assessing disability weights based on the responses of 30,660 people from four European countries. Population Health Metrics 2015; 13: 10

Harbers MM. Influenza: are there international differences? In: Public Health Forecast, National Compass Public Health [Available at http://www.rivm.nl/vtv/object_document/o1736n18081.html]. 2005. Bilthoven: National Institute for Public Health and the Environment (RIVM).

Hayward AC et al. The community burden of influenza and Influenza-Like-Illness in England – Early results from the MRC Flu Watch Study. 2010. Personal communication with Professor Hayward.

Hayward AC, Fragaszy EB, Bermingham A, Wang L, Copas A, Edmunds WJ, et al. Comparative community burden and severity of seasonal and pandemic influenza: Results of the Flu Watch cohort study. Lancet Respir. Med. 2014, 2014, doi:10.1016/S2213-2600(14)70034-70037.

Hopkins RO, Weaver LK, Pope D, Orme JF Jr., Bigler ED, Larson-Lohr V. Neuropsychological sequelae and impaired health status in survivors of severe acute respiratory distress syndrome. American Journal of Respiratory and Critical Care Medicine. 1999;160(1):50–56.

Korosec Jagodic H, Jagodic K, Podbregar M. Long-term outcome and quality of life of patients treated in surgical intensive care: a comparison between sepsis and trauma. Crit Care 2006;10: R134.

Manzano F, Yuste E, Colmenero M, Garcia-Horcajadas A, Rivera R, Fernandez-Mondejar E. Granada Respiratory Failure Study Group: Incidence of acute respiratory distress syndrome and its relation to age. J Crit Care. 2005;20:274–80.

Meier CR, Napalkov PN, Wegmuller Y, Jefferson T, Jick H. Population-based study on incidence, risk factors, clinical complications and drug utilisation associated with influenza in the United Kingdom. Eur J Clin Microbiol Infect Dis 2000. 19 (11): 834-42.

Meijer A, Paget WJ, Meerhoff TJ, Brown C, Meuwissen LE, Van Der Velden J. Epidemiological and virological assessment of influenza activity in Europe, during the 2004-2005 winter. Euro Surveill 2006;11(5): 111-8.

Mikkelsen ME, Christie JD, Lanken PN, Biester RC, Thompson BT, Bellamy SL, et al. The adult respiratory distress syndrome cognitive outcomes study: long-term neuropsychological function in survivors of acute lung injury. Am J Respir Crit Care Med. 2012;17:1307–1315. doi: 10.1164/rccm.201111-2025OC.

Murray CJL, Lopez AD (eds.) (1996). Global health statistics: a compendium of incidence, prevalence and mortality estimates for over 200 conditions (Global burden of disease and injuries series; II). Cambridge: Harvard School of Public Health on behalf of the World Health Organization and the World Bank.

Neurologic Complications in Children Hospitalized with Influenza: Characteristics, Incidence, and Risk Factors. Newland JG, Laurich VM, Rosenquist AW, Heydon K, Licht DJ, Keren R et al. The Journal of Pediatrics, 2007; Vol. 150, 3,306–310

Nicholson KG, Wood JM, Zambon M. Influenza. Lancet 2003; 362:1733-45.

Rothberg MB, Haessler SD, Brown RB (2008). Complications of viral influenza. Am J Med 121(4): 258-64.

Rothberg MB, Haessler SD (2010). Complications of seasonal and pandemic influenza. Crit Care Med 38(4 Suppl): e91-7.

Sprenger MJ, Mulder PG, Beyer WE, Van Strik R, Masurel N (1993). Impact of influenza on mortality in relation to age and underlying disease, 1967-1989. Int J Epidemiol 22(2): 334-40.

van den Wijngaard CC, van Asten L, Meijer A, van Pelt W, Nagelkerke NJ, Donker GA, et al (2010). Detection of excess influenza severity: associating respiratory hospitalization and mortality data with reports of influenza-like illness by primary care physicians. Am J Public Health 100(11): 2248-54.

van Lier EA, Havelaar AH (2007). Disease burden of infectious diseases in Europe: a pilot study. Bilthoven: National Institute for Public Health and the Environment (RIVM) (RIVM report 215011001).

van Steenbergen J, Timen EA (eds) (2006). Guidelines Infectious Disease Control Edition 2006. Bilthoven: LCI, Coordinator Infectious Disease Control for the Netherlands.

Webster, Monto, Bracciale, Lamb. Textbook of Influenza. 2nd Edition, 2013. Wiley Blackwell Ed.

Wielders CCH, van Lier EA, van 't Klooster TM, van Gageldonk-Lafeber AB, van den Wijngaard CC, Haagsma JA, et al. The burden of 2009 pandemic influenza A(H1N1) in the Netherlands. Eur J Public Health 2010. http://dx.doi.org/10.1093/eurpub/ckq187

Zimmerman JJ, Akhtar SR, Caldwell E, Rubenfeld GD. Incidence and outcomes of pediatric acute lung injury. Pediatrics. 2009;124:87–95.

Zucs P, Buchholz U, Haas W, Uphoff H. Influenza associated excess mortality in Germany, 1985-2001. Emerg Themes Epidemiol 2005.2:6.

Invasive haemophilus influenza disease

The major disease burden of invasive H. influenzae infection occurs in children under five years (Fogarty, 1995). The most harmful complication is bacteraemia, which is accompanied by a focal infection such as meningitis, pneumonia, or cellulitis in 30–50% of cases (Devarajan, 2009).

Risk of complications

Meningitis is the principal clinical presentation of invasive disease, but bone and joint infections, pneumonia, epiglottitis, cellulitis and septicaemia can also occur. Skin and soft tissue infections may occur in around 6% of patients, followed by a limited number of sequelae (Otero Reigada, 2005). Only the invasive forms are considered as health states in the model.

To estimate the risk of meningitis we used the surveillance data reported in the ECDC Invasive Disease Surveillance report on clinical presentations of the acute symptomatic disease (ECDC, 2013a; ECDC, 2013b). Reported data indicates that meningitis and septicaemia occur together in 0–1% of cases, whereas meningitis alone occurs in 15–18% (15% in 2010, 18% in 2011) of cases, resulting in an overall risk of 15– 18% of developing meningitis. The risk of developing meningitis during the acute phase of the disease is age-specific. Age and gender-specific data were extracted from ECDC's TESSy database on the meningitis complications of IHID for 2010 and 2011 (see Table 4). The risk of developing the long-term sequelae is age and gender-specific.

Long-term sequelae

Bacterial meningitis may cause long-term sequelae and permanent disabilities. To investigate this we extracted the risk of developing these complications after meningitis episodes from Edmond et al. (Edmond, 2010).

Meningitis accounts for various long-term sequelae (each of which is multiplied by the risk of developing meningitis during the acute phase of the disease: 15–18%): cognitive difficulties (0.17–0.20%), seizure disorders (0.23–0.27%), hearing loss (0.48–0.58%), motor deficit (0.33– 0.40%), visual disturbance (0.08–0.09%), behavioural problems (0.32–0.38%), clinical impairments (0.18–0.22%) and multiple impairments (0.39–0.47%) (Edmond, 2010).

Case fatality proportion

The parameters for the case fatality proportion were based on data for EU/EEA countries in 2011, see Table 3 (ECDC, 2013).

Model input summary

Table 1. Transition probabilities used in the outcome tree

Health outcome<br><br>(Health state)	Distribution of health states in health outcome	Transition probability	Source/assumption
Hearing loss		0.48-0.58%	Edmond, 2010

Cognitive difficulties		0.17-0.20%	Edmond, 2010
Seizure disorder		0.23-0.27%	Edmond, 2010
Motor deficit		0.33-0.40%	Edmond, 2010
Visual disturbance		0.08-0.09%	Edmond, 2010
Behavioural problems		0.32-0.38%	Edmond, 2010
Clinical impairments		0.18-0.22%	Edmond, 2010
Multiple impairments		0.39-0.47%	Edmond, 2010
Fatal cases due to symptomatic infection		See Table 3 (5.4-19.5%)	ECDC, 2013

Table 2. Disability weights and duration

Health outcome (Health state)	Disability Weight (DW) (Haagsma, 2015)		Duration	
	DW	Label	In years	Source
Symptomatic infection	0.655 (0.579-0.727)	Intensive care unit admission	0.019	Tunkel, 2004 Assuming the duration of antimicrobial therapy

Permanent disability following meningitis			Remaining life expectancy	
1. Hearing loss	0.008-0.103	From lowest to highest hearing loss related DWs		
2. Cognitive difficulties	0.044-0.188	From lowest to highest intellectual disability related DWs		
3. Seizure disorder	0.07 (0.057-0.088)	Generic uncomplicated disease: worry and daily medication		
4. Motor deficit	0.011-0.421	From lowest to highest motor impairment related DWs		
5. Visual disturbance	0.004-0.171	From lowest to highest vision impairment related DWs		
6. Behavioural problems	0.088 (0.07-0.108)	Subacute sclerosing panencephalitis – phase 1 (assuming best fitting health state description)		
7. Clinical impairments	0.004-0.421	From lowest to highest DW included in this model		
8. Multiple impairments	0.004-0.421	From lowest to highest DW included in this model		

Table 3. CFR following symptomatic infection

Age	CFR
0	19.5%
1-4	6.5%
5-14	5.7%
15-64	5.4%
≥65	15%

Table 4. Age specific distribution per gender of the 15-18% risk of developing meningitis manifestation during the symptomatic infection (TESSy 2010-2011)

Age group		%

	F	M
0	15.69	17.12
01-04	15.69	18.49
05-09	2.61	5.48
10-14	1.31	2.74
15-19	1.31	2.74
20-24	2.61	4.11
25-29	0.00	0.00
30-34	3.27	1.37
35-39	1.96	4.79
40-44	3.92	7.53
45-49	3.92	8.22
50-54	7.19	2.74
55-59	7.19	2.74
60-64	3.27	4.79
65-69	10.46	3.42
70-74	11.11	5.48
75-79	5.23	4.11
80-84	2.61	1.37
85+	0.65	2.74
Total	100	100

References

Buysse CM, Oranje AP, Zuidema E, Hazelzet JA, Hop WC, Diepstraten AF, et al. Long-term skin scarring and orthopaedic sequelae in survivors of meningococcal septic shock. Arch Dis Child. 2009 May;94(5):381-6.

Devarajan VR. Haemophilus Influenzae Infections. 2009. http://emedicine.medscape.com/article/218271-overview

Edmond K, Clark A, Korczak VS, Sanderson C, Griffiths UK, Rudan I. Global and regional risk of disabling sequelae from bacterial meningitis: a systematic review and meta-analysis. Lancet Infect Dis. 2010 May;10(5):317-28.

Eidelman LA, Putterman D, Putterman C, Sprung CL. The Spectrum of Septic Encephalopathy: Definitions, Etiologies, and Mortalities. JAMA. 1996;275(6):470-473. doi:10.1001/jama.1996.03530300054040.

European Centre for Disease Prevention and Control. Surveillance of invasive bacterial diseases in Europe, 2010. Stockholm: ECDC; 2013.

European Centre for Disease Prevention and Control. Surveillance of invasive bacterial diseases in Europe, 2011. Stockholm: ECDC; 2013.

Fogarty J, Moloney AC and Newell JB. The epidemiology of Haemophilus influenzae type b disease in the Republic of Ireland. Epidemiol. Infect. (1995). 114. 451-463.

Haagsma JA, Maertens de Noordhout C, Polinder S, Vos T, Havelaar AH, Cassini A, Devleesschauwer B, Kretzschmar ME, Speybroeck N, Salomon JA. Assessing disability weights based on the responses of 30,660 people from four European countries. Population Health Metrics 2015; 13: 10

Jit M. The risk of sequelae due to pneumococcal meningitis in high-income countries: a systematic review and meta-analysis. J Infect 2010;61(2):114-24.

Otero Reigada MC, Piqueras Arenas AI, Ferrer Lorente B, Pérez-Tamarit D, Asensi Botet F, Santos Durantez M. Skin and soft tissue infections caused by Haemophilus influenzae type b over a 30 year period. An Pediatr (Barc). 2005 Jul;63(1):29-33.

Tunkel AR, Hartman BJ, Kaplan SL, Kaufman BA, Roos KL, Scheld WM, et al. Practice guidelines for the management of bacterial meningitis. Clin Infect Dis 2004, 39(9):1267–1284.

Ziaja M. Septic encephalopathy. Curr Neurol Neurosci Rep. 2013;13:383

Invasive meningococcal disease (IMD)

As many as 10% of adolescents and adults are asymptomatic transient carriers of N. meningitidis, most strains of which are not pathogenic. In more than 99% of the cases the infection is asymptomatic, but about 1% of the those infected develop acute illness (CDC, 2009). Invasive disease usually requires a seven-day course of antibiotic therapy (Brigham & Sandora, 2009; Tunkel 2004), but may also result in lifelong major sequelae.

Risk of complications

Meningitis is the most common manifestation of invasive disease, and may occur in 47.3% of all patients suffering from N. meningitidis symptomatic infection and in 52.2% of the patients who develop bacteraemia. It always follows hematogenous dissemination, which occurs in 91% of all patients suffering from symptomatic infection. Sepsis occurs in 5–20% of patients with invasive disease (CDC, 2009). Complications are also possible with non-invasive disease; pneumonia occurs in 6% of symptomatic infections, otitis media in 1% of cases and epiglottitis, which is rare, in 0.3% of all manifestations (CDC, 2009).

We decided to use surveillance data reported to TESSy on clinical presentations of the acute symptomatic disease to estimate the risk of meningitis (ECDC, 2013). Reported data indicates that meningitis and septicaemia together occur in 17–18% of cases, whereas meningitis alone occurs in 43–45% of cases, resulting in an overall risk of 60–63% of developing meningitis. The risk of developing meningitis during the acute phase of the disease is age-specific. Age-specific data were extracted for each gender from ECDC's TESSy database on the meningitis complications of IMD for 2010 and 2011 (see Table 4). The risk of developing long-term sequelae is age and gender-specific.

Long term sequelae

Bacterial meningitis may cause long-term sequelae and permanent disabilities. The risk of developing these complications after meningitis episodes was extracted from Edmond et al. (Edmond, 2010).

Meningitis accounts for various long-term sequelae (each of which is multiplied by the risk of developing meningitis during the acute phase of the disease: 60–63%): cognitive difficulties (0.96–1.01%), seizure disorders (0.3–0.35%), hearing loss (1.56–1.64%), motor deficit (0.6– 0.63%), visual disturbance (0.9–0.95%), behavioural problems (0.36–0.38%), clinical impairments (0.12–0.13%) and multiple impairments (0.78-0.82%) (Edmond, 2010).

Case fatality proportion

The parameters for the case fatality ratio were based on data for EU/EEA countries in 2011, see Table 3 (ECDC, 2013).

Model input summary

Table 1. Transition probabilities used in the outcome tree

Health outcome (Health state)		Distribution of health states in health outcome		Transition probability		Source/assumption

Hearing loss		1.56–1.64%	Edmond, 2010
Cognitive difficulties		0.96–1.01%	Edmond, 2010
Seizure disorder		0.3–0.35%	Edmond, 2010
Motor deficit		0.6–0.63%	Edmond, 2010
Visual disturbance		0.9–0.95%	Edmond, 2010
Behavioural problems		0.36–0.38%	Edmond, 2010
Clinical impairments		0.12–0.13%	Edmond, 2010
Multiple impairments		0.78–0.82%	Edmond, 2010
Fatal cases due to symptomatic infection		See Table 3 (6.9-17.1%)	ECDC, 2013

Table 2. Disability weights and duration

Health outcome (Health state)	Disability Weight (DW) (Haagsma, 2015)		Duration	
	DW	Label	In years	Source
Symptomatic infection	0.655 (0.579-0.727)	Intensive care unit admission	0.019	

				Tunkel, 2004 Assuming the duration of antimicrobial therapy
Permanent disability following meningitis:			Remaining life expectancy	
1. Hearing loss	0.008-0.103	From lowest to highest hearing loss related DWs		
2. Cognitive difficulties	0.044-0.188	From lowest to highest intellectual disability related DWs		
3. Seizure disorder	0.07 (0.057-0.088)	Generic uncomplicated disease: worry and daily medication		
4. Motor deficit	0.011-0.421	From lowest to highest motor impairment related DWs		
5. Visual disturbance	0.004-0.171	From lowest to highest vision impairment related DWs		
6. Behavioural problems	0.088 (0.07-0.108)	Subacute sclerosing panencephalitis – phase 1 (assuming best fitting health state description)		
7. Clinical impairments	0.004-0.421	From lowest to highest DW included in this model		
8. Multiple impairments	0.004-0.421	From lowest to highest DW included in this model		

Table 3. CFP following symptomatic infection

Age	CFR
0	7.8%

1-4	6.9%
5-14	5.6%
15-24	9.5%
25-49	8.9%
50-64	7.6%
≥65	17.1%

Table 4. Age specific distribution per gender of the 60-63% risk of developing meningitis manifestation during the symptomatic infection (TESSy 2010-2011)

Age group	%	
	F	M
0	16.22	16.64
01-04	18.19	23.79
05-09	7.13	8.65
10-14	5.90	4.46
15-19	14.53	15.97
20-24	7.21	8.06
25-29	3.93	3.62
30-34	2.62	3.04

35-39	2.05	1.69
40-44	2.54	1.84
45-49	2.81	2.01
50-54	3.47	2.43
55-59	3.28	2.43
60-64	1.97	1.42
65-69	1.88	1.42
70-74	1.97	0.76
75-79	2.05	1.35
80-84	1.31	0.34
85+	0.93	0.08
Total	100	100

References

Brigham KS, Sandora TJ. Neisseria meningitidis: epidemiology, treatment and prevention in adolescents. Curr Opin Pediatr. 2009 Aug;21(4):437-43.

Buysse CM, Oranje AP, Zuidema E, Hazelzet JA, Hop WC, Diepstraten AF, et al. Long-term skin scarring and orthopaedic sequelae in survivors of meningococcal septic shock. Arch Dis Child. 2009 May;94(5):381-6.

Centers for Disease Control and Prevention. Epidemiology and Prevention of Vaccine-Preventable Diseases. Atkinson W, Wolfe S, Hamborsky J, McIntyre L, eds. 11th ed. Washington DC: Public Health Foundation, 2009.

Edmond K, Clark A, Korczak VS, Sanderson C, Griffiths UK, Rudan I. Global and regional risk of disabling sequelae from bacterial meningitis: a systematic review and meta-analysis. Lancet Infect Dis. 2010 May;10(5):317-28.

Eidelman LA, Putterman D, Putterman C, Sprung CL. The Spectrum of Septic Encephalopathy: Definitions, Etiologies, and Mortalities. JAMA. 1996;275(6):470-473. doi:10.1001/jama.1996.03530300054040.

European Centre for Disease Prevention and Control. Surveillance of invasive bacterial diseases in Europe, 2010. Stockholm: ECDC; 2013.

European Centre for Disease Prevention and Control. Surveillance of invasive bacterial diseases in Europe, 2011. Stockholm: ECDC; 2013.

Haagsma JA, Maertens de Noordhout C, Polinder S, Vos T, Havelaar AH, Cassini A, Devleesschauwer B, Kretzschmar ME, Speybroeck N, Salomon JA. Assessing disability weights based on the responses of 30,660 people from four European countries. Population Health Metrics 2015; 13: 10

Jit M. The risk of sequelae due to pneumococcal meningitis in high-income countries: a systematic review and meta-analysis. J Infect 2010;61(2):114-24.

Tunkel AR, Hartman BJ, Kaplan SL, Kaufman BA, Roos KL, Scheld WM, et al. Practice guidelines for the management of bacterial meningitis. Clin Infect Dis 2004, 39(9):1267–1284.

Ziaja M. Septic encephalopathy. Curr Neurol Neurosci Rep. 2013;13:383.

Invasive pneumococcal disease

Despite the large number of serogroups and serotypes known, most cases of invasive pneumococcal disease (IPD) on a global scale are attributed to the 1, 3, 4, 6, 7, 9, 14, 18, 23 (Jefferson, 2006) and 19a serogroups.

Risk of complications

Invasive pneumococcal infection can manifest as meningitis, bacteraemic pneumonia, bacteraemia without a focus, and bacteraemia with a focus other than the lungs or meninges (e.g. endocarditis, osteomyelitis, and arthritis, although rare). Complications, such as pneumonia or otitis media, are also possible with non-invasive forms of infection but are not considered in this study.

Most observed complications of invasive bacterial diseases, including IPD, are related to the meningitis event. The risk of meningitis was estimated using surveillance data reported to TESSy on clinical presentations of the acute symptomatic disease (ECDC, 2013) and it was found that 10% of IPD cases are reported to manifest meningitis. The risk of developing meningitis during the acute phase of the disease is age- specific. Age and gender-specific data were extracted from ECDC's TESSy database on the risk of developing meningitis for IPD cases from 2010 to 2011 (see Table 4). The risk of developing long-term sequelae is age and gender-specific.

Long-term sequelae

Bacterial meningitis may cause long-term sequelae and permanent disabilities. In order to account for these, information was extracted on the risk of developing permanent sequelae from Edmond et al. (Edmond, 2010).

Meningitis can result in various long-term sequelae: cognitive difficulties (4.2%), seizure disorders (2.5%), hearing loss (7.5%), motor deficit (5.8%), visual disturbance (1.1%), behavioural problems (4.6%) multiple (5.7%) and clinical impairments (3.3%) (Edmond, 2010). Therefore, we assumed that 10% of all IPD patients would be at risk of developing long-term sequelae.

Case fatality proportion

The case fatality proportion for invasive pneumococcal disease has been estimated at 18% in a population-based study of 19 000 people (Harboe, 2009); however, important differences were observed between age groups, with a lower (3%) mortality rate observed in children <5 years. The overall lethality rate due to bacteraemia is about 10–20% (CDC, 2009; Rudan, 2009; Lin, 2010; Saldías, 2009) and may be as high as 60% among elderly patients (CDC, 2009).

Overall mortality due to endocarditis is 50%, but it can reach 60–65% in children (Elward, 1990). The case-fatality proportion for pneumococcal meningitis is about 30%, but may be as high as 80% in elderly persons (CDC, 2009; Burckhardt et al. 2010). The parameters for the case fatality proportion were based on data for EU/EEA countries in 2011, see Table 3 (ECDC, 2013).

Model input summary

Table 1. Transition probabilities used in the outcome tree

Health outcome (Health state)	Distribution of health states in health outcome	Transition probability	Source/assumption

Hearing loss		0.75%	Edmond, 2010
Cognitive difficulties		0.42%	Edmond, 2010
Seizure disorder		0.25%	Edmond, 2010
Motor deficit		0.58%	Edmond, 2010
Visual disturbance		0.11%	Edmond, 2010
Behavioural problems		0.46%	Edmond, 2010
Clinical impairments		0.33%	Edmond, 2010
Multiple impairments		0.57%	Edmond, 2010
Fatal cases due to symptomatic infection		See Table 3 (3-24%)	Harboe, 2009

Table 2. Disability weights and duration

Health outcome (Health state)	Disability Weight (DW) (Haagsma, 2015)	Duration

	DW	Label	In years	Source
Symptomatic infection	0.655 (0.579-0.727)	Intensive care unit admission	0.027-0.038	Tunkel, 2004<br>Assuming the duration of antimicrobial therapy
Permanent disability following meningitis			Remaining life expectancy	
1. Hearing loss	0.008-0.103	From lowest to highest hearing loss related DWs		
2. Cognitive difficulties	0.044-0.188	From lowest to highest intellectual disability related DWs		
3. Seizure disorder	0.07 (0.057-0.088)	Generic uncomplicated disease: worry and daily medication		
4. Motor deficit	0.011-0.421	From lowest to highest motor impairment related DWs		
5. Visual disturbance	0.004-0.171	From lowest to highest vision impairment related DWs		
6. Behavioural problems	0.088 (0.07-0.108)	Subacute sclerosing panencephalitis – phase 1 (assuming best fitting health state description)		
7. Clinical impairments	0.004-0.421	From lowest to highest DW included in this model		
8. Multiple impairments	0.004-0.421	From lowest to highest DW included in this model		

Table 3. CFP following symptomatic infection

Age	CFR
0	5.1%
1-4	3%
5-14	7.1%
15-64	8%

≥65	14.3%

Table 4. Age specific distribution per gender of the 60-63% risk of developing meningitis manifestation during the symptomatic infection (TESSy 2010-2011)

Age group	%	
	F	M
0	10.37	11.45
01-04	8.13	8.52
05-09	2.70	3.56
10-14	1.54	2.54
15-19	0.39	1.57
20-24	1.29	1.22
25-29	1.02	2.23
30-34	2.45	3.56
35-39	3.29	5.68
40-44	3.74	5.58
45-49	5.47	6.90
50-54	6.70	7.31
55-59	9.21	7.76
60-64	11.28	9.02

65-69	9.78	7.04
70-74	7.60	6.24
75-79	6.51	4.91
80-84	5.15	2.98
85+	3.36	1.93
Total	100	100

References

Burckhardt I, Burckhardt F, Van Der Linden M, Heeg C, Reinert RR. Risk factor analysis for pneumococcal meningitis in adults with invasive pneumococcal infection. Epidemiol Infect 2010;138(9):1353-8.

Buysse CM, Oranje AP, Zuidema E, Hazelzet JA, Hop WC, Diepstraten AF, et al. Long-term skin scarring and orthopaedic sequelae in survivors of meningococcal septic shock. Arch Dis Child. 2009 May;94(5):381-6.

Centers for Disease Control and Prevention. Epidemiology and Prevention of Vaccine-Preventable Diseases. Atkinson W, Wolfe S, Hamborsky J, McIntyre L, eds. 11th ed. Washington DC: Public Health Foundation, 2009.

Edmond K, Clark A, Korczak VS, Sanderson C, Griffiths UK, Rudan I. Global and regional risk of disabling sequelae from bacterial meningitis: a systematic review and meta-analysis. Lancet Infect Dis. 2010 May;10(5):317-28.

Eidelman LA, Putterman D, Putterman C, Sprung CL. The Spectrum of Septic Encephalopathy: Definitions, Etiologies, and Mortalities. JAMA. 1996;275(6):470-473. doi:10.1001/jama.1996.03530300054040.

Elward K, Hruby N, Christy C. Pneumococcal endocarditis in infants and children: Report of a case and review of the literature. Pediatr Infect Dis J. 1990;9:652.

European Centre for Disease Prevention and Control. Surveillance of invasive bacterial diseases in Europe, 2010. Stockholm: ECDC; 2013.

European Centre for Disease Prevention and Control. Surveillance of invasive bacterial diseases in Europe, 2011. Stockholm: ECDC; 2013.

Haagsma JA, Maertens de Noordhout C, Polinder S, Vos T, Havelaar AH, Cassini A, Devleesschauwer B, Kretzschmar ME, Speybroeck N, Salomon JA. Assessing disability weights based on the responses of 30,660 people from four European countries. Population Health Metrics 2015; 13: 10

Harboe ZB, Thomsen RW, Riis A, et al. Pneumococcal Serotypes and Mortality following Invasive Pneumococcal Disease: A Population-Based Cohort Study. PLoS Med. 2009 May; 6(5): e1000081.

Jefferson T, Ferroni E, Curtale F, et al. Streptococcus pneumoniae in western Europe: serotype distribution and incidence in children less than 2 years old. Lancet Infect Dis 2006;6:405-10.

Jit M. The risk of sequelae due to pneumococcal meningitis in high-income countries: a systematic review and meta-analysis. J Infect 2010;61(2):114-24.

Lin SH, Lai CC, Tan CK, Liao WH, Hsueh PR. Outcomes of hospitalized patients with bacteraemic and non-bacteraemic community-acquired pneumonia caused by Streptococcus pneumoniae. Epidemiol Infect. 2010 Oct 26:1-10. [Epub ahead of print].

Rudan I, Campbell H. The deadly toll of S pneumoniae and H influenzae type b. Lancet 2009; 374(9.693):854-6.

Saldías PF, Viviani GP, Pulgar BD, Valenzuela FF, Paredes ES, Díaz PO. Prognostic factors and mortality in immunocompetent adult patients hospitalized with community-acquired pneumococcal pneumonia. Rev Med Chil 2009; 137(12):1.545-52.

Tunkel AR, Hartman BJ, Kaplan SL, Kaufman BA, Roos KL, Scheld WM, et al: Practice guidelines for the management of bacterial meningitis. Clin Infect Dis 2004, 39(9):1267–1284.

Ziaja M. Septic encephalopathy. Curr Neurol Neurosci Rep. 2013; 13: 383

Legionnaires' disease

Since 2008, the EU case definition focuses solely on Legionnaires' disease, dismissing Pontiac fever cases. Therefore, the present disease outcome tree focuses only on Legionnaires' disease and its sequelae.

Legionnaires' disease is mostly observed in the elderly and conditions associated with immunodeficiency constitute a risk for Legionnaires'.

In rare cases, Legionnaires' disease may also cause extra-pulmonary symptoms, mainly developing cardiac complications (WHO, 2007). Myocarditis, pericarditis, post-cardiomyotomy syndrome or endocarditis are examples of such manifestations although, according to other studies, most of these complication are related to nosocomial infections (Stout, 1997). Extra-pulmonary manifestations are also often observed in immunocompromised patients. For the purpose of this disease model, we focus on community-acquired Legionnaires' cases and extra- pulmonary manifestations are excluded.

Legionnaires' disease causes acute consolidating pneumonia. In most cases, and without testing for the causative agent, pneumonia arising from infection with Legionella pneumophila cannot be distinguished from other types of pneumonia. Symptoms of Legionnaires' disease are an unproductive cough, chest pain, shortness of breath, myalgia and digestive symptoms such as diarrhoea, vomiting and nausea. Patients may also present neurological symptoms such as confusion or deliria (WHO, 2007).

In many cases, the acute phase requires admission to hospital. Studies have shown that in-patient stays in the hospital vary between eight and 13 days (Lettinga, 2002a; von Baum, 2008). However, it may take more than 90 days to recover to the premorbid health state (Lettinga, 2002a) and roentgenographic clearance can take 2–4 months (Edelstein, 2008). For the model the duration of acute Legionnaires' disease is set at 8– 13 days, as stated in one European study (Lettinga, 2002a).

We consider three different health states occurring during the acute phase of the disease, mild (outpatient, uncomplicated cases), moderate (hospitalised, complicated cases not admitted to an intensive-care unit) and severe (complicated cases admitted to an intensive care unit). Studies have shown that hospitalisation is required in 69–74% of Legionnaires' cases (von Baum, 2008; Garcia-Fulgueiras, 2003). We therefore assume that 26–31% of cases will be mild. Moreover, it is shown that 30% of hospitalised cases require a stay in an intensive-care unit (ICU) (Lettinga, 2002b), thus the proportion of complicated cases (not requiring ICU) is set to 46.7–53.2% and those requiring ICU is set to 20.7– 22.2% of all symptomatic infections.

The case-fatality proportion (CFP) differs widely and is associated with the severity level. The CFP for severe cases was found to be higher, ranging from 10 to 30% (Lettinga, 2002b; Benin, 2002; Falco, 1991). In a review conducted by WHO, case-fatality proportions of community- acquired infections ranged from 5 to 10% (WHO, 2007; Benin, 2002; Howden, 2003). The European working group on Legionella infections (EWGLI) suggested a 12% case-fatality in Europe (von Baum, 2008). In our model, CFP for uncomplicated and complicated cases not requiring a stay in an ICU is set at 5–12% and 10–30% for severe cases requiring an ICU.

Risk of complications

Legionnaires' disease is associated with pulmonary (e.g. severe respiratory failure, pulmonary abscess and pleural empyema), cardiac (e.g. acute pericarditis, myocarditis), neuromuscular (e.g. headache, confusion, fatigue) and renal (e.g. acute renal failure, interstitial nephritis) complications. Multi-organ involvement or septic shock are also possible. In the outcome-tree these complications are not treated separately as they are part of the acute phase of Legionnaires' disease.

Studies on the long-term sequelae of Legionnaires' are scarce, however some reported consequences up to two years after the initial infection (Lattimer, 1979). Two studies reported fatigue in 58–81% of cases, concentration problems and memory loss in 6–81%, muscle/joint pain or muscle weakness in 25–79% and post-traumatic stress disorder in 15% (Lattimer, 1979; Lettinga, 2002a). Given the lack of evidence on the causality of Legionnaires' and the long-term consequences, these were not considered.

Model input summary

Table 1. Transition probabilities and distributions used in the outcome tree

Health outcome (Health state)	Distribution of health states in health outcome	Transition probability	Source/assumption
Symptomatic infection			von Baum, 2008; Garcia-Fulgueiras, 2003; Lettinga, 2002b
(Uncomplicated)	26–31%		
(Complicated)	46.7–53.2%		
(Complicated ICU)	20.7–22.2%		
Fatal cases			Lettinga, 2002b; Benin, 2002; Falco, 1991; WHO, 2007; Benin, 2002; Howden, 2003; von Baum, 2008
(Uncomplicated)		5–12%	
(Complicated)		5–12%	
(Complicated ICU)		10–30%	

Table 2. Disability weights and duration

Health outcome (Health state)	Disability Weight (DW) (Haagsma, 2015)		Duration	
	DW	Label	In years	Source
Symptomatic infection		Infectious disease, acute episode, moderate	0.022–0.036	Lettinga, 2002a; von Baum, 2008
(Uncomplicated)	0.051 (0.039–0.06)	Infectious disease, acute episode, severe		
(Complicated)	0.125 (0.104–0.152)	Intensive care unit admission		
(Complicated ICU)	0.655 (0.579–0.727)			

References

Edelstein PH. Detection of antibodies to Legionella. In Manual of Molecular and Clinical Laboratory Immunology. Edited by Detrick B, Hamilton R, Folds J. Washington, DC: ASM Press; 2006: 468-476

Haagsma JA, Maertens de Noordhout C, Polinder S, Vos T, Havelaar AH, Cassini A, Devleesschauwer B, Kretzschmar ME, Speybroeck N, Salomon JA. Assessing disability weights based on the responses of 30,660 people from four European countries. Population Health Metrics 2015; 13: 10

World Health Organization: and the prevention of legionellosis. Geneva; 2007.

Stout JE, Yu VL. Legionellosis. N Engl J Med 1997, 337:682-687.

Lettinga KD, Verbon A, Nieuwkerk PT, Jonkers RE, Gersons BP, Prins JM, et al. Health-related quality of life and post-traumatic stress disorder among survivors of an outbreak of Legionnaires disease. Clin Infect Dis 2002, 35:11-17.

von Baum H, Ewig S, Marre R, Suttorp N, Gonschior S, Welte T, et al. Community-acquired Legionella pneumonia: new insights from the German competence network for community acquired pneumonia. Clin Infect Dis 2008, 46:1356-1364.

Edelstein PH: Legionnaires' Disease: History and Clinical findings. In Legionella: Molecular Microbiology. Edited by Heuner K, Swanson M. Portland, Oregan: Caister Academic Press; 2008: 1-17

Garcia-Fulgueiras A, Navarro C, Fenoll D, Garcia J, Gonzalez-Diego P, Jimenez-Bunuales T, et al. Legionnaires' disease outbreak in Murcia, Spain. Emerg Infect Dis 2003, 9:915-921.

Lettinga KD, Verbon A, Weverling GJ, Schellekens JF, Den Boer JW, Yzerman EP, et al. Legionnaires' disease at a Dutch flower show: prognostic factors and impact of therapy. Emerg Infect Dis 2002, 8:1448-1454.

Benin AL, Benson RF, Arnold KE, Fiore AE, Cook PG, Williams LK, et al. An outbreak of travel-associated Legionnaires disease and Pontiac fever: the need for enhanced surveillance of travel-associated legionellosis in the United States. J Infect Dis 2002, 185:237-243.

Howden BP, Stuart RL, Tallis G, Bailey M, Johnson PD. Treatment and outcome of 104 hospitalized patients with Legionnaires' disease. Intern Med J 2003, 33:484-488.

Falco V, Fernandez de Sevilla T, Alegre J, Ferrer A, Martinez Vazquez JM. Legionella pneumophila. A cause of severe community-acquired pneumonia. CHEST Journal 1991, 100:1007-1011.

Lattimer GL, Rhodes LV, 3rd, Salventi JS, Galgon JP, Stonebraker V, Boley S, et al. The Philadelphia epidemic of Legionnaire's disease: clinical, pulmonary, and serologic findings two years later. Ann Intern Med 1979, 90:522-526.

Listeriosis

Acquired listeriosis

Listeriosis is an infection caused by the gram-positive bacterium Listeria monocytogenes. The infection is generally asymptomatic but can become extremely severe in immunocompromised patients, pregnant women and their foetuses/newborn and elderly. The severity of the disease is related to its invasiveness: if the infection is not invasive, it will generally cause mild or no symptoms and therefore no burden (with the exception of acute gastroenteritis if a person ingests a large amount of bacteria). Therefore, it is not surprising that most notified cases are invasive listeriosis diseases, hence complicated ones. In order to estimate the number of complicated cases we referred to the US Centers for Disease Control's 2012 and 2011 Listeriosis Annual Surveillance Summaries (CDC, 2014), reporting 95–97% of cases as invasive, and we applied this to the proportion of complicated symptomatic cases.

Manifestations of listeriosis are meningitis, septicaemia, pneumonia, and gastroenteritis. Based on reports from enhanced surveillance in the Netherlands (Doorduyn, 2006 a,b) and a Gamma distribution used to express the uncertainty, Kemmeren et al. (Kemmeren, 2006) and Haagsma et al. (Haagsma, 2009) estimated the distribution of these health states for acquired listeriosis. However, from a clinical perspective it is conceivable that most cases present a mixed form of the disease and isolates are available from multiple anatomical sites. We therefore defined symptomatic infections as either complicated (invasive) or uncomplicated.

In order to determine those long-term sequelae which are linked only to the manifestation of meningitis, we looked at enhanced surveillance in a few European countries, however data on the risk of developing meningitis during invasive listeriosis disease was inconsistent. Therefore, we referred to CDC enhanced surveillance in the USA from 2007 to 2012 and estimated that 13–18% of invasive (complicated) symptomatic cases would present with meningitis (CDC, 2014).

In the current model, the age-specific case fatality proportion related to listeriosis is derived from cases of acquired listeriosis notified to TESSy from 2009 to 2013 (see Table 3) by all EEA Member States except Bulgaria and Lithuania because they report only aggregate data. The case fatality proportion is applied to complicated cases only.

Perinatal listeriosis

Perinatal listeriosis encompasses both pregnant women and their foetuses or newborns. Of the pregnant women with listeriosis, around two out of three will present with prodromal influenza-like symptoms such as fever, chills and headache. Three to seven days after the prodromal symptoms, the pregnant woman may abort the foetus or have premature labour (Gellin, 1989). To the mother, listeriosis is rarely life- threatening, however, infection in the first trimester of pregnancy may result in spontaneous abortion and, in later stages, in stillbirth or a critically ill newborn (Farber, 1991a). Newborns may present with an early-onset or a late-onset form of listeriosis. Early-onset listeriosis is defined as a case of symptomatic listeriosis in a newborn that is less than seven days old. Early-onset listeriosis is acquired by the foetus prenatally. Newborns with early-onset listeriosis mostly develop sepsis and meningitis (Farber, 1991b; Mylonakis, 2002). Late-onset listeriosis is defined as symptomatic listeriosis in a newborn during the first eight to 28 days of life. In this case, the unborn child is infected during childbirth when passing through the birth canal. Newborns with late-onset listeriosis are usually born healthy and at full term, but are at higher risk of developing meningitis during their first weeks of life (Farber, 1991a).

In the current study, the disease burden for health outcomes of early- and late-onset listeriosis are combined into one category. Based on data reported to TESSy between 2009 and 2013, the case fatality proportion was set to 18.71%.

Risk of complications

Long-term sequelae due to meningitis may occur, and will therefore be considered in the outcome tree. The frequency of other post-infectious complications following listeriosis is low (Haagsma, 2009) and therefore they have been disregarded in the current study.

According to Aouaj et al. (Aouaj, 2002), 20% of all listeriosis cases in their study are perinatal. Therefore, of the 147 cases analysed for long- term outcomes (Aouaj, 2002), we estimated that there were 118 acquired cases (29 perinatal). The study stated that 15 (12.7%) of the total number of acquired listeriosis cases presenting meningitis developed neurological long-term sequelae.

Given that 13–18% of all acute cases present meningitis, the risk of developing neurological long-term sequelae from all cases of complicated acquired listeriosis is 1.65–2.29%.

Similarly, knowing that seven of the 29 perinatal listeriosis cases (24%) developed long-term neurological sequelae and that all acute cases present meningitis, the risk of developing life-long neurological disabilities from a perinatal listeria infection is 24%.

Model input summary

Table 1. Transition probabilities and distributions used in the outcome tree

Health outcome<br><br>(Health state)	Distribution of health states<br><br>in health outcome	Transition probability	Source/assumption
Acquired listeriosis			
Symptomatic infection<br><br>(Uncomplicated)<br><br>(Complicated)	3–5%<br><br>95–97%		CDC, 2014

Fatal cases		Age dependent (Table 3)	TESSy 2009–2013
Permanent disability following meningitis		1.65–2.29% of complicated cases	Aouaj, 2002; CDC 2014
Perinatal listeriosis			
Fatal cases		18.71%	TESSy 2009–2013
Permanent disability due to meningitis		24%	Aouaj, 2002

Table 2. Disability weights and duration

Health outcome (Health state)	Disability Weight (DW) (Haagsma, 2015)		Duration	
	DW	Label	In years	Source
Acquired listeriosis				
Symptomatic infection (Uncomplicated) (Complicated)	0.149 (0.12–0.182)<br>0.655 (0.579–0.727)	Diarrhoea, moderate<br>Intensive care unit admission	0.02–0.5	Kemmeren, 2006 Haagsma, 2009;
Permanent disability following meningitis	0.011–0.421	From lowest to highest motor and cognitive difficulties	Remaining life expectancy	
Perinatal listeriosis				
Symptomatic infection	0.655 (0.579–0.727)	Intensive care unit admission	0.02–0.5	Kemmeren 2006 & Haagsma 2009
Permanent disability due to meningitis	0.011–0.421	From lowest to highest motor and cognitive difficulties	Remaining life expectancy	

Table 3. Age-group acquired listeriosis case fatality proportion based on cases and deaths notified to TESSy (2009– 2013)

Age groups	%
0	11.90
1-4	0.00
5-9	5.88
10-14	20.00
15-19	13.16
20-24	1.75
25-29	4.10
30-34	1.39
35-39	8.40
40-44	12.50
45-49	14.08
50-54	16.59
55-59	13.77

60-64	18.16
65-69	15.65
70-74	15.17
75-79	17.83
80-84	17.35
>85	23.15
All ages	15.74

References

Aouaj Y, Spanjaard L, van Leeuwen N, Dankert J. (2002). Listeria monocytogenes meningitis: serotype distribution and patient characteristics in The Netherlands, 1976-95. Epidemiol Infect, 128(3):405-409.

CDC (2014). Centers for Disease Control and Prevention, National Surveillance of Bacterial Foodborne Illnesses (Enteric Diseases) National Listeria Surveillance. Available at: http://www.cdc.gov/nationalsurveillance/listeria-surveillance.html [accessed 2 October 2014].

Doorduyn Y, de Jager CM, van der Zwaluw WK, Wannet WJ, van der Ende A, Spanjaard L, et al. (2006a). Invasive Listeria monocytogenes infections in the Netherlands, 1995-2003. Eur J Clin Microbiol Infect Dis, 25(7):433-442.

Doorduyn Y, de Jager CM, van der Zwaluw WK, Wannet WJ, van der Ende A, Spanjaard L, et al (2006b). First results of the active surveillance of Listeria monocytogenes infections in the Netherlands reveal higher than expected incidence. Euro Surveill, 11(4):E060420 060424.

Doorduyn Y, De Jager CM, Van der Zwaluw WK, Wannet WJB, Heuvelink AE, Van der Ende A, et al. (2007). Intensieve surveillance van Listeria monocytogenes in Nederland, 2006. Infectieziekten Bulletin, 9:308-314.

Farber JM, Peterkin PI (1991a). Listeria monocytogenes, a food-borne pathogen. Microbiol Rev, 55(3):476-511.

Farber JM, Peterkin PI, Carter AO, Varughese PV, Ashton FE, Ewan EP. (1991b). Neonatal listeriosis due to cross-infection confirmed by isoenzyme typing and DNA fingerprinting. J Infect Dis, 163(4):927-928.

Haagsma JA, van der Zanden BP, Tariq L, van Pelt W, van Duynhoven YTHP, Havelaar AH (2009). Disease burden and costs of selected foodborne pathogens in the Netherlands, 2006. Bilthoven: National Institute of Public Health and Environment. RIVM rapport 330331001. Available online at: www.rivm.nl/bibliotheek/rapporten/330331001.pdf

Haagsma JA, Maertens de Noordhout C, Polinder S, Vos T, Havelaar AH, Cassini A, Devleesschauwer B, Kretzschmar ME, Speybroeck N, Salomon JA. Assessing disability weights based on the responses of 30,660 people from four European countries. Population Health Metrics 2015; 13: 10

Kemmeren JM, Mangen MJJ, van Duynhoven YTHP, Havelaar AH. (2006). Priority setting of foodborne pathogens: disease burden and costs of selected enteric pathogens. Bilthoven: National Institute of Public Health and Environment: RIVM rapport 330080001. Available online at: www.rivm.nl/bibliotheek/rapporten/330080001.pdf

Lorber B (1997). Listeriosis. Clin Infect Dis, 24(1):1-9; quiz 10-11.

McLauchlin J, Mitchell RT, Smerdon WJ, Jewell K. (2004). Listeria monocytogenes and listeriosis: a review of hazard characterisation for use in microbiological risk assessment of foods. Int J Food Microbiol, 92(1):15-33.

Mylonakis E, Paliou M, Hohmann EL, Calderwood SB, Wing EJ. (2002). Listeriosis during pregnancy: a case series and review of 222 cases. Medicine (Baltimore), 81(4):260-269.

Measles

According to the US Centers for Disease Control and Prevention (CDC, 2012), approximately 30% of reported symptomatic measles cases have one or more complications. The most important complications are: otitis media (occurring in approximately 10% of infected cases), encephalitis (0.1% of cases), and post-infectious encephalomyelitis (0.1–0.3% of cases). Other complications of acute measles include pneumonia (5–6% of untreated cases; Kabra, 2008; CDC, 1991) and diarrhoea (8%) (CDC, 2012). Convulsions are also a relatively frequent complication (5% of cases; Miller, 1978). Complications during pregnancy occur in up to 30% of women with severe measles (Atmar, 1992).

Complications occurring during the acute phase of the disease may overlap and cannot be treated as independent. Two health states were therefore used in our model: complicated and uncomplicated. We derived the risk of complications from data reported to TESSy between 2006 and 2013. Given the high number of cases notified to TESSy without information on complications and in order to account for this uncertainty we included two scenarios. We estimated the proportion of cases reported as uncomplicated out of the number of known cases as 57.24% (excluding cases for which complications were reported as unknown or left blank). We then added the uncomplicated cases to the unknown and blank and obtained the total number 88.64% (assuming that all unknown and blank cases were uncomplicated).

In the model, the rare permanent disabilities due to otitis media, encephalitis, post-infectious encephalomyelitis and subacute sclerosing panencephalitis (SSPE) (van Steenbergen, 2006) are treated as distinct sequelae.

Otitis media and permanent disability due to otitis media

The health state otitis media occurs in around one in ten cases of acute measles and can result in permanent hearing loss (CDC, 2011). The probability of developing permanent disability due to otitis media is 0.01% (CDC, 1991) of all cases of otitis media, therefore the overall risk of developing a permanent disability has been set to 0.001%.

Encephalitis and permanent disability due to encephalitis

Encephalitis occurs in approximately 0.1% of acute symptomatic cases (Weissbrich, 2003; Beutels, 2002; Miller, 1957). Long-term sequelae of measles encephalitis are reported to occur in 20–30% of measles-related encephalitis cases (Beutels, 2002; Filia, 2007); therefore the transition probability for the health outcome 'permanent disability due to encephalitis' was set to 0.02-0.033%.

Encephalitis of the delayed type (Barthez Carpentier, 1992) can occur after acute illness in immunocompromised patients and may occur after asymptomatic infection (Kidd, 2003). Because of the specific population affected, and its relative rarity, the outcome tree was not modified accordingly.

Post-infectious encephalomyelitis (PIE) and permanent disability due to PIE

Post-infectious encephalomyelitis occurs in 1–3 per 1 000 infected persons, usually three to ten days after the onset of rash. Higher rates of PIE due to measles occur in adolescents and adults than in school-aged children (Perry & Halsey, 2004). The condition is associated with demyelination and is thought to have an autoimmune basis. A total of 33% of those afflicted with PIE who survive have lifelong neurological sequelae, including severe retardation, motor impairment, blindness and sometimes hemiparesis (Perry & Halsey, 2004). The transition probability in the model for developing the health outcome 'permanent disability due to PIE' was set to the range 0.033–0.1%.

Subacute sclerosing panencephalitis (SSPE)

On average, the symptoms of SSPE begin seven to ten years after measles infection, but they can appear anytime from one month to 27 years after infection (CDC, 2012).

Various estimates are available for the proportion of cases that develop the SSPE health outcome. SSPE is observed at a rate of 1 per 10 000– 20 000 (Weissbrich, 2003; Takasu, 2003; Bellini, 2005; Garg, 2008). In children who have previously had natural measles, the risk of developing SSPE is between 0.6 and 2.2 per 100 000 cases (Hosoya, 2006). Other estimates include: one SSPE case in every 100 000 cases of measles (Rezende, 1989); 4–11 cases of SSPE per 100 000 cases of measles (CDC, 2009); one in every 25 000 measles infections (Miller, 2004); one in 8 000 for children under two years (Miller, 1992; 2004) and a 16-fold greater risk for those infected under one year of age compared with those over five years (Miller, 1992). The risk of developing SSPE is known to be age-specific (Beutels, 2002; Farrington, 1991; Miller, 2004; CDC, 2012).Therefore, transitional probabilities in the model were also specified as age-dependent (see Table 3) (Beutels, 2002). In the model, the duration for this health outcome was specified as one to two years (CDC, 2012). In the model the transition probability from SSPE to death was set to 100%.

Case fatality proportion

Measles is fatal in approximately 0.05–0.1% of cases (Wolfson, 2007; Lozano, 2012). The risk of death is higher among young children and adults (CDC, 2012). According to CDC (CDC, 2012), the most common causes of death are pneumonia in children and acute encephalitis in adults, but due to the lack of specific data for different age groups we applied the same CFP for all the same age groups.

Model input summary

Table 1. Transition probabilities used in the outcome tree

Health outcome (Health state)	Distribution of health states in health outcome	Transition probability	Source/assumption

Symptomatic infection<br>(Complicated)<br>(Uncomplicated)	11.36-42.76%<br>57.24-88.64%		TESSy, 2006–2013
Permanent disability following otitis media		0.001%	CDC, 1991
Permanent disability following encephalitis		0.02–0.033%	Beutels, 2002; Filia, 2007
Permanent disability following PIE		0.033–0.1%	Perry & Halsey, 2004
SSPE		See Table 3	Beutels, 2002
Fatal cases following SSPE		100%	
Fatal cases following symptomatic infection		0.05–0.1%	Wolfson, 2007; Lozano, 2012

Table 2. Disability weights and duration

Health outcome (Health state)	Disability Weight (DW) (Haagsma, 2015)		Duration	
	DW	Label	In years	Source
Symptomatic infection<br>(Complicated)<br>(Uncomplicated)	0.125 (0.104-0.152)<br>0.051 (0.039-0.06)	Infectious disease, acute episode, severe<br>Infectious disease, acute episode, moderate	0.03	Kwong, 2012
Permanent disability due to otitis media	0.008-0.103	From lowest to highest hearing loss related DWs	Remaining life expectancy	
Permanent disability due to encephalitis	0.054-0.425	From lowest to highest Motor plus cognitive impairments related DWs	Remaining life	

			expectancy	
Permanent disability due to PIE	0.054-0.425	From lowest to highest Motor plus cognitive impairments related DWs	Remaining life expectancy	
Latency period before SSPE	0		0.082–27	CDC, 2012
SSPE	0.276 (0.088-0.543)	From Phase 1 to Phase 3 (median is Phase 2) of subacute sclerosing panencephalitis related DWs	1–2	CDC, 2012

Table 3. Transition probabilities subacute sclerosing panencephalitis (SSPE)

Age	%
0-4	0.0081
5-9	0.0011
≥10	0.0010

References

Barthez Carpentier MA, Billard C, Maheut J, Jourdan ML, Degenne D, Ruchoux MM, et al. Acute measles encephalitis of the delayed type: neuroradiological and immunological findings. Eur Neurol. 1992;32(4):235-7.

Bellini WJ, Rota JS, Lowe LE, et al (2005). Subacute sclerosing panencephalitis: More cases of this fatal disease are prevented by measles immunization than previously recognized. J Infect Dis 192:1686–1693.

Beutels P, Van Damme P, Van Casteren V, Gay NJ, De Schrijver K, Meheus A. The difficult quest for data on "vanishing" vaccine-preventable infections in Europe: the case of measles in Flanders (Belgium). Vaccine. 2002 Oct 4;20(29-30):3551-9.

Centers for Disease Control (CDC). Measles: United States, 1990. MMWR Morb Mortal Wkly Rep 1991;40:369–372.

Centers for Disease Control and Prevention. Epidemiology and Prevention of Vaccine-Preventable Diseases. Atkinson W, Wolfe S, Hamborsky J, McIntyre L, eds. 11th ed. Washington DC: Public Health Foundation, 2009

Centers for Disease Control and Prevention (CDC). The Pink Book: Course Textbook - 12th Edition Second Printing (May 2012).

Farrington CP. Subacute sclerosing panencephalitis in England and Wales: transient effects and risk estimates. Stat Med 1991;10:1733–1744;

Filia A, Brenna A, Panà A, Maggio Cavallaro G, Massari M, Ciofi degli Atti LM. Health burden and economic impact of measles-related hospitalizations in Italy in 2002–2003. BMC Public Health. 2007; 7: 169

Garg RK (2008). Subacute sclerosing panencephalitis. J Neurol 255(12):1861–1871.

Haagsma JA, Maertens de Noordhout C, Polinder S, Vos T, Havelaar AH, Cassini A, Devleesschauwer B, Kretzschmar ME, Speybroeck N, Salomon JA. Assessing disability weights based on the responses of 30,660 people from four European countries. Population Health Metrics 2015; 13: 10

Hosoya M. Measles encephalitis: direct viral invasion or autoimmune-mediated inflammation? Intern Med. 2006;45(14):841-2.

Kabra SK, Lodha R, Hilton DJ. Antibiotics for preventing complications in children with measles. Cochrane Database of Systematic Reviews 2008, Issue 3. Art. No.: CD001477. DOI: 10.1002/14651858.CD001477.pub3.

Kwong JC, Ratnasingham S, Campitelli MA, Daneman N, Deeks SL, et al. (2012) The Impact of Infection on Population Health: Results of the Ontario Burden of Infectious Diseases Study. PLoS ONE 7(9): e44103. doi:10.1371/journal.pone.0044103

Lozano R, Naghavi M, Foreman K, Lim S, Shibuya K, Aboyans V, et al. (Dec 15, 2012). Global and regional mortality from 235 causes of death for 20 age groups in 1990 and 2010: a systematic analysis for the Global Burden of Disease Study 2010. Lancet 380 (9859): 2095–128

Miller HG, Stanton JB, Gibbons JL. Acute disseminated encephalomyelitis and related syndromes. British medical journal.1957 Mar 23;1(5020):668-72

Miller CL. Severity of notified measles. British Medical Journal. 1978; 1(6122): 1253-1255.

Miller C, Andrews N, Rush M, Munro H, Jin L, Miller E. The epidemiology of subacute sclerosing panencephalitis in England and Wales 1990-2002. Arch Dis Child. 2004 Dec;89(12):1145-8.

Murray CJL, Lopez AD, editors. The global burden of disease: a comprehensive assessment of mortality and disability from diseases, injuries, and risk factors in 1990 and projected to 2020. Cambridge: Harvard University Press, 1996.

Rezende MA. Nursing care and the neurological sequelae of measles. Revista da Escola de Enfermagem USP. 1989 Apr;23(2):141-8.

Stein CE, et al, The Global Burden of Measles in the Year 2000—A Model that Uses Country-Specific Indicators. Journal of Infectious Diseases 2003; 187(Suppl 1):S8–14.

Takasu T, Mgone JM, Mgone CS, et al (2003). A continuing high incidence of subacute sclerosing panencephalitis (SSPE) in the eastern highlands of Papua New Guinea. Epidemiol Infect 131:887–898.

Tishler M, Abramov AL. Liver involvement in measles infection of young adults. Isr J Med Sci. 1983 Sep;19(9):791-3.

Wolfson LJ, Strebel PM, Gacic-Dobo M, Hoekstra EJ, McFarland JW, et al. (2007) Has the 2005 measles mortality reduction goal been achieved? A natural history modelling study. Lancet 369: 191–200

Zhou F, Reef S, Massoudi M, Papania MJ, Yusuf HR, Bardenheier B, et al. An Economic Analysis of the Current Universal 2-Dose Measles-Mumps- Rubella Vaccination Program in the United States. The Journal of Infectious Diseases. 2004 May 1;189 Suppl 1:S131-45.

Mumps

Mumps is symptomatic in 80% of infections (CDC, 2012), the main symptom being parotitis.

Risk of complications

The principal complications with mumps are orchitis, oorphoritis, meningitis, pancreatitis, and encephalitis.

Epididymo-orchitis occurs in 15–30% of adult men with mumps infection, but it is rare before puberty (Hviid, 2008). Oophoritis (ovarian inflammation), the counterpart of orchitis in females, is associated with pelvic pain and tenderness. It occurs in 5% of post-pubertal females (CDC, 2009).

Mumps meningitis is a benign entity with no significant risk of mortality or long-term sequelae. Even though cerebrospinal fluid pleiocytosis occurs in about half of the patients with mumps, clinical manifestations of meningitis arise in 1–10% of the cases (Hviid, 2008), and long-term morbidity is rare. Encephalitis occurs in 0.1% of acute cases (Hviid, 2008).

Acute pancreatitis, with symptoms of abdominal distention and pain, fever, nausea, and vomiting (Demirci, 2011), occurs in approximately 4% of mumps cases (Vanlioglu & Chua, 2011).

With mumps, the acute complications of symptomatic infections are considered as a single health state (complicated) because they can occur concomitantly.

Of all mumps infections, 40–50% may have only non-specific or primarily respiratory symptoms (CDC, 2012). Therefore, knowing that 20% of infections are asymptomatic, 32–40% of symptomatic cases were considered to be uncomplicated. Durations were set to 7–10 days for the uncomplicated cases and 7–14 days for the complicated ones.

Permanent deafness caused by mumps occurs with an estimated frequency of one in 20 000 cases (0.005%) and in 80% of the cases, hearing loss is monolateral (Hviid, 2008).

Case fatality proportion

Death is very rare in mumps cases and the mortality rate following encephalitis is 1.5%. Therefore, 0.0015% was used in the model for the risk of death resulting from all symptomatic infections. More than half of fatalities occur in patients over 19 years (Hviid, 2008; Demirci, 2011). This age distribution also applies to the symptomatic complicated cases (see Table 3).

Model input summary

Table 1. Transition probabilities used in the outcome tree

Health outcome<br>(Health state)		Distribution of health states in health outcome	Transition probability	Source/assumption

Symptomatic infection<br>(Uncomplicated)<br>(Complicated)	32–40%<br>60–68%		CDC, 2012
Permanent disability due to hearing loss		0.005%	Hviid, 2008
Fatal cases		0.0015%<br>Age dependent<br>(see Table 3)	Hviid, 2008<br>Assuming 1.5% of encephalitis cases (0.1%) become fatal

Table 2. Disability weights and duration

Health outcome (Health state)	Disability Weight (DW) (Haagsma, 2015)		Duration	
	DW	Label	In years	Source
Symptomatic infection<br>Uncomplicated<br>Complicated	0.051 (0.039-0.06)<br>0.125 (0.104-0.152)	Infectious disease, acute episode, moderate<br>Infectious disease, acute episode, severe	0.019-0.027<br>0.019-0.038	Hviid, 2008
Permanent hearing loss	0.008 (0.005-0.012)	Unilateral hearing loss	Remaining life expectancy	Hviid, 2008

Table 3. Age distribution – case fatality ratio

0-19	50
≥20	50

References

Centers for Disease Control and Prevention. Epidemiology and Prevention of Vaccine-Preventable Diseases. Atkinson W, Wolfe S, Hamborsky J, eds. 12th ed., second printing. Washington DC: Public Health Foundation, 2012.

Demirci CS, Abuhammour W, Quintana EC, Shahidi H, Wilkes G. Mumps. Available from http://emedicine.medscape.com/article/966678- overview [accessed 12 September, 2011].

Haagsma JA, Maertens de Noordhout C, Polinder S, Vos T, Havelaar AH, Cassini A, Devleesschauwer B, Kretzschmar ME, Speybroeck N, Salomon JA. Assessing disability weights based on the responses of 30,660 people from four European countries. Population Health Metrics 2015; 13: 10

Hviid A, Rubin S, Mühlemann K. Mumps. Lancet. 2008 Mar 15;371(9616):932-44.

Mandell GL, Bennet JE, Dolin R. Mandell, Douglas and Bennett's Principles and Practice of Infectious Disease 5th edition. Churchill Livingstone, 1999.

Vanlioglu B, Chua TC. Presentation of mumps infection as acute pancreatitis without parotitis. Pancreas. 2011 Jan;40(1):167-8.

Pertussis

Pertussis is principally toxin-mediated. Toxins paralyse the cilia of the respiratory tract cells, leading to the clinical features and complications of the disease. The clinical course of the illness is divided into three stages. The first one is the catarrhal stage, characterised by coryza, sneezing, low-grade fever and a mild, occasional cough. The cough gradually becomes more severe, and after 1–2 weeks, the paroxysmal stage begins, usually lasting one to six weeks. In the convalescent stage, which lasts two to three weeks, recovery is gradual and the cough becomes less paroxysmal. However, paroxysms often recur for many months after the onset of pertussis (CDC, 2009; Mandell, 1999).

Clinical manifestations of pertussis may be mild in adults and vaccinated children. Around 20% of infected persons develop mild/asymptomatic disease (Rothstein, 2005). Based on this finding, an asymptomatic proportion of 20% was specified in the model.

Risk of complications

The principal complications of pertussis are secondary infections, such as otitis media and pneumonia, neurological complications, such as seizures and encephalopathy. Other possible complications include physical sequelae of paroxysmal cough (e.g. subconjunctival haemorrhages, epistaxis, petechiae, central nervous system haemorrhage, pneumothorax and hernia) (CDC, 2009; Mandell, 1999).

Pneumonia can result from aspiration during whooping and vomiting or from impaired clearance mechanisms. It occurs in 5.2% of all patients (CDC, 2009), in up to 25% of cases reported in infants (Mandell, 1999), in 2–4% of individuals aged 10–19 years, in 2.7–5.5% of those over 20 years and in 5–9% of those over 30 years (Rothstein, 2005).

Approximately 4% of adolescents and adults with symptomatic pertussis infection develop otitis media (De Serres, 2000).

Neurological complications of pertussis are more common among infants. In children 12 months of age or younger with pertussis in the USA (1980–1989), convulsions occurred in 3.0% and encephalopathy in 0.9% of cases. Encephalopathy, febrile and afebrile convulsions occur infrequently in adults with pertussis (CDC, 2009), with encephalopathy observed in 0.1% of cases during the period 1997–2000 (CDC, 2009).

Seizures were reported among 0.8% of all pertussis cases in the period 1997–2000 (CDC, 2009).

Infants with pertussis are at greater risk of complications and permanent sequelae, however complications of pertussis, including serious ones, are not uncommon in adolescents and adults, especially the elderly. Complications occur in up to 23% of patients aged 19–83 years. Complications are more frequent in adults than in adolescents (28% compared to 16%) (CDC, 2009; Mandell, 1999; Rothstein, 2005).

Most complications occurring during the symptomatic acute disease phase overlap with one other. We therefore decided to aggregate all complicated cases into one health state. Risk of complications is reported to be 50% in infants (<1 year), 16% in children and adolescents and 28% in cases 20 years (CDC, 2013).

We assumed that in complete and active surveillance systems, those cases notified represent the complicated cases of pertussis. The United Kingdom has an enhanced surveillance system for pertussis where information is compiled from different sources. We therefore chose to consider the number of cases reported in the UK (2007–2013) as complicated. In order to estimate the proportion of complicated cases, we divided the number of cases reported in the UK by the estimated true incidence of pertussis derived from the literature: 71–507 per 100 000 10 years; 46 per 100 000 <10 years (Wirsing von Konig, 2002; Diez-Domingo, 2004) (see Table 3).

Case fatality proportion

Death from pertussis is rare beyond the age of 10 years, occurring in less than 0.1% of all cases, with older adults being at greater risk than younger adults (Rothstein, 2005). Pneumonia is a leading cause of death, but in a study of 99 patients aged 55–94 years who died of pertussis (Rothstein, 2005), intracranial haemorrhage was the cause of death for two of the four deaths thought to be associated with pertussis. Among patients who died, apnoea, pneumonia, seizures, and encephalopathy were reported for 58% (40 of 69), 54% (39 of 72), 21% (14 of 68), and 12% (7 of 57), respectively (Rothstein, 2005; Farizo, 1992).

‘The case fatality proportion in the United States between 1990 and 1996 was 0.2%. Eighty-four per cent of pertussis-related deaths occur in infants younger than six months of age’ (Ratnapalam, 2005).

In general, we considered that only complicated cases were at risk of dying. We used the CFP reported in the UK for deaths of infants <1 year old because of its comprehensive surveillance system, compiling data from different sources and deemed to be capturing approximately 94% of the cases in recent capture-recapture studies. There were 33 deaths due to pertussis reported to TESSy between 2007 and 2013 out of 1 791 cases. This resulted in a CFP of 1.84% which was applied to complicated cases <1 year.

We chose 0.1% of complicated cases for all other age groups.

Model input summary

Table 1. Transition probabilities used in the outcome tree

Health outcome<br><br>(Health state)	Distribution of health states in<br><br>health outcome	Transition probability	Source/assumption
Symptomatic infection<br><br>(Complicated)<br><br>(Uncomplicated)	Age dependent (see Table 3)<br><br>Remaining cases		CDC, 2013

Fatal cases		1.84% <1 yr.<br>0.1% ≥ 1 yr.	TESSy<br>Rothstein, 2005

Table 2. Disability weights and duration

Health outcome (Health state)	Disability Weight (DW) (Haagsma, 2015)		Duration	
	DW	Label	In years	Source
Symptomatic infection			0.077–0.211	CDC, 2009; Mandell, 1999
(Complicated)	0.125 (0.104–0.152)	Infectious disease, acute episode, severe		
(Uncomplicated)	0.051 (0.039–0.06)	Infectious disease, acute episode, moderate		

Table 3. Risk of complications

Age	%	
	Estimated from low true incidence	Estimated from high true incidence
0	28.04	
01-04	8.04	
05-09	5.85	
10-14	0.35	2.46
15-19	0.39	2.81
20-24	1.05	7.50
25-29	1.59	11.38
30-34	1.92	13.68
35-39	1.45	10.32
40-44	1.84	13.12
45-49	2.23	15.96
50-54	2.00	14.29
55-59	1.68	11.97

60-64	1.20	8.57
65-69	1.48	10.58
70-74	1.24	8.83
75-79	1.30	9.26
80-84	0.91	6.52
85+	0.54	3.88

References

Centers for Disease Control and Prevention. Epidemiology and Prevention of Vaccine-Preventable Diseases. Atkinson W, Wolfe S, Hamborsky J, McIntyre L, eds. 11th Edition. Washington DC: Public Health Foundation, 2009.

Centers for Disease Control and Prevention. http://www.cdc.gov/pertussis/about/complications.html Last update January 2013 [accessed 25 September 2014].

De Serres G, Shadmani R, Duval B, Boulianne N, Déry P, Douville Fradet M, et al. Morbidity of pertussis in adolescents and adults. J Infect Dis. 2000 Jul;182(1):174-9.

Diez-Domingo J, Ballester A, Baldo JM, Planelles MV, Villarroya JV, Alvarez T et al. Incidence of pertussis in persons < 15 years of age in Valencia, Spain: seroprevalence of antibodies to pertussis toxin (PT) in children, adolescents and adults. Journal of Infection (2004) 49, 242- 247

Farizo KM, Cochi SL, Zell ER, Brink EW, Wassilak SG, Patriarca PA. Epidemiological features of pertussis in the United States, 1980-1989. Clin Infect Dis. 1992 Mar;14(3):708-19.

Haagsma JA, Maertens de Noordhout C, Polinder S, Vos T, Havelaar AH, Cassini A, Devleesschauwer B, Kretzschmar ME, Speybroeck N, Salomon JA. Assessing disability weights based on the responses of 30,660 people from four European countries. Population Health Metrics 2015; 13: 10

Mandell GL, Bennet JE, Dolin R. Mandell, Douglas and Bennett's Principles and Practice of Infectious Disease 5th edition. Churchill Livingstone, 1999.

Ratnapalan S, Parkin PC, Allen U. Case 1: The deadly danger of pertussis. Paediatr Child Health. 2005 Apr;10(4):221-2

Rothstein E, Edwards K. Health burden of pertussis in adolescents and adults. Pediatr Infect Dis J. 2005 May;24(5 Suppl):S44-7. Wirsing von Konig CH, Halperin S, Riffelmann M, Guiso N. Pertussis of adults and infants. Lancet Infect Dis 2002; 2: 744-50.

Poliomyelitis

Poliomyelitis is an acute illness which may result from invasion of the gastro-intestinal tract by one of three types of polio virus. It usually affects small children under the age of three years. The virus is transmitted through contaminated food and water, and multiplies in the intestine, from where it can invade the nervous system. Transmission occurs through contact with faeces or pharyngeal secretions of an infected person. The incubation period ranges from three to 21 days, but may be longer. Cases are infectious from about ten days before to seven days after the onset of symptoms; however, carriers and some immuno-compromised persons may shed the virus in faeces for longer than six weeks (Howard, 2005).

Most infections are not clinically apparent; up to 95% of infections are asymptomatic (CDC, 2009).

Risk of complications

Clinical disease may range in severity from minor illness (abortive poliomyelitis), to non-paralytic poliomyelitis (aseptic meningitis) and paralytic poliomyelitis (Feigin, 2009).

Approximately 4–8% of polio infections consist of a non-specific 'minor illness' without clinical or laboratory evidence of central nervous system invasion (CDC, 2009; Feigin, 2009). This clinical presentation is known as abortive poliomyelitis, and is characterised by complete recovery in less than one week (CDC, 2009).

Nonparalytic aseptic meningitis (symptoms of stiffness of the neck, back, and/or legs) which usually follows several days after a prodrome similar to that of a minor illness, occurs in 1–2% of polio infections (CDC, 2009). Increased or abnormal sensations can also occur. Typically these symptoms will last from two to ten days, followed by complete recovery (CDC, 2009).

Less than 1% of all polio infections result in flaccid paralysis (CDC, 2009; Heymann, 2004). Paralytic symptoms generally begin one to ten days after prodromal symptoms and progress for two to three days. Generally, no further paralysis occurs after fever subsides (CDC, 2009). Many patients with paralytic poliomyelitis recover completely and, in most of them, muscle function returns to some degree. Weakness or paralysis 12 months after onset is usually permanent (CDC, 2009).

In acute flaccid paralysis (AFP), the legs are usually more often affected than the muscles of the upper body. However, the polio virus may invade the brain stem, potentially leading to breathing difficulty and even death. Symptoms include headache, gastro-intestinal disturbance, malaise and stiffness of the neck and back, with or without paralysis (American Academy of Pediatrics, 2006; Shibuya & Murray, 2002). Improvements are seen within the first six months (Farbu, 2013; Neumann, 2004). The principal complication is painful, acute, asymmetric paralysis of the arms or the legs, reaching its maximum extent over the course of three to four days and leading to permanent lameness of the affected limbs and breathing difficulties (UK Department of Health, 2006; WHO, 2014).

Given the estimates of symptomatic polio cases, we considered that on average 8.5% of infections are symptomatic (6–11%; CDC, 2011); hence 70.59% of cases on average will be abortive (uncomplicated), 17.65% will be non-paralytic and 11.76 will be paralytic.

According to WHO (WHO, 2014), 1 in 200 infections leads to irreversible paralysis. Given that 1% of all infections has a paralytic form, we considered that 50% of all paralytic forms would develop a permanent disability due to paralysis.

Post-polio syndrome is a long-term sequela that occurs 30–35 years after infection in approximately 25–50% of cases (Jubelt & Drucker, 1999). A slowly progressing condition, it can also occur in patients who have had the non-paralytic form of poliomyelitis. The most common symptoms include slow, progressive muscle weakness, fatigue (both generalised and muscular) and a gradual decrease in the size of muscles (muscle atrophy). Pain from joint degeneration and increasing skeletal deformities such as scoliosis (curvature of the spine) is common and may precede the weakness and muscle atrophy. Some individuals experience only minor symptoms while others develop visible muscle weakness and atrophy. Fatigue is clearly the most prominent manifestation, occurring in up to 80% of patients (Jubelt & Drucker, 1999). Post-polio syndrome is rarely life-threatening (NINDS, 2012).

Case fatality proportion

The case fatality proportion is 5–10% of paralytic forms (WHO, 2014).

Model input summary

Table 1. Transition probabilities used in the outcome tree

Health outcome (Health state)	Distribution of health states in health outcome	Transition probability	Source/assumption
Symptomatic infection		6-11%	
(Uncomplicated)	70.59%		CDC, 2009
(Non-paralytic poliomyelitis)	17.65%		CDC, 2009; Heymann, 2004
(Paralytic poliomyelitis)	11.76%		
Post-polio syndrome		25–50%	Jubelt & Drucker, 1999
Permanent disability following paralytic poliomyelitis		50%	WHO, 2014

Fatal cases following paralytic poliomyelitis		5-10%	WHO, 2014

Table 2. Disability weights and duration

Health outcome (Health state)	Disability Weight (DW) (Haagsma, 2015)		Duration	
	DW	Label	In years	Source
Symptomatic infection				
(Uncomplicated)	0.007 (0.005–0.01)	Infectious disease, acute episode, mild	0.019	CDC, 2009
(Non-paralytic poliomyelitis)	0.051 (0.039–0.06)	Infectious disease, acute episode, moderate	0.005–0.027	CDC, 2009
(Paralytic poliomyelitis)	0.125 (0.104–0.152)	Infectious disease, acute episode, severe	0.011–0.038	CDC, 2009
Permanent disability following paralytic poliomyelitis	0.067 (0.054–0.081)	Spinal cord lesion below neck level (treated)	Remaining life expectancy	
Latency period before PPS	0		30–35	Jubelt & Drucker, 1999
Post-polio syndrome (PPS)	0.344 (0.3–0.391)	Musculoskeletal problems, generalised, moderate	Remaining life expectancy	

References

American Academy of Pediatrics. Red Book: 2006 Report of the Committee on Infectious Diseases. 27th ed. Elk Grove Village. 2006.

Centers for Disease Control and Prevention (CDC). Epidemiology and Prevention of Vaccine-Preventable Diseases. Atkinson W, Wolfe S, Hamborsky J, McIntyre L, eds. 11th ed. Washington DC: Public Health Foundation, 2009.

Department of Health, UK. Immunisation against infectious disease (the Green Book). 3rd ed. London: The Stationery Office. 2006.

Feigin R, Cherry J, Demmler-Harrison GJ, Kaplan S. Feigin and Cherry's Textbook of Pediatric Infectious Diseases. Sixth Edition, 2009.

Farbu E. 2013. Post-polio Syndrome. In: JH Stone, M Blouin, editors. International Encyclopedia of Rehabilitation. Available online: http://cirrie.buffalo.edu/encyclopedia/en/article/138/

Haagsma JA, Maertens de Noordhout C, Polinder S, Vos T, Havelaar AH, Cassini A, Devleesschauwer B, Kretzschmar ME, Speybroeck N, Salomon JA. Assessing disability weights based on the responses of 30,660 people from four European countries. Population Health Metrics 2015; 13: 10

Heymann D, Aylward B. Poliomyelitis. Orphanet Encyclopedia. WHO. 2004.

Howard RS. Poliomyelitis and the post polio syndrome. BMJ 2005;330(7503):1314–1318.

Jubelt B, Drucker J. Poliomyelitis and the Post-Polio Syndrome in Motor Disorders. Philadelphia: Lippincott Williams and Wilkins. 1999. pp. 381.

Neumann D. Polio: its impact on the people of the United States and the emerging profession of physical therapy (PDF).The Journal of Orthopaedic and Sports Physical Therapy 2004; 34 (8): 479–92. PMID 15373011

Post-Polio Syndrome Fact Sheet. NINDS. 2012.

Shibuya K, Murray CJL. Poliomyelitis. In: Stein CE, Murray CJL, Lopez AD (eds). The Global Burden of Disease and Injuries Series, Volume 4: The Global Epidemiology of Infectious Diseases. WHO, 2002.

UNICEF Executive Board Special (2009). Focus Session on Global Health, with particular focus on polio eradication. Available online from: http://www.unicef.org/about/execboard/files/Special_focus_session-Background_SFS_English_8_June_09(1).pdf

World Health Organization (WHO). Poliomyelitis. Available from http://www.who.int/topics/poliomyelitis/en/ [accessed 19 September 2014].

Q fever

Q fever infection becomes symptomatic in 40% of cases (Dijkstra, 2012). Symptomatic infections are divided into two health states: uncomplicated and complicated (more severe cases) and the proportion of complications is based on the hospitalisation rate (2–5%) for Q fever (Maurin & Raoult, 1999; Raoult, 2005).

Around 1–2% of Q fever cases are fatal (ECDC, 2010). This CFR is applied to complicated cases only, based on the US Centers for Disease Control (CDC) Fact Sheet which states that 'the case fatality ratio for hospitalized patients is under 2%' (CDC, 2013).

Chronic Q fever

The transition probability that cases with symptomatic infections will develop chronic Q fever is set to 1.6% (1.5–2%) (van der Hoek, 2011; ECDC, 2010). Due to the lack of evidence, development of chronic Q fever was not associated with asymptomatic Q fever (ECDC, 2010). The average duration of chronic Q fever before developing symptoms is 0.5 years (0.08–1.5 years) (Fenollar, 2001) and this is included in the burden calculation as it reduces the life expectancy of later health outcomes.

Taking the duration of treatment as a proxy for the duration of chronic Q fever, we set the duration to 12–18 months (CDC, 2013) although there are studies recommending life-long treatment which could vary from one year to a person's entire lifespan (Forland, 2012). However, we assume that symptoms due to the infection resolve during the treatment; if symptoms continue, we consider them not to be associated with the Q fever infection but with underlying conditions.

The most common manifestation of chronic Q fever is heart failure, of which a quarter of cases show conduction disorders (Marrie, 2010); other possible manifestations include vascular and pulmonary infections and chronic hepatitis (Maurin & Raoult, 1999). Therefore disability weights describing heart failure were applied.

The case fatality proportion for chronic Q fever has been estimated to be from to 5 to 50%, according to time of diagnosis and onset of treatment (ECDC, 2010).

Post-infectious fatigue syndrome

Follow-up studies after large outbreaks provide some information regarding duration and the probability of developing post-infectious fatigue syndrome. One large cohort following an outbreak in the UK used standard clinical criteria to quantify the proportion of patients developing fatigue after five years (Ayres JG, 1998) and ten years (Wildman, 2002). The first follow-up reported a larger proportion of idiopathic chronic fatigue (ICF) in Q fever cases (42.3%) than in matched controls (26%), with a difference of 16.3%. At the 10-year follow-up point, cases were matched to controls for the presence of comorbidities and hospital attendance, but there was still a higher proportion of ICF (21.6% vs. 5.4%), with a difference of 16.2%. A recent study from a Dutch outbreak indicates the proportion of patients with fatigue after 12 to 26 months to be higher (43.5%) than after five or ten years of follow-up (Morroy, 2011). Therefore, two health states were specified in order to differentiate short-term fatigue (43.5–16.2/16.3% = 27.2/27.3%) from long-term fatigue (16.2–16.3%). The short-term health state consists of clinical cases that recover within 12 to 26 months; severe cases are assumed to recover after 10 years. Regarding the sources of post-infectious fatigue syndrome (PFS), it is surprising that after 10 years the proportion of PFS is reduced to the same extent in controls as in the cases. We therefore considered the bias to be prevalent and decided to exclude PFS from the model.

Model input summary

Table 1. Transition probabilities and distributions used in the outcome tree

Health outcome	Distribution of health states in health outcome	Transition probability	Source/assumption

(Health state)			
Symptomatic infection<br>(Mild)<br>(Severe)	95–98%<br>2–5%		Maurin & Raoult, 1999; Raoult, 2005
Chronic Q fever		1.6% (1.5–2%)	van der Hoek, 2011; ECDC, 2010
Fatal cases following symptomatic infection		1-2% of severe cases	ECDC, 2010
Fatal cases following chronic infection		5-50%	ECDC, 2010

Table 2. Disability weights and duration

Health outcome (Health state)	Disability Weight (DW) (Haagsma, 2015)		Duration	
	DW	Label	In years	Source
Symptomatic infection<br>(Mild)	0.007 (0.005-0.01)<br>0.125 (0.104-0.152)	Infectious disease, acute episode, mild	0.038<br>0.038	Stouthard, 1997<br>Stouthard, 1997

(Severe)		Infectious disease, acute episode, severe		
Latency period (before chronic Q fever)			0.5 (0.08-1.5)	Fenollar, 2001
Chronic Q fever	0.173 (0.14-0.205)	Heart failure, severe	1-1.5	CDC, 2013

References

Ayres JG, Flint N, Smith EG, Tunnicliffe WS, Fletcher TJ, Hammond K, et al. Post-infection fatigue syndrome following Q fever. QJM: Monthly Journal of the Association of Physicians, 1998 Feb;91(2):105-23.

Dijkstra F, van der Hoek W, Wijers N, Schimmer B, Rietveld A, Wijkmans CJ, et al. The 2007–2010 Q fever epidemic in the Netherlands: Characteristics of notified acute Q fever patients and the association with dairy goat farming. FEMS Immunology and Medical Microbiology. 2012;64(1):3-12 (epub 2011/11/10).

ECDC Technical Report – Risk Assessment on Q fever. May 2010. Available at:

http://www.ecdc.europa.eu/en/publications/Publications/1005_TER_Risk_Assessment_Qfever.pdf

Fenollar F, Fournier PE, Carrieri MP, Habib G, Messana T, Raoult D. Risks factors and prevention of Q fever endocarditis. Clinical Infectious Diseases, 2001 Aug 1;33(3):312-6.

Forland F, De Carvalho Gomes H, Nokleby H, Escriva A, Coulombier D, Giesecke J, et al. Applicability of evidence-based practice in public health: risk assessment on Q fever under an ongoing outbreak. Euro Surveill. 2012;17(3):pii=20060. Available at: http://www.eurosurveillance.org/ViewArticle.aspx?ArticleId=20060

Haagsma JA, Maertens de Noordhout C, Polinder S, Vos T, Havelaar AH, Cassini A, Devleesschauwer B, Kretzschmar ME, Speybroeck N, Salomon JA. Assessing disability weights based on the responses of 30,660 people from four European countries. Population Health Metrics 2015; 13: 10

Karakousis PC, Trucksis M, Dumler SJ: Chronic Q fever in United States. J Clin Microbiol 2006;44: 2283–2287.

Marrie TJ, Raoult D, 2010. Coxiella burnetii (Q Fever). In Mandell, Douglas, and Bennett's Principles and Practice of Infectious Diseases [eds] Gerald L. Mandell, John E. Bennett, Raphael Dolin.—7th edition 2010, Chapter 189, 2511-2519.Maurin M, Raoult D. Q fever. Clinical Microbiology Reviews. 1999;12(4):518-53 (epub 1999/10/09).

Morroy G, Peters JB, van Nieuwenhof M, Bor HH, Hautvast JL, van der Hoek W, et al. The health status of Q-fever patients after long-term follow- up. BMC Infectious Disease, 2011 Apr 18;11:97.

Q Fever Fact Sheet: Symptoms, Diagnosis, and Treatment – CDC, Atlanta. 2013. Available at: http://www.cdc.gov/qfever/symptoms/

Raoult D, Marrie T, Mege J. Natural history and pathophysiology of Q fever. Lancet Infectious Diseases, 2005 Apr;5(4):219-26.

Stouthard ME, Essink-Bot ML, Bonsel GJ, Barendregt JJ, Kramers PGN, van de Water HPA, et al. Disability Weights for Diseases in the Netherlands. Rotterdam, the Netherlands: Department of Public Health, Erasmus University Rotterdam, the Netherlands; 1997.

van der Hoek W, Meekelenkamp JC, Leenders AC, Wijers N, Notermans DW, Hukkelhoven CW. Antibodies against Coxiella burnetii and pregnancy outcome during the 2007-2008 Q fever outbreaks in The Netherlands. BMC Infectious Diseases, 2011 Feb 11;11:44.

Rabies

The initial symptoms of rabies resemble those of other systemic viral infections (Anderson, 1984). Two kinds of central nervous system (CNS) presentation can be seen: the furious form in 70% of all cases and the paralytic form in the remainder (WHO, 2013).

The furious form usually lasts around 12 days on average (range 9�17.8 days) (Udow, 2014). The paralytic form has a longer survival period of 22 days on average (range 18�28 days) and generally results in death.

Case fatality proportion

Once the symptomatic disease onset is confirmed the case fatality proportion is considered to be 100%.

Model input summary

Table 1. Transition probabilities used in the outcome tree

Health outcome<br><br>(Health state)	Distribution of health states<br><br>in health outcome	Transition probability	Source/assumption
Symptomatic infection<br><br>(Furious form)<br><br>(Paralytic form)	 <br>70%<br><br>30%		WHO, 2013
Fatal cases		100%	WHO, 2013

Table 2. Disability weights and duration

Health outcome<br><br>(Health state)	Disability Weight (DW) (Haagsma, 2015)		Duration		
	DW	Label	In years		Source
Symptomatic infection	0.655 (0.579-0.727)	Intensive Care Unit admission			

(Furious form)	As above	As above	0.033 (0.025-0.049)	Udow, 2014
(Paralytic form)	As above	As above	0.060 (0.049-0.077)	Udow, 2014

References

Anderson LJ, Nicholson KG, Tauxe RV, Winkler WG. Human rabies in the United States, 1960 to 1979: Epidemiology, diagnosis, and prevention. Ann Intern Med 1984;100:728-735.

Haagsma JA, Maertens de Noordhout C, Polinder S, Vos T, Havelaar AH, Cassini A, Devleesschauwer B, Kretzschmar ME, Speybroeck N, Salomon JA. Assessing disability weights based on the responses of 30,660 people from four European countries. Population Health Metrics 2015; 13: 10

Udow S, Marrie R, Jackson A. Clinical Features of Dog- and Bat-Acquired Rabies in Humans. Clinical Infectious Diseases Volume 57, Issue 5 Pp. 689-696.

WHO. Rabies. Fact Sheet N�99. Available at: http://www.who.int/mediacentre/factsheets/fs099/en/.

Rubella

Acquired rubella

Acquired, or non-congenital rubella usually gives rise to a mild rash and asymptomatic infections are common. The rash usually begins on the face and then progresses from head to foot. It lasts about three days and is occasionally pruritic (CDC, 2009). Since up to 50% of infections may not present with a rash, many cases are not detected or reported (CDC, 2009; Ang, 2010).

Risk of complications

The most relevant complications associated with rubella virus infection include arthritis or arthralgia, thrombocytopenia, and encephalitis (Zhou, 2004). Additional, but rare complications include orchitis, neuritis, bacterial superinfection, a late syndrome of progressive panencephalitis and mild hepatitis (CDC, 2009).

Arthritis/arthralgia

Arthralgia or arthritis may occur in 30–70% of adult women who contract rubella, but it is rare in children and adult males. It rarely develops into chronic arthritis (CDC, 2009; Mandell, 1999; Johnson, 1958). An age-independent range of 30–70% was estimated as the proportion of acute infections with this complication in the model, for females only. In 11 patients with rubella arthritis studied by Yanez et al. (Yanez, 1966), the onset of arthritis occurred one to six days after the beginning of the exanthem and lasted three to 28 days (mean of nine days).

Thrombocytopenic purpura

Hemorrhagic manifestations occur in approximately one case in 3 000 – more frequently in children than in adults – of which thrombocytopenic purpura is the most common (CDC, 2009; White, 1985; Mandell, 1999; Heggie, 1969; Boyer, 1965). Based on this estimated rate of occurrence (1/3 000), the proportion with the complication was estimated as 0.03% in the model.

Acute throbocytopenic purpura is commonly seen in children aged 1–7 years, and is defined as thrombocytopenia that lasts less than six months. In cases where thrombocytopenia persists for more than six months, it is considered chronic. Chronic thrombocytopenia occurs in a very small number of children (Taghizadeh, 2008).

Encephalitis

Encephalitis occurs in one in 5 000-6 000 cases, more frequently in adults (especially in females) than in children (CDC 2009; Mandell, 1999). Notwithstanding this occurrence rate, an age/sex-independent range of 0.01–0.02% was estimated for the proportion of acute cases with this complication in the model. The severity is highly variable. Symptoms in survivors usually resolve within 1–3 weeks without neurological sequelae (Gülen, 2008; Wolinsky, 1994).

Case fatality proportion

The case fatality proportion for thrombocytopenic purpura is 2.6% (Portielje, 2001). For encephalitis the overall lethality rate is 0–50% (CDC, 2009). Therefore in the model, the case fatality proportion following the health state thrombocytopenic purpura was specified with a point estimate of 4%, and the case fatality proportion following the health state encephalitis was set to the range of 20–50%.

Congenital rubella

Symptomatic infection occurs in 100% of infected foetuses between weeks 1 and 11. During weeks 11–20, symptomatic infection occurs in 30% of foetuses. After week 20 no foetus develops any manifestation of Congenital Rubella Syndrome (CRS) (Feigin, 2004). However, occasional foetal damage (deafness only) has been observed after the twentieth week (Mandell, 1999). Up to 50% of affected foetuses may appear healthy at birth and develop central nervous system abnormalities later (Duszak, 2009). Among children with CRS, 13% have one congenital defect, 24% have two defects and 63% have three or more defects (Reef, 2000).

We did not consider any loss of quality of life before birth and therefore the disability weight and duration for the symptomatic infection was set to 0.

Risk of sequelae

Hearing impairment occurs in 60% of children with CRS, heart disease in 45%, microcephaly in 27% (Reef, 2000), cataracts in 16–25% (Bloom, 2005), mental retardation in 13–25% (Lanzieri, 2004; Reef, 2000), and retinopathy in 5% (Reef, 2000). Overall, 20–40% of CRS survivors aged 35 or older have insulin-dependent diabetes (Mandell, 1999; Duszak, 2009) and 5% of survivors aged 13–19 develop some form of thyroid disease. (Duszak, 2009). Panencephalitis is a rare, fatal, late complication. The incidence of other late complications is still unknown (Duszak, 2009).

The case fatality ratio for infants with confirmed CRS is 10% (Reef, 2000).

Model input summary

Table 1. Transition probabilities used in the outcome tree

Health outcome<br><br>(Health state)	Distribution of health states in health outcome	Transition probability	Source/assumption

Acquired			
Symptomatic infection<br>(Arthritis/arthralgia)<br>(Thrombocytopenic purpura)<br>(Encephalitis) (Uncomplicated)	30–70%; females only<br>0.03%<br>0.01–0.02%<br>Remaining cases		CDC 2009, Mandell 1999, Johnson 1958<br>CDC 2009, White 1985<br>CDC 2009, Mandell 1999
Fatal cases following thrombocytopenic purpura		2.6%	Portielje, 2001
Fatal cases following encephalitis		0–50%	CDC, 2009
Congenital			
Permanent disability due to hearing impairment		60%	Reef, 2000
Permanent disability due to congenital heart defects		45%	Reef, 2000
Permanent disability due to microcephaly		27%	Reef, 2000
Permanent disability due to cataract		16–25%	Bloom, 2005
Permanent disability due to mental retardation		13–25%	Lanzieri, 2004; Reef, 2000
Permanent disability due to retinopathy		5%	Reef, 2000
Permanent disability due to insulin-dependent diabetes		20–40%	Mandell, 1999; Duszak, 2009 (aged >35 years)
Permanent disability due to thyroid gland dysfunction		5%	Duszak, 2009 (aged 13–19 years)
Fatal cases		10%	Reef, 2000

Table 2. Disability weights and duration

Health outcome<br>(Health state)	Disability Weight (DW) (Haagsma, 2015)	Duration

	DW	Label	In years	Source/assumption
Symptomatic infection<br>(Uncomplicated)<br>(Arthritis/arthralgia)<br>(Thrombocytopenic purpura)<br>(Encephalitis)	0.007 (0.005–0.01)<br>0.344 (0.3–0.391)<br>0.167 (0.134–0.201)<br>0.41 (0.358–0.47)	Infectious disease, acute episode, mild<br>Musculoskeletal problems, generalised,moderate<br>Thrombocytopenic purpura<br>Encephalopathy - moderate	0.008<br>0.008–0.077<br>0.008–0.5<br>0.019–0.058	CDC, 2009<br>CDC, 2009<br>Yanez, 1996<br>Taghizadeh, 2008 Gülen, 2008; Wolinsky, 1994/without any neurological sequelae
Congenital				
Symptomatic infection	0		0	
Permanent disability due to hearing impairment	0.008–0.103	From lowest to highest hearing loss related DWs	Remaining life expectancy	
Permanent disability due to congenital heart defects	0.052–0.173	From lowest to highest heart failure related DWs	Remaining life expectancy	
Permanent disability due to microcephaly	0.011–0.421		Remaining life expectancy	

		From lowest to highest cognitive difficulties related DWs		
Permanent disability due to cataract	0.004–0.171	From lowest to highest visual impairment related DWs	Remaining life expectancy	
Permanent disability due to mental retardation	0.011–0.421	From lowest to highest cognitive difficulties related DWs	Remaining life expectancy	
Permanent disability due to retinopathy	0.004–0.171	From lowest to highest visual impairment related DWs	Remaining life expectancy	
Latency period before diabetes	0		35	Mandell, 1999; Duszak, 2009
Latency period before thyroid dysfunction	0		13–19	Duszak, 2009
Permanent disability due to insulin-dependent diabetes	0.07 (0.057–0.088)	Generic uncomplicated disease: worry and daily medication	Remaining life expectancy	
Permanent disability due to thyroid gland dysfunction	0.07 (0.057–0.088)	Generic uncomplicated disease: worry and daily medication.	Remaining life expectancy	

References

Ang LW, Chua LT, James L, Goh KT. Epidemiological surveillance and control of rubella in Singapore, 1991-2007. Ann Acad Med Singapore. 2010 Feb;39(2):95-101.

Assaad F, Ljungars-Esteves K. Rubella-world impact. Rev Infect Dis. 1985 Mar-Apr;7 Suppl 1:S29-36.

Boyer WL, Sherman FE, Michaels RH, et al. Purpura in congenital and acquired rubella. N Engl J Med. 1965;273:1362.

Bloom S, Rguig A, Berraho A, Zniber L, Bouazzaoui N, Zaghloul Z, et al. Congenital rubella syndrome burden in Morocco: a rapid retrospective assessment. Lancet. 2005 Jan 8-14;365(9454):135-41.

Centers for Disease Control and Prevention. Epidemiology and Prevention of Vaccine-Preventable Diseases. Atkinson W, Wolfe S, Hamborsky J, McIntyre L, eds. 11th ed. Washington DC: Public Health Foundation, 2009.

Duszak RS. Congenital rubella syndrome--major review. Optometry. 2009 Jan;80(1):36-43.

Feigin RD. Textbook of pediatric infectious diseases, Volume 1. 2004. Elsevier Health Sciences.

Haagsma JA, Maertens de Noordhout C, Polinder S, Vos T, Havelaar AH, Cassini A, Devleesschauwer B, Kretzschmar ME, Speybroeck N, Salomon JA. Assessing disability weights based on the responses of 30,660 people from four European countries. Population Health Metrics 2015; 13: 10

Heggie AD, Robbins FC. Natural rubella acquired after birth. Am J Dis Child. 1969;118:12.

Gülen F, Cagliyan E, Aydinok Y, Ozen S, Yildiz B. A patient with rubella encephalitis and status epilepticus. Minerva Pediatr. 2008 Feb;60(1):141- 4.

Johnson RE, Hall AP. Rubella arthritis. N Engl J Med. 1958;258:743.

Lanzieri TM, Parise MS, Siqueira MM, Fortaleza BM, Segatto TC, Prevots DR. Incidence, clinical features and estimated costs of congenital rubella syndrome after a large rubella outbreak in Recife, Brazil, 1999-2000. Pediatr Infect Dis J. 2004 Dec;23(12):1116-22

Mandell GL, Bennet JE, Dolin R. Mandell, Douglas and Bennett's Principles and Practice of Infectious Disease - 5th edition. Churchill Livingstone, 1999.

Portielje JE, Westendorp RG, Kluin-Nelemans HC, Brand A. Morbidity and mortality in adults with idiopathic thrombocytopenic purpura. Blood 2001 97:2549–2554.

Reef SE, Plotkin S, Cordero JF, Katz M, Cooper L, Schwartz B, et al. Preparing for elimination of Congenital Rubella Syndrome (CRS): summary of a workshop on CRS elimination in the United States. Clin Infect Dis. 2000 Jul;31(1):85-95

Sherman FE, Michaels RH, Kenny FM. Acute encephalopathy (encephalitis) complicating rubella. JAMA. 1965;192:675.

Steen E, Torp KH. Encephalitis and thrombocytopenic purpura after rubella. Arch Dis Child. 1956;31:470.

Taghizadeh M. An Update on Immune-Mediated Thrombocytopenia. Labmedicine. Volume 39, Number 1, January 2008.

White CC, Koplan JP, Orenstein WA. Benefits, risks and costs of immunization for measles, mumps and rubella. Am J Public Health. 1985 July; 75(7): 739–744.

Wolinsky JS. Rubella. In: Fields BM, Knipe DM, Chanock RM, et al., eds. Virology. 3rd ed. New York: Raven Press, 1994;899-929.

Yanez JE, Thompson GR, Mikkelsen WM, et al. Rubella arthritis. Ann Intern Med 64:772-777, 1966.

Zhou F, Reef S, Massoudi M, Papania MJ, Yusuf HR, Bardenheier B, et al. An Economic Analysis of the Current Universal 2-Dose Measles-Mumps- Rubella Vaccination Program in the United States. J Infect Dis. (2004) 189 Supplement 1: S131-S145.

Salmonellosis

Acute gastroenteritis associated with Salmonella infections in humans is, in most cases, self-limiting within a few days or weeks, but for some patients the disease is fatal. Studies estimated the duration to be 5.58 days for gastroenteritis cases not requiring medical help, 10.65 days for gastroenteritis cases visiting a doctor but not hospitalised and 16.15 days for hospitalised gastroenteritis cases (Kemmeren 2006).

The proportion of mild (uncomplicated), moderate (complicated, doctor) and severe (complicated, doctor) symptomatic infections is set at 83.3%, 15% and 1.7% (Kemmeren 2006; Kwong 2012; redistributing in order to total 100%)

In many reports bacteraemia is highlighted as a possible extra-intestinal complication of salmonellosis (0.03% of laboratory-confirmed cases, Ternhag 2008), although these complicated cases are often considered within the hospitalised proportion of cases (Cressey & Lake 2007; Kemmeren 2006).

The case fatality proportion for symptomatic salmonellosis cases ranged from 0.1% (Kemmeren 2006; Helms 2003) to 0.05% in salmonellosis outbreaks in Austria (Much 2005) and 0.3 for non-typhoid infections in England and Wales (Adak, 2002). These were in line with case fatality proportions observed in cases reported to TESSy between 2009 and 2013 (personal communication).

We chose to estimate the overall case fatality proportion as being within the range 0.05–0.1% and assumed a different age-group distribution of this risk, based on the age-group distribution of deaths reported to TESSy between 2009 and 2013 (see Table 3). This table is based on all TESSy notified cases from EU Member States except Bulgaria, Latvia and Poland which report only aggregate data, and Italy because the outcome was not reported.

Risk of complications

Reactive arthritis (ReA) and Irritable Bowel Syndrome (IBS) are the most frequent sequelae of salmonellosis reported in the literature (Haagsma 2009; Raybourne 2003). The frequency of other post-infectious complications following salmonellosis is extremely low and these were disregarded in the current study.

Reactive arthritis (ReA)

Many studies reported ReA as sequelae of salmonellosis (Keat 1983; Fendler 2001; Raybourne, 2003). A review of the literature, which included mostly cases of salmonellosis occurring during outbreaks, estimated that 8% (2.3–15%) of cases are at risk of developing ReA (Raybourne, 2003), although most of these studies have estimated risk based on laboratory-confirmed cases and duration of diarrhoea is highly correlated with the development of ReA (Yu & Thomson, 1994). In order to account for the considerable uncertainty, the risk of developing ReA from all symptomatic cases is set at 1.31% (0.29–5.43%) (Kemmeren, 2006).

Little is known about the duration of ReA; the average duration is set at between 1.5 months, derived from Hannu et al. (Hannu, 2005) and 222 days (Kemmeren, 2006).

Irritable bowel syndrome (IBS)

In a recent literature review, 8.8% (7.2–10.4%) of symptomatic salmonellosis cases were considered at risk of developing IBS, irrespective of age and gender; the duration was set to five years (Haagsma, 2010). However, the causality is largely debated and the impact of concurrent factors significant. Therefore, IBS is not considered as part of the salmonellosis outcome tree in our study.

Model input summary

Table 1. Transition probabilities and distributions used in the outcome tree

Health outcome (Health state)	Distribution of health states in health outcome	Transition probability	Source/assumption
Symptomatic infection:<br>(Uncomplicated)<br>(Complicated, doctor)<br>(Complicated, hospital)	83.3%<br>15%<br>1.7%		Kemmeren, 2006; Kwong, 2012
Fatal cases following symptomatic infection		0.05–0.1%<br>Age dep. Table 3	Kemmeren, 2006; Much, 2005; TESSy 2009-2013
Reactive arthritis		1.31% (0.29-5.43%)	Kemmeren, 2006

Table 2. Disability weights and duration

Health outcome (Health state)	Disability Weight (DW) (Haagsma, 2015)		Duration
	DW	Label	In years

				Source
Symptomatic infection	0.073 (0.061–0.092)	Diarrhoea, mild	0.015	Kemmeren, 2006
(Uncomplicated)	0.149 (0.12–0.182)	Diarrhoea, moderate	0.029	
(Complicated, doctor)	0.239 (0.202–0.285)	Diarrhoea, severe	0.044	
(Complicated, hospital)				
Reactive arthritis	0.344 (0.3–0.391)	Musculoskeletal problems, generalised, moderate	0.131–0.608	Hannu, 2002

Table 3. Age-group redistribution of CFR (0.05–0.1%)

Age groups	%
0	0.69
1–4	1.72
5–9	1.38
10–14	0.34
15–19	1.03
20–24	0.00
25–29	1.72
30–34	0.34
35–39	1.03
40–44	0.69
45–49	2.07

50–54	3.45
55–59	4.14
60–64	5.17
65–69	9.31
70–74	12.41
75–79	16.55
80–84	18.62
>85	19.31
All ages	100.00

References

Adak GK, Long SM, O'Brien SJ. Trends in indigenous foodborne disease and deaths, England and Wales: 1992 to 2000. Gut. 2002 Dec;51(6):832- 41.

Cressey PJ and Lake R (2007). Risk ranking: Estimates of the burden of foodborne disease for New Zealand. Institute of Environmental Science & Research Limited; Christchurch Science Center.

Fendler CS, Laitko HH, Sorensen C, Gripenberg-Lerche A, Groh J, Uksila K et al. Frequency of triggering bacteria in patients with reactive arthritis and undifferentiated oligoarthritis and the relative importance of the tests used for diagnosis. Ann Rheum Dis 2001;60(4): 337-43.

Food Standards Agency (2000). A Report of the Study of Infectious Intestinal Disease in England. Food Standards Agency London: HMSO.

Haagsma JA, Havelaar AH, Janssen BM, Bonsel GJ. Disability Adjusted Life Years and minimal disease: application of a preference-based relevance criterion to rank enteric pathogens. Popul Health Metr 2008;6:7.

Haagsma JA, van der Zanden BP, Tariq L, van Pelt W, van Duynhoven YTHP, Havelaar AH. Disease burden and costs of selected foodborne pathogens in the Netherlands, 2006. Bilthoven: National Institute of Public Health and Environment. 2009. RIVM rapport 330331001. Available online at: www.rivm.nl/bibliotheek/rapporten/330331001.pdf

Haagsma JA, Siersema PD, De Wit NJ, Havelaar AH. Disease burden of post-infectious irritable bowel syndrome in the Netherlands. Epidemiol Infect 2010;138(11):1650-6.

Haagsma JA, Maertens de Noordhout C, Polinder S, Vos T, Havelaar AH, Cassini A, Devleesschauwer B, Kretzschmar ME, Speybroeck N, Salomon JA. Assessing disability weights based on the responses of 30,660 people from four European countries. Population Health Metrics 2015; 13: 10

Hannu TL, Mattila H, Rautelin P, Pelkonen P, Lahdenne A, Siitonen M et al. Campylobacter-triggered reactive arthritis: a population-based study. Rheumatology 2002;41(3): 312-318.

Havelaar AH, de Wit MAS, van Koningsveld R, van Kempen E (2000a). Health burden in the Netherlands due to infection with thermophilic Campylobacter spp. Epidemiol Infect 2000;125(3): 505-22.

Havelaar AH, de Wit MAS, van Koningsveld R (2000b). Health burden in the Netherlands (1990–1995) due to infection with thermophilic Campylobacter species. Bilthoven, the Netherlands: National Institute for Public Health and the Environment. Report no. 284550004.

Havelaar AH, Ivarsson S, Löfdahl M, Nauta MJ. Estimating the true incidence of campylobacteriosis and salmonellosis in the EU. 2012. [Submitted for publication].

Helms M, Vastrup P, Gerner-Smidt P, Molbak K. Short and long term mortality associated with foodborne bacterial gastrointestinal infections: registry based study. BMJ 2003; 326(7385): 357-60.

Keat A. Reiter's syndrome and reactive arthritis in perspective. N Engl J Med 1983;309(26): 1606-15.

Kemmeren JM, Mangen MJJ, van Duynhoven YTHP, Havelaar AH. Priority setting of foodborne pathogens: disease burden and costs of selected enteric pathogens. Bilthoven: National Institute of Public Health and Environment: RIVM rapport 330080001. 2006. Available online at: www.rivm.nl/bibliotheek/rapporten/330080001.pdf

Mangen MJJ, Havelaar AH, de Wit GA. Campylobacteriosis and sequelae in the Netherlands - Estimating the disease burden and the cost-of- illness. Bilthoven: National Institute for Public Health and the Environment; Report no. 250911004. 2004. Available online at: www.rivm.nl/bibliotheek/rapporten/250911004.pdf

Mangen MJJ, Havelaar AH, Bernsen RAJAM, van Koningsveld R, de Wit GA. The costs of human Campylobacter infections and sequelae in the Netherlands: A DALY and cost-of-illness approach. Acta Agriculturae Scandinavica Section C Food Economics 2005;(1): 35-51.

Melse JM and Kramers PGN (1998). Berekening van de ziektelast in Nederland. Achtergronddocument bij VTV-1997; deel III, hoofdstuk 7. Bilthoven; Report no. 431501028.

Much P, Pichler J, Allerberg F. Food borne infectious outbreaks, Austria 2005." Wien Klin Wochenschr 2007;119 (5-6):150-7.

Raybourne RB, Williams KM, Roberts T and Arthritis Working Group. Food Poisoning: Economic Implications. In: Caballero BL, Trugo P, Finglas (Editors). Encylopaedia of Food Sciences and Nutrition. 10 volume set edition. London: 2003; 2672-82.

Robert Koch Institut. Infektionsepidemiologisches Jahrbuch (various years). Available online at:

www.rki.de/cln_178/nn_205772/DE/Content/Infekt/Jahrbuch/jahrbuch__node.html?__nnn=true

Ternhag A, Törner A, Svensson A, Ekdahl K, Giesecke J. Short- and long-term effects of bacterial gastrointestinal infections. Emerg Infect Dis 2008;14 (1):143-8.

WHO. Global Burden of Disease 2004 Update: Disability weights for diseases and conditions. Geneva: 2004, WHO. Available online at: http://www.who.int/healthinfo/global_burden_disease/GBD2004_DisabilityWeights.pdf

Yu DTY and Thomson GTD. Clinical, epidemiological and pathogenic aspects of reactive arthritis. Food-Microbiology 1994;11(2): 97-108.

Shigellosis

Acute gastroenteritis associated with Shigella spp. infections in humans is, in most cases, self-limiting within days to weeks, but for a few patients the disease may be severe and fatal.

We assume that more complicated cases visit their doctor or are hospitalised and will subsequently be laboratory-tested and reported as confirmed. The proportion of reported cases over the total symptomatic cases is 5.45% (2.18–40%) (Haagsma, 2010).

We assumed a similar duration of symptoms as for salmonellosis: 5.58 days for uncomplicated cases and 10.65–16.15 for complicated ones (Kemmeren, 2006).

On average, patients aged 65 years and over are hospitalised for a greater number of days and are more likely to die of shigellosis than other patients (van Pelt, 2010; Barton Behravesh, 2011). We assumed that only complicated cases lead to fatalities and set the case fatality proportion for complicated cases as 0.06–0.97% (Van Pelt, 2010; Barton Behravesh, 2011). Assuming a different age-group distribution of this risk, we distributed the case fatality proportion based on the age-group distribution of deaths reported to TESSy between 2009 and 2013 (see Table 3). This table is based on all TESSy-notified cases from EU Member States except Bulgaria, Lithuania and Poland, because they report only aggregate data, and Liechtenstein, Luxembourg and Italy which do not report on the death outcome.

Risk of complications

Reactive arthritis (ReA), Post-Infectious Irritable Bowel Syndrome (PI-IBS), Haemolytic Uraemic Syndrome (HUS) and End-stage Renal Disease (ESRD) are possible sequelae of shigellosis.

Asymptomatic cases, which themselves do not have a disease burden for acute illness, might also develop sequelae. However neither the number of asymptomatic cases in the population, nor the percentage of asymptomatic cases that develop sequelae is known and these are therefore not included in the model.

Reactive arthritis (ReA)

The risk of developing ReA has been found to be 6.6% of all laboratory-confirmed cases of shigellosis (Hannu, 2005), 1.2% (Rees, 2004) and 9.8% (Schiellerup, 2008). However, severity of the acute infection and duration of diarrhoea are associated with a higher risk of developing ReA (Townes, 2008; Hannu, 2005; Rees, 2004; Schiellerup, 2008); moreover, these figures relate to laboratory-confirmed cases only. Therefore, we assume that only 'complicated' cases have a risk of 6.6% (1.2-9.8%) of developing ReA.

Little is known about the duration of ReA; the average duration is set between 1.5 months (Hannu, 2005) and 222 days (Kemmeren, 2006).

Irritable bowel syndrome (IBS)

In a recent literature review, 8.8% (7.2–10.4%) of symptomatic infections involving foodborne pathogens (salmonellosis, campylobacteriosis and shigellosis) were associated with a risk of developing IBS, irrespective of age and gender. The duration of IBS was set to five years (Haagsma, 2010). However, the causality is largely debated and the impact of concurrent factors significant. Therefore, IBS is not considered as part of the shigellosis outcome tree in our study.

Haemolytic uraemic syndrome (HUS)

>HUS is characterised by haemolytic anaemia (severe anaemia due to increased destruction of red blood cells), thrombocytopenia (reduced platelet count) and impaired kidney function (acute renal failure). Haemolytic anaemia and thrombocytopenia often occur after bloody diarrhoea. Acute renal failure may then follow.

Several studies have associated HUS with shigellosis infections, in particular Shigella dysenteriae type 1, a species which occurs mainly in tropical countries and accounts for approximately 30% of S. dysenteriae isolates in those countries (Mark Taylor, 2008; Chopra, 1997; Bennis, 2006; Kotloff, 1999; Ekdahl, 2005).

In Europe, based on data reported to TESSy, S. dysenteriae accounts for less than 3% of laboratory-confirmed shigellosis cases, whereas S. sonnei is the most common Shigella species (ECDC, 2013 a & b). This means that around 0.9% of the shigellosis cases occurring in Europe, caused by Shigella dysenteriae type 1, are at risk of developing HUS; however, the risk varies according to EU Member State.

The incidence of S. dysenteriae-induced HUS is unknown and it is affected by antibiotic treatment (Bennish, 2006). HUS caused by S. dysenteriae type 1 is often perceived as more severe than HUS caused by enterohaemorrhagic E. coli (EHEC), however this is probably due to the fact that such infections mainly occur in countries with limited access to high-quality healthcare. Though the age range of Shigella-induced- HUS is wider and the 'median time from the onset of diarrhoea to the presentation of HUS' is longer, HUS caused by Shigella and EHEC is very similar (Mark Taylor, 2008). Therefore, we assume that the risk of developing HUS after symptomatic infection with Shigella dysenteriae type 1 is the same as the risk for symptomatic infections with Shiga-toxin producing E. coli O157 (STEC), around 0.94–1.25% (Cressey & Lake, 2007).

Given that 0.9% of shigellosis cases occurring in Europe are caused by Shigella dysenteriae type 1, the overall risk of developing HUS after symptomatic shigellosis is set to 0.008–0.011%.

HUS occurs mainly in children aged one to five years, and less frequently in children over five years. In one study (Havelaar, 2003) 72% of all HUS cases were under 15 years of age, and 28% were older. The distribution of HUS patients admitted to the Paediatric Nephrology Department of University Hospital Nijmegen from 1974–1993 was used for cases under 15 years (Havelaar, 2003). For the current study we distributed the age risk of developing HUS (0.008-0.011%) according to TESSy-notified cases of HUS by age due to VTEC infection from 2009 to 2013 (see Table 4). Cases were from all EU Member States except Bulgaria and Lithuania, because they report only aggregate data, and Italy because it has sentinel surveillance for STEC/VTEC which does not provide sufficient coverage.

Duration of HUS is reported to be seven days (range 3–31 days) and 41% (19/46) of patients were admitted to hospital (McPherson, 2011). Hospitalisation is reported to last 2–4 weeks for HUS patients (Havelaar, 2003).

The case fatality proportion is assumed to be 3.7% (Cressey & Lake, 2007; Oxford Medical Dictionary, 2003; Havelaar, 2003). This low case- fatality might be valid for cases up to 65 years and be as high as 56% for those aged ≥65 years as data from an outbreak in Scotland suggests (Dundas, 1999). For the current study we use age-specific fatality proportions as reported by Havelaar et al. (Havelaar, 2003; see Table 5).

End-stage renal disease (ESRD)

ESRD is one of the most serious outcomes associated with HUS and is the most advanced stage of kidney failure (Oxford Medical Dictionary, 2003). HUS cases may develop ESRD briefly after HUS or after a long latency period. In the current study we assume that 13.4% develop ESRD, of which 2.9% briefly after HUS and 10.5% after a latency period of 20 years (Havelaar, 2004; Cressey & Lake, 2007). We also assume that all cases experiencing ESRD are in dialysis treatment until transplantation occurs.

The case-fatality proportion is based on the assumption that in the first year after starting dialysis mortality ratios are relatively high and differ between age-groups (see Table 6) and that only few fatalities occur after renal transplantation (Havelaar, 2003). Duration of dialysis (time to transplantation) is age-dependent and is applied to the duration of ESRD, see Table 7 (Havelaar, 2003).

Model input summary

Table 1. Transition probabilities used in the outcome tree

Health outcome<br>(Health state)	Distribution of health states in health outcome	Transition probability	Source/assumption
Symptomatic infection<br>(Uncomplicated)<br>(Complicated)	Rem. cases<br>5.45% (2.18–40%)		Haagsma, 2010
Fatal cases following complicated symptomatic infection		0.06–0.97<br>Age dep.Table 3	Van Pelt, 2010; Barton Behravesh, 2011; TESSy 2009–2013
ReA		6.6% (1.2–9.8%)	Hannu, 2005; Rees, 2004; Townes, 2008; Schiellerup, 2008
HUS		0.008–0.011%<br>Age dep.<br>Table 4	Mark Taylor, 2008; Chopra, 1997; Bennis, 2006; Kotloff, 1999; Ekdahl, 2005; ECDC, 2013 a & b; Cressey & Lake, 2007
Latency period before ESRD		10.5%	Havelaar, 2004; Cressey & Lake, 2007
ESRD after HUS		2.9%	Havelaar, 2004; Cressey and Lake, 2007

ESRD after latency period		100%	
Fatal cases following HUS		< 65 years: 3.7%<br>>=65 years: 56%<br>Table 5	Haavelar, 2004; Dundas, 1999
Fatal cases following ESRD		Age dep. & different for dialysis and transplantation<br>See Table 6.	Havelaar, 2003<br>see Table 6
Transplanted		Remaining %	

Table 2. Disability weights and duration

Health outcome (Health state)	Disability Weight (DW) (Haagsma, 2015)		Duration	
	DW	Label	In years	Source/assumption
Symptomatic infection<br>(Uncomplicated)<br>(Complicated)	0.073–0.149<br>0.239 (0.202–0.285)	Diarrhoea, from mild to moderate<br>Diarrhoea, severe	0.015<br>0.029–0.044	Kemmeren, 2006
ReA	0.344 (0.3–0.391)	Musculoskeletal problems, generalised, moderate	0.131-0.608	Estimated from Hannu, 2005; Kemmeren, 2006
HUS	0.108 (0.09–0.132)	Chronic kidney disease (stage IV)	0.019 (0.008-0.085)	McPherson, 2011
ESRD	0.487 (0.432–0.544)	End-stage renal disease, on dialysis	See Table 7	Assuming that all ESRD are in dialysis

Transplanted	0.070 (0.057–0.088)	Generic uncomplicated disease: worry and daily medication	Remaining life expectancy	Assuming no risk of re-transplantation

Table 3. Age group distribution of the case fatality proportion (0.06–0.97%)

Age groups	%
0	0.00
1–4	10.00
5–9	10.00
10–14	0.00
15–19	0.00
20–24	0.00
25–29	0.00
30–34	10.00
35–39	0.00
40–44	10.00
45–49	20.00
50–54	0.00
55–59	0.00
60–64	10.00
65–69	0.00
70–74	0.00
75–79	10.00
80–84	10.00
>85	10.00
All ages	100.00

Table 4. Age-group redistribution of risk of developing HUS (0.008–0.011%) following infection (TESSy 2009– 2013)

Age groups	%
0	5.67
1–4	33.74
5–9	13.09
10–14	6.62

15–19	2.88
20–24	2.27
25–29	3.83
30–34	3.54

35–39	2.88
40–44	3.40
45–49	3.45
50–54	2.36
55–59	2.88
60–64	3.02
65–69	2.27
70–74	3.36
75–79	1.89
80–84	1.65
85+	0.99
All ages	100

Table 5. HUS case-fatality proportion per age group

Age groups	CFR
0–65	3.7%
>65	56%

Table 6. Case-fatality proportions in the first year after starting dialysis and after renal transplantation

Age class	Case-fatality ratio dialysis	Case-fatality ratio renal transplantation
0–14	4.1% (0.9–11.1%)	7% (2.2–16%)
15–44	8.7% (5.8–12.4%)	7% (2.2–16%)
45–64	37% (31–44%)	7% (2.2–16%)
65–74	65% (58–72%)	7% (2.2–16%)
75+	79% (70–87%)	7% (2.2–16%)

Table 7. Age-specific duration of dialysis

Age class	Duration of dialysis
0–14	1.7 (0.2–5.3)
15–44	2.5 (0.2–9.6)
45–64	6.7 (0.5–30)
>65	5 to remaining life expectancy

References

Barton Behravesh C, Jones TF, Vugia DJ, Long C, Marcus R, Smith K et al. Deaths associated with bacterial pathogens transmitted commonly through food: foodborne diseases active surveillance network (FoodNet), 1996-2005. J Infect Dis, 2011. 204(2):263-7.

Bennish ML, Khan WA, Begum M, Bridges EA, Ahmed S, Saha D, et al. Low risk of hemolytic uremic syndrome after early effective antimicrobial therapy for Shigella dysenteriae type 1 infection in Bangladesh. Clin Infect Dis, 2006. 42(3):356-62.

Chopra, M., D. Wilkinson, and S. Stirling, Epidemic shigella dysentery in children in northern KwaZulu-Natal. S Afr Med J, 1997. 87(1):48-51.

Cressey PJ, Lake R. Risk ranking: Estimates of the burden of foodborne disease for New Zealand, 2007, Institute of Environmental Science & Research Limited; Christchurch Science Center: Christchurch.

Dundas S, et al. Effectiveness of therapeutic plasma exchange in the 1996 Lanarkshire Escherichia coli O157:H7 outbreak. Lancet, 1999. 354(9187):1327-30.

Ekdahl, K. and Y. Andersson, The epidemiology of travel-associated shigellosis--regional risks, seasonality and serogroups. Journal of Infection, 2005. 51(3):222-229.

European Centre for Disease Prevention and Control. Annual Epidemiological Report 2012. Reporting on 2010 surveillance data and 2011 epidemic intelligence data. Stockholm: ECDC; 2013.

European Centre for Disease Prevention and Control. Annual Epidemiological Report 2013. Reporting on 2011 surveillance data and 2012 epidemic intelligence data. Stockholm: ECDC; 2013.

Haagsma JA, Siersema PD, De Wit NJ, Havelaar AH. Disease burden of post-infectious irritable bowel syndrome in the Netherlands. Epidemiol Infect, 2010. 138(11):1650-6.

Haagsma JA, Maertens de Noordhout C, Polinder S, Vos T, Havelaar AH, Cassini A, Devleesschauwer B, Kretzschmar ME, Speybroeck N, Salomon JA. Assessing disability weights based on the responses of 30,660 people from four European countries. Population Health Metrics 2015; 13: 10

Hannu T, Mattila L, Siitonen A, Leirisalo-Repo M. Reactive arthritis attributable to Shigella infection: a clinical and epidemiological nationwide study. Annals of the rheumatic diseases, 2005. 64(4):594.

Havelaar AH, Van Duynhoven YT, Nauta MJ, Bouwknegt M, Heuvelink AE, De Wit GA, et al. Disease burden in the Netherlands due to infections with Shiga toxin-producing Escherichia coli O157. Epidemiol Infect, 2004. 132(3):467-84.

Havelaar AH, Mangen MJJ, van Duynhoven YTHP, Havelaar, AH. Disease burden in the Netherlands due to infections with Shiga toxin-producing Escherichia coli O157., in RIVM report 284550008/20032003, National Institute of Public Health and the Environment: Bilthoven, 2003.

Kemmeren J, Mangen MJJ, van Duynhoven YTHP, Havelaar AH. Priority setting of foodborne pathogens: disease burden and costs of selected enteric pathogens. RIVM report. National Institute of Public Health and the Environment: Bilthoven, 2006.

2006. Available at: http://www.rivm.nl/bibliotheek/rapporten/330080001.pdf.

Mark Taylor C. Enterohaemorrhagic Escherichia coli and Shigella dysenteriae type 1-induced haemolytic uraemic syndrome. Pediatric Nephrology, 2008. 23(9):1425-1431.

McPherson M, et al. Economic costs of Shiga toxin-producing Escherichia coli infection in Australia. Foodborne Pathogen Diseases, 2011. 8(1):55-62.

Rees JR, et al., Persistent diarrhea, arthritis, and other complications of enteric infections: a pilot survey based on California FoodNet surveillance, 1998-1999. Clin Infect Dis, 2004. 38 Suppl 3: S311-7.

Schiellerup P, Krogfelt KA, Locht H. A comparison of self-reported joint symptoms following infection with different enteric pathogens: effect of HLA-B27. J Rheumatol, 2008. 35(3): p. 480-7.

Tariq L, Haagsma J, Havelaar A. Cost of Illness and Disease Burden in The Netherlands Due to Infections with Shiga ToxinProducing Escherichia coli O157. Journal of Food Protection&# 174;, 2011. 74(4):545-552.

Townes JM, et al. Reactive arthritis following culture-confirmed infections with bacterial enteric pathogens in Minnesota and Oregon: a population-based study. Ann Rheum Dis, 2008. 67(12): 1689-96.

Van Pelt W, et al. Trends in Gastro-enteritis in Nederland; notitie met betrekking tot 2009 en 2010, in RIVM report project V/210221 December 2010, RIVM: Bilthoven.

STEC/VTEC

The current disease model relies strongly on publications focused around STEC/VTEC O157 infections. Shiga toxin-producing Escherichia coli O157 (STEC/VTEC O157) infection may be asymptomatic, or may result in acute gastroenteritis (GE), and potentially in haemorrhagic colitis: 44.5% of cases had bloody diarrhoea (Michel, 2000). Duration is assumed to be longer than for non-bloody diarrhoea (Havelaar, 2004): median duration of five days and three days for bloody and non-bloody diarrhoea respectively (Cressey & Lake, 2007), which are proposed in the model as a uniform distribution.

There is little information on STEC/VTEC-associated mortality. Study findings range from 0.083% of the total estimated/VTEC O157:H7 (Mead, 1999), 0.03% (Buzby & Roberts, 2009), 0.04% (Walkerton outbreak, one fatal case in 2 321 patients, Bruce-Grey-Owen Sound Health Unit, 2000) and 0.045 (Havelaar, 2004). We therefore assume a uniformly distributed case-fatality proportion of between 0.03% and 0.045% for this study.

Fatal cases occur mainly in elderly people (Bauch, 2007); therefore, we assumed that the case fatality proportion of 0.03–0.045% is distributed across age-groups in accordance with the observed age-group distribution of TESSy-reported deaths between 2009 and 2013 (see Table 3). This table is based on all TESSy-notified cases from EU Member States except Bulgaria and Lithuania, because they report only aggregate data, and Italy because it has sentinel surveillance for STEC/VTEC for which we do not have the coverage.

Risk of complications

STEC/VTEC infection has been associated with post-diarrhoeal haemolytic uremic syndrome (HUS), which may result in death, end-stage renal disease (ESRD) or other sequelae. HUS and ESRD are the most frequently occurring sequelae of STEC and will be considered in the outcome tree. Irritable Bowel Syndrome (IBS) is another frequently occurring sequelae of bacteria-triggered gastroenteritis (Haagsma, 2010; Marshall, 2010; Thabane, 2009) and was considered for inclusion in the outcome tree (see below). The frequency of other post-infectious complications following STEC is low and they were therefore disregarded (Havelaar, 2004; Frenzen, 2005; Cressey & Lake, 2007; Buzby, 2009; McPherson, 2011; Tariq, 2011).

Haemolytic uraemic syndrome (HUS)

Haemolytic Uraemic Syndrome (HUS) is ‘a condition in which sudden rapid destruction of red blood cells causes acute renal failure’ (Oxford Medical Dictionary, 2003). HUS may occur following a respiratory or gastrointestinal infection, especially by pathogenic Escherichia coli or

Shigella spp.

The risk of developing HUS after STEC/VTEC infection has been found to be 3–7% (McPherson, 2011), 1% (Havelaar, 2004), 0.94–1.25% (Cressey & Lake, 2007) and 1.6% of laboratory-confirmed EHEC infections although authors mention under-estimation due to misclassification (13/820; Ternhag, 2008). In the current study we assume that the probability of developing HUS after a VTEC/STEC symptomatic infection is 0.94–1.25%.

HUS occurs mainly in children between the ages of one and five years, and less frequently in children over five years. In one study, 72% of all HUS cases were under 15 years of age and 28% were older (Havelaar, 2003). Member States report HUS outcomes relating to STEC/VTEC infections and we therefore redistributed the age-group risk of developing HUS (0.94–1.24%) based on the age-group of HUS cases reported to TESSy between 2009 and 2013 (all Member States except Bulgaria, Italy and Lithuania) (see Table 4).

Duration is reported to be seven days (range 3–31 days) and 41% (19/46) of patients were admitted to hospital (McPherson, 2011); hospitalisation is reported to last two to four weeks for HUS patients (Havelaar, 2003).

The case fatality proportion was found to be 3.7% (Cressey & Lake, 2007; Oxford Medical Dictionary, 2003; Havelaar, 2003). This low case- fatality may be valid for cases up to 65 years and then as high as 56% for cases ≥65 years, as indicated by data from an outbreak in Scotland (Dundas, 1999). Other studies assume age-specific fatality rates, as reported by Havelaar et al. (Havelaar, 2003). We estimated the age-group case fatality proportion from HUS based on STEC/VTEC infections notified to TESSy between 2009 and 2013 from all Member States, except Bulgaria, Italy and Lithuania (see Table 5).

End-stage renal disease (ESRD)

ESRD is one of the most serious outcomes associated with HUS and is the most advanced stage of kidney failure (Oxford Medical Dictionary, 2003). HUS cases may develop ESRD briefly after HUS or after a long latency period. In the current study we assume that 13.4% develop ESRD, 2.9% of whom develop it briefly after HUS and 10.5% after a latency period of 20 years (Havelaar, 2004; Cressey & Lake, 2007). We also assume that all cases experiencing ESRD undergo dialysis treatment until transplantation occurs.

The case-fatality proportion is based on the assumption that in the first year after starting dialysis mortality is relatively high and differs among age-groups (see Table 6) and that only few fatalities occur after renal transplantation (Havelaar, 2003). Duration of dialysis (time to transplantation) is age-dependent and is applied to the duration of ESRD – see Table 7 (Havelaar, 2003).

Irritable bowel syndrome (IBS)

In a recent literature review, 8.8% (7.2-10.4%) of symptomatic infections with foodborne pathogens were considered at risk of developing IBS, irrespective of age and gender; the duration was set to 5 years (Haagsma, 2010). However, the causality is largely debated and the impact of concurrent factors significant. Therefore, IBS is not considered as part of the STEC/VTEC outcome tree in our study.

Model input summary

Table 1. Transition probabilities and distributions used in the outcome tree

Health outcome (Health state)	Distribution of health states in health outcome	Transition probability	Source/assumption
Fatal cases following symptomatic infection		0.03-0.045% Age-dependent (Table 3)	Buzby & Roberts, 2009; TESSy 2009-2013
Haemolytic uraemic syndrome (HUS)		0.94-1.25% Age-dependent (Table 4)	Havelaar, 2004; Cressey and Lake, 2007; TESSy 2009-2013
Latency period before ESRD		10.5%	Havelaar, 2004; Cressey and Lake, 2007
ESRD after HUS		2.9%	Havelaar, 2004; Cressey and Lake, 2007
ESRD after latency period		100%	
Fatal cases following HUS		Age-dependent (Table 5)	TESSy 2009-2013
Fatal cases following ESRD		Age-dependent, different for dialysis and transplantation (Table 6)	Havelaar, 2003 see Table 6
Transplanted		Remaining %	

Table 2. Disability weights and duration

Health outcome (Health state)	Disability Weight (DW) (Haagsma, 2015)		Duration	
	DW	Label	In years	Source
Symptomatic infection (Gastroenteritis)	0.149 (0.12-0.182)	Diarrhoea, moderate	0.008-0.014	Havelaar, 2004; Cressey & Lake, 2007
HUS	0.108 (0.09–0.132)	Chronic kidney disease (stage IV)	0.019 (0.008–0.085)	McPherson, 2011
ESRD	0.487 (0.432–0.544)	End-stage renal disease, on dialysis	Age dependent(See Table 7)	Assuming that all ESRD are in dialysis
Transplanted	0.070 (0.057–0.088)	Generic uncomplicated disease: worry and daily medication	Remaining life expectancy	

Table 3. Age-group redistribution of case fatality proportion (0.03–0.045%)

Age groups	%
0	4.30
1-4	9.68
5-9	4.30
10-14	0.00
15-19	0.00
20-24	2.15
25-29	0.00
30-34	0.00
35-39	3.23
40-44	3.23
45-49	2.15
50-54	1.08
55-59	4.30

60-64	8.60
65-69	4.30
70-74	10.75
75-79	10.75
80-84	15.05
>85	16.13
All ages	100.00

Table 4. Age-group redistribution of risk of developing haemolytic uraemic syndrome (0.94–1.25%)

Age	%
0	5.67
1-4	33.74
5-9	13.09
10-14	6.62
15-19	2.88
20-24	2.27
25-29	3.83
30-34	3.54
35-39	2.88
40-44	3.40
45-49	3.45
50-54	2.36
55-59	2.88
60-64	3.02
65-69	2.27
70-74	3.36
75-79	1.89
80-84	1.65
85+	0.99
All ages	100

Table 5. Age-group case fatality proportion from haemolytic uraemic syndrome (TESSy 2009–2013)

Age	%

0	6.06
1-4	2.63
5-9	3.25
10-14	0.00

15-19	0.00
20-24	5.13
25-29	0.00
30-34	0.00
35-39	3.64
40-44	3.28
45-49	3.17
50-54	2.13
55-59	2.00
60-64	4.44
65-69	8.33
70-74	4.62
75-79	17.86
80-84	25.93
85+	28.57
All ages	3.91

Table 6. Case-fatality proportions in the first year after starting dialysis and after renal transplantation

Age class	Case-fatality ratio dialysis	Case-fatality ratio renal transplantation
0–14	4.1% (0.9–11.1%)	7% (2.2–16%)
15–44	8.7% (5.8–12.4%)	7% (2.2–16%)
45–64	37% (31–44%)	7% (2.2–16%)
65–74	65% (58–72%)	7% (2.2–16%)
75+	79% (70–87%)	7% (2.2–16%)

Table 7 .Age specific duration of dialysis

Age class	Duration of dialysis
0-14	1.7 (0.2-5.3)
15-44	2.5 (0.2-9.6)
45-64	6.7 (0.5-30)

>65	5 years to remaining life expectancy

References

Bauch CT, Anonychuk AM, Pham BZ, Gilca V, Duval B, Krahn MD (2007). Cost-utility of universal hepatitis A vaccination in Canada. Vaccine 25(51): 8536-48.

Buzby JC, Roberts T (2009). The economics of enteric infections: human foodborne disease costs. Gastroenterology 136: 1851-1862.

Bruce-Grey-Owen Sound Health Unit (2000). The investigative report of the Walkerton outbreak of waterborne gastro-enteritis. Owen Sound, Ont. Canada: Bruce-Grey-Owen Sound Health Unit. Cressey PJ, Lake R (2007). Risk ranking: Estimates of the burden of foodborne disease for New Zealand. Institute of Environmental Science & Research Limited; Christchurch Science Center.

Dundas S, Murphy J, Soutar RL, Jones GA, Hutchinson SJ, Todd WT. Effectiveness of therapeutic plasma exchange in the 1996 Lanarkshire Escherichia coli O157:H7 outbreak. Lancet. 1999;354: 1327–30

Frenzen PD, Drake A, Angulo FJ (2005). Economic cost of illness due to Escherichia coli O157 infections in the United States. J Food Prot 68 (12): 2623-30.

Haagsma J.A., P.D. Siersema, N.J. De Wit, A.H. Havelaar (2010). Disease burden of post-infectious irritable bowel syndrome in the Netherlands. Epidemiol Infect 138(11): 1650-6.

Haagsma JA, Maertens de Noordhout C, Polinder S, Vos T, Havelaar AH, Cassini A, Devleesschauwer B, Kretzschmar ME, Speybroeck N, Salomon JA. Assessing disability weights based on the responses of 30,660 people from four European countries. Population Health Metrics 2015; 13: 10

Havelaar AH, van Duynhoven YT, Nauta MJ, Bouwknegt M, Heuvelink AE, de Wit GA, et al. (2003) Disease burden in The Netherlands due to infections with Shiga toxin-producing Escherichia coli O157. Epidemiol Infect 132(3): 467-84.

Marshall JK, Thabane M, Garg AX, Clark WF, Moayyedi P, Collins SM. Walkerton Health Study Investigators (2010). Eight year prognosis of post- infectious irritable bowel syndrome following waterborne bacterial dysentery. Gut;59: 605-611.

McPherson M, Kirk MD, Raupach J, Combs B, Butler JR. (2011) Economic costs of Shiga toxin-producing Escherichia coli infection in Australia. Foodborne Pathog Dis 8 (1): 55-62.

Mead PS, Slutsker L, Dietz V, McCaig LF, Bresee JS, Shapiro C et al. (1999). Food-related illness and death in the United States. Emerg Infect Dis 5(5): 607-625.

Michel P, Wilson JB, Martin SW, Clarke RC, McEwen SA, Gyles CL (2000). Estimation of the under-reporting rate for surveillance of Escherichia coli O157:H7 cases in Ontario, Canada. Epidemiol Infect 125: 35-45.

Oxford Medical Dictionary (2003). Oxford, Oxford University Press; 3rd edition.

Tariq L, Haagsma J, Havelaar A (2011). Cost of Illness and Disease Burden in The Netherlands Due to Infections with Shiga Toxin-Producing Escherichia coli O157. J Food Prot 74 (4): 545-52.

Ternhag A, Törner A, Svensson A, Ekdahl K, Giesecke J (2008). Short- and long-term effects of bacterial gastrointestinal infections. Emerg Infect Dis 14 (1): 143-8.

Thabane M, Simunovic M, Akhtar-Danesh N, Marshall JK (2009). Development and Validation of a Risk Score for Post-Infectious Irritable Bowel Syndrome. Am J Gastroenterol; 104:2267–2274.

Syphilis

Syphilis is a complex, systemic disease caused by the spirochaete Treponema pallidum (T. pallidum), a gram-negative bacterium. Syphilis is preventable and curable with effective and inexpensive antibiotics. The only known natural hosts are humans, and the pathogen is not able to survive outside its host due to limited metabolic capacities to synthetise its own bio-nutrients. Syphilis spirochetes, like other treponemas, cannot be cultivated in vitro. The primary mode of syphilis transmission is by sexual contact (acquired syphilis). Vertical transmission from infected mother to child is possible (congenital syphilis), either in utero (transfer across the placenta) or through contact with an active genital lesion during delivery (Singh, 1999). Untreated syphilis can adversely affect pregnancy outcomes, resulting in spontaneous abortion, stillbirth, premature delivery, or perinatal death. Prematurity and low birth weight have been observed in 10 to 40% of infants born to untreated mothers (Salojee, 2004). The rate of infection through sexual intercourse with an infected partner has been estimated at about 50% (Ficarra & Carlos, 2009).

In Europe and other high-income countries, the transmission via blood or blood products is rare because of the low incidence rates of the disease and improved blood screening and blood donor testing for syphilis (Tramont, 2005).

Only 50% of those infected with T. pallidum will develop symptoms (RKI, 2003). Primary syphilis lasts from two weeks to six months (Baughn & Musher, 2005). Secondary syphilis may last two to eight weeks (Zetola, 2007). Early latent disease is diagnosed less than one year after infection (WHO, 2003; MMWR, 2010). Late latent syphilis infection is diagnosed after more than one year (WHO, 2003; MMWR, 2010).

Health outcomes and states associated with syphilis infection in adults

The incubation period for primary syphilis is on average three weeks (10–90 days) and depends on bacterial load, the immune status of the infected person and the existence of other co-morbid conditions (e.g. HIV/AIDS) (Weir & Fisman, 2002; Krause, 2006). Acquired syphilis is divided into primary, secondary, latent and tertiary syphilis. The disease can also be divided into early and late syphilis. Early syphilis implies the primary, secondary and early latent stages. Late syphilis refers to late latent syphilis and tertiary syphilis (Hook, 1992).

Primary syphilis is characterised by an ulcer and/or chancre at the site of infection or inoculation. This primary lesion appears about three weeks after exposure as an indurated, painless ulcer and may not be clinically evident (i.e. it may be in the rectum or the cervix). Invasion of the bloodstream precedes the initial lesion. In 50% of cases, the chancre is accompanied by regional lymphadenopathy (a firm, non-tender satellite lymph node) (Genc, 2000). After three to six weeks the chancre begins to involute, but may persist in the secondary stage in 15–30% of those infected (Zetola, 2007; Krause, 2006; Parish, 2000).

After 2–12 weeks on average (sometimes 12 months) the untreated infection may progress to secondary syphilis caused by the haematogenic spread and lymphatic dissemination of T. pallidum in the body. The time at which the secondary lesions manifest depends on the bacterial load of the treponeme and the immune response of the host (Baughn, 2005). This stage is characterised by skin rash, condylomata lata (5–22% of patients), mucocutaneous lesions, alopecia (5–7% of patients), and generalised lymphadenopathy (Ficarra & Carlos, 2009). A patient with secondary syphilis may have one, several or all of the signs of the secondary stage. Since each of the signs may also be associated with other diseases, none are specific to syphilis. Neurological involvement in secondary syphilis (known as syphilitic meningitis) can occur, especially in HIV co-infected patients (Marra, 2004). The manifestations of secondary syphilis last two to eight weeks and then may resolve, even without treatment (Zetola, 2007).

After resolution of the secondary manifestations, around one-third of untreated patients will enter into a latent phase. The latent or asymptomatic stage of syphilis is defined as the period from disappearance of the secondary manifestations until therapeutic cure or development of late sequelae. An infection without any clinical symptoms lasting less than one year is referred to as early latent syphilis, whereas an infection of more than one year's duration without clinical evidence of treponemal infection is referred to as late latent syphilis (WHO, 2003). The definitions of duration may vary across countries. The early latent period corresponds to the highest risk of transmission.

Tertiary syphilis may appear after a long period of untreated syphilis (5–20 years after initial infection) and its manifestations can include gummas (late benign syphilis), cardiovascular symptoms and neurosyphilis (Hutto, 2001). In developed countries gummas and cardiovascular symptoms are rarely seen and most of the late sequelae are associated with neuro-syphilis. The timescale for development of neuro-syphilis may vary from a period of one or two years to more than 30 years after primary syphilis, and may involve 5–10% of untreated patients (Gjestland, 1955). It is characterised by the involvement of the central nervous system which leads to a number of different syndromes, included in the health outcome 'neuro-syphilis' in our model. In two thirds of patients the infection will not progress to late complications (Mindel, 2000).

Health outcomes and states associated with congenital syphilis infection

Postnatal manifestations of congenital syphilis are divided into early and late stages. Clinical manifestations occurring within the first two years after birth (<2 years) are categorised as early congenital syphilis. Clinical manifestations which occur later than two years after birth are late congenital syphilis (Parish, 2000). For the underlying model, and due to scarce data, only congenital syphilis was included, with no distinction between early or late.

Outcome tree parameters

Due to the high complexity of syphilis outcomes and for reasons of feasibility, the outcome tree for the adult population was split into symptomatic and asymptomatic infections at the first level of disaggregation. The natural course of syphilis was subdivided into the three main disease states: primary, secondary and neuro-syphilis. The focus was on neuro-syphilis because other forms of late syphilis sequelae are very rare in developed countries.

The percentage of asymptomatic cases was estimated at 50% (RKI 2003, Singh, 1999; Ficarra, 2009; Genc, 2000; Parish, 2000). Gerbase and colleagues presented treatment rates of 85% for both primary and secondary symptomatic syphilis cases in regions with established market economies (Gerbase, 2000). As a result of high cure rates (up to 100%), it was estimated that about 85% of all primary syphilis cases are treated and subsequently cured. The remaining 15% of untreated symptomatic cases have a 30–50% possibility of developing secondary syphilis, resulting in a probability of 4.5–7.5% that they will develop secondary syphilis, after having had primary syphilis (Singh, 1999; Weir & Fisman, 2002; Krause, 2006; Gerbase, 2000; Golden, 2003). In asymptomatic primary syphilis the primary chancre is not visible and will generally go unnoticed, meaning that it is less likely to be treated, hence the greater risk of progression to secondary syphilis (30–50%).

Furthermore, 85% of symptomatic secondary syphilis cases are treated and again, as a result of the high cure rates (around 100%), the remaining 15% of untreated cases have a probability of 5–12% of developing neuro-syphilis. Thus, the proportion of people developing neuro- syphilis from preceding secondary syphilis was set at 0.75–1.88% (Tramont, 2005; Zetola, 2007; Gerbase, 2000; Goldmeier & Guallar, 2003).

The probability of dying due to syphilis before reaching the late (tertiary) phase of the disease is very low and there is little evidence of a case fatality ratio associated with syphilis in general, or neurosyphilis in particular, within Europe. We assumed that neurosyphilis in Europe is successfully treated; although with a possibility of developing permanent disabilities for which it was impossible to define the impact due to lack of data. Antibiotic treatment is highly effective and is therefore not associated with a case fatality ratio.

For infants the main outcome is congenital infection with a probability of 20% (2–64%) for an infected child (Singh, 1999; Salojee, 2004; Genc, 2000; Gerbase, 2000). In total, 1% of all children with congenital infection die (Gerbase, 2000).

Model input summary

Table 1. Transition probabilities and distributions used in the outcome tree

Health outcome (Health state)	Transition probability	Source/assumption
Acquired		
Primary syphilis from infection	50%	RKI, 2003
Secondary syphilis from asymptomatic infection	30–50%	Singh, 1999; Weir & Fisman, 2002; Gerbase, 2000; Golden 2003
Secondary syphilis from symptomatic infection	4.5–7.5%	Singh, 1999; Weir & Fisman, 2002; Gerbase, 2000; Golden 2003
Neuro-syphilis	0.75–1.88%	Tramont, 2005; Zetola, 2007; Krause, 2006; Weir&Fisman, 2002; Gerbase, 2000; Golden, 2003; Goldmeier, 2003
Fatal cases due to neurosyphilis	0%	Assuming all cases are identified and treated, and no treatment failure
Congenital		
Symptomatic infection	20% (2–64%)	Singh, 1999; Saloojee, 2004; Genc & Ledger, 2000

Fatal cases due to congenital infection	1%	Genc & Ledger, 2000

Table 2. Disability weights and duration

Health outcome (Health state)	Disability Weight (DW) (Haagsma, 2015)		Duration	
	DW	Label	In years	Source
Acquired				
Primary syphilis	0.007 (0.005-0.01)	Infectious disease, acute episode, mild	0.121-0.5	Baughn & Musher, 2005
Latency period (from primary to secondary)	0		0.23 (0.038-1)	Baughn, 2005
Secondary syphilis	0.125 (0.104-0.152)	Infectious disease, acute episode, severe	0.038-0.153	Zetola, 2007
Latency period (from secondary to neurosyphilis)	0		4.77-19.77	Hutto, 2001
Neurosyphilis	0.407 (0.36-0.46)	Motor plus cognitive impairments, severe	0.027-0.038	Workowski, 2010 Assuming 10–14 days of treatment
Congenital				
Symptomatic infection	0.125 (0.104-0.152)	Infectious disease, acute episode, severe	3	Kwong, 2010

References

Baughn RE, Musher DM. Secondary syphilitic lesions. Clin Microbiol Rev 2005, 18:205-216.

Bremer, V, Marcus U, Hamouda O (2012). Syphilis on the rise again in Germany--results from surveillance data for 2011. Euro Surveill 17(29).

[SW1] Doherty L, Fenton KA, Jones J, Paine TC, Higgins SP, Williams D, et al. Syphilis: old problem, new strategy. BMJ 2002, 325:153-156.

Ficarra G, Carlos R. Syphilis: the renaissance of an old disease with oral implications. Head Neck Pathol 2009, 3:195-206.

Genc M, Ledger WJ. Syphilis in pregnancy. Sex Transm Infect 2000, 76:73-79.

Gerbase A, Stein C, Levison J, Htun Y. Global burden of sexually transmitted diseases (excluding HIV) in the year 2000. In: Global burden of sexually transmitted diseases (excluding HIV) in the year 2000. Geneva; World Health Organization; 2000.

Gjestland T. The Oslo study of untreated syphilis; an epidemiologic investigation of the natural course of the syphilitic infection based upon a re-study of the Boeck-Bruusgaard material. Acta Derm Venereol Suppl (Stockh) 1955, 35:3-368; Annex I-LVI.

Golden MR, Marra CM, Holmes KK. Update on syphilis: resurgence of an old problem. JAMA 2003, 290:1510-1514.

Goldmeier D, Guallar C. Syphilis: an update. Clin Med 2003, 3:209-211.

Haagsma JA, Maertens de Noordhout C, Polinder S, Vos T, Havelaar AH, Cassini A, Devleesschauwer B, Kretzschmar ME, Speybroeck N, Salomon JA. Assessing disability weights based on the responses of 30,660 people from four European countries. Population Health Metrics 2015; 13: 10

Hook EW, 3rd, Marra CM. Acquired syphilis in adults. N Engl J Med 1992, 326:1060-1069.

Hutto B. Syphilis in clinical psychiatry: a review. Psychosomatics 2001, 42:453-460.

Krause W. [Syphilis]. Urologe A 2006, 45:1494-1500.

Kwong JC, Crowcroft NS, Campitelli MA, Ratanasingham S, Daneman N, Deeks SL, et al. Ontario Burden of Infectious Disease Study. OAHPP/ECES Report. City: Ontario Agency for Health Protection and Promotion and the Institute for Clinical Evaluative Sciences; 2010.

Limb M. New scheme aims to eradicate congenital syphilis in three years. BMJ 2012, 344:e1616.

Marra C. Neurosyphilis. Curr Neurol Neurosci Rep 2004, 4:435-440.

Mindel A, Estcourt C. Syphilis. In: Sexually transmitted diseases: Vaccines, Prevention and Control. Edited by Stanberry LR, Bernstein DI: Academic Press, San Diego, San Francisco, New York, Boston, London, Sydney, Tokyo; 2000

Murray CJL, Lopez AD, World Health Organization, World Bank, Harvard School of Public Health. Global health statistics: a compendium of incidence, prevalence and mortality estimates for over 200 conditions. Geneva: World Health Organization; 1996.

Murray CJL, Lopez AD. The Global Burden of Disease - A comprehensive assessment of mortality and disability from diseases, injuries, and risk factors in 1990 and projected to 2020. Cambridge: Harvard School of Public Health on behalf of the World Health Organization and the World Bank; 1996.

Parish JL. Treponemal infections in the pediatric population. Clin Dermatol 2000, 18:687-700.

Robert Koch Institut. RKI-Ratgeber Infektionskrankheiten - Merkblätter für Ärzte: Syphilis (Lues). Epidemiologisches Bulletin 2003, 30:229-233.

Saloojee H, Velaphi S, Goga Y, Afadapa N, Steen R, Lincetto O. The prevention and management of congenital syphilis: an overview and recommendations. Bull World Health Organ 2004, 82:424-430.

Singh AE, Romanowski B. Syphilis: review with emphasis on clinical, epidemiologic, and some biologic features. Clin Microbiol Rev 1999, 12:187- 209.

Tramont EC. Treponema pallidum (Syphilis). In: Principles and practice of infectious diseases. Volume Volume 2, 6th Edition. Edited by Mandel GL, Bennet JE, Dolin R. Pennsylvania: Elsevier Inc. 2005: 2768-2785

Weir E, Fisman D. Syphilis: have we dropped the ball? CMAJ 2002, 167:1267-1268.

Workowski KA, Berman SM; Centers for Disease Control and Prevention. Sexually transmitted diseases treatment guidelines, 2010. MMWR Recomm Rep. 2010;59(RR-12):1-110.

World Health Organization. Dept. of Reproductive Health and Research. Guidelines for the management of sexually transmitted infections. Geneva: World Health Organization; 2003.

Zetola NM, Engelman J, Jensen TP, Klausner JD. Syphilis in the United States: an update for clinicians with an emphasis on HIV coinfection. Mayo Clin Proc 2007, 82:1091-1102.

Tetanus

Tetanus is an acute and often fatal disease induced by the tetanospasmin, an exotoxin produced by Clostridium tetani, a gram-positive anaerobic bacillus (Bleck, 2005; CDC, 2012). C. tetani is sensitive to heat and not viable under aerobic conditions (CDC, 2012). In contrast, the spores of C. tetani are resistant to heat and antiseptics and are widely present in soil and in the intestines and faeces of animals (e.g. horses, sheep and dogs). Tetanus is primarily contracted via contaminated wounds and is not contagious. Effective vaccination programmes significantly reduced the burden of tetanus. Globally around 800 000 to 1 000 000 people die of tetanus each year (Dietz, 1996). Around 90% of all deaths occur in developing countries which are largely affected by tetanus and especially neonatal and maternal tetanus. In developed countries, high-risk groups, such as unvaccinated persons and injecting drug users, are prone to infection with C. tetani (CDC, 2012). The proportion of asymptomatic/subclinical infections is unknown but it can be assumed that cases of tetanus are symptomatic in nearly 100% of those infected. The first symptoms of tetanus appear after an average incubation period of eight days (range: 3–21 days) (CDC, 2012). The duration of the symptomatic disease for generalised, localised and cephalic tetanus is two to three weeks (CDC, 2012).

Health outcomes/states associated with tetanus infection

The clinical features of acute tetanus infections can be subdivided into three health states that are observed in developed countries. A fourth type, tetanus neonatorum is a specific form of generalised tetanus that affects neonates and is mostly observed in the developing world with a high case fatality of up to 90% (Roper, 2007). As neonatal tetanus has been eliminated in Europe this health outcome is not considered in our outcome tree and model.

The distribution of the three health states is set according to the observed risk of developing the different forms of acute infection in USA (Bardenheier, 1998): 81% were generalised; 13% localised and 6% cephalic.

Localised tetanus

Localised tetanus is an uncommon health state of tetanus. Localised tetanus appears as a persistent contraction of muscles in the injured area, commonly preceding generalised tetanus, and lasts around two to three 3 weeks (CDC, 2012).

Generalised tetanus

The most common health state of tetanus infection is generalised tetanus. The probability of developing generalised tetanus after initial infection is around 80% (CDC, 2012; Bardenheier, 1998; Guilfoile, 2008). The symptoms of generalised tetanus are trismus or lockjaw in the early stages, developing into stiffness of the neck, difficulty in swallowing and rigidity of abdominal muscles. Further, unspecific symptoms such as elevated temperature, sweating, elevated blood pressure, and episodic rapid heart rate may occur. Generalised tetanus can last for 3-4 weeks and full recovery may take several months (CDC, 2012).

Cephalic tetanus

Cephalic tetanus is another uncommon health state involving the cranial nerves. The same duration has been assumed for this health state as for localised tetanus: 2–3 weeks.

Further complications and case fatality proportion

In cases of cephalic tetanus otitis media may occur (CDC, 2012). Long-term sequelae/disabilities from tetanus are not reported in the literature.

The overall mortality rate of tetanus ranges from 28/100 000 in developing countries to 0.1/100 000 in developed countries such as the USA. The case fatality proportion ranges between 5 and 55% (Guilfoile, 2008; Brook, 2004; Cook, 2001; Farrar, 2000; Kanchanapongkul, 2001; Miranda-Filho Dde 2004; Saltoglu, 2004; Sanford, 1995; Thwaites, 2004; Trujillo, 1987). Mortality from tetanus is clearly dependent on age, immune status and vaccination. People over 60 years of age or unvaccinated persons have an elevated lethality of 18 and 22%, respectively. In the model, the mortality rate following symptomatic cases was set at 11% (CDC, 2012; Bardenheier, 1998).

Model input summary

Table 1. Transition probabilities used in the outcome tree

Health outcome<br>(Health state)	Distribution of health states in health outcome	Transition probability	Source/assumption
Symptomatic infection<br>(Localised tetanus)<br>(Generalised tetanus)<br>(Cephalic tetanus)	 13%<br>81%<br>6%		Bardenheier, 1998
Fatal cases		11%	CDC, 2012<br>Bardenheier, 1998

Table 2. Disability weights and duration

Health outcome (Health state)	Disability Weight (DW) (Haagsma, 2015)		Duration	
	DW	Label	In years	Source
Symptomatic infection				
(Generalised tetanus)	0.421 (0.377-0.477)	Motor impairment, severe	0.06-0.08	CDC, 2012
(Localised tetanus)	0.011 (0.008-0.014)	Motor impairment, mild	0.04-0.06	CDC, 2012
(Cephalic tetanus)	0.053 (0.042-0.064)	Motor impairment, moderate	0.04-0.06	CDC, 2012 Assumed same as for localised

References

Bardenheier B, Prevots DR, Khetsuriani N, Wharton M. Tetanus surveillance - United States, 1995-1997. MMWR CDC Surveill Summ 1998, 47:1- 13.

Bleck TP. Clostridium tetani (Tetanus). In Principles and practice of infectious diseases. Volume 2. Edited by Mandell GL, Bennett JE, Dolin R. Pennsylvania: Elsevier Inc.; 2005:2817–2822

Brook I. Tetanus in children. Pediatr Emerg Care 2004, 20:48-51.

Centers for Disease Control and Prevention: Tetanus. In Epidemiology and Prevention of Vaccine-Preventable Diseases. Edited by Atkinson W, Wolfe S, Hamborsky J. Washington DC: Public Health Foundation; 2012:291-300

Cook TM, Protheroe RT, Handel JM. Tetanus: a review of the literature. Br J Anaesth 2001, 87:477-487.

Dietz V, Milstien JB, van Loon F, Cochi S, Bennett J: Performance and potency of tetanus toxoid: implications for eliminating neonatal tetanus. Bull World Health Organ 1996, 74:619-628.

Farrar JJ, Yen LM, Cook T, Fairweather N, Binh N, Parry J, et al. Tetanus. J Neurol Neurosurg Psychiatry 2000, 69:292-301.

Guilfoile PG. Tetanus. New York: Infobase Publishing; Chelsea House; 2008.

Haagsma JA, Maertens de Noordhout C, Polinder S, Vos T, Havelaar AH, Cassini A, Devleesschauwer B, Kretzschmar ME, Speybroeck N, Salomon JA. Assessing disability weights based on the responses of 30,660 people from four European countries. Population Health Metrics 2015; 13: 10

Hsu SS, Groleau G. Tetanus in the emergency department: a current review. J Emerg Med 2001, 20:357-365.

Kanchanapongkul J. Tetanus in adults: a review of 85 cases at Chon Buri Hospital. J Med Assoc Thai 2001, 84:494-499.

Miranda-Filho Dde B, Ximenes RA, Barone AA, Vaz VL, Vieira AG, Albuquerque VM. Randomised controlled trial of tetanus treatment with anti- tetanus immunoglobulin by the intrathecal or intramuscular route. BMJ 2004, 328:615.

Murray CJL, Lopez AD. World Health Organization., World Bank., Harvard School of Public Health.: Global health statistics : a compendium of incidence, prevalence and mortality estimates for over 200 conditions. Geneva: World Health Organization; 1996.

Roper MH, Vandelaer JH, Gasse FL. Maternal and neonatal tetanus. Lancet 2007, 370:1947-1959.

Saltoglu N, Tasova Y, Midikli D, Burgut R, Dundar IH. Prognostic factors affecting deaths from adult tetanus. Clin Microbiol Infect 2004, 10:229- 233.

Sanford JP. Tetanus: forgotten but not gone. Waltham, MA, : Massachusetts Medical Society; 1995.

Thwaites CL, Yen LM, Nga NT, Parry J, Binh NT, Loan HT et al. Impact of improved vaccination programme and intensive care facilities on incidence and outcome of tetanus in southern Vietnam, 1993-2002. Trans R Soc Trop Med Hyg 2004, 98:671-677.

Trujillo MH, Castillo A, Espana J, Manzo A, Zerpa R. Impact of intensive care management on the prognosis of tetanus. Analysis of 641 cases. Chest 1987, 92:63-65.

Tick-borne encephalitis (TBE)

Most cases of tick-borne encephalitis (TBE) in Europe involve a biphasic presentation of the disease with fever during the first phase and neurological disorders during the second phase (Gubler, 2007). Severity of tick-borne encephalitis increases with age. TBE in children (<14 years) usually runs a more benign course (Mickiene, 2002; Kaiser, 1999). The proportion of asymptomatic cases is 66–80% (Gustafson, 1992). To calculate the burden of disease we assume that asymptomatic patients do not develop sequelae and are not included in the burden estimation.

The subtype considered is the Central European encephalitis subtype (Western tick-borne encephalitis virus) which is the dominant one in Europe. Another subtype does occur, the Russian spring-summer encephalitis subtype, however this occurs less in EU Member States and is not considered in the outcome tree.

The symptomatic infection (viraemic phase) begins after an average incubation period of eight days (range 4–28 days) (Kaiser, 1999). Symptoms of this first phase include fever, muscle pain, fatigue and headache (Gunther, 1997; Kaiser, 1999), normally lasting for five (2–7) days (Gubler, 2007).

Meningoencephalitic phase

After a symptom-free period, usually less than two weeks, a meningoencephalitic second phase occurs in 20–30% of symptomatic patients (Gustafson, 1990; 1992; Kiffner, 2010). The duration of the meningoencephalitic phase is set to 15 days (10–70) (Kaiser, 1999). The case fatality proportion of the meningoencephalitic phase is set to 0.75% (Mickiene, 2002).

Paralysis and residual paresis

Following the meningoencephalitic phase there is a latency period of six days (range 1–17 days), after which paralysis occurs in an estimated 11% of patients (Gunther, 1997). The duration is set to 3–10 days (Kaiser, 1999). Overall, 56% of paralytic patients are at risk of developing lifelong residual paresis (partial loss of or impaired movement) (Gunther, 1997).

Post-encephalitic TBE syndrome

A long-term post-encephalitic TBE syndrome, with symptoms including cognitive or neuropsychiatric complaints, balance disorders, headache, dysphasia, hearing defects and spinal paralysis, has been reported in 39–46% of meningoencephalitic patients (Gunther, 1997; Mickiene, 2002). The duration of post-encephalitic TBE syndrome is set to one year ('Post TBE syndrome existed after 1 year in more than one third of the patients' Gunther, 1997).

Lifelong chronic sequelae can persist in 35.7% (Haglund & Gunther, 2003) to 38.8% of post-encephalitic syndrome patients (Gunther, 1997: 'persisting symptoms at 12 months in 33/85 patients'). Males are affected twice as much as females and 12% of patients with post-encephalitic TBE syndrome were under 14 years of age (Kaiser, 1999). However, the association between gender, age and severity still needs more research and is not considered in the outcome tree.

Model input summary

Table 1. Transition probabilities used in the outcome tree

Health outcome (Health state)	Distribution of health states in health outcome	Transition probability	Source/assumption
Symptomatic infection		20–34%	Gustafson, 1992
Meningoencephalitic phase		20–30%	Gustafson, 1990, 1992; Kiffner, 2010
Paralysis		11%	Kaiser, 1999; Gunther, 1997
Residual paresis		56%	Gunther, 1997
Post-encephalitic TBE syndrome		39–46%	Gunther 1997; Mickiene, 2002
Chronic post-encephalitic TBE syndrome		35.7–38.8%	Haglund & Gunther, 2003<br>Gunther, 1997
Fatal cases following meningoencephalitic phase		0.75%	Mickiene, 2002

Table 2. Disability weights and duration

Health outcome (Health state)	Disability Weight (DW) (Haagsma, 2015)		Duration	
	DW	Label	In years	Source

Symptomatic infection	0.051 (0.039-0.06)	Infectious disease, acute episode, moderate	0.014 (0.005-0.019)	Gubler, 2007
Meningoencephalitic phase	0.447 (0.391-0.501)	Encephalopathy - severe	0.041 (0.027-0.192)	Kaiser, 1999
Paralysis	0.526 (0.469-0.586)	Spinal cord lesion at neck level (treated)	0.0137	Kaiser, 1999
Residual paresis	0.056 (0.044-0.067)	Motor plus cognitive impairments, mild	Remaining life expectancy	Remaining life expectancy
Post-encephalitic TBE syndrome	0.202 (0.167-0.242)	Motor plus cognitive impairments, moderate	1	Gunther, 1997
Chronic post-encephalitic TBE syndrome	0.056 (0.044-0.067)	Motor plus cognitive impairments, mild	Remaining life expectancy	Remaining life expectancy

References

Gubler JD, Kuno G, Markoff L. 2007. Flaviviruses. In: Knipe, D.M., Howley, P.M. (Eds.), Fields Virology 5th ed., vol. 1. Lippincott Williams & Wilkins, London, New York, Tokyo, pp. 1043–1125.

Gustafson R, Svenungsson B, Forsgren M, Gardulf A, Granström M. Two-year survey of the incidence of Lyme borreliosis and tick-borne encephalitis in a high-risk population in Sweden. European Journal of Clinical Microbiology and Infectious Diseases, 1992 Oct;11(10):894-900. PMID: 1486884

Gustafson R, Svenungsson B, Gardulf A, Stiernstedt G, Forsgren M. Prevalence of tick-borne encephalitis and Lyme borreliosis in a defined Swedish population. Scandinavian Journal of Infectious Diseases, 1990;22(3):297-306. PMID: 2371545

Günther G, Haglund M, Lindquist L, Forsgren M, Sköldenberg B. Tick-bone encephalitis in Sweden in relation to aseptic meningo-encephalitis of other etiology: a prospective study of clinical course and outcome. Journal of Neurology, 1997 Apr;244(4):230-8. PMID: 9112591

Haglund M, Günther G. Tick-borne encephalitis--pathogenesis, clinical course and long-term follow-up. Vaccine, 2003 Apr 1;21 Suppl 1:S11-8. PMID: 12628810

Haagsma JA, Maertens de Noordhout C, Polinder S, Vos T, Havelaar AH, Cassini A, Devleesschauwer B, Kretzschmar ME, Speybroeck N, Salomon JA. Assessing disability weights based on the responses of 30,660 people from four European countries. Population Health Metrics 2015; 13: 10

Kaiser R. The clinical and epidemiological profile of tick-borne encephalitis in southern Germany 1994-98: a prospective study of 656 patients. Brain 1999; 122 (Pt 11): 2067-78. PMID: 10545392

Kiffner C, Zucchini W, Schomaker P, Vor T, Hagedorn P, Niedrig M, et al. Determinants of tick-borne encephalitis in counties of southern Germany, 2001-2008. International Journal of Health Geography, 2010 Aug 13;9:42. PMID: 20707897

Mickiene A, Laiskonis A, Gunther G, et al. Tick-borne encephalitis in an area of high endemicity in Lithuania: disease severity and long-term prognosis. Clin Infect Dis 2002; 35 (6): 650-8 PMID: 12203160

Toxoplasmosis

Acquired toxoplasmosis

In Europe, most cases of acquired toxoplasmosis are asymptomatic and self-limiting (Rorman, 2006). Acquired toxoplasmosis will lead to symptomatic illness in approximately 10–20% of infected cases (Montoya, 2000). It is estimated that 4.67% (0–15.3%) of symptomatic cases will manifest more severe symptoms and approximately 2% (0–4.67%) are at risk of developing life-long sequelae relative to chorioretinitis. However, it is unclear if this risk is attributable mainly to more severe, symptomatic infections or all infections (Kemmeren, 2006). All other symptomatic cases will manifest minor symptoms, such as fever and lymphoadenopathy (Rorman, 2006; Anand, 2012).

Mortality due to acquired toxoplasmosis is extremely rare and occurs in immunocompromised patients. It has therefore been decided to exclude fatal cases from the outcome tree of acquired toxoplasmosis.

Toxoplasmosis may also play a role in the development of psychiatric disorders, such as schizophrenia and bipolar depression (Torrey, 2003; Henriquez, 2009; Brown, 2010). However, insight into causality is still insufficient and these sequelae are not included in the model.

Congenital toxoplasmosis

Vertical transmission from a recently infected pregnant woman to her foetus may lead to congenital toxoplasmosis. Infections occurring during the first and second trimester of pregnancy may result in foetal loss (1.5–1.7% of seroconverting pregnant women, Havelaar 2007) or stillbirth (although neither of these are included in the present burden estimation) and symptoms in newborn infants are generally more severe.

However, if the infection occurs in the third trimester the disease manifestation is generally subclinical. When present, symptoms vary from a triad including chorioretinitis, intracranial calcification and hydrocephalus to abnormalities of the central nervous system. These complications may lead to life-long sequelae, including subclinical congenital toxoplasmosis which could increase the risk of developing chorioretinitis later in life. Death can occur in a small proportion of infections. Other symptoms are very rare and have not been considered in this model.

Several studies have described clinical manifestations and follow-up of newborns infected with toxoplasmosis: 89% of children were asymptomatic at birth (16% of them developed chorioretinitis later in life) (Berrebi, 2010), 85% had no clinical findings at birth (Lebech, 1999) and 74.5% were asymptomatic at birth (Schmidt, 2006). Therefore, the proportion of asymptomatic infections out of the total congenital toxoplasmosis infections is 11–25%.

Asymptomatic congenital toxoplasmosis-infected infants have a 2% (1–3%) per year risk of developing chorioretinitis at a later age. The studies followed cases of asymptomatic congenital toxoplasmosis for 10–14 years (Havelaar, 2007).

Based on an extensive literature review, Havelaar et al. (Haavelar, 2007) estimated the risk of developing permanent disabilities related to congenital toxoplasmosis infections. We applied the same estimates to our model for all infections: 13% (12–15%) will develop permanent disabilities due to complications related to chorioretinitis, 11% (8–12%) to intracranial calcification, 3% (1-6%) to the central nervous system and 2% (1–3%) to hydrocephalus.

Model input summary

Table 1. Percentages used in the outcome tree

Health outcome<br><br>(Health state)	Distribution of health<br><br>states in health outcome	Transition probability	Source/assumption

Acquired toxoplasmosis			
Symptomatic infections:<br>(Uncomplicated)<br>(Complicated)	 Remaining cases<br>4.67% (0–15.3%)	10–20%	Kemmeren, 2006
Chorioretinitis following symptomatic infection		2% (0–4.67%)	Kemmeren, 2006
Congenital toxoplasmosis			
Symptomatic infections:<br>(Asymptomatic)<br>(Symptomatic)	 75–89%<br>Remaining cases		Berrebi, 2010<br>Lebech, 1999<br>Schmidt, 2006
Permanent disability due to chorioretinitis after the first year following asymptomatic infection		2% (1-3%) per year (ATP) for 10–14 years	Havelaar, 2007<br>Starting one year after infection up to the age of 10–14 years<br>ATP: Annual Transition Probability

Permanent disability due to chorioretinitis within first year		13% (12–15%)	Havelaar, 2007
Permanent disability due to intracranial calcification		11% (8–12%)	Havelaar, 2007
Permanent disability due to hydrocephalus		2% (1–3%)	Havelaar, 2007
Permanent disability due to CNS abnormalities		3% (1–6%)	Havelaar, 2007
Fatal cases		0.7% (0.4–1.2%)	Havelaar, 2007

Table 2. Disability weights and duration

Health outcome (Health state)	Disability Weight (DW) (Haagsma, 2015)		Duration	
	DW	Label	In years	Source
Acquired toxoplasmosis				
Acquired toxoplasmosis<br>(Uncomplicated)<br>(Complicated)	0.007 (0.005–0.01)<br>0.125 (0.104–0.152)	Infectious disease, acute episode, mild<br>Infectious disease, acute episode, severe	0.04	Kemmeren, 2006
Congenital toxoplasmosis				
Congenital toxoplasmosis<br>(Asymptomatic)<br>(Symptomatic)	0<br>0.125 (0.104–0.152)	Infectious disease, acute episode, mild<br>Infectious disease, acute episode, severe	1<br>0.167	Assuming chorioretinitis starts after one year<br>Melse, 2000
Permanent disability due to chorioretinitis following asymptomatic infections	0.015 (0.011–0.019)	Conjunctivitis without corneal scar	rem life exp.	Havelaar, 2007
Permanent disability due to	0.015 (0.011–0.019)	Conjunctivitis without corneal	rem life exp.	Havelaar, 2007

chorioretinitis following symptomatic infections		scar		
Permanent disability due to intracranial calcification	0.044–0.087	Intellectual disability/mental retardation, from mild to moderate	rem life exp.	Havelaar, 2007
Permanent disability due to hydrocephalus	0.044–0.188	Intellectual disability/mental retardation, from mild to severe	rem life exp.	Havelaar, 2007
Permanent disability due to CNS abnormalities	0.056–0.407	Motor plus cognitive impairments, from mild to severe	rem life exp.	Havelaar, 2007
Permanent disability due to chorioretinitis	0.015 (0.011–0.019)	Conjunctivitis without corneal scar	rem life exp.	Kemmeren, 2006

References

Anand R, Jones CW, Ricks JH, Sofarelli TA, Hale DC. Acute primary toxoplasmosis in travelers returning from endemic countries. J Travel Med. 2012; 19(1): 57-60.

Berrebi A, Assouline C, Bessieres MH, Lathiere M, Cassaing S, Minville V, et al. Long-term outcome of children with congenital toxoplasmosis. American journal of obstetrics and gynecology. 2010; 203(6): 552 e1-6.

Brown AS, Derkits EJ. Prenatal infection and schizophrenia: a review of epidemiologic and translational studies. Am J Psychiatry. 2010; 167(3): 261-80.

Burnett AJ, Shortt SG, Isaac-Renton J, King A, Werker D, Bowie WR. Multiple cases of acquired toxoplasmosis retinitis presenting in an outbreak. Ophthalmology. 1998; 105(6): 1032-7.

Haagsma JA, Maertens de Noordhout C, Polinder S, Vos T, Havelaar AH, Cassini A, Devleesschauwer B, Kretzschmar ME, Speybroeck N, Salomon JA. Assessing disability weights based on the responses of 30,660 people from four European countries. Population Health Metrics 2015; 13: 10

Havelaar AH, Kemmeren JM, Kortbeek LM. Disease burden of congenital toxoplasmosis. Clin Infect Dis. 2007; 44(11): 1467-74.

Henriquez SA, Brett R, Alexander J, Pratt J, Roberts CW. Neuropsychiatric disease and Toxoplasma gondii infection. Neuroimmunomodulation. 2009; 16(2): 122-33.

Kemmeren JM, Mangen, MJJ, van Duynhoven YTHP, Havelaar AH. Priority setting of foodborne pathogens: disease burden and costs of selected enteric pathogens. Bilthoven: National Institute of Public Health and Environment; 2006. Report No.: RIVM rapport 330080001.

Lebech M, Andersen O, Christensen NC, Hertel J, Nielsen HE, Peitersen B, et al. Feasibility of neonatal screening for toxoplasma infection in the absence of prenatal treatment. Danish Congenital Toxoplasmosis Study Group. Lancet. 1999; 353(9167): 1834-7.

Melse JM, Essink-Bot ML, Kramers PG, Hoeymans N. A national burden of disease calculation: Dutch disability-adjusted life-years. Dutch Burden of Disease Group. Am J Public Health. 2000; 90(8): 1241-7.

Montoya J, Remingtons J. Toxoplasma gondii. In: Mandell G, Douglas, Benett J, editors. Principles and Practice of Infectious Diseases. Philadelphia: Churchill Livingstone; 2000.

Rorman E, Zamir CS, Rilkis I, Ben-David H. Congenital toxoplasmosis--prenatal aspects of Toxoplasma gondii infection. Reprod Toxicol. 2006; 21(4): 458-72.

Schmidt DR, Hogh B, Andersen O, Fuchs J, Fledelius H, Petersen E. The national neonatal screening programme for congenital toxoplasmosis in Denmark: results from the initial four years, 1999-2002. Archives of disease in childhood. 2006; 91(8): 661-5.

Torrey EF, Yolken RH. Toxoplasma gondii and schizophrenia. Emerg Infect Dis. 2003; 9(11): 1375-80.

Tuberculosis

Tuberculosis (TB) is an infectious disease caused by the Mycobacterium tuberculosis. The term �tuberculosis� is also used for other similar diseases caused by M. bovis and M. africanum (Fitzgerald, 2005; Comstock, 1998). However, for the purposes of the disease report, outcome tree and model presented here, only those infections caused by M. tuberculosis complex are considered.

Tuberculosis bacteria are transmitted via droplets � by coughing, sneezing or talking � and mostly affect the lungs of humans, although they can also result in a systemic disease, affecting virtually all organs (Fitzgerald, 2005). The course �of TB can be split into several phases. The first phase after infection, �primary TB�, is observed in a minority of patients. The majority of infected (asymptomatic) persons proceed to a �latent� stage, lasting from months to several years or even for the rest of their life. Due to endogenous or exogenous reactivation, people may develop active TB after a certain time spent in the latent stage of the disease.

According to published literature only 5�10% of all infected individuals develop symptoms of active (primary) TB (cough, fever, lethargy, and weight loss) in their lifetime (Castillo-Chavez & Feng, 1997; Gideon & Flynn, 2011, Lin & Flynn, 2010, North & Jung, 2004).

Health outcomes and health states associated with tuberculosis infection

The main health outcomes associated with TB infection are active (primary) TB, MDR (multidrug-resistant) TB and XDR (extensively drug- resistant) TB. After initial infection with M. tuberculosis, an immuno-competent person is generally able to stop the replication and spread of bacilli and thus does not develop any symptoms. Primary TB can be split in pulmonary TB (the majority of cases) and extra-pulmonary TB, affecting different sites of the human organism. Given the complexity of the disease course, all TB cases are considered in the model, with a focus on the distinction between drug-susceptible (DS TB), MDR and XDR TB and their relative case fatality proportions (CFP), irrespective of the site of infection.

Of all laboratory-confirmed TB cases notified to ECDC/WHO between 2009 and 2013, on average 4.5% were multidrug-resistant and 14.6% of these cases were extensively drug resistant (ECDC/WHO, 2015). Therefore, in our model of all symptomatic infections 4.5% are considered to be MDR TB and 0.64% are considered to be XDR TB. However, it should be noted that these proportions vary widely across countries and users are advised to tailor them according to the epidemiology of the population under study.

Transition probabilities

In a cost-effectiveness analysis performed by Tseng and colleagues the authors used various assumptions on the progression of TB. Their model estimates the risk of active TB to be about 5% within the first two years of TB infection. Spontaneous resolution without treatment was set to 25%. Cure rates of TB with treatment and cure rates of MDR TB with treatment were 62.4% and 68.6% respectively (Tseng, 2011).

Tiemersma and colleagues estimated CFP and assessed durations of untreated pulmonary TB in HIV-negative patients and stated an overall case-fatality proportion of 30.7% in the first year of follow-up. The highest proportions were observed shortly after diagnosis. The 5-year and 10-year averages for case fatalities were 58% and 73% respectively (Tiemersma, 2011). In their review they also included the study conducted by Berg, estimating sex- and age-specific 10-year mortality rates. For men aged 15�29, 30�49 and >50 years, the 10-year mortality rates were 66%, 70% and 94% respectively. For women aged 15�29, 30�49 and >50 years, 10-year mortality rates were 70%, 69% and 92% respectively (Berg, 1951). Assuming that detected TB cases are treated in Europe, the case fatality proportions cited above overestimate current TB mortality patterns. Duration of pulmonary TB and TB is difficult to estimate due to difficulties in establishing onset of disease; based on estimates from prevalence and incidence studies an average duration of three years was suggested (Tiemersma, 2011).

A cost-effectiveness analysis using Markov models estimated active TB progression rates from underlying latent TB on the basis of disease duration and age-dependent case-fatality rates. Base case rates for developing active TB from latent TB within 1�2 years, 3�5 years and 6�7 years of exposure were estimated at 0.74%, 0.31% (0�2.5%), and 0.16% respectively. Age-specific death rates for people aged 35, 50 and 70 years were 1%, 5% and 10% respectively (Pisu, 2009).

Based on an international TB network, the US Centers for Disease Control and Prevention (CDC) and the World Health Organization (WHO) estimated that in 2004, of 17 960 TB isolates, 20% were MDR and 2% XDR. In population-based trials in the US, Latvia and South Korea 4%, 19% and 15% of all MDR TB cases were XDR in 2004. The studies in the US and Latvia also provided additional information on the progression of MDR and XDR TB in 2004. In the US study 55% of MDR patients completed treatment/were cured and 25% died during treatment. With regard to XDR, 31% completed treatment/were cured and 23% died. Results from Latvia show the percentage of completed treatment/cases cured of MDR TB to be 69% and that of deaths/failures to be 17%. For XDR 61% completed treatment/were cured and 17% died/or had failed treatment (CDC, 2006).

Jaquet and colleagues estimated the impact of DOTS[*] in Haiti and therefore conducted a cost-effectiveness analysis with probability estimates and outcome features of TB taken from literature. For reactivation of latent TB they estimated a probability of 0.1% per year for infection present for more than two years. Within two years of a new TB infection they estimated a base case rate of 5% (2�15%) for developing TB. Cure rates of treated smear positive (drug-sensitive) TB were estimated at 62.4%. For MDR TB, authors assumed a cure rate of 48% (base case; range 48�73%) and the proportion of deaths to be 12% (base case; range 12�26%) (Jacquet, 2006).

Outcome tree parameters

Given the changes in TB epidemiology in Europe during recent decades, the situation has not been sufficiently stable to enable incidence of infection to be estimated from active TB case data. It was therefore decided not to consider latent TB in the model.

Duration of symptomatic TB is set to 0.2�2 years, irrespective of whether it is active, MDR or XDR TB (WHO, 2014).

The case fatality proportion for active TB cases is estimated to be 43% in cases not on TB treatment (Corbett, 2003; Tiemersma, 2011) and 3% in cases on TB treatment (Straetemans, 2011). Given that the estimated incidence of active TB (non-MDR or XDR) in EU/EEA is 10% higher than the notification rate (ECDC/WHO, 2015) and, assuming that all notified cases are being treated, the CFP of active TB (non-MDR or XDR) cases was set at 7%.

The case fatality proportion for MDR TB was set at 12.8% (2.3�23.3%) (Straetemans, 2011). Given the lack of evidence on the case fatality ratio for XDR TB, we used the treatment outcome result category �Died�, notified in the EU/EEA, as a proxy for estimating the XDR TB case fatality proportion and set the value at 27% (ECDC/WHO, 2015).

Model input summary

Table 1. Transition probabilities and distributions used in the outcome tree

Health outcome (Health state)	Distribution of health states in health outcome	Transition probability	Source/assumption
Active TB<br>(Remaining active cases)<br>(MDR, non-XDR)<br>(XDR)	94.86%<br>4.5%<br>0.64%		ECDC/WHO, 2015
Fatal cases following remaining active cases		7%	Modelled based on Corbett, 2003; Tiemersma, 2011; Straetemans, 2011
Fatal cases following MDR TB		12.8% (2.3�23.3%)	Straetemans, 2011
Fatal cases following XDR TB		27%	ECDC/WHO, 2015

Table 2. Disability weights and duration

Health outcome (Health state)	Disability Weight (DW) (Haagsma, 2015)		Duration	
	DW	Label	In years	Source
Active TB<br>(Remaining active cases)<br>(MDR, non-XDR)<br>(XDR)	0.308 (0.264�0.353)<br>0.308 (0.264�0.353)<br>0.308 (0.264�0.353)	Tuberculosis, not HIV infected<br>Tuberculosis, not HIV infected<br>Tuberculosis, not HIV infected	2<br>2<br>2	WHO, 2013<br>WHO, 2013<br>WHO, 2013

References

Berg G. Statistik �ber die Tuberkulosemortalit�t in Kriegszeiten. Beitr�ge zur Klinik der Tuberkulose 1951, 106:1-9.

Castillo-Chavez C, Feng Z. To treat or not to treat: the case of tuberculosis. J Math Biol 1997, 35:629-656.

Centers for Disease Control and Prevention: Emergence of Mycobacterium tuberculosis with extensive resistance to second-line drugs - worldwide, 2000�2004. MMWR Morb Mortal Wkly Rep 2006, 55:301-305.

Comstock G, O'Brian R. Tuberculosis. In Bacterial Infections in Humans - Epidemiology and Control. Volume 3rd edition. Edited by Evans A, Brachman P. New York: Plenum Publishing Corporation; 1998: 777-804

Corbett EL, Watt CJ, Walker N, Maher D, Williams BG, et al. (2003) The growing burden of tuberculosis: global trends and interactions with the HIV epidemic. Arch Intern Med 163: 1009-1021.

Dye C, Scheele S, Dolin P, Pathania V, Raviglione M (for the WHO Global Surveillance Monitoring Project). Global burden of tuberculosis: Estimated incidence, prevalence, and mortality by country. JAMA 1999, 282:677-686.

European Centre for Disease Prevention and Control/WHO Regional Office for Europe. Tuberculosis surveillance and monitoring in Europe 2015.

European Centre for Disease Prevention and Control, WHO Regional Office for Europe: Tuberculosis surveillance and monitoring in Europe. In: Tuberculosis surveillance and monitoring in Europe. Stockholm: European Centre for Disease Prevention and Control; 2012.

Fitzgerald D, Haas D. Mycobacterium tuberculosis. In: Principles and Practice of Infectious Diseases. Sixth edition. Edited by Mandell G, Bennett J, Dolin R. Philadelphia: Elsevier Churchill Livingstone; 2005: 2852-2886

Gideon HP, Flynn JL. Latent tuberculosis: what the host "sees"? Immunol Res 2011, 50:202-212.

Global Tuberculosis Report 2013. 1.Tuberculosis � epidemiology. 2.Tuberculosis, Pulmonary � prevention and control. 3.Tuberculosis � economics. 4.Tuberculosis, Multidrug-Resistant. 5. Annual reports. World Health Organization. ISBN 978 92 4 156465 6. Available at: http://apps.who.int/iris/bitstream/10665/91355/1/9789241564656_eng.pdf

Global Tuberculosis Report 2014. 1.Tuberculosis � epidemiology. 2.Tuberculosis, Pulmonary � prevention and control. 3.Tuberculosis � economics. 4.Tuberculosis, Multidrug-Resistant. 5. Annual reports. World Health Organization. ISBN 978 92 4 156480 9. Available at: http://www.who.int/tb/publications/global_report/gtbr14_main_text.pdf?ua=1

Haagsma JA, Maertens de Noordhout C, Polinder S, Vos T, Havelaar AH, Cassini A, Devleesschauwer B, Kretzschmar ME, Speybroeck N, Salomon JA. Assessing disability weights based on the responses of 30,660 people from four European countries. Population Health Metrics 2015; 13: 10

Jacquet V, Morose W, Schwartzman K, Oxlade O, Barr G, Grimard F, et al. Impact of DOTS expansion on tuberculosis related outcomes and costs in Haiti. BMC Public Health 2006, 6:209.

Jassal M, Bishai WR. Extensively drug-resistant tuberculosis. Lancet Infect Dis 2009, 9:19-30.

Lin PL, Flynn JL. Understanding Latent Tuberculosis: A Moving Target. The Journal of Immunology 2010, 185:15-22.

Murray CJL, Lopez AD, World Health Organization, World Bank, Harvard School of Public Health. The global burden of disease: a comprehensive assessment of mortality and disability from diseases, injuries, and risk factors in 1990 and projected to 2020. Geneva: World Health Organization; 1996.

North RJ, Jung YJ. Immunity to tuberculosis. Annu Rev Immunol 2004, 22:599-623.

Pisu M, Gerald J, Shamiyeh JE, Bailey WC, Gerald LB. Targeted tuberculosis contact investigation saves money without sacrificing health. J Public Health Manag Pract 2009, 15:319-327.

Raviglione M. Tuberculosis - the essentials. New York: Informa Health Care; 2010.

Straetemans M, Glaziou P, Bierrenbach AL, Sismanidis C, van der Werf MJ. Assessing tuberculosis case fatality ratio: a meta-analysis. PLoS One 2011, 6:e20755.

Tiemersma EW, van der Werf MJ, Borgdorff MW, Williams BG, Nagelkerke NJ. Natural history of tuberculosis: duration and fatality of untreated pulmonary tuberculosis in HIV negative patients: a systematic review. PLoS One 2011, 6:e17601.

Tseng CL, Oxlade O, Menzies D, Aspler A, Schwartzman K. Cost-effectiveness of novel vaccines for tuberculosis control: a decision analysis study. BMC Public Health 2011, 11:55.

World Health Organization: Global tuberculosis control: epidemiology, planning, financing. WHO report 2009. Geneva: World Health Organization; 2009.

Variant Creutzfeldt-Jakob disease (vCJD)

The initial symptoms of variant Creutzfeldt-Jakob disease (vCJD) are usually psychiatric, most frequently depression, anxiety and withdrawal (Henry & Knight, 2002; Will & Ward, 2004). After a median of six months, neurological features develop, including cognitive impairment, ataxia and involuntary movements. The clinical course is progressive with the development of dementia and diffuse cortical deficits.

Death occurs after a median of 14 months from the onset of symptoms (range 6–39 months) and is often due to an intercurrent infection (Will & Ward, 2004). However, Henry and Knight stated that the disease is fatal after a median of 13 months and a range of 6–39 months (Henry & Knight, 2002).

In the study by Hilton (Hilton, 2006) the mean age at death for vCJD is 26 years and 29 years with a range of 12–74 years (Will & Ward, 2004; Smiths, 2004). This is in line with the overall median age of 28 at death for all vCJD diagnoses in the UK during the period January 1994– December 2009, with a range from 14 to 75 (Andrews, 2010). During the epidemic, the median age of onset did not change over time, suggesting an important age-related risk. This could be due to an age-dependent susceptibility, age-related exposure or both (Hilton, 2006). There is no significant difference in deaths between males and females (56% male, p=0.12).

Precise estimates of the length and variability of the incubation period for vCJD are difficult to obtain since they require knowledge of the time of infection, whereas exposure may have occurred over several years. Ghani assumes that the incubation period is approximately 15–18 years (Ghani, 2002), whereas Collinge concludes that the incubation period would be at least 11 years (Collinge, 1999).

Although a peak has passed, it is possible that there will be future peaks, possibly in other genetic groups. To date, all cases of vCJD have been genotyped as methionine homozygous at codon 129 of the PrP gene (about 40% of the population). If the other 60% of the population is not completely resistant to infection, the disease in these individuals is associated with a longer incubation period, therefore epidemics in this group may still occur (Smith, 2004). Kaski et al. reported the first suspected clinical case of vCJD in an individual heterozygous for methionine/valine (Kaski, 2009).

There is also the possibility of ongoing person-to-person transmission, as seen with three cases of vCJD infection following transfusion of packed red blood cells from asymptomatic donors who subsequently died from vCJD (Ironside, 2010). Furthermore, Peden et al. described a vCJD infection in the first known asymptomatic patient (Millar, 2010; Peden, 2010). The patient died from unrelated pathology with no evidence of neurological diseases. The infection was detected in a study of autopsy and biopsy materials from 17 neurologically asymptomatic patients with haemophilia, considered to be at increased risk of vCJD. The most likely route of infection was receipt of UK plasma products.

Finally, Smith assumes that the ascertainment of vCJD cases in young adults is nearly complete. In the absence of a reliable, minimally invasive, diagnostic test, the possibility remains that cases in the elderly are being missed due to the small proportion of those dying with dementia that are subject to post-mortem examination (Smiths, 2004).

Model input summary

Table 1. Transition probabilities and distributions used in the outcome tree

Health outcome<br><br>(Health state)	Percent of health<br><br>outcome in health state	Transition probability	Source/assumption
Fatal cases following symptomatic infection		100%	

Table 2. Disability weights and duration

Health outcome (Health state)	Disability Weight (DW) (Haagsma, 2015)		Duration	
	DW	Label	In years	Source
Symptomatic infection	0.407 (0.36–0.46)	Motor plus cognitive impairments, severe.	1.151 (0.5–3.205)	Will & Ward, 2004

References

Ang LW, Chua LT, James L, Goh KT. Epidemiological surveillance and control of rubella in Singapore, 1991-2007. Ann Acad Med Singapore. 2010 Feb;39(2):95-101.

Andrews NJ. Incidence of variant Creutzfeldt-Jakob disease diagnoses and deaths in the UK, January 1994 - December 2009. 2010 [updated 22 June 2010]; Available from: http://www.cjd.ed.ac.uk/cjdq64jun10.pdf.

Collinge J. Variant Creutzfeldt-Jakob disease. Lancet. 1999 Jul 24;354(9175):317-23.

Ghani AC. The epidemiology of variant Creutzfeldt-Jakob disease in Europe. Microbes Infect. 2002 Mar;4(3):385-93.

Haagsma JA, Maertens de Noordhout C, Polinder S, Vos T, Havelaar AH, Cassini A, Devleesschauwer B, Kretzschmar ME, Speybroeck N, Salomon JA. Assessing disability weights based on the responses of 30,660 people from four European countries. Population Health Metrics 2015; 13: 10

Henry C, Knight R. Clinical features of variant Creutzfeldt-Jakob disease. Rev Med Virol. 2002 May-Jun;12(3):143-50.

Hilton DA. Pathogenesis and prevalence of variant Creutzfeldt-Jakob disease. J Pathol. 2006 Jan;208(2):134-41.

Ironside JW. Variant Creutzfeldt-Jakob disease. Haemophilia. 2010 Jul;16 Suppl 5:175-80.

Kaski D, Mead S, Hyare H, Cooper S, Jampana R, Overell J, et al. Variant CJD in an individual heterozygous for PRNP codon 129. Lancet. 2009 Dec 19;374(9707):2128.

Millar CM, Connor N, Dolan G, Lee CA, Makris M, Wilde J, et al. Risk reduction strategies for variant Creutzfeldt-Jakob disease transmission by UK plasma products and their impact on patients with inherited bleeding disorders. Haemophilia. 2010 Mar;16(2):305-15.

Peden A, McCardle L, Head MW, Love S, Ward HJ, Cousens SN, et al. Variant CJD infection in the spleen of a neurologically asymptomatic UK adult patient with haemophilia. Haemophilia. 2010 Mar;16(2):296-304.

Smith PG, Cousens SN, d' Huillard Aignaux JN, Ward HJ, Will RG. The epidemiology of variant Creutzfeldt-Jakob disease. Curr Top Microbiol Immunol. 2004;284:161-91.

Will RG, Ward HJ. Clinical features of variant Creutzfeldt-Jakob disease. Curr Top Microbiol Immunol. 2004;284:121-32.

Campylobacteriosis

Acute gastroenteritis associated with Campylobacter infections in humans is in most cases self-limiting after a few days to weeks, but for some patients the disease may be fatal. When available, information on duration of illness mainly relates to cases having requested medical help. These cases are often the most severe cases of longer duration. For example, the overall mean duration of illness due to Campylobacter

infection observed in the GP[*] case control component of the IID study in England and Wales was 9.34 days, whereas it was only 6.52 days for all cases observed in the community component (Adak, 2002). About 47% of the community component cases would have visited their GP (Food Standards Agency, 2000). Based on the IID study, it is has been assumed that gastroenteritis caused by campylobacteriosis would last 3.22 days (no medical help), 9.72 days (visiting GP) and 14.39 days (hospitalised) (Mangen, 2004; Mangen, 2005). In our current model we chose to apply 3.22–9.72 days for all uncomplicated cases and 14.39 days for the complicated ones.

Bacteraemia is highlighted in many reports as a possible extra-intestinal complication of campylobacteriosis. For example, Skirrow et al. (Skirrow, 1993) estimated a bacteraemia incidence of 1.5 per 1 000 reported campylobacteriosis cases, whereas Ternhag et al. (Ternhag, 2008) reported an absolute risk of bacteraemia/sepsis of 0.02% for laboratory-confirmed campylobacteriosis cases.

Assuming that GP visits represent an indication of moderate diarrhoea and that the proportion of hospitalised cases represents severe diarrhoea, we divided cases into the following groups: uncomplicated (mild diarrhoea) 75.5%, complicated (GP, moderate diarrhoea) 23.5% and complicated hospitalised (severe diarrhoea) cases 1% (Kemmeren, 2006; Kwong, 2012; redistributing to total 100%).

Estimates of campylobacteriosis case fatality proportions range from 0.001% to 0.05%: 0.05% (Mead, 1999), 0.024% of all foodborne campylobacteriosis cases in the IID study (Adak, 2002), 2–6% of the hospitalised cases (Buzby, 1996; corresponding to 0.012–0.036% of all cases, considering that 0.6% of cases are hospitalised according to Mangen et al. 2004), 1.3 fatal cases per year, corresponding to 0.001% of the estimated 123 000 Campylobacter cases (Cressey & Lake, 2007), 0.038% of all symptomatic cases (Mangen, 2004).

We chose to estimate the overall case fatality proportion as being within the range 0.001–0.05% and assumed a different age-group distribution of this risk based on the age-group distribution of reported deaths to TESSy between 2009 and 2013 (see Table 3). This table is based on all TESSy notified cases from EU Member States except Bulgaria, reporting only aggregate data, Greece, Portugal and Liechtenstein which do not report.

Risk of complications

Reactive arthritis (ReA), irritable bowel syndrome (IBS) (but not inflammatory bowel disease due to lack of confirmation of a biological link and limited evidence) and Guillain-Barré syndrome (GBS) may be associated with campylobacteriosis.

Reactive arthritis (ReA)

ReA is a significant long-term sequelae following campylobacteriosis (Keat, 1983; Johnsen, 1983; Hannu, 2002). A retrospective study carried out in Finland found that 7.4% (45/609) of laboratory-confirmed campylobacteriosis cases fulfilled the criteria for ReA (Hannu, 2002), which is similar to that found by another study: 8.1% (3/37) (Johnsen, 1983). A further study reported a 2.6% (9 of 350) frequency of ReA in patients contacting a municipal health centre following an outbreak of C. jejuni (Hannu, 2004) and 16% of laboratory-confirmed cases self-reported having had ReA (Locht & Krogfeld, 2002), although self-reporting might be prone to overestimation (Hannu, 2002). Other studies including clinical testing report a 2.8% and a 2.4% risk of developing rheumatological symptoms (Rees, 2004; Kosunen, 1980). In order to account for the large uncertainty, the risk of developing ReA from all symptomatic cases is 1.7% (0.73–4.4%) (Kemmeren, 2006).

Little is known about the duration of ReA; the average duration is between 1.5 months derived from Hannu et al. (Hannu, 2005) and 222 days (Kemmeren, 2006).

Irritable Bowel Syndrome (IBS)

In a recent literature review, 8.8% (7.2–10.4%) of symptomatic campylobacteriosis symptomatic cases were considered at risk of developing IBS, irrespective of age and gender; the duration was set to five years (Haagsma, 2010). However, the causality is largely debated and the impact of concurrent factors significant. Therefore, IBS is not considered as part of the campylobacteriosis outcome tree in our study.

Guillain-Barré syndrome (GBS)

GBS is a neurological disease frequently preceded by an acute infectious illness, mainly upper respiratory infections and gastrointestinal infections. The functional status of patients with GBS is scored on a seven-point disability scale (F-score), ranking from 0 (healthy) to 6 (death). GBS-patients with an F-score at nadir of < 3 (able to walk unaided at nadir) are considered to be mildly affected. GBS patients with an F-score of ≥ 3 (unable to walk unaided at nadir) are considered to be severely affected (van Koningsveld, 2001). Paralysis from GBS is generally reversible over time, but some patients are bedridden for life and others die prematurely.

Incidence is estimated at 0.8–2.0 or 0.4–4 cases per 100 000 persons year (van Koningsveld, 2001; Mc Grogan, 2009; Hughes & Rees, 1997) in Europe and North America. A systematic review of the literature and metanalysis estimated an age-specific GBS rate per 100 000 person years of $\exp[-12.0771 + 0.01813(\text{age in years})] \times 100\ 000$ (Sejvar, 2011).

Studies show that 14–36% of GBS patients previously had a Campylobacter infection (Jacobs, 1998); 33–50% of GBS patients had increased levels of Campylobacter spp. (Mishu, 1993). A more recent systematic literature review estimated that 31% of the 2 502 GBS cases studied were attributable to Campylobacter infection (Poropatich, 2010).

Research has found that about 0.022% of laboratory-confirmed campylobacteriosis cases would develop GBS (13/57,425) (Ternhag, 2008), resulting for all symptomatic cases in a 0.0015% risk of developing GBS; in Sweden one GBS case per 3 285 Campylobacter jejuni infections (95% C.I.: 1.729 – 7.210) resulting in a risk of 0.03% (McCarthy & Giesecke, 2001); in the USA one per 1 058 campylobacteriosis cases (0.09% risk; Allos, 1997). Studies estimating the burden of campylobacteriosis assumed a 0.075% and 0.023% risk of developing GBS (Mangen, 2004 and 2005; Cressy & Lake, 2007). Given the large diversity found in the literature, the risk of developing GBS following a symptomatic Campylobacter infection is set to 0.0015–0.09%.

Males were more commonly affected by GBS in almost all studies (Sedano, 1994; Hughes & Rees, 1997; Nachamkin, 1998; Nagpal, 1999; van Koningsveld, 2000; Sejvar, 2011). However, these differences might be based on environmental factors as well as biological factors (van Koningsveld, 2000) and therefore it is difficult to speculate about the origin of this gender difference and the cause and determinants of GBS and therefore we do not distinguish in risk between genders.

Havelaar et al. (Havelaar 2000 a,b) estimated the proportion of mild and severe GBS cases after Campylobacter infections to be 17% and 83%, respectively. Age plays a role (van Koningsveld et al., 2000; Sejvar et al., 2011), we therefore assume that the age-group-specific distribution of the risk of developing a mild GBS is 17% and a severe GBS is 83% – see Table 4 and 5 (Havelaar 2000a, b). A total of 69% of mild GBS cases are under the age of 50, whereas for severe GBS cases this is only 48%.

The clinical course of GBS is highly variable. Very limited information is available for mildly affected patients. About 50% of the patients recover fully after six months, and the others have an F-score of 1. Most will recover after one year and the remainder will only suffer from minor symptoms (Havelaar, 2000a). We therefore assumed that mild cases will recover fully after one year.

There is a high heterogeneity among the severely-affected GBS patients: 60% of patients are reported to have an F-score of 4 when hospitalised, and approximately 20% of the patients had an F-score of 5 at nadir (Van der Meché, 1992). All patients recovered from intensive care, but after six months, 17% of them still had an F-score of 3 or 4. In a follow-up study the residual symptoms were evaluated up to six years after onset (Bernsen, 1997): only 25% recovered fully, whereas 44% of patients continued to suffer from minor symptoms (F-score=1) and 31% had functional limitations (F-score 2-4). Given that there had been no significant improvement since the acute phase, we assume that 17–31% of severely affected GBS patients would have permanent sequelae; this risk is distributed by age groups, see Table 6 (Havelaar 2000 a;b).

The case fatality rate for GBS ranges from 2–5% (Havelaar, 2000a) to 3.4% in a retrospective study (Van Koningsveld, 2000). However, generally only the severe cases are at risk of dying, therefore the risk is only estimated for these cases (CFR/83% severe cases x 100): 4.1% (2.41–6.02). The case fatality rate is age-dependent (Havelaar, 2000a) and strictly linked to the risk of developing permanent disabilities due to GBS; therefore, we apply the same age-group distribution as the risk of dying, see Table 6).

Model input summary

Table 1. Transition probabilities used in the outcome tree

Health outcome<br>(Health state)	Distribution of health states in health outcome	Transition probability	Source/assumption
Symptomatic infection<br>(Uncomplicated)<br>(Complicated, GP)<br>(Complicated, hosp)	 76%<br>23%<br>1%		Kemmeren, 2006; Kwong, 2012
Fatal cases following symptomatic infection		0.001–0.05%<br>Age dep. Table 3	Adak, 2002; Cressey & Lake, 2007; Mangen, 2005; Mead, 1999; TESSy 2009-2013
Reactive arthritis		1.7% (0.73–4.4%)	Kemmeren, 2006

Guillain-Barré syndrome (Mild)<br><br>(Severe)	17%<br>Age dep. Table 4<br><br>83%<br>Age dep. Table 5	0.0015–0.09%	Allos, 1987; Ternhag, 2008; Havelaar 2000a, b
Fatal cases following severe GBS		4.1% (2.41–6.02%)<br>Age dep. Table 6	Koningsveld, 2001; Havelaar, 2000a<br>Assuming only severe cases are fatal
Permanent disability following GBS		17–31%<br>Age dep. Table 6	Havelaar, 2000a, b<br>Assuming only severe cases

Table 2. Disability weights and duration

Health outcome (Health state)	Disability Weight (DW) (Haagsma, 2015)		Duration	
	DW	Label	In years	Source/assumption

Symptomatic infection<br><br>(Uncomplicated)<br>(Complicated, GP)<br>(Complicated, hosp)	 <br><br>0.073 (0.061–0.092)<br>0.149 (0.12–0.182)<br>0.239 (0.202–0.285)	 <br><br>Diarrhoea, mild<br>Diarrhoea, moderate<br>Diarrhoea, severe	 <br><br>0.009<br>0.027<br>0.039	Food Standard Agency, 2000; Mangen, 2004, 2005
Reactive arthritis	0.344 (0.3-0.391)	Musculoskeletal problems, generalized, moderate	0.131-0.608	Hannu, 2002; Kemmeren, 2006
Guillain-Barré syndrome<br>(Mild)<br>(Severe)	 <br>0.053 (0.042-0.064)<br><br>0.520 (0.465-0.581)	 <br>Motor impairment, moderate<br><br>Spinal cord lesion at neck level (treated)	 <br>1<br>1	Havelaar, 2000a, b
Permanent disability following GBS	0.421 (0.377-0.477)	Motor impairment, severe	Remaining life expectancy	Van der Meché, 1992; Bernsen, 1997

Table 3. Age-group distribution of the case fatality rate (0.001–0.05%)

Age groups	%
0	0.54
1-4	1.09
5-9	3.26
10-14	1.63
15-19	0.54
20-24	4.35
25-29	5.98
30-34	1.63
35-39	3.26
40-44	3.80
45-49	3.80
50-54	5.43

55-59	5.98
60-64	5.98
65-69	8.15
70-74	6.52
75-79	11.96
80-84	11.96
>85	14.13
All ages	100.00

Table 4. Age distribution mild GBS

Age	%
0	0.63
01-04	5.02
05-09	2.51
10-14	1.25
15-19	6.27
20-24	6.90

25-29	10.04
30-34	9.41
35-39	9.41
40-44	8.78
45-49	8.78
50-54	5.17
55-59	4.82
60-64	4.13
65-69	5.51
70-74	5.17
75-79	4.13
80-84	0.69
85+	1.38
Total	100

Table 5. Age distribution – severe GBS

Age	%
0	0.44
01-04	3.49
05-09	1.75
10-14	0.87
15-19	4.36
20-24	4.80
25-29	6.98
30-34	6.55
35-39	6.55
40-44	6.11
45-49	6.11
50-54	8.67
55-59	8.09
60-64	6.93
65-69	9.24
70-74	8.67
75-79	6.93

80-84	1.16
85+	2.31

Total	100

Table 6. Age distribution permanent GBS and case fatality rate

Age	%
0	0.00
01-04	0.00
05-09	0.00
10-14	0.00
15-19	0.00
20-24	1.56
25-29	1.56
30-34	1.56
35-39	1.56
40-44	2.08
45-49	2.08
50-54	2.08
55-59	6.25
60-64	6.25
65-69	6.25
70-74	18.75
75-79	25.00
80-84	18.75
85+	6.25
Total	100.00

References

Adak GK, Long SM, O'Brien SJ (2002). Trends in indigenous foodborne disease and deaths, England and Wales: 1992 to 2000. Gut 2002. 51:832- 841.

Allos BM. (1997). Association between Campylobacter infection and Guillain-Barré syndrome. J Infect Dis 1997.176(Suppl 2): S125-S128.

Bernsen RA, Jacobs HM, de Jager AE, van der Meché FGA (1997). Residual health status after Guillain-Barré syndrome. J. Neurol Neurosurg Psychiatry 1997. 62:637-640.

Buzby JC, Roberts T, Lin JCT, MacDonald JM (1996). Bacterial Foodborne Disease – Health care costs & Productivity losses. Washington, USA: Economic Research service, USDA. Agricultural Economic Report No 741.

Cressey P J and Lake R (2007). Risk ranking: Estimates of the burden of foodborne disease for New Zealand. Institute of Environmental Science & Research Limited; Christchurch Science Center.

Food Standards Agency (2000). A Report of the Study of Infectious Intestinal Disease in England. Food Standards Agency London: HMSO.

Haagsma JA, Havelaar AH, Janssen BM, Bonsel GJ. Disability Adjusted Life Years and minimal disease: application of a preference-based relevance criterion to rank enteric pathogens. Popul Health Metr 2008;6:7.

Haagsma JA, van der Zanden BP, Tariq L, van Pelt W, van Duynhoven YTHP, Havelaar AH (2009). Disease burden and costs of selected foodborne pathogens in the Netherlands, 2006. Bilthoven: National Institute of Public Health and Environment. RIVM rapport 330331001. Available online at: www.rivm.nl/bibliotheek/rapporten/330331001.pdf

Haagsma JA, Siersema PD, De Wit NJ, Havelaar AH. Disease burden of post-infectious irritable bowel syndrome in the Netherlands. Epidemiol Infect 2010.138(11):1650-6.

Haagsma JA, Maertens de Noordhout C, Polinder S, Vos T, Havelaar AH, Cassini A, Devleesschauwer B, Kretzschmar ME, Speybroeck N, Salomon JA. Assessing disability weights based on the responses of 30,660 people from four European countries. Population Health Metrics 2015; 13: 10

Hannu TL, Mattila H, Rautelin P, Pelkonen P, Lahdenne A, Siitonen M et al. Campylobacter-triggered reactive arthritis: a population-based study. Rheumatology 2002. 41(3):312-318.

Hannu TL, Kauppi M, Tuomala M, Laaksonen I, Klemets P, Kuusi M. Reactive arthritis following an outbreak of Campylobacter jejuni infection. J Rheumatol 2004. 31(3):528-30.

Havelaar AH, de Wit MAS, van Koningsveld R, van Kempen E (2000a). Health burden in the Netherlands due to infection with thermophilic Campylobacter spp. Epidemiol Infect 2000. 125(3):505-22.

Havelaar AH, de Wit MAS, van Koningsveld R (2000b). Health burden in the Netherlands (1990-1995) due to infection with thermophilic Campylobacter species. Bilthoven, the Netherlands: National Institute for Public Health and the Environment. Report no. 284550004.

Havelaar AH, Haagsma JA, Mangen MJJ, Kemmeren JM, Verhoef LPB, Vijgen SMC, et al. (2012a). Disease burden of foodborne pathogens in the Netherlands, 2009. International Journal of Microbiology in press.

Havelaar AH, Ivarsson S, Löfdahl M, Nauta MJ (2012b). Estimating the true incidence of campylobacteriosis and salmonellosis in the EU. Submitted.

Helms M, Simonsen J, Molbak K. Foodborne bacterial infection and hospitalization: a registry-based study. Clin Infect Dis 2006.42(4):498-506.

Hughes RAC and Rees JH (1997). Clinical and epidemiologic features of Guillain-Barré syndrome. Journal of infectious diseases 1997. 176 (suppl 2): S92-S98.

Jacobs BC, Rothbarth PH, van der Meché FG, Herbrink P, Schmitz PI, de Klerk MA, et al. (1998). The spectrum of antecedent infections in Guillain-Barré syndrome: a case-control study. Neurology 1998. 51(4):1110-1115.

Jess T, Simonsen J, Nielsen NM, Jørgensen KT, Bager P, Ethelberg S et al. (2011). Enteric Salmonella or Campylobacter infections and the risk of inflammatory bowel disease. Gut 2011;60(3):318-324.

Johnsen K, Ostensen M, Melbeye AC, Melby K (1983). HLA-B27-negative arthritis related to Campylobacter jejuni enteritis in three children and two adults. Acta Med Scand 1983. 214(2): 165-168.

Kemmeren JM, Mangen MJJ, van Duynhoven YTHP, Havelaar AH. (2006). Priority setting of foodborne pathogens: disease burden and costs of selected enteric pathogens. Bilthoven: National Institute of Public Health and Environment: RIVM rapport 330080001. Available online at: www.rivm.nl/bibliotheek/rapporten/330080001.pdf

Keat A. (1983). Reiter's syndrome and reactive arthritis in perspective. N Engl J Med 1983. 309(26):1606-15.

Kosunen TU, Kauranen O, Martio J, Pitkanen T, Ponka A, Hortling L, et al. (1980). Reactive arthritis after campylobacter jejuni enteritis in patients with HLA-B27. Lancet 1980. 1 (8181):1312-3.

Kwong JC, Ratnasingham S, Campitelli MA, Daneman N, Deeks SL, et al. (2012) The Impact of Infection on Population Health: Results of the Ontario Burden of Infectious Diseases Study. PLoS ONE 7(9): e44103. doi:10.1371/journal.pone.0044103

Lake RJ, Cressey PJ, Campbell DM, Oakley E (2010). Risk ranking for foodborne microbial hazards in New Zealand: burden of disease estimates. Risk Analysis 2010. 30(5):743-52.

Locht H, Krogfelt KA (2002). Comparison of rheumatological and gastrointestinal symptoms after infection with Campylobacter jejuni/coli and enterotoxigenic Escherichia coli. Ann Rheum Dis 2002.61(5): 448-52.

Mangen MJJ, Havelaar AH, de Wit GA. (2004). Campylobacteriosis and sequelae in the Netherlands - Estimating the disease burden and the cost-of-illness. Bilthoven: National Institute for Public Health and the Environment; Report nr. 250911004. Available online at: www.rivm.nl/bibliotheek/rapporten/250911004.pdf

Mangen MJJ, Havelaar AH, Bernsen RAJAM, van Koningsveld R, de Wit GA (2005). The costs of human Campylobacter infections and sequelae in the Netherlands: A DALY and cost-of-illness approach. 2005. Acta Agriculturae Scandinavica Section C Food Economics 2005. 2(1):35-51.

McCarthy N, Giesecke J (2001). Incidence of Guillain-Barre syndrome following infection with Campylobacter jejuni. Am J Epidemiol 2001. 153 (6): 610-614.

McGrogan A, Madle GC, Seaman HE, de Vries CS (2009). The epidemiology of Guillain-Barré syndrome worldwide. A systematic literature review. Neuroepidemiology 2009. 32(2):150-163.

Mead PS, Slutsker L, Dietz V, McCaig LF, Bresee JF, Shapiro C, et al. (1999). Food-related illness and death in the United States. Emerg Infect Dis 1999.5(5): 607-625.

Melse JM, Kramers PGN (1998). Berekening van de ziektelast in Nederland. Achtergronddocument bij VTV-1997; deel III, hoofdstuk 7. Bilthoven; Report nr. 431501028.

Mishu B, Ilyas AA, Koski CL, Vriesendorp F, Cook SD, Mithen FA, et al. (1993). Serological evidence of previous Campylobacter jejuni infection in patients with the Guillain-Barré Syndrome. Ann Intern Med 1993. 118(12): 947-953.

Nachamkin I, Allos BM, Ho T. (1998). Campylobacter species and Guillain-Barré syndrome. Clin Microbiol Rev 1998. 11(3): 555-567.

Nagpal S, Benstead T, Shumak K, Rock G, Brown M, Anderson DR (1999). Treatment of Guillain-Barré syndrome: a cost-effectiveness analysis. J. Clin Apheresis 1999. 14(3):107-113.

Poropatich KO, Walker CL, Black RE (2010). Quantifying the association between Campylobacter infection and Guillain-Barré syndrome: a systematic review. J. Health Popul Nutr; 2010. 28(6):545-52.

Sedano MJ, Calleja J, Canga E, Berciano J (1994). Guillain-Barré syndrome in Cantabria, Spain. An epidemiological and clinical study. Acta Neurol Scand 89(4): 287-292.

Sejvar JJ, Baughman AL, Wise M, Morgan OW (2011). Population incidence of Guillain-Barré syndrome: a systematic review and meta-analysis. Neuroepidemiology; 2011 36(2):123-133.

Skirrow MB, Jones DM, Sutcliffe E, Benjamin J (1993). Campylobacter bacteraemia in England and Wales, 191-91. Epidemiology and Infection 110 (3): 49-57.

Tam CC, Rodrigues IC, Viviani L, Dodds JP, Evans MR, Hunter PR et al. Longitudinal study of infectious intestinal disease in the UK (IID2 study): incidence in the community and presenting to general practice. Gut. 2012 Jan;61(1):69-77

Ternhag A, Törner A, Svensson A, Ekdahl K, Giesecke J (2008). Short- and long-term effects of bacterial gastrointestinal infections. Emerg Infect Dis 2008.14 (1):143-8.

Van der Meché FGA, Schmitz PIM and Dutch Guillain-Barré Study Group (1992). A randomized trial comparing intravenous immune globulin and plasma exchange in Guillain-Barré syndrome.N Engl. J. Med. 1992 326:1123-1129.

Van Koningsveld R, van Doorn PA, Schmitz PIM, Ang CW, van der Meché FGA (2000). Mild forms of Guillain-Barré syndrome in an epidemiological survey in the Netherlands. Neurology 54: 620-625.

Van Koningsveld R. (2001). Epidemiological and clinical aspects of the Guillain-Barré syndrome. Rotterdam, the Netherlands: Erasmus University Rotterdam. PhD-Thesis.

WHO (2004). Global Burden of Disease 2004 Update: Disability weights for diseases and conditions. Geneva: WHO. Available online at: http://www.who.int/healthinfo/global_burden_disease/GBD2004_DisabilityWeights.pdf

Yu DTY, Thomson GTD (1994). Clinical, epidemiological and pathogenic aspects of reactive arthritis. Food-Microbiology 1994. 11(2):97-108.

Chlamydia

Chlamydia trachomatis is a bacterium that causes a sexually transmitted infection (STI). WHO estimates a global annual incidence of about 90 million cases. Chlamydia trachomatis affects both women and men and can cause severe harm to the reproductive system of women. Additionally, children born to infected mothers are at high risk of developing severe complications (e.g. ophthalmia neonatorum, pneumonia). C. trachomatis has various serovars with different transmission modes and consequences. Serovars A, B, Ba and C, often transmitted by close eye- to-eye contact, cause ocular trachoma and are responsible for about 7–9 million cases of blindness (Stamm, 2005). Serovars D–K, responsible for genital infections, are associated with various adverse health outcomes in both men and women (Carey & Beagley, 2010). Serovars L1, L2 and L3 cause Lymphogranuloma venerum, a systemic STI mainly observed in the high-risk group of men having sex with men (MSM) (Martin- Iguacel, 2010). For the current outcome trees only serovars D–K responsible for genital infection are taken into consideration.

C. trachomatis mostly affects the young and sexually-active population with a female-male sex ratio of 1:0.7 (in tested individuals) (ECDC, 2014a). The genito-urinary infections present different disease patterns in the female and male hosts.

The asymptomatic infection poses serious threats to the health of the population as asymptomatic carriers represent a pool for new infections, and asymptomatic infections are associated with the risk of developing severe sequelae.

Rates of asymptomatic cases reported in literature vary widely. More than 50% of the infections due to C. trachomatis in males do not produce any symptoms or present a mild symptomatic illness (van de Laar & Morre, 2007). In a study of male army recruits, 85.6% of men testing positive for Chlamydia reported no symptoms (Cecil, 2001). Comparable rates were also reported by McKay and colleagues, with 88% of infected men being asymptomatic (McKay, 2003). Long-term sequelae due to chronic asymptomatic infections in men are still under discussion, but the pool of asymptomatic C. trachomatis carriers poses a serious threat to women's health due to continuous transmission and re-infection. Gaydos and Quinn refer to a percentage of asymptomatic male cases above 50%, in line with the above-mentioned estimates (Gaydos & Quinn, 2012).

Genital infections in women may present with short-term acute symptoms of cervicitis and urethritis (Stamm, 2005). Women also face a high number of asymptomatic infections. In total, 70–90% of all female and 50–88% of all male chlamydial infections do not present any symptoms (Stamm, 2005; Gaydos, 1998; Kalwij, 2010). Quinn and colleagues noted that around 79% of women with a Chlamydia infection attending a STI clinic were asymptomatic (Quinn, 1996). Clinical textbooks report a range of 70–90% of female cases being asymptomatic (Stamm, 2005; Gaydos & Quinn, 2012).

For the model we decided to use a range of 70–90% for the asymptomatic proportion (Stamm, 2005; Gaydos & Quinn, 2012) for female and 50– 88% for male cases (Stamm, 2005; Gaydos, 1998; Kalwij, 2010).

Health outcomes associated with chlamydial infection

Genital infection in men

Urethritis: with an incubation period of 7–14 days, urethritis causes symptoms of dysuria and urethral discharge (Stamm, 2005). We selected a range of 12–50% of infected men to represent symptomatic cases developing non-gonococcal urethritis (NGU) (Carey & Beagley, 2010; McKay, 2003).

Epididymitis: epididymitis is an acute inflammation of the epididymis (Carey & Beagley, 2010). The symptoms are oligospermia during the acute phase, swollen epididymis (and/or testicles), and dysuria. Fever and chills may occur in some cases. The association between epididymitis and future (in)fertility is an ongoing debate in research with no clear indication (Stamm, 2005).

Proctitis and proctocolitis: this clinical picture is most common in the MSM community. The classic symptoms are rectal pruritus, -pain and - bleeding. Fever often accompanies the initial proctitis and proctocolitis (Stamm, 2005; Carey & Beagley, 2010). This health outcome was not considered in the model due to lack of information.

Reactive arthritis: a further clinical picture is sexually-acquired reactive arthritis occurring as an acute aseptic arthritis or presenting as Reiter's syndrome. Reiter's syndrome includes symptoms of arthritis, conjunctivitis, urethritis and skin lesions (Stamm, 2005; Keat, 1983).

Genital infection in men can also include chronic pelvic pain. However, due to lack of information we decided not to include it in the model (Haggerty, 2010).

Genital infection in women

Urethritis/cervicitis

The acute form of C. trachomatis infection in women is urethritis and/or cervicitis. The majority of cases of both urethritis and cervicitis are asymptomatic, but can lead to severe sequelae (Low, 2007).

Pelvic inflammatory disease (PID)

Both symptomatic and asymptomatic infections can lead to serious consequences. Pelvic inflammatory disease is a commonly reported health outcome of a chlamydial infection. The literature shows very heterogeneous patterns regarding the transition probabilities from acute infection to PID. Carey and Beagley state that 12–50% of women infected with C. trachomatis develop PID (Carey & Beagley, 2010). In other literature the risk of PID after lower genital tract infection with Chlamydia varied from 0 to 30% (Risser & Risser, 2007) and from 0 to 72% (Boeke, 2005). Cates and Wasserheit reported that 40% of women with an untreated C. trachomatis infection develop PID (Cates & Wasserheit, 1991). Van Valkengoed and colleagues reported that complications of Chlamydia trachomatis infections are overestimated in the literature. They found five Cost Effectiveness Analyses (CEA) using decision trees to estimate the effect of screening programmes (Van Valkengoed, 2004). In these studies the estimates of the probability of developing PID after infection varied from 25 to 80%. ECDC has undertaken a systematic literature review and found a risk of developing PID from chlamydial infections of 9% (4–19%) (ECDC, 2014b).

Acute PID with pelvic pain, lasting for about 15 days, and silent PID with no or mild symptoms can cause severe long-term sequelae (Carey & Beagley, 2010; Westrom, 1980).

The estimated risk of tubal infertility as a sequelae of PID varies between 10–20% (Carey & Beagley, 2010; Lan, 1995; Land, 2010). Land and colleagues estimated the risk of tubal infertility after asymptomatic Chlamydia infection to be around 0.07% (Land, 2010). The risk of tubal infertility was found to be dependent on the course of infection (mild vs. severe) and the frequencies of re-infection (e.g. after three episodes of PID the risk is five-fold compared to a single episode.) ECDC's systematic review found that 16% of women with PID will develop infertility (ECDC, 2014b), which applies to women of reproductive age.

In total, 7–9% of pregnant women develop ectopic pregnancy after PID (Lan, 1995). Around 15% of women with previous PID develop chronic pelvic pain (Rogstad, 2008). Tubo-ovarian abscesses (tubal pathology) incur a risk of 7–16% for women who have previously had PID (Kottmann, 1995). The risk of cervical neoplasia is still under debate due to the fact that most cervical neoplasia are due to human papilloma virus (HPV) (Stamm, 2005).

Based on registration data from Amsterdam it was estimated that 0.07% and 0.02% of women exposed to chlamydia infection develop ectopic pregnancy and tubal factor infertility, respectively (Van Valkengoed, 2004).

Perinatal infections

Perinatal chlamydia may complicate as conjunctivitis (ophthalmia neonatorum) and neonatal pneumonia. We considered the ONBoID study for the input parameters which estimated that 15% of cases would develop ophthalmia neonatorum and 16% neonatal pneumonia (Kwong 2012). Assuming that in EU/EEA Member States all notified cases will have had symptoms, we used the same proportion: 48.39% are affected by ophthalmia and 51.61% will present pneumonia.

Outcome-tree parameters

Male outcome tree

For the male outcome tree a minimum of 50% and maximum of 88% was estimated as the percentage of asymptomatic cases (Carey & Beagley, 2010; McKay, 2003). The probability of developing epididymitis from symptomatic infections (10%) was taken from the World Health Organization STD Burden of Disease Study by Gerbase and colleagues (Gerbase, 2000). For asymptomatic infections a probability of 1–4% was taken from the cost effectiveness analysis of Welte and colleagues (Welte, 2001). Data on sexually acquired reactive arthritis (1% of symptomatic urethritis) and the resulting Reiters' syndrome (33% of reactive arthritis) were taken from a clinical text book (Stamm, 2005).

Female outcome tree

For the percentage of asymptomatic cases a range of 70–90% was included in the model (Stamm, 2005; Gaydos & Quinn, 2012; Gaydos, 1998; Kalwij, 2010; Stamm, 1999).

For the development of PID, estimates are included from the systematic review conducted by ECDC for the minimum (4%) (Van Valkengoed, 2004), maximum (19%) and most likely values (9%) (ECDC, 2014b).

The probability of developing ectopic pregnancy and tubal infertility after chlamydia infection is set to 0.07% and 0.02% respectively (Van Valkengoed, 2004). The probability of dying due to ectopic pregnancy was set to 0.038%, based on the study from Goldner (Goldner, 1993).

The risk of moving from PID to chronic pelvic pain was set at 18–75% and from PID to tubo-ovarian abscess at 0.8% (ECDC, 2014b; Ness, 2002, Soper 2010).

We decided to set the case fatality proportion for abscesses that have not ruptured to zero. Current mortality proportions for patients with ruptured abscesses are not reported in the literature; data from the 1960s suggested a mortality proportion ranging from 1.7 to 3.7 percent (Pedowitz, 2004; Paik, 2006). Due to the fact that these figures come from old studies and that diagnostics and treatment have significantly improved, we decided not to include the risk of dying from tubo-ovarian abscess.

Model input summary

Table 1. Transition probabilities and distributions used in the outcome tree

Health outcome (Health state)	Distribution of health states within health outcome	Transition probability	Source/assumption
Men			
Symptomatic infection		12–50%	Carey & Beagley, 2010; McKay, 2003
Epididymitis following symptomatic infection		10%	Gerbase, 2000
Reactive arthritis		1%	Stamm, 2005
(Mild)	67%		Stamm, 2005
(Severe)	33%		Stamm, 2005
Epididymitis following asymptomatic infection		1–4%	Gerbase, 2000; Welte, 2001

Women<br><br>Symptomatic infection		10–30%	Stamm, 1999; Stamm, 2005; Gaydos & Quinn, 2012; Gaydos, 1998; Kalwij, 2010
Pelvic inflammatory disease (PID)		9% (4–19%)	ECDC, 2014b
Tubo-ovarian abscess from PID		0.8%	Ness, 2002
Chronic pelvic pain after PID		18–75%	ECDC, 2014b; Soper 2010
Ectopic pregnancy		0.07%<br>Age dep. See Table 4	van Valkengoed, 2004<br>Female reproductive age 15-49
Tubal Infertility		0.02%<br>Age dep. See Table 4	Land, 2010; ECDC, 2014b<br>Female reproductive age 15-49
Fatal cases following ectopic pregnancy		0.038%	Goldner, 1993
Perinatal			
Symptomatic infection<br>(Neonatal pneumonia) (Ophthalmia neonatorum)	48.39%<br><br>51.61%		Kwong, 2012<br><br>Assuming that all reported cases have symptoms, we used the same proportion

Table 2. Disability weights and duration

Health outcome<br>(Health state)	Disability Weight (DW) (Haagsma, 2015)			Duration
	DW	ECDC European Disability Weight Project (2014)	In years	Source

Men				
Symptomatic infection	0.007 (0.005–0.01)	Infectious disease, acute episode, mild	0.02	Trojian, 2009
Epididymitis	0.176 (0.143–0.208)	Epididymo-orchitis	0.04	Murray, 1996
Reactive arthritis<br>(Mild)<br><br><br><br>(Severe)	 0.344 (0.3-0.391)<br><br><br><br>0.518 (0.457–0.576)	 Musculoskeletal problems, generalised, moderate<br>Musculoskeletal problems, generalised, severe	 0.13–0.28<br><br><br>0.41	 Özgül, 2006; Hannu, 2002<br><br><br><br>Miehle, 2003
Women				
Symptomatic infection	0.007 (0.005–0.01)	Infectious disease, acute episode, mild	0.03	Murray, 1996
Pelvic inflammatory disease (PID)	0.018–0.310	Abdominopelvic problem, mild to severe	0.04	Westrom, 1980
Tubo-ovarian abscess	0.31 (0.262–0.355)	Abdominopelvic problem, severe	0.01	Goharkhay, 2007; Teisala, 1990
Chronic pelvic pain	0.018–0.123	Abdominopelvic problem, mild to moderate	2.8	Sharma, 2011
Ectopic pregnancy	0.31 (0.262–0.355)	Abdominopelvic problem, severe	0.08	Murray, 1996
Tubal infertility	0.007 (0.005–0.01)	Infertility, secondary	See Table 3	Female reproductive age 15-49 (See Table 4)

Perinatal				
Neonatal pneumonia	0.125 (0.104–0.152)	Infectious disease, acute episode, severe	0.038	Zar, 2005<br><br>Assuming two weeks of treatment
Ophthalmia neonatorum	0.015 (0.011–0.019)	Conjunctivitis without corneal scar	0.038	American Academy of Pediatrics, 2012.<br>Assuming two weeks of treatment

Table 3. Duration of tubal infertility (female outcome tree)

Age	Duration in years
15–19	32
20–24	27
25–29	22
30–34	17
35–39	12
40–44	7
45–49	2

Table 4. Age-group risk (only reproductive age)

Age	%
0–14	0
15–49	100
≥50	0

References

American Academy of Pediatrics. Chlamydia trachomatis. In: Pickering LK, editor. Red book: 2012. Report of the Committee on Infectious Diseases. Elk Grove Village, IL: American Academy of Pediatrics; 2012. pp. 276–81.

Boeke AJ, van Bergen JE, Morre SA, van Everdingen JJ: [The risk of pelvic inflammatory disease associated with urogenital infection with Chlamydia trachomatis; literature review]. Ned Tijdschr Geneeskd 2005, 149:878-884.

Carey AJ, Beagley KW: Chlamydia trachomatis, a hidden epidemic: effects on female reproduction and options for treatment. Am J Reprod Immunol 2010, 63:576-586.

Cates W Jr. Wasserheit JN: Genital chlamydial infections: epidemiology and reproductive sequelae. Am J Obstet Gynecol 1991, 164:1771-1781.

Cecil JA, Howell MR, Tawes JJ, Gaydos JC, McKee KT, Quinn TC, et al. Features of Chlamydia trachomatis and Neisseria gonorrhoeae Infection in Male Army Recruits. Journal of Infectious Diseases 2001, 184:1216-1219.

European Centre for Disease Prevention and Control. Sexually transmitted infections in Europe 2012: ECDC; 2014a.

European Centre for Disease Prevention and Control. Chlamydia control in Europe: literature review. Stockholm: ECDC; 2014b.

Gaydos CA, Howell MR, Pare B, Clark KL, Ellis DA, Hendrix RM, et al. Chlamydia trachomatis Infections in Female Military Recruits. New England Journal of Medicine 1998, 339:739-744.

Gaydos CA, Quinn TC. Chlamydial infections. In Harrison's Principles of Internal Medicine. Volume 2. 18th Edition. Edited by Longo D, Fauci A, Kasper D, Hauser S, Jameson J, Loscalzo J. New York: McGraw Hill; 2012: 1421-1432

Gerbase A, Stein C, Levison J, Htun Y: Global burden of sexually transmitted diseases (excluding HIV) in the year 2000. In: Global burden of sexually transmitted diseases (excluding HIV) in the year 2000 (Ed.). World Health Organization; 2000.

Goldner TE, Lawson HW, Xia Z, Atrash HK. Surveillance for ectopic pregnancy—United States, 1970–1989. MMWR CDC Surveill Summ. 1993;42:73–85.

Goharkhay N, Verma U, Maggiorotto F: Comparison of CT- or ultrasound-guided drainage with concomitant intravenous antibiotics vs. intravenous antibiotics alone in the management of tubo-ovarian abscesses. Ultrasound Obstet Gynecol 2007, 29:65-69.

Haggerty CL, Gottlieb SL, Taylor BD, Low N, Xu F, Ness RB. Risk of sequelae after Chlamydia trachomatis genital infection in women. J Infect Dis 2010, 201 Suppl 2:S134-155.

Haagsma JA, Maertens de Noordhout C, Polinder S, Vos T, Havelaar AH, Cassini A, Devleesschauwer B, Kretzschmar ME, Speybroeck N, Salomon JA. Assessing disability weights based on the responses of 30,660 people from four European countries. Population Health Metrics 2015; 13: 10

Hammerschlag MR, Chandler JW, Alexander ER, English M, Koutsky L. Longitudinal studies on chlamydial infections in the first year of life. The Pediatric Infectious Disease Journal 1982, 1:395-401.

Hammerschlag MR. Chlamydial infections. J Pediatr 1989, 114:727-734.

Hannu TL, Mattila H, Rautelin P, Pelkonen P, Lahdenne A, Siitonen M et al. (2002) Campylobacter-triggered reactive arthritis: a population- based study. Rheumatology 41(3): 312-318.

Kalwij S, Macintosh M, Baraitser P. Screening and treatment of Chlamydia trachomatis infections. BMJ 2010, 340.

Keat A, Thomas BJ, Taylor-Robinson D. Chlamydial infection in the aetiology of arthritis. Br Med Bull 1983, 39:168-174.

Kottmann LM. Pelvic inflammatory disease: clinical overview. J Obstet Gynecol Neonatal Nurs 1995, 24:759-767.

Kwong JC, Ratnasingham S, Campitelli MA, Daneman N, Deeks SL, et al. (2012) The Impact of Infection on Population Health: Results of the Ontario Burden of Infectious Diseases Study. PLoS ONE 7(9): e44103. doi:10.1371/journal.pone.0044103

Lan J, van den Brule AJ, Hemrika DJ, Risse EK, Walboomers JM, Schipper ME, et al. Chlamydia trachomatis and ectopic pregnancy: retrospective analysis of salpingectomy specimens, endometrial biopsies, and cervical smears. J Clin Pathol 1995, 48:815-819.

Land JA, Van Bergen JE, Morre SA, Postma MJ. Epidemiology of Chlamydia trachomatis infection in women and the cost-effectiveness of screening. Hum Reprod Update 2010, 16:189-204.

Low N, McCarthy A, Macleod J, Salisbury C, Campbell R, Roberts TE, et al. Epidemiological, social, diagnostic and economic evaluation of population screening for genital chlamydial infection. Health Technol Assess 2007, 11:iii-iv, ix-xii, 1-165.

Martin-Iguacel R, Llibre JM, Nielsen H, Heras E, Matas L, Lugo R, et al. Lymphogranuloma venereum proctocolitis: a silent endemic disease in men who have sex with men in industrialised countries. Eur J Clin Microbiol Infect Dis 2010, 29:917-925.

McKay L, Clery H, Carrick-Anderson K, Hollis S, Scott G. Genital Chlamydia trachomatis infection in a subgroup of young men in the UK. Lancet 2003, 361:1792.

Miehle W. Medikamentöse Therapie rheumatischer Erkrankungen. Stuttgart: Georg Thieme Verlag; 2003.

Murray CJL, Lopez AD. The Global Burden of Disease - A comprehensive assessment of mortality and disability from diseases, injuries and risk factors in 1990 and projected to 2020. Cambridge: Harvard School of Public Health on behalf of the World Health Organization and the World Bank; 1996.

Ness RB, Soper DE, Holley RL, Peipert J, Randall H, Sweet RL, Sondheimer SJ, Hendrix SL, Amortegui A, Trucco G, Songer T, Lave JR, Hillier SL, Bass DC, Kelsey SF (2002) Effectiveness of inpatient and outpatient treatment strategies for women with pelvic inflammatory disease: results from the Pelvic Inflammatory Disease Evaluation and Clinical Health (PEACH) Randomized Trial. Am J Obstet Gynecol 186: 929-937.

Özgül A, et al. (2006). Clinical presentations of chlamydial and non-chlamydial reactive arthritis. Rheumatology International 26(10): 879-885.

Paik CK, Waetjen LE, Xing G, et al. Hospitalizations for pelvic inflammatory disease and tuboovarian abscess. Obstet Gynecol 2006; 107:611.

Pedowitz P, Bloomfield Rd. Ruptured adnexal abcess (tuboovarian) with generalized peritonitis. Am J Obstet Gynecol 1964; 88:721.

Peipert JF. Clinical practice. Genital chlamydial infections. N Engl J Med 2003, 349:2424-2430.

Quinn TC, Gaydos C, Shepherd M, Bobo L, Hook EW, Viscidi R, et al. Epidemiological and microbiological correlates of Chlamydia trachomatis infection in sexual partnerships. JAMA: Journal of the American Medical Association 1996, 276:1737-1742.

Risser WL, Risser JM. The incidence of pelvic inflammatory disease in untreated women infected with Chlamydia trachomatis: a structured review. Int J STD AIDS 2007, 18:727-731.

Rogstad K. Complications in the female and their management. In: International handbook of Chlamydia. 3rd Edition. Edited by Moss T. Haslemere, UK: Alden Press; 2008: 111-121

Salomon JA, Haagsma JA, Davis A, Maertens de Noordhout C, Polinder S, Havelaar AH, et al. Disability weights for the Global Burden of Disease 2013 study. The Lancet Global Health. [Accepted, in press.]

Sharma D, Dahiya K, Duhan N, Bansal R. Diagnostic laparoscopy in chronic pelvic pain. Arch Gynecol Obstet 2011, 283:295-297.

Soper DE. Pelvic inflammatory disease. Obstetrics and Gynecology, vol. 116, no. 2, part 1, pp. 419–428, 2010.

Stamm WE, Jones RB, Batteiger BE. Chlamydia trachomatis (Trachoma, Prinatal Infections, Lymphogranuloma Venerum and other genital Infections). In: Principles and practice of infectious diseases. Volume 2, 6th Edition. Edited by Mandel GL, Bennet JE, Dolin R. Pennsylvania: Elsevier Inc.; 2005: 2239-2255

Stamm WE. Chlamydia trachomatis infections of the adult. In: Sexually transmitted diseases. 3rd Edition. Edited by Holmes KK, Sparling PF, Mardh PA. New York: McGraw Hill; 1999:407–422

Trojian TH, Lishnak TS, Heiman D. Epididymitis and orchitis: an overview. Am Fam Physician 2009, 79:583-587.

Teisala K, Heinonen PK, Punnonen R. Transvaginal ultrasound in the diagnosis and treatment of tubo-ovarian abscess. Br J Obstet Gynaecol 1990, 97:178-180.

van de Laar MJ, Morre SA. Chlamydia: a major challenge for public health. Euro Surveill 2007, 12:E1-2.

van Valkengoed IG, Morre SA, van den Brule AJ, Meijer CJ, Bouter LM, Boeke AJ. Overestimation of complication rates in evaluations of Chlamydia trachomatis screening programmes--implications for cost-effectiveness analyses. Int J Epidemiol 2004, 33:416-425.

Welte R, Jager H, Postma MJ. Cost-effectiveness of screening for genital Chlamydia trachomatis. Expert Rev Pharmacoecon Outcomes Res 2001, 1:145-156.

Westrom L. Incidence, prevalence, and trends of acute pelvic inflammatory disease and its consequences in industrialized countries. Am J Obstet Gynecol 1980, 138:880-892.

Zar HJ. Neonatal chlamydial infections: prevention and treatment. Pediatric Drugs, vol. 7, no. 2, pp. 103–110, 2005

Cryptosporidiosis

Acute gastroenteritis associated with cryptosporidiosis in humans is in most cases self-limiting and symptoms disappear within a few days or weeks, but in very small number of cases the disease can be fatal.

We assumed that only a small proportion of cases (0.150%) experience the disease as more severe and complicated (Vijgen, 2007).

The average duration of the uncomplicated, mild disease is 3.5 days and 7–18.4 days for the complicated form (Vijgen, 2007).

The case fatality proportion was found to be 0.0042% (Vijgen, 2007), in line with 0.005% found in other studies (Mead, 1999). Mortality from acute gastroenteritis was assumed to be age-dependent and was redistributed according to the age-group-distributed cryptosporidiosis and giardiasis case fatality proportion reported to TESSy between 2009 and 2013 (see Table 3). This table is based on all TESSy notified cases from EEA Member States except Bulgaria, Poland (reporting only aggregate data), Austria, Czech Republic, Iceland, Luxembourg, Malta, Norway, Romania, Slovenia and Slovakia (because the very low incidence reported seems to indicate low sensitivity of the surveillance system).

Cryptosporidiosis can become chronic in immunocompromised persons, especially those with AIDS (Caccio and Pozio, 2006; Call, 2000; Pozio, 1997). However, several studies showed that AIDS-related cryptosporidiosis can be cured following successful antiretroviral therapy (Miao, 2000; Maggi, 2000; Foudraine, 1998).

Model input summary

Table 1. Transition probabilities and distributions used in the outcome tree

Health outcome (Health state)	Distribution of health states in health outcome	Transition probability	Source/assumption
Symptomatic infection			Vijgen, 2007
Uncomplicated)	99.85%		
Complicated)	0.15%		
Fatal cases following symptomatic infection		0.0042%<br>Age dependent (Table 3)	Vijgen, 2007;<br>TESSy 2009–2013

Table 2. Disability weights and duration

Health outcome (Health state)	Disability Weight (DW) (Haagsma, 2015)			Duration	

	DW	Label	In years		Source
Symptomatic infection					Vijgen, 2007
(Moderate)	0.073 (0.061–0.092)	Diarrhoea, mild	0.01		
(Severe)	0.239 (0.202–0.285)	Diarrhoea, severe	0.019–0.05		

Table 3. Age-group redistribution of case fatality proportion due to cryptosporidiosis (0.0042%)

Age groups	%
0	12.50
1–4	6.25
5–9	6.25
10–14	0.00
15–19	0.00
20–24	0.00
25–29	0.00
30–34	6.25

35–39	0.00
40–44	0.00
45–49	6.25
50–54	12.50
55–59	6.25
60–64	6.25
65–69	6.25
70–74	6.25
75–79	18.75
80–84	6.25
>85	0.00
All ages	100.00

References

Caccio SM, Pozio E (2006). Advances in the epidemiology, diagnosis and treatment of cryptosporidiosis. Expert Rev Anti Infect Ther. 4(3):429- 443.

Call SA, Heudebert G, Saag M, Wilcox CM (2000). The changing etiology of chronic diarrhea in HIV-infected patients with CD4 cell counts less than 200 cells/mm3. Am J Gastroenterol , 95(11):3142-3146.

Foudraine NA, Weverling GJ, van Gool T, Roos MT, de Wolf F, Koopmans PP, et al. (1998). Improvement of chronic diarrhoea in patients with advanced HIV-1 infection during potent antiretroviral therapy. Aids, 12(1):35-41.

Haagsma JA, Maertens de Noordhout C, Polinder S, Vos T, Havelaar AH, Cassini A, Devleesschauwer B, Kretzschmar ME, Speybroeck N, Salomon JA. Assessing disability weights based on the responses of 30,660 people from four European countries. Population Health Metrics 2015; 13: 10

Maggi P, Larocca AM, Quarto M, Serio G, Brandonisio O, Angarano G, et al. (2000) Effect of antiretroviral therapy on cryptosporidiosis and microsporidiosis in patients infected with human immunodeficiency virus type 1. Eur J Clin Microbiol Infect Dis, 19(3):213-217.

Mead PS, Slutsker L, Dietz V, McCaig LF, Bresee JS, Shapiro C, et al. (1999) Food-related illness and death in the United States. Emerg Infect Dis, 5(5):607-625.

Miao YM, Awad-El-Kariem FM, Franzen C, Ellis DS, Muller A, Counihan HM, et al. (2000) Eradication of cryptosporidia and microsporidia following successful antiretroviral therapy. J Acquir Immune Defic Syndr, 25(2):124-129.

Pozio E, Rezza G, Boschini A, Pezzotti P, Tamburrini A, Rossi P, et al. (1997) Clinical cryptosporidiosis and human immunodeficiency virus (HIV)- induced immunosuppression: findings from a longitudinal study of HIV-positive and HIV-negative former injection drug users. J Infect Dis, 176(4):969-975.

Vijgen SMC, Mangen MJM, Kortbeek LM, Van Duynhoven YTHP, Havelaar AH (2007). Disease burden and related costs of cryptosporidiosis and giardiasis in the Netherlands. 58 pp. Bilthoven.

Diphtheria

Thanks to vaccination, respiratory diphtheria has almost disappeared from many European countries. In total, 85% of patients suffer from subclinical disease or turn into asymptomatic carriers (Vitek, 1998) and only an estimated 15% of infections lead to a symptomatic case. The duration of acute illness was based on the Ontario Burden of Infectious Disease Study [AC1] ('the Ontario Study') [SW2] and set at 12 days (Kwong, 2012).

Risk of complications

Systemic toxicity (a toxic form of the disease with swelling of the neck) occurs in 8.1% of all diphtheria patients and may lead to complications such as myocarditis, neuropathies and renal failure (Rakhmanova, 1996). The more frequent complications of acute illness are myocarditis and polyneuropathies/nerve palsies. Other complications, such as sepsis, septic arthritis, pneumonia, otitis media, splenic and hepatic abscesses and rhinitis, were not included in the outcome tree because they are either extremely rare or mild.

Our model is based on the assumption that 8.1% of symptomatic patients would have a complicated form of the disease (Rakhmanova, 1996).

Permanent disability following myocarditis (arrhythmias)

Assuming that myocarditis represents 66.6% of the complicated diphtheria cases (Jayashree, 2006) and that 0.25% (Mandell, 1999) of these will develop permanent conduction defects (arrhythmias), the transition probability of patients with complications developing permanent cardiac disability is 0.17%.

Case fatality ratio

The US Centers for Disease Control and Prevention (US CDC) have reported a case-fatality proportion (CFP) of 5–10% for diphtheria, with higher death rates (up to 20%) among persons under five and over 40 years. The case fatality proportion has changed very little over the last 50 years (CDC, 2009).

In the model, the CFP associated with uncomplicated disease is 1% and with complicated disease 25.7% (Rakhmanova, 1996).

Model input summary

Table 1. Transition probabilities used in the outcome tree

Health outcome (Health state)	Distribution of health states in health outcome	Transition probability	Source/assumption
Symptomatic infection<br>(Uncomplicated)<br>(Complicated)	 91.9%<br>8.1%		Rakhmanova, 1996
Permanent disability (arrhythmias) following complicated symptomatic infection		0.17%	Jayashree, 2006; Mandell, 1999

Fatal cases following uncomplicated symptomatic infection		1%	Rakhmanova, 1996
Fatal cases following complicated symptomatic infection		25.7%	Rakhmanova, 1996

Table 2. Disability weights and duration

Health outcome (Health state)	Disability Weight (DW) (Haagsma, 2015)		Duration	
	DW	Label	In years	Source
Symptomatic infection (Uncomplicated) (Complicated)	0.051 (0.039-0.06) 0.125 (0.104-0.152)	Infectious disease, acute episode, moderate Infectious disease, acute episode, severe	0.003	Kwong 2012
Permanent disability (arrhythmias) following complicated symptomatic infection	0.295 (0.258-0.343)	Cardiac conduction disorders and cardiac dysrhythmias	Remaining life expectancy	

References

Centers for Disease Control and Prevention. Epidemiology and Prevention of Vaccine-Preventable Diseases. Atkinson W, Wolfe S, Hamborsky J, McIntyre L, eds. 11th ed. Washington DC: Public Health Foundation, 2009.

Demirci CS, Abuhammour W. Diphtheria. Available from: http://emedicine.medscape.com/article/963334-clinical#showall Accessed: 1 September 2011.

Hadfield TL, McEvoy P, Polotsky Y, Tzinserling VA, Yakovlev AA. The pathology of diphtheria. J Infect Dis. 2000;181(SUPPL. 1):S116-20

Haagsma JA, Maertens de Noordhout C, Polinder S, Vos T, Havelaar AH, Cassini A, Devleesschauwer B, Kretzschmar ME, Speybroeck N, Salomon JA. Assessing disability weights based on the responses of 30,660 people from four European countries. Population Health Metrics 2015; 13: 10

Jayashree M, Shruthi N, Singhi S. Predictors of outcome in patients with diphtheria receiving intensive care. Indian Pediatr. 2006;43(2):155-60.

Kwong JC, Ratnasingham S, Campitelli MA, Daneman N, Deeks SL, et al. (2012) The Impact of Infection on Population Health: Results of the Ontario Burden of Infectious Diseases Study. PLoS ONE 7(9): e44103. doi:10.1371/journal.pone.0044103

Lodha R, Dash NR, Kapil A, Kabra SK. Diphtheria in urban slums in north India. Lancet, 2000 Jan 15;355(9199):204. PMID: 10675127

Mandell GL, Bennet JE, Dolin R. Mandell, Douglas and Bennett's principles and practice of infectious disease 5th edition. Churchill Livingstone, 1999.

Merck Manual available at http://www.merckmanuals.com/professional/infectious_diseases/gram-positive_bacilli/diphtheria.html?qt=diphtheria&alt=sh#v1005972 (last update May 2013)

Rakhmanova AG, Nosikova E, Tanasijchuk T, Saikku J. Diphtheria Outbreak in St. Petersburg: Clinical Characteristics of 1,860 Adult Patients. Scand J Infect Dis 1996; 28: 37-40.

Solders G, Nennesmo I, Perssont A, Diphtheritic neuropathy, an analysis based on muscle and nerve biopsy and repeated neurophysiological and autonomic function tests. Journal of Neurology, Neurosurgery, and Psychiatry 1989;52:876-880

Vitek CR, Wharton M. Diphtheria in the former soviet union: Reemergence of a pandemic disease. Emerging Infectious Diseases. 1998;4(4):539-50.

WHO (2004). Global Burden of Disease 2004 Update: Disability weights for diseases and conditions. Geneva: WHO. Available online at: http://www.who.int/healthinfo/global_burden_disease/

GBD2004_DisabilityWeights.pdf

Giardiasis

Acute gastroenteritis associated with giardia in humans is in most cases self-limiting within a few weeks (Wolfe, 2000). Vijgen et al. (Vijgen, 2007) assumed for their disease burden estimates a mean duration of 10 days for gastroenteritis cases not requiring medical help or requiring a visit to the doctor. Severe hospitalised gastroenteritis cases were assumed to last for 30 days.

We assumed that the proportion of more severe cases requiring hospitalisation would be 0.265% (360 cases requiring hospitalisation out of an estimated 136 000 incident cases) (Vijgen, 2007). Moreover, the study presents an age-specific risk of hospitalisation which we applied to the 'severe' health state of the symptomatic infection outcome (see Table 3).

The Dutch Association of Parasitology is not aware of fatal cases of giardia (Vijgen, 2007). Additionally, studies by Adak et al. (Adak, 2002) and Levy et al. (Levy, 1998) have not reported fatal cases.

However, a small number of deaths associated with giardiasis were reported to TESSy: nine cases between 2009 and 2013, resulting in 0.014% of notified cases. The CFP is applied to all symptomatic cases and re-distributed according to the age-group observed deaths for giardiasis and cryptosporidiosis notified between 2009 and 2013 from all Member States, with the exception of Denmark, France, Greece, Italy, Liechtenstein, the Netherlands and Portugal, because they do not report (see Table 4). Data from Bulgaria and Poland were also excluded because they only report aggregate data. It is important to note that the CFP will increase in case multipliers adjusting for under-estimation are applied to the incidence inputted in the toolkit and this should be taken into account.

Risk of complications

Apart from Irritable Bowel Syndrome (IBS) as a possible sequela of giardia, no other sequelae could be identified. However, given the fact that few studies expressed a statistical link between IBS and giardia (1–2%) (Nygard, 2006; Hanevik, 2009; Haagsma, 2010), IBS was not included as a possible complication.

Model input summary

Table 1. Transition probabilities used in the outcome tree

Health outcome<br><br>(Health state)	Distribution of health states in health outcome	Transition probability	Source/assumption
Symptomatic infection<br>(Uncomplicated)<br>(Complicated)	99.735%<br>0.265%<br>Age dep. (Table 3)		
Fatal cases following		0.014%	TESSy 2009-2013

symptomatic infection		Age dependent (Table 4)	

Table 2. Disability weights and duration

Health outcome (Health state)	Disability Weight (DW) (Haagsma, 2015)		Duration	
	DW	Label	In years	Source/assumption
Symptomatic infection				Vijgen, 2007
(Moderate)	0.149 (0.12–0.182)	Diarrhoea, moderate	0.027	
(Severe)	0.239 (0.202-0.285)	Diarrhoea, severe	0.082	

Table 3. Age distribution of severe cases

Age class	%
0–4	27
5–9	27
10–14	3
15–64	34
≥65	8

Table 4. Age-group redistribution of CFR (applied only to complicated cases)

Age groups	%
0	12.50
1–4	6.25
5–9	6.25
10–14	0.00
15–19	0.00
20–24	0.00
25–29	0.00
30–34	6.25
35–39	0.00
40–44	0.00
45–49	6.25
50–54	12.50
55–59	6.25
60–64	6.25
65–69	6.25
70–74	6.25
75–79	18.75
80–84	6.25
>85	0.00
All ages	100.00

References

Adak GK, Long SM, O'Brien SJ. Trends in indigenous foodborne disease and deaths, England and Wales: 1992 to 2000. Gut, 2002. 51(6):832-841.

Food Standards Agency. A Report of the Study of Infectious Intestinal Disease in England. Food Standards Agency London, 2000. HMSO.

Haagsma JA, Siersema PD, De Wit NJ, Havelaar AH. Disease burden of post-infectious irritable bowel syndrome in the Netherlands. Epidemiol Infect 2010. 138(11):1650-6.

Haagsma JA, Maertens de Noordhout C, Polinder S, Vos T, Havelaar AH, Cassini A, Devleesschauwer B, Kretzschmar ME, Speybroeck N, Salomon JA. Assessing disability weights based on the responses of 30,660 people from four European countries. Population Health Metrics 2015; 13: 10

Hanevik K, Dizdar V, Langeland N, Hausken T. Development of functional gastrointestinal disorders after Giardia lamblia infection. BMC Gastroenterol, 2009. 9:27.

Lengerich EJ, Addiss DG, Juranek DD. Severe giardiasis in the United States. Clin Infect Dis, 1994.18(5):760-763.

Levy DA, Bens MS, Craun GF, Calderon RL, Herwaldt BL. Surveillance for waterborne-disease outbreaks – United States, 1995–96. MMWR CDC Surveill Summ, 1998. 47(5):1-34.

Nygard K, Schimmer B, Sobstad O, Walde A, Tveit I, Langeland N. A large community outbreak of waterborne giardiasis-delayed detection in a non-endemic urban area. BMC Public Health, 2006. 6:141.

Vijgen SMC, Mangen MJM, Kortbeek LM, Van Duynhoven YTHP, Havelaar AH. Disease burden and related costs of cryptosporidiosis and giardiasis in the Netherlands. 58 pp. Bilthoven. 2007. National Institute of Public Health and the Environment.

Wolfe MS. Giardiasis. Clin Microbiol Rev, 1992. 5(1):93-100.

Gonorrhoea

Gonorrhoea is the second most commonly reported sexually transmitted disease (STD) in the United States of America (Skolnik & Neil, 2008). Neisseria gonorrhoeae is almost exclusively transmitted by sexual contact and perinatally (from mother to child during labour) (Handsfield & Sparling, 2005). The bacteria affect the mucous membranes of the urethra and the cervix. Less frequently, mucous membranes of the rectum, oropharynx and conjunctivae are also involved during infection. N. gonorrhoeae primarily infects columnar and cuboidal epithelium. Gonorrhoeal infections in women may lead to pelvic inflammatory disease (PID) and may be a cause of female infertility. Further complications resulting from infection with N. gonorrhoeae are epididymitis, ophthalmitis, ectopic pregnancy and disseminated gonococcal infection (DGI). Untreated infections mostly resolve spontaneously over time (several weeks or months) but can lead to serious sequelae associated with adverse effects on health. Even though the duration of disease is hard to estimate, mean duration is assumed to be several days for men and less than two weeks for women. The incubation period is short and re-infection is common (Handsfield & Sparling, 2005).

The true number of gonorrhoea cases is largely affected by under-estimation due to high percentages of asymptomatic cases and diagnosed cases not being reported to the surveillance system. It was estimated that the true number of new infections is twice as high as the reported number (CDC, 2002). Brunham and Embree reported that gonorrhoea is posing serious threats in Africa, Latin America, Asia and eastern Europe (Brunham & Embree, 1992). In 2008, WHO estimated that there were around 46.8 million cases of STDs in the European Region, with 3.4 million cases being due to N. gonorrhoeae (WHO, 2012).

About 40–80% of women are asymptomatically infected (De Maio & Zenilman, 1998; Nelson, 2007). For men symptomatic rates of up to 95- 99% were observed for genital infection (De Maio & Zenilman, 1998; Nelson, 2007; Stamm, 2005).

Health outcomes and health states associated with gonococcal infection

Infection with N. gonorrhoeae results in different clinical pictures in women, men and infants. In our study, we only considered disease models which reflect genital infection; pharyngeal and rectal infections are not considered to be the cause of significant short or long-term sequelae and therefore do not contribute to the burden of gonorrhoea.

Infections in men

An uncomplicated infection presents as an acute urethritis, infection in the pharynx or rectum are likely to be asymptomatic. In 2013, 36% of reported gonorrhoea cases were detected at these sites. In most cases (95–99%) the disease has a symptomatic course with typical signs of dysuria and urethral discharge (De Maio & Zenilman, 1998; Nelson, 2007; Stamm, 2005). In a few cases the infection remains asymptomatic and is neither recognised nor diagnosed (Sherrard, 1996). These infections pose a serious problem as they provide a pool of further transmissible infections. In most cases gonococcal urethritis resolves spontaneously over several weeks but may also trigger sequelae (Handsfield & Sparling, 2005).

The most common sequela of gonococcal infections in men is the acute epididymitis (Stamm, 2005; Trojian, 2009). The symptoms associated with epididymitis are oligospermia during the acute phase, swollen epididymis (and/or testicles), and dysuria. The association between epididymitis and future infertility is an ongoing debate in research with no clear evidence (Stamm, 2005). Uncommon complications are penile oedema, penile lymphangitis, periurethral abscess, acute prostatitis, seminal vasculitis and Tyson's or Cowper's gland infections (Handsfield & Sparling, 2005). Due to their rare occurrence they are not considered in the outcome tree.

Infections in women

Uncomplicated infections in women mostly affect the endocervix and N. gonorrhoeae are also recovered from the urethra, rectum or occasionally from the periurethral (Skene's) glands and the ducts of Bartholin's glands. Many women with gonococcal infections only develop minor symptoms or are entirely asymptomatic and thus do not seek medical advice and are consequently not reported to the surveillance system.

A major complication resulting in remarkable disease burden is pelvic inflammatory disease (PID) (Handsfield & Sparling, 2005; De Maio & Zenilman, 1998). Studies report 10–40% of infected women developing PID (Handsfield, 1974; McCormack, 1977; Westrom, 1980; Westrom, 1992). In a cost effectiveness analysis, Bernstein and colleagues estimated a base case scenario of 30% (range 10–40%) of infected women developing PID (Bernstein, 2006). Women with

PID have an increased risk of developing infertility in the future (Handsfield & Sparling, 2005; De Maio & Zenilman, 1998; Westrom, 1980; Westrom, 1992; Ross, 2002). The study of Weström (1992) and colleagues reported a 10% probability of infected women developing tubal infertility. The risk of infertility is linked to number and severity of PID episodes. Ross reported 15–20% and 50–80% of infected women developing tubal infertility after one and three or more PID episodes, respectively. PID itself is also a cause of further (long-term) sequelae such as chronic pelvic pain, ectopic pregnancy and perihepatitis. Pelvic pain occurs in 20% of cases and ectopic pregnancy in 9.1% of PID cases (Handsfield & Sparling, 2005; Westrom, 1980). Infections with N. gonorrhoeae during pregnancy can result in spontaneous abortion, premature labour, early rupture of fetal membranes and perinatal infant mortality (Handsfield & Sparling, 2005). The cost effectiveness study by Bernstein and colleagues estimated transition probabilities from PID to chronic pelvic pain, ectopic pregnancy and tubal factor infertility of 18% (range 15–30), 7.8% (range 7.8–9.1%), and 15% (range 9–18%), respectively (Bernstein, 2006).

Sequelae reported for both sexes

As a result of bacteraemic dissemination, disseminated gonococcal infection (DGI) can occur in 0.5–3% of people infected with N. gonorrhoeae. This may cause infective arthritis and also be the cause of endocarditis and meningitis in very rare cases (Holmes, 2007).

Gonococcal infections in infants

Infants born to infected mothers can suffer from gonococcal conjunctivitis (ophthalmia neonatorum). Gonococcal conjunctivitis affects 30–35% of children born to infected mothers and is a major problem in many developing countries causing blindness (De Maio & Zenilman, 1998; Nelson, 2007). Ophthalmia neonatorum can lead to corneal scars, resulting in low-vision or complete blindness. Effective treatment is available which has led to very low numbers of sequelae resulting from ophthalmia neonatorum in the developed world (Darling, 2010; Schaller & Klauss, 2001). Consequently, we did not consider corneal-scar-related ‘low-vision’ or ‘blindness’ in our model.

Infected infants may have a low birth weight; some studies relate low birth weight to gonococcal infections (15% from Gerbase, 2000), however the attribution of this condition to the infection is extremely difficult in a developed country setting. Therefore, we decided to discard this relationship.

Case fatality proportion

Fatal cases resulting from gonococcal infections are extremely rare and mainly result from endocarditis, meningitis and DGI. Estimating the mortality of PID is complicated due to the lack of standardised case definitions, inconsistent reporting practices and unclear aetiology (percentage of fatal cases attributable to gonococcal PID) (De Meaio & Zenilman, 1998).

Outcome tree parameters

Male outcome-tree

The proportion of infections in men who develop symptoms is set at 95–99% (De Maio & Zenilman, 1998; Trojian, 2009, Nelson, 2007). The probability of developing DGI (which is part of the initial symptomatic phase of the disease) is set at 0.5–3% (Holmes, 2007), whereas the probability of developing epididymitis is set to 3% (1–5%) (Bernstein, 2006). Debate is currently ongoing as to whether asymptomatic cases also develop epididymitis, however, due to lack of a proven association, this was not taken into account.

Female outcome-tree

Information on the proportion of symptomatic (20–60%) and asymptomatic (40–80%) gonococcal infections were taken from reviews, clinical text books and a study conducted by Weström (Handsfield & Sparling, 2005; De Maio & Zenilman, 1998; Nelson, 2007; Westrom, 1992). Information on PID as a major sequela were obtained from reviews, clinical text books and a cost effectiveness analysis which provided an estimate that 30% (10–40%) of women were symptomatically infected (Bernstein, 2006). The probabilities of developing an ectopic pregnancy (7.8-9.1%), chronic pelvic pain (18%, range 15–30%) or tubal infertility (15%, range 9–18%) were taken from Bernstein`s cost-effectiveness study (Bernstein, 2006). Case fatality proportions from ectopic pregnancies were estimated at 0.038% (Goldner, 1993). The probability of developing a tubo-ovarian abscess is set at 0.8% (Ness, 2002). However, diagnosis and treatment have significantly improved it was therefore decided not to include a case fatality event for tubo-ovarian abscess.

Congenital outcome-tree

The burden studies on STDs by Gerbase and colleagues and Nelson et al. report 30–35% of cases developing ophthalmia neonatorum (Nelson, 2007; Gerbase, 2000).

Assuming that in EU/EEA Member States all notified cases will have had symptoms, in our model all cases of symptomatic infant gonococcal infections manifest as ophthalmia neonatorum and will represent the only health state included in the model.

Model input summary

Table 1. Transition probabilities and distributions used in the outcome tree

Health outcome	Distribution of health states in health	Transition probability	Source/assumption

(health state)	outcome		
Men			
Symptomatic infection<br>(Urethritis)<br>- Uncomplicated<br>- Complicated	97–99.5%<br>0.5–3%	95–99%	De Maio & Zenilman, 1998; Nelson, 2007; Stamm, 2005<br>Holmes, 2007
Epididymitis from symptomatic		3% (1–5%)	Bernstein, 2006
Women			
Symptomatic infection<br>(Cervicitis)<br>- Uncomplicated<br>- Complicated	97–99.5%<br>0.5–3%	20–60%	Handsfield & Sparling, 2005; De Maio & Zenilman, 1998; Nelson, 2007;<br>Westrom, 1992;<br>Holmes, 2007
Pelvic Inflammatory Disease (PID) from symptomatic and asymptomatic		30% (10–40%)	Bernstein, 2006
Ectopic pregnancy		7.8–9.1%<br>Age dep.<br>See Table 4	Bernstein, 2006<br>Female reproductive age 15-49
Tubo-ovarian abscess		0.8%	Ness, 2002
Chronic pelvic pain syndrome		18% (15–30%)	Bernstein, 2006

Tubal infertility		15% (9–18%)<br>Age dep.<br>See Table 4	Bernstein, 2006<br>Female reproductive age 15-49
Fatal cases due to ectopic pregnancy		0.038%	Goldner, 1993
Congenital			
Symptomatic infection<br>(Ophthalmia neonatorum)		100%	

Table 2. Disability weights and duration

Health outcome<br>(health state)	Disability Weight (DW) (Haagsma, 2015)		Duration	
	DW	Label	In years	Source
Men				
Uncomplicated<br>Complicated	0.051 (0.039-0.06)<br>0.125 (0.104-0.152)	Infectious disease, acute episode, moderate<br>Infectious disease, acute episode, severe	0.02<br>0.02	Trojian, 2009<br>Trojian, 2009
Epididymitis	0.176 (0.143-0.208)	Epididymo-orchitis	0.08	Trojian, 2009
Women				
Uncomplicated<br>Complicated	0.051 (0.039-0.06)<br>0.125 (0.104-0.152)	Infectious disease, acute episode, moderate<br>Infectious disease, acute episode, severe	0.03<br>0.03	Murray, 1996<br>Murray, 1996
Pelvic Inflammatory Disease (PID)	0.123 (0.1-0.15)	Abdominopelvic problem, moderate	0.07	De Maio & Zenilman, 1998
Tubo-ovarian abscess	0.31 (0.262-0.355)	Abdominopelvic problem, severe	0.01	Goharkhay, 2007; Teisala, 1990
Chronic pelvic pain	0.123 (0.1-0.15)	Abdominopelvic problem, moderate	2.8	Sharma, 2011
Ectopic pregnancy	0.31 (0.262-0.355)	Abdominopelvic problem, severe	0.08	Murray, 1996
Tubal infertility	0.007 (0.005-0.01)	Infertility, secondary	See Table 3	Female reproductive age 15–49 years<br>See Table 4

Congenital				
Symptomatic infection (Ophthalmia neonatorum)	0.015 (0.011-0.019)	Conjunctivitis without corneal scar	0.038	American Academy of Pediatrics, 2012. Assuming 2 weeks of treatment

Table 3. Duration of tubal infertility

Age	Duration in years
15–19	32
20–24	27
25–29	22
30–34	17
35–39	12

40–44	7
45–49	2

Table 4. Age group risk (only reproductive age)

Age	%
0–14	0
15–49	100
≥50	0

References

Adelman D, Casale T, Corren J. Manual of Allergy and Immunology. Philadelphia, USA: Lippincott, Williams and Wilkins; 2002.

American Academy of Pediatrics. Chlamydia trachomatis. In: Pickering LK, editor. Red book: 2012. Report of the Committee on Infectious Diseases. Elk Grove Village, IL: American Academy of Pediatrics; 2012:276–81.

American Social Health Association. Sexually Transmitted Diseases in America: How Many Cases and at What Cost? In: Sexually Transmitted Disease in America: How Many Cases and at What Cost? (Ed.). City; 1998:1-27.

Barr J, Danielsson D. Septic gonococcal dermatitis. Br Med J 1971, 1:482-485.

Bernstein KT, Mehta SD, Rompalo AM, Erbelding EJ. Cost-effectiveness of screening strategies for gonorrhea among females in private sector care. Obstet Gynecol 2006, 107:813-821.

Brunham R, Embree J. Sexually transmitted diseases: Current and future dimensions of the problem of the Third World. In: Reproductive Tract Infections Global Impact on Priorities for Women's Reproductive Health. Edited by Germanie A, Holmes K, Piot P, Wasserheit N. New York: Plenum Press; 1992: 35-38

Centers for Disease Control and Prevention. Sexually Transmitted Disease Surveillance, 2002. Atlanta: US Department of Health and Human Services; 2003.

Darling EK, McDonald H. A Meta-analysis of the Efficacy of Ocular Prophylactic Agents Used for the Prevention of Gonococcal and Chlamydial Ophthalmia Neonatorum. Journal of Midwifery & Women's Health 2010, 55:319-327.

DeMaio J, Zenilman J. Gonococcal Infections. In: Bacterial Infections of Humans: Epidemiology and Control. Edited by Evans A, Brachman P. New York: Plenum Medical Book Company; 1998: 285-304

Gerbase A, Stein C, Levison J, Htun Y. Global burden of sexually transmitted diseases (excluding HIV) in the year 2000. In: Global burden of sexually transmitted diseases (excluding HIV) in the year 2000 (Ed.) City: World Health Organization; 2000.

Goharkhay N, Verma U, Maggiorotto F. Comparison of CT- or ultrasound-guided drainage with concomitant intravenous antibiotics vs. intravenous antibiotics alone in the management of tubo-ovarian abscesses. Ultrasound Obstet Gynecol 2007, 29:65-69.

Goldner TE, Lawson HW, Xia Z, Atrash HK. Surveillance for ectopic pregnancy—United States, 1970–1989. MMWR CDC Surveill Summ. 1993;42:73–85.

Haagsma JA, Maertens de Noordhout C, Polinder S, Vos T, Havelaar AH, Cassini A, Devleesschauwer B, Kretzschmar ME, Speybroeck N, Salomon JA. Assessing disability weights based on the responses of 30,660 people from four European countries. Population Health Metrics 2015; 13: 10

Handsfield HH, Lipman TO, Harnisch JP, Tronca E, Holmes KK. Asymptomatic gonorrhea in men. Diagnosis, natural course, prevalence and significance. N Engl J Med 1974, 290:117-123.

Handsfield HH, Sparling RF. Neisseria gonorrhoeae. In Principles and Practice of Infectious Diseases. Volume Volume 2; 6th Edition. Edited by Mandel GL, Bennet JE, Dolin R. Peensylvania: Elsevier Inc.; 2005: 2514-2529

Holmes KK et al. Sexually Transmitted Diseases. 4th Edition 2007. McGraw-Hill Companies: New York. ISBN: 978-0071417488

Marra F, Marra CA, Patrick DM. Cost effectiveness analysis of azithromycin and doxycycline for Chlamydia trachomatis infection in women: A Canadian perspective. Can J Infect Dis 1997, 8:202-208.

McCormack WM, Stumacher RJ, Johnson K, Donner A. Clinical spectrum of gonococcal infection in women. Lancet 1977, 1:1182-1185.

Murray CJL, Lopez AD, World Health Organization, World Bank, Harvard School of Public Health. Global health statistics: a compendium of incidence, prevalence and mortality estimates for over 200 conditions. Geneva: World Health Organization; 1996.

Murray CJL, Lopez AD. The Global Burden of Disease - A comprehensive assessment of mortality and disability from diseases, injuries, and risk factors in 1990 and projected to 2020. Cambridge: Harvard School of Public Health on behalf of the World Health Organization and the World Bank; 1996.

Nelson AL. Gonorrheal Infections. In: Sexually Transmitted Infections - Current Clinical Practice. Edited by Nelson AL, Woodward J, Wysocki S: Humana Press; 2007: 153-182

Ness RB, Soper DE, Holley RL, Peipert J, Randall H, Sweet RL, et al. Effectiveness of inpatient and outpatient treatment strategies for women with pelvic inflammatory disease: results from the Pelvic Inflammatory Disease Evaluation and Clinical Health (PEACH) Randomized Trial. Am J Obstet Gynecol 2002. 186: 929-937.

Newman LM, Moran JS, Workowski KA. Update on the management of gonorrhea in adults in the United States. Clin Infect Dis 2007, 44 Suppl 3:S84-101.

Ross JD. An update on pelvic inflammatory disease. Sex Transm Infect 2002, 78:18-19.

Rowley J, Berkely S (1998). Sexually Transmitted Diseases. In: Health Dimensions of Sex and Reproduction. The Global Burden of Disease and Injuries Series, Volume 3. Harvard University Press, Cambridge.

Schaller UC, Klauss V. Is Crede's prophylaxis for ophthalmia neonatorum still valid? Bull World Health Organ 2001, 79:262-263.

Sharma D, Dahiya K, Duhan N, Bansal R. Diagnostic laparoscopy in chronic pelvic pain. Arch Gynecol Obstet 2011, 283:295-297.

Sharp JT. Editorial: Gonococcal infections and arthritis. Arthritis Rheum 1974, 17:511-512.

Sherrard J, Barlow D. Gonorrhoea in men: clinical and diagnostic aspects. Genitourin Med. 1996;72:422–6.

Skolnik NS, Neil S. Sexually Transmitted Diseases. In: Essential Infectious Disease Topics in Primary Care - Current Clinical Practice. Edited by Albert RH: Humana Press; 2008: 105-166

Spencer SE, Bash MC. Extragenital manifestations of Neisseria gonorrhoeae. Curr Infect Dis Rep 2006, 8:132-138.

Stamm WE, Jones RB, Batteiger BE. Chlamydia trachomatis (Trachoma, prinatal infections, Lymphogranuloma venerum and other genital infections). In Principles and Practice of Infectious Diseases. Volume 2, 6th Edition. Edited by Mandel GL, Bennet JE, Dolin R. Pennsylvania: Elsevier Inc.; 2005: 2239-2255

Teisala K, Heinonen PK, Punnonen R. Transvaginal ultrasound in the diagnosis and treatment of tubo-ovarian abscess. Br J Obstet Gynaecol 1990, 97:178-180.

Trojian TH, Lishnak TS, Heiman D. Epididymitis and orchitis: an overview. Am Fam Physician 2009, 79:583-587.

Westrom L, Joesoef R, Reynolds G, Hagdu A, Thompson SE. Pelvic inflammatory disease and fertility. A cohort study of 1,844 women with laparoscopically verified disease and 657 control women with normal laparoscopic results. Sex Transm Dis 1992, 19:185-192.

Westrom L. Incidence, prevalence, and trends of acute pelvic inflammatory disease and its consequences in industrialized countries. Am J Obstet Gynecol 1980, 138:880-892.

Wise CM, Morris CR, Wasilauskas BL, Salzer WL. Gonococcal arthritis in an era of increasing penicillin resistance. Presentations and outcomes in 41 recent cases (1985-1991). Arch Intern Med 1994, 154:2690-2695.

World Health Organization. Global incidence and prevalence of selected curable sexually transmitted infections - 2008. Geneva: World Health Organization; 2012.

Hepatitis A

Hepatitis A virus (HAV) infections range from asymptomatic health state to fulminant hepatitis (Jeong & Lee, 2010). Hepatitis A symptomatic infections depend strongly on the age: approximately 30% of infected children develop symptoms (Jeong & Lee, 2010; Ciocca, 2000), whereas, according to literature, this is 70–80% for adults (Jeong & Lee, 2010; Ciocca, 2000; Cuthbert, 2001). The manifestation of HAV infection in young children generally includes mild flu-like, but anicteric symptoms (Gingrich, 1983), whereas in adults frequently reported symptoms are jaundice, dark urine, fatigue, loss of appetite, abdominal pain and light-coloured stool lasting for several weeks (Koff, 1992).

Not only severity, also duration is related to the age of the patient. Symptoms in young children last for one to two weeks (Gingrich, 1983). According to Koff, around 80% of adults are ill for up to eight weeks (Koff, 1992). Haagsma et al. assumed that symptomatic HAV cases not requiring medical help would have symptoms for 14 days, and symptomatic HAV cases requiring any kind of medical help would have symptoms for 30 days (Haagsma, 2009). Havelaar et al. assumed that hospitalised HAV cases would have symptoms for up to 0.3 years(Haavelar, 2012). According to the US Centers for Disease Control and Prevention, clinical illness usually does not last longer than two months, although 10–15% of persons have prolonged or relapsing signs of symptoms for up to six months (CDC, 2012).

The case fatality proportions are reported to be 0.1% (Mead, 1999), 1% of hospitalised HAV cases (Arteaga Rodriguez, 2010) and 0.3% (Bauch, 2007; Fiore, 2004).

Fatal cases occur mainly in elderly people (Bauch, 2007; Jacobs, 2004; Jacobs, 2000). In the following table we have summarised the rates of mortality attributable to HAV as used in various cost-effectiveness analyses (Bauch, 2007; Jacobs, 2004; Jacobs, 2000).

Table 1. Deaths among symptomatic patients per 10 000 stratified for age classes

Age classes (in years)		Sources	
	Bauch 2007	Jacobs 2004	Jacobs 2000
	30	-	
5-14	18	-	
15-19	18	-	18 (6-30)
20-29	18	18	18 (6-30)
30-39	21	21	21 (10-32)
40-49	59	36	36 (23-49)

50-59	59	81	81 (70-92)
60-69	272	149	149 (146-152)
70-79	272	283	283 (154-310)
>80	272	283	385 (356-414)

We chose to consider the overall case fatality proportion to be within the range 0.1–0.3% and assumed a different age-group distribution of this risk based on the age-group distribution of fatal cases reported to TESSy between 2009 and 2013 (see Table 4). This table is based on all TESSy notified cases from EU Member States except Bulgaria, Lithuania, Latvia and Poland, because they report only aggregate data, and Liechtenstein which does not report.

Risk of complications

Fulminant hepatitis is a rare complication of hepatitis (Jeong & Lee, 2010). According to Bauch et al. (Bauch, 2007), the probability of fulminant infection in hospitalised HAV cases is 0.011%. Jacobs et al. (Jacobs, 2004) assumed that the probability of liver transplantation would be 0.02% for symptomatic HAV cases in 25 to 29-year olds, increasing slightly with age to 0.08% for symptomatic HAV cases in 70-year olds. According to Jeong and Lee (Jeong & Lee, 2010), a liver transplantation may be necessary, however HAV-related fulminant hepatitis does resolve spontaneously on a more frequent basis than fulminant hepatitis of other aetiologies. Given the low incidence, and the resulting negligible burden, fulminant hepatitis was not considered as a separate health outcome in the current study.

In a current review (Jeong & Lee, 2010), rare atypical clinical manifestations and extra-hepatic manifestations are listed. Atypical clinical manifestations occasionally reported are: relapsing hepatitis, prolonged cholestasis, and complicated cases with acute kidney injury. Rarely reported extra-hepatic manifestations are autoimmune haemolytic anaemia, aplastic anaemia, pure red cell aplasia, pleural or pericardial effusion, acute reactive arthritis, acute pancreatitis, acalculous cholecystitis, mononeuritis, and Guillain-Barré syndrome. None of these manifestations were considered in a recent disease burden study (Havelaar, 2012), nor in cost-effectiveness studies evaluating HAV vaccination programmes (Bauch, 2007; Jacobs, 2000, 2004).

Model input summary

Table 2. Transition probabilities used in the outcome tree

Health outcome (Health state)	Distribution of health states in health outcome	Transition probability	Source/assumption
Fatal cases		0.1–0.3%. Age-dependent (Table 4)	Mead 1999, Bauch 2007, Fiore 2004

Table 3. Disability weights and duration

Health outcome (Health state)	Disability Weight (DW) (Haagsma, 2015)		Duration	
	DW	Label	In years	Source/assumption
Symptomatic infection	0.125 (0.104–0.152)	Infectious disease, acute episode, severe	0–9 years: 0.019–0.038 ≥ 10 years: 0.082 (0.038–0.5). See Table 5.	CDC 2012; Haagsma 2009, age-dependent

Table 4. Age-group redistribution of case fatality proportion (0.1–0.3%)

Age groups	%
0	0.00
1-4	0.00
5-9	0.00
10-14	0.00
15-19	0.00
20-24	10.00
25-29	0.00
30-34	0.00
35-39	0.00

40-44	10.00
45-49	0.00
50-54	10.00
55-59	20.00
60-64	10.00
65-69	0.00
70-74	20.00
75-79	20.00
80-84	0.00
>85	0.00
All ages	100.00

Table 5. Duration of symptomatic disease by age group

Age	%
0-9	0.019–0.038
$\geq$ 10	0.082 (0.038–0.5)

References

Allen CJ, Ferson MJ. Notification of infectious diseases by general practitioners: a quantitative and qualitative study. Med J Aust 2000, 172:325- 328.

Arteaga-Rodriguez A, Carrasco-Garrido P, de Andres AL, de Miguel AG, Santos J, Jimenez-Garcia R. Changes in the epidemiology of hepatitis A in Spain (2005-2008): trends of acute hepatitis A hospitalizations, comorbidities, and costs associated with the hospitalization. Eur J Gastroenterol Hepatol 2010, 22:1284-1289.

Bauch CT, Anonychuk AM, Pham BZ, Gilca V, Duval B, Krahn MD. Cost-utility of universal hepatitis A vaccination in Canada. Vaccine 2007, 25:8536-8548.

Centers for Disease Control and Prevention. Epidemiology and Prevention of Vaccine-Preventable Diseases. Atkinson W, Wolfe S, Hamborsky J, eds. 12th ed. Washington DC: Public Health Foundation, 2012.

Ciocca M. Clinical course and consequences of hepatitis A infection. Vaccine 2000, 18 Suppl 1:S71-74.

Cuthbert JA: Hepatitis A: old and new. Clin Microbiol Rev 2001, 14:38-58.

Fiore AE. Hepatitis A transmitted by food. Clin Infect Dis 2004, 38:705-715.

Gingrich GA, Hadler SC, Elder HA, Ash KO. Serologic investigation of an outbreak of hepatitis A in a rural day-care center. Am J Public Health 1983, 73:1190-1193.

Haagsma JA, Janssen MF, Bonsel GJ. Comparing generic and disease specific health state valuations by a laymen panel. In EuroQol proceedings; Oslo, Norway. 2005

Haagsma JA, van der Zanden BP, Tariq L, van Pelt W, van Duynhoven YTHP, Havelaar AH. Disease burden and costs of selected foodborne pathogens in the Netherlands, 2006. vol. RIVM rapport 330331001. Bilthoven: National Institute of Public Health and Environment; 2009.

Haagsma JA, Maertens de Noordhout C, Polinder S, Vos T, Havelaar AH, Cassini A, Devleesschauwer B, Kretzschmar ME, Speybroeck N, Salomon JA. Assessing disability weights based on the responses of 30,660 people from four European countries. Population Health Metrics 2015; 13: 10

Havelaar AH, Haagsma J, Mangen MJJ, Kemmeren JM, Verhoef L, Vijgen SMC, et al. Disease burden of foodborne pathogens in the Netherlands, 2009. International Journal of Food Microbiology 2012 156:231-238.

Havelaar AH, Melse JM. Quantifying public health risks in the WHO Guidelines for Drinking-Water Quality. Bilthoven, the Netherlands: National Institute for Public Health and the Environment; 2003.

Jacobs RJ, Gibson GA, Meyerhoff AS: Cost-effectiveness of hepatitis A-B vaccine versus hepatitis B vaccine for healthcare and public safety workers in the western United States. Infect Control Hosp Epidemiol 2004, 25:563-569.

Jacobs RJ, Margolis HS, Coleman PJ. The cost-effectiveness of adolescent hepatitis A vaccination in states with the highest disease rates. Arch Pediatr Adolesc Med 2000, 154:763-770.

Jeong SH, Lee HS. Hepatitis A: clinical manifestations and management. Intervirology 2010, 53:15-19.

Koff RS. Clinical manifestations and diagnosis of hepatitis A virus infection. Vaccine 1992, 10 Suppl 1:S15-17.

Matin N, Grant A, Granerod J, Crowcroft N. Hepatitis A surveillance in England--how many cases are not reported and does it really matter? Epidemiol Infect 2006, 134:1299-1302.

Mead PS, Slutsker L, Dietz V, McCaig LF, Bresee JS, Shapiro C, et al. Food-related illness and death in the United States. Emerg Infect Dis 1999, 5:607-625.

Van de Laar MJW, Wijgergangs LM, Rijlaarsdam J, de Wit A. Vaccineren tegen hepatitis A: een verkenning van kosten. Infectieziektebulletin 2000, 11:262-266.

WHO. Global Burden of Disease 2004 Update: Disability weights for diseases and conditions. Geneva: WHO; 2004.

Hepatitis B

Hepatitis B is caused by the hepatitis B virus (HBV) which affects the liver and can cause both acute and chronic infections. Many patients present no symptoms during the initial infection.

The following estimates have been calculated for the proportion of infected individuals who develop symptoms:

- 30–50% of those adults infected develop acute icteric hepatitis (McMahon et al. 1985)

- Over 90 percent of perinatal HBV infections are asymptomatic, while the typical manifestations of acute hepatitis are noted in 5–15 percent of newly-infected young children (1–5 years of age) and in 33–50 percent of older children, adolescents, and adults (Shepard et al. 2006).

We therefore assumed that the range for the symptomatic proportion of new infections was age-dependent (see Table 1). The duration of acute illness has been estimated at six weeks (Kwong, 2012).

Chronicity rate

There is much evidence of age-related variation in the development rate for chronic HBV infection after acute infection. For example:

- The likelihood of developing chronic HBV infection is higher in individuals infected perinatally (90%) or during childhood (20–30%), when the immune system is thought to be immature, compared with immunocompetent subjects infected during adulthood (<1%)' (Fattovich, 2008)

- The overall chronicity rate for HBV has been estimated at 5–10%, although it is higher in those who were infected perinatally (90%) or during childhood (20%) (Yim & Lok, 2005)

- More than 90% of infected infants, 25–50% of children infected between and 5 years, and 6–10% of acutely infected older children and adults develop chronic infection (Shepard et al. 2006)

- About 30% of children aged 1–5 years and 5% of adults develop chronic hepatitis B infection (Pungpadong et al. 2007).

- Nearly all persons infected perinatally and up to 50% of children infected between the ages of 1–5 years develop chronic hepatitis (NIH, 2008)

- 5% of adults with acute infection develop chronic hepatitis B (Wilt et al. 2008)

- 5-10% of adult patients do not clear the virus and either progress to become asymptomatic carriers or develop chronic hepatitis (WHO 2002)

- The chronicity rate is approximately 90% for infants in the first year of life, 30% for children infected between the ages of 1 and 4 years and <5% for healthy adults (Edmunds et al. 1993).

In the model, we adopted the age-dependent chronicity rates reported above by Fattovich et al. presented in the results of a systematic review of the literature (2008).

The duration of the chronic carrier stage varies according to the presence or absence of active viral replication, estimated at 4.5 years in the case of active viral replication and 33.24 years in the case of no active replication (Stouthard, 1997). Information on the proportion of chronic hepatitis cases with active viral replication to those without active replication is not available and we chose to set the duration as uncertain, between 4.5 and 33.24 years.

Risk of complications

Fulminant liver failure

Fulminant liver failure occurs in approximately 0.5 to 1.0% of adults with reported acute hepatitis B but rarely in infants and children (Pappas, 1995; Hoofnagle et al. 1995). In the model we specified a range (0.5–1.0%) for this transition probability for all age groups as we were unable to locate specific values for infants and children. However, we modelled the age-specific probability of the case fatality rate based on the observed rates, hence a zero probability of children dying of acute hepatitis (see Table 5).

The case fatality rate (CFR) among patients who develop fulminant liver failure is approximately 20–33% (Bernua et al. 1986; Wai et al. 2005) and this figure was chosen for our model. There were no recent specific European studies stating the frequency and impact of orthotopic liver transplantation (OLT) (Steinmuller et al. 2002) and new antiviral medications (Eisenbach, 2006).

The duration of fulminant liver failure, estimated based on the time from onset of symptoms to encephalopathy, is one to 56 days (Trey and Davidson 1970).

Compensated cirrhosis (CC)

According to Chu (2000), on average, 2.1% of people with chronic HBV infection develop compensated cirrhosis annually. This does not take into account variations due to other effects such as alcohol consumption, diabetes and obesity (in the BCoDE toolkit the yearly rate refers to an Annual Transition Probability, ATP, as opposed to the Lifetime Transition Probability, LTP). However, it is important to consider that individuals who have a severe acute exacerbation complicated by subacute hepatic failure or who have recurrent episodes of acute exacerbations with bridging hepatic necrosis are more likely to develop cirrhosis (Chu, 2000)

Decompensated cirrhosis (DC)

According to a systematic review undertaken by D'Amico et al. (2006. The review undertaken by Fattovich et al. (2008) estimated an annual probability of 3–4% for Europe which we chose for our model.

The 20–57% case fatality rate for DC was estimated based on the review by D`Amico et al. (20% from the first of two DC stages, characterised by ascites with or without non-bleeding esophageal varices; 57% from the second of two DC stages, characterised by bleeding varices, with or without ascites).

The duration of DC is based on the average waiting time for liver transplants in EU countries which publish their data online (UK and Spain): between 124 and 142 days (NHS, 2014; Matesanz 2009).

Hepatocellular carcinoma (HCC)

The annual rate of developing HCC is 0.1% in asymptomatic HBsAg individuals, and between 0.3 and 1% in patients with chronic hepatitis B, but this rate increases to 2–10% in patients with compensated cirrhosis (Fattovich, 2008; Yim & Lok, 2005; Pungpadong, 2007; Chu, 2000; D'Amico, 2006). Chu and Liaw (2006) and Fattovich (2008) estimated the CC to HCC transition probability to range between 1.5 and 2.2%/year for Europe.

For the model, we adopted Fattovich`s (2008) estimate stemming from an extensive systematic literature review of 0.3% (0.12–0.41) per year to develop HCC from chronic hepatitis B infection and 2.2% (1.71–2.71) per year for the development of HCC from compensated cirrhosis.

In a European setting, Shepherd`s (2006) cost-effectiveness analysis set the annual case fatality rate for HCC to 56%, while Kanwal (2005) set it to 43.3% (20–60). We chose the latter range for our model as it includes Shepherd`s assumption.

Model input summary

Table 1. Transition probabilities and distributions used in the outcome tree

Health outcome<br><br>(Health state)	Distribution of health states within health outcome	Transition probability	Source/assumption
Symptomatic infection		10–50%<br>See Table 3	Age-dependent<br>McMahon, 1985; Shepard, 2006
Chronic hepatitis		1–90%<br>See Table 4	Age-dependent<br>Fattovich, 2008
Fulminant liver failure		0.5–1%	Pappas, 1995; Hoofnagle et al. 1995
Fatal cases due to liver failure		20-33.3%<br>See Table 5	Bernau et al. 1986 ; Wait et al. 2005<br>Assuming different age-specific probabilities based on observed mortality

Compensated cirrhosis		2.1%/year	Chu, 2000 (ATP)
Decompensated cirrhosis		3-4%/year	Fattovich, 2008 (ATP)
HCC, following<br>- Chronic hepatitis<br>- Compensated cirrhosis		0.3% (0.12–0.41)/year<br>2.2% (1.71–2.71)/year	Fattovich, 2008 (ATP<br>Fattovich, 2008 (ATP
CFR, following:<br>- DC<br>- HCC		20-57%/year<br>43.3% (20-60)/year	D'Amico, 2006 (ATP)<br>Kanwal, 2005 (ATP)

Table 2. Disability weights and duration

Health outcome (Health state)	Disability Weight (DW) (Haagsma, 2015)		Duration	
	DW	Source: ECDC European Disability Weight Project (2014)	In years	Source/assumption
Symptomatic infection	0.051 (0.039–0.06)	Infectious disease, acute episode, moderate	0.115	Kwong 2012
Fulminant liver failure	0.515 (0.459–0.572)	Terminal phase, with medication (for cancers, end-stage kidney/liver disease)	0.003–0.153	Trey, 1970
Chronic hepatitis	0.07 (0.057–0.088)	Generic, uncomplicated disease: worry and daily medication	4.5–33.24	Stouthard, 1997

				Assuming uncertainty between proportion with active replication and without
Compensated cirrhosis	0.07 (0.057–0.088)	Generic uncomplicated disease: worry and daily medication	6-10.4<br><br>See Table 6	Murray, 1996<br><br>Age and gender specific
Decompensated cirrhosis	0.163 (0.136–0.194)	Decompensated cirrhosis of the liver	0.34–039	Assuming average waiting time before liver transplantation in the UK and Spain (NHS and Matesanz 2009)
Hepatocellular carcinoma	0.265 (0.222–0.303)	Cancer, diagnosis and primary therapy	0.72–4.48<br><br>See Table 7	Murray, 1996<br><br>Age and gender specific

Table 3. Hepatitis B infected developing symptoms

Age group	Symptomatic hepatitis B
0	10%
1–4	5–15%
5–80+	30–50%

Table 4. Hepatitis B infected developing chronic hepatitis

Age group	Chronic hepatitis B
0	90%
1–4	20–30%
5–80+	1%

Table 5. CFR age distribution for acute hepatitis observed in Estonia, Germany and the Netherlands 2005–2007

Age groups	CFR
0	0.00

1-4	0.00
5-9	0.10
10-14	0.00
15-19	0.00
20-24	0.14
25-29	0.30
30-34	0.53
35-39	1.27
40-44	1.75
45-49	4.56
50-54	5.81
55-59	5.83
60-64	7.90
65-69	11.86
70-74	11.97
75-79	19.77
80-84	15.67
>85	12.54
All ages	100

Table 6. Duration of compensated cirrhosis

	F	M
0–4	10.4	10.3
5–14	10.4	10.4
15–44	10.2	10
45–59	9.3	8.8
60+	6.5	6

Table 7. Duration of HCC

Age group	Duration (years)	
	F	M
0–14	4.48	4.11
15–44	1.45	2.92
45–59	1.91	2.88
60+	0.72	1.56

References

Alter MJ, Mares A, Hadler SC, Maynard JE. The effect of underreporting on the apparent incidence and epidemiology of acute viral hepatitis. Am J Epidemiol. 1987;125(1):133-9.

Bernuau J, Goudeau A, Poynard T. Multivariate analysis of prognostic factors in fulminant hepatitis B. Hepatology, vol.6, no. 4, pp. 648–651, 1986

Bernuau J, Rueff B, Benhamou JP. Fulminant and subfulminant liver failure: definitions and causes. Seminars in Liver Disease, vol. 6, no. 2, pp. 97–106, 1986

Christensen PB, et al. Outbreak of Hepatitis B among injecting drug users in Denmark. J Clinical Virology 2001;22(1):133-141.

Chu CM. (2000), Natural history of chronic hepatitis B virus infection in adults with emphasis on the occurrence of cirrhosis and hepatocellular carcinoma. Journal of Gastroenterology and Hepatology, 15: E25–E30.

D'Amico G, Garcia-Tsao G, Pagliaro L. Natural history and prognostic indicators of survival in cirrhosis: a systematic review of 118 studies. Journal of Hepatology 2006;44(1):217-31.

Edmunds WJ, Medley GF, Nokes DJ, Hall AJ, Whittle HC. The influence of age on the development of the hepatitis B carrier state. Proceedings Biological Sciences, 1993;253(1337):197-201.

Elhouhari HM, Tamimi TIAR, Carey WD. Hepatitis B virus infection: Understanding its epidemiology, course, and diagnosis. Cleveland Clinical Journal of Medicine 2008;75:881-889.

Eisenbach C, Sauer P, Mehrabi A, Stremmel W, Encke J. Prevention of hepatitis B virus recurrence after liver transplantation. Clin Transplant 2006; 20 Suppl 17: 111–116

Fattovich G, Bortolotti F, Donato F. Natural history of chronic hepatitis B: special emphasis on disease progression and prognostic factors. [Review] [143 refs]. Journal of Hepatology. 2008 20071204 [Epub ahead of print];48(2):335-52.

Garfein RS, Vlahov D, Galai N, et al. Viral infections in short-term injection drug users: the prevalence of the hepatitis C, hepatitis B, human immunodeficiency, and human T-lymphotropic viruses. Am J Public Health 1996;86:655–661.

Greten TF, Papendorf F, Bleck JS, Kirchhoff T, Wohlberedt T, Kubicka S, et al. Survival rate in patients with hepatocellular carcinoma: a retrospective analysis of 389 patients. British Journal of Cancer, 2005 May 23;92(10):1862-8.

Haagsma JA, Maertens de Noordhout C, Polinder S, Vos T, Havelaar AH, Cassini A, Devleesschauwer B, Kretzschmar ME, Speybroeck N, Salomon JA. Assessing disability weights based on the responses of 30,660 people from four European countries. Population Health Metrics 2015; 13: 10

Hoofnagle JH, Carithers RL Jr, Shapiro C, Ascher N. Fulminant hepatic failure: summary of a workshop. Hepatology 1995;21:240-252.

Hoofnagle JH, Doo E, Liang TJ, Fleischer R, Lok ASF. Management of Hepatitis B: Summary of a Clinical Research Workshop. Hepatology 2007;45:1056-1075.

Kanwal F, Gralnek IM, Martin P et al. Treatment alternatives for chronic hepatitis B viral infection: a cost-effectiveness analysis. Annals of Internal Medicine. 2005;142:821-31.

Kim WR, Brown RS, Terrault NA, El-Serag H. Burden of liver disease in the United States: summary of a workshop. Hepatology 2002; 36: 227-242

Kwong JC, Ratnasingham S, Campitelli MA, Daneman N, Deeks SL, et al. (2012) The Impact of Infection on Population Health: Results of the Ontario Burden of Infectious Diseases Study. PLoS ONE 7(9): e44103. doi:10.1371/journal.pone.0044103

Levine OS, Vlahov D, Nelson KE. Epidemiology of hepatitis B virus infections among injecting drug users: seroprevalence, risk factors, and viral interactions. Epidemiol Rev 1994;16:418–36

Levine OS, Vlahov D, Koehler J, et al. Seroepidemiology of hepatitis B virus in a population of injecting drug users. Association with drug injection patterns. Am J Epidemiol 1995;142:331–41.

Liaw YF (2009) Natural history of chronic hepatitis B virus infection and long-term outcome under treatment. Liver international Vol. 29; pp 100- 107.

Lok ASF, McMahon (2007) Chronic Hepatitis B. Hepatology Vol. 45; pp 507 - 539

Matesanzemail R, de la Rosa G. Liver transplantation: The Spanish experience. Digestive and Liver Disease Supplements. Volume 3, Issue 4 , Pages 75-81, December 2009.

McMahon BJ, Alward WL, Hall DB, Heyward WL, Bender TR, Francis DP, et al. Acute hepatitis B virus infection: relation of age to the clinical expression of disease and subsequent development of the carrier state. The Journal of Infectious Diseases, 1985 Apr;151(4):599-603.

McMahon BJ (2005). Epidemiology and natural history of Hepatitis B. Seminars in liver disease. Vol. 25. Supplement 1.

Murray CJL, Lopez AD. World Health Organization, World Bank, Harvard School of Public Health. Global health statistics: a compendium of incidence, prevalence and mortality estimates for over 200 conditions. Geneva: World Health Organization; 1996.

Murray CJL, Lopez AD. The Global Burden of Disease - A comprehensive assessment of mortality and disability from diseases, injuries, and risk factors in 1990 and projected to 2020. Cambridge: Harvard School of Public Health on behalf of the World Health Organization and the World Bank; 1996.

National Health System (NHS), UK. h p://www.nhs.uk/condi̭ons/Liver-transplant/Pages/Introduc̭on.aspx. Website visited on 31 July 2014

National Institutes of Health (2008) NIH Consensus Development Conference Statement on Management of Hepatitis B. NIH Consensus and State-of-the-Science Statements Volume 25, Number 2. October 20–22, 2008

Pungpapong S, Kim WR, Poterucha JJ. Natural history of hepatitis B virus infection: an update for clinicians. Mayo Clin Proc 2007; 82:967–975.

Shepard CW, Simard EP, Finelli L, Fiore AE, Bell BP (2006) Hepatitis B virus infection: Epidemiology and vaccination. Epidemiological Reviews; Vol. 28; pp 112-125

Shepherd J, Jones J, Takeda A et al. Adefovir dipivoxil and pegylated interferon alfa-2a for the treatment of chronic hepatitis B: a systematic review and economic evaluation. [Review] [136 refs]. Health Technology Assessment (Winchester, England). 2006;10(28):iii-iiv.

Stouthard M , Essink-Bot M, Bonsel G, Barendregt J, Kramers P. 1997. Disability weights for diseases in the Netherlands. Rotterdam, Department of Public Health, Erasmus University.

Trey C, Davidson CS. The management of fulminant hepatic failure. In: Popper H, Schaffner F, eds. Progress in liver disease. Volume 3. New York: Grune & Stratton, 1970:282-98

Wai CT, Fontana RJ, Polson J, Hussain M, Shakil AO, Han SH, et al. Clinical outcome and virological characteristics of hepatitis B-related acute liver failure in the United States. J Viral Hepat 2005; 12: 192-198

Wilt TJ, Shamliyan T, Shaukat A, Taylor BC, MacDonald R, Yuan J-M, et al. (2008) . Management of Chronic Hepatitis B. Evidence Report/Technology Assessment No. 174. (Prepared by the Minnesota Evidence-based Practice Center under Contract No. 290-02-0009.) AHRQ Publication No. 09-E002. Rockville, MD. Agency for Healthcare Research and Quality. October 2008.

WHO (2002) Hepatitis B; WHO/CDS/CSR/LYO/2002.2:Hepatitis B

WHO (2001) Introduction of Hepatitis B vaccine into childhood immunization services

Yim HJ, Lok ASF. Natural history of chronic hepatitis B virus infection: what we knew in 1981 and what we know in 2005. Hepatology 2006; 43:pp 173–S181.

Hepatitis C

A total of 20–30% of newly infected individuals develop clinical symptoms of acute hepatitis (e.g. anorexia, abdominal discomfort, nausea, vomiting and jaundice) within 2–24 weeks of exposure (CDC, 2011; Wasmuth, 2010; World Health Organization, 2002). In persons who do develop symptoms of acute hepatitis, the illness lasts between two and 12 weeks (Wasmuth, 2010).

In the model, it was assumed that 20–30% of newly infected individuals develop clinical symptoms of acute hepatitis (CDC, 2011).

Rate of developing chronic hepatitis C

Acute hepatitis C develops into chronic infection in 75.6% (67.3–84.9) of all symptomatic and asymptomatic cases over 20 years old, with the infection resolving in the remaining proportion (Alter & Seef, 1994). The chronicity rate is known to be lower in younger individuals. A recent review of the literature by Alter et al. (2000), has estimated that the rate of spontaneous recovery is 29–45% in those aged under 20 years and this was used for the disease model (chronicity rate: 55–69%).

In the early stages of chronic infection there is a small chance of spontaneous remission. The rate of remission of chronic hepatitis C was set at 0.31 (0.26–0.36)% per year in accordance with the findings of Micallef et al. (2006) (in the Burden of Communicable Diseases in Europe toolkit a yearly rate refers to an Annual Transition Probability, ATP, as opposed to the Lifetime Transition Probability, LTP).

In the absence of spontaneous remission or successful antiviral therapy, chronic infections may progress from mild to moderate hepatitis to liver cirrhosis, with a risk of developing life-threatening sequelae such as decompensated liver disease and hepatocellular carcinoma. Progression to severe liver disease can take 20–40 years. However, progression, which is non-linear, is strongly influenced by cofactors including alcohol intake, HIV or HBV coinfection, gender (male) and an older age at infection (Alberti, 2005; Alter & Seeff, 2000; Freeman, 2003; Lauer & Walker, 2001; Poynard, 2001; Thein, 2008).

Given emerging knowledge of the disease, the most appropriate approach to simulating the progression from chronic infection to cirrhosis would be to specify a model with five health stages, representing the METAVIR fibrosis stages F0–F4, linked by multivariate risk functions. A further possibility could be to represent mild and moderate pre-cirrhotic disease stages. However, for the sake of simplicity and in the context of a burden of disease study in which the objective is to compare a broad spectrum of diseases, a single, chronic hepatitis health outcome was applied.

Risk of complications

Compensated cirrhosis (CC)

The risk of HCV-infected persons developing cirrhosis within 20–30 years is estimated in most studies to be within the range of between 5 and 20%, although some studies give estimates of up to 50% (CDC, 2011; Freeman 2001; Freeman 2003; Lauer & Walker, 2001; Poynard, 1997; Poynard, 2001; Thein, 2008; Wasmuth, 2010). Thein et al. predicted via meta-analysis an average 20-year cirrhosis risk of 16% (95% CI: 14%-19%), and a 30-year risk of 41% (95% CI: 36%–45%), which underlines that the progression to cirrhosis is not a linear process (Thein et al. 2008).

The annual risk of progressing to compensated cirrhosis was calculated based on the transitional probabilities between the five METAVIR stages of fibrosis, as estimated by Thein et al. (2008), using random-effect meta-analysis applied to non-clinical studies only. The point estimate for the risk of developing compensated cirrhosis from chronic hepatitis, calculated as the inverse of the summed durations in the first four METAVIR stages (each duration in turn was estimated as 1/probability of leaving the METAVIR stage), was 1.9% per year. The disability duration was calculated at 36.5 years; this is the average time taken for 50% of those with chronic hepatitis to exit the compartment: $1- \exp(-0.019 * 36.5) = 0.5$.

Decompensated cirrhosis (DC)

HCV-associated cirrhosis leads to liver failure and death in about 20–25% of cirrhotic cases. The annual risk of compensated cirrhosis progressing to the decompensated stage (characterised by ascites, bleeding oesophagal varices, or jaundice) is estimated to be 3.9–7% (D'Amico, 2006; Fattovich, 1997; Grieve, 2006; Poynard, 1997; Wasmuth, 2010). In the model, hepatic decompensation was assumed to occur with an annual risk of 3.9 to 12.9 (Dienstag, 2011).

Without transplantation the prognosis is poor. The five-year survival rate with decompensated liver cirrhosis is roughly 50% (Planas, 2004). One report based on a small study population (n=65) estimated the annual mortality rate at 12.9% (Fattovich et al. 1997), but higher values were reported in the systematic review by D'Amico et al. (2006) (20% 1-year mortality from the first stage of DC; 55% from the second DC stage, which is indicated by bleeding varices with or without ascites). The estimated annual risk of death from DC was set to within a range of 13– 38.5% (Fattovich, 1997; Grieve, 2006; D'Amico, 2006); the upper bound was calculated as the mean of the rates for the two DC stages reported by D'Amico et al (2006).

Duration of DC is based on average waiting time for liver transplant in the UK and in Spain which are represented as an average duration (142 days, NHS and 124 days, Matesanz 2009).

Hepatocellular carcinoma (HCC)

In contrast to hepatitis B, development of primary liver cancer, or hepatocellular carcinoma (HCC), is rare in patients with chronic hepatitis C who do not have cirrhosis (Lauer & Walker, 2001; Spengler, 2010; Wasmuth, 2010; WHO, 2002). Once cirrhosis is established, the risk of hepatocellular carcinoma is estimated to be 1–4% per year (Fattovich et al. 1997; Lauer & Walker, 2001). Studies modelling the natural course of hepatitis C have assumed annual risks of around 1.5% (Grieve et al. 2006; Siebert et al. 2003).

HCC is an outcome that can occur after either the compensated or decompensated cirrhosis stages. The annual risk of developing HCC following either CC or DC was set to 3%, based on the estimate by D'Amico et al. (2006).

Studies modelling the natural course of hepatitis C have assumed annual case fatality rates (CFR) due to liver cancer ranging widely from 43– 86% (Grieve, 2006; Siebert, 2003; Wong, 2000). This variation might be a consequence of stage and treatment-specific survival rates, and other underlying conditions including alcohol consumption, diabetes or obesity, where the higher estimate is used to simulate a situation without early diagnosis and effective treatment. In the model, this CFR is set to 48.9%/year, based on the 1-year survival rate (Kwong, 2012).

Other complications

Fulminant hepatic failure due to acute HCV infection is considered to be very rare (CDC, 2011; Lauer & Walker, 2001; Wasmuth, 2010; World Health Organization, 2002) except in cases of HBV coinfection (Chu, 1999). Fulminant liver failure and death was reported to occur in approximately 0.1% (2/1536) of adults with reported (notified) acute hepatitis C (Bianco, 2003). Due to this condition being extremely rare, no health outcome was specified in the outcome tree.

Model input summary

Table 1. Transition probabilities and distributions used in the outcome tree

Health outcome (Health state)	Distribution of health states in health outcome	Transition probability	Source/assumption
Symptomatic infection	20–30%		CDC, 2011
Chronic hepatitis		> 19yr: 75.6% (67.3–84.9)<br>< 20yr: 55–69%	Alter, 1994; Alter, 2000<br>Age dependent
Remission from chronic hepatitis		0.31 (0.26–0.36)%/year	Micallef, 2006 (ATP)
Compensated cirrhosis		1.9%/year	Modelled from Thein, 2008 (ATP)
Decompensated cirrhosis		3.9–12.9%/year	Dienstag, 2011 (ATP)
HCC, following<br>- Compensated cirrhosis<br>- Decompensated cirrhosis		3.0%/year<br>3.0%/year	D'Amico, 2006 (ATP)<br>D'Amico, 2006 (ATP)
CFR, following:<br>- Decompensated cirrhosis<br>- Hepatocellular carcinoma		13–38.5%/year<br>48.9%/year	Fattovich, 1997; Grieve 2006;<br>D’Amico 2006 (ATP)<br>Kwong, 2012 (ATP)

Table 2. Disability weights and duration

Health outcome (Health state)	Disability Weight (DW) (Haagsma, 2015)		Duration	
	DW	Label	In years	Source
Symptomatic infection	0.051 (0.039–0.06)	Infectious disease, acute episode, moderate	0.038–0.23	CDC, 2011; Wasmuth, 2010; World Health Organization, 2002
Chronic hepatitis	0.07 (0.057–0.088)	Generic uncomplicated disease: worry and daily medication	36.5	Modelled from Thein, 2008
Compensated cirrhosis	0.07 (0.057–0.088)	Generic uncomplicated disease: worry and daily medication	6-10.4<br>See table 3	Murray, 1996<br>Age and gender specific
Decompensated cirrhosis	0.163 (0.136–0.194)	Decompensated cirrhosis of the liver	0.34–039	Assuming average waiting time before liver transplantation in UK and Spain (NHS and Matesanz 2009)
Hepatocellular carcinoma	0.265 (0.222–0.303)	Cancer, diagnosis and primary therapy	0.72–4.48<br>See table 4	Murray, 1996<br>Age and gender specific

Table 3. Duration of compensated cirrhosis

Age group	Duration (years)	
	F	M

0-4	10.4	10.3
5-14	10.4	10.4
15-44	10.2	10
45-59	9.3	8.8
60+	6.5	6

Table 4. Duration of hepatocellular carcinoma

Age group	Duration (years)	
	F	M
0-14	4.48	4.11
15-44	1.45	2.92
45-59	1.91	2.88
60+	0.72	1.56

References

Alberti A, Vario A, Ferrari A, Pistis R. (2005). Review article: chronic hepatitis C--natural history and cofactors. Aliment Pharmacol Ther 22 Suppl 2, 74-78.

Alter MJ, Kruszon-Moran D, Nainan OV, McQuillan GM, Gao F, Moyer LA, et al. The prevalence of hepatitis C virus infection in the United States, 1988 through 1994. Engl J Med. 1999 Aug 19; 341(8):556-62.

Alter HJ, Seeff LB. (2000). Recovery, persistence, and sequelae in hepatitis C virus infection: a perspective on long-term outcome. Semin Liver Dis 20, 17-35.

Bianco E, Stroffolini T, Spada E, Szklo A, Marzolini F, Ragni P, et al. (2003). Case fatality rate of acute viral hepatitis in Italy: 1995-2000. An update. Dig Liver Dis 35, 404-408.

Centers for Disease Control and Prevention (CDC). Hepatitis C: Information for Health Professionals. 2011. Available at: http://www.cdc.gov/hepatitis/HCV/index.htm.

Chu CM, Yeh CT, Liaw YF. Fulminant hepatic failure in acute hepatitis C: increased risk in chronic carriers of hepatitis B virus. Gut 1999;45:613- 617.

Crofts N, Jolley D, Kaldor J, et al. Epidemiology of hepatitis C virus infection among injecting drug users in Australia. J Epidemiol Community Health 1997;1:692–697.

D'Amico G, Garcia-Tsao G, Pagliaro L. Natural history and prognostic indicators of survival in cirrhosis: a systematic review of 118 studies. J Hepatol. 2006;44(1):217-31.

Dienstag JL, Ghany MG, Morgan TR, Di Bisceglie AM, Bonkovsky HL, Kim HY, et al. and the HALT-C Trial Group: A prospective study of the rate of progression in compensated, histologically advanced chronic hepatitis C. Hepatology 2011, 54:396–405

ECDC. Surveillance and prevention of hepatitis B and C in Europe. Stockholm: ECDC 2010.

Fattovich G, Giustina G, Degos F, Tremolada F, Diodati G, Almasio P, et al. (1997). Morbidity and mortality in compensated cirrhosis type C: a retrospective follow-up study of 384 patients. Gastroenterology 112, 463-472.

Freeman AJ, Dore GJ, Law MG, Thorpe M, Von Overbeck J, Lloyd AR et al. Estimating progression to cirrhosis in chronic hepatitis C virus infection. Hepatology 2001;34:809-816.

Freeman AJ, Law MG, Kaldor JM, and Dore GJ. Predicting progression to cirrhosis in chronic hepatitis C virus infection. J Viral Hepat 2003;10:285-293.

Grieve R, Roberts J, Wright M, Sweeting M, DeAngelis D, Rosenberg W, et al. Cost effectiveness of interferon alpha or peginterferon alpha with ribavirin for histologically mild chronic hepatitis C. Gut 2006;55:1332-1338.

Hagan H, Snyder N, Hough E, Yu T, McKeirnan S, Boase J, et al. Case-reporting of acute hepatitis B and C among injection drug users. J Urban Health 2002;79(4):579-85.

Haagsma JA, Maertens de Noordhout C, Polinder S, Vos T, Havelaar AH, Cassini A, Devleesschauwer B, Kretzschmar ME, Speybroeck N, Salomon JA. Assessing disability weights based on the responses of 30,660 people from four European countries. Population Health Metrics 2015; 13: 10

Kwong JC, Ratnasingham S, Campitelli MA, Daneman N, Deeks SL, et al. (2012) The Impact of Infection on Population Health: Results of the Ontario Burden of Infectious Diseases Study. PLoS ONE 7(9): e44103. doi:10.1371/journal.pone.0044103

Lauer GM, Walker BD. Hepatitis C virus infection. N Engl J Med. 2001;345:41-52.

Mann AG, Ramsay ME, Brant LJ, Balogun MA, Costella A, Harris HE. Diagnoses of, and deaths from, severe liver disease due to hepatitis C in England between 2000 and 2005 estimated using multiple data sources. Epidemiol Infect. 2009;137(4):513-8.

Matesanzemail R, de la Rosa G. Liver transplantation: The Spanish experience. Digestive and Liver Disease Supplements. Volume 3, Issue 4 , Pages 75-81, December 2009.

McDonald SA, Hutchinson SJ, Mills PR, et al. Diagnosis of hepatitis C virus infection in Scotland's injecting drug user population. Epidemiol. Infect 2010;138:393–402.

Micallef JM, Kaldor JM, Dore GJ (2006) Spontaneous viral clearance following acute hepatitis C infection: a systematic review of longitudinal studies. J Viral Hepat 13: 34–41.

Murray CJL, Lopez AD, World Health Organization., World Bank., Harvard School of Public Health.: Global health statistics: a compendium of incidence, prevalence and mortality estimates for over 200 conditions. Geneva: World Health Organization; 1996.

Murray CJL, Lopez AD: The Global Burden of Disease - A comprehensive assessment of mortality and disability from diseases, injuries, and risk factors in 1990 and projected to 2020. Cambridge: Harvard School of Public Health on behalf of the World Health Organization and the World Bank; 1996.

National Health System (NHS), UK. Available at: http://www.nhs.uk/conditions/Liver-transplant/Pages/Introduction.aspx(accessed 31 July 2014)

Okuda K, Ohtsuki T, Obata H, Tomimatsu M, Okazaki N, Hasegawa H. et al. (1985). Natural history of hepatocellular carcinoma and prognosis in relation to treatment. Study of 850 patients. Cancer 1985;56:918-928.

Planas R, Balleste B, Alvarez MA, Rivera M, Montoliu S, Galeras JA, et al. Natural history of decompensated hepatitis C virus-related cirrhosis. A study of 200 patients. J Hepatol. 2004;40823-830.

Poynard T, Bedossa P, Opolon P. Natural history of liver fibrosis progression in patients with chronic hepatitis C. The OBSVIRC, METAVIR, CLINIVIR, and DOSVIRC groups. Lancet 1997;349:825-832.

Poynard T, Ratziu V, Charlotte F, Goodman Z, McHutchison J, Albrecht J. Rates and risk factors of liver fibrosis progression in patients with chronic hepatitis c. J Hepatol. 2001;34:730-739.

Siebert U, Sroczynski G, Rossol S, Wasem J, Ravens-Sieberer U, Kurth BM, et al. (2003). Cost effectiveness of peginterferon alpha-2b plus ribavirin versus interferon alpha-2b plus ribavirin for initial treatment of chronic hepatitis C. Gut 52, 425-432.

Spengler U. (2010). Hepatocellular Carcinoma - Diagnosis, Prognosis & Therapy. In Hepatology - A Clinical Textbook. S. Mauss, T. Berg, J. Rockstroh, C. Sarrazin, and H. Wedemeyer, eds. (Düsseldorf, Flying Publisher).

Strauss R, Fülöp G, Pfeifer C. Hepatitis C in Austria 1993–2000: reporting bias distort HCV epidemiology in Austria. Euro Surveill. 2003;8(5):pii=412.

Thein HH, Yi Q, Dore GJ, Krahn MD. (2008). Estimation of stage-specific fibrosis progression rates in chronic hepatitis C virus infection: a meta- analysis and meta-regression. Hepatology 48, 418-431.

Wasmuth, J. (2010). Hepatitis C - Epidemiology, transmission and natural history. In Hepatology - A Clinical Textbook. S. Mauss, T. Berg, J. Rockstroh, C. Sarrazin, and H. Wedemeyer, eds. (Düsseldorf, Flying Publisher).

Wong JB, McQuillan GM, McHutchison JG, Poynard T. (2000). Estimating future hepatitis C morbidity, mortality, and costs in the United States. Am J Public Health 90, 1562-1569.

World Health Organization (2002). Hepatitis C. Available at: http://www.who.int/csr/disease/hepatitis/whocdscsrlyo2003/en/index.html. Accessed: February 2011.

Yousuf M, Nakano Y, Tanaka E, Sodeyama T, Kiyosawa K. Persistence of viremia in patients with type-C chronic hepatitis during long-term follow-up. Scand J Gastroenterol. 1992;27:812-816.

HIV

Acquired Immunodeficiency Syndrome (AIDS) is the most severe outcome of an untreated HIV infection. AIDS presents with severe opportunistic infections, malignancies, neurological complications or other HIV-induced disease conditions (Del Rio & Curran, 2005). After infection with HIV, individuals may remain asymptomatic or develop Acute Retroviral Syndrome (ARS) (Del Rio & Curran, 2005). ARS occurs in 50–66% of all recently infected cases (Sterling & Chaisson, 2005). Due to mild and non-specific flu-like symptoms many people do not seek medical advice, and thus are not diagnosed and treated and proceed to a latent stage where they may remain asymptomatic for years before subsequently developing AIDS.

Within the EU, it is estimated that around 8–45% of all HIV infections are undiagnosed and therefore not reported to the health authorities (ECDC, 2014). The overall duration is difficult to estimate because since introduction of Anti-Retroviral Therapy (ART) HIV is increasingly observed as being a chronic disease and individuals receiving treatment have a similar life expectancy to the rest of the population in Europe (Bhaskaran, 2008). Persistent asymptomatic HIV infection is estimated to be on average 17.2 years for long-term non-progressors (Herida, 2006).

Health outcomes/states associated with HIV-infection

HIV is associated with a heterogeneous set of health outcomes/states. In most cases, certain health outcomes/states are caused by subsequent infections with a secondary or tertiary pathogen. HIV compromises the immune status of an individual and thus increases the risk of further additional pathogens causing severe sequelae.

For our study, we considered that in Europe development of AIDS is significantly limited through ART.

HIV/AIDS is a complicated, multi-faceted and systemic disease and for reasons of feasibility, we developed a simplified model which does not differentiate between the CD4 count stages of the disease at the point of diagnosis, even though this is known to affect mortality (Aghaizu, 2013). Moreover, the current model does not take into account transmitted drug resistance, or the issue of co-morbidity (HIV–HCV or HIV–TB) and the consequent need for a specific therapeutic pathway. We assumed that all diagnosed cases are offered treatment and we applied a certain burden to the disease (e.g. side effects).

HIV infection-related deaths are associated with the development of an acquired immunodeficiency syndrome (AIDS) which, after a prolonged latent period, eventually enables opportunistic infections to develop which are generally the cause of death. Therefore, the nature of AIDS itself consists of comorbidities introducing the issue of attributable cause of death. However, we assumed that the severity of the co-infection and the precipitation to death would not have occurred without the primary HIV infection and deaths were therefore attributed entirely to the initial HIV infection. We also did not include the burden associated with HIV-related malignancies or complications linked to long-term antiretroviral therapy (e.g. cardiovascular disease).

Outcome-tree parameters

The main input is 'persistent HIV infection' and this is subdivided according to the speed of progression (Qu, 2008). In general, 5–15% of all patients are rapid progressors (RP) and are at risk of developing AIDS within 2–5 years (Qu, 2008). Another 5–15% are long-term non- progressors (LNP) with, on average 17.2 years duration of development (Qu, 2008; Sterling & Chaisson, 2005). The remainder (70–90%) are typical progressors (TP) with an average duration of 8–10 years (Qu, 2008).

The risk of developing early symptomatic AIDS is set at between 4.5% and 7% (Grinsztejn, 2014: 40 observed cases out of 886 in the group with early ART initiation versus 61 out of 877 in the delayed group).

Terminal AIDS has a duration of one month (Kwong, 2010) and the risk of developing terminal AIDS from early symptomatic AIDS is set at 32.09% as this was the case fatality proportion estimated for AIDS in a recent study (Serraino, 2010).

Model input summary

Table 1. Transition probabilities and distributions used in the outcome tree

Health outcome (Health state)	Distribution of health states in health outcome	Transition probability	Source/assumption
Persistent HIV infection			No cure available
(Rapid progressors)	5–15%		Qu, 2008
(Typical progressors)	70–90%		Qu, 2008
(Long-term non-progressors)	5–15%		Qu, 2008; Herida, 2006
AIDS early symptomatic		4.5–7%	Grinsztejn, 2014
AIDS terminal phase		32.09%	Serraino, 2010
CFR from AIDS		100%	

Table 2. Disability weights and duration

	Disability Weight (DW) (Haagsma, 2015)		Duration

Health outcome<br>(Health state)	DW	Label	In years	Source
Persistent HIV infection	0.108<br>(0.089-0.132)	HIV/AIDS cases, receiving ARV treatment		
(Rapid progressors)			2-5	Qu, 2008
(Typical progressors)			8-10	Qu, 2008
(Long-term non-progressors)			17.2	Qu, 2008; Herida, 2006
Permanent ARV treatment	0.108<br>(0.089-0.132)	HIV/AIDS cases, receiving ARV treatment	Remaining life expectancy	Assuming ARV treatment has optimal effectiveness and good compliance
AIDS early symptomatic	0.351<br>(0.299–0.394)	HIV cases, symptomatic, pre-AIDS	5.36	Herida, 2006
AIDS terminal phase	0.574<br>(0.518–0.635)	AIDS cases, not receiving ARV treatment	0.08	Kwong, 2010

References

Aghaizu A, Brown AE, Nardone A, Gill ON, Delpech VC & contributors. HIV in the United Kingdom 2013 - Report: data to end 2012. November 2013. Public Health England, London. Available at: https://www.gov.uk/government/uploads/system/uploads/attachment_data/file/326601/HIV_annual_report_2013.pdf

Barre-Sinoussi F, Chermann JC, Rey F, Nugeyre MT, Chamaret S, Gruest J, et al. Isolation of a T-lymphotropic retrovirus from a patient at risk for acquired immune deficiency syndrome (AIDS). Science 1983, 220:868-871.

Bhaskaran K, Hamouda O, Sannes M, Boufassa F, Johnson AM, Lambert PC, et al. for the CASCADE Collaboration. Changes in the Risk of Death After HIV Seroconversion Compared With Mortality in the General Population. JAMA. 2008;300(1):51-59. doi:10.1001/jama.300.1.51.

Cameron DW, Simonsen JN, D'Costa LJ, Ronald AR, Maitha GM, Gakinya MN, et al. Female to male transmission of human immunodeficiency virus type 1: risk factors for seroconversion in men. Lancet 1989, 2:403-407.

Cleghorn FR, Reits Jr. MS, Popovic M, Gallo RC. Human Immunodeficiency Viruses. In Principles and Practice of Infectious Diseases. Volume 2. Edited by Mandell GL, Bennet JE, Dolin R. Pennsylvania: Elsevier Inc.; 2005: 2119-2132

HIV/AIDS. Available at: http://www.who.int/topics/hiv_aids/en/

Del Rio C, Curran JW. Epidemiology and Prevention of Acquired Immunodeficiency Virus Infection. In Principles and Practice of Infectious Diseases. Volume 1. Edited by Mandell GL, Bennet JE, Dolin R. Pennsylvania: Elsevier Inc.; 2005: 1477-1506

European Centre for Disease Prevention and Control. Monitoring implementation of the Dublin Declaration on Partnership to Fight HIV/AIDS in Europe and Central Asia: 2014 progress report, in press.

Gallo RC, Sarin PS, Gelmann EP, Robert-Guroff M, Richardson E, Kalyanaraman VS, et al. Isolation of human T-cell leukemia virus in acquired immune deficiency syndrome (AIDS). Science 1983, 220:865-867.

Grinsztejn B, Hosseinipour M, Ribaudo H, Havlir D, Cohen M, et al. (2014). Effects of Early versus Delayed Initiation of Antiretroviral Therapy on HIV Clinical Outcomes: Results from the HPTN 052. The Lancet Infectious Diseases, Volume 14, Issue 4, April 2014, Pages 281-290

Haagsma JA, Maertens de Noordhout C, Polinder S, Vos T, Havelaar AH, Cassini A, Devleesschauwer B, Kretzschmar ME, Speybroeck N, Salomon JA. Assessing disability weights based on the responses of 30,660 people from four European countries. Population Health Metrics 2015; 13: 10

Herida M, Larsen C, Lot F, Laporte A, Desenclos JC, Hamers FF. Cost-effectiveness of HIV post-exposure prophylaxis in France. AIDS 2006, 20:1753-1761.

Kwong JC, Crowcroft NS, Campitelli MA, Ratanasingham S, Daneman N, Deeks SL, et al. Ontario Burden of Infectious Disease Study. In: Ontario Burden of Infectious Disease Study (ed.), Vol. OAHPP/ECES Report. City: Ontario Agency for Health Protection and Promotion and the Institute for Clinical Evaluative Sciences; 2010.

Laga M, Manoka A, Kivuvu M, Malele B, Tuliza M, Nzila N,et al. Non-ulcerative sexually transmitted diseases as risk factors for HIV-1 transmission in women: results from a cohort study. AIDS 1993, 7:95-102.

Merson MH, Piot P. Global Perspectives on Human Immunodeficiency Virus Infection and Acquired Immunodeficiency Syndrome. In Principles and Practice of Infectious Diseases. Volume 1. Edited by Mandell GL, Bennet JE, Dolin R. Pennsylvania: Elsevier Inc.; 2005: 1456-1506

Qu W, Robinson M, Zhang FJ. Factors influencing the natural history of HIV-1 infection. Chin Med J (Engl) 2008, 121:2613-2621.

Serraino D, Bruzzone S, Zucchetta A, Suligoi B, De Paoli A, Pennazza S, et al. Elevated risks of death for diabetes mellitus and cardiovascular diseases in Italian AIDS cases. AIDS Research and Therapy 2010, 7(11)

Sterling TR, Chaisson RE: General Clinical Manifestations of Human Immunodeficiency Virus Infection (Including the Acute Retroviral Syndrome and Oral, Cutaneous, Renal, Ocular and Cardia Diseases). In Principles and Practice of Infectious Diseases. Volume 1. Edited by Mandell GL, Bennet JE, Dolin R. Pennsylvania: Elsevier Inc.; 2005: 1547-1574

Influenza

In most cases influenza infection in humans is uncomplicated and self-limiting within a few days or weeks, but for some patients the disease is fatal. Approximately one third of influenza infections are mild or asymptomatic, to the extent that infected persons do not even see a doctor (Hayward, 2010; Hayward, 2014). Our model assumes a mean duration of five days (Nicholson, 2003).

Wielders et al. (2010) included four different outcomes and their long-term sequelae following acute illness. These were pneumonia, otitis media, acute respiratory distress syndrome (ARDS) and sepsis. The frequency of other post-infectious complications following an influenza infection is low and these were therefore disregarded in the current study. From a clinical perspective, the acute manifestations of the disease often occur in concomitance as complicated cases

Based on information derived from the General Practice Research Database (GPRD), Meier et al. (2000) estimated the number of patients consulting a doctor with symptoms of influenza-like illness (ILI) who developed complications. The percentages were based on subjects who had at least one clinical diagnosis of influenza or influenza-like-illness (ILI) recorded in the GPRD between 1991 and 1996. In addition to the wide range of national case definitions, estimated consultation rates will also vary among countries due to differences in consultation behaviour, estimation procedure (estimation of incidence, given that many surveillance systems are based on sentinel reporting), vaccination coverage (although vaccination has a limited impact on the number of consultations) and obligatory doctor visits for absence from work or school (Harbers, 2005; Meijer et al., 2006). Therefore, doctor consultations were not considered to be indicative of acute complicated influenza disease.

Given very little specific information on the ratio of complicated/uncomplicated acute disease, no distinction was made between these and the variability was accounted for by including all possible manifestations in the disability weight (mild, moderate and severe): 0.051 (0.007–0.125).

Case fatality ratio

Research has shown that clinicians often attribute influenza-related deaths to a pre-existing underlying condition rather than to influenza (Zucs et al., 2005). Therefore, it is difficult to identify true mortality due to influenza only. Distinguishing further between mortality due to influenza with or without complications such as cardiac problems or pneumonia is even more difficult. Therefore in the current study only one category of death was considered, encompassing all causes which, in the model, occur shortly after infection.

For the Netherlands, it was estimated that during the period 1967–1989 the overall impact of influenza on mortality was greater than registered mortality by a factor of 3.6 (Sprenger et al., 1993). Using this multiplication factor for more recent data may overestimate the number of deaths due to influenza, because in many Member States today vaccination coverage is considerably higher than in the period 1967– 1989. In the study by Sprenger et al. almost half of the non-registered influenza deaths were registered as deaths from heart disease, approximately 25% from lung disease and approximately 30% from other diseases (Sprenger et al., 1993). Recently, time series analysis has also been used to estimate mortality attributable to influenza and other respiratory pathogens (van den Wijngaard et al., 2010).

In about 0.1% of all influenza cases the disease will be fatal (Flu.gov, 2012). This includes both uncomplicated and complicated influenza cases.

Approximately 90% of persons with influenza as cause of death were aged ≥ 65 years (Webster, 2013). Therefore, given that the case fatality proportion for influenza is age-dependent, we modelled the age-specific risk according to the observed mortality data in Estonia, Germany and the Netherlands (see Table 3) (CBS, 2009).

Risk of complications

The most vulnerable populations in terms of complications following influenza are children aged under one 1 year and adults over 65 years, pregnant woman, and people of any age with comorbid illnesses (Rothberg et al., 2008).

The most common complications of influenza are secondary bacterial infections, especially otitis media and pneumonia (van Steenbergen, et al., 2006). It is estimated that 0.65% of influenza cases develop otitis media and 0.36% pneumonia (Meier et al., 2000). Secondary

bacterial pneumonia most often complicate the condition 4–14 days after primary seasonal influenza infection (Rothberg et al., 2008). Neurological complications such as encephalopathy (Reye's syndrome), encephalomyelitis, transverse myelitis, aseptic meningitis, focal neurological disorders, and Guillain-Barré syndrome most often appear in small children (Rothberg et al., 2010). The incidence of neurological complications among <5 years was estimated to be 4 per 100 000 (Newland, 2007).

Wielders et al. (2010) assumed that about 1.23% of all influenza cases develop pneumonia. Earlier, van Lier et al. (2007) assumed that this fraction was 0.36%. In most cases the disease will be self-limiting within a few days, and only in a few cases will it be fatal. According to Murray et al. (1996) long-term outcomes of pneumonia in developed countries are very rare and can be disregarded when estimating disease burden.

Wielders et al. (2010) assumed that 0.65% of influenza cases will develop otitis media as a complication of influenza. Most affected persons will fully recover, but 0.006% of otitis media cases will develop deafness as a life-long disability (Murray, 1996). Given the very low risk, we considered this complication as negligible.

A few cases will develop sepsis during an influenza infection, estimated at 0.0097% of all cases (Wielders, 2010). In some cases the disease will be fatal but again, since there was no detailed information available on the percentage, we assumed that fatal cases would be included in the death estimate related to influenza. Long-term disability was estimated to occur in 82% of patients surviving sepsis (Korosec Jagodic, 2006). However, given the fact that sepsis is caused by bacteria giving rise to super-infections possibly related to other factors, the long-term sequelae of sepsis are not considered to be part of the burden of influenza infections.

Acute respiratory distress syndrome (ARDS) and life-long disability

Following Wielders et al. (2010), we assumed that 0.023% of influenza cases will develop ARDS as a complication of influenza. We assumed that the risk of developing ARDS changes according to age (Manzano, 2005). Wielder's study, however, does not consider cases <15 years and in order to account for these, we also included a study on younger populations (Zimmerman, 2009). We combined the ARDS incidence from the two studies, added them together and estimated the age-group risk of developing ARDS (see Table 4).

In a few cases the disease will be fatal. However, having no detailed information on the specific risk, we assumed that fatal cases would be included in the death estimate related to influenza. Around 30–55% (Hopkins, 1999; Mikkelsen, 2012) of patients surviving ARDS will have developed disabilities related to cognitive impairments at one year follow-up. Therefore, in our model, we estimated that 0.007–0.013% of all symptomatic influenza cases will develop cognitive sequelae assumed to be permanent.

Model input summary

Table 1. Transition probabilities used in the outcome tree

Health outcome (Health state)	Distribution of health states in health outcome	Transition probability	Source/assumption
Permanent disability due to ARDS		0.007–0.013% Age dep. (Table 4)	Wielders, 2010; Manzano, 2005; Hopkins, 1999; Mikkelsen, 2012
Fatal cases		0.10% Age dep. (Table 3)	Flu.gov, 2012; observed cases

Table 2. Disability weights and duration

Health outcome (Health state)	Disability Weight (DW) (Haagsma, 2015)		Duration	
	DW	Label	In years	Source
Symptomatic infection	0.051 (0.007–0.125)	Infectious disease, acute episode, from mild to severe	0.014	Nicholson, 2003
Permanent disability due to ARDS	0.056 (0.044–0.067)	Motor plus cognitive impairments, mild	Remaining life expectancy	Hopkins, 1999; Mikkelsen, 2012

Table 3. Age group distribution of 0.1% risk of fatal cases

Age	%
0	0.58
01-04	0.51
05-09	0.24
10-14	0.27
15-19	0.24

20-24	0.33
25-29	0.31
30-34	0.33
35-39	0.75
40-44	1.15
45-49	1.56
50-54	1.53
55-59	2.21
60-64	3.23
65-69	4.54
70-74	5.22
75-79	11.42
80-84	18.72

85+	46.85

Source: based on all reported fatal influenza cases in Estonia, Germany and the Netherlands for the years 2005–2007.

Table 4. Age group distribution of 0.007–0.013% risk of developing ARDS

Age	%
0-14	7.21
15-29	2.59
30-44	7.66
45-59	12.17
60-74	28.73
≥75	41.63

References

CBS (Statistics Netherlands). Causes of death, 2009. Available at: http://statline.cbs.nl/StatWeb/

U.S. Department of Health & Human Services, Flu.gov (US). Community strategy for pandemic influenza mitigation. 2007 Feb.www.flu.gov/professional/community/mitigation.html [cited 12 March 2010]

Haagsma JA, Maertens de Noordhout C, Polinder S, Vos T, Havelaar AH, Cassini A, Devleesschauwer B, Kretzschmar ME, Speybroeck N, Salomon JA. Assessing disability weights based on the responses of 30,660 people from four European countries. Population Health Metrics 2015; 13: 10

Harbers MM. Influenza: are there international differences? In: Public Health Forecast, National Compass Public Health [Available at http://www.rivm.nl/vtv/object_document/o1736n18081.html]. 2005. Bilthoven: National Institute for Public Health and the Environment (RIVM).

Hayward AC et al. The community burden of influenza and Influenza-Like-Illness in England – Early results from the MRC Flu Watch Study. 2010. Personal communication with Professor Hayward.

Hayward AC, Fragaszy EB, Bermingham A, Wang L, Copas A, Edmunds WJ, et al. Comparative community burden and severity of seasonal and pandemic influenza: Results of the Flu Watch cohort study. Lancet Respir. Med. 2014, 2014, doi:10.1016/S2213-2600(14)70034-70037.

Hopkins RO, Weaver LK, Pope D, Orme JF Jr., Bigler ED, Larson-Lohr V. Neuropsychological sequelae and impaired health status in survivors of severe acute respiratory distress syndrome. American Journal of Respiratory and Critical Care Medicine. 1999;160(1):50–56.

Korosec Jagodic H, Jagodic K, Podbregar M. Long-term outcome and quality of life of patients treated in surgical intensive care: a comparison between sepsis and trauma. Crit Care 2006;10: R134.

Manzano F, Yuste E, Colmenero M, Garcia-Horcajadas A, Rivera R, Fernandez-Mondejar E. Granada Respiratory Failure Study Group: Incidence of acute respiratory distress syndrome and its relation to age. J Crit Care. 2005;20:274–80.

Meier CR, Napalkov PN, Wegmuller Y, Jefferson T, Jick H. Population-based study on incidence, risk factors, clinical complications and drug utilisation associated with influenza in the United Kingdom. Eur J Clin Microbiol Infect Dis 2000. 19 (11): 834-42.

Meijer A, Paget WJ, Meerhoff TJ, Brown C, Meuwissen LE, Van Der Velden J. Epidemiological and virological assessment of influenza activity in Europe, during the 2004-2005 winter. Euro Surveill 2006;11(5): 111-8.

Mikkelsen ME, Christie JD, Lanken PN, Biester RC, Thompson BT, Bellamy SL, et al. The adult respiratory distress syndrome cognitive outcomes study: long-term neuropsychological function in survivors of acute lung injury. Am J Respir Crit Care Med. 2012;17:1307–1315. doi: 10.1164/rccm.201111-2025OC.

Murray CJL, Lopez AD (eds.) (1996). Global health statistics: a compendium of incidence, prevalence and mortality estimates for over 200 conditions (Global burden of disease and injuries series; II). Cambridge: Harvard School of Public Health on behalf of the World Health Organization and the World Bank.

Neurologic Complications in Children Hospitalized with Influenza: Characteristics, Incidence, and Risk Factors. Newland JG, Laurich VM, Rosenquist AW, Heydon K, Licht DJ, Keren R et al. The Journal of Pediatrics, 2007; Vol. 150, 3,306–310

Nicholson KG, Wood JM, Zambon M. Influenza. Lancet 2003; 362:1733-45.

Rothberg MB, Haessler SD, Brown RB (2008). Complications of viral influenza. Am J Med 121(4): 258-64.

Rothberg MB, Haessler SD (2010). Complications of seasonal and pandemic influenza. Crit Care Med 38(4 Suppl): e91-7.

Sprenger MJ, Mulder PG, Beyer WE, Van Strik R, Masurel N (1993). Impact of influenza on mortality in relation to age and underlying disease, 1967-1989. Int J Epidemiol 22(2): 334-40.

van den Wijngaard CC, van Asten L, Meijer A, van Pelt W, Nagelkerke NJ, Donker GA, et al (2010). Detection of excess influenza severity: associating respiratory hospitalization and mortality data with reports of influenza-like illness by primary care physicians. Am J Public Health 100(11): 2248-54.

van Lier EA, Havelaar AH (2007). Disease burden of infectious diseases in Europe: a pilot study. Bilthoven: National Institute for Public Health and the Environment (RIVM) (RIVM report 215011001).

van Steenbergen J, Timen EA (eds) (2006). Guidelines Infectious Disease Control Edition 2006. Bilthoven: LCI, Coordinator Infectious Disease Control for the Netherlands.

Webster, Monto, Bracciale, Lamb. Textbook of Influenza. 2nd Edition, 2013. Wiley Blackwell Ed.

Wielders CCH, van Lier EA, van 't Klooster TM, van Gageldonk-Lafeber AB, van den Wijngaard CC, Haagsma JA, et al. The burden of 2009 pandemic influenza A(H1N1) in the Netherlands. Eur J Public Health 2010. http://dx.doi.org/10.1093/eurpub/ckq187

Zimmerman JJ, Akhtar SR, Caldwell E, Rubenfeld GD. Incidence and outcomes of pediatric acute lung injury. Pediatrics. 2009;124:87–95.

Zucs P, Buchholz U, Haas W, Uphoff H. Influenza associated excess mortality in Germany, 1985-2001. Emerg Themes Epidemiol 2005.2:6.

Invasive haemophilus influenza disease

The major disease burden of invasive H. influenzae infection occurs in children under five years (Fogarty, 1995). The most harmful complication is bacteraemia, which is accompanied by a focal infection such as meningitis, pneumonia, or cellulitis in 30–50% of cases (Devarajan, 2009).

Risk of complications

Meningitis is the principal clinical presentation of invasive disease, but bone and joint infections, pneumonia, epiglottitis, cellulitis and septicaemia can also occur. Skin and soft tissue infections may occur in around 6% of patients, followed by a limited number of sequelae (Otero Reigada, 2005). Only the invasive forms are considered as health states in the model.

To estimate the risk of meningitis we used the surveillance data reported in the ECDC Invasive Disease Surveillance report on clinical presentations of the acute symptomatic disease (ECDC, 2013a; ECDC, 2013b). Reported data indicates that meningitis and septicaemia occur together in 0–1% of cases, whereas meningitis alone occurs in 15–18% (15% in 2010, 18% in 2011) of cases, resulting in an overall risk of 15– 18% of developing meningitis. The risk of developing meningitis during the acute phase of the disease is age-specific. Age and gender-specific data were extracted from ECDC's TESSy database on the meningitis complications of IHID for 2010 and 2011 (see Table 4). The risk of developing the long-term sequelae is age and gender-specific.

Long-term sequelae

Bacterial meningitis may cause long-term sequelae and permanent disabilities. To investigate this we extracted the risk of developing these complications after meningitis episodes from Edmond et al. (Edmond, 2010).

Meningitis accounts for various long-term sequelae (each of which is multiplied by the risk of developing meningitis during the acute phase of the disease: 15–18%): cognitive difficulties (0.17–0.20%), seizure disorders (0.23–0.27%), hearing loss (0.48–0.58%), motor deficit (0.33– 0.40%), visual disturbance (0.08–0.09%), behavioural problems (0.32–0.38%), clinical impairments (0.18–0.22%) and multiple impairments (0.39–0.47%) (Edmond, 2010).

Case fatality proportion

The parameters for the case fatality proportion were based on data for EU/EEA countries in 2011, see Table 3 (ECDC, 2013).

Model input summary

Table 1. Transition probabilities used in the outcome tree

Health outcome (Health state)	Distribution of health states in health outcome	Transition probability	Source/assumption
Hearing loss		0.48-0.58%	Edmond, 2010

Cognitive difficulties		0.17-0.20%	Edmond, 2010
Seizure disorder		0.23-0.27%	Edmond, 2010
Motor deficit		0.33-0.40%	Edmond, 2010
Visual disturbance		0.08-0.09%	Edmond, 2010
Behavioural problems		0.32-0.38%	Edmond, 2010
Clinical impairments		0.18-0.22%	Edmond, 2010
Multiple impairments		0.39-0.47%	Edmond, 2010
Fatal cases due to symptomatic infection		See Table 3 (5.4-19.5%)	ECDC, 2013

Table 2. Disability weights and duration

Health outcome (Health state)	Disability Weight (DW) (Haagsma, 2015)		Duration	
	DW	Label	In years	Source
Symptomatic infection	0.655 (0.579-0.727)	Intensive care unit admission	0.019	Tunkel, 2004 Assuming the duration of antimicrobial therapy

Permanent disability following meningitis			Remaining life expectancy	
1. Hearing loss	0.008-0.103	From lowest to highest hearing loss related DWs		
2. Cognitive difficulties	0.044-0.188	From lowest to highest intellectual disability related DWs		
3. Seizure disorder	0.07 (0.057-0.088)	Generic uncomplicated disease: worry and daily medication		
4. Motor deficit	0.011-0.421	From lowest to highest motor impairment related DWs		
5. Visual disturbance	0.004-0.171	From lowest to highest vision impairment related DWs		
6. Behavioural problems	0.088 (0.07-0.108)	Subacute sclerosing panencephalitis – phase 1 (assuming best fitting health state description)		
7. Clinical impairments	0.004-0.421	From lowest to highest DW included in this model		
8. Multiple impairments	0.004-0.421	From lowest to highest DW included in this model		

Table 3. CFR following symptomatic infection

Age	CFR
0	19.5%
1-4	6.5%
5-14	5.7%
15-64	5.4%
≥65	15%

Table 4. Age specific distribution per gender of the 15-18% risk of developing meningitis manifestation during the symptomatic infection (TESSy 2010-2011)

Age group		%

	F	M
0	15.69	17.12
01-04	15.69	18.49
05-09	2.61	5.48
10-14	1.31	2.74
15-19	1.31	2.74
20-24	2.61	4.11
25-29	0.00	0.00
30-34	3.27	1.37
35-39	1.96	4.79
40-44	3.92	7.53
45-49	3.92	8.22
50-54	7.19	2.74
55-59	7.19	2.74
60-64	3.27	4.79
65-69	10.46	3.42
70-74	11.11	5.48
75-79	5.23	4.11
80-84	2.61	1.37
85+	0.65	2.74
Total	100	100

References

Buysse CM, Oranje AP, Zuidema E, Hazelzet JA, Hop WC, Diepstraten AF, et al. Long-term skin scarring and orthopaedic sequelae in survivors of meningococcal septic shock. Arch Dis Child. 2009 May;94(5):381-6.

Devarajan VR. Haemophilus Influenzae Infections. 2009. http://emedicine.medscape.com/article/218271-overview

Edmond K, Clark A, Korczak VS, Sanderson C, Griffiths UK, Rudan I. Global and regional risk of disabling sequelae from bacterial meningitis: a systematic review and meta-analysis. Lancet Infect Dis. 2010 May;10(5):317-28.

Eidelman LA, Putterman D, Putterman C, Sprung CL. The Spectrum of Septic Encephalopathy: Definitions, Etiologies, and Mortalities. JAMA. 1996;275(6):470-473. doi:10.1001/jama.1996.03530300054040.

European Centre for Disease Prevention and Control. Surveillance of invasive bacterial diseases in Europe, 2010. Stockholm: ECDC; 2013.

European Centre for Disease Prevention and Control. Surveillance of invasive bacterial diseases in Europe, 2011. Stockholm: ECDC; 2013.

Fogarty J, Moloney AC and Newell JB. The epidemiology of Haemophilus influenzae type b disease in the Republic of Ireland. Epidemiol. Infect. (1995). 114. 451-463.

Haagsma JA, Maertens de Noordhout C, Polinder S, Vos T, Havelaar AH, Cassini A, Devleesschauwer B, Kretzschmar ME, Speybroeck N, Salomon JA. Assessing disability weights based on the responses of 30,660 people from four European countries. Population Health Metrics 2015; 13: 10

Jit M. The risk of sequelae due to pneumococcal meningitis in high-income countries: a systematic review and meta-analysis. J Infect 2010;61(2):114-24.

Otero Reigada MC, Piqueras Arenas AI, Ferrer Lorente B, Pérez-Tamarit D, Asensi Botet F, Santos Durantez M. Skin and soft tissue infections caused by Haemophilus influenzae type b over a 30 year period. An Pediatr (Barc). 2005 Jul;63(1):29-33.

Tunkel AR, Hartman BJ, Kaplan SL, Kaufman BA, Roos KL, Scheld WM, et al. Practice guidelines for the management of bacterial meningitis. Clin Infect Dis 2004, 39(9):1267–1284.

Ziaja M. Septic encephalopathy. Curr Neurol Neurosci Rep. 2013;13:383

Invasive meningococcal disease (IMD)

As many as 10% of adolescents and adults are asymptomatic transient carriers of N. meningitidis, most strains of which are not pathogenic. In more than 99% of the cases the infection is asymptomatic, but about 1% of the those infected develop acute illness (CDC, 2009). Invasive disease usually requires a seven-day course of antibiotic therapy (Brigham & Sandora, 2009; Tunkel 2004), but may also result in lifelong major sequelae.

Risk of complications

Meningitis is the most common manifestation of invasive disease, and may occur in 47.3% of all patients suffering from N. meningitidis symptomatic infection and in 52.2% of the patients who develop bacteraemia. It always follows hematogenous dissemination, which occurs in 91% of all patients suffering from symptomatic infection. Sepsis occurs in 5–20% of patients with invasive disease (CDC, 2009). Complications are also possible with non-invasive disease; pneumonia occurs in 6% of symptomatic infections, otitis media in 1% of cases and epiglottitis, which is rare, in 0.3% of all manifestations (CDC, 2009).

We decided to use surveillance data reported to TESSy on clinical presentations of the acute symptomatic disease to estimate the risk of meningitis (ECDC, 2013). Reported data indicates that meningitis and septicaemia together occur in 17–18% of cases, whereas meningitis alone occurs in 43–45% of cases, resulting in an overall risk of 60–63% of developing meningitis. The risk of developing meningitis during the acute phase of the disease is age-specific. Age-specific data were extracted for each gender from ECDC's TESSy database on the meningitis complications of IMD for 2010 and 2011 (see Table 4). The risk of developing long-term sequelae is age and gender-specific.

Long term sequelae

Bacterial meningitis may cause long-term sequelae and permanent disabilities. The risk of developing these complications after meningitis episodes was extracted from Edmond et al. (Edmond, 2010).

Meningitis accounts for various long-term sequelae (each of which is multiplied by the risk of developing meningitis during the acute phase of the disease: 60–63%): cognitive difficulties (0.96–1.01%), seizure disorders (0.3–0.35%), hearing loss (1.56–1.64%), motor deficit (0.6– 0.63%), visual disturbance (0.9–0.95%), behavioural problems (0.36–0.38%), clinical impairments (0.12–0.13%) and multiple impairments (0.78-0.82%) (Edmond, 2010).

Case fatality proportion

The parameters for the case fatality ratio were based on data for EU/EEA countries in 2011, see Table 3 (ECDC, 2013).

Model input summary

Table 1. Transition probabilities used in the outcome tree

Health outcome (Health state)		Distribution of health states in health outcome		Transition probability		Source/assumption

Hearing loss		1.56–1.64%	Edmond, 2010
Cognitive difficulties		0.96–1.01%	Edmond, 2010
Seizure disorder		0.3–0.35%	Edmond, 2010
Motor deficit		0.6–0.63%	Edmond, 2010
Visual disturbance		0.9–0.95%	Edmond, 2010
Behavioural problems		0.36–0.38%	Edmond, 2010
Clinical impairments		0.12–0.13%	Edmond, 2010
Multiple impairments		0.78–0.82%	Edmond, 2010
Fatal cases due to symptomatic infection		See Table 3 (6.9-17.1%)	ECDC, 2013

Table 2. Disability weights and duration

Health outcome (Health state)	Disability Weight (DW) (Haagsma, 2015)		Duration	
	DW	Label	In years	Source
Symptomatic infection	0.655 (0.579-0.727)	Intensive care unit admission	0.019	

				Tunkel, 2004 Assuming the duration of antimicrobial therapy
Permanent disability following meningitis:			Remaining life expectancy	
1. Hearing loss	0.008-0.103	From lowest to highest hearing loss related DWs		
2. Cognitive difficulties	0.044-0.188	From lowest to highest intellectual disability related DWs		
3. Seizure disorder	0.07 (0.057-0.088)	Generic uncomplicated disease: worry and daily medication		
4. Motor deficit	0.011-0.421	From lowest to highest motor impairment related DWs		
5. Visual disturbance	0.004-0.171	From lowest to highest vision impairment related DWs		
6. Behavioural problems	0.088 (0.07-0.108)	Subacute sclerosing panencephalitis – phase 1 (assuming best fitting health state description)		
7. Clinical impairments	0.004-0.421	From lowest to highest DW included in this model		
8. Multiple impairments	0.004-0.421	From lowest to highest DW included in this model		

Table 3. CFP following symptomatic infection

Age	CFR
0	7.8%

1-4	6.9%
5-14	5.6%
15-24	9.5%
25-49	8.9%
50-64	7.6%
≥65	17.1%

Table 4. Age specific distribution per gender of the 60-63% risk of developing meningitis manifestation during the symptomatic infection (TESSy 2010-2011)

Age group	%	
	F	M
0	16.22	16.64
01-04	18.19	23.79
05-09	7.13	8.65
10-14	5.90	4.46
15-19	14.53	15.97
20-24	7.21	8.06
25-29	3.93	3.62
30-34	2.62	3.04

35-39	2.05	1.69
40-44	2.54	1.84
45-49	2.81	2.01
50-54	3.47	2.43
55-59	3.28	2.43
60-64	1.97	1.42
65-69	1.88	1.42
70-74	1.97	0.76
75-79	2.05	1.35
80-84	1.31	0.34
85+	0.93	0.08
Total	100	100

References

Brigham KS, Sandora TJ. Neisseria meningitidis: epidemiology, treatment and prevention in adolescents. Curr Opin Pediatr. 2009 Aug;21(4):437-43.

Buysse CM, Oranje AP, Zuidema E, Hazelzet JA, Hop WC, Diepstraten AF, et al. Long-term skin scarring and orthopaedic sequelae in survivors of meningococcal septic shock. Arch Dis Child. 2009 May;94(5):381-6.

Centers for Disease Control and Prevention. Epidemiology and Prevention of Vaccine-Preventable Diseases. Atkinson W, Wolfe S, Hamborsky J, McIntyre L, eds. 11th ed. Washington DC: Public Health Foundation, 2009.

Edmond K, Clark A, Korczak VS, Sanderson C, Griffiths UK, Rudan I. Global and regional risk of disabling sequelae from bacterial meningitis: a systematic review and meta-analysis. Lancet Infect Dis. 2010 May;10(5):317-28.

Eidelman LA, Putterman D, Putterman C, Sprung CL. The Spectrum of Septic Encephalopathy: Definitions, Etiologies, and Mortalities. JAMA. 1996;275(6):470-473. doi:10.1001/jama.1996.03530300054040.

European Centre for Disease Prevention and Control. Surveillance of invasive bacterial diseases in Europe, 2010. Stockholm: ECDC; 2013.

European Centre for Disease Prevention and Control. Surveillance of invasive bacterial diseases in Europe, 2011. Stockholm: ECDC; 2013.

Haagsma JA, Maertens de Noordhout C, Polinder S, Vos T, Havelaar AH, Cassini A, Devleesschauwer B, Kretzschmar ME, Speybroeck N, Salomon JA. Assessing disability weights based on the responses of 30,660 people from four European countries. Population Health Metrics 2015; 13: 10

Jit M. The risk of sequelae due to pneumococcal meningitis in high-income countries: a systematic review and meta-analysis. J Infect 2010;61(2):114-24.

Tunkel AR, Hartman BJ, Kaplan SL, Kaufman BA, Roos KL, Scheld WM, et al. Practice guidelines for the management of bacterial meningitis. Clin Infect Dis 2004, 39(9):1267–1284.

Ziaja M. Septic encephalopathy. Curr Neurol Neurosci Rep. 2013;13:383.

Invasive pneumococcal disease

Despite the large number of serogroups and serotypes known, most cases of invasive pneumococcal disease (IPD) on a global scale are attributed to the 1, 3, 4, 6, 7, 9, 14, 18, 23 (Jefferson, 2006) and 19a serogroups.

Risk of complications

Invasive pneumococcal infection can manifest as meningitis, bacteraemic pneumonia, bacteraemia without a focus, and bacteraemia with a focus other than the lungs or meninges (e.g. endocarditis, osteomyelitis, and arthritis, although rare). Complications, such as pneumonia or otitis media, are also possible with non-invasive forms of infection but are not considered in this study.

Most observed complications of invasive bacterial diseases, including IPD, are related to the meningitis event. The risk of meningitis was estimated using surveillance data reported to TESSy on clinical presentations of the acute symptomatic disease (ECDC, 2013) and it was found that 10% of IPD cases are reported to manifest meningitis. The risk of developing meningitis during the acute phase of the disease is age- specific. Age and gender-specific data were extracted from ECDC's TESSy database on the risk of developing meningitis for IPD cases from 2010 to 2011 (see Table 4). The risk of developing long-term sequelae is age and gender-specific.

Long-term sequelae

Bacterial meningitis may cause long-term sequelae and permanent disabilities. In order to account for these, information was extracted on the risk of developing permanent sequelae from Edmond et al. (Edmond, 2010).

Meningitis can result in various long-term sequelae: cognitive difficulties (4.2%), seizure disorders (2.5%), hearing loss (7.5%), motor deficit (5.8%), visual disturbance (1.1%), behavioural problems (4.6%) multiple (5.7%) and clinical impairments (3.3%) (Edmond, 2010). Therefore, we assumed that 10% of all IPD patients would be at risk of developing long-term sequelae.

Case fatality proportion

The case fatality proportion for invasive pneumococcal disease has been estimated at 18% in a population-based study of 19 000 people (Harboe, 2009); however, important differences were observed between age groups, with a lower (3%) mortality rate observed in children <5 years. The overall lethality rate due to bacteraemia is about 10–20% (CDC, 2009; Rudan, 2009; Lin, 2010; Saldías, 2009) and may be as high as 60% among elderly patients (CDC, 2009).

Overall mortality due to endocarditis is 50%, but it can reach 60–65% in children (Elward, 1990). The case-fatality proportion for pneumococcal meningitis is about 30%, but may be as high as 80% in elderly persons (CDC, 2009; Burckhardt et al. 2010). The parameters for the case fatality proportion were based on data for EU/EEA countries in 2011, see Table 3 (ECDC, 2013).

Model input summary

Table 1. Transition probabilities used in the outcome tree

Health outcome (Health state)	Distribution of health states in health outcome	Transition probability	Source/assumption

Hearing loss		0.75%	Edmond, 2010
Cognitive difficulties		0.42%	Edmond, 2010
Seizure disorder		0.25%	Edmond, 2010
Motor deficit		0.58%	Edmond, 2010
Visual disturbance		0.11%	Edmond, 2010
Behavioural problems		0.46%	Edmond, 2010
Clinical impairments		0.33%	Edmond, 2010
Multiple impairments		0.57%	Edmond, 2010
Fatal cases due to symptomatic infection		See Table 3 (3-24%)	Harboe, 2009

Table 2. Disability weights and duration

Health outcome (Health state)	Disability Weight (DW) (Haagsma, 2015)	Duration

	DW	Label	In years	Source
Symptomatic infection	0.655 (0.579-0.727)	Intensive care unit admission	0.027-0.038	Tunkel, 2004<br>Assuming the duration of antimicrobial therapy
Permanent disability following meningitis			Remaining life expectancy	
1. Hearing loss	0.008-0.103	From lowest to highest hearing loss related DWs		
2. Cognitive difficulties	0.044-0.188	From lowest to highest intellectual disability related DWs		
3. Seizure disorder	0.07 (0.057-0.088)	Generic uncomplicated disease: worry and daily medication		
4. Motor deficit	0.011-0.421	From lowest to highest motor impairment related DWs		
5. Visual disturbance	0.004-0.171	From lowest to highest vision impairment related DWs		
6. Behavioural problems	0.088 (0.07-0.108)	Subacute sclerosing panencephalitis – phase 1 (assuming best fitting health state description)		
7. Clinical impairments	0.004-0.421	From lowest to highest DW included in this model		
8. Multiple impairments	0.004-0.421	From lowest to highest DW included in this model		

Table 3. CFP following symptomatic infection

Age	CFR
0	5.1%
1-4	3%
5-14	7.1%
15-64	8%

≥65	14.3%

Table 4. Age specific distribution per gender of the 60-63% risk of developing meningitis manifestation during the symptomatic infection (TESSy 2010-2011)

Age group	%	
	F	M
0	10.37	11.45
01-04	8.13	8.52
05-09	2.70	3.56
10-14	1.54	2.54
15-19	0.39	1.57
20-24	1.29	1.22
25-29	1.02	2.23
30-34	2.45	3.56
35-39	3.29	5.68
40-44	3.74	5.58
45-49	5.47	6.90
50-54	6.70	7.31
55-59	9.21	7.76
60-64	11.28	9.02

65-69	9.78	7.04
70-74	7.60	6.24
75-79	6.51	4.91
80-84	5.15	2.98
85+	3.36	1.93
Total	100	100

References

Burckhardt I, Burckhardt F, Van Der Linden M, Heeg C, Reinert RR. Risk factor analysis for pneumococcal meningitis in adults with invasive pneumococcal infection. Epidemiol Infect 2010;138(9):1353-8.

Buysse CM, Oranje AP, Zuidema E, Hazelzet JA, Hop WC, Diepstraten AF, et al. Long-term skin scarring and orthopaedic sequelae in survivors of meningococcal septic shock. Arch Dis Child. 2009 May;94(5):381-6.

Centers for Disease Control and Prevention. Epidemiology and Prevention of Vaccine-Preventable Diseases. Atkinson W, Wolfe S, Hamborsky J, McIntyre L, eds. 11th ed. Washington DC: Public Health Foundation, 2009.

Edmond K, Clark A, Korczak VS, Sanderson C, Griffiths UK, Rudan I. Global and regional risk of disabling sequelae from bacterial meningitis: a systematic review and meta-analysis. Lancet Infect Dis. 2010 May;10(5):317-28.

Eidelman LA, Putterman D, Putterman C, Sprung CL. The Spectrum of Septic Encephalopathy: Definitions, Etiologies, and Mortalities. JAMA. 1996;275(6):470-473. doi:10.1001/jama.1996.03530300054040.

Elward K, Hruby N, Christy C. Pneumococcal endocarditis in infants and children: Report of a case and review of the literature. Pediatr Infect Dis J. 1990;9:652.

European Centre for Disease Prevention and Control. Surveillance of invasive bacterial diseases in Europe, 2010. Stockholm: ECDC; 2013.

European Centre for Disease Prevention and Control. Surveillance of invasive bacterial diseases in Europe, 2011. Stockholm: ECDC; 2013.

Haagsma JA, Maertens de Noordhout C, Polinder S, Vos T, Havelaar AH, Cassini A, Devleesschauwer B, Kretzschmar ME, Speybroeck N, Salomon JA. Assessing disability weights based on the responses of 30,660 people from four European countries. Population Health Metrics 2015; 13: 10

Harboe ZB, Thomsen RW, Riis A, et al. Pneumococcal Serotypes and Mortality following Invasive Pneumococcal Disease: A Population-Based Cohort Study. PLoS Med. 2009 May; 6(5): e1000081.

Jefferson T, Ferroni E, Curtale F, et al. Streptococcus pneumoniae in western Europe: serotype distribution and incidence in children less than 2 years old. Lancet Infect Dis 2006;6:405-10.

Jit M. The risk of sequelae due to pneumococcal meningitis in high-income countries: a systematic review and meta-analysis. J Infect 2010;61(2):114-24.

Lin SH, Lai CC, Tan CK, Liao WH, Hsueh PR. Outcomes of hospitalized patients with bacteraemic and non-bacteraemic community-acquired pneumonia caused by Streptococcus pneumoniae. Epidemiol Infect. 2010 Oct 26:1-10. [Epub ahead of print].

Rudan I, Campbell H. The deadly toll of S pneumoniae and H influenzae type b. Lancet 2009; 374(9.693):854-6.

Saldías PF, Viviani GP, Pulgar BD, Valenzuela FF, Paredes ES, Díaz PO. Prognostic factors and mortality in immunocompetent adult patients hospitalized with community-acquired pneumococcal pneumonia. Rev Med Chil 2009; 137(12):1.545-52.

Tunkel AR, Hartman BJ, Kaplan SL, Kaufman BA, Roos KL, Scheld WM, et al: Practice guidelines for the management of bacterial meningitis. Clin Infect Dis 2004, 39(9):1267–1284.

Ziaja M. Septic encephalopathy. Curr Neurol Neurosci Rep. 2013; 13: 383

Legionnaires’ disease

Since 2008, the EU case definition focuses solely on Legionnaires’ disease, dismissing Pontiac fever cases. Therefore, the present disease outcome tree focuses only on Legionnaires’ disease and its sequelae.

Legionnaires’ disease is mostly observed in the elderly and conditions associated with immunodeficiency constitute a risk for Legionnaires’.

In rare cases, Legionnaires’ disease may also cause extra-pulmonary symptoms, mainly developing cardiac complications (WHO, 2007). Myocarditis, pericarditis, post-cardiomyotomy syndrome or endocarditis are examples of such manifestations although, according to other studies, most of these complication are related to nosocomial infections (Stout, 1997). Extra-pulmonary manifestations are also often observed in immunocompromised patients. For the purpose of this disease model, we focus on community-acquired Legionnaires’ cases and extra- pulmonary manifestations are excluded.

Legionnaires’ disease causes acute consolidating pneumonia. In most cases, and without testing for the causative agent, pneumonia arising from infection with Legionella pneumophila cannot be distinguished from other types of pneumonia. Symptoms of Legionnaires’ disease are an unproductive cough, chest pain, shortness of breath, myalgia and digestive symptoms such as diarrhoea, vomiting and nausea. Patients may also present neurological symptoms such as confusion or deliria (WHO, 2007).

In many cases, the acute phase requires admission to hospital. Studies have shown that in-patient stays in the hospital vary between eight and 13 days (Lettinga, 2002a; von Baum, 2008). However, it may take more than 90 days to recover to the premorbid health state (Lettinga, 2002a) and roentgenographic clearance can take 2–4 months (Edelstein, 2008). For the model the duration of acute Legionnaires’ disease is set at 8– 13 days, as stated in one European study (Lettinga, 2002a).

We consider three different health states occurring during the acute phase of the disease, mild (outpatient, uncomplicated cases), moderate (hospitalised, complicated cases not admitted to an intensive-care unit) and severe (complicated cases admitted to an intensive care unit). Studies have shown that hospitalisation is required in 69–74% of Legionnaires’ cases (von Baum, 2008; Garcia-Fulgueiras, 2003). We therefore assume that 26–31% of cases will be mild. Moreover, it is shown that 30% of hospitalised cases require a stay in an intensive-care unit (ICU) (Lettinga, 2002b), thus the proportion of complicated cases (not requiring ICU) is set to 46.7–53.2% and those requiring ICU is set to 20.7– 22.2% of all symptomatic infections.

The case-fatality proportion (CFP) differs widely and is associated with the severity level. The CFP for severe cases was found to be higher, ranging from 10 to 30% (Lettinga, 2002b; Benin, 2002; Falco, 1991). In a review conducted by WHO, case-fatality proportions of community- acquired infections ranged from 5 to 10% (WHO, 2007; Benin, 2002; Howden, 2003). The European working group on Legionella infections (EWGLI) suggested a 12% case-fatality in Europe (von Baum, 2008). In our model, CFP for uncomplicated and complicated cases not requiring a stay in an ICU is set at 5–12% and 10–30% for severe cases requiring an ICU.

Risk of complications

Legionnaires’ disease is associated with pulmonary (e.g. severe respiratory failure, pulmonary abscess and pleural empyema), cardiac (e.g. acute pericarditis, myocarditis), neuromuscular (e.g. headache, confusion, fatigue) and renal (e.g. acute renal failure, interstitial nephritis) complications. Multi-organ involvement or septic shock are also possible. In the outcome-tree these complications are not treated separately as they are part of the acute phase of Legionnaires’ disease.

Studies on the long-term sequelae of Legionnaires’ are scarce, however some reported consequences up to two years after the initial infection (Lattimer, 1979). Two studies reported fatigue in 58–81% of cases, concentration problems and memory loss in 6–81%, muscle/joint pain or muscle weakness in 25–79% and post-traumatic stress disorder in 15% (Lattimer, 1979; Lettinga, 2002a). Given the lack of evidence on the causality of Legionnaires’ and the long-term consequences, these were not considered.

Model input summary

Table 1. Transition probabilities and distributions used in the outcome tree

Health outcome (Health state)	Distribution of health states in health outcome	Transition probability	Source/assumption
Symptomatic infection			von Baum, 2008; Garcia-Fulgueiras, 2003; Lettinga, 2002b
(Uncomplicated)	26–31%		
(Complicated)	46.7–53.2%		
(Complicated ICU)	20.7–22.2%		
Fatal cases			Lettinga, 2002b; Benin, 2002; Falco, 1991; WHO, 2007; Benin, 2002; Howden, 2003; von Baum, 2008
(Uncomplicated)		5–12%	
(Complicated)		5–12%	
(Complicated ICU)		10–30%	

Table 2. Disability weights and duration

Health outcome (Health state)	Disability Weight (DW) (Haagsma, 2015)		Duration	
	DW	Label	In years	Source
Symptomatic infection		Infectious disease, acute episode, moderate	0.022–0.036	Lettinga, 2002a; von Baum, 2008
(Uncomplicated)	0.051 (0.039–0.06)	Infectious disease, acute episode, severe		
(Complicated)	0.125 (0.104–0.152)	Intensive care unit admission		
(Complicated ICU)	0.655 (0.579–0.727)			

References

Edelstein PH. Detection of antibodies to Legionella. In Manual of Molecular and Clinical Laboratory Immunology. Edited by Detrick B, Hamilton R, Folds J. Washington, DC: ASM Press; 2006: 468-476

Haagsma JA, Maertens de Noordhout C, Polinder S, Vos T, Havelaar AH, Cassini A, Devleesschauwer B, Kretzschmar ME, Speybroeck N, Salomon JA. Assessing disability weights based on the responses of 30,660 people from four European countries. Population Health Metrics 2015; 13: 10

World Health Organization: and the prevention of legionellosis. Geneva; 2007.

Stout JE, Yu VL. Legionellosis. N Engl J Med 1997, 337:682-687.

Lettinga KD, Verbon A, Nieuwkerk PT, Jonkers RE, Gersons BP, Prins JM, et al. Health-related quality of life and post-traumatic stress disorder among survivors of an outbreak of Legionnaires disease. Clin Infect Dis 2002, 35:11-17.

von Baum H, Ewig S, Marre R, Suttorp N, Gonschior S, Welte T, et al. Community-acquired Legionella pneumonia: new insights from the German competence network for community acquired pneumonia. Clin Infect Dis 2008, 46:1356-1364.

Edelstein PH: Legionnaires’ Disease: History and Clinical findings. In Legionella: Molecular Microbiology. Edited by Heuner K, Swanson M. Portland, Oregan: Caister Academic Press; 2008: 1-17

Garcia-Fulgueiras A, Navarro C, Fenoll D, Garcia J, Gonzalez-Diego P, Jimenez-Bunuales T, et al. Legionnaires' disease outbreak in Murcia, Spain. Emerg Infect Dis 2003, 9:915-921.

Lettinga KD, Verbon A, Weverling GJ, Schellekens JF, Den Boer JW, Yzerman EP, et al. Legionnaires' disease at a Dutch flower show: prognostic factors and impact of therapy. Emerg Infect Dis 2002, 8:1448-1454.

Benin AL, Benson RF, Arnold KE, Fiore AE, Cook PG, Williams LK, et al. An outbreak of travel-associated Legionnaires disease and Pontiac fever: the need for enhanced surveillance of travel-associated legionellosis in the United States. J Infect Dis 2002, 185:237-243.

Howden BP, Stuart RL, Tallis G, Bailey M, Johnson PD. Treatment and outcome of 104 hospitalized patients with Legionnaires' disease. Intern Med J 2003, 33:484-488.

Falco V, Fernandez de Sevilla T, Alegre J, Ferrer A, Martinez Vazquez JM. Legionella pneumophila. A cause of severe community-acquired pneumonia. CHEST Journal 1991, 100:1007-1011.

Lattimer GL, Rhodes LV, 3rd, Salventi JS, Galgon JP, Stonebraker V, Boley S, et al. The Philadelphia epidemic of Legionnaire's disease: clinical, pulmonary, and serologic findings two years later. Ann Intern Med 1979, 90:522-526.

Listeriosis

Acquired listeriosis

Listeriosis is an infection caused by the gram-positive bacterium Listeria monocytogenes. The infection is generally asymptomatic but can become extremely severe in immunocompromised patients, pregnant women and their foetuses/newborn and elderly. The severity of the disease is related to its invasiveness: if the infection is not invasive, it will generally cause mild or no symptoms and therefore no burden (with the exception of acute gastroenteritis if a person ingests a large amount of bacteria). Therefore, it is not surprising that most notified cases are invasive listeriosis diseases, hence complicated ones. In order to estimate the number of complicated cases we referred to the US Centers for Disease Control's 2012 and 2011 Listeriosis Annual Surveillance Summaries (CDC, 2014), reporting 95–97% of cases as invasive, and we applied this to the proportion of complicated symptomatic cases.

Manifestations of listeriosis are meningitis, septicaemia, pneumonia, and gastroenteritis. Based on reports from enhanced surveillance in the Netherlands (Doorduyn, 2006 a,b) and a Gamma distribution used to express the uncertainty, Kemmeren et al. (Kemmeren, 2006) and Haagsma et al. (Haagsma, 2009) estimated the distribution of these health states for acquired listeriosis. However, from a clinical perspective it is conceivable that most cases present a mixed form of the disease and isolates are available from multiple anatomical sites. We therefore defined symptomatic infections as either complicated (invasive) or uncomplicated.

In order to determine those long-term sequelae which are linked only to the manifestation of meningitis, we looked at enhanced surveillance in a few European countries, however data on the risk of developing meningitis during invasive listeriosis disease was inconsistent. Therefore, we referred to CDC enhanced surveillance in the USA from 2007 to 2012 and estimated that 13–18% of invasive (complicated) symptomatic cases would present with meningitis (CDC, 2014).

In the current model, the age-specific case fatality proportion related to listeriosis is derived from cases of acquired listeriosis notified to TESSy from 2009 to 2013 (see Table 3) by all EEA Member States except Bulgaria and Lithuania because they report only aggregate data. The case fatality proportion is applied to complicated cases only.

Perinatal listeriosis

Perinatal listeriosis encompasses both pregnant women and their foetuses or newborns. Of the pregnant women with listeriosis, around two out of three will present with prodromal influenza-like symptoms such as fever, chills and headache. Three to seven days after the prodromal symptoms, the pregnant woman may abort the foetus or have premature labour (Gellin, 1989). To the mother, listeriosis is rarely life- threatening, however, infection in the first trimester of pregnancy may result in spontaneous abortion and, in later stages, in stillbirth or a critically ill newborn (Farber, 1991a). Newborns may present with an early-onset or a late-onset form of listeriosis. Early-onset listeriosis is defined as a case of symptomatic listeriosis in a newborn that is less than seven days old. Early-onset listeriosis is acquired by the foetus prenatally. Newborns with early-onset listeriosis mostly develop sepsis and meningitis (Farber, 1991b; Mylonakis, 2002). Late-onset listeriosis is defined as symptomatic listeriosis in a newborn during the first eight to 28 days of life. In this case, the unborn child is infected during childbirth when passing through the birth canal. Newborns with late-onset listeriosis are usually born healthy and at full term, but are at higher risk of developing meningitis during their first weeks of life (Farber, 1991a).

In the current study, the disease burden for health outcomes of early- and late-onset listeriosis are combined into one category. Based on data reported to TESSy between 2009 and 2013, the case fatality proportion was set to 18.71%.

Risk of complications

Long-term sequelae due to meningitis may occur, and will therefore be considered in the outcome tree. The frequency of other post-infectious complications following listeriosis is low (Haagsma, 2009) and therefore they have been disregarded in the current study.

According to Aouaj et al. (Aouaj, 2002), 20% of all listeriosis cases in their study are perinatal. Therefore, of the 147 cases analysed for long- term outcomes (Aouaj, 2002), we estimated that there were 118 acquired cases (29 perinatal). The study stated that 15 (12.7%) of the total number of acquired listeriosis cases presenting meningitis developed neurological long-term sequelae.

Given that 13–18% of all acute cases present meningitis, the risk of developing neurological long-term sequelae from all cases of complicated acquired listeriosis is 1.65–2.29%.

Similarly, knowing that seven of the 29 perinatal listeriosis cases (24%) developed long-term neurological sequelae and that all acute cases present meningitis, the risk of developing life-long neurological disabilities from a perinatal listeria infection is 24%.

Model input summary

Table 1. Transition probabilities and distributions used in the outcome tree

Health outcome<br>(Health state)	Distribution of health states in health outcome	Transition probability	Source/assumption
Acquired listeriosis			
Symptomatic infection<br>(Uncomplicated)<br>(Complicated)	3–5%<br>95–97%		CDC, 2014

Fatal cases		Age dependent (Table 3)	TESSy 2009–2013
Permanent disability following meningitis		1.65–2.29% of complicated cases	Aouaj, 2002; CDC 2014
Perinatal listeriosis			
Fatal cases		18.71%	TESSy 2009–2013
Permanent disability due to meningitis		24%	Aouaj, 2002

Table 2. Disability weights and duration

Health outcome (Health state)	Disability Weight (DW) (Haagsma, 2015)		Duration	
	DW	Label	In years	Source
Acquired listeriosis				
Symptomatic infection (Uncomplicated) (Complicated)	0.149 (0.12–0.182)<br>0.655 (0.579–0.727)	Diarrhoea, moderate<br>Intensive care unit admission	0.02–0.5	Kemmeren, 2006 Haagsma, 2009;
Permanent disability following meningitis	0.011–0.421	From lowest to highest motor and cognitive difficulties	Remaining life expectancy	
Perinatal listeriosis				
Symptomatic infection	0.655 (0.579–0.727)	Intensive care unit admission	0.02–0.5	Kemmeren 2006 & Haagsma 2009
Permanent disability due to meningitis	0.011–0.421	From lowest to highest motor and cognitive difficulties	Remaining life expectancy	

Table 3. Age-group acquired listeriosis case fatality proportion based on cases and deaths notified to TESSy (2009– 2013)

Age groups	%
0	11.90
1-4	0.00
5-9	5.88
10-14	20.00
15-19	13.16
20-24	1.75
25-29	4.10
30-34	1.39
35-39	8.40
40-44	12.50
45-49	14.08
50-54	16.59
55-59	13.77

60-64	18.16
65-69	15.65
70-74	15.17
75-79	17.83
80-84	17.35
>85	23.15
All ages	15.74

References

Aouaj Y, Spanjaard L, van Leeuwen N, Dankert J. (2002). Listeria monocytogenes meningitis: serotype distribution and patient characteristics in The Netherlands, 1976-95. Epidemiol Infect, 128(3):405-409.

CDC (2014). Centers for Disease Control and Prevention, National Surveillance of Bacterial Foodborne Illnesses (Enteric Diseases) National Listeria Surveillance. Available at: http://www.cdc.gov/nationalsurveillance/listeria-surveillance.html [accessed 2 October 2014].

Doorduyn Y, de Jager CM, van der Zwaluw WK, Wannet WJ, van der Ende A, Spanjaard L, et al. (2006a). Invasive Listeria monocytogenes infections in the Netherlands, 1995-2003. Eur J Clin Microbiol Infect Dis, 25(7):433-442.

Doorduyn Y, de Jager CM, van der Zwaluw WK, Wannet WJ, van der Ende A, Spanjaard L, et al (2006b). First results of the active surveillance of Listeria monocytogenes infections in the Netherlands reveal higher than expected incidence. Euro Surveill, 11(4):E060420 060424.

Doorduyn Y, De Jager CM, Van der Zwaluw WK, Wannet WJB, Heuvelink AE, Van der Ende A, et al. (2007). Intensieve surveillance van Listeria monocytogenes in Nederland, 2006. Infectieziekten Bulletin, 9:308-314.

Farber JM, Peterkin PI (1991a). Listeria monocytogenes, a food-borne pathogen. Microbiol Rev, 55(3):476-511.

Farber JM, Peterkin PI, Carter AO, Varughese PV, Ashton FE, Ewan EP. (1991b). Neonatal listeriosis due to cross-infection confirmed by isoenzyme typing and DNA fingerprinting. J Infect Dis, 163(4):927-928.

Haagsma JA, van der Zanden BP, Tariq L, van Pelt W, van Duynhoven YTHP, Havelaar AH (2009). Disease burden and costs of selected foodborne pathogens in the Netherlands, 2006. Bilthoven: National Institute of Public Health and Environment. RIVM rapport 330331001. Available online at: www.rivm.nl/bibliotheek/rapporten/330331001.pdf

Haagsma JA, Maertens de Noordhout C, Polinder S, Vos T, Havelaar AH, Cassini A, Devleesschauwer B, Kretzschmar ME, Speybroeck N, Salomon JA. Assessing disability weights based on the responses of 30,660 people from four European countries. Population Health Metrics 2015; 13: 10

Kemmeren JM, Mangen MJJ, van Duynhoven YTHP, Havelaar AH. (2006). Priority setting of foodborne pathogens: disease burden and costs of selected enteric pathogens. Bilthoven: National Institute of Public Health and Environment: RIVM rapport 330080001. Available online at: www.rivm.nl/bibliotheek/rapporten/330080001.pdf

Lorber B (1997). Listeriosis. Clin Infect Dis, 24(1):1-9; quiz 10-11.

McLauchlin J, Mitchell RT, Smerdon WJ, Jewell K. (2004). Listeria monocytogenes and listeriosis: a review of hazard characterisation for use in microbiological risk assessment of foods. Int J Food Microbiol, 92(1):15-33.

Mylonakis E, Paliou M, Hohmann EL, Calderwood SB, Wing EJ. (2002). Listeriosis during pregnancy: a case series and review of 222 cases. Medicine (Baltimore), 81(4):260-269.

Measles

According to the US Centers for Disease Control and Prevention (CDC, 2012), approximately 30% of reported symptomatic measles cases have one or more complications. The most important complications are: otitis media (occurring in approximately 10% of infected cases), encephalitis (0.1% of cases), and post-infectious encephalomyelitis (0.1–0.3% of cases). Other complications of acute measles include pneumonia (5–6% of untreated cases; Kabra, 2008; CDC, 1991) and diarrhoea (8%) (CDC, 2012). Convulsions are also a relatively frequent complication (5% of cases; Miller, 1978). Complications during pregnancy occur in up to 30% of women with severe measles (Atmar, 1992).

Complications occurring during the acute phase of the disease may overlap and cannot be treated as independent. Two health states were therefore used in our model: complicated and uncomplicated. We derived the risk of complications from data reported to TESSy between 2006 and 2013. Given the high number of cases notified to TESSy without information on complications and in order to account for this uncertainty we included two scenarios. We estimated the proportion of cases reported as uncomplicated out of the number of known cases as 57.24% (excluding cases for which complications were reported as unknown or left blank). We then added the uncomplicated cases to the unknown and blank and obtained the total number 88.64% (assuming that all unknown and blank cases were uncomplicated).

In the model, the rare permanent disabilities due to otitis media, encephalitis, post-infectious encephalomyelitis and subacute sclerosing panencephalitis (SSPE) (van Steenbergen, 2006) are treated as distinct sequelae.

Otitis media and permanent disability due to otitis media

The health state otitis media occurs in around one in ten cases of acute measles and can result in permanent hearing loss (CDC, 2011). The probability of developing permanent disability due to otitis media is 0.01% (CDC, 1991) of all cases of otitis media, therefore the overall risk of developing a permanent disability has been set to 0.001%.

Encephalitis and permanent disability due to encephalitis

Encephalitis occurs in approximately 0.1% of acute symptomatic cases (Weissbrich, 2003; Beutels, 2002; Miller, 1957). Long-term sequelae of measles encephalitis are reported to occur in 20–30% of measles-related encephalitis cases (Beutels, 2002; Filia, 2007); therefore the transition probability for the health outcome 'permanent disability due to encephalitis' was set to 0.02-0.033%.

Encephalitis of the delayed type (Barthez Carpentier, 1992) can occur after acute illness in immunocompromised patients and may occur after asymptomatic infection (Kidd, 2003). Because of the specific population affected, and its relative rarity, the outcome tree was not modified accordingly.

Post-infectious encephalomyelitis (PIE) and permanent disability due to PIE

Post-infectious encephalomyelitis occurs in 1–3 per 1 000 infected persons, usually three to ten days after the onset of rash. Higher rates of PIE due to measles occur in adolescents and adults than in school-aged children (Perry & Halsey, 2004). The condition is associated with demyelination and is thought to have an autoimmune basis. A total of 33% of those afflicted with PIE who survive have lifelong neurological sequelae, including severe retardation, motor impairment, blindness and sometimes hemiparesis (Perry & Halsey, 2004). The transition probability in the model for developing the health outcome 'permanent disability due to PIE' was set to the range 0.033–0.1%.

Subacute sclerosing panencephalitis (SSPE)

On average, the symptoms of SSPE begin seven to ten years after measles infection, but they can appear anytime from one month to 27 years after infection (CDC, 2012).

Various estimates are available for the proportion of cases that develop the SSPE health outcome. SSPE is observed at a rate of 1 per 10 000– 20 000 (Weissbrich, 2003; Takasu, 2003; Bellini, 2005; Garg, 2008). In children who have previously had natural measles, the risk of developing SSPE is between 0.6 and 2.2 per 100 000 cases (Hosoya, 2006). Other estimates include: one SSPE case in every 100 000 cases of measles (Rezende, 1989); 4–11 cases of SSPE per 100 000 cases of measles (CDC, 2009); one in every 25 000 measles infections (Miller, 2004); one in 8 000 for children under two years (Miller, 1992; 2004) and a 16-fold greater risk for those infected under one year of age compared with those over five years (Miller, 1992). The risk of developing SSPE is known to be age-specific (Beutels, 2002; Farrington, 1991; Miller, 2004; CDC, 2012).Therefore, transitional probabilities in the model were also specified as age-dependent (see Table 3) (Beutels, 2002). In the model, the duration for this health outcome was specified as one to two years (CDC, 2012). In the model the transition probability from SSPE to death was set to 100%.

Case fatality proportion

Measles is fatal in approximately 0.05–0.1% of cases (Wolfson, 2007; Lozano, 2012). The risk of death is higher among young children and adults (CDC, 2012). According to CDC (CDC, 2012), the most common causes of death are pneumonia in children and acute encephalitis in adults, but due to the lack of specific data for different age groups we applied the same CFP for all the same age groups.

Model input summary

Table 1. Transition probabilities used in the outcome tree

Health outcome (Health state)	Distribution of health states in health outcome	Transition probability	Source/assumption

Symptomatic infection<br><br>(Complicated)<br>(Uncomplicated)	 <br>11.36-42.76%<br>57.24-88.64%		TESSy, 2006–2013
Permanent disability following otitis media		0.001%	CDC, 1991
Permanent disability following encephalitis		0.02–0.033%	Beutels, 2002; Filia, 2007
Permanent disability following PIE		0.033–0.1%	Perry & Halsey, 2004
SSPE		See Table 3	Beutels, 2002
Fatal cases following SSPE		100%	
Fatal cases following symptomatic infection		0.05–0.1%	Wolfson, 2007; Lozano, 2012

Table 2. Disability weights and duration

Health outcome (Health state)	Disability Weight (DW) (Haagsma, 2015)		Duration	
	DW	Label	In years	Source
Symptomatic infection<br>(Complicated)<br>(Uncomplicated)	 <br>0.125 (0.104-0.152)<br>0.051 (0.039-0.06)	 <br>Infectious disease, acute episode, severe<br>Infectious disease, acute episode, moderate	0.03	Kwong, 2012
Permanent disability due to otitis media	0.008-0.103	From lowest to highest hearing loss related DWs	Remaining life expectancy	
Permanent disability due to encephalitis	0.054-0.425	From lowest to highest Motor plus cognitive impairments related DWs	Remaining life	

			expectancy	
Permanent disability due to PIE	0.054-0.425	From lowest to highest Motor plus cognitive impairments related DWs	Remaining life expectancy	
Latency period before SSPE	0		0.082–27	CDC, 2012
SSPE	0.276 (0.088-0.543)	From Phase 1 to Phase 3 (median is Phase 2) of subacute sclerosing panencephalitis related DWs	1–2	CDC, 2012

Table 3. Transition probabilities subacute sclerosing panencephalitis (SSPE)

Age	%
0-4	0.0081
5-9	0.0011
≥10	0.0010

References

Barthez Carpentier MA, Billard C, Maheut J, Jourdan ML, Degenne D, Ruchoux MM, et al. Acute measles encephalitis of the delayed type: neuroradiological and immunological findings. Eur Neurol. 1992;32(4):235-7.

Bellini WJ, Rota JS, Lowe LE, et al (2005). Subacute sclerosing panencephalitis: More cases of this fatal disease are prevented by measles immunization than previously recognized. J Infect Dis 192:1686–1693.

Beutels P, Van Damme P, Van Casteren V, Gay NJ, De Schrijver K, Meheus A. The difficult quest for data on "vanishing" vaccine-preventable infections in Europe: the case of measles in Flanders (Belgium). Vaccine. 2002 Oct 4;20(29-30):3551-9.

Centers for Disease Control (CDC). Measles: United States, 1990. MMWR Morb Mortal Wkly Rep 1991;40:369–372.

Centers for Disease Control and Prevention. Epidemiology and Prevention of Vaccine-Preventable Diseases. Atkinson W, Wolfe S, Hamborsky J, McIntyre L, eds. 11th ed. Washington DC: Public Health Foundation, 2009

Centers for Disease Control and Prevention (CDC). The Pink Book: Course Textbook - 12th Edition Second Printing (May 2012).

Farrington CP. Subacute sclerosing panencephalitis in England and Wales: transient effects and risk estimates. Stat Med 1991;10:1733–1744;

Filia A, Brenna A, Panà A, Maggio Cavallaro G, Massari M, Ciofi degli Atti LM. Health burden and economic impact of measles-related hospitalizations in Italy in 2002–2003. BMC Public Health. 2007; 7: 169

Garg RK (2008). Subacute sclerosing panencephalitis. J Neurol 255(12):1861–1871.

Haagsma JA, Maertens de Noordhout C, Polinder S, Vos T, Havelaar AH, Cassini A, Devleesschauwer B, Kretzschmar ME, Speybroeck N, Salomon JA. Assessing disability weights based on the responses of 30,660 people from four European countries. Population Health Metrics 2015; 13: 10

Hosoya M. Measles encephalitis: direct viral invasion or autoimmune-mediated inflammation? Intern Med. 2006;45(14):841-2.

Kabra SK, Lodha R, Hilton DJ. Antibiotics for preventing complications in children with measles. Cochrane Database of Systematic Reviews 2008, Issue 3. Art. No.: CD001477. DOI: 10.1002/14651858.CD001477.pub3.

Kwong JC, Ratnasingham S, Campitelli MA, Daneman N, Deeks SL, et al. (2012) The Impact of Infection on Population Health: Results of the Ontario Burden of Infectious Diseases Study. PLoS ONE 7(9): e44103. doi:10.1371/journal.pone.0044103

Lozano R, Naghavi M, Foreman K, Lim S, Shibuya K, Aboyans V, et al. (Dec 15, 2012). Global and regional mortality from 235 causes of death for 20 age groups in 1990 and 2010: a systematic analysis for the Global Burden of Disease Study 2010. Lancet 380 (9859): 2095–128

Miller HG, Stanton JB, Gibbons JL. Acute disseminated encephalomyelitis and related syndromes. British medical journal.1957 Mar 23;1(5020):668-72

Miller CL. Severity of notified measles. British Medical Journal. 1978; 1(6122): 1253-1255.

Miller C, Andrews N, Rush M, Munro H, Jin L, Miller E. The epidemiology of subacute sclerosing panencephalitis in England and Wales 1990-2002. Arch Dis Child. 2004 Dec;89(12):1145-8.

Murray CJL, Lopez AD, editors. The global burden of disease: a comprehensive assessment of mortality and disability from diseases, injuries, and risk factors in 1990 and projected to 2020. Cambridge: Harvard University Press, 1996.

Rezende MA. Nursing care and the neurological sequelae of measles. Revista da Escola de Enfermagem USP. 1989 Apr;23(2):141-8.

Stein CE, et al, The Global Burden of Measles in the Year 2000—A Model that Uses Country-Specific Indicators. Journal of Infectious Diseases 2003; 187(Suppl 1):S8–14.

Takasu T, Mgone JM, Mgone CS, et al (2003). A continuing high incidence of subacute sclerosing panencephalitis (SSPE) in the eastern highlands of Papua New Guinea. Epidemiol Infect 131:887–898.

Tishler M, Abramov AL. Liver involvement in measles infection of young adults. Isr J Med Sci. 1983 Sep;19(9):791-3.

Wolfson LJ, Strebel PM, Gacic-Dobo M, Hoekstra EJ, McFarland JW, et al. (2007) Has the 2005 measles mortality reduction goal been achieved? A natural history modelling study. Lancet 369: 191–200

Zhou F, Reef S, Massoudi M, Papania MJ, Yusuf HR, Bardenheier B, et al. An Economic Analysis of the Current Universal 2-Dose Measles-Mumps- Rubella Vaccination Program in the United States. The Journal of Infectious Diseases. 2004 May 1;189 Suppl 1:S131-45.

Mumps

Mumps is symptomatic in 80% of infections (CDC, 2012), the main symptom being parotitis.

Risk of complications

The principal complications with mumps are orchitis, oorphoritis, meningitis, pancreatitis, and encephalitis.

Epididymo-orchitis occurs in 15–30% of adult men with mumps infection, but it is rare before puberty (Hviid, 2008). Oophoritis (ovarian inflammation), the counterpart of orchitis in females, is associated with pelvic pain and tenderness. It occurs in 5% of post-pubertal females (CDC, 2009).

Mumps meningitis is a benign entity with no significant risk of mortality or long-term sequelae. Even though cerebrospinal fluid pleiocytosis occurs in about half of the patients with mumps, clinical manifestations of meningitis arise in 1–10% of the cases (Hviid, 2008), and long-term morbidity is rare. Encephalitis occurs in 0.1% of acute cases (Hviid, 2008).

Acute pancreatitis, with symptoms of abdominal distention and pain, fever, nausea, and vomiting (Demirci, 2011), occurs in approximately 4% of mumps cases (Vanlioglu & Chua, 2011).

With mumps, the acute complications of symptomatic infections are considered as a single health state (complicated) because they can occur concomitantly.

Of all mumps infections, 40–50% may have only non-specific or primarily respiratory symptoms (CDC, 2012). Therefore, knowing that 20% of infections are asymptomatic, 32–40% of symptomatic cases were considered to be uncomplicated. Durations were set to 7–10 days for the uncomplicated cases and 7–14 days for the complicated ones.

Permanent deafness caused by mumps occurs with an estimated frequency of one in 20 000 cases (0.005%) and in 80% of the cases, hearing loss is monolateral (Hviid, 2008).

Case fatality proportion

Death is very rare in mumps cases and the mortality rate following encephalitis is 1.5%. Therefore, 0.15% was used in the model for the risk of death resulting from all symptomatic infections. More than half of fatalities occur in patients over 19 years (Hviid, 2008; Demirci, 2011). This age distribution also applies to the symptomatic complicated cases (see Table 3).

Model input summary

Table 1. Transition probabilities used in the outcome tree

Health outcome (Health state)		Distribution of health states in health outcome	Transition probability	Source/assumption

Symptomatic infection<br>(Uncomplicated)<br>(Complicated)	32–40%<br>60–68%		CDC, 2012
Permanent disability due to hearing loss		0.005%	Hviid, 2008
Fatal cases		0.15%<br>Age dependent<br>(see Table 3)	Hviid, 2008<br>Assuming 1.5% of encephalitis cases (0.1%) become fatal

Table 2. Disability weights and duration

Health outcome (Health state)	Disability Weight (DW) (Haagsma, 2015)		Duration	
	DW	Label	In years	Source
Symptomatic infection<br>Uncomplicated<br>Complicated	0.051 (0.039-0.06)<br>0.125 (0.104-0.152)	Infectious disease, acute episode, moderate<br>Infectious disease, acute episode, severe	0.019-0.027<br>0.019-0.038	Hviid, 2008
Permanent hearing loss	0.008 (0.005-0.012)	Unilateral hearing loss	Remaining life expectancy	Hviid, 2008

Table 3. Age distribution – case fatality ratio

0-19	50
≥20	50

References

Centers for Disease Control and Prevention. Epidemiology and Prevention of Vaccine-Preventable Diseases. Atkinson W, Wolfe S, Hamborsky J, eds. 12th ed., second printing. Washington DC: Public Health Foundation, 2012.

Demirci CS, Abuhammour W, Quintana EC, Shahidi H, Wilkes G. Mumps. Available from http://emedicine.medscape.com/article/966678- overview [accessed 12 September, 2011].

Haagsma JA, Maertens de Noordhout C, Polinder S, Vos T, Havelaar AH, Cassini A, Devleesschauwer B, Kretzschmar ME, Speybroeck N, Salomon JA. Assessing disability weights based on the responses of 30,660 people from four European countries. Population Health Metrics 2015; 13: 10

Hviid A, Rubin S, Mühlemann K. Mumps. Lancet. 2008 Mar 15;371(9616):932-44.

Mandell GL, Bennet JE, Dolin R. Mandell, Douglas and Bennett's Principles and Practice of Infectious Disease 5th edition. Churchill Livingstone, 1999.

Vanlioglu B, Chua TC. Presentation of mumps infection as acute pancreatitis without parotitis. Pancreas. 2011 Jan;40(1):167-8.

Pertussis

Pertussis is principally toxin-mediated. Toxins paralyse the cilia of the respiratory tract cells, leading to the clinical features and complications of the disease. The clinical course of the illness is divided into three stages. The first one is the catarrhal stage, characterised by coryza, sneezing, low-grade fever and a mild, occasional cough. The cough gradually becomes more severe, and after 1–2 weeks, the paroxysmal stage begins, usually lasting one to six weeks. In the convalescent stage, which lasts two to three weeks, recovery is gradual and the cough becomes less paroxysmal. However, paroxysms often recur for many months after the onset of pertussis (CDC, 2009; Mandell, 1999).

Clinical manifestations of pertussis may be mild in adults and vaccinated children. Around 20% of infected persons develop mild/asymptomatic disease (Rothstein, 2005). Based on this finding, an asymptomatic proportion of 20% was specified in the model.

Risk of complications

The principal complications of pertussis are secondary infections, such as otitis media and pneumonia, neurological complications, such as seizures and encephalopathy. Other possible complications include physical sequelae of paroxysmal cough (e.g. subconjunctival haemorrhages, epistaxis, petechiae, central nervous system haemorrhage, pneumothorax and hernia) (CDC, 2009; Mandell, 1999).

Pneumonia can result from aspiration during whooping and vomiting or from impaired clearance mechanisms. It occurs in 5.2% of all patients (CDC, 2009), in up to 25% of cases reported in infants (Mandell, 1999), in 2–4% of individuals aged 10–19 years, in 2.7–5.5% of those over 20 years and in 5–9% of those over 30 years (Rothstein, 2005).

Approximately 4% of adolescents and adults with symptomatic pertussis infection develop otitis media (De Serres, 2000).

Neurological complications of pertussis are more common among infants. In children 12 months of age or younger with pertussis in the USA (1980–1989), convulsions occurred in 3.0% and encephalopathy in 0.9% of cases. Encephalopathy, febrile and afebrile convulsions occur infrequently in adults with pertussis (CDC, 2009), with encephalopathy observed in 0.1% of cases during the period 1997–2000 (CDC, 2009).

Seizures were reported among 0.8% of all pertussis cases in the period 1997–2000 (CDC, 2009).

Infants with pertussis are at greater risk of complications and permanent sequelae, however complications of pertussis, including serious ones, are not uncommon in adolescents and adults, especially the elderly. Complications occur in up to 23% of patients aged 19–83 years. Complications are more frequent in adults than in adolescents (28% compared to 16%) (CDC, 2009; Mandell, 1999; Rothstein, 2005).

Most complications occurring during the symptomatic acute disease phase overlap with one other. We therefore decided to aggregate all complicated cases into one health state. Risk of complications is reported to be 50% in infants (<1 year), 16% in children and adolescents and 28% in cases 20 years (CDC, 2013).

We assumed that in complete and active surveillance systems, those cases notified represent the complicated cases of pertussis. The United Kingdom has an enhanced surveillance system for pertussis where information is compiled from different sources. We therefore chose to consider the number of cases reported in the UK (2007–2013) as complicated. In order to estimate the proportion of complicated cases, we divided the number of cases reported in the UK by the estimated true incidence of pertussis derived from the literature: 71–507 per 100 000 10 years; 46 per 100 000 <10 years (Wirsing von Konig, 2002; Diez-Domingo, 2004) (see Table 3).

Case fatality proportion

Death from pertussis is rare beyond the age of 10 years, occurring in less than 0.1% of all cases, with older adults being at greater risk than younger adults (Rothstein, 2005). Pneumonia is a leading cause of death, but in a study of 99 patients aged 55–94 years who died of pertussis (Rothstein, 2005), intracranial haemorrhage was the cause of death for two of the four deaths thought to be associated with pertussis. Among patients who died, apnoea, pneumonia, seizures, and encephalopathy were reported for 58% (40 of 69), 54% (39 of 72), 21% (14 of 68), and 12% (7 of 57), respectively (Rothstein, 2005; Farizo, 1992).

‘The case fatality proportion in the United States between 1990 and 1996 was 0.2%. Eighty-four per cent of pertussis-related deaths occur in infants younger than six months of age’ (Ratnapalam, 2005).

In general, we considered that only complicated cases were at risk of dying. We used the CFP reported in the UK for deaths of infants <1 year old because of its comprehensive surveillance system, compiling data from different sources and deemed to be capturing approximately 94% of the cases in recent capture-recapture studies. There were 33 deaths due to pertussis reported to TESSy between 2007 and 2013 out of 1 791 cases. This resulted in a CFP of 1.84% which was applied to complicated cases <1 year.

We chose 0.1% of complicated cases for all other age groups.

Model input summary

Table 1. Transition probabilities used in the outcome tree

Health outcome<br>(Health state)	Distribution of health states in health outcome	Transition probability	Source/assumption
Symptomatic infection<br>(Complicated)<br>(Uncomplicated)	Age dependent (see Table 3)<br>Remaining cases		CDC, 2013

Fatal cases		1.84% <1 yr.<br>0.1% ≥ 1 yr.	TESSy<br>Rothstein, 2005

Table 2. Disability weights and duration

Health outcome (Health state)	Disability Weight (DW) (Haagsma, 2015)		Duration	
	DW	Label	In years	Source
Symptomatic infection			0.077–0.211	CDC, 2009; Mandell, 1999
(Complicated)	0.125 (0.104–0.152)	Infectious disease, acute episode, severe		
(Uncomplicated)	0.051 (0.039–0.06)	Infectious disease, acute episode, moderate		

Table 3. Risk of complications

Age	%	
	Estimated from low true incidence	Estimated from high true incidence
0	28.04	
01-04	8.04	
05-09	5.85	
10-14	0.35	2.46
15-19	0.39	2.81
20-24	1.05	7.50
25-29	1.59	11.38
30-34	1.92	13.68
35-39	1.45	10.32
40-44	1.84	13.12
45-49	2.23	15.96
50-54	2.00	14.29
55-59	1.68	11.97

60-64	1.20	8.57
65-69	1.48	10.58
70-74	1.24	8.83
75-79	1.30	9.26
80-84	0.91	6.52
85+	0.54	3.88

References

Centers for Disease Control and Prevention. Epidemiology and Prevention of Vaccine-Preventable Diseases. Atkinson W, Wolfe S, Hamborsky J, McIntyre L, eds. 11th Edition. Washington DC: Public Health Foundation, 2009.

Centers for Disease Control and Prevention. http://www.cdc.gov/pertussis/about/complications.html Last update January 2013 [accessed 25 September 2014].

De Serres G, Shadmani R, Duval B, Boulianne N, Déry P, Douville Fradet M, et al. Morbidity of pertussis in adolescents and adults. J Infect Dis. 2000 Jul;182(1):174-9.

Diez-Domingo J, Ballester A, Baldo JM, Planelles MV, Villarroya JV, Alvarez T et al. Incidence of pertussis in persons < 15 years of age in Valencia, Spain: seroprevalence of antibodies to pertussis toxin (PT) in children, adolescents and adults. Journal of Infection (2004) 49, 242- 247

Farizo KM, Cochi SL, Zell ER, Brink EW, Wassilak SG, Patriarca PA. Epidemiological features of pertussis in the United States, 1980-1989. Clin Infect Dis. 1992 Mar;14(3):708-19.

Haagsma JA, Maertens de Noordhout C, Polinder S, Vos T, Havelaar AH, Cassini A, Devleesschauwer B, Kretzschmar ME, Speybroeck N, Salomon JA. Assessing disability weights based on the responses of 30,660 people from four European countries. Population Health Metrics 2015; 13: 10

Mandell GL, Bennet JE, Dolin R. Mandell, Douglas and Bennett's Principles and Practice of Infectious Disease 5th edition. Churchill Livingstone, 1999.

Ratnapalan S, Parkin PC, Allen U. Case 1: The deadly danger of pertussis. Paediatr Child Health. 2005 Apr;10(4):221-2

Rothstein E, Edwards K. Health burden of pertussis in adolescents and adults. Pediatr Infect Dis J. 2005 May;24(5 Suppl):S44-7. Wirsing von Konig CH, Halperin S, Riffelmann M, Guiso N. Pertussis of adults and infants. Lancet Infect Dis 2002; 2: 744-50.

Poliomyelitis

Poliomyelitis is an acute illness which may result from invasion of the gastro-intestinal tract by one of three types of polio virus. It usually affects small children under the age of three years. The virus is transmitted through contaminated food and water, and multiplies in the intestine, from where it can invade the nervous system. Transmission occurs through contact with faeces or pharyngeal secretions of an infected person. The incubation period ranges from three to 21 days, but may be longer. Cases are infectious from about ten days before to seven days after the onset of symptoms; however, carriers and some immuno-compromised persons may shed the virus in faeces for longer than six weeks (Howard, 2005).

Most infections are not clinically apparent; up to 95% of infections are asymptomatic (CDC, 2009).

Risk of complications

Clinical disease may range in severity from minor illness (abortive poliomyelitis), to non-paralytic poliomyelitis (aseptic meningitis) and paralytic poliomyelitis (Feigin, 2009).

Approximately 4–8% of polio infections consist of a non-specific 'minor illness' without clinical or laboratory evidence of central nervous system invasion (CDC, 2009; Feigin, 2009). This clinical presentation is known as abortive poliomyelitis, and is characterised by complete recovery in less than one week (CDC, 2009).

Nonparalytic aseptic meningitis (symptoms of stiffness of the neck, back, and/or legs) which usually follows several days after a prodrome similar to that of a minor illness, occurs in 1–2% of polio infections (CDC, 2009). Increased or abnormal sensations can also occur. Typically these symptoms will last from two to ten days, followed by complete recovery (CDC, 2009).

Less than 1% of all polio infections result in flaccid paralysis (CDC, 2009; Heymann, 2004). Paralytic symptoms generally begin one to ten days after prodromal symptoms and progress for two to three days. Generally, no further paralysis occurs after fever subsides (CDC, 2009). Many patients with paralytic poliomyelitis recover completely and, in most of them, muscle function returns to some degree. Weakness or paralysis 12 months after onset is usually permanent (CDC, 2009).

In acute flaccid paralysis (AFP), the legs are usually more often affected than the muscles of the upper body. However, the polio virus may invade the brain stem, potentially leading to breathing difficulty and even death. Symptoms include headache, gastro-intestinal disturbance, malaise and stiffness of the neck and back, with or without paralysis (American Academy of Pediatrics, 2006; Shibuya & Murray, 2002). Improvements are seen within the first six months (Farbu, 2013; Neumann, 2004). The principal complication is painful, acute, asymmetric paralysis of the arms or the legs, reaching its maximum extent over the course of three to four days and leading to permanent lameness of the affected limbs and breathing difficulties (UK Department of Health, 2006; WHO, 2014).

Given the estimates of symptomatic polio cases, we considered that on average 8.5% of infections are symptomatic (6–11%; CDC, 2011); hence 70.59% of cases on average will be abortive (uncomplicated), 17.65% will be non-paralytic and 11.76 will be paralytic.

According to WHO (WHO, 2014), 1 in 200 infections leads to irreversible paralysis. Given that 1% of all infections has a paralytic form, we considered that 50% of all paralytic forms would develop a permanent disability due to paralysis.

Post-polio syndrome is a long-term sequela that occurs 30–35 years after infection in approximately 25–50% of cases (Jubelt & Drucker, 1999). A slowly progressing condition, it can also occur in patients who have had the non-paralytic form of poliomyelitis. The most common symptoms include slow, progressive muscle weakness, fatigue (both generalised and muscular) and a gradual decrease in the size of muscles (muscle atrophy). Pain from joint degeneration and increasing skeletal deformities such as scoliosis (curvature of the spine) is common and may precede the weakness and muscle atrophy. Some individuals experience only minor symptoms while others develop visible muscle weakness and atrophy. Fatigue is clearly the most prominent manifestation, occurring in up to 80% of patients (Jubelt & Drucker, 1999). Post-polio syndrome is rarely life-threatening (NINDS, 2012).

Case fatality proportion

The case fatality proportion is 5–10% of paralytic forms (WHO, 2014).

Model input summary

Table 1. Transition probabilities used in the outcome tree

Health outcome (Health state)	Distribution of health states in health outcome	Transition probability	Source/assumption
Symptomatic infection<br>(Uncomplicated)<br>(Non-paralytic poliomyelitis)<br>(Paralytic poliomyelitis)	70.59%<br>17.65%<br>11.76%	6-11%	CDC, 2009<br>CDC, 2009; Heymann, 2004
Post-polio syndrome		25–50%	Jubelt & Drucker, 1999
Permanent disability following paralytic poliomyelitis		50%	WHO, 2014

Fatal cases following paralytic poliomyelitis		5-10%	WHO, 2014

Table 2. Disability weights and duration

Health outcome (Health state)	Disability Weight (DW) (Haagsma, 2015)		Duration	
	DW	Label	In years	Source
Symptomatic infection				
(Uncomplicated)	0.007 (0.005–0.01)	Infectious disease, acute episode, mild	0.019	CDC, 2009
(Non-paralytic poliomyelitis)	0.051 (0.039–0.06)	Infectious disease, acute episode, moderate	0.005–0.027	CDC, 2009
(Paralytic poliomyelitis)	0.125 (0.104–0.152)	Infectious disease, acute episode, severe	0.011–0.038	CDC, 2009
Permanent disability following paralytic poliomyelitis	0.067 (0.054–0.081)	Spinal cord lesion below neck level (treated)	Remaining life expectancy	
Latency period before PPS	0		30–35	Jubelt & Drucker, 1999
Post-polio syndrome (PPS)	0.344 (0.3–0.391)	Musculoskeletal problems, generalised, moderate	Remaining life expectancy	

References

American Academy of Pediatrics. Red Book: 2006 Report of the Committee on Infectious Diseases. 27th ed. Elk Grove Village. 2006.

Centers for Disease Control and Prevention (CDC). Epidemiology and Prevention of Vaccine-Preventable Diseases. Atkinson W, Wolfe S, Hamborsky J, McIntyre L, eds. 11th ed. Washington DC: Public Health Foundation, 2009.

Department of Health, UK. Immunisation against infectious disease (the Green Book). 3rd ed. London: The Stationery Office. 2006.

Feigin R, Cherry J, Demmler-Harrison GJ, Kaplan S. Feigin and Cherry's Textbook of Pediatric Infectious Diseases. Sixth Edition, 2009.

Farbu E. 2013. Post-polio Syndrome. In: JH Stone, M Blouin, editors. International Encyclopedia of Rehabilitation. Available online: http://cirrie.buffalo.edu/encyclopedia/en/article/138/

Haagsma JA, Maertens de Noordhout C, Polinder S, Vos T, Havelaar AH, Cassini A, Devleesschauwer B, Kretzschmar ME, Speybroeck N, Salomon JA. Assessing disability weights based on the responses of 30,660 people from four European countries. Population Health Metrics 2015; 13: 10

Heymann D, Aylward B. Poliomyelitis. Orphanet Encyclopedia. WHO. 2004.

Howard RS. Poliomyelitis and the post polio syndrome. BMJ 2005;330(7503):1314–1318.

Jubelt B, Drucker J. Poliomyelitis and the Post-Polio Syndrome in Motor Disorders. Philadelphia: Lippincott Williams and Wilkins. 1999. pp. 381.

Neumann D. Polio: its impact on the people of the United States and the emerging profession of physical therapy (PDF).The Journal of Orthopaedic and Sports Physical Therapy 2004; 34 (8): 479–92. PMID 15373011

Post-Polio Syndrome Fact Sheet. NINDS. 2012.

Shibuya K, Murray CJL. Poliomyelitis. In: Stein CE, Murray CJL, Lopez AD (eds). The Global Burden of Disease and Injuries Series, Volume 4: The Global Epidemiology of Infectious Diseases. WHO, 2002.

UNICEF Executive Board Special (2009). Focus Session on Global Health, with particular focus on polio eradication. Available online from: http://www.unicef.org/about/execboard/files/Special_focus_session-Background_SFS_English_8_June_09(1).pdf

World Health Organization (WHO). Poliomyelitis. Available from http://www.who.int/topics/poliomyelitis/en/ [accessed 19 September 2014].

Q fever

Q fever infection becomes symptomatic in 40% of cases (Dijkstra, 2012). Symptomatic infections are divided into two health states: uncomplicated and complicated (more severe cases) and the proportion of complications is based on the hospitalisation rate (2–5%) for Q fever (Maurin & Raoult, 1999; Raoult, 2005).

Around 1–2% of Q fever cases are fatal (ECDC, 2010). This CFR is applied to complicated cases only, based on the US Centers for Disease Control (CDC) Fact Sheet which states that 'the case fatality ratio for hospitalized patients is under 2%' (CDC, 2013).

Chronic Q fever

The transition probability that cases with symptomatic infections will develop chronic Q fever is set to 1.6% (1.5–2%) (van der Hoek, 2011; ECDC, 2010). Due to the lack of evidence, development of chronic Q fever was not associated with asymptomatic Q fever (ECDC, 2010). The average duration of chronic Q fever before developing symptoms is 0.5 years (0.08–1.5 years) (Fenollar, 2001) and this is included in the burden calculation as it reduces the life expectancy of later health outcomes.

Taking the duration of treatment as a proxy for the duration of chronic Q fever, we set the duration to 12–18 months (CDC, 2013) although there are studies recommending life-long treatment which could vary from one year to a person's entire lifespan (Forland, 2012). However, we assume that symptoms due to the infection resolve during the treatment; if symptoms continue, we consider them not to be associated with the Q fever infection but with underlying conditions.

The most common manifestation of chronic Q fever is heart failure, of which a quarter of cases show conduction disorders (Marrie, 2010); other possible manifestations include vascular and pulmonary infections and chronic hepatitis (Maurin & Raoult, 1999). Therefore disability weights describing heart failure were applied.

The case fatality proportion for chronic Q fever has been estimated to be from to 5 to 50%, according to time of diagnosis and onset of treatment (ECDC, 2010).

Post-infectious fatigue syndrome

Follow-up studies after large outbreaks provide some information regarding duration and the probability of developing post-infectious fatigue syndrome. One large cohort following an outbreak in the UK used standard clinical criteria to quantify the proportion of patients developing fatigue after five years (Ayres JG, 1998) and ten years (Wildman, 2002). The first follow-up reported a larger proportion of idiopathic chronic fatigue (ICF) in Q fever cases (42.3%) than in matched controls (26%), with a difference of 16.3%. At the 10-year follow-up point, cases were matched to controls for the presence of comorbidities and hospital attendance, but there was still a higher proportion of ICF (21.6% vs. 5.4%), with a difference of 16.2%. A recent study from a Dutch outbreak indicates the proportion of patients with fatigue after 12 to 26 months to be higher (43.5%) than after five or ten years of follow-up (Morroy, 2011). Therefore, two health states were specified in order to differentiate short-term fatigue (43.5–16.2/16.3% = 27.2/27.3%) from long-term fatigue (16.2–16.3%). The short-term health state consists of clinical cases that recover within 12 to 26 months; severe cases are assumed to recover after 10 years. Regarding the sources of post-infectious fatigue syndrome (PFS), it is surprising that after 10 years the proportion of PFS is reduced to the same extent in controls as in the cases. We therefore considered the bias to be prevalent and decided to exclude PFS from the model.

Model input summary

Table 1. Transition probabilities and distributions used in the outcome tree

Health outcome	Distribution of health states in health outcome	Transition probability			Source/assumption

(Health state)			
Symptomatic infection<br><br>(Mild)<br><br>(Severe)	95–98%<br><br>2–5%		Maurin & Raoult, 1999; Raoult, 2005
Chronic Q fever		1.6% (1.5–2%)	van der Hoek, 2011;<br>ECDC, 2010
Fatal cases following symptomatic infection		1-2% of severe cases	ECDC, 2010
Fatal cases following chronic infection		5-50%	ECDC, 2010

Table 2. Disability weights and duration

Health outcome (Health state)	Disability Weight (DW) (Haagsma, 2015)		Duration	
	DW	Label	In years	Source
Symptomatic infection<br><br>(Mild)	0.007 (0.005-0.01)<br><br>0.125 (0.104-0.152)	Infectious disease, acute episode, mild	0.038<br><br>0.038	Stouthard, 1997<br><br>Stouthard, 1997

(Severe)		Infectious disease, acute episode, severe		
Latency period (before chronic Q fever)			0.5 (0.08-1.5)	Fenollar, 2001
Chronic Q fever	0.173 (0.14-0.205)	Heart failure, severe	1-1.5	CDC, 2013

References

Ayres JG, Flint N, Smith EG, Tunnicliffe WS, Fletcher TJ, Hammond K, et al. Post-infection fatigue syndrome following Q fever. QJM: Monthly Journal of the Association of Physicians, 1998 Feb;91(2):105-23.

Dijkstra F, van der Hoek W, Wijers N, Schimmer B, Rietveld A, Wijkmans CJ, et al. The 2007–2010 Q fever epidemic in the Netherlands: Characteristics of notified acute Q fever patients and the association with dairy goat farming. FEMS Immunology and Medical Microbiology. 2012;64(1):3-12 (epub 2011/11/10).

ECDC Technical Report – Risk Assessment on Q fever. May 2010. Available at:

http://www.ecdc.europa.eu/en/publications/Publications/1005_TER_Risk_Assessment_Qfever.pdf

Fenollar F, Fournier PE, Carrieri MP, Habib G, Messana T, Raoult D. Risks factors and prevention of Q fever endocarditis. Clinical Infectious Diseases, 2001 Aug 1;33(3):312-6.

Forland F, De Carvalho Gomes H, Nokleby H, Escriva A, Coulombier D, Giesecke J, et al. Applicability of evidence-based practice in public health: risk assessment on Q fever under an ongoing outbreak. Euro Surveill. 2012;17(3):pii=20060. Available at: http://www.eurosurveillance.org/ViewArticle.aspx?ArticleId=20060

Haagsma JA, Maertens de Noordhout C, Polinder S, Vos T, Havelaar AH, Cassini A, Devleesschauwer B, Kretzschmar ME, Speybroeck N, Salomon JA. Assessing disability weights based on the responses of 30,660 people from four European countries. Population Health Metrics 2015; 13: 10

Karakousis PC, Trucksis M, Dumler SJ: Chronic Q fever in United States. J Clin Microbiol 2006;44: 2283–2287.

Marrie TJ, Raoult D, 2010. Coxiella burnetii (Q Fever). In Mandell, Douglas, and Bennett's Principles and Practice of Infectious Diseases [eds] Gerald L. Mandell, John E. Bennett, Raphael Dolin.—7th edition 2010, Chapter 189, 2511-2519.Maurin M, Raoult D. Q fever. Clinical Microbiology Reviews. 1999;12(4):518-53 (epub 1999/10/09).

Morroy G, Peters JB, van Nieuwenhof M, Bor HH, Hautvast JL, van der Hoek W, et al. The health status of Q-fever patients after long-term follow- up. BMC Infectious Disease, 2011 Apr 18;11:97.

Q Fever Fact Sheet: Symptoms, Diagnosis, and Treatment – CDC, Atlanta. 2013. Available at: http://www.cdc.gov/qfever/symptoms/

Raoult D, Marrie T, Mege J. Natural history and pathophysiology of Q fever. Lancet Infectious Diseases, 2005 Apr;5(4):219-26.

Stouthard ME, Essink-Bot ML, Bonsel GJ, Barendregt JJ, Kramers PGN, van de Water HPA, et al. Disability Weights for Diseases in the Netherlands. Rotterdam, the Netherlands: Department of Public Health, Erasmus University Rotterdam, the Netherlands; 1997.

van der Hoek W, Meekelenkamp JC, Leenders AC, Wijers N, Notermans DW, Hukkelhoven CW. Antibodies against Coxiella burnetii and pregnancy outcome during the 2007-2008 Q fever outbreaks in The Netherlands. BMC Infectious Diseases, 2011 Feb 11;11:44.

Rabies

The initial symptoms of rabies resemble those of other systemic viral infections (Anderson, 1984). Two kinds of central nervous system (CNS) presentation can be seen: the furious form in 70% of all cases and the paralytic form in the remainder (WHO, 2013).

The furious form usually lasts around 12 days on average (range 9�17.8 days) (Udow, 2014). The paralytic form has a longer survival period of 22 days on average (range 18�28 days) and generally results in death.

Case fatality proportion

Once the symptomatic disease onset is confirmed the case fatality proportion is considered to be 100%.

Model input summary

Table 1. Transition probabilities used in the outcome tree

Health outcome<br>(Health state)	Distribution of health states in health outcome	Transition probability	Source/assumption
Symptomatic infection<br>(Furious form)<br>(Paralytic form)	 70%<br>30%		WHO, 2013
Fatal cases		100%	WHO, 2013

Table 2. Disability weights and duration

Health outcome<br>(Health state)	Disability Weight (DW) (Haagsma, 2015)		Duration	
	DW	Label	In years	Source
Symptomatic infection	0.655 (0.579-0.727)	Intensive Care Unit admission		

(Furious form)	As above	As above	0.033 (0.025-0.049)	Udow, 2014
(Paralytic form)	As above	As above	0.060 (0.049-0.077)	Udow, 2014

References

Anderson LJ, Nicholson KG, Tauxe RV, Winkler WG. Human rabies in the United States, 1960 to 1979: Epidemiology, diagnosis, and prevention. Ann Intern Med 1984;100:728-735.

Haagsma JA, Maertens de Noordhout C, Polinder S, Vos T, Havelaar AH, Cassini A, Devleesschauwer B, Kretzschmar ME, Speybroeck N, Salomon JA. Assessing disability weights based on the responses of 30,660 people from four European countries. Population Health Metrics 2015; 13: 10

Udow S, Marrie R, Jackson A. Clinical Features of Dog- and Bat-Acquired Rabies in Humans. Clinical Infectious Diseases Volume 57, Issue 5 Pp. 689-696.

WHO. Rabies. Fact Sheet N�99. Available at: http://www.who.int/mediacentre/factsheets/fs099/en/.

Rubella

Acquired rubella

Acquired, or non-congenital rubella usually gives rise to a mild rash and asymptomatic infections are common. The rash usually begins on the face and then progresses from head to foot. It lasts about three days and is occasionally pruritic (CDC, 2009). Since up to 50% of infections may not present with a rash, many cases are not detected or reported (CDC, 2009; Ang, 2010).

Risk of complications

The most relevant complications associated with rubella virus infection include arthritis or arthralgia, thrombocytopenia, and encephalitis (Zhou, 2004). Additional, but rare complications include orchitis, neuritis, bacterial superinfection, a late syndrome of progressive panencephalitis and mild hepatitis (CDC, 2009).

Arthritis/arthralgia

Arthralgia or arthritis may occur in 30–70% of adult women who contract rubella, but it is rare in children and adult males. It rarely develops into chronic arthritis (CDC, 2009; Mandell, 1999; Johnson, 1958). An age-independent range of 30–70% was estimated as the proportion of acute infections with this complication in the model, for females only. In 11 patients with rubella arthritis studied by Yanez et al. (Yanez, 1966), the onset of arthritis occurred one to six days after the beginning of the exanthem and lasted three to 28 days (mean of nine days).

Thrombocytopenic purpura

Hemorrhagic manifestations occur in approximately one case in 3 000 – more frequently in children than in adults – of which thrombocytopenic purpura is the most common (CDC, 2009; White, 1985; Mandell, 1999; Heggie, 1969; Boyer, 1965). Based on this estimated rate of occurrence (1/3 000), the proportion with the complication was estimated as 0.03% in the model.

Acute throbocytopenic purpura is commonly seen in children aged 1–7 years, and is defined as thrombocytopenia that lasts less than six months. In cases where thrombocytopenia persists for more than six months, it is considered chronic. Chronic thrombocytopenia occurs in a very small number of children (Taghizadeh, 2008).

Encephalitis

Encephalitis occurs in one in 5 000-6 000 cases, more frequently in adults (especially in females) than in children (CDC 2009; Mandell, 1999). Notwithstanding this occurrence rate, an age/sex-independent range of 0.01–0.02% was estimated for the proportion of acute cases with this complication in the model. The severity is highly variable. Symptoms in survivors usually resolve within 1–3 weeks without neurological sequelae (Gülen, 2008; Wolinsky, 1994).

Case fatality proportion

The case fatality proportion for thrombocytopenic purpura is 2.6% (Portielje, 2001). For encephalitis the overall lethality rate is 0–50% (CDC, 2009). Therefore in the model, the case fatality proportion following the health state thrombocytopenic purpura was specified with a point estimate of 4%, and the case fatality proportion following the health state encephalitis was set to the range of 20–50%.

Congenital rubella

Symptomatic infection occurs in 100% of infected foetuses between weeks 1 and 11. During weeks 11–20, symptomatic infection occurs in 30% of foetuses. After week 20 no foetus develops any manifestation of Congenital Rubella Syndrome (CRS) (Feigin, 2004). However, occasional foetal damage (deafness only) has been observed after the twentieth week (Mandell, 1999). Up to 50% of affected foetuses may appear healthy at birth and develop central nervous system abnormalities later (Duszak, 2009). Among children with CRS, 13% have one congenital defect, 24% have two defects and 63% have three or more defects (Reef, 2000).

We did not consider any loss of quality of life before birth and therefore the disability weight and duration for the symptomatic infection was set to 0.

Risk of sequelae

Hearing impairment occurs in 60% of children with CRS, heart disease in 45%, microcephaly in 27% (Reef, 2000), cataracts in 16–25% (Bloom, 2005), mental retardation in 13–25% (Lanzieri, 2004; Reef, 2000), and retinopathy in 5% (Reef, 2000). Overall, 20–40% of CRS survivors aged 35 or older have insulin-dependent diabetes (Mandell, 1999; Duszak, 2009) and 5% of survivors aged 13–19 develop some form of thyroid disease. (Duszak, 2009). Panencephalitis is a rare, fatal, late complication. The incidence of other late complications is still unknown (Duszak, 2009).

The case fatality ratio for infants with confirmed CRS is 10% (Reef, 2000).

Model input summary

Table 1. Transition probabilities used in the outcome tree

Health outcome<br><br>(Health state)	Distribution of health states in health outcome	Transition probability	Source/assumption

Acquired			
Symptomatic infection<br>(Arthritis/arthralgia)<br>(Thrombocytopenic purpura)<br>(Encephalitis) (Uncomplicated)	30–70%; females only<br>0.03%<br>0.01–0.02%<br>Remaining cases		CDC 2009, Mandell 1999, Johnson 1958<br>CDC 2009, White 1985<br>CDC 2009, Mandell 1999
Fatal cases following thrombocytopenic purpura		2.6%	Portielje, 2001
Fatal cases following encephalitis		0–50%	CDC, 2009
Congenital			
Permanent disability due to hearing impairment		60%	Reef, 2000
Permanent disability due to congenital heart defects		45%	Reef, 2000
Permanent disability due to microcephaly		27%	Reef, 2000
Permanent disability due to cataract		16–25%	Bloom, 2005
Permanent disability due to mental retardation		13–25%	Lanzieri, 2004; Reef, 2000
Permanent disability due to retinopathy		5%	Reef, 2000
Permanent disability due to insulin-dependent diabetes		20–40%	Mandell, 1999; Duszak, 2009 (aged >35 years)
Permanent disability due to thyroid gland dysfunction		5%	Duszak, 2009 (aged 13–19 years)
Fatal cases		10%	Reef, 2000

Table 2. Disability weights and duration

Health outcome (Health state)	Disability Weight (DW) (Haagsma, 2015)		Duration

	DW	Label	In years	Source/assumption
Symptomatic infection<br>(Uncomplicated)<br>(Arthritis/arthralgia)<br>(Thrombocytopenic purpura)<br>(Encephalitis)	0.007 (0.005–0.01)<br>0.344 (0.3–0.391)<br>0.167 (0.134–0.201)<br>0.41 (0.358–0.47)	Infectious disease, acute episode, mild<br>Musculoskeletal problems, generalised,moderate<br>Thrombocytopenic purpura<br>Encephalopathy - moderate	0.008<br>0.008–0.077<br>0.008–0.5<br>0.019–0.058	CDC, 2009<br>CDC, 2009<br>Yanez, 1996<br>Taghizadeh, 2008 Gülen, 2008; Wolinsky, 1994/without any neurological sequelae
Congenital				
Symptomatic infection	0		0	
Permanent disability due to hearing impairment	0.008–0.103	From lowest to highest hearing loss related DWs	Remaining life expectancy	
Permanent disability due to congenital heart defects	0.052–0.173	From lowest to highest heart failure related DWs	Remaining life expectancy	
Permanent disability due to microcephaly	0.011–0.421		Remaining life expectancy	

		From lowest to highest cognitive difficulties related DWs		
Permanent disability due to cataract	0.004–0.171	From lowest to highest visual impairment related DWs	Remaining life expectancy	
Permanent disability due to mental retardation	0.011–0.421	From lowest to highest cognitive difficulties related DWs	Remaining life expectancy	
Permanent disability due to retinopathy	0.004–0.171	From lowest to highest visual impairment related DWs	Remaining life expectancy	
Latency period before diabetes	0		35	Mandell, 1999; Duszak, 2009
Latency period before thyroid dysfunction	0		13–19	Duszak, 2009
Permanent disability due to insulin-dependent diabetes	0.07 (0.057–0.088)	Generic uncomplicated disease: worry and daily medication	Remaining life expectancy	
Permanent disability due to thyroid gland dysfunction	0.07 (0.057–0.088)	Generic uncomplicated disease: worry and daily medication.	Remaining life expectancy	

References

Ang LW, Chua LT, James L, Goh KT. Epidemiological surveillance and control of rubella in Singapore, 1991-2007. Ann Acad Med Singapore. 2010 Feb;39(2):95-101.

Assaad F, Ljungars-Esteves K. Rubella-world impact. Rev Infect Dis. 1985 Mar-Apr;7 Suppl 1:S29-36.

Boyer WL, Sherman FE, Michaels RH, et al. Purpura in congenital and acquired rubella. N Engl J Med. 1965;273:1362.

Bloom S, Rguig A, Berraho A, Zniber L, Bouazzaoui N, Zaghloul Z, et al. Congenital rubella syndrome burden in Morocco: a rapid retrospective assessment. Lancet. 2005 Jan 8-14;365(9454):135-41.

Centers for Disease Control and Prevention. Epidemiology and Prevention of Vaccine-Preventable Diseases. Atkinson W, Wolfe S, Hamborsky J, McIntyre L, eds. 11th ed. Washington DC: Public Health Foundation, 2009.

Duszak RS. Congenital rubella syndrome--major review. Optometry. 2009 Jan;80(1):36-43.

Feigin RD. Textbook of pediatric infectious diseases, Volume 1. 2004. Elsevier Health Sciences.

Haagsma JA, Maertens de Noordhout C, Polinder S, Vos T, Havelaar AH, Cassini A, Devleesschauwer B, Kretzschmar ME, Speybroeck N, Salomon JA. Assessing disability weights based on the responses of 30,660 people from four European countries. Population Health Metrics 2015; 13: 10

Heggie AD, Robbins FC. Natural rubella acquired after birth. Am J Dis Child. 1969;118:12.

Gülen F, Cagliyan E, Aydinok Y, Ozen S, Yildiz B. A patient with rubella encephalitis and status epilepticus. Minerva Pediatr. 2008 Feb;60(1):141- 4.

Johnson RE, Hall AP. Rubella arthritis. N Engl J Med. 1958;258:743.

Lanzieri TM, Parise MS, Siqueira MM, Fortaleza BM, Segatto TC, Prevots DR. Incidence, clinical features and estimated costs of congenital rubella syndrome after a large rubella outbreak in Recife, Brazil, 1999-2000. Pediatr Infect Dis J. 2004 Dec;23(12):1116-22

Mandell GL, Bennet JE, Dolin R. Mandell, Douglas and Bennett's Principles and Practice of Infectious Disease - 5th edition. Churchill Livingstone, 1999.

Portielje JE, Westendorp RG, Kluin-Nelemans HC, Brand A. Morbidity and mortality in adults with idiopathic thrombocytopenic purpura. Blood 2001 97:2549–2554.

Reef SE, Plotkin S, Cordero JF, Katz M, Cooper L, Schwartz B, et al. Preparing for elimination of Congenital Rubella Syndrome (CRS): summary of a workshop on CRS elimination in the United States. Clin Infect Dis. 2000 Jul;31(1):85-95

Sherman FE, Michaels RH, Kenny FM. Acute encephalopathy (encephalitis) complicating rubella. JAMA. 1965;192:675.

Steen E, Torp KH. Encephalitis and thrombocytopenic purpura after rubella. Arch Dis Child. 1956;31:470.

Taghizadeh M. An Update on Immune-Mediated Thrombocytopenia. Labmedicine. Volume 39, Number 1, January 2008.

White CC, Koplan JP, Orenstein WA. Benefits, risks and costs of immunization for measles, mumps and rubella. Am J Public Health. 1985 July; 75(7): 739–744.

Wolinsky JS. Rubella. In: Fields BM, Knipe DM, Chanock RM, et al., eds. Virology. 3rd ed. New York: Raven Press, 1994;899-929.

Yanez JE, Thompson GR, Mikkelsen WM, et al. Rubella arthritis. Ann Intern Med 64:772-777, 1966.

Zhou F, Reef S, Massoudi M, Papania MJ, Yusuf HR, Bardenheier B, et al. An Economic Analysis of the Current Universal 2-Dose Measles-Mumps- Rubella Vaccination Program in the United States. J Infect Dis. (2004) 189 Supplement 1: S131-S145.

Salmonellosis

Acute gastroenteritis associated with Salmonella infections in humans is, in most cases, self-limiting within a few days or weeks, but for some patients the disease is fatal. Studies estimated the duration to be 5.58 days for gastroenteritis cases not requiring medical help, 10.65 days for gastroenteritis cases visiting a doctor but not hospitalised and 16.15 days for hospitalised gastroenteritis cases (Kemmeren 2006).

The proportion of mild (uncomplicated), moderate (complicated, doctor) and severe (complicated, doctor) symptomatic infections is set at 83.3%, 15% and 1.7% (Kemmeren 2006; Kwong 2012; redistributing in order to total 100%)

In many reports bacteraemia is highlighted as a possible extra-intestinal complication of salmonellosis (0.03% of laboratory-confirmed cases, Ternhag 2008), although these complicated cases are often considered within the hospitalised proportion of cases (Cressey & Lake 2007; Kemmeren 2006).

The case fatality proportion for symptomatic salmonellosis cases ranged from 0.1% (Kemmeren 2006; Helms 2003) to 0.05% in salmonellosis outbreaks in Austria (Much 2005) and 0.3 for non-typhoid infections in England and Wales (Adak, 2002). These were in line with case fatality proportions observed in cases reported to TESSy between 2009 and 2013 (personal communication).

We chose to estimate the overall case fatality proportion as being within the range 0.05–0.1% and assumed a different age-group distribution of this risk, based on the age-group distribution of deaths reported to TESSy between 2009 and 2013 (see Table 3). This table is based on all TESSy notified cases from EU Member States except Bulgaria, Latvia and Poland which report only aggregate data, and Italy because the outcome was not reported.

Risk of complications

Reactive arthritis (ReA) and Irritable Bowel Syndrome (IBS) are the most frequent sequelae of salmonellosis reported in the literature (Haagsma 2009; Raybourne 2003). The frequency of other post-infectious complications following salmonellosis is extremely low and these were disregarded in the current study.

Reactive arthritis (ReA)

Many studies reported ReA as sequelae of salmonellosis (Keat 1983; Fendler 2001; Raybourne, 2003). A review of the literature, which included mostly cases of salmonellosis occurring during outbreaks, estimated that 8% (2.3–15%) of cases are at risk of developing ReA (Raybourne, 2003), although most of these studies have estimated risk based on laboratory-confirmed cases and duration of diarrhoea is highly correlated with the development of ReA (Yu & Thomson, 1994). In order to account for the considerable uncertainty, the risk of developing ReA from all symptomatic cases is set at 1.31% (0.29–5.43%) (Kemmeren, 2006).

Little is known about the duration of ReA; the average duration is set at between 1.5 months, derived from Hannu et al. (Hannu, 2005) and 222 days (Kemmeren, 2006).

Irritable bowel syndrome (IBS)

In a recent literature review, 8.8% (7.2–10.4%) of symptomatic salmonellosis cases were considered at risk of developing IBS, irrespective of age and gender; the duration was set to five years (Haagsma, 2010). However, the causality is largely debated and the impact of concurrent factors significant. Therefore, IBS is not considered as part of the salmonellosis outcome tree in our study.

Model input summary

Table 1. Transition probabilities and distributions used in the outcome tree

Health outcome (Health state)	Distribution of health states in health outcome	Transition probability	Source/assumption
Symptomatic infection:<br>(Uncomplicated)<br>(Complicated, doctor)<br>(Complicated, hospital)	83.3%<br>15%<br>1.7%		Kemmeren, 2006; Kwong, 2012
Fatal cases following symptomatic infection		0.05–0.1%<br>Age dep. Table 3	Kemmeren, 2006; Much, 2005; TESSy 2009-2013
Reactive arthritis		1.31% (0.29-5.43%)	Kemmeren, 2006

Table 2. Disability weights and duration

Health outcome (Health state)	Disability Weight (DW) (Haagsma, 2015)		Duration
	DW	Label	In years

				Source
Symptomatic infection	0.073 (0.061–0.092)	Diarrhoea, mild	0.015	Kemmeren, 2006
(Uncomplicated)	0.149 (0.12–0.182)	Diarrhoea, moderate	0.029	
(Complicated, doctor)	0.239 (0.202–0.285)	Diarrhoea, severe	0.044	
(Complicated, hospital)				
Reactive arthritis	0.344 (0.3–0.391)	Musculoskeletal problems, generalised, moderate	0.131–0.608	Hannu, 2002

Table 3. Age-group redistribution of CFR (0.05–0.1%)

Age groups	%
0	0.69
1–4	1.72
5–9	1.38
10–14	0.34
15–19	1.03
20–24	0.00
25–29	1.72
30–34	0.34
35–39	1.03
40–44	0.69
45–49	2.07

50–54	3.45
55–59	4.14
60–64	5.17
65–69	9.31
70–74	12.41
75–79	16.55
80–84	18.62
>85	19.31
All ages	100.00

References

Adak GK, Long SM, O'Brien SJ. Trends in indigenous foodborne disease and deaths, England and Wales: 1992 to 2000. Gut. 2002 Dec;51(6):832- 41.

Cressey PJ and Lake R (2007). Risk ranking: Estimates of the burden of foodborne disease for New Zealand. Institute of Environmental Science & Research Limited; Christchurch Science Center.

Fendler CS, Laitko HH, Sorensen C, Gripenberg-Lerche A, Groh J, Uksila K et al. Frequency of triggering bacteria in patients with reactive arthritis and undifferentiated oligoarthritis and the relative importance of the tests used for diagnosis. Ann Rheum Dis 2001;60(4): 337-43.

Food Standards Agency (2000). A Report of the Study of Infectious Intestinal Disease in England. Food Standards Agency London: HMSO.

Haagsma JA, Havelaar AH, Janssen BM, Bonsel GJ. Disability Adjusted Life Years and minimal disease: application of a preference-based relevance criterion to rank enteric pathogens. Popul Health Metr 2008;6:7.

Haagsma JA, van der Zanden BP, Tariq L, van Pelt W, van Duynhoven YTHP, Havelaar AH. Disease burden and costs of selected foodborne pathogens in the Netherlands, 2006. Bilthoven: National Institute of Public Health and Environment. 2009. RIVM rapport 330331001. Available online at: www.rivm.nl/bibliotheek/rapporten/330331001.pdf

Haagsma JA, Siersema PD, De Wit NJ, Havelaar AH. Disease burden of post-infectious irritable bowel syndrome in the Netherlands. Epidemiol Infect 2010;138(11):1650-6.

Haagsma JA, Maertens de Noordhout C, Polinder S, Vos T, Havelaar AH, Cassini A, Devleesschauwer B, Kretzschmar ME, Speybroeck N, Salomon JA. Assessing disability weights based on the responses of 30,660 people from four European countries. Population Health Metrics 2015; 13: 10

Hannu TL, Mattila H, Rautelin P, Pelkonen P, Lahdenne A, Siitonen M et al. Campylobacter-triggered reactive arthritis: a population-based study. Rheumatology 2002;41(3): 312-318.

Havelaar AH, de Wit MAS, van Koningsveld R, van Kempen E (2000a). Health burden in the Netherlands due to infection with thermophilic Campylobacter spp. Epidemiol Infect 2000;125(3): 505-22.

Havelaar AH, de Wit MAS, van Koningsveld R (2000b). Health burden in the Netherlands (1990–1995) due to infection with thermophilic Campylobacter species. Bilthoven, the Netherlands: National Institute for Public Health and the Environment. Report no. 284550004.

Havelaar AH, Ivarsson S, Löfdahl M, Nauta MJ. Estimating the true incidence of campylobacteriosis and salmonellosis in the EU. 2012. [Submitted for publication].

Helms M, Vastrup P, Gerner-Smidt P, Molbak K. Short and long term mortality associated with foodborne bacterial gastrointestinal infections: registry based study. BMJ 2003; 326(7385): 357-60.

Keat A. Reiter's syndrome and reactive arthritis in perspective. N Engl J Med 1983;309(26): 1606-15.

Kemmeren JM, Mangen MJJ, van Duynhoven YTHP, Havelaar AH. Priority setting of foodborne pathogens: disease burden and costs of selected enteric pathogens. Bilthoven: National Institute of Public Health and Environment: RIVM rapport 330080001. 2006. Available online at: www.rivm.nl/bibliotheek/rapporten/330080001.pdf

Mangen MJJ, Havelaar AH, de Wit GA. Campylobacteriosis and sequelae in the Netherlands - Estimating the disease burden and the cost-of- illness. Bilthoven: National Institute for Public Health and the Environment; Report no. 250911004. 2004. Available online at: www.rivm.nl/bibliotheek/rapporten/250911004.pdf

Mangen MJJ, Havelaar AH, Bernsen RAJAM, van Koningsveld R, de Wit GA. The costs of human Campylobacter infections and sequelae in the Netherlands: A DALY and cost-of-illness approach. Acta Agriculturae Scandinavica Section C Food Economics 2005;(1): 35-51.

Melse JM and Kramers PGN (1998). Berekening van de ziektelast in Nederland. Achtergronddocument bij VTV-1997; deel III, hoofdstuk 7. Bilthoven; Report no. 431501028.

Much P, Pichler J, Allerberg F. Food borne infectious outbreaks, Austria 2005." Wien Klin Wochenschr 2007;119 (5-6):150-7.

Raybourne RB, Williams KM, Roberts T and Arthritis Working Group. Food Poisoning: Economic Implications. In: Caballero BL, Trugo P, Finglas (Editors). Encylopaedia of Food Sciences and Nutrition. 10 volume set edition. London: 2003; 2672-82.

Robert Koch Institut. Infektionsepidemiologisches Jahrbuch (various years). Available online at:

www.rki.de/cln_178/nn_205772/DE/Content/Infekt/Jahrbuch/jahrbuch__node.html?__nnn=true

Ternhag A, Törner A, Svensson A, Ekdahl K, Giesecke J. Short- and long-term effects of bacterial gastrointestinal infections. Emerg Infect Dis 2008;14 (1):143-8.

WHO. Global Burden of Disease 2004 Update: Disability weights for diseases and conditions. Geneva: 2004, WHO. Available online at: http://www.who.int/healthinfo/global_burden_disease/GBD2004_DisabilityWeights.pdf

Yu DTY and Thomson GTD. Clinical, epidemiological and pathogenic aspects of reactive arthritis. Food-Microbiology 1994;11(2): 97-108.

Shigellosis

Acute gastroenteritis associated with Shigella spp. infections in humans is, in most cases, self-limiting within days to weeks, but for a few patients the disease may be severe and fatal.

We assume that more complicated cases visit their doctor or are hospitalised and will subsequently be laboratory-tested and reported as confirmed. The proportion of reported cases over the total symptomatic cases is 5.45% (2.18–40%) (Haagsma, 2010).

We assumed a similar duration of symptoms as for salmonellosis: 5.58 days for uncomplicated cases and 10.65–16.15 for complicated ones (Kemmeren, 2006).

On average, patients aged 65 years and over are hospitalised for a greater number of days and are more likely to die of shigellosis than other patients (van Pelt, 2010; Barton Behravesh, 2011). We assumed that only complicated cases lead to fatalities and set the case fatality proportion for complicated cases as 0.06–0.97% (Van Pelt, 2010; Barton Behravesh, 2011). Assuming a different age-group distribution of this risk, we distributed the case fatality proportion based on the age-group distribution of deaths reported to TESSy between 2009 and 2013 (see Table 3). This table is based on all TESSy-notified cases from EU Member States except Bulgaria, Lithuania and Poland, because they report only aggregate data, and Liechtenstein, Luxembourg and Italy which do not report on the death outcome.

Risk of complications

Reactive arthritis (ReA), Post-Infectious Irritable Bowel Syndrome (PI-IBS), Haemolytic Uraemic Syndrome (HUS) and End-stage Renal Disease (ESRD) are possible sequelae of shigellosis.

Asymptomatic cases, which themselves do not have a disease burden for acute illness, might also develop sequelae. However neither the number of asymptomatic cases in the population, nor the percentage of asymptomatic cases that develop sequelae is known and these are therefore not included in the model.

Reactive arthritis (ReA)

The risk of developing ReA has been found to be 6.6% of all laboratory-confirmed cases of shigellosis (Hannu, 2005), 1.2% (Rees, 2004) and 9.8% (Schiellerup, 2008). However, severity of the acute infection and duration of diarrhoea are associated with a higher risk of developing ReA (Townes, 2008; Hannu, 2005; Rees, 2004; Schiellerup, 2008); moreover, these figures relate to laboratory-confirmed cases only. Therefore, we assume that only ‘complicated’ cases have a risk of 6.6% (1.2-9.8%) of developing ReA.

Little is known about the duration of ReA; the average duration is set between 1.5 months (Hannu, 2005) and 222 days (Kemmeren, 2006).

Irritable bowel syndrome (IBS)

In a recent literature review, 8.8% (7.2–10.4%) of symptomatic infections involving foodborne pathogens (salmonellosis, campylobacteriosis and shigellosis) were associated with a risk of developing IBS, irrespective of age and gender. The duration of IBS was set to five years (Haagsma, 2010). However, the causality is largely debated and the impact of concurrent factors significant. Therefore, IBS is not considered as part of the shigellosis outcome tree in our study.

Haemolytic uraemic syndrome (HUS)

>HUS is characterised by haemolytic anaemia (severe anaemia due to increased destruction of red blood cells), thrombocytopenia (reduced platelet count) and impaired kidney function (acute renal failure). Haemolytic anaemia and thrombocytopenia often occur after bloody diarrhoea. Acute renal failure may then follow.

Several studies have associated HUS with shigellosis infections, in particular Shigella dysenteriae type 1, a species which occurs mainly in tropical countries and accounts for approximately 30% of S. dysenteriae isolates in those countries (Mark Taylor, 2008; Chopra, 1997; Bennis, 2006; Kotloff, 1999; Ekdahl, 2005).

In Europe, based on data reported to TESSy, S. dysenteriae accounts for less than 3% of laboratory-confirmed shigellosis cases, whereas S. sonnei is the most common Shigella species (ECDC, 2013 a & b). This means that around 0.9% of the shigellosis cases occurring in Europe, caused by Shigella dysenteriae type 1, are at risk of developing HUS; however, the risk varies according to EU Member State.

The incidence of S. dysenteriae-induced HUS is unknown and it is affected by antibiotic treatment (Bennish, 2006). HUS caused by S. dysenteriae type 1 is often perceived as more severe than HUS caused by enterohaemorrhagic E. coli (EHEC), however this is probably due to the fact that such infections mainly occur in countries with limited access to high-quality healthcare. Though the age range of Shigella-induced- HUS is wider and the 'median time from the onset of diarrhoea to the presentation of HUS' is longer, HUS caused by Shigella and EHEC is very similar (Mark Taylor, 2008). Therefore, we assume that the risk of developing HUS after symptomatic infection with Shigella dysenteriae type 1 is the same as the risk for symptomatic infections with Shiga-toxin producing E. coli O157 (STEC), around 0.94–1.25% (Cressey & Lake, 2007).

Given that 0.9% of shigellosis cases occurring in Europe are caused by Shigella dysenteriae type 1, the overall risk of developing HUS after symptomatic shigellosis is set to 0.008–0.011%.

HUS occurs mainly in children aged one to five years, and less frequently in children over five years. In one study (Havelaar, 2003) 72% of all HUS cases were under 15 years of age, and 28% were older. The distribution of HUS patients admitted to the Paediatric Nephrology Department of University Hospital Nijmegen from 1974–1993 was used for cases under 15 years (Havelaar, 2003). For the current study we distributed the age risk of developing HUS (0.008-0.011%) according to TESSy-notified cases of HUS by age due to VTEC infection from 2009 to 2013 (see Table 4). Cases were from all EU Member States except Bulgaria and Lithuania, because they report only aggregate data, and Italy because it has sentinel surveillance for STEC/VTEC which does not provide sufficient coverage.

Duration of HUS is reported to be seven days (range 3–31 days) and 41% (19/46) of patients were admitted to hospital (McPherson, 2011). Hospitalisation is reported to last 2–4 weeks for HUS patients (Havelaar, 2003).

The case fatality proportion is assumed to be 3.7% (Cressey & Lake, 2007; Oxford Medical Dictionary, 2003; Havelaar, 2003). This low case- fatality might be valid for cases up to 65 years and be as high as 56% for those aged ≥65 years as data from an outbreak in Scotland suggests (Dundas, 1999). For the current study we use age-specific fatality proportions as reported by Havelaar et al. (Havelaar, 2003; see Table 5).

End-stage renal disease (ESRD)

ESRD is one of the most serious outcomes associated with HUS and is the most advanced stage of kidney failure (Oxford Medical Dictionary, 2003). HUS cases may develop ESRD briefly after HUS or after a long latency period. In the current study we assume that 13.4% develop ESRD, of which 2.9% briefly after HUS and 10.5% after a latency period of 20 years (Havelaar, 2004; Cressey & Lake, 2007). We also assume that all cases experiencing ESRD are in dialysis treatment until transplantation occurs.

The case-fatality proportion is based on the assumption that in the first year after starting dialysis mortality ratios are relatively high and differ between age-groups (see Table 6) and that only few fatalities occur after renal transplantation (Havelaar, 2003). Duration of dialysis (time to transplantation) is age-dependent and is applied to the duration of ESRD, see Table 7 (Havelaar, 2003).

Model input summary

Table 1. Transition probabilities used in the outcome tree

Health outcome<br>(Health state)	Distribution of health states in health outcome	Transition probability	Source/assumption
Symptomatic infection<br>(Uncomplicated)<br>(Complicated)	Rem. cases<br>5.45% (2.18–40%)		Haagsma, 2010
Fatal cases following complicated symptomatic infection		0.06–0.97<br>Age dep.Table 3	Van Pelt, 2010; Barton Behravesh, 2011; TESSy 2009–2013
ReA		6.6% (1.2–9.8%)	Hannu, 2005; Rees, 2004; Townes, 2008; Schiellerup, 2008
HUS		0.008–0.011%<br>Age dep.<br>Table 4	Mark Taylor, 2008; Chopra, 1997; Bennis, 2006; Kotloff, 1999; Ekdahl, 2005; ECDC, 2013 a & b; Cressey & Lake, 2007
Latency period before ESRD		10.5%	Havelaar, 2004; Cressey & Lake, 2007
ESRD after HUS		2.9%	Havelaar, 2004; Cressey and Lake, 2007

ESRD after latency period		100%	
Fatal cases following HUS		< 65 years: 3.7%<br>>=65 years: 56%<br>Table 5	Haavelar, 2004; Dundas, 1999
Fatal cases following ESRD		Age dep. & different for dialysis and transplantation<br>See Table 6.	Havelaar, 2003<br>see Table 6
Transplanted		Remaining %	

Table 2. Disability weights and duration

Health outcome (Health state)	Disability Weight (DW) (Haagsma, 2015)		Duration	
	DW	Label	In years	Source/assumption
Symptomatic infection<br>(Uncomplicated)<br>(Complicated)	0.073–0.149<br>0.239 (0.202–0.285)	Diarrhoea, from mild to moderate<br>Diarrhoea, severe	0.015<br>0.029–0.044	Kemmeren, 2006
ReA	0.344 (0.3–0.391)	Musculoskeletal problems, generalised, moderate	0.131-0.608	Estimated from Hannu, 2005; Kemmeren, 2006
HUS	0.108 (0.09–0.132)	Chronic kidney disease (stage IV)	0.019 (0.008-0.085)	McPherson, 2011
ESRD	0.487 (0.432–0.544)	End-stage renal disease, on dialysis	See Table 7	Assuming that all ESRD are in dialysis

Transplanted	0.070 (0.057–0.088)	Generic uncomplicated disease: worry and daily medication	Remaining life expectancy	Assuming no risk of re-transplantation

Table 3. Age group distribution of the case fatality proportion (0.06–0.97%)

Age groups	%
0	0.00
1–4	10.00
5–9	10.00
10–14	0.00
15–19	0.00
20–24	0.00
25–29	0.00
30–34	10.00
35–39	0.00
40–44	10.00
45–49	20.00
50–54	0.00
55–59	0.00
60–64	10.00
65–69	0.00
70–74	0.00
75–79	10.00
80–84	10.00
>85	10.00
All ages	100.00

Table 4. Age-group redistribution of risk of developing HUS (0.008–0.011%) following infection (TESSy 2009– 2013)

Age groups	%
0	5.67
1–4	33.74
5–9	13.09
10–14	6.62

15–19	2.88
20–24	2.27
25–29	3.83
30–34	3.54

35–39	2.88
40–44	3.40
45–49	3.45
50–54	2.36
55–59	2.88
60–64	3.02
65–69	2.27
70–74	3.36
75–79	1.89
80–84	1.65
85+	0.99
All ages	100

Table 5. HUS case-fatality proportion per age group

Age groups	CFR
0–65	3.7%
>65	56%

Table 6. Case-fatality proportions in the first year after starting dialysis and after renal transplantation

Age class	Case-fatality ratio dialysis	Case-fatality ratio renal transplantation
0–14	4.1% (0.9–11.1%)	7% (2.2–16%)
15–44	8.7% (5.8–12.4%)	7% (2.2–16%)
45–64	37% (31–44%)	7% (2.2–16%)
65–74	65% (58–72%)	7% (2.2–16%)
75+	79% (70–87%)	7% (2.2–16%)

Table 7. Age-specific duration of dialysis

Age class	Duration of dialysis
0–14	1.7 (0.2–5.3)
15–44	2.5 (0.2–9.6)
45–64	6.7 (0.5–30)
>65	5 to remaining life expectancy

References

Barton Behravesh C, Jones TF, Vugia DJ, Long C, Marcus R, Smith K et al. Deaths associated with bacterial pathogens transmitted commonly through food: foodborne diseases active surveillance network (FoodNet), 1996-2005. J Infect Dis, 2011. 204(2):263-7.

Bennish ML, Khan WA, Begum M, Bridges EA, Ahmed S, Saha D, et al. Low risk of hemolytic uremic syndrome after early effective antimicrobial therapy for Shigella dysenteriae type 1 infection in Bangladesh. Clin Infect Dis, 2006. 42(3):356-62.

Chopra, M., D. Wilkinson, and S. Stirling, Epidemic shigella dysentery in children in northern KwaZulu-Natal. S Afr Med J, 1997. 87(1):48-51.

Cressey PJ, Lake R. Risk ranking: Estimates of the burden of foodborne disease for New Zealand, 2007, Institute of Environmental Science & Research Limited; Christchurch Science Center: Christchurch.

Dundas S, et al. Effectiveness of therapeutic plasma exchange in the 1996 Lanarkshire Escherichia coli O157:H7 outbreak. Lancet, 1999. 354(9187):1327-30.

Ekdahl, K. and Y. Andersson, The epidemiology of travel-associated shigellosis--regional risks, seasonality and serogroups. Journal of Infection, 2005. 51(3):222-229.

European Centre for Disease Prevention and Control. Annual Epidemiological Report 2012. Reporting on 2010 surveillance data and 2011 epidemic intelligence data. Stockholm: ECDC; 2013.

European Centre for Disease Prevention and Control. Annual Epidemiological Report 2013. Reporting on 2011 surveillance data and 2012 epidemic intelligence data. Stockholm: ECDC; 2013.

Haagsma JA, Siersema PD, De Wit NJ, Havelaar AH. Disease burden of post-infectious irritable bowel syndrome in the Netherlands. Epidemiol Infect, 2010. 138(11):1650-6.

Haagsma JA, Maertens de Noordhout C, Polinder S, Vos T, Havelaar AH, Cassini A, Devleesschauwer B, Kretzschmar ME, Speybroeck N, Salomon JA. Assessing disability weights based on the responses of 30,660 people from four European countries. Population Health Metrics 2015; 13: 10

Hannu T, Mattila L, Siitonen A, Leirisalo-Repo M. Reactive arthritis attributable to Shigella infection: a clinical and epidemiological nationwide study. Annals of the rheumatic diseases, 2005. 64(4):594.

Havelaar AH, Van Duynhoven YT, Nauta MJ, Bouwknegt M, Heuvelink AE, De Wit GA, et al. Disease burden in the Netherlands due to infections with Shiga toxin-producing Escherichia coli O157. Epidemiol Infect, 2004. 132(3):467-84.

Havelaar AH, Mangen MJJ, van Duynhoven YTHP, Havelaar, AH. Disease burden in the Netherlands due to infections with Shiga toxin-producing Escherichia coli O157., in RIVM report 284550008/20032003, National Institute of Public Health and the Environment: Bilthoven, 2003.

Kemmeren J, Mangen MJJ, van Duynhoven YTHP, Havelaar AH. Priority setting of foodborne pathogens: disease burden and costs of selected enteric pathogens. RIVM report. National Institute of Public Health and the Environment: Bilthoven, 2006.

2006. Available at: http://www.rivm.nl/bibliotheek/rapporten/330080001.pdf.

Mark Taylor C. Enterohaemorrhagic Escherichia coli and Shigella dysenteriae type 1-induced haemolytic uraemic syndrome. Pediatric Nephrology, 2008. 23(9):1425-1431.

McPherson M, et al. Economic costs of Shiga toxin-producing Escherichia coli infection in Australia. Foodborne Pathogen Diseases, 2011. 8(1):55-62.

Rees JR, et al., Persistent diarrhea, arthritis, and other complications of enteric infections: a pilot survey based on California FoodNet surveillance, 1998-1999. Clin Infect Dis, 2004. 38 Suppl 3: S311-7.

Schiellerup P, Krogfelt KA, Locht H. A comparison of self-reported joint symptoms following infection with different enteric pathogens: effect of HLA-B27. J Rheumatol, 2008. 35(3): p. 480-7.

Tariq L, Haagsma J, Havelaar A. Cost of Illness and Disease Burden in The Netherlands Due to Infections with Shiga ToxinProducing Escherichia coli O157. Journal of Food Protection&# 174;, 2011. 74(4):545-552.

Townes JM, et al. Reactive arthritis following culture-confirmed infections with bacterial enteric pathogens in Minnesota and Oregon: a population-based study. Ann Rheum Dis, 2008. 67(12): 1689-96.

Van Pelt W, et al. Trends in Gastro-enteritis in Nederland; notitie met betrekking tot 2009 en 2010, in RIVM report project V/210221 December 2010, RIVM: Bilthoven.

STEC/VTEC

The current disease model relies strongly on publications focused around STEC/VTEC O157 infections. Shiga toxin-producing Escherichia coli O157 (STEC/VTEC O157) infection may be asymptomatic, or may result in acute gastroenteritis (GE), and potentially in haemorrhagic colitis: 44.5% of cases had bloody diarrhoea (Michel, 2000). Duration is assumed to be longer than for non-bloody diarrhoea (Havelaar, 2004): median duration of five days and three days for bloody and non-bloody diarrhoea respectively (Cressey & Lake, 2007), which are proposed in the model as a uniform distribution.

There is little information on STEC/VTEC-associated mortality. Study findings range from 0.083% of the total estimated/VTEC O157:H7 (Mead, 1999), 0.03% (Buzby & Roberts, 2009), 0.04% (Walkerton outbreak, one fatal case in 2 321 patients, Bruce-Grey-Owen Sound Health Unit, 2000) and 0.045 (Havelaar, 2004). We therefore assume a uniformly distributed case-fatality proportion of between 0.03% and 0.045% for this study.

Fatal cases occur mainly in elderly people (Bauch, 2007); therefore, we assumed that the case fatality proportion of 0.03–0.045% is distributed across age-groups in accordance with the observed age-group distribution of TESSy-reported deaths between 2009 and 2013 (see Table 3). This table is based on all TESSy-notified cases from EU Member States except Bulgaria and Lithuania, because they report only aggregate data, and Italy because it has sentinel surveillance for STEC/VTEC for which we do not have the coverage.

Risk of complications

STEC/VTEC infection has been associated with post-diarrhoeal haemolytic uremic syndrome (HUS), which may result in death, end-stage renal disease (ESRD) or other sequelae. HUS and ESRD are the most frequently occurring sequelae of STEC and will be considered in the outcome tree. Irritable Bowel Syndrome (IBS) is another frequently occurring sequelae of bacteria-triggered gastroenteritis (Haagsma, 2010; Marshall, 2010; Thabane, 2009) and was considered for inclusion in the outcome tree (see below). The frequency of other post-infectious complications following STEC is low and they were therefore disregarded (Havelaar, 2004; Frenzen, 2005; Cressey & Lake, 2007; Buzby, 2009; McPherson, 2011; Tariq, 2011).

Haemolytic uraemic syndrome (HUS)

Haemolytic Uraemic Syndrome (HUS) is ‘a condition in which sudden rapid destruction of red blood cells causes acute renal failure’ (Oxford Medical Dictionary, 2003). HUS may occur following a respiratory or gastrointestinal infection, especially by pathogenic Escherichia coli or

Shigella spp.

The risk of developing HUS after STEC/VTEC infection has been found to be 3–7% (McPherson, 2011), 1% (Havelaar, 2004), 0.94–1.25% (Cressey & Lake, 2007) and 1.6% of laboratory-confirmed EHEC infections although authors mention under-estimation due to misclassification (13/820; Ternhag, 2008). In the current study we assume that the probability of developing HUS after a VTEC/STEC symptomatic infection is 0.94–1.25%.

HUS occurs mainly in children between the ages of one and five years, and less frequently in children over five years. In one study, 72% of all HUS cases were under 15 years of age and 28% were older (Havelaar, 2003). Member States report HUS outcomes relating to STEC/VTEC infections and we therefore redistributed the age-group risk of developing HUS (0.94–1.24%) based on the age-group of HUS cases reported to TESSy between 2009 and 2013 (all Member States except Bulgaria, Italy and Lithuania) (see Table 4).

Duration is reported to be seven days (range 3–31 days) and 41% (19/46) of patients were admitted to hospital (McPherson, 2011); hospitalisation is reported to last two to four weeks for HUS patients (Havelaar, 2003).

The case fatality proportion was found to be 3.7% (Cressey & Lake, 2007; Oxford Medical Dictionary, 2003; Havelaar, 2003). This low case- fatality may be valid for cases up to 65 years and then as high as 56% for cases ≥65 years, as indicated by data from an outbreak in Scotland (Dundas, 1999). Other studies assume age-specific fatality rates, as reported by Havelaar et al. (Havelaar, 2003). We estimated the age-group case fatality proportion from HUS based on STEC/VTEC infections notified to TESSy between 2009 and 2013 from all Member States, except Bulgaria, Italy and Lithuania (see Table 5).

End-stage renal disease (ESRD)

ESRD is one of the most serious outcomes associated with HUS and is the most advanced stage of kidney failure (Oxford Medical Dictionary, 2003). HUS cases may develop ESRD briefly after HUS or after a long latency period. In the current study we assume that 13.4% develop ESRD, 2.9% of whom develop it briefly after HUS and 10.5% after a latency period of 20 years (Havelaar, 2004; Cressey & Lake, 2007). We also assume that all cases experiencing ESRD undergo dialysis treatment until transplantation occurs.

The case-fatality proportion is based on the assumption that in the first year after starting dialysis mortality is relatively high and differs among age-groups (see Table 6) and that only few fatalities occur after renal transplantation (Havelaar, 2003). Duration of dialysis (time to transplantation) is age-dependent and is applied to the duration of ESRD – see Table 7 (Havelaar, 2003).

Irritable bowel syndrome (IBS)

In a recent literature review, 8.8% (7.2-10.4%) of symptomatic infections with foodborne pathogens were considered at risk of developing IBS, irrespective of age and gender; the duration was set to 5 years (Haagsma, 2010). However, the causality is largely debated and the impact of concurrent factors significant. Therefore, IBS is not considered as part of the STEC/VTEC outcome tree in our study.

Model input summary

Table 1. Transition probabilities and distributions used in the outcome tree

Health outcome (Health state)	Distribution of health states in health outcome	Transition probability	Source/assumption
Fatal cases following symptomatic infection		0.03-0.045%<br>Age-dependent (Table 3)	Buzby & Roberts, 2009; TESSy 2009-2013
Haemolytic uraemic syndrome (HUS)		0.94-1.25%<br>Age-dependent (Table 4)	Havelaar, 2004; Cressey and Lake, 2007; TESSy 2009-2013
Latency period before ESRD		10.5%	Havelaar, 2004; Cressey and Lake, 2007
ESRD after HUS		2.9%	Havelaar, 2004; Cressey and Lake, 2007
ESRD after latency period		100%	
Fatal cases following HUS		Age-dependent (Table 5)	TESSy 2009-2013
Fatal cases following ESRD		Age-dependent, different for dialysis and transplantation (Table 6)	Havelaar, 2003 see Table 6
Transplanted		Remaining %	

Table 2. Disability weights and duration

Health outcome (Health state)	Disability Weight (DW) (Haagsma, 2015)		Duration	
	DW	Label	In years	Source
Symptomatic infection (Gastroenteritis)	0.149 (0.12-0.182)	Diarrhoea, moderate	0.008-0.014	Havelaar, 2004; Cressey & Lake, 2007
HUS	0.108 (0.09–0.132)	Chronic kidney disease (stage IV)	0.019 (0.008–0.085)	McPherson, 2011
ESRD	0.487 (0.432–0.544)	End-stage renal disease, on dialysis	Age dependent(See Table 7)	Assuming that all ESRD are in dialysis
Transplanted	0.070 (0.057–0.088)	Generic uncomplicated disease: worry and daily medication	Remaining life expectancy	

Table 3. Age-group redistribution of case fatality proportion (0.03–0.045%)

Age groups	%
0	4.30
1-4	9.68
5-9	4.30
10-14	0.00
15-19	0.00
20-24	2.15
25-29	0.00
30-34	0.00
35-39	3.23
40-44	3.23
45-49	2.15
50-54	1.08
55-59	4.30

60-64	8.60
65-69	4.30
70-74	10.75
75-79	10.75
80-84	15.05
>85	16.13
All ages	100.00

Table 4. Age-group redistribution of risk of developing haemolytic uraemic syndrome (0.94–1.25%)

Age	%
0	5.67
1-4	33.74
5-9	13.09
10-14	6.62
15-19	2.88
20-24	2.27
25-29	3.83
30-34	3.54
35-39	2.88
40-44	3.40
45-49	3.45
50-54	2.36
55-59	2.88
60-64	3.02
65-69	2.27
70-74	3.36
75-79	1.89
80-84	1.65
85+	0.99
All ages	100

Table 5. Age-group case fatality proportion from haemolytic uraemic syndrome (TESSy 2009–2013)

Age	%

0	6.06
1-4	2.63
5-9	3.25
10-14	0.00

15-19	0.00
20-24	5.13
25-29	0.00
30-34	0.00
35-39	3.64
40-44	3.28
45-49	3.17
50-54	2.13
55-59	2.00
60-64	4.44
65-69	8.33
70-74	4.62
75-79	17.86
80-84	25.93
85+	28.57
All ages	3.91

Table 6. Case-fatality proportions in the first year after starting dialysis and after renal transplantation

Age class	Case-fatality ratio dialysis	Case-fatality ratio renal transplantation
0–14	4.1% (0.9–11.1%)	7% (2.2–16%)
15–44	8.7% (5.8–12.4%)	7% (2.2–16%)
45–64	37% (31–44%)	7% (2.2–16%)
65–74	65% (58–72%)	7% (2.2–16%)
75+	79% (70–87%)	7% (2.2–16%)

Table 7 .Age specific duration of dialysis

Age class	Duration of dialysis
0-14	1.7 (0.2-5.3)
15-44	2.5 (0.2-9.6)
45-64	6.7 (0.5-30)

>65	5 years to remaining life expectancy

References

Bauch CT, Anonychuk AM, Pham BZ, Gilca V, Duval B, Krahn MD (2007). Cost-utility of universal hepatitis A vaccination in Canada. Vaccine 25(51): 8536-48.

Buzby JC, Roberts T (2009). The economics of enteric infections: human foodborne disease costs. Gastroenterology 136: 1851-1862.

Bruce-Grey-Owen Sound Health Unit (2000). The investigative report of the Walkerton outbreak of waterborne gastro-enteritis. Owen Sound, Ont. Canada: Bruce-Grey-Owen Sound Health Unit. Cressey PJ, Lake R (2007). Risk ranking: Estimates of the burden of foodborne disease for New Zealand. Institute of Environmental Science & Research Limited; Christchurch Science Center.

Dundas S, Murphy J, Soutar RL, Jones GA, Hutchinson SJ, Todd WT. Effectiveness of therapeutic plasma exchange in the 1996 Lanarkshire Escherichia coli O157:H7 outbreak. Lancet. 1999;354: 1327–30

Frenzen PD, Drake A, Angulo FJ (2005). Economic cost of illness due to Escherichia coli O157 infections in the United States. J Food Prot 68 (12): 2623-30.

Haagsma J.A., P.D. Siersema, N.J. De Wit, A.H. Havelaar (2010). Disease burden of post-infectious irritable bowel syndrome in the Netherlands. Epidemiol Infect 138(11): 1650-6.

Haagsma JA, Maertens de Noordhout C, Polinder S, Vos T, Havelaar AH, Cassini A, Devleesschauwer B, Kretzschmar ME, Speybroeck N, Salomon JA. Assessing disability weights based on the responses of 30,660 people from four European countries. Population Health Metrics 2015; 13: 10

Havelaar AH, van Duynhoven YT, Nauta MJ, Bouwknegt M, Heuvelink AE, de Wit GA, et al. (2003) Disease burden in The Netherlands due to infections with Shiga toxin-producing Escherichia coli O157. Epidemiol Infect 132(3): 467-84.

Marshall JK, Thabane M, Garg AX, Clark WF, Moayyedi P, Collins SM. Walkerton Health Study Investigators (2010). Eight year prognosis of post- infectious irritable bowel syndrome following waterborne bacterial dysentery. Gut;59: 605-611.

McPherson M, Kirk MD, Raupach J, Combs B, Butler JR. (2011) Economic costs of Shiga toxin-producing Escherichia coli infection in Australia. Foodborne Pathog Dis 8 (1): 55-62.

Mead PS, Slutsker L, Dietz V, McCaig LF, Bresee JS, Shapiro C et al. (1999). Food-related illness and death in the United States. Emerg Infect Dis 5(5): 607-625.

Michel P, Wilson JB, Martin SW, Clarke RC, McEwen SA, Gyles CL (2000). Estimation of the under-reporting rate for surveillance of Escherichia coli O157:H7 cases in Ontario, Canada. Epidemiol Infect 125: 35-45.

Oxford Medical Dictionary (2003). Oxford, Oxford University Press; 3rd edition.

Tariq L, Haagsma J, Havelaar A (2011). Cost of Illness and Disease Burden in The Netherlands Due to Infections with Shiga Toxin-Producing Escherichia coli O157. J Food Prot 74 (4): 545-52.

Ternhag A, Törner A, Svensson A, Ekdahl K, Giesecke J (2008). Short- and long-term effects of bacterial gastrointestinal infections. Emerg Infect Dis 14 (1): 143-8.

Thabane M, Simunovic M, Akhtar-Danesh N, Marshall JK (2009). Development and Validation of a Risk Score for Post-Infectious Irritable Bowel Syndrome. Am J Gastroenterol; 104:2267–2274.

Syphilis

Syphilis is a complex, systemic disease caused by the spirochaete Treponema pallidum (T. pallidum), a gram-negative bacterium. Syphilis is preventable and curable with effective and inexpensive antibiotics. The only known natural hosts are humans, and the pathogen is not able to survive outside its host due to limited metabolic capacities to synthetise its own bio-nutrients. Syphilis spirochetes, like other treponemas, cannot be cultivated in vitro. The primary mode of syphilis transmission is by sexual contact (acquired syphilis). Vertical transmission from infected mother to child is possible (congenital syphilis), either in utero (transfer across the placenta) or through contact with an active genital lesion during delivery (Singh, 1999). Untreated syphilis can adversely affect pregnancy outcomes, resulting in spontaneous abortion, stillbirth, premature delivery, or perinatal death. Prematurity and low birth weight have been observed in 10 to 40% of infants born to untreated mothers (Salojee, 2004). The rate of infection through sexual intercourse with an infected partner has been estimated at about 50% (Ficarra & Carlos, 2009).

In Europe and other high-income countries, the transmission via blood or blood products is rare because of the low incidence rates of the disease and improved blood screening and blood donor testing for syphilis (Tramont, 2005).

Only 50% of those infected with T. pallidum will develop symptoms (RKI, 2003). Primary syphilis lasts from two weeks to six months (Baughn & Musher, 2005). Secondary syphilis may last two to eight weeks (Zetola, 2007). Early latent disease is diagnosed less than one year after infection (WHO, 2003; MMWR, 2010). Late latent syphilis infection is diagnosed after more than one year (WHO, 2003; MMWR, 2010).

Health outcomes and states associated with syphilis infection in adults

The incubation period for primary syphilis is on average three weeks (10–90 days) and depends on bacterial load, the immune status of the infected person and the existence of other co-morbid conditions (e.g. HIV/AIDS) (Weir & Fisman, 2002; Krause, 2006). Acquired syphilis is divided into primary, secondary, latent and tertiary syphilis. The disease can also be divided into early and late syphilis. Early syphilis implies the primary, secondary and early latent stages. Late syphilis refers to late latent syphilis and tertiary syphilis (Hook, 1992).

Primary syphilis is characterised by an ulcer and/or chancre at the site of infection or inoculation. This primary lesion appears about three weeks after exposure as an indurated, painless ulcer and may not be clinically evident (i.e. it may be in the rectum or the cervix). Invasion of the bloodstream precedes the initial lesion. In 50% of cases, the chancre is accompanied by regional lymphadenopathy (a firm, non-tender satellite lymph node) (Genc, 2000). After three to six weeks the chancre begins to involute, but may persist in the secondary stage in 15–30% of those infected (Zetola, 2007; Krause, 2006; Parish, 2000).

After 2–12 weeks on average (sometimes 12 months) the untreated infection may progress to secondary syphilis caused by the haematogenic spread and lymphatic dissemination of T. pallidum in the body. The time at which the secondary lesions manifest depends on the bacterial load of the treponeme and the immune response of the host (Baughn, 2005). This stage is characterised by skin rash, condylomata lata (5–22% of patients), mucocutaneous lesions, alopecia (5–7% of patients), and generalised lymphadenopathy (Ficarra & Carlos, 2009). A patient with secondary syphilis may have one, several or all of the signs of the secondary stage. Since each of the signs may also be associated with other diseases, none are specific to syphilis. Neurological involvement in secondary syphilis (known as syphilitic meningitis) can occur, especially in HIV co-infected patients (Marra, 2004). The manifestations of secondary syphilis last two to eight weeks and then may resolve, even without treatment (Zetola, 2007).

After resolution of the secondary manifestations, around one-third of untreated patients will enter into a latent phase. The latent or asymptomatic stage of syphilis is defined as the period from disappearance of the secondary manifestations until therapeutic cure or development of late sequelae. An infection without any clinical symptoms lasting less than one year is referred to as early latent syphilis, whereas an infection of more than one year's duration without clinical evidence of treponemal infection is referred to as late latent syphilis (WHO, 2003). The definitions of duration may vary across countries. The early latent period corresponds to the highest risk of transmission.

Tertiary syphilis may appear after a long period of untreated syphilis (5–20 years after initial infection) and its manifestations can include gummas (late benign syphilis), cardiovascular symptoms and neurosyphilis (Hutto, 2001). In developed countries gummas and cardiovascular symptoms are rarely seen and most of the late sequelae are associated with neuro-syphilis. The timescale for development of neuro-syphilis may vary from a period of one or two years to more than 30 years after primary syphilis, and may involve 5–10% of untreated patients (Gjestland, 1955). It is characterised by the involvement of the central nervous system which leads to a number of different syndromes, included in the health outcome 'neuro-syphilis' in our model. In two thirds of patients the infection will not progress to late complications (Mindel, 2000).

Health outcomes and states associated with congenital syphilis infection

Postnatal manifestations of congenital syphilis are divided into early and late stages. Clinical manifestations occurring within the first two years after birth (<2 years) are categorised as early congenital syphilis. Clinical manifestations which occur later than two years after birth are late congenital syphilis (Parish, 2000). For the underlying model, and due to scarce data, only congenital syphilis was included, with no distinction between early or late.

Outcome tree parameters

Due to the high complexity of syphilis outcomes and for reasons of feasibility, the outcome tree for the adult population was split into symptomatic and asymptomatic infections at the first level of disaggregation. The natural course of syphilis was subdivided into the three main disease states: primary, secondary and neuro-syphilis. The focus was on neuro-syphilis because other forms of late syphilis sequelae are very rare in developed countries.

The percentage of asymptomatic cases was estimated at 50% (RKI 2003, Singh, 1999; Ficarra, 2009; Genc, 2000; Parish, 2000). Gerbase and colleagues presented treatment rates of 85% for both primary and secondary symptomatic syphilis cases in regions with established market economies (Gerbase, 2000). As a result of high cure rates (up to 100%), it was estimated that about 85% of all primary syphilis cases are treated and subsequently cured. The remaining 15% of untreated symptomatic cases have a 30–50% possibility of developing secondary syphilis, resulting in a probability of 4.5–7.5% that they will develop secondary syphilis, after having had primary syphilis (Singh, 1999; Weir & Fisman, 2002; Krause, 2006; Gerbase, 2000; Golden, 2003). In asymptomatic primary syphilis the primary chancre is not visible and will generally go unnoticed, meaning that it is less likely to be treated, hence the greater risk of progression to secondary syphilis (30–50%).

Furthermore, 85% of symptomatic secondary syphilis cases are treated and again, as a result of the high cure rates (around 100%), the remaining 15% of untreated cases have a probability of 5–12% of developing neuro-syphilis. Thus, the proportion of people developing neuro- syphilis from preceding secondary syphilis was set at 0.75–1.88% (Tramont, 2005; Zetola, 2007; Gerbase, 2000; Goldmeier & Guallar, 2003).

The probability of dying due to syphilis before reaching the late (tertiary) phase of the disease is very low and there is little evidence of a case fatality ratio associated with syphilis in general, or neurosyphilis in particular, within Europe. We assumed that neurosyphilis in Europe is successfully treated; although with a possibility of developing permanent disabilities for which it was impossible to define the impact due to lack of data. Antibiotic treatment is highly effective and is therefore not associated with a case fatality ratio.

For infants the main outcome is congenital infection with a probability of 20% (2–64%) for an infected child (Singh, 1999; Salojee, 2004; Genc, 2000; Gerbase, 2000). In total, 1% of all children with congenital infection die (Gerbase, 2000).

Model input summary

Table 1. Transition probabilities and distributions used in the outcome tree

Health outcome (Health state)	Transition probability	Source/assumption
Acquired		
Primary syphilis from infection	50%	RKI, 2003
Secondary syphilis from asymptomatic infection	30–50%	Singh, 1999; Weir & Fisman, 2002; Gerbase, 2000; Golden 2003
Secondary syphilis from symptomatic infection	4.5–7.5%	Singh, 1999; Weir & Fisman, 2002; Gerbase, 2000; Golden 2003
Neuro-syphilis	0.75–1.88%	Tramont, 2005; Zetola, 2007; Krause, 2006; Weir&Fisman, 2002; Gerbase, 2000; Golden, 2003; Goldmeier, 2003
Fatal cases due to neurosyphilis	0%	Assuming all cases are identified and treated, and no treatment failure
Congenital		
Symptomatic infection	20% (2–64%)	Singh, 1999; Saloojee, 2004; Genc & Ledger, 2000

Fatal cases due to congenital infection	1%	Genc & Ledger, 2000

Table 2. Disability weights and duration

Health outcome (Health state)	Disability Weight (DW) (Haagsma, 2015)		Duration	
	DW	Label	In years	Source
Acquired				
Primary syphilis	0.007 (0.005-0.01)	Infectious disease, acute episode, mild	0.121-0.5	Baughn & Musher, 2005
Latency period (from primary to secondary)	0		0.23 (0.038-1)	Baughn, 2005
Secondary syphilis	0.125 (0.104-0.152)	Infectious disease, acute episode, severe	0.038-0.153	Zetola, 2007
Latency period (from secondary to neurosyphilis)	0		4.77-19.77	Hutto, 2001
Neurosyphilis	0.407 (0.36-0.46)	Motor plus cognitive impairments, severe	0.027-0.038	Workowski, 2010 Assuming 10–14 days of treatment
Congenital				
Symptomatic infection	0.125 (0.104-0.152)	Infectious disease, acute episode, severe	3	Kwong, 2010

References

Baughn RE, Musher DM. Secondary syphilitic lesions. Clin Microbiol Rev 2005, 18:205-216.

Bremer, V, Marcus U, Hamouda O (2012). Syphilis on the rise again in Germany--results from surveillance data for 2011. Euro Surveill 17(29).

[SW1] Doherty L, Fenton KA, Jones J, Paine TC, Higgins SP, Williams D, et al. Syphilis: old problem, new strategy. BMJ 2002, 325:153-156.

Ficarra G, Carlos R. Syphilis: the renaissance of an old disease with oral implications. Head Neck Pathol 2009, 3:195-206.

Genc M, Ledger WJ. Syphilis in pregnancy. Sex Transm Infect 2000, 76:73-79.

Gerbase A, Stein C, Levison J, Htun Y. Global burden of sexually transmitted diseases (excluding HIV) in the year 2000. In: Global burden of sexually transmitted diseases (excluding HIV) in the year 2000. Geneva; World Health Organization; 2000.

Gjestland T. The Oslo study of untreated syphilis; an epidemiologic investigation of the natural course of the syphilitic infection based upon a re-study of the Boeck-Bruusgaard material. Acta Derm Venereol Suppl (Stockh) 1955, 35:3-368; Annex I-LVI.

Golden MR, Marra CM, Holmes KK. Update on syphilis: resurgence of an old problem. JAMA 2003, 290:1510-1514.

Goldmeier D, Guallar C. Syphilis: an update. Clin Med 2003, 3:209-211.

Haagsma JA, Maertens de Noordhout C, Polinder S, Vos T, Havelaar AH, Cassini A, Devleesschauwer B, Kretzschmar ME, Speybroeck N, Salomon JA. Assessing disability weights based on the responses of 30,660 people from four European countries. Population Health Metrics 2015; 13: 10

Hook EW, 3rd, Marra CM. Acquired syphilis in adults. N Engl J Med 1992, 326:1060-1069.

Hutto B. Syphilis in clinical psychiatry: a review. Psychosomatics 2001, 42:453-460.

Krause W. [Syphilis]. Urologe A 2006, 45:1494-1500.

Kwong JC, Crowcroft NS, Campitelli MA, Ratanasingham S, Daneman N, Deeks SL, et al. Ontario Burden of Infectious Disease Study. OAHPP/ECES Report. City: Ontario Agency for Health Protection and Promotion and the Institute for Clinical Evaluative Sciences; 2010.

Limb M. New scheme aims to eradicate congenital syphilis in three years. BMJ 2012, 344:e1616.

Marra C. Neurosyphilis. Curr Neurol Neurosci Rep 2004, 4:435-440.

Mindel A, Estcourt C. Syphilis. In: Sexually transmitted diseases: Vaccines, Prevention and Control. Edited by Stanberry LR, Bernstein DI: Academic Press, San Diego, San Francisco, New York, Boston, London, Sydney, Tokyo; 2000

Murray CJL, Lopez AD, World Health Organization, World Bank, Harvard School of Public Health. Global health statistics: a compendium of incidence, prevalence and mortality estimates for over 200 conditions. Geneva: World Health Organization; 1996.

Murray CJL, Lopez AD. The Global Burden of Disease - A comprehensive assessment of mortality and disability from diseases, injuries, and risk factors in 1990 and projected to 2020. Cambridge: Harvard School of Public Health on behalf of the World Health Organization and the World Bank; 1996.

Parish JL. Treponemal infections in the pediatric population. Clin Dermatol 2000, 18:687-700.

Robert Koch Institut. RKI-Ratgeber Infektionskrankheiten - Merkblätter für Ärzte: Syphilis (Lues). Epidemiologisches Bulletin 2003, 30:229-233.

Saloojee H, Velaphi S, Goga Y, Afadapa N, Steen R, Lincetto O. The prevention and management of congenital syphilis: an overview and recommendations. Bull World Health Organ 2004, 82:424-430.

Singh AE, Romanowski B. Syphilis: review with emphasis on clinical, epidemiologic, and some biologic features. Clin Microbiol Rev 1999, 12:187- 209.

Tramont EC. Treponema pallidum (Syphilis). In: Principles and practice of infectious diseases. Volume Volume 2, 6th Edition. Edited by Mandel GL, Bennet JE, Dolin R. Pennsylvania: Elsevier Inc. 2005: 2768-2785

Weir E, Fisman D. Syphilis: have we dropped the ball? CMAJ 2002, 167:1267-1268.

Workowski KA, Berman SM; Centers for Disease Control and Prevention. Sexually transmitted diseases treatment guidelines, 2010. MMWR Recomm Rep. 2010;59(RR-12):1-110.

World Health Organization. Dept. of Reproductive Health and Research. Guidelines for the management of sexually transmitted infections. Geneva: World Health Organization; 2003.

Zetola NM, Engelman J, Jensen TP, Klausner JD. Syphilis in the United States: an update for clinicians with an emphasis on HIV coinfection. Mayo Clin Proc 2007, 82:1091-1102.

Tetanus

Tetanus is an acute and often fatal disease induced by the tetanospasmin, an exotoxin produced by Clostridium tetani, a gram-positive anaerobic bacillus (Bleck, 2005; CDC, 2012). C. tetani is sensitive to heat and not viable under aerobic conditions (CDC, 2012). In contrast, the spores of C. tetani are resistant to heat and antiseptics and are widely present in soil and in the intestines and faeces of animals (e.g. horses, sheep and dogs). Tetanus is primarily contracted via contaminated wounds and is not contagious. Effective vaccination programmes significantly reduced the burden of tetanus. Globally around 800 000 to 1 000 000 people die of tetanus each year (Dietz, 1996). Around 90% of all deaths occur in developing countries which are largely affected by tetanus and especially neonatal and maternal tetanus. In developed countries, high-risk groups, such as unvaccinated persons and injecting drug users, are prone to infection with C. tetani (CDC, 2012). The proportion of asymptomatic/subclinical infections is unknown but it can be assumed that cases of tetanus are symptomatic in nearly 100% of those infected. The first symptoms of tetanus appear after an average incubation period of eight days (range: 3–21 days) (CDC, 2012). The duration of the symptomatic disease for generalised, localised and cephalic tetanus is two to three weeks (CDC, 2012).

Health outcomes/states associated with tetanus infection

The clinical features of acute tetanus infections can be subdivided into three health states that are observed in developed countries. A fourth type, tetanus neonatorum is a specific form of generalised tetanus that affects neonates and is mostly observed in the developing world with a high case fatality of up to 90% (Roper, 2007). As neonatal tetanus has been eliminated in Europe this health outcome is not considered in our outcome tree and model.

The distribution of the three health states is set according to the observed risk of developing the different forms of acute infection in USA (Bardenheier, 1998): 81% were generalised; 13% localised and 6% cephalic.

Localised tetanus

Localised tetanus is an uncommon health state of tetanus. Localised tetanus appears as a persistent contraction of muscles in the injured area, commonly preceding generalised tetanus, and lasts around two to three 3 weeks (CDC, 2012).

Generalised tetanus

The most common health state of tetanus infection is generalised tetanus. The probability of developing generalised tetanus after initial infection is around 80% (CDC, 2012; Bardenheier, 1998; Guilfoile, 2008). The symptoms of generalised tetanus are trismus or lockjaw in the early stages, developing into stiffness of the neck, difficulty in swallowing and rigidity of abdominal muscles. Further, unspecific symptoms such as elevated temperature, sweating, elevated blood pressure, and episodic rapid heart rate may occur. Generalised tetanus can last for 3-4 weeks and full recovery may take several months (CDC, 2012).

Cephalic tetanus

Cephalic tetanus is another uncommon health state involving the cranial nerves. The same duration has been assumed for this health state as for localised tetanus: 2–3 weeks.

Further complications and case fatality proportion

In cases of cephalic tetanus otitis media may occur (CDC, 2012). Long-term sequelae/disabilities from tetanus are not reported in the literature.

The overall mortality rate of tetanus ranges from 28/100 000 in developing countries to 0.1/100 000 in developed countries such as the USA. The case fatality proportion ranges between 5 and 55% (Guilfoile, 2008; Brook, 2004; Cook, 2001; Farrar, 2000; Kanchanapongkul, 2001; Miranda-Filho Dde 2004; Saltoglu, 2004; Sanford, 1995; Thwaites, 2004; Trujillo, 1987). Mortality from tetanus is clearly dependent on age, immune status and vaccination. People over 60 years of age or unvaccinated persons have an elevated lethality of 18 and 22%, respectively. In the model, the mortality rate following symptomatic cases was set at 11% (CDC, 2012; Bardenheier, 1998).

Model input summary

Table 1. Transition probabilities used in the outcome tree

Health outcome (Health state)	Distribution of health states in health outcome	Transition probability	Source/assumption
Symptomatic infection<br>(Localised tetanus)<br>(Generalised tetanus)<br>(Cephalic tetanus)	 13%<br>81%<br>6%		Bardenheier, 1998
Fatal cases		11%	CDC, 2012<br>Bardenheier, 1998

Table 2. Disability weights and duration

Health outcome (Health state)	Disability Weight (DW) (Haagsma, 2015)		Duration	
	DW	Label	In years	Source
Symptomatic infection				
(Generalised tetanus)	0.421 (0.377-0.477)	Motor impairment, severe	0.06-0.08	CDC, 2012
(Localised tetanus)	0.011 (0.008-0.014)	Motor impairment, mild	0.04-0.06	CDC, 2012
(Cephalic tetanus)	0.053 (0.042-0.064)	Motor impairment, moderate	0.04-0.06	CDC, 2012<br>Assumed same as for localised

References

Bardenheier B, Prevots DR, Khetsuriani N, Wharton M. Tetanus surveillance - United States, 1995-1997. MMWR CDC Surveill Summ 1998, 47:1- 13.

Bleck TP. Clostridium tetani (Tetanus). In Principles and practice of infectious diseases. Volume 2. Edited by Mandell GL, Bennett JE, Dolin R. Pennsylvania: Elsevier Inc.; 2005:2817–2822

Brook I. Tetanus in children. Pediatr Emerg Care 2004, 20:48-51.

Centers for Disease Control and Prevention: Tetanus. In Epidemiology and Prevention of Vaccine-Preventable Diseases. Edited by Atkinson W, Wolfe S, Hamborsky J. Washington DC: Public Health Foundation; 2012:291-300

Cook TM, Protheroe RT, Handel JM. Tetanus: a review of the literature. Br J Anaesth 2001, 87:477-487.

Dietz V, Milstien JB, van Loon F, Cochi S, Bennett J: Performance and potency of tetanus toxoid: implications for eliminating neonatal tetanus. Bull World Health Organ 1996, 74:619-628.

Farrar JJ, Yen LM, Cook T, Fairweather N, Binh N, Parry J, et al. Tetanus. J Neurol Neurosurg Psychiatry 2000, 69:292-301.

Guilfoile PG. Tetanus. New York: Infobase Publishing; Chelsea House; 2008.

Haagsma JA, Maertens de Noordhout C, Polinder S, Vos T, Havelaar AH, Cassini A, Devleesschauwer B, Kretzschmar ME, Speybroeck N, Salomon JA. Assessing disability weights based on the responses of 30,660 people from four European countries. Population Health Metrics 2015; 13: 10

Hsu SS, Groleau G. Tetanus in the emergency department: a current review. J Emerg Med 2001, 20:357-365.

Kanchanapongkul J. Tetanus in adults: a review of 85 cases at Chon Buri Hospital. J Med Assoc Thai 2001, 84:494-499.

Miranda-Filho Dde B, Ximenes RA, Barone AA, Vaz VL, Vieira AG, Albuquerque VM. Randomised controlled trial of tetanus treatment with anti- tetanus immunoglobulin by the intrathecal or intramuscular route. BMJ 2004, 328:615.

Murray CJL, Lopez AD. World Health Organization., World Bank., Harvard School of Public Health.: Global health statistics : a compendium of incidence, prevalence and mortality estimates for over 200 conditions. Geneva: World Health Organization; 1996.

Roper MH, Vandelaer JH, Gasse FL. Maternal and neonatal tetanus. Lancet 2007, 370:1947-1959.

Saltoglu N, Tasova Y, Midikli D, Burgut R, Dundar IH. Prognostic factors affecting deaths from adult tetanus. Clin Microbiol Infect 2004, 10:229- 233.

Sanford JP. Tetanus: forgotten but not gone. Waltham, MA, : Massachusetts Medical Society; 1995.

Thwaites CL, Yen LM, Nga NT, Parry J, Binh NT, Loan HT et al. Impact of improved vaccination programme and intensive care facilities on incidence and outcome of tetanus in southern Vietnam, 1993-2002. Trans R Soc Trop Med Hyg 2004, 98:671-677.

Trujillo MH, Castillo A, Espana J, Manzo A, Zerpa R. Impact of intensive care management on the prognosis of tetanus. Analysis of 641 cases. Chest 1987, 92:63-65.

Tick-borne encephalitis (TBE)

Most cases of tick-borne encephalitis (TBE) in Europe involve a biphasic presentation of the disease with fever during the first phase and neurological disorders during the second phase (Gubler, 2007). Severity of tick-borne encephalitis increases with age. TBE in children (<14 years) usually runs a more benign course (Mickiene, 2002; Kaiser, 1999). The proportion of asymptomatic cases is 66–80% (Gustafson, 1992). To calculate the burden of disease we assume that asymptomatic patients do not develop sequelae and are not included in the burden estimation.

The subtype considered is the Central European encephalitis subtype (Western tick-borne encephalitis virus) which is the dominant one in Europe. Another subtype does occur, the Russian spring-summer encephalitis subtype, however this occurs less in EU Member States and is not considered in the outcome tree.

The symptomatic infection (viraemic phase) begins after an average incubation period of eight days (range 4–28 days) (Kaiser, 1999). Symptoms of this first phase include fever, muscle pain, fatigue and headache (Gunther, 1997; Kaiser, 1999), normally lasting for five (2–7) days (Gubler, 2007).

Meningoencephalitic phase

After a symptom-free period, usually less than two weeks, a meningoencephalitic second phase occurs in 20–30% of symptomatic patients (Gustafson, 1990; 1992; Kiffner, 2010). The duration of the meningoencephalitic phase is set to 15 days (10–70) (Kaiser, 1999). The case fatality proportion of the meningoencephalitic phase is set to 0.75% (Mickiene, 2002).

Paralysis and residual paresis

Following the meningoencephalitic phase there is a latency period of six days (range 1–17 days), after which paralysis occurs in an estimated 11% of patients (Gunther, 1997). The duration is set to 3–10 days (Kaiser, 1999). Overall, 56% of paralytic patients are at risk of developing lifelong residual paresis (partial loss of or impaired movement) (Gunther, 1997).

Post-encephalitic TBE syndrome

A long-term post-encephalitic TBE syndrome, with symptoms including cognitive or neuropsychiatric complaints, balance disorders, headache, dysphasia, hearing defects and spinal paralysis, has been reported in 39–46% of meningoencephalitic patients (Gunther, 1997; Mickiene, 2002). The duration of post-encephalitic TBE syndrome is set to one year ('Post TBE syndrome existed after 1 year in more than one third of the patients' Gunther, 1997).

Lifelong chronic sequelae can persist in 35.7% (Haglund & Gunther, 2003) to 38.8% of post-encephalitic syndrome patients (Gunther, 1997: 'persisting symptoms at 12 months in 33/85 patients'). Males are affected twice as much as females and 12% of patients with post-encephalitic TBE syndrome were under 14 years of age (Kaiser, 1999). However, the association between gender, age and severity still needs more research and is not considered in the outcome tree.

Model input summary

Table 1. Transition probabilities used in the outcome tree

Health outcome (Health state)	Distribution of health states in health outcome	Transition probability	Source/assumption
Symptomatic infection		20–34%	Gustafson, 1992
Meningoencephalitic phase		20–30%	Gustafson, 1990, 1992; Kiffner, 2010
Paralysis		11%	Kaiser, 1999; Gunther, 1997
Residual paresis		56%	Gunther, 1997
Post-encephalitic TBE syndrome		39–46%	Gunther 1997; Mickiene, 2002
Chronic post-encephalitic TBE syndrome		35.7–38.8%	Haglund & Gunther, 2003<br>Gunther, 1997
Fatal cases following meningoencephalitic phase		0.75%	Mickiene, 2002

Table 2. Disability weights and duration

Health outcome (Health state)	Disability Weight (DW) (Haagsma, 2015)		Duration	
	DW	Label	In years	Source

Symptomatic infection	0.051 (0.039-0.06)	Infectious disease, acute episode, moderate	0.014 (0.005-0.019)	Gubler, 2007
Meningoencephalitic phase	0.447 (0.391-0.501)	Encephalopathy - severe	0.041 (0.027-0.192)	Kaiser, 1999
Paralysis	0.526 (0.469-0.586)	Spinal cord lesion at neck level (treated)	0.0137	Kaiser, 1999
Residual paresis	0.056 (0.044-0.067)	Motor plus cognitive impairments, mild	Remaining life expectancy	Remaining life expectancy
Post-encephalitic TBE syndrome	0.202 (0.167-0.242)	Motor plus cognitive impairments, moderate	1	Gunther, 1997
Chronic post-encephalitic TBE syndrome	0.056 (0.044-0.067)	Motor plus cognitive impairments, mild	Remaining life expectancy	Remaining life expectancy

References

Gubler JD, Kuno G, Markoff L. 2007. Flaviviruses. In: Knipe, D.M., Howley, P.M. (Eds.), Fields Virology 5th ed., vol. 1. Lippincott Williams & Wilkins, London, New York, Tokyo, pp. 1043–1125.

Gustafson R, Svenungsson B, Forsgren M, Gardulf A, Granström M. Two-year survey of the incidence of Lyme borreliosis and tick-borne encephalitis in a high-risk population in Sweden. European Journal of Clinical Microbiology and Infectious Diseases, 1992 Oct;11(10):894-900. PMID: 1486884

Gustafson R, Svenungsson B, Gardulf A, Stiernstedt G, Forsgren M. Prevalence of tick-borne encephalitis and Lyme borreliosis in a defined Swedish population. Scandinavian Journal of Infectious Diseases, 1990;22(3):297-306. PMID: 2371545

Günther G, Haglund M, Lindquist L, Forsgren M, Sköldenberg B. Tick-bone encephalitis in Sweden in relation to aseptic meningo-encephalitis of other etiology: a prospective study of clinical course and outcome. Journal of Neurology, 1997 Apr;244(4):230-8. PMID: 9112591

Haglund M, Günther G. Tick-borne encephalitis--pathogenesis, clinical course and long-term follow-up. Vaccine, 2003 Apr 1;21 Suppl 1:S11-8. PMID: 12628810

Haagsma JA, Maertens de Noordhout C, Polinder S, Vos T, Havelaar AH, Cassini A, Devleesschauwer B, Kretzschmar ME, Speybroeck N, Salomon JA. Assessing disability weights based on the responses of 30,660 people from four European countries. Population Health Metrics 2015; 13: 10

Kaiser R. The clinical and epidemiological profile of tick-borne encephalitis in southern Germany 1994-98: a prospective study of 656 patients. Brain 1999; 122 (Pt 11): 2067-78. PMID: 10545392

Kiffner C, Zucchini W, Schomaker P, Vor T, Hagedorn P, Niedrig M, et al. Determinants of tick-borne encephalitis in counties of southern Germany, 2001-2008. International Journal of Health Geography, 2010 Aug 13;9:42. PMID: 20707897

Mickiene A, Laiskonis A, Gunther G, et al. Tick-borne encephalitis in an area of high endemicity in Lithuania: disease severity and long-term prognosis. Clin Infect Dis 2002; 35 (6): 650-8 PMID: 12203160

Toxoplasmosis

Acquired toxoplasmosis

In Europe, most cases of acquired toxoplasmosis are asymptomatic and self-limiting (Rorman, 2006). Acquired toxoplasmosis will lead to symptomatic illness in approximately 10–20% of infected cases (Montoya, 2000). It is estimated that 4.67% (0–15.3%) of symptomatic cases will manifest more severe symptoms and approximately 2% (0–4.67%) are at risk of developing life-long sequelae relative to chorioretinitis. However, it is unclear if this risk is attributable mainly to more severe, symptomatic infections or all infections (Kemmeren, 2006). All other symptomatic cases will manifest minor symptoms, such as fever and lymphoadenopathy (Rorman, 2006; Anand, 2012).

Mortality due to acquired toxoplasmosis is extremely rare and occurs in immunocompromised patients. It has therefore been decided to exclude fatal cases from the outcome tree of acquired toxoplasmosis.

Toxoplasmosis may also play a role in the development of psychiatric disorders, such as schizophrenia and bipolar depression (Torrey, 2003; Henriquez, 2009; Brown, 2010). However, insight into causality is still insufficient and these sequelae are not included in the model.

Congenital toxoplasmosis

Vertical transmission from a recently infected pregnant woman to her foetus may lead to congenital toxoplasmosis. Infections occurring during the first and second trimester of pregnancy may result in foetal loss (1.5–1.7% of seroconverting pregnant women, Havelaar 2007) or stillbirth (although neither of these are included in the present burden estimation) and symptoms in newborn infants are generally more severe.

However, if the infection occurs in the third trimester the disease manifestation is generally subclinical. When present, symptoms vary from a triad including chorioretinitis, intracranial calcification and hydrocephalus to abnormalities of the central nervous system. These complications may lead to life-long sequelae, including subclinical congenital toxoplasmosis which could increase the risk of developing chorioretinitis later in life. Death can occur in a small proportion of infections. Other symptoms are very rare and have not been considered in this model.

Several studies have described clinical manifestations and follow-up of newborns infected with toxoplasmosis: 89% of children were asymptomatic at birth (16% of them developed chorioretinitis later in life) (Berrebi, 2010), 85% had no clinical findings at birth (Lebech, 1999) and 74.5% were asymptomatic at birth (Schmidt, 2006). Therefore, the proportion of asymptomatic infections out of the total congenital toxoplasmosis infections is 11–25%.

Asymptomatic congenital toxoplasmosis-infected infants have a 2% (1–3%) per year risk of developing chorioretinitis at a later age. The studies followed cases of asymptomatic congenital toxoplasmosis for 10–14 years (Havelaar, 2007).

Based on an extensive literature review, Havelaar et al. (Haavelar, 2007) estimated the risk of developing permanent disabilities related to congenital toxoplasmosis infections. We applied the same estimates to our model for all infections: 13% (12–15%) will develop permanent disabilities due to complications related to chorioretinitis, 11% (8–12%) to intracranial calcification, 3% (1-6%) to the central nervous system and 2% (1–3%) to hydrocephalus.

Model input summary

Table 1. Percentages used in the outcome tree

Health outcome<br><br>(Health state)	Distribution of health<br><br>states in health outcome	Transition probability	Source/assumption

Acquired toxoplasmosis			
Symptomatic infections:<br>(Uncomplicated)<br>(Complicated)	 Remaining cases<br>4.67% (0–15.3%)	10–20%	Kemmeren, 2006
Chorioretinitis following symptomatic infection		2% (0–4.67%)	Kemmeren, 2006
Congenital toxoplasmosis			
Symptomatic infections:<br>(Asymptomatic)<br>(Symptomatic)	75–89%<br>Remaining cases		Berrebi, 2010<br>Lebech, 1999<br>Schmidt, 2006
Permanent disability due to chorioretinitis after the first year following asymptomatic infection		2% (1-3%) per year (ATP) for 10–14 years	Havelaar, 2007<br>Starting one year after infection up to the age of 10–14 years<br>ATP: Annual Transition Probability

Permanent disability due to chorioretinitis within first year		13% (12–15%)	Havelaar, 2007
Permanent disability due to intracranial calcification		11% (8–12%)	Havelaar, 2007
Permanent disability due to hydrocephalus		2% (1–3%)	Havelaar, 2007
Permanent disability due to CNS abnormalities		3% (1–6%)	Havelaar, 2007
Fatal cases		0.7% (0.4–1.2%)	Havelaar, 2007

Table 2. Disability weights and duration

Health outcome (Health state)	Disability Weight (DW) (Haagsma, 2015)		Duration	
	DW	Label	In years	Source
Acquired toxoplasmosis				
Acquired toxoplasmosis (Uncomplicated)<br>(Complicated)	0.007 (0.005–0.01)<br>0.125 (0.104–0.152)	Infectious disease, acute episode, mild<br>Infectious disease, acute episode, severe	0.04	Kemmeren, 2006
Congenital toxoplasmosis				
Congenital toxoplasmosis (Asymptomatic)<br>(Symptomatic)	0<br>0.125 (0.104–0.152)	Infectious disease, acute episode, mild<br>Infectious disease, acute episode, severe	1<br>0.167	Assuming chorioretinitis starts after one year<br>Melse, 2000
Permanent disability due to chorioretinitis following asymptomatic infections	0.015 (0.011–0.019)	Conjunctivitis without corneal scar	rem life exp.	Havelaar, 2007
Permanent disability due to	0.015 (0.011–0.019)	Conjunctivitis without corneal	rem life exp.	Havelaar, 2007

chorioretinitis following symptomatic infections		scar		
Permanent disability due to intracranial calcification	0.044–0.087	Intellectual disability/mental retardation, from mild to moderate	rem life exp.	Havelaar, 2007
Permanent disability due to hydrocephalus	0.044–0.188	Intellectual disability/mental retardation, from mild to severe	rem life exp.	Havelaar, 2007
Permanent disability due to CNS abnormalities	0.056–0.407	Motor plus cognitive impairments, from mild to severe	rem life exp.	Havelaar, 2007
Permanent disability due to chorioretinitis	0.015 (0.011–0.019)	Conjunctivitis without corneal scar	rem life exp.	Kemmeren, 2006

References

Anand R, Jones CW, Ricks JH, Sofarelli TA, Hale DC. Acute primary toxoplasmosis in travelers returning from endemic countries. J Travel Med. 2012; 19(1): 57-60.

Berrebi A, Assouline C, Bessieres MH, Lathiere M, Cassaing S, Minville V, et al. Long-term outcome of children with congenital toxoplasmosis. American journal of obstetrics and gynecology. 2010; 203(6): 552 e1-6.

Brown AS, Derkits EJ. Prenatal infection and schizophrenia: a review of epidemiologic and translational studies. Am J Psychiatry. 2010; 167(3): 261-80.

Burnett AJ, Shortt SG, Isaac-Renton J, King A, Werker D, Bowie WR. Multiple cases of acquired toxoplasmosis retinitis presenting in an outbreak. Ophthalmology. 1998; 105(6): 1032-7.

Haagsma JA, Maertens de Noordhout C, Polinder S, Vos T, Havelaar AH, Cassini A, Devleesschauwer B, Kretzschmar ME, Speybroeck N, Salomon JA. Assessing disability weights based on the responses of 30,660 people from four European countries. Population Health Metrics 2015; 13: 10

Havelaar AH, Kemmeren JM, Kortbeek LM. Disease burden of congenital toxoplasmosis. Clin Infect Dis. 2007; 44(11): 1467-74.

Henriquez SA, Brett R, Alexander J, Pratt J, Roberts CW. Neuropsychiatric disease and Toxoplasma gondii infection. Neuroimmunomodulation. 2009; 16(2): 122-33.

Kemmeren JM, Mangen, MJJ, van Duynhoven YTHP, Havelaar AH. Priority setting of foodborne pathogens: disease burden and costs of selected enteric pathogens. Bilthoven: National Institute of Public Health and Environment; 2006. Report No.: RIVM rapport 330080001.

Lebech M, Andersen O, Christensen NC, Hertel J, Nielsen HE, Peitersen B, et al. Feasibility of neonatal screening for toxoplasma infection in the absence of prenatal treatment. Danish Congenital Toxoplasmosis Study Group. Lancet. 1999; 353(9167): 1834-7.

Melse JM, Essink-Bot ML, Kramers PG, Hoeymans N. A national burden of disease calculation: Dutch disability-adjusted life-years. Dutch Burden of Disease Group. Am J Public Health. 2000; 90(8): 1241-7.

Montoya J, Remingtons J. Toxoplasma gondii. In: Mandell G, Douglas, Benett J, editors. Principles and Practice of Infectious Diseases. Philadelphia: Churchill Livingstone; 2000.

Rorman E, Zamir CS, Rilkis I, Ben-David H. Congenital toxoplasmosis--prenatal aspects of Toxoplasma gondii infection. Reprod Toxicol. 2006; 21(4): 458-72.

Schmidt DR, Hogh B, Andersen O, Fuchs J, Fledelius H, Petersen E. The national neonatal screening programme for congenital toxoplasmosis in Denmark: results from the initial four years, 1999-2002. Archives of disease in childhood. 2006; 91(8): 661-5.

Torrey EF, Yolken RH. Toxoplasma gondii and schizophrenia. Emerg Infect Dis. 2003; 9(11): 1375-80.

Tuberculosis

Tuberculosis (TB) is an infectious disease caused by the Mycobacterium tuberculosis. The term �tuberculosis� is also used for other similar diseases caused by M. bovis and M. africanum (Fitzgerald, 2005; Comstock, 1998). However, for the purposes of the disease report, outcome tree and model presented here, only those infections caused by M. tuberculosis complex are considered.

Tuberculosis bacteria are transmitted via droplets � by coughing, sneezing or talking � and mostly affect the lungs of humans, although they can also result in a systemic disease, affecting virtually all organs (Fitzgerald, 2005). The course �of TB can be split into several phases. The first phase after infection, �primary TB�, is observed in a minority of patients. The majority of infected (asymptomatic) persons proceed to a �latent� stage, lasting from months to several years or even for the rest of their life. Due to endogenous or exogenous reactivation, people may develop active TB after a certain time spent in the latent stage of the disease.

According to published literature only 5�10% of all infected individuals develop symptoms of active (primary) TB (cough, fever, lethargy, and weight loss) in their lifetime (Castillo-Chavez & Feng, 1997; Gideon & Flynn, 2011, Lin & Flynn, 2010, North & Jung, 2004).

Health outcomes and health states associated with tuberculosis infection

The main health outcomes associated with TB infection are active (primary) TB, MDR (multidrug-resistant) TB and XDR (extensively drug- resistant) TB. After initial infection with M. tuberculosis, an immuno-competent person is generally able to stop the replication and spread of bacilli and thus does not develop any symptoms. Primary TB can be split in pulmonary TB (the majority of cases) and extra-pulmonary TB, affecting different sites of the human organism. Given the complexity of the disease course, all TB cases are considered in the model, with a focus on the distinction between drug-susceptible (DS TB), MDR and XDR TB and their relative case fatality proportions (CFP), irrespective of the site of infection.

Of all laboratory-confirmed TB cases notified to ECDC/WHO between 2009 and 2013, on average 4.5% were multidrug-resistant and 14.6% of these cases were extensively drug resistant (ECDC/WHO, 2015). Therefore, in our model of all symptomatic infections 4.5% are considered to be MDR TB and 0.64% are considered to be XDR TB. However, it should be noted that these proportions vary widely across countries and users are advised to tailor them according to the epidemiology of the population under study.

Transition probabilities

In a cost-effectiveness analysis performed by Tseng and colleagues the authors used various assumptions on the progression of TB. Their model estimates the risk of active TB to be about 5% within the first two years of TB infection. Spontaneous resolution without treatment was set to 25%. Cure rates of TB with treatment and cure rates of MDR TB with treatment were 62.4% and 68.6% respectively (Tseng, 2011).

Tiemersma and colleagues estimated CFP and assessed durations of untreated pulmonary TB in HIV-negative patients and stated an overall case-fatality proportion of 30.7% in the first year of follow-up. The highest proportions were observed shortly after diagnosis. The 5-year and 10-year averages for case fatalities were 58% and 73% respectively (Tiemersma, 2011). In their review they also included the study conducted by Berg, estimating sex- and age-specific 10-year mortality rates. For men aged 15�29, 30�49 and >50 years, the 10-year mortality rates were 66%, 70% and 94% respectively. For women aged 15�29, 30�49 and >50 years, 10-year mortality rates were 70%, 69% and 92% respectively (Berg, 1951). Assuming that detected TB cases are treated in Europe, the case fatality proportions cited above overestimate current TB mortality patterns. Duration of pulmonary TB and TB is difficult to estimate due to difficulties in establishing onset of disease; based on estimates from prevalence and incidence studies an average duration of three years was suggested (Tiemersma, 2011).

A cost-effectiveness analysis using Markov models estimated active TB progression rates from underlying latent TB on the basis of disease duration and age-dependent case-fatality rates. Base case rates for developing active TB from latent TB within 1�2 years, 3�5 years and 6�7 years of exposure were estimated at 0.74%, 0.31% (0�2.5%), and 0.16% respectively. Age-specific death rates for people aged 35, 50 and 70 years were 1%, 5% and 10% respectively (Pisu, 2009).

Based on an international TB network, the US Centers for Disease Control and Prevention (CDC) and the World Health Organization (WHO) estimated that in 2004, of 17 960 TB isolates, 20% were MDR and 2% XDR. In population-based trials in the US, Latvia and South Korea 4%, 19% and 15% of all MDR TB cases were XDR in 2004. The studies in the US and Latvia also provided additional information on the progression of MDR and XDR TB in 2004. In the US study 55% of MDR patients completed treatment/were cured and 25% died during treatment. With regard to XDR, 31% completed treatment/were cured and 23% died. Results from Latvia show the percentage of completed treatment/cases cured of MDR TB to be 69% and that of deaths/failures to be 17%. For XDR 61% completed treatment/were cured and 17% died/or had failed treatment (CDC, 2006).

Jaquet and colleagues estimated the impact of DOTS[*] in Haiti and therefore conducted a cost-effectiveness analysis with probability estimates and outcome features of TB taken from literature. For reactivation of latent TB they estimated a probability of 0.1% per year for infection present for more than two years. Within two years of a new TB infection they estimated a base case rate of 5% (2�15%) for developing TB. Cure rates of treated smear positive (drug-sensitive) TB were estimated at 62.4%. For MDR TB, authors assumed a cure rate of 48% (base case; range 48�73%) and the proportion of deaths to be 12% (base case; range 12�26%) (Jacquet, 2006).

Outcome tree parameters

Given the changes in TB epidemiology in Europe during recent decades, the situation has not been sufficiently stable to enable incidence of infection to be estimated from active TB case data. It was therefore decided not to consider latent TB in the model.

Duration of symptomatic TB is set to 0.2�2 years, irrespective of whether it is active, MDR or XDR TB (WHO, 2014).

The case fatality proportion for active TB cases is estimated to be 43% in cases not on TB treatment (Corbett, 2003; Tiemersma, 2011) and 3% in cases on TB treatment (Straetemans, 2011). Given that the estimated incidence of active TB (non-MDR or XDR) in EU/EEA is 10% higher than the notification rate (ECDC/WHO, 2015) and, assuming that all notified cases are being treated, the CFP of active TB (non-MDR or XDR) cases was set at 7%.

The case fatality proportion for MDR TB was set at 12.8% (2.3�23.3%) (Straetemans, 2011). Given the lack of evidence on the case fatality ratio for XDR TB, we used the treatment outcome result category �Died�, notified in the EU/EEA, as a proxy for estimating the XDR TB case fatality proportion and set the value at 27% (ECDC/WHO, 2015).

Model input summary

Table 1. Transition probabilities and distributions used in the outcome tree

Health outcome (Health state)	Distribution of health states in health outcome	Transition probability	Source/assumption
Active TB<br>(Remaining active cases)<br>(MDR, non-XDR)<br>(XDR)	94.86%<br>4.5%<br>0.64%		ECDC/WHO, 2015
Fatal cases following remaining active cases		7%	Modelled based on Corbett, 2003; Tiemersma, 2011; Straetemans, 2011
Fatal cases following MDR TB		12.8% (2.3�23.3%)	Straetemans, 2011
Fatal cases following XDR TB		27%	ECDC/WHO, 2015

Table 2. Disability weights and duration

Health outcome (Health state)	Disability Weight (DW) (Haagsma, 2015)		Duration	
	DW	Label	In years	Source
Active TB<br>(Remaining active cases)<br>(MDR, non-XDR)<br>(XDR)	0.308 (0.264�0.353)<br>0.308 (0.264�0.353)<br>0.308 (0.264�0.353)	Tuberculosis, not HIV infected<br>Tuberculosis, not HIV infected<br>Tuberculosis, not HIV infected	2<br>2<br>2	WHO, 2013<br>WHO, 2013<br>WHO, 2013

References

Berg G. Statistik �ber die Tuberkulosemortalit�t in Kriegszeiten. Beitr�ge zur Klinik der Tuberkulose 1951, 106:1-9.

Castillo-Chavez C, Feng Z. To treat or not to treat: the case of tuberculosis. J Math Biol 1997, 35:629-656.

Centers for Disease Control and Prevention: Emergence of Mycobacterium tuberculosis with extensive resistance to second-line drugs - worldwide, 2000�2004. MMWR Morb Mortal Wkly Rep 2006, 55:301-305.

Comstock G, O'Brian R. Tuberculosis. In Bacterial Infections in Humans - Epidemiology and Control. Volume 3rd edition. Edited by Evans A, Brachman P. New York: Plenum Publishing Corporation; 1998: 777-804

Corbett EL, Watt CJ, Walker N, Maher D, Williams BG, et al. (2003) The growing burden of tuberculosis: global trends and interactions with the HIV epidemic. Arch Intern Med 163: 1009-1021.

Dye C, Scheele S, Dolin P, Pathania V, Raviglione M (for the WHO Global Surveillance Monitoring Project). Global burden of tuberculosis: Estimated incidence, prevalence, and mortality by country. JAMA 1999, 282:677-686.

European Centre for Disease Prevention and Control/WHO Regional Office for Europe. Tuberculosis surveillance and monitoring in Europe 2015.

European Centre for Disease Prevention and Control, WHO Regional Office for Europe: Tuberculosis surveillance and monitoring in Europe. In: Tuberculosis surveillance and monitoring in Europe. Stockholm: European Centre for Disease Prevention and Control; 2012.

Fitzgerald D, Haas D. Mycobacterium tuberculosis. In: Principles and Practice of Infectious Diseases. Sixth edition. Edited by Mandell G, Bennett J, Dolin R. Philadelphia: Elsevier Churchill Livingstone; 2005: 2852-2886

Gideon HP, Flynn JL. Latent tuberculosis: what the host "sees"? Immunol Res 2011, 50:202-212.

Global Tuberculosis Report 2013. 1.Tuberculosis � epidemiology. 2.Tuberculosis, Pulmonary � prevention and control. 3.Tuberculosis � economics. 4.Tuberculosis, Multidrug-Resistant. 5. Annual reports. World Health Organization. ISBN 978 92 4 156465 6. Available at: http://apps.who.int/iris/bitstream/10665/91355/1/9789241564656_eng.pdf

Global Tuberculosis Report 2014. 1.Tuberculosis � epidemiology. 2.Tuberculosis, Pulmonary � prevention and control. 3.Tuberculosis � economics. 4.Tuberculosis, Multidrug-Resistant. 5. Annual reports. World Health Organization. ISBN 978 92 4 156480 9. Available at: http://www.who.int/tb/publications/global_report/gtbr14_main_text.pdf?ua=1

Haagsma JA, Maertens de Noordhout C, Polinder S, Vos T, Havelaar AH, Cassini A, Devleesschauwer B, Kretzschmar ME, Speybroeck N, Salomon JA. Assessing disability weights based on the responses of 30,660 people from four European countries. Population Health Metrics 2015; 13: 10

Jacquet V, Morose W, Schwartzman K, Oxlade O, Barr G, Grimard F, et al. Impact of DOTS expansion on tuberculosis related outcomes and costs in Haiti. BMC Public Health 2006, 6:209.

Jassal M, Bishai WR. Extensively drug-resistant tuberculosis. Lancet Infect Dis 2009, 9:19-30.

Lin PL, Flynn JL. Understanding Latent Tuberculosis: A Moving Target. The Journal of Immunology 2010, 185:15-22.

Murray CJL, Lopez AD, World Health Organization, World Bank, Harvard School of Public Health. The global burden of disease: a comprehensive assessment of mortality and disability from diseases, injuries, and risk factors in 1990 and projected to 2020. Geneva: World Health Organization; 1996.

North RJ, Jung YJ. Immunity to tuberculosis. Annu Rev Immunol 2004, 22:599-623.

Pisu M, Gerald J, Shamiyeh JE, Bailey WC, Gerald LB. Targeted tuberculosis contact investigation saves money without sacrificing health. J Public Health Manag Pract 2009, 15:319-327.

Raviglione M. Tuberculosis - the essentials. New York: Informa Health Care; 2010.

Straetemans M, Glaziou P, Bierrenbach AL, Sismanidis C, van der Werf MJ. Assessing tuberculosis case fatality ratio: a meta-analysis. PLoS One 2011, 6:e20755.

Tiemersma EW, van der Werf MJ, Borgdorff MW, Williams BG, Nagelkerke NJ. Natural history of tuberculosis: duration and fatality of untreated pulmonary tuberculosis in HIV negative patients: a systematic review. PLoS One 2011, 6:e17601.

Tseng CL, Oxlade O, Menzies D, Aspler A, Schwartzman K. Cost-effectiveness of novel vaccines for tuberculosis control: a decision analysis study. BMC Public Health 2011, 11:55.

World Health Organization: Global tuberculosis control: epidemiology, planning, financing. WHO report 2009. Geneva: World Health Organization; 2009.

Variant Creutzfeldt-Jakob disease (vCJD)

The initial symptoms of variant Creutzfeldt-Jakob disease (vCJD) are usually psychiatric, most frequently depression, anxiety and withdrawal (Henry & Knight, 2002; Will & Ward, 2004). After a median of six months, neurological features develop, including cognitive impairment, ataxia and involuntary movements. The clinical course is progressive with the development of dementia and diffuse cortical deficits.

Death occurs after a median of 14 months from the onset of symptoms (range 6–39 months) and is often due to an intercurrent infection (Will & Ward, 2004). However, Henry and Knight stated that the disease is fatal after a median of 13 months and a range of 6–39 months (Henry & Knight, 2002).

In the study by Hilton (Hilton, 2006) the mean age at death for vCJD is 26 years and 29 years with a range of 12–74 years (Will & Ward, 2004; Smiths, 2004). This is in line with the overall median age of 28 at death for all vCJD diagnoses in the UK during the period January 1994– December 2009, with a range from 14 to 75 (Andrews, 2010). During the epidemic, the median age of onset did not change over time, suggesting an important age-related risk. This could be due to an age-dependent susceptibility, age-related exposure or both (Hilton, 2006). There is no significant difference in deaths between males and females (56% male, p=0.12).

Precise estimates of the length and variability of the incubation period for vCJD are difficult to obtain since they require knowledge of the time of infection, whereas exposure may have occurred over several years. Ghani assumes that the incubation period is approximately 15–18 years (Ghani, 2002), whereas Collinge concludes that the incubation period would be at least 11 years (Collinge, 1999).

Although a peak has passed, it is possible that there will be future peaks, possibly in other genetic groups. To date, all cases of vCJD have been genotyped as methionine homozygous at codon 129 of the PrP gene (about 40% of the population). If the other 60% of the population is not completely resistant to infection, the disease in these individuals is associated with a longer incubation period, therefore epidemics in this group may still occur (Smith, 2004). Kaski et al. reported the first suspected clinical case of vCJD in an individual heterozygous for methionine/valine (Kaski, 2009).

There is also the possibility of ongoing person-to-person transmission, as seen with three cases of vCJD infection following transfusion of packed red blood cells from asymptomatic donors who subsequently died from vCJD (Ironside, 2010). Furthermore, Peden et al. described a vCJD infection in the first known asymptomatic patient (Millar, 2010; Peden, 2010). The patient died from unrelated pathology with no evidence of neurological diseases. The infection was detected in a study of autopsy and biopsy materials from 17 neurologically asymptomatic patients with haemophilia, considered to be at increased risk of vCJD. The most likely route of infection was receipt of UK plasma products.

Finally, Smith assumes that the ascertainment of vCJD cases in young adults is nearly complete. In the absence of a reliable, minimally invasive, diagnostic test, the possibility remains that cases in the elderly are being missed due to the small proportion of those dying with dementia that are subject to post-mortem examination (Smiths, 2004).

Model input summary

Table 1. Transition probabilities and distributions used in the outcome tree

Health outcome<br><br>(Health state)	Percent of health<br><br>outcome in health state	Transition probability	Source/assumption
Fatal cases following symptomatic infection		100%	

Table 2. Disability weights and duration

Health outcome (Health state)	Disability Weight (DW) (Haagsma, 2015)		Duration	
	DW	Label	In years	Source
Symptomatic infection	0.407 (0.36–0.46)	Motor plus cognitive impairments, severe.	1.151 (0.5–3.205)	Will & Ward, 2004

References

Ang LW, Chua LT, James L, Goh KT. Epidemiological surveillance and control of rubella in Singapore, 1991-2007. Ann Acad Med Singapore. 2010 Feb;39(2):95-101.

Andrews NJ. Incidence of variant Creutzfeldt-Jakob disease diagnoses and deaths in the UK, January 1994 - December 2009. 2010 [updated 22 June 2010]; Available from: http://www.cjd.ed.ac.uk/cjdq64jun10.pdf.

Collinge J. Variant Creutzfeldt-Jakob disease. Lancet. 1999 Jul 24;354(9175):317-23.

Ghani AC. The epidemiology of variant Creutzfeldt-Jakob disease in Europe. Microbes Infect. 2002 Mar;4(3):385-93.

Haagsma JA, Maertens de Noordhout C, Polinder S, Vos T, Havelaar AH, Cassini A, Devleesschauwer B, Kretzschmar ME, Speybroeck N, Salomon JA. Assessing disability weights based on the responses of 30,660 people from four European countries. Population Health Metrics 2015; 13: 10

Henry C, Knight R. Clinical features of variant Creutzfeldt-Jakob disease. Rev Med Virol. 2002 May-Jun;12(3):143-50.

Hilton DA. Pathogenesis and prevalence of variant Creutzfeldt-Jakob disease. J Pathol. 2006 Jan;208(2):134-41.

Ironside JW. Variant Creutzfeldt-Jakob disease. Haemophilia. 2010 Jul;16 Suppl 5:175-80.

Kaski D, Mead S, Hyare H, Cooper S, Jampana R, Overell J, et al. Variant CJD in an individual heterozygous for PRNP codon 129. Lancet. 2009 Dec 19;374(9707):2128.

Millar CM, Connor N, Dolan G, Lee CA, Makris M, Wilde J, et al. Risk reduction strategies for variant Creutzfeldt-Jakob disease transmission by UK plasma products and their impact on patients with inherited bleeding disorders. Haemophilia. 2010 Mar;16(2):305-15.

Peden A, McCardle L, Head MW, Love S, Ward HJ, Cousens SN, et al. Variant CJD infection in the spleen of a neurologically asymptomatic UK adult patient with haemophilia. Haemophilia. 2010 Mar;16(2):296-304.

Smith PG, Cousens SN, d' Huillard Aignaux JN, Ward HJ, Will RG. The epidemiology of variant Creutzfeldt-Jakob disease. Curr Top Microbiol Immunol. 2004;284:161-91.

Will RG, Ward HJ. Clinical features of variant Creutzfeldt-Jakob disease. Curr Top Microbiol Immunol. 2004;284:121-32.

Campylobacteriosis

Acute gastroenteritis associated with Campylobacter infections in humans is in most cases self-limiting after a few days to weeks, but for some patients the disease may be fatal. When available, information on duration of illness mainly relates to cases having requested medical help. These cases are often the most severe cases of longer duration. For example, the overall mean duration of illness due to Campylobacter

infection observed in the GP[*] case control component of the IID study in England and Wales was 9.34 days, whereas it was only 6.52 days for all cases observed in the community component (Adak, 2002). About 47% of the community component cases would have visited their GP (Food Standards Agency, 2000). Based on the IID study, it is has been assumed that gastroenteritis caused by campylobacteriosis would last 3.22 days (no medical help), 9.72 days (visiting GP) and 14.39 days (hospitalised) (Mangen, 2004; Mangen, 2005). In our current model we chose to apply 3.22–9.72 days for all uncomplicated cases and 14.39 days for the complicated ones.

Bacteraemia is highlighted in many reports as a possible extra-intestinal complication of campylobacteriosis. For example, Skirrow et al. (Skirrow, 1993) estimated a bacteraemia incidence of 1.5 per 1 000 reported campylobacteriosis cases, whereas Ternhag et al. (Ternhag, 2008) reported an absolute risk of bacteraemia/sepsis of 0.02% for laboratory-confirmed campylobacteriosis cases.

Assuming that GP visits represent an indication of moderate diarrhoea and that the proportion of hospitalised cases represents severe diarrhoea, we divided cases into the following groups: uncomplicated (mild diarrhoea) 75.5%, complicated (GP, moderate diarrhoea) 23.5% and complicated hospitalised (severe diarrhoea) cases 1% (Kemmeren, 2006; Kwong, 2012; redistributing to total 100%).

Estimates of campylobacteriosis case fatality proportions range from 0.001% to 0.05%: 0.05% (Mead, 1999), 0.024% of all foodborne campylobacteriosis cases in the IID study (Adak, 2002), 2–6% of the hospitalised cases (Buzby, 1996; corresponding to 0.012–0.036% of all cases, considering that 0.6% of cases are hospitalised according to Mangen et al. 2004), 1.3 fatal cases per year, corresponding to 0.001% of the estimated 123 000 Campylobacter cases (Cressey & Lake, 2007), 0.038% of all symptomatic cases (Mangen, 2004).

We chose to estimate the overall case fatality proportion as being within the range 0.001–0.05% and assumed a different age-group distribution of this risk based on the age-group distribution of reported deaths to TESSy between 2009 and 2013 (see Table 3). This table is based on all TESSy notified cases from EU Member States except Bulgaria, reporting only aggregate data, Greece, Portugal and Liechtenstein which do not report.

Risk of complications

Reactive arthritis (ReA), irritable bowel syndrome (IBS) (but not inflammatory bowel disease due to lack of confirmation of a biological link and limited evidence) and Guillain-Barré syndrome (GBS) may be associated with campylobacteriosis.

Reactive arthritis (ReA)

ReA is a significant long-term sequelae following campylobacteriosis (Keat, 1983; Johnsen, 1983; Hannu, 2002). A retrospective study carried out in Finland found that 7.4% (45/609) of laboratory-confirmed campylobacteriosis cases fulfilled the criteria for ReA (Hannu, 2002), which is similar to that found by another study: 8.1% (3/37) (Johnsen, 1983). A further study reported a 2.6% (9 of 350) frequency of ReA in patients contacting a municipal health centre following an outbreak of C. jejuni (Hannu, 2004) and 16% of laboratory-confirmed cases self-reported having had ReA (Locht & Krogfeld, 2002), although self-reporting might be prone to overestimation (Hannu, 2002). Other studies including clinical testing report a 2.8% and a 2.4% risk of developing rheumatological symptoms (Rees, 2004; Kosunen, 1980). In order to account for the large uncertainty, the risk of developing ReA from all symptomatic cases is 1.7% (0.73–4.4%) (Kemmeren, 2006).

Little is known about the duration of ReA; the average duration is between 1.5 months derived from Hannu et al. (Hannu, 2005) and 222 days (Kemmeren, 2006).

Irritable Bowel Syndrome (IBS)

In a recent literature review, 8.8% (7.2–10.4%) of symptomatic campylobacteriosis symptomatic cases were considered at risk of developing IBS, irrespective of age and gender; the duration was set to five years (Haagsma, 2010). However, the causality is largely debated and the impact of concurrent factors significant. Therefore, IBS is not considered as part of the campylobacteriosis outcome tree in our study.

Guillain-Barré syndrome (GBS)

GBS is a neurological disease frequently preceded by an acute infectious illness, mainly upper respiratory infections and gastrointestinal infections. The functional status of patients with GBS is scored on a seven-point disability scale (F-score), ranking from 0 (healthy) to 6 (death). GBS-patients with an F-score at nadir of < 3 (able to walk unaided at nadir) are considered to be mildly affected. GBS patients with an F-score of ≥ 3 (unable to walk unaided at nadir) are considered to be severely affected (van Koningsveld, 2001). Paralysis from GBS is generally reversible over time, but some patients are bedridden for life and others die prematurely.

Incidence is estimated at 0.8–2.0 or 0.4–4 cases per 100 000 persons year (van Koningsveld, 2001; Mc Grogan, 2009; Hughes & Rees, 1997) in Europe and North America. A systematic review of the literature and metanalysis estimated an age-specific GBS rate per 100 000 person years of exp[-12.0771 + 0.01813(age in years)] × 100 000 (Sejvar, 2011).

Studies show that 14–36% of GBS patients previously had a Campylobacter infection (Jacobs, 1998); 33–50% of GBS patients had increased levels of Campylobacter spp. (Mishu, 1993). A more recent systematic literature review estimated that 31% of the 2 502 GBS cases studied were attributable to Campylobacter infection (Poropatich, 2010).

Research has found that about 0.022% of laboratory-confirmed campylobacteriosis cases would develop GBS (13/57,425) (Ternhag, 2008), resulting for all symptomatic cases in a 0.0015% risk of developing GBS; in Sweden one GBS case per 3 285 Campylobacter jejuni infections (95% C.I.: 1.729 – 7.210) resulting in a risk of 0.03% (McCarthy & Giesecke, 2001); in the USA one per 1 058 campylobacteriosis cases (0.09% risk; Allos, 1997). Studies estimating the burden of campylobacteriosis assumed a 0.075% and 0.023% risk of developing GBS (Mangen, 2004 and 2005; Cressy & Lake, 2007). Given the large diversity found in the literature, the risk of developing GBS following a symptomatic Campylobacter infection is set to 0.0015–0.09%.

Males were more commonly affected by GBS in almost all studies (Sedano, 1994; Hughes & Rees, 1997; Nachamkin, 1998; Nagpal, 1999; van Koningsveld, 2000; Sejvar, 2011). However, these differences might be based on environmental factors as well as biological factors (van Koningsveld, 2000) and therefore it is difficult to speculate about the origin of this gender difference and the cause and determinants of GBS and therefore we do not distinguish in risk between genders.

Havelaar et al. (Havelaar 2000 a,b) estimated the proportion of mild and severe GBS cases after Campylobacter infections to be 17% and 83%, respectively. Age plays a role (van Koningsveld et al., 2000; Sejvar et al., 2011), we therefore assume that the age-group-specific distribution of the risk of developing a mild GBS is 17% and a severe GBS is 83% – see Table 4 and 5 (Havelaar 2000a, b). A total of 69% of mild GBS cases are under the age of 50, whereas for severe GBS cases this is only 48%.

The clinical course of GBS is highly variable. Very limited information is available for mildly affected patients. About 50% of the patients recover fully after six months, and the others have an F-score of 1. Most will recover after one year and the remainder will only suffer from minor symptoms (Havelaar, 2000a). We therefore assumed that mild cases will recover fully after one year.

There is a high heterogeneity among the severely-affected GBS patients: 60% of patients are reported to have an F-score of 4 when hospitalised, and approximately 20% of the patients had an F-score of 5 at nadir (Van der Meché, 1992). All patients recovered from intensive care, but after six months, 17% of them still had an F-score of 3 or 4. In a follow-up study the residual symptoms were evaluated up to six years after onset (Bernsen, 1997): only 25% recovered fully, whereas 44% of patients continued to suffer from minor symptoms (F-score=1) and 31% had functional limitations (F-score 2-4). Given that there had been no significant improvement since the acute phase, we assume that 17–31% of severely affected GBS patients would have permanent sequelae; this risk is distributed by age groups, see Table 6 (Havelaar 2000 a;b).

The case fatality rate for GBS ranges from 2–5% (Havelaar, 2000a) to 3.4% in a retrospective study (Van Koningsveld, 2000). However, generally only the severe cases are at risk of dying, therefore the risk is only estimated for these cases (CFR/83% severe cases x 100): 4.1% (2.41–6.02). The case fatality rate is age-dependent (Havelaar, 2000a) and strictly linked to the risk of developing permanent disabilities due to GBS; therefore, we apply the same age-group distribution as the risk of dying, see Table 6).

Model input summary

Table 1. Transition probabilities used in the outcome tree

Health outcome<br>(Health state)	Distribution of health states in health outcome	Transition probability	Source/assumption
Symptomatic infection<br>(Uncomplicated)<br>(Complicated, GP)<br>(Complicated, hosp)	 76%<br>23%<br>1%		Kemmeren, 2006; Kwong, 2012
Fatal cases following symptomatic infection		0.001–0.05%<br>Age dep. Table 3	Adak, 2002; Cressey & Lake, 2007; Mangen, 2005; Mead, 1999; TESSy 2009-2013
Reactive arthritis		1.7% (0.73–4.4%)	Kemmeren, 2006

Guillain-Barré syndrome<br>(Mild)<br><br>(Severe)	17%<br>Age dep. Table 4<br><br>83%<br>Age dep. Table 5	0.0015–0.09%	Allos, 1987; Ternhag, 2008; Havelaar 2000a, b
Fatal cases following severe GBS		4.1% (2.41–6.02%)<br>Age dep. Table 6	Koningsveld, 2001; Havelaar, 2000a<br>Assuming only severe cases are fatal
Permanent disability following GBS		17–31%<br>Age dep. Table 6	Havelaar, 2000a, b<br>Assuming only severe cases

Table 2. Disability weights and duration

Health outcome (Health state)	Disability Weight (DW) (Haagsma, 2015)		Duration	
	DW	Label	In years	Source/assumption

Symptomatic infection<br><br>(Uncomplicated)<br>(Complicated, GP)<br>(Complicated, hosp)	 <br><br>0.073 (0.061–0.092)<br>0.149 (0.12–0.182)<br>0.239 (0.202–0.285)	 <br><br>Diarrhoea, mild<br>Diarrhoea, moderate<br>Diarrhoea, severe	 <br><br>0.009<br>0.027<br>0.039	Food Standard Agency, 2000; Mangen, 2004, 2005
Reactive arthritis	0.344 (0.3-0.391)	Musculoskeletal problems, generalized, moderate	0.131-0.608	Hannu, 2002; Kemmeren, 2006
Guillain-Barré syndrome<br>(Mild)<br>(Severe)	 <br>0.053 (0.042-0.064)<br><br>0.520 (0.465-0.581)	 <br>Motor impairment, moderate<br><br>Spinal cord lesion at neck level (treated)	 <br>1<br>1	Havelaar, 2000a, b
Permanent disability following GBS	0.421 (0.377-0.477)	Motor impairment, severe	Remaining life expectancy	Van der Meché, 1992; Bernsen, 1997

Table 3. Age-group distribution of the case fatality rate (0.001–0.05%)

Age groups	%
0	0.54
1-4	1.09
5-9	3.26
10-14	1.63
15-19	0.54
20-24	4.35
25-29	5.98
30-34	1.63
35-39	3.26
40-44	3.80
45-49	3.80
50-54	5.43

55-59	5.98
60-64	5.98
65-69	8.15
70-74	6.52
75-79	11.96
80-84	11.96
>85	14.13
All ages	100.00

Table 4. Age distribution mild GBS

Age	%
0	0.63
01-04	5.02
05-09	2.51
10-14	1.25
15-19	6.27
20-24	6.90

25-29	10.04
30-34	9.41
35-39	9.41
40-44	8.78
45-49	8.78
50-54	5.17
55-59	4.82
60-64	4.13
65-69	5.51
70-74	5.17
75-79	4.13
80-84	0.69
85+	1.38
Total	100

Table 5. Age distribution – severe GBS

Age	%
0	0.44
01-04	3.49
05-09	1.75
10-14	0.87
15-19	4.36
20-24	4.80
25-29	6.98
30-34	6.55
35-39	6.55
40-44	6.11
45-49	6.11
50-54	8.67
55-59	8.09
60-64	6.93
65-69	9.24
70-74	8.67
75-79	6.93

80-84	1.16
85+	2.31

Total	100

Table 6. Age distribution permanent GBS and case fatality rate

Age	%
0	0.00
01-04	0.00
05-09	0.00
10-14	0.00
15-19	0.00
20-24	1.56
25-29	1.56
30-34	1.56
35-39	1.56
40-44	2.08
45-49	2.08
50-54	2.08
55-59	6.25
60-64	6.25
65-69	6.25
70-74	18.75
75-79	25.00
80-84	18.75
85+	6.25
Total	100.00

References

Adak GK, Long SM, O'Brien SJ (2002). Trends in indigenous foodborne disease and deaths, England and Wales: 1992 to 2000. Gut 2002. 51:832- 841.

Allos BM. (1997). Association between Campylobacter infection and Guillain-Barré syndrome. J Infect Dis 1997.176(Suppl 2): S125-S128.

Bernsen RA, Jacobs HM, de Jager AE, van der Meché FGA (1997). Residual health status after Guillain-Barré syndrome. J. Neurol Neurosurg Psychiatry 1997. 62:637-640.

Buzby JC, Roberts T, Lin JCT, MacDonald JM (1996). Bacterial Foodborne Disease – Health care costs & Productivity losses. Washington, USA: Economic Research service, USDA. Agricultural Economic Report No 741.

Cressey P J and Lake R (2007). Risk ranking: Estimates of the burden of foodborne disease for New Zealand. Institute of Environmental Science & Research Limited; Christchurch Science Center.

Food Standards Agency (2000). A Report of the Study of Infectious Intestinal Disease in England. Food Standards Agency London: HMSO.

Haagsma JA, Havelaar AH, Janssen BM, Bonsel GJ. Disability Adjusted Life Years and minimal disease: application of a preference-based relevance criterion to rank enteric pathogens. Popul Health Metr 2008;6:7.

Haagsma JA, van der Zanden BP, Tariq L, van Pelt W, van Duynhoven YTHP, Havelaar AH (2009). Disease burden and costs of selected foodborne pathogens in the Netherlands, 2006. Bilthoven: National Institute of Public Health and Environment. RIVM rapport 330331001. Available online at: www.rivm.nl/bibliotheek/rapporten/330331001.pdf

Haagsma JA, Siersema PD, De Wit NJ, Havelaar AH. Disease burden of post-infectious irritable bowel syndrome in the Netherlands. Epidemiol Infect 2010.138(11):1650-6.

Haagsma JA, Maertens de Noordhout C, Polinder S, Vos T, Havelaar AH, Cassini A, Devleesschauwer B, Kretzschmar ME, Speybroeck N, Salomon JA. Assessing disability weights based on the responses of 30,660 people from four European countries. Population Health Metrics 2015; 13: 10

Hannu TL, Mattila H, Rautelin P, Pelkonen P, Lahdenne A, Siitonen M et al. Campylobacter-triggered reactive arthritis: a population-based study. Rheumatology 2002. 41(3):312-318.

Hannu TL, Kauppi M, Tuomala M, Laaksonen I, Klemets P, Kuusi M. Reactive arthritis following an outbreak of Campylobacter jejuni infection. J Rheumatol 2004. 31(3):528-30.

Havelaar AH, de Wit MAS, van Koningsveld R, van Kempen E (2000a). Health burden in the Netherlands due to infection with thermophilic Campylobacter spp. Epidemiol Infect 2000. 125(3):505-22.

Havelaar AH, de Wit MAS, van Koningsveld R (2000b). Health burden in the Netherlands (1990-1995) due to infection with thermophilic Campylobacter species. Bilthoven, the Netherlands: National Institute for Public Health and the Environment. Report no. 284550004.

Havelaar AH, Haagsma JA, Mangen MJJ, Kemmeren JM, Verhoef LPB, Vijgen SMC, et al. (2012a). Disease burden of foodborne pathogens in the Netherlands, 2009. International Journal of Microbiology in press.

Havelaar AH, Ivarsson S, Löfdahl M, Nauta MJ (2012b). Estimating the true incidence of campylobacteriosis and salmonellosis in the EU. Submitted.

Helms M, Simonsen J, Molbak K. Foodborne bacterial infection and hospitalization: a registry-based study. Clin Infect Dis 2006.42(4):498-506.

Hughes RAC and Rees JH (1997). Clinical and epidemiologic features of Guillain-Barré syndrome. Journal of infectious diseases 1997. 176 (suppl 2): S92-S98.

Jacobs BC, Rothbarth PH, van der Meché FG, Herbrink P, Schmitz PI, de Klerk MA, et al. (1998). The spectrum of antecedent infections in Guillain-Barré syndrome: a case-control study. Neurology 1998. 51(4):1110-1115.

Jess T, Simonsen J, Nielsen NM, Jørgensen KT, Bager P, Ethelberg S et al. (2011). Enteric Salmonella or Campylobacter infections and the risk of inflammatory bowel disease. Gut 2011;60(3):318-324.

Johnsen K, Ostensen M, Melbeye AC, Melby K (1983). HLA-B27-negative arthritis related to Campylobacter jejuni enteritis in three children and two adults. Acta Med Scand 1983. 214(2): 165-168.

Kemmeren JM, Mangen MJJ, van Duynhoven YTHP, Havelaar AH. (2006). Priority setting of foodborne pathogens: disease burden and costs of selected enteric pathogens. Bilthoven: National Institute of Public Health and Environment: RIVM rapport 330080001. Available online at: www.rivm.nl/bibliotheek/rapporten/330080001.pdf

Keat A. (1983). Reiter's syndrome and reactive arthritis in perspective. N Engl J Med 1983. 309(26):1606-15.

Kosunen TU, Kauranen O, Martio J, Pitkanen T, Ponka A, Hortling L, et al. (1980). Reactive arthritis after campylobacter jejuni enteritis in patients with HLA-B27. Lancet 1980. 1 (8181):1312-3.

Kwong JC, Ratnasingham S, Campitelli MA, Daneman N, Deeks SL, et al. (2012) The Impact of Infection on Population Health: Results of the Ontario Burden of Infectious Diseases Study. PLoS ONE 7(9): e44103. doi:10.1371/journal.pone.0044103

Lake RJ, Cressey PJ, Campbell DM, Oakley E (2010). Risk ranking for foodborne microbial hazards in New Zealand: burden of disease estimates. Risk Analysis 2010. 30(5):743-52.

Locht H, Krogfelt KA (2002). Comparison of rheumatological and gastrointestinal symptoms after infection with Campylobacter jejuni/coli and enterotoxigenic Escherichia coli. Ann Rheum Dis 2002.61(5): 448-52.

Mangen MJJ, Havelaar AH, de Wit GA. (2004). Campylobacteriosis and sequelae in the Netherlands - Estimating the disease burden and the cost-of-illness. Bilthoven: National Institute for Public Health and the Environment; Report nr. 250911004. Available online at: www.rivm.nl/bibliotheek/rapporten/250911004.pdf

Mangen MJJ, Havelaar AH, Bernsen RAJAM, van Koningsveld R, de Wit GA (2005). The costs of human Campylobacter infections and sequelae in the Netherlands: A DALY and cost-of-illness approach. 2005. Acta Agriculturae Scandinavica Section C Food Economics 2005. 2(1):35-51.

McCarthy N, Giesecke J (2001). Incidence of Guillain-Barre syndrome following infection with Campylobacter jejuni. Am J Epidemiol 2001. 153 (6): 610-614.

McGrogan A, Madle GC, Seaman HE, de Vries CS (2009). The epidemiology of Guillain-Barré syndrome worldwide. A systematic literature review. Neuroepidemiology 2009. 32(2):150-163.

Mead PS, Slutsker L, Dietz V, McCaig LF, Bresee JF, Shapiro C, et al. (1999). Food-related illness and death in the United States. Emerg Infect Dis 1999.5(5): 607-625.

Melse JM, Kramers PGN (1998). Berekening van de ziektelast in Nederland. Achtergronddocument bij VTV-1997; deel III, hoofdstuk 7. Bilthoven; Report nr. 431501028.

Mishu B, Ilyas AA, Koski CL, Vriesendorp F, Cook SD, Mithen FA, et al. (1993). Serological evidence of previous Campylobacter jejuni infection in patients with the Guillain-Barré Syndrome. Ann Intern Med 1993. 118(12): 947-953.

Nachamkin I, Allos BM, Ho T. (1998). Campylobacter species and Guillain-Barré syndrome. Clin Microbiol Rev 1998. 11(3): 555-567.

Nagpal S, Benstead T, Shumak K, Rock G, Brown M, Anderson DR (1999). Treatment of Guillain-Barré syndrome: a cost-effectiveness analysis. J. Clin Apheresis 1999. 14(3):107-113.

Poropatich KO, Walker CL, Black RE (2010). Quantifying the association between Campylobacter infection and Guillain-Barré syndrome: a systematic review. J. Health Popul Nutr; 2010. 28(6):545-52.

Sedano MJ, Calleja J, Canga E, Berciano J (1994). Guillain-Barré syndrome in Cantabria, Spain. An epidemiological and clinical study. Acta Neurol Scand 89(4): 287-292.

Sejvar JJ, Baughman AL, Wise M, Morgan OW (2011). Population incidence of Guillain-Barré syndrome: a systematic review and meta-analysis. Neuroepidemiology; 2011 36(2):123-133.

Skirrow MB, Jones DM, Sutcliffe E, Benjamin J (1993). Campylobacter bacteraemia in England and Wales, 191-91. Epidemiology and Infection 110 (3): 49-57.

Tam CC, Rodrigues IC, Viviani L, Dodds JP, Evans MR, Hunter PR et al. Longitudinal study of infectious intestinal disease in the UK (IID2 study): incidence in the community and presenting to general practice. Gut. 2012 Jan;61(1):69-77

Ternhag A, Törner A, Svensson A, Ekdahl K, Giesecke J (2008). Short- and long-term effects of bacterial gastrointestinal infections. Emerg Infect Dis 2008.14 (1):143-8.

Van der Meché FGA, Schmitz PIM and Dutch Guillain-Barré Study Group (1992). A randomized trial comparing intravenous immune globulin and plasma exchange in Guillain-Barré syndrome.N Engl. J. Med. 1992 326:1123-1129.

Van Koningsveld R, van Doorn PA, Schmitz PIM, Ang CW, van der Meché FGA (2000). Mild forms of Guillain-Barré syndrome in an epidemiological survey in the Netherlands. Neurology 54: 620-625.

Van Koningsveld R. (2001). Epidemiological and clinical aspects of the Guillain-Barré syndrome. Rotterdam, the Netherlands: Erasmus University Rotterdam. PhD-Thesis.

WHO (2004). Global Burden of Disease 2004 Update: Disability weights for diseases and conditions. Geneva: WHO. Available online at: http://www.who.int/healthinfo/global_burden_disease/GBD2004_DisabilityWeights.pdf

Yu DTY, Thomson GTD (1994). Clinical, epidemiological and pathogenic aspects of reactive arthritis. Food-Microbiology 1994. 11(2):97-108.

Chlamydia

Chlamydia trachomatis is a bacterium that causes a sexually transmitted infection (STI). WHO estimates a global annual incidence of about 90 million cases. Chlamydia trachomatis affects both women and men and can cause severe harm to the reproductive system of women. Additionally, children born to infected mothers are at high risk of developing severe complications (e.g. ophthalmia neonatorum, pneumonia). C. trachomatis has various serovars with different transmission modes and consequences. Serovars A, B, Ba and C, often transmitted by close eye- to-eye contact, cause ocular trachoma and are responsible for about 7–9 million cases of blindness (Stamm, 2005). Serovars D–K, responsible for genital infections, are associated with various adverse health outcomes in both men and women (Carey & Beagley, 2010). Serovars L1, L2 and L3 cause Lymphogranuloma venerum, a systemic STI mainly observed in the high-risk group of men having sex with men (MSM) (Martin- Iguacel, 2010). For the current outcome trees only serovars D–K responsible for genital infection are taken into consideration.

C. trachomatis mostly affects the young and sexually-active population with a female-male sex ratio of 1:0.7 (in tested individuals) (ECDC, 2014a). The genito-urinary infections present different disease patterns in the female and male hosts.

The asymptomatic infection poses serious threats to the health of the population as asymptomatic carriers represent a pool for new infections, and asymptomatic infections are associated with the risk of developing severe sequelae.

Rates of asymptomatic cases reported in literature vary widely. More than 50% of the infections due to C. trachomatis in males do not produce any symptoms or present a mild symptomatic illness (van de Laar & Morre, 2007). In a study of male army recruits, 85.6% of men testing positive for Chlamydia reported no symptoms (Cecil, 2001). Comparable rates were also reported by McKay and colleagues, with 88% of infected men being asymptomatic (McKay, 2003). Long-term sequelae due to chronic asymptomatic infections in men are still under discussion, but the pool of asymptomatic C. trachomatis carriers poses a serious threat to women's health due to continuous transmission and re-infection. Gaydos and Quinn refer to a percentage of asymptomatic male cases above 50%, in line with the above-mentioned estimates (Gaydos & Quinn, 2012).

Genital infections in women may present with short-term acute symptoms of cervicitis and urethritis (Stamm, 2005). Women also face a high number of asymptomatic infections. In total, 70–90% of all female and 50–88% of all male chlamydial infections do not present any symptoms (Stamm, 2005; Gaydos, 1998; Kalwij, 2010). Quinn and colleagues noted that around 79% of women with a Chlamydia infection attending a STI clinic were asymptomatic (Quinn, 1996). Clinical textbooks report a range of 70–90% of female cases being asymptomatic (Stamm, 2005; Gaydos & Quinn, 2012).

For the model we decided to use a range of 70–90% for the asymptomatic proportion (Stamm, 2005; Gaydos & Quinn, 2012) for female and 50– 88% for male cases (Stamm, 2005; Gaydos, 1998; Kalwij, 2010).

Health outcomes associated with chlamydial infection

Genital infection in men

Urethritis: with an incubation period of 7–14 days, urethritis causes symptoms of dysuria and urethral discharge (Stamm, 2005). We selected a range of 12–50% of infected men to represent symptomatic cases developing non-gonococcal urethritis (NGU) (Carey & Beagley, 2010; McKay, 2003).

Epididymitis: epididymitis is an acute inflammation of the epididymis (Carey & Beagley, 2010). The symptoms are oligospermia during the acute phase, swollen epididymis (and/or testicles), and dysuria. Fever and chills may occur in some cases. The association between epididymitis and future (in)fertility is an ongoing debate in research with no clear indication (Stamm, 2005).

Proctitis and proctocolitis: this clinical picture is most common in the MSM community. The classic symptoms are rectal pruritus, -pain and - bleeding. Fever often accompanies the initial proctitis and proctocolitis (Stamm, 2005; Carey & Beagley, 2010). This health outcome was not considered in the model due to lack of information.

Reactive arthritis: a further clinical picture is sexually-acquired reactive arthritis occurring as an acute aseptic arthritis or presenting as Reiter's syndrome. Reiter's syndrome includes symptoms of arthritis, conjunctivitis, urethritis and skin lesions (Stamm, 2005; Keat, 1983).

Genital infection in men can also include chronic pelvic pain. However, due to lack of information we decided not to include it in the model (Haggerty, 2010).

Genital infection in women

Urethritis/cervicitis

The acute form of C. trachomatis infection in women is urethritis and/or cervicitis. The majority of cases of both urethritis and cervicitis are asymptomatic, but can lead to severe sequelae (Low, 2007).

Pelvic inflammatory disease (PID)

Both symptomatic and asymptomatic infections can lead to serious consequences. Pelvic inflammatory disease is a commonly reported health outcome of a chlamydial infection. The literature shows very heterogeneous patterns regarding the transition probabilities from acute infection to PID. Carey and Beagley state that 12–50% of women infected with C. trachomatis develop PID (Carey & Beagley, 2010). In other literature the risk of PID after lower genital tract infection with Chlamydia varied from 0 to 30% (Risser & Risser, 2007) and from 0 to 72% (Boeke, 2005). Cates and Wasserheit reported that 40% of women with an untreated C. trachomatis infection develop PID (Cates & Wasserheit, 1991). Van Valkengoed and colleagues reported that complications of Chlamydia trachomatis infections are overestimated in the literature. They found five Cost Effectiveness Analyses (CEA) using decision trees to estimate the effect of screening programmes (Van Valkengoed, 2004). In these studies the estimates of the probability of developing PID after infection varied from 25 to 80%. ECDC has undertaken a systematic literature review and found a risk of developing PID from chlamydial infections of 9% (4–19%) (ECDC, 2014b).

Acute PID with pelvic pain, lasting for about 15 days, and silent PID with no or mild symptoms can cause severe long-term sequelae (Carey & Beagley, 2010; Westrom, 1980).

The estimated risk of tubal infertility as a sequelae of PID varies between 10–20% (Carey & Beagley, 2010; Lan, 1995; Land, 2010). Land and colleagues estimated the risk of tubal infertility after asymptomatic Chlamydia infection to be around 0.07% (Land, 2010). The risk of tubal infertility was found to be dependent on the course of infection (mild vs. severe) and the frequencies of re-infection (e.g. after three episodes of PID the risk is five-fold compared to a single episode.) ECDC's systematic review found that 16% of women with PID will develop infertility (ECDC, 2014b), which applies to women of reproductive age.

In total, 7–9% of pregnant women develop ectopic pregnancy after PID (Lan, 1995). Around 15% of women with previous PID develop chronic pelvic pain (Rogstad, 2008). Tubo-ovarian abscesses (tubal pathology) incur a risk of 7–16% for women who have previously had PID (Kottmann, 1995). The risk of cervical neoplasia is still under debate due to the fact that most cervical neoplasia are due to human papilloma virus (HPV) (Stamm, 2005).

Based on registration data from Amsterdam it was estimated that 0.07% and 0.02% of women exposed to chlamydia infection develop ectopic pregnancy and tubal factor infertility, respectively (Van Valkengoed, 2004).

Perinatal infections

Perinatal chlamydia may complicate as conjunctivitis (ophthalmia neonatorum) and neonatal pneumonia. We considered the ONBoID study for the input parameters which estimated that 15% of cases would develop ophthalmia neonatorum and 16% neonatal pneumonia (Kwong 2012). Assuming that in EU/EEA Member States all notified cases will have had symptoms, we used the same proportion: 48.39% are affected by ophthalmia and 51.61% will present pneumonia.

Outcome-tree parameters

Male outcome tree

For the male outcome tree a minimum of 50% and maximum of 88% was estimated as the percentage of asymptomatic cases (Carey & Beagley, 2010; McKay, 2003). The probability of developing epididymitis from symptomatic infections (10%) was taken from the World Health Organization STD Burden of Disease Study by Gerbase and colleagues (Gerbase, 2000). For asymptomatic infections a probability of 1–4% was taken from the cost effectiveness analysis of Welte and colleagues (Welte, 2001). Data on sexually acquired reactive arthritis (1% of symptomatic urethritis) and the resulting Reiters' syndrome (33% of reactive arthritis) were taken from a clinical text book (Stamm, 2005).

Female outcome tree

For the percentage of asymptomatic cases a range of 70–90% was included in the model (Stamm, 2005; Gaydos & Quinn, 2012; Gaydos, 1998; Kalwij, 2010; Stamm, 1999).

For the development of PID, estimates are included from the systematic review conducted by ECDC for the minimum (4%) (Van Valkengoed, 2004), maximum (19%) and most likely values (9%) (ECDC, 2014b).

The probability of developing ectopic pregnancy and tubal infertility after chlamydia infection is set to 0.07% and 0.02% respectively (Van Valkengoed, 2004). The probability of dying due to ectopic pregnancy was set to 0.038%, based on the study from Goldner (Goldner, 1993).

The risk of moving from PID to chronic pelvic pain was set at 18–75% and from PID to tubo-ovarian abscess at 0.8% (ECDC, 2014b; Ness, 2002, Soper 2010).

We decided to set the case fatality proportion for abscesses that have not ruptured to zero. Current mortality proportions for patients with ruptured abscesses are not reported in the literature; data from the 1960s suggested a mortality proportion ranging from 1.7 to 3.7 percent (Pedowitz, 2004; Paik, 2006). Due to the fact that these figures come from old studies and that diagnostics and treatment have significantly improved, we decided not to include the risk of dying from tubo-ovarian abscess.

Model input summary

Table 1. Transition probabilities and distributions used in the outcome tree

Health outcome (Health state)	Distribution of health states within health outcome	Transition probability	Source/assumption
Men			
Symptomatic infection		12–50%	Carey & Beagley, 2010; McKay, 2003
Epididymitis following symptomatic infection		10%	Gerbase, 2000
Reactive arthritis		1%	Stamm, 2005
(Mild)	67%		Stamm, 2005
(Severe)	33%		Stamm, 2005
Epididymitis following asymptomatic infection		1–4%	Gerbase, 2000; Welte, 2001

Women<br><br>Symptomatic infection		10–30%	Stamm, 1999; Stamm, 2005; Gaydos & Quinn, 2012; Gaydos, 1998; Kalwij, 2010
Pelvic inflammatory disease (PID)		9% (4–19%)	ECDC, 2014b
Tubo-ovarian abscess from PID		0.8%	Ness, 2002
Chronic pelvic pain after PID		18–75%	ECDC, 2014b; Soper 2010
Ectopic pregnancy		0.07%<br>Age dep. See Table 4	van Valkengoed, 2004<br>Female reproductive age 15-49
Tubal Infertility		0.02%<br>Age dep. See Table 4	Land, 2010; ECDC, 2014b<br>Female reproductive age 15-49
Fatal cases following ectopic pregnancy		0.038%	Goldner, 1993
Perinatal			
Symptomatic infection<br>(Neonatal pneumonia) (Ophthalmia neonatorum)	48.39%<br>51.61%		Kwong, 2012<br><br>Assuming that all reported cases have symptoms, we used the same proportion

Table 2. Disability weights and duration

Health outcome (Health state)	Disability Weight (DW) (Haagsma, 2015)			Duration
	DW	ECDC European Disability Weight Project (2014)	In years	Source

Men				
Symptomatic infection	0.007 (0.005–0.01)	Infectious disease, acute episode, mild	0.02	Trojian, 2009
Epididymitis	0.176 (0.143–0.208)	Epididymo-orchitis	0.04	Murray, 1996
Reactive arthritis				
(Mild)	0.344 (0.3-0.391)	Musculoskeletal problems, generalised, moderate	0.13–0.28	Özgül, 2006; Hannu, 2002
		Musculoskeletal problems, generalised, severe	0.41	
(Severe)	0.518 (0.457–0.576)			Miehle, 2003
Women				
Symptomatic infection	0.007 (0.005–0.01)	Infectious disease, acute episode, mild	0.03	Murray, 1996
Pelvic inflammatory disease (PID)	0.018–0.310	Abdominopelvic problem, mild to severe	0.04	Westrom, 1980
Tubo-ovarian abscess	0.31 (0.262–0.355)	Abdominopelvic problem, severe	0.01	Goharkhay, 2007; Teisala, 1990
Chronic pelvic pain	0.018–0.123	Abdominopelvic problem, mild to moderate	2.8	Sharma, 2011
Ectopic pregnancy	0.31 (0.262–0.355)	Abdominopelvic problem, severe	0.08	Murray, 1996
Tubal infertility	0.007 (0.005–0.01)	Infertility, secondary	See Table 3	Female reproductive age 15-49 (See Table 4)

Perinatal				
Neonatal pneumonia	0.125 (0.104–0.152)	Infectious disease, acute episode, severe	0.038	Zar, 2005<br><br>Assuming two weeks of treatment
Ophthalmia neonatorum	0.015 (0.011–0.019)	Conjunctivitis without corneal scar	0.038	American Academy of Pediatrics, 2012.<br><br>Assuming two weeks of treatment

Table 3. Duration of tubal infertility (female outcome tree)

Age	Duration in years
15–19	32
20–24	27
25–29	22
30–34	17
35–39	12
40–44	7
45–49	2

Table 4. Age-group risk (only reproductive age)

Age	%
0–14	0
15–49	100
≥50	0

References

American Academy of Pediatrics. Chlamydia trachomatis. In: Pickering LK, editor. Red book: 2012. Report of the Committee on Infectious Diseases. Elk Grove Village, IL: American Academy of Pediatrics; 2012. pp. 276–81.

Boeke AJ, van Bergen JE, Morre SA, van Everdingen JJ: [The risk of pelvic inflammatory disease associated with urogenital infection with Chlamydia trachomatis; literature review]. Ned Tijdschr Geneeskd 2005, 149:878-884.

Carey AJ, Beagley KW: Chlamydia trachomatis, a hidden epidemic: effects on female reproduction and options for treatment. Am J Reprod Immunol 2010, 63:576-586.

Cates W Jr. Wasserheit JN: Genital chlamydial infections: epidemiology and reproductive sequelae. Am J Obstet Gynecol 1991, 164:1771-1781.

Cecil JA, Howell MR, Tawes JJ, Gaydos JC, McKee KT, Quinn TC, et al. Features of Chlamydia trachomatis and Neisseria gonorrhoeae Infection in Male Army Recruits. Journal of Infectious Diseases 2001, 184:1216-1219.

European Centre for Disease Prevention and Control. Sexually transmitted infections in Europe 2012: ECDC; 2014a.

European Centre for Disease Prevention and Control. Chlamydia control in Europe: literature review. Stockholm: ECDC; 2014b.

Gaydos CA, Howell MR, Pare B, Clark KL, Ellis DA, Hendrix RM, et al. Chlamydia trachomatis Infections in Female Military Recruits. New England Journal of Medicine 1998, 339:739-744.

Gaydos CA, Quinn TC. Chlamydial infections. In Harrison's Principles of Internal Medicine. Volume 2. 18th Edition. Edited by Longo D, Fauci A, Kasper D, Hauser S, Jameson J, Loscalzo J. New York: McGraw Hill; 2012: 1421-1432

Gerbase A, Stein C, Levison J, Htun Y: Global burden of sexually transmitted diseases (excluding HIV) in the year 2000. In: Global burden of sexually transmitted diseases (excluding HIV) in the year 2000 (Ed.). World Health Organization; 2000.

Goldner TE, Lawson HW, Xia Z, Atrash HK. Surveillance for ectopic pregnancy—United States, 1970–1989. MMWR CDC Surveill Summ. 1993;42:73–85.

Goharkhay N, Verma U, Maggiorotto F: Comparison of CT- or ultrasound-guided drainage with concomitant intravenous antibiotics vs. intravenous antibiotics alone in the management of tubo-ovarian abscesses. Ultrasound Obstet Gynecol 2007, 29:65-69.

Haggerty CL, Gottlieb SL, Taylor BD, Low N, Xu F, Ness RB. Risk of sequelae after Chlamydia trachomatis genital infection in women. J Infect Dis 2010, 201 Suppl 2:S134-155.

Haagsma JA, Maertens de Noordhout C, Polinder S, Vos T, Havelaar AH, Cassini A, Devleesschauwer B, Kretzschmar ME, Speybroeck N, Salomon JA. Assessing disability weights based on the responses of 30,660 people from four European countries. Population Health Metrics 2015; 13: 10

Hammerschlag MR, Chandler JW, Alexander ER, English M, Koutsky L. Longitudinal studies on chlamydial infections in the first year of life. The Pediatric Infectious Disease Journal 1982, 1:395-401.

Hammerschlag MR. Chlamydial infections. J Pediatr 1989, 114:727-734.

Hannu TL, Mattila H, Rautelin P, Pelkonen P, Lahdenne A, Siitonen M et al. (2002) Campylobacter-triggered reactive arthritis: a population- based study. Rheumatology 41(3): 312-318.

Kalwij S, Macintosh M, Baraitser P. Screening and treatment of Chlamydia trachomatis infections. BMJ 2010, 340.

Keat A, Thomas BJ, Taylor-Robinson D. Chlamydial infection in the aetiology of arthritis. Br Med Bull 1983, 39:168-174.

Kottmann LM. Pelvic inflammatory disease: clinical overview. J Obstet Gynecol Neonatal Nurs 1995, 24:759-767.

Kwong JC, Ratnasingham S, Campitelli MA, Daneman N, Deeks SL, et al. (2012) The Impact of Infection on Population Health: Results of the Ontario Burden of Infectious Diseases Study. PLoS ONE 7(9): e44103. doi:10.1371/journal.pone.0044103

Lan J, van den Brule AJ, Hemrika DJ, Risse EK, Walboomers JM, Schipper ME, et al. Chlamydia trachomatis and ectopic pregnancy: retrospective analysis of salpingectomy specimens, endometrial biopsies, and cervical smears. J Clin Pathol 1995, 48:815-819.

Land JA, Van Bergen JE, Morre SA, Postma MJ. Epidemiology of Chlamydia trachomatis infection in women and the cost-effectiveness of screening. Hum Reprod Update 2010, 16:189-204.

Low N, McCarthy A, Macleod J, Salisbury C, Campbell R, Roberts TE, et al. Epidemiological, social, diagnostic and economic evaluation of population screening for genital chlamydial infection. Health Technol Assess 2007, 11:iii-iv, ix-xii, 1-165.

Martin-Iguacel R, Llibre JM, Nielsen H, Heras E, Matas L, Lugo R, et al. Lymphogranuloma venereum proctocolitis: a silent endemic disease in men who have sex with men in industrialised countries. Eur J Clin Microbiol Infect Dis 2010, 29:917-925.

McKay L, Clery H, Carrick-Anderson K, Hollis S, Scott G. Genital Chlamydia trachomatis infection in a subgroup of young men in the UK. Lancet 2003, 361:1792.

Miehle W. Medikamentöse Therapie rheumatischer Erkrankungen. Stuttgart: Georg Thieme Verlag; 2003.

Murray CJL, Lopez AD. The Global Burden of Disease - A comprehensive assessment of mortality and disability from diseases, injuries and risk factors in 1990 and projected to 2020. Cambridge: Harvard School of Public Health on behalf of the World Health Organization and the World Bank; 1996.

Ness RB, Soper DE, Holley RL, Peipert J, Randall H, Sweet RL, Sondheimer SJ, Hendrix SL, Amortegui A, Trucco G, Songer T, Lave JR, Hillier SL, Bass DC, Kelsey SF (2002) Effectiveness of inpatient and outpatient treatment strategies for women with pelvic inflammatory disease: results from the Pelvic Inflammatory Disease Evaluation and Clinical Health (PEACH) Randomized Trial. Am J Obstet Gynecol 186: 929-937.

Özgül A, et al. (2006). Clinical presentations of chlamydial and non-chlamydial reactive arthritis. Rheumatology International 26(10): 879-885.

Paik CK, Waetjen LE, Xing G, et al. Hospitalizations for pelvic inflammatory disease and tuboovarian abscess. Obstet Gynecol 2006; 107:611.

Pedowitz P, Bloomfield Rd. Ruptured adnexal abcess (tuboovarian) with generalized peritonitis. Am J Obstet Gynecol 1964; 88:721.

Peipert JF. Clinical practice. Genital chlamydial infections. N Engl J Med 2003, 349:2424-2430.

Quinn TC, Gaydos C, Shepherd M, Bobo L, Hook EW, Viscidi R, et al. Epidemiological and microbiological correlates of Chlamydia trachomatis infection in sexual partnerships. JAMA: Journal of the American Medical Association 1996, 276:1737-1742.

Risser WL, Risser JM. The incidence of pelvic inflammatory disease in untreated women infected with Chlamydia trachomatis: a structured review. Int J STD AIDS 2007, 18:727-731.

Rogstad K. Complications in the female and their management. In: International handbook of Chlamydia. 3rd Edition. Edited by Moss T. Haslemere, UK: Alden Press; 2008: 111-121

Salomon JA, Haagsma JA, Davis A, Maertens de Noordhout C, Polinder S, Havelaar AH, et al. Disability weights for the Global Burden of Disease 2013 study. The Lancet Global Health. [Accepted, in press.]

Sharma D, Dahiya K, Duhan N, Bansal R. Diagnostic laparoscopy in chronic pelvic pain. Arch Gynecol Obstet 2011, 283:295-297.

Soper DE. Pelvic inflammatory disease. Obstetrics and Gynecology, vol. 116, no. 2, part 1, pp. 419–428, 2010.

Stamm WE, Jones RB, Batteiger BE. Chlamydia trachomatis (Trachoma, Prinatal Infections, Lymphogranuloma Venerum and other genital Infections). In: Principles and practice of infectious diseases. Volume 2, 6th Edition. Edited by Mandel GL, Bennet JE, Dolin R. Pennsylvania: Elsevier Inc.; 2005: 2239-2255

Stamm WE. Chlamydia trachomatis infections of the adult. In: Sexually transmitted diseases. 3rd Edition. Edited by Holmes KK, Sparling PF, Mardh PA. New York: McGraw Hill; 1999:407–422

Trojian TH, Lishnak TS, Heiman D. Epididymitis and orchitis: an overview. Am Fam Physician 2009, 79:583-587.

Teisala K, Heinonen PK, Punnonen R. Transvaginal ultrasound in the diagnosis and treatment of tubo-ovarian abscess. Br J Obstet Gynaecol 1990, 97:178-180.

van de Laar MJ, Morre SA. Chlamydia: a major challenge for public health. Euro Surveill 2007, 12:E1-2.

van Valkengoed IG, Morre SA, van den Brule AJ, Meijer CJ, Bouter LM, Boeke AJ. Overestimation of complication rates in evaluations of Chlamydia trachomatis screening programmes--implications for cost-effectiveness analyses. Int J Epidemiol 2004, 33:416-425.

Welte R, Jager H, Postma MJ. Cost-effectiveness of screening for genital Chlamydia trachomatis. Expert Rev Pharmacoecon Outcomes Res 2001, 1:145-156.

Westrom L. Incidence, prevalence, and trends of acute pelvic inflammatory disease and its consequences in industrialized countries. Am J Obstet Gynecol 1980, 138:880-892.

Zar HJ. Neonatal chlamydial infections: prevention and treatment. Pediatric Drugs, vol. 7, no. 2, pp. 103–110, 2005

Cryptosporidiosis

Acute gastroenteritis associated with cryptosporidiosis in humans is in most cases self-limiting and symptoms disappear within a few days or weeks, but in very small number of cases the disease can be fatal.

We assumed that only a small proportion of cases (0.150%) experience the disease as more severe and complicated (Vijgen, 2007).

The average duration of the uncomplicated, mild disease is 3.5 days and 7–18.4 days for the complicated form (Vijgen, 2007).

The case fatality proportion was found to be 0.0042% (Vijgen, 2007), in line with 0.005% found in other studies (Mead, 1999). Mortality from acute gastroenteritis was assumed to be age-dependent and was redistributed according to the age-group-distributed cryptosporidiosis and giardiasis case fatality proportion reported to TESSy between 2009 and 2013 (see Table 3). This table is based on all TESSy notified cases from EEA Member States except Bulgaria, Poland (reporting only aggregate data), Austria, Czech Republic, Iceland, Luxembourg, Malta, Norway, Romania, Slovenia and Slovakia (because the very low incidence reported seems to indicate low sensitivity of the surveillance system).

Cryptosporidiosis can become chronic in immunocompromised persons, especially those with AIDS (Caccio and Pozio, 2006; Call, 2000; Pozio, 1997). However, several studies showed that AIDS-related cryptosporidiosis can be cured following successful antiretroviral therapy (Miao, 2000; Maggi, 2000; Foudraine, 1998).

Model input summary

Table 1. Transition probabilities and distributions used in the outcome tree

Health outcome (Health state)	Distribution of health states in health outcome	Transition probability	Source/assumption
Symptomatic infection			Vijgen, 2007
Uncomplicated)	99.85%		
Complicated)	0.15%		
Fatal cases following symptomatic infection		0.0042%<br>Age dependent (Table 3)	Vijgen, 2007;<br>TESSy 2009–2013

Table 2. Disability weights and duration

Health outcome (Health state)	Disability Weight (DW) (Haagsma, 2015)			Duration	

	DW	Label	In years		Source
Symptomatic infection					Vijgen, 2007
(Moderate)	0.073 (0.061–0.092)	Diarrhoea, mild	0.01		
(Severe)	0.239 (0.202–0.285)	Diarrhoea, severe	0.019–0.05		

Table 3. Age-group redistribution of case fatality proportion due to cryptosporidiosis (0.0042%)

Age groups	%
0	12.50
1–4	6.25
5–9	6.25
10–14	0.00
15–19	0.00
20–24	0.00
25–29	0.00
30–34	6.25

35–39	0.00
40–44	0.00
45–49	6.25
50–54	12.50
55–59	6.25
60–64	6.25
65–69	6.25
70–74	6.25
75–79	18.75
80–84	6.25
>85	0.00
All ages	100.00

References

Caccio SM, Pozio E (2006). Advances in the epidemiology, diagnosis and treatment of cryptosporidiosis. Expert Rev Anti Infect Ther. 4(3):429- 443.

Call SA, Heudebert G, Saag M, Wilcox CM (2000). The changing etiology of chronic diarrhea in HIV-infected patients with CD4 cell counts less than 200 cells/mm3. Am J Gastroenterol , 95(11):3142-3146.

Foudraine NA, Weverling GJ, van Gool T, Roos MT, de Wolf F, Koopmans PP, et al. (1998). Improvement of chronic diarrhoea in patients with advanced HIV-1 infection during potent antiretroviral therapy. Aids, 12(1):35-41.

Haagsma JA, Maertens de Noordhout C, Polinder S, Vos T, Havelaar AH, Cassini A, Devleesschauwer B, Kretzschmar ME, Speybroeck N, Salomon JA. Assessing disability weights based on the responses of 30,660 people from four European countries. Population Health Metrics 2015; 13: 10

Maggi P, Larocca AM, Quarto M, Serio G, Brandonisio O, Angarano G, et al. (2000) Effect of antiretroviral therapy on cryptosporidiosis and microsporidiosis in patients infected with human immunodeficiency virus type 1. Eur J Clin Microbiol Infect Dis, 19(3):213-217.

Mead PS, Slutsker L, Dietz V, McCaig LF, Bresee JS, Shapiro C, et al. (1999) Food-related illness and death in the United States. Emerg Infect Dis, 5(5):607-625.

Miao YM, Awad-El-Kariem FM, Franzen C, Ellis DS, Muller A, Counihan HM, et al. (2000) Eradication of cryptosporidia and microsporidia following successful antiretroviral therapy. J Acquir Immune Defic Syndr, 25(2):124-129.

Pozio E, Rezza G, Boschini A, Pezzotti P, Tamburrini A, Rossi P, et al. (1997) Clinical cryptosporidiosis and human immunodeficiency virus (HIV)- induced immunosuppression: findings from a longitudinal study of HIV-positive and HIV-negative former injection drug users. J Infect Dis, 176(4):969-975.

Vijgen SMC, Mangen MJM, Kortbeek LM, Van Duynhoven YTHP, Havelaar AH (2007). Disease burden and related costs of cryptosporidiosis and giardiasis in the Netherlands. 58 pp. Bilthoven.

Diphtheria

Thanks to vaccination, respiratory diphtheria has almost disappeared from many European countries. In total, 85% of patients suffer from subclinical disease or turn into asymptomatic carriers (Vitek, 1998) and only an estimated 15% of infections lead to a symptomatic case. The duration of acute illness was based on the Ontario Burden of Infectious Disease Study [AC1] ('the Ontario Study') [SW2] and set at 12 days (Kwong, 2012).

Risk of complications

Systemic toxicity (a toxic form of the disease with swelling of the neck) occurs in 8.1% of all diphtheria patients and may lead to complications such as myocarditis, neuropathies and renal failure (Rakhmanova, 1996). The more frequent complications of acute illness are myocarditis and polyneuropathies/nerve palsies. Other complications, such as sepsis, septic arthritis, pneumonia, otitis media, splenic and hepatic abscesses and rhinitis, were not included in the outcome tree because they are either extremely rare or mild.

Our model is based on the assumption that 8.1% of symptomatic patients would have a complicated form of the disease (Rakhmanova, 1996).

Permanent disability following myocarditis (arrhythmias)

Assuming that myocarditis represents 66.6% of the complicated diphtheria cases (Jayashree, 2006) and that 0.25% (Mandell, 1999) of these will develop permanent conduction defects (arrhythmias), the transition probability of patients with complications developing permanent cardiac disability is 0.17%.

Case fatality ratio

The US Centers for Disease Control and Prevention (US CDC) have reported a case-fatality proportion (CFP) of 5–10% for diphtheria, with higher death rates (up to 20%) among persons under five and over 40 years. The case fatality proportion has changed very little over the last 50 years (CDC, 2009).

In the model, the CFP associated with uncomplicated disease is 1% and with complicated disease 25.7% (Rakhmanova, 1996).

Model input summary

Table 1. Transition probabilities used in the outcome tree

Health outcome (Health state)	Distribution of health states in health outcome	Transition probability	Source/assumption
Symptomatic infection<br>(Uncomplicated)<br>(Complicated)	 91.9%<br>8.1%		Rakhmanova, 1996
Permanent disability (arrhythmias) following complicated symptomatic infection		0.17%	Jayashree, 2006; Mandell, 1999

Fatal cases following uncomplicated symptomatic infection		1%	Rakhmanova, 1996
Fatal cases following complicated symptomatic infection		25.7%	Rakhmanova, 1996

Table 2. Disability weights and duration

Health outcome (Health state)	Disability Weight (DW) (Haagsma, 2015)		Duration	
	DW	Label	In years	Source
Symptomatic infection (Uncomplicated)<br>(Complicated)	0.051 (0.039-0.06)<br>0.125 (0.104-0.152)	Infectious disease, acute episode, moderate<br>Infectious disease, acute episode, severe	0.003	Kwong 2012
Permanent disability (arrhythmias) following complicated symptomatic infection	0.295 (0.258-0.343)	Cardiac conduction disorders and cardiac dysrhythmias	Remaining life expectancy	

References

Centers for Disease Control and Prevention. Epidemiology and Prevention of Vaccine-Preventable Diseases. Atkinson W, Wolfe S, Hamborsky J, McIntyre L, eds. 11th ed. Washington DC: Public Health Foundation, 2009.

Demirci CS, Abuhammour W. Diphtheria. Available from: http://emedicine.medscape.com/article/963334-clinical#showall Accessed: 1 September 2011.

Hadfield TL, McEvoy P, Polotsky Y, Tzinserling VA, Yakovlev AA. The pathology of diphtheria. J Infect Dis. 2000;181(SUPPL. 1):S116-20

Haagsma JA, Maertens de Noordhout C, Polinder S, Vos T, Havelaar AH, Cassini A, Devleesschauwer B, Kretzschmar ME, Speybroeck N, Salomon JA. Assessing disability weights based on the responses of 30,660 people from four European countries. Population Health Metrics 2015; 13: 10

Jayashree M, Shruthi N, Singhi S. Predictors of outcome in patients with diphtheria receiving intensive care. Indian Pediatr. 2006;43(2):155-60.

Kwong JC, Ratnasingham S, Campitelli MA, Daneman N, Deeks SL, et al. (2012) The Impact of Infection on Population Health: Results of the Ontario Burden of Infectious Diseases Study. PLoS ONE 7(9): e44103. doi:10.1371/journal.pone.0044103

Lodha R, Dash NR, Kapil A, Kabra SK. Diphtheria in urban slums in north India. Lancet, 2000 Jan 15;355(9199):204. PMID: 10675127

Mandell GL, Bennet JE, Dolin R. Mandell, Douglas and Bennett's principles and practice of infectious disease 5th edition. Churchill Livingstone, 1999.

Merck Manual available at http://www.merckmanuals.com/professional/infectious_diseases/gram-positive_bacilli/diphtheria.html?qt=diphtheria&alt=sh#v1005972 (last update May 2013)

Rakhmanova AG, Nosikova E, Tanasijchuk T, Saikku J. Diphtheria Outbreak in St. Petersburg: Clinical Characteristics of 1,860 Adult Patients. Scand J Infect Dis 1996; 28: 37-40.

Solders G, Nennesmo I, Perssont A, Diphtheritic neuropathy, an analysis based on muscle and nerve biopsy and repeated neurophysiological and autonomic function tests. Journal of Neurology, Neurosurgery, and Psychiatry 1989;52:876-880

Vitek CR, Wharton M. Diphtheria in the former soviet union: Reemergence of a pandemic disease. Emerging Infectious Diseases. 1998;4(4):539-50.

WHO (2004). Global Burden of Disease 2004 Update: Disability weights for diseases and conditions. Geneva: WHO. Available online at: http://www.who.int/healthinfo/global_burden_disease/

GBD2004_DisabilityWeights.pdf

Giardiasis

Acute gastroenteritis associated with giardia in humans is in most cases self-limiting within a few weeks (Wolfe, 2000). Vijgen et al. (Vijgen, 2007) assumed for their disease burden estimates a mean duration of 10 days for gastroenteritis cases not requiring medical help or requiring a visit to the doctor. Severe hospitalised gastroenteritis cases were assumed to last for 30 days.

We assumed that the proportion of more severe cases requiring hospitalisation would be 0.265% (360 cases requiring hospitalisation out of an estimated 136 000 incident cases) (Vijgen, 2007). Moreover, the study presents an age-specific risk of hospitalisation which we applied to the 'severe' health state of the symptomatic infection outcome (see Table 3).

The Dutch Association of Parasitology is not aware of fatal cases of giardia (Vijgen, 2007). Additionally, studies by Adak et al. (Adak, 2002) and Levy et al. (Levy, 1998) have not reported fatal cases.

However, a small number of deaths associated with giardiasis were reported to TESSy: nine cases between 2009 and 2013, resulting in 0.014% of notified cases. The CFP is applied to all symptomatic cases and re-distributed according to the age-group observed deaths for giardiasis and cryptosporidiosis notified between 2009 and 2013 from all Member States, with the exception of Denmark, France, Greece, Italy, Liechtenstein, the Netherlands and Portugal, because they do not report (see Table 4). Data from Bulgaria and Poland were also excluded because they only report aggregate data. It is important to note that the CFP will increase in case multipliers adjusting for under-estimation are applied to the incidence inputted in the toolkit and this should be taken into account.

Risk of complications

Apart from Irritable Bowel Syndrome (IBS) as a possible sequela of giardia, no other sequelae could be identified. However, given the fact that few studies expressed a statistical link between IBS and giardia (1–2%) (Nygard, 2006; Hanevik, 2009; Haagsma, 2010), IBS was not included as a possible complication.

Model input summary

Table 1. Transition probabilities used in the outcome tree

Health outcome<br><br>(Health state)	Distribution of health states in health outcome	Transition probability	Source/assumption
Symptomatic infection<br>(Uncomplicated)<br>(Complicated)	99.735%<br>0.265%<br>Age dep. (Table 3)		
Fatal cases following		0.014%	TESSy 2009-2013

symptomatic infection		Age dependent (Table 4)	

Table 2. Disability weights and duration

Health outcome	Disability Weight (DW) (Haagsma, 2015)		Duration	
(Health state)	DW	Label	In years	Source/assumption
Symptomatic infection				Vijgen, 2007
(Moderate)	0.149 (0.12–0.182)	Diarrhoea, moderate	0.027	
(Severe)	0.239 (0.202-0.285)	Diarrhoea, severe	0.082	

Table 3. Age distribution of severe cases

Age class	%
0–4	27
5–9	27
10–14	3
15–64	34
≥65	8

Table 4. Age-group redistribution of CFR (applied only to complicated cases)

Age groups	%
0	12.50
1–4	6.25
5–9	6.25
10–14	0.00
15–19	0.00
20–24	0.00
25–29	0.00
30–34	6.25
35–39	0.00
40–44	0.00
45–49	6.25
50–54	12.50
55–59	6.25
60–64	6.25
65–69	6.25
70–74	6.25
75–79	18.75
80–84	6.25
>85	0.00
All ages	100.00

References

Adak GK, Long SM, O'Brien SJ. Trends in indigenous foodborne disease and deaths, England and Wales: 1992 to 2000. Gut, 2002. 51(6):832-841.

Food Standards Agency. A Report of the Study of Infectious Intestinal Disease in England. Food Standards Agency London, 2000. HMSO.

Haagsma JA, Siersema PD, De Wit NJ, Havelaar AH. Disease burden of post-infectious irritable bowel syndrome in the Netherlands. Epidemiol Infect 2010. 138(11):1650-6.

Haagsma JA, Maertens de Noordhout C, Polinder S, Vos T, Havelaar AH, Cassini A, Devleesschauwer B, Kretzschmar ME, Speybroeck N, Salomon JA. Assessing disability weights based on the responses of 30,660 people from four European countries. Population Health Metrics 2015; 13: 10

Hanevik K, Dizdar V, Langeland N, Hausken T. Development of functional gastrointestinal disorders after Giardia lamblia infection. BMC Gastroenterol, 2009. 9:27.

Lengerich EJ, Addiss DG, Juranek DD. Severe giardiasis in the United States. Clin Infect Dis, 1994.18(5):760-763.

Levy DA, Bens MS, Craun GF, Calderon RL, Herwaldt BL. Surveillance for waterborne-disease outbreaks – United States, 1995–96. MMWR CDC Surveill Summ, 1998. 47(5):1-34.

Nygard K, Schimmer B, Sobstad O, Walde A, Tveit I, Langeland N. A large community outbreak of waterborne giardiasis-delayed detection in a non-endemic urban area. BMC Public Health, 2006. 6:141.

Vijgen SMC, Mangen MJM, Kortbeek LM, Van Duynhoven YTHP, Havelaar AH. Disease burden and related costs of cryptosporidiosis and giardiasis in the Netherlands. 58 pp. Bilthoven. 2007. National Institute of Public Health and the Environment.

Wolfe MS. Giardiasis. Clin Microbiol Rev, 1992. 5(1):93-100.

Gonorrhoea

Gonorrhoea is the second most commonly reported sexually transmitted disease (STD) in the United States of America (Skolnik & Neil, 2008). Neisseria gonorrhoeae is almost exclusively transmitted by sexual contact and perinatally (from mother to child during labour) (Handsfield & Sparling, 2005). The bacteria affect the mucous membranes of the urethra and the cervix. Less frequently, mucous membranes of the rectum, oropharynx and conjunctivae are also involved during infection. N. gonorrhoeae primarily infects columnar and cuboidal epithelium. Gonorrhoeal infections in women may lead to pelvic inflammatory disease (PID) and may be a cause of female infertility. Further complications resulting from infection with N. gonorrhoeae are epididymitis, ophthalmitis, ectopic pregnancy and disseminated gonococcal infection (DGI). Untreated infections mostly resolve spontaneously over time (several weeks or months) but can lead to serious sequelae associated with adverse effects on health. Even though the duration of disease is hard to estimate, mean duration is assumed to be several days for men and less than two weeks for women. The incubation period is short and re-infection is common (Handsfield & Sparling, 2005).

The true number of gonorrhoea cases is largely affected by under-estimation due to high percentages of asymptomatic cases and diagnosed cases not being reported to the surveillance system. It was estimated that the true number of new infections is twice as high as the reported number (CDC, 2002). Brunham and Embree reported that gonorrhoea is posing serious threats in Africa, Latin America, Asia and eastern Europe (Brunham & Embree, 1992). In 2008, WHO estimated that there were around 46.8 million cases of STDs in the European Region, with 3.4 million cases being due to N. gonorrhoeae (WHO, 2012).

About 40–80% of women are asymptomatically infected (De Maio & Zenilman, 1998; Nelson, 2007). For men symptomatic rates of up to 95- 99% were observed for genital infection (De Maio & Zenilman, 1998; Nelson, 2007; Stamm, 2005).

Health outcomes and health states associated with gonococcal infection

Infection with N. gonorrhoeae results in different clinical pictures in women, men and infants. In our study, we only considered disease models which reflect genital infection; pharyngeal and rectal infections are not considered to be the cause of significant short or long-term sequelae and therefore do not contribute to the burden of gonorrhoea.

Infections in men

An uncomplicated infection presents as an acute urethritis, infection in the pharynx or rectum are likely to be asymptomatic. In 2013, 36% of reported gonorrhoea cases were detected at these sites. In most cases (95–99%) the disease has a symptomatic course with typical signs of dysuria and urethral discharge (De Maio & Zenilman, 1998; Nelson, 2007; Stamm, 2005). In a few cases the infection remains asymptomatic and is neither recognised nor diagnosed (Sherrard, 1996). These infections pose a serious problem as they provide a pool of further transmissible infections. In most cases gonococcal urethritis resolves spontaneously over several weeks but may also trigger sequelae (Handsfield & Sparling, 2005).

The most common sequela of gonococcal infections in men is the acute epididymitis (Stamm, 2005; Trojian, 2009). The symptoms associated with epididymitis are oligospermia during the acute phase, swollen epididymis (and/or testicles), and dysuria. The association between epididymitis and future infertility is an ongoing debate in research with no clear evidence (Stamm, 2005). Uncommon complications are penile oedema, penile lymphangitis, periurethral abscess, acute prostatitis, seminal vasculitis and Tyson's or Cowper's gland infections (Handsfield & Sparling, 2005). Due to their rare occurrence they are not considered in the outcome tree.

Infections in women

Uncomplicated infections in women mostly affect the endocervix and N. gonorrhoeae are also recovered from the urethra, rectum or occasionally from the periurethral (Skene's) glands and the ducts of Bartholin's glands. Many women with gonococcal infections only develop minor symptoms or are entirely asymptomatic and thus do not seek medical advice and are consequently not reported to the surveillance system.

A major complication resulting in remarkable disease burden is pelvic inflammatory disease (PID) (Handsfield & Sparling, 2005; De Maio & Zenilman, 1998). Studies report 10–40% of infected women developing PID (Handsfield, 1974; McCormack, 1977; Westrom, 1980; Westrom, 1992). In a cost effectiveness analysis, Bernstein and colleagues estimated a base case scenario of 30% (range 10–40%) of infected women developing PID (Bernstein, 2006). Women with

PID have an increased risk of developing infertility in the future (Handsfield & Sparling, 2005; De Maio & Zenilman, 1998; Westrom, 1980; Westrom, 1992; Ross, 2002). The study of Weström (1992) and colleagues reported a 10% probability of infected women developing tubal infertility. The risk of infertility is linked to number and severity of PID episodes. Ross reported 15–20% and 50–80% of infected women developing tubal infertility after one and three or more PID episodes, respectively. PID itself is also a cause of further (long-term) sequelae such as chronic pelvic pain, ectopic pregnancy and perihepatitis. Pelvic pain occurs in 20% of cases and ectopic pregnancy in 9.1% of PID cases (Handsfield & Sparling, 2005; Westrom, 1980). Infections with N. gonorrhoeae during pregnancy can result in spontaneous abortion, premature labour, early rupture of fetal membranes and perinatal infant mortality (Handsfield & Sparling, 2005). The cost effectiveness study by Bernstein and colleagues estimated transition probabilities from PID to chronic pelvic pain, ectopic pregnancy and tubal factor infertility of 18% (range 15–30), 7.8% (range 7.8–9.1%), and 15% (range 9–18%), respectively (Bernstein, 2006).

Sequelae reported for both sexes

As a result of bacteraemic dissemination, disseminated gonococcal infection (DGI) can occur in 0.5–3% of people infected with N. gonorrhoeae. This may cause infective arthritis and also be the cause of endocarditis and meningitis in very rare cases (Holmes, 2007).

Gonococcal infections in infants

Infants born to infected mothers can suffer from gonococcal conjunctivitis (ophthalmia neonatorum). Gonococcal conjunctivitis affects 30–35% of children born to infected mothers and is a major problem in many developing countries causing blindness (De Maio & Zenilman, 1998; Nelson, 2007). Ophthalmia neonatorum can lead to corneal scars, resulting in low-vision or complete blindness. Effective treatment is available which has led to very low numbers of sequelae resulting from ophthalmia neonatorum in the developed world (Darling, 2010; Schaller & Klauss, 2001). Consequently, we did not consider corneal-scar-related 'low-vision' or 'blindness' in our model.

Infected infants may have a low birth weight; some studies relate low birth weight to gonococcal infections (15% from Gerbase, 2000), however the attribution of this condition to the infection is extremely difficult in a developed country setting. Therefore, we decided to discard this relationship.

Case fatality proportion

Fatal cases resulting from gonococcal infections are extremely rare and mainly result from endocarditis, meningitis and DGI. Estimating the mortality of PID is complicated due to the lack of standardised case definitions, inconsistent reporting practices and unclear aetiology (percentage of fatal cases attributable to gonococcal PID) (De Meaio & Zenilman, 1998).

Outcome tree parameters

Male outcome-tree

The proportion of infections in men who develop symptoms is set at 95–99% (De Maio & Zenilman, 1998; Trojian, 2009, Nelson, 2007). The probability of developing DGI (which is part of the initial symptomatic phase of the disease) is set at 0.5–3% (Holmes, 2007), whereas the probability of developing epididymitis is set to 3% (1–5%) (Bernstein, 2006). Debate is currently ongoing as to whether asymptomatic cases also develop epididymitis, however, due to lack of a proven association, this was not taken into account.

Female outcome-tree

Information on the proportion of symptomatic (20–60%) and asymptomatic (40–80%) gonococcal infections were taken from reviews, clinical text books and a study conducted by Weström (Handsfield & Sparling, 2005; De Maio & Zenilman, 1998; Nelson, 2007; Westrom, 1992). Information on PID as a major sequela were obtained from reviews, clinical text books and a cost effectiveness analysis which provided an estimate that 30% (10–40%) of women were symptomatically infected (Bernstein, 2006). The probabilities of developing an ectopic pregnancy (7.8-9.1%), chronic pelvic pain (18%, range 15–30%) or tubal infertility (15%, range 9–18%) were taken from Bernstein`s cost-effectiveness study (Bernstein, 2006). Case fatality proportions from ectopic pregnancies were estimated at 0.038% (Goldner, 1993). The probability of developing a tubo-ovarian abscess is set at 0.8% (Ness, 2002). However, diagnosis and treatment have significantly improved it was therefore decided not to include a case fatality event for tubo-ovarian abscess.

Congenital outcome-tree

The burden studies on STDs by Gerbase and colleagues and Nelson et al. report 30–35% of cases developing ophthalmia neonatorum (Nelson, 2007; Gerbase, 2000).

Assuming that in EU/EEA Member States all notified cases will have had symptoms, in our model all cases of symptomatic infant gonococcal infections manifest as ophthalmia neonatorum and will represent the only health state included in the model.

Model input summary

Table 1. Transition probabilities and distributions used in the outcome tree

Health outcome	Distribution of health states in health	Transition probability	Source/assumption

(health state)	outcome		
Men			
Symptomatic infection (Urethritis)<br>- Uncomplicated<br>- Complicated	97–99.5%<br>0.5–3%	95–99%	De Maio & Zenilman, 1998; Nelson, 2007; Stamm, 2005<br>Holmes, 2007
Epididymitis from symptomatic		3% (1–5%)	Bernstein, 2006
Women			
Symptomatic infection (Cervicitis)<br>- Uncomplicated<br>- Complicated	97–99.5%<br>0.5–3%	20–60%	Handsfield & Sparling, 2005; De Maio & Zenilman, 1998; Nelson, 2007; Westrom, 1992;<br>Holmes, 2007
Pelvic Inflammatory Disease (PID) from symptomatic and asymptomatic		30% (10–40%)	Bernstein, 2006
Ectopic pregnancy		7.8–9.1%<br>Age dep.<br>See Table 4	Bernstein, 2006<br>Female reproductive age 15-49
Tubo-ovarian abscess		0.8%	Ness, 2002
Chronic pelvic pain syndrome		18% (15–30%)	Bernstein, 2006

Tubal infertility		15% (9–18%)<br>Age dep.<br>See Table 4	Bernstein, 2006<br>Female reproductive age 15-49
Fatal cases due to ectopic pregnancy		0.038%	Goldner, 1993
Congenital			
Symptomatic infection<br>(Ophthalmia neonatorum)		100%	

Table 2. Disability weights and duration

Health outcome<br>(health state)	Disability Weight (DW) (Haagsma, 2015)		Duration	
	DW	Label	In years	Source
Men				
Uncomplicated<br>Complicated	0.051 (0.039-0.06)<br>0.125 (0.104-0.152)	Infectious disease, acute episode, moderate<br>Infectious disease, acute episode, severe	0.02<br>0.02	Trojian, 2009<br>Trojian, 2009
Epididymitis	0.176 (0.143-0.208)	Epididymo-orchitis	0.08	Trojian, 2009
Women				
Uncomplicated<br>Complicated	0.051 (0.039-0.06)<br>0.125 (0.104-0.152)	Infectious disease, acute episode, moderate<br>Infectious disease, acute episode, severe	0.03<br>0.03	Murray, 1996<br>Murray, 1996
Pelvic Inflammatory Disease (PID)	0.123 (0.1-0.15)	Abdominopelvic problem, moderate	0.07	De Maio & Zenilman, 1998
Tubo-ovarian abscess	0.31 (0.262-0.355)	Abdominopelvic problem, severe	0.01	Goharkhay, 2007; Teisala, 1990
Chronic pelvic pain	0.123 (0.1-0.15)	Abdominopelvic problem, moderate	2.8	Sharma, 2011
Ectopic pregnancy	0.31 (0.262-0.355)	Abdominopelvic problem, severe	0.08	Murray, 1996
Tubal infertility	0.007 (0.005-0.01)	Infertility, secondary	See Table 3	Female reproductive age 15–49 years<br>See Table 4

Congenital				
Symptomatic infection (Ophthalmia neonatorum)	0.015 (0.011-0.019)	Conjunctivitis without corneal scar	0.038	American Academy of Pediatrics, 2012. Assuming 2 weeks of treatment

Table 3. Duration of tubal infertility

Age	Duration in years
15–19	32
20–24	27
25–29	22
30–34	17
35–39	12

40–44	7
45–49	2

Table 4. Age group risk (only reproductive age)

Age	%
0–14	0
15–49	100
≥50	0

References

Adelman D, Casale T, Corren J. Manual of Allergy and Immunology. Philadelphia, USA: Lippincott, Williams and Wilkins; 2002.

American Academy of Pediatrics. Chlamydia trachomatis. In: Pickering LK, editor. Red book: 2012. Report of the Committee on Infectious Diseases. Elk Grove Village, IL: American Academy of Pediatrics; 2012:276–81.

American Social Health Association. Sexually Transmitted Diseases in America: How Many Cases and at What Cost? In: Sexually Transmitted Disease in America: How Many Cases and at What Cost? (Ed.). City; 1998:1-27.

Barr J, Danielsson D. Septic gonococcal dermatitis. Br Med J 1971, 1:482-485.

Bernstein KT, Mehta SD, Rompalo AM, Erbelding EJ. Cost-effectiveness of screening strategies for gonorrhea among females in private sector care. Obstet Gynecol 2006, 107:813-821.

Brunham R, Embree J. Sexually transmitted diseases: Current and future dimensions of the problem of the Third World. In: Reproductive Tract Infections Global Impact on Priorities for Women's Reproductive Health. Edited by Germanie A, Holmes K, Piot P, Wasserheit N. New York: Plenum Press; 1992: 35-38

Centers for Disease Control and Prevention. Sexually Transmitted Disease Surveillance, 2002. Atlanta: US Department of Health and Human Services; 2003.

Darling EK, McDonald H. A Meta-analysis of the Efficacy of Ocular Prophylactic Agents Used for the Prevention of Gonococcal and Chlamydial Ophthalmia Neonatorum. Journal of Midwifery & Women's Health 2010, 55:319-327.

DeMaio J, Zenilman J. Gonococcal Infections. In: Bacterial Infections of Humans: Epidemiology and Control. Edited by Evans A, Brachman P. New York: Plenum Medical Book Company; 1998: 285-304

Gerbase A, Stein C, Levison J, Htun Y. Global burden of sexually transmitted diseases (excluding HIV) in the year 2000. In: Global burden of sexually transmitted diseases (excluding HIV) in the year 2000 (Ed.) City: World Health Organization; 2000.

Goharkhay N, Verma U, Maggiorotto F. Comparison of CT- or ultrasound-guided drainage with concomitant intravenous antibiotics vs. intravenous antibiotics alone in the management of tubo-ovarian abscesses. Ultrasound Obstet Gynecol 2007, 29:65-69.

Goldner TE, Lawson HW, Xia Z, Atrash HK. Surveillance for ectopic pregnancy—United States, 1970–1989. MMWR CDC Surveill Summ. 1993;42:73–85.

Haagsma JA, Maertens de Noordhout C, Polinder S, Vos T, Havelaar AH, Cassini A, Devleesschauwer B, Kretzschmar ME, Speybroeck N, Salomon JA. Assessing disability weights based on the responses of 30,660 people from four European countries. Population Health Metrics 2015; 13: 10

Handsfield HH, Lipman TO, Harnisch JP, Tronca E, Holmes KK. Asymptomatic gonorrhea in men. Diagnosis, natural course, prevalence and significance. N Engl J Med 1974, 290:117-123.

Handsfield HH, Sparling RF. Neisseria gonorrhoeae. In Principles and Practice of Infectious Diseases. Volume Volume 2; 6th Edition. Edited by Mandel GL, Bennet JE, Dolin R. Peensylvania: Elsevier Inc.; 2005: 2514-2529

Holmes KK et al. Sexually Transmitted Diseases. 4th Edition 2007. McGraw-Hill Companies: New York. ISBN: 978-0071417488

Marra F, Marra CA, Patrick DM. Cost effectiveness analysis of azithromycin and doxycycline for Chlamydia trachomatis infection in women: A Canadian perspective. Can J Infect Dis 1997, 8:202-208.

McCormack WM, Stumacher RJ, Johnson K, Donner A. Clinical spectrum of gonococcal infection in women. Lancet 1977, 1:1182-1185.

Murray CJL, Lopez AD, World Health Organization, World Bank, Harvard School of Public Health. Global health statistics: a compendium of incidence, prevalence and mortality estimates for over 200 conditions. Geneva: World Health Organization; 1996.

Murray CJL, Lopez AD. The Global Burden of Disease - A comprehensive assessment of mortality and disability from diseases, injuries, and risk factors in 1990 and projected to 2020. Cambridge: Harvard School of Public Health on behalf of the World Health Organization and the World Bank; 1996.

Nelson AL. Gonorrheal Infections. In: Sexually Transmitted Infections - Current Clinical Practice. Edited by Nelson AL, Woodward J, Wysocki S: Humana Press; 2007: 153-182

Ness RB, Soper DE, Holley RL, Peipert J, Randall H, Sweet RL, et al. Effectiveness of inpatient and outpatient treatment strategies for women with pelvic inflammatory disease: results from the Pelvic Inflammatory Disease Evaluation and Clinical Health (PEACH) Randomized Trial. Am J Obstet Gynecol 2002. 186: 929-937.

Newman LM, Moran JS, Workowski KA. Update on the management of gonorrhea in adults in the United States. Clin Infect Dis 2007, 44 Suppl 3:S84-101.

Ross JD. An update on pelvic inflammatory disease. Sex Transm Infect 2002, 78:18-19.

Rowley J, Berkely S (1998). Sexually Transmitted Diseases. In: Health Dimensions of Sex and Reproduction. The Global Burden of Disease and Injuries Series, Volume 3. Harvard University Press, Cambridge.

Schaller UC, Klauss V. Is Crede's prophylaxis for ophthalmia neonatorum still valid? Bull World Health Organ 2001, 79:262-263.

Sharma D, Dahiya K, Duhan N, Bansal R. Diagnostic laparoscopy in chronic pelvic pain. Arch Gynecol Obstet 2011, 283:295-297.

Sharp JT. Editorial: Gonococcal infections and arthritis. Arthritis Rheum 1974, 17:511-512.

Sherrard J, Barlow D. Gonorrhoea in men: clinical and diagnostic aspects. Genitourin Med. 1996;72:422–6.

Skolnik NS, Neil S. Sexually Transmitted Diseases. In: Essential Infectious Disease Topics in Primary Care - Current Clinical Practice. Edited by Albert RH: Humana Press; 2008: 105-166

Spencer SE, Bash MC. Extragenital manifestations of Neisseria gonorrhoeae. Curr Infect Dis Rep 2006, 8:132-138.

Stamm WE, Jones RB, Batteiger BE. Chlamydia trachomatis (Trachoma, prinatal infections, Lymphogranuloma venerum and other genital infections). In Principles and Practice of Infectious Diseases. Volume 2, 6th Edition. Edited by Mandel GL, Bennet JE, Dolin R. Pennsylvania: Elsevier Inc.; 2005: 2239-2255

Teisala K, Heinonen PK, Punnonen R. Transvaginal ultrasound in the diagnosis and treatment of tubo-ovarian abscess. Br J Obstet Gynaecol 1990, 97:178-180.

Trojian TH, Lishnak TS, Heiman D. Epididymitis and orchitis: an overview. Am Fam Physician 2009, 79:583-587.

Westrom L, Joesoef R, Reynolds G, Hagdu A, Thompson SE. Pelvic inflammatory disease and fertility. A cohort study of 1,844 women with laparoscopically verified disease and 657 control women with normal laparoscopic results. Sex Transm Dis 1992, 19:185-192.

Westrom L. Incidence, prevalence, and trends of acute pelvic inflammatory disease and its consequences in industrialized countries. Am J Obstet Gynecol 1980, 138:880-892.

Wise CM, Morris CR, Wasilauskas BL, Salzer WL. Gonococcal arthritis in an era of increasing penicillin resistance. Presentations and outcomes in 41 recent cases (1985-1991). Arch Intern Med 1994, 154:2690-2695.

World Health Organization. Global incidence and prevalence of selected curable sexually transmitted infections - 2008. Geneva: World Health Organization; 2012.

Hepatitis A

Hepatitis A virus (HAV) infections range from asymptomatic health state to fulminant hepatitis (Jeong & Lee, 2010). Hepatitis A symptomatic infections depend strongly on the age: approximately 30% of infected children develop symptoms (Jeong & Lee, 2010; Ciocca, 2000), whereas, according to literature, this is 70–80% for adults (Jeong & Lee, 2010; Ciocca, 2000; Cuthbert, 2001). The manifestation of HAV infection in young children generally includes mild flu-like, but anicteric symptoms (Gingrich, 1983), whereas in adults frequently reported symptoms are jaundice, dark urine, fatigue, loss of appetite, abdominal pain and light-coloured stool lasting for several weeks (Koff, 1992).

Not only severity, also duration is related to the age of the patient. Symptoms in young children last for one to two weeks (Gingrich, 1983). According to Koff, around 80% of adults are ill for up to eight weeks (Koff, 1992). Haagsma et al. assumed that symptomatic HAV cases not requiring medical help would have symptoms for 14 days, and symptomatic HAV cases requiring any kind of medical help would have symptoms for 30 days (Haagsma, 2009). Havelaar et al. assumed that hospitalised HAV cases would have symptoms for up to 0.3 years(Haavelar, 2012). According to the US Centers for Disease Control and Prevention, clinical illness usually does not last longer than two months, although 10–15% of persons have prolonged or relapsing signs of symptoms for up to six months (CDC, 2012).

The case fatality proportions are reported to be 0.1% (Mead, 1999), 1% of hospitalised HAV cases (Arteaga Rodriguez, 2010) and 0.3% (Bauch, 2007; Fiore, 2004).

Fatal cases occur mainly in elderly people (Bauch, 2007; Jacobs, 2004; Jacobs, 2000). In the following table we have summarised the rates of mortality attributable to HAV as used in various cost-effectiveness analyses (Bauch, 2007; Jacobs, 2004; Jacobs, 2000).

Table 1. Deaths among symptomatic patients per 10 000 stratified for age classes

Age classes (in years)		Sources	
	Bauch 2007	Jacobs 2004	Jacobs 2000
	30	-	
5-14	18	-	
15-19	18	-	18 (6-30)
20-29	18	18	18 (6-30)
30-39	21	21	21 (10-32)
40-49	59	36	36 (23-49)

50-59	59	81	81 (70-92)
60-69	272	149	149 (146-152)
70-79	272	283	283 (154-310)
>80	272	283	385 (356-414)

We chose to consider the overall case fatality proportion to be within the range 0.1–0.3% and assumed a different age-group distribution of this risk based on the age-group distribution of fatal cases reported to TESSy between 2009 and 2013 (see Table 4). This table is based on all TESSy notified cases from EU Member States except Bulgaria, Lithuania, Latvia and Poland, because they report only aggregate data, and Liechtenstein which does not report.

Risk of complications

Fulminant hepatitis is a rare complication of hepatitis (Jeong & Lee, 2010). According to Bauch et al. (Bauch, 2007), the probability of fulminant infection in hospitalised HAV cases is 0.011%. Jacobs et al. (Jacobs, 2004) assumed that the probability of liver transplantation would be 0.02% for symptomatic HAV cases in 25 to 29-year olds, increasing slightly with age to 0.08% for symptomatic HAV cases in 70-year olds. According to Jeong and Lee (Jeong & Lee, 2010), a liver transplantation may be necessary, however HAV-related fulminant hepatitis does resolve spontaneously on a more frequent basis than fulminant hepatitis of other aetiologies. Given the low incidence, and the resulting negligible burden, fulminant hepatitis was not considered as a separate health outcome in the current study.

In a current review (Jeong & Lee, 2010), rare atypical clinical manifestations and extra-hepatic manifestations are listed. Atypical clinical manifestations occasionally reported are: relapsing hepatitis, prolonged cholestasis, and complicated cases with acute kidney injury. Rarely reported extra-hepatic manifestations are autoimmune haemolytic anaemia, aplastic anaemia, pure red cell aplasia, pleural or pericardial effusion, acute reactive arthritis, acute pancreatitis, acalculous cholecystitis, mononeuritis, and Guillain-Barré syndrome. None of these manifestations were considered in a recent disease burden study (Havelaar, 2012), nor in cost-effectiveness studies evaluating HAV vaccination programmes (Bauch, 2007; Jacobs, 2000, 2004).

Model input summary

Table 2. Transition probabilities used in the outcome tree

Health outcome (Health state)	Distribution of health states in health outcome	Transition probability	Source/assumption
Fatal cases		0.1–0.3%. Age-dependent (Table 4)	Mead 1999, Bauch 2007, Fiore 2004

Table 3. Disability weights and duration

Health outcome (Health state)	Disability Weight (DW) (Haagsma, 2015)		Duration	
	DW	Label	In years	Source/assumption
Symptomatic infection	0.125 (0.104–0.152)	Infectious disease, acute episode, severe	0–9 years: 0.019–0.038 ≥ 10 years: 0.082 (0.038–0.5). See Table 5.	CDC 2012; Haagsma 2009, age-dependent

Table 4. Age-group redistribution of case fatality proportion (0.1–0.3%)

Age groups	%
0	0.00
1-4	0.00
5-9	0.00
10-14	0.00
15-19	0.00
20-24	10.00
25-29	0.00
30-34	0.00
35-39	0.00

40-44	10.00
45-49	0.00
50-54	10.00
55-59	20.00
60-64	10.00
65-69	0.00
70-74	20.00
75-79	20.00
80-84	0.00
>85	0.00
All ages	100.00

Table 5. Duration of symptomatic disease by age group

Age	%
0-9	0.019–0.038
≥ 10	0.082 (0.038–0.5)

References

Allen CJ, Ferson MJ. Notification of infectious diseases by general practitioners: a quantitative and qualitative study. Med J Aust 2000, 172:325- 328.

Arteaga-Rodriguez A, Carrasco-Garrido P, de Andres AL, de Miguel AG, Santos J, Jimenez-Garcia R. Changes in the epidemiology of hepatitis A in Spain (2005-2008): trends of acute hepatitis A hospitalizations, comorbidities, and costs associated with the hospitalization. Eur J Gastroenterol Hepatol 2010, 22:1284-1289.

Bauch CT, Anonychuk AM, Pham BZ, Gilca V, Duval B, Krahn MD. Cost-utility of universal hepatitis A vaccination in Canada. Vaccine 2007, 25:8536-8548.

Centers for Disease Control and Prevention. Epidemiology and Prevention of Vaccine-Preventable Diseases. Atkinson W, Wolfe S, Hamborsky J, eds. 12th ed. Washington DC: Public Health Foundation, 2012.

Ciocca M. Clinical course and consequences of hepatitis A infection. Vaccine 2000, 18 Suppl 1:S71-74.

Cuthbert JA: Hepatitis A: old and new. Clin Microbiol Rev 2001, 14:38-58.

Fiore AE. Hepatitis A transmitted by food. Clin Infect Dis 2004, 38:705-715.

Gingrich GA, Hadler SC, Elder HA, Ash KO. Serologic investigation of an outbreak of hepatitis A in a rural day-care center. Am J Public Health 1983, 73:1190-1193.

Haagsma JA, Janssen MF, Bonsel GJ. Comparing generic and disease specific health state valuations by a laymen panel. In EuroQol proceedings; Oslo, Norway. 2005

Haagsma JA, van der Zanden BP, Tariq L, van Pelt W, van Duynhoven YTHP, Havelaar AH. Disease burden and costs of selected foodborne pathogens in the Netherlands, 2006. vol. RIVM rapport 330331001. Bilthoven: National Institute of Public Health and Environment; 2009.

Haagsma JA, Maertens de Noordhout C, Polinder S, Vos T, Havelaar AH, Cassini A, Devleesschauwer B, Kretzschmar ME, Speybroeck N, Salomon JA. Assessing disability weights based on the responses of 30,660 people from four European countries. Population Health Metrics 2015; 13: 10

Havelaar AH, Haagsma J, Mangen MJJ, Kemmeren JM, Verhoef L, Vijgen SMC, et al. Disease burden of foodborne pathogens in the Netherlands, 2009. International Journal of Food Microbiology 2012 156:231-238.

Havelaar AH, Melse JM. Quantifying public health risks in the WHO Guidelines for Drinking-Water Quality. Bilthoven, the Netherlands: National Institute for Public Health and the Environment; 2003.

Jacobs RJ, Gibson GA, Meyerhoff AS: Cost-effectiveness of hepatitis A-B vaccine versus hepatitis B vaccine for healthcare and public safety workers in the western United States. Infect Control Hosp Epidemiol 2004, 25:563-569.

Jacobs RJ, Margolis HS, Coleman PJ. The cost-effectiveness of adolescent hepatitis A vaccination in states with the highest disease rates. Arch Pediatr Adolesc Med 2000, 154:763-770.

Jeong SH, Lee HS. Hepatitis A: clinical manifestations and management. Intervirology 2010, 53:15-19.

Koff RS. Clinical manifestations and diagnosis of hepatitis A virus infection. Vaccine 1992, 10 Suppl 1:S15-17.

Matin N, Grant A, Granerod J, Crowcroft N. Hepatitis A surveillance in England--how many cases are not reported and does it really matter? Epidemiol Infect 2006, 134:1299-1302.

Mead PS, Slutsker L, Dietz V, McCaig LF, Bresee JS, Shapiro C, et al. Food-related illness and death in the United States. Emerg Infect Dis 1999, 5:607-625.

Van de Laar MJW, Wijgergangs LM, Rijlaarsdam J, de Wit A. Vaccineren tegen hepatitis A: een verkenning van kosten. Infectieziektebulletin 2000, 11:262-266.

WHO. Global Burden of Disease 2004 Update: Disability weights for diseases and conditions. Geneva: WHO; 2004.

Hepatitis B

Hepatitis B is caused by the hepatitis B virus (HBV) which affects the liver and can cause both acute and chronic infections. Many patients present no symptoms during the initial infection.

The following estimates have been calculated for the proportion of infected individuals who develop symptoms:

- 30–50% of those adults infected develop acute icteric hepatitis (McMahon et al. 1985)

- Over 90 percent of perinatal HBV infections are asymptomatic, while the typical manifestations of acute hepatitis are noted in 5–15 percent of newly-infected young children (1–5 years of age) and in 33–50 percent of older children, adolescents, and adults (Shepard et al. 2006).

We therefore assumed that the range for the symptomatic proportion of new infections was age-dependent (see Table 1). The duration of acute illness has been estimated at six weeks (Kwong, 2012).

Chronicity rate

There is much evidence of age-related variation in the development rate for chronic HBV infection after acute infection. For example:

- The likelihood of developing chronic HBV infection is higher in individuals infected perinatally (90%) or during childhood (20–30%), when the immune system is thought to be immature, compared with immunocompetent subjects infected during adulthood (<1%)' (Fattovich, 2008)

- The overall chronicity rate for HBV has been estimated at 5–10%, although it is higher in those who were infected perinatally (90%) or during childhood (20%) (Yim & Lok, 2005)

- More than 90% of infected infants, 25–50% of children infected between and 5 years, and 6–10% of acutely infected older children and adults develop chronic infection (Shepard et al. 2006)

- About 30% of children aged 1–5 years and 5% of adults develop chronic hepatitis B infection (Pungpadong et al. 2007).

- Nearly all persons infected perinatally and up to 50% of children infected between the ages of 1–5 years develop chronic hepatitis (NIH, 2008)

- 5% of adults with acute infection develop chronic hepatitis B (Wilt et al. 2008)

- 5-10% of adult patients do not clear the virus and either progress to become asymptomatic carriers or develop chronic hepatitis (WHO 2002)

- The chronicity rate is approximately 90% for infants in the first year of life, 30% for children infected between the ages of 1 and 4 years and <5% for healthy adults (Edmunds et al. 1993).

In the model, we adopted the age-dependent chronicity rates reported above by Fattovich et al. presented in the results of a systematic review of the literature (2008).

The duration of the chronic carrier stage varies according to the presence or absence of active viral replication, estimated at 4.5 years in the case of active viral replication and 33.24 years in the case of no active replication (Stouthard, 1997). Information on the proportion of chronic hepatitis cases with active viral replication to those without active replication is not available and we chose to set the duration as uncertain, between 4.5 and 33.24 years.

Risk of complications

Fulminant liver failure

Fulminant liver failure occurs in approximately 0.5 to 1.0% of adults with reported acute hepatitis B but rarely in infants and children (Pappas, 1995; Hoofnagle et al. 1995). In the model we specified a range (0.5–1.0%) for this transition probability for all age groups as we were unable to locate specific values for infants and children. However, we modelled the age-specific probability of the case fatality rate based on the observed rates, hence a zero probability of children dying of acute hepatitis (see Table 5).

The case fatality rate (CFR) among patients who develop fulminant liver failure is approximately 20–33% (Bernua et al. 1986; Wai et al. 2005) and this figure was chosen for our model. There were no recent specific European studies stating the frequency and impact of orthotopic liver transplantation (OLT) (Steinmuller et al. 2002) and new antiviral medications (Eisenbach, 2006).

The duration of fulminant liver failure, estimated based on the time from onset of symptoms to encephalopathy, is one to 56 days (Trey and Davidson 1970).

Compensated cirrhosis (CC)

According to Chu (2000), on average, 2.1% of people with chronic HBV infection develop compensated cirrhosis annually. This does not take into account variations due to other effects such as alcohol consumption, diabetes and obesity (in the BCoDE toolkit the yearly rate refers to an Annual Transition Probability, ATP, as opposed to the Lifetime Transition Probability, LTP). However, it is important to consider that individuals who have a severe acute exacerbation complicated by subacute hepatic failure or who have recurrent episodes of acute exacerbations with bridging hepatic necrosis are more likely to develop cirrhosis (Chu, 2000)

Decompensated cirrhosis (DC)

According to a systematic review undertaken by D'Amico et al. (2006. The review undertaken by Fattovich et al. (2008) estimated an annual probability of 3–4% for Europe which we chose for our model.

The 20–57% case fatality rate for DC was estimated based on the review by D`Amico et al. (20% from the first of two DC stages, characterised by ascites with or without non-bleeding esophageal varices; 57% from the second of two DC stages, characterised by bleeding varices, with or without ascites).

The duration of DC is based on the average waiting time for liver transplants in EU countries which publish their data online (UK and Spain): between 124 and 142 days (NHS, 2014; Matesanz 2009).

Hepatocellular carcinoma (HCC)

The annual rate of developing HCC is 0.1% in asymptomatic HBsAg individuals, and between 0.3 and 1% in patients with chronic hepatitis B, but this rate increases to 2–10% in patients with compensated cirrhosis (Fattovich, 2008; Yim & Lok, 2005; Pungpadong, 2007; Chu, 2000; D'Amico, 2006). Chu and Liaw (2006) and Fattovich (2008) estimated the CC to HCC transition probability to range between 1.5 and 2.2%/year for Europe.

For the model, we adopted Fattovich`s (2008) estimate stemming from an extensive systematic literature review of 0.3% (0.12–0.41) per year to develop HCC from chronic hepatitis B infection and 2.2% (1.71–2.71) per year for the development of HCC from compensated cirrhosis.

In a European setting, Shepherd`s (2006) cost-effectiveness analysis set the annual case fatality rate for HCC to 56%, while Kanwal (2005) set it to 43.3% (20–60). We chose the latter range for our model as it includes Shepherd`s assumption.

Model input summary

Table 1. Transition probabilities and distributions used in the outcome tree

Health outcome (Health state)	Distribution of health states within health outcome	Transition probability	Source/assumption
Symptomatic infection		10–50%<br>See Table 3	Age-dependent<br>McMahon, 1985; Shepard, 2006
Chronic hepatitis		1–90%<br>See Table 4	Age-dependent<br>Fattovich, 2008
Fulminant liver failure		0.5–1%	Pappas, 1995; Hoofnagle et al. 1995
Fatal cases due to liver failure		20-33.3%<br>See Table 5	Bernau et al. 1986 ; Wait et al. 2005<br>Assuming different age-specific probabilities based on observed mortality

Compensated cirrhosis		2.1%/year	Chu, 2000 (ATP)
Decompensated cirrhosis		3-4%/year	Fattovich, 2008 (ATP)
HCC, following<br>- Chronic hepatitis<br>- Compensated cirrhosis		0.3% (0.12–0.41)/year<br>2.2% (1.71–2.71)/year	Fattovich, 2008 (ATP<br>Fattovich, 2008 (ATP
CFR, following:<br>- DC<br>- HCC		20-57%/year<br>43.3% (20-60)/year	D'Amico, 2006 (ATP)<br>Kanwal, 2005 (ATP)

Table 2. Disability weights and duration

Health outcome (Health state)	Disability Weight (DW) (Haagsma, 2015)		Duration	
	DW	Source: ECDC European Disability Weight Project (2014)	In years	Source/assumption
Symptomatic infection	0.051 (0.039–0.06)	Infectious disease, acute episode, moderate	0.115	Kwong 2012
Fulminant liver failure	0.515 (0.459–0.572)	Terminal phase, with medication (for cancers, end-stage kidney/liver disease)	0.003–0.153	Trey, 1970
Chronic hepatitis	0.07 (0.057–0.088)	Generic, uncomplicated disease: worry and daily medication	4.5–33.24	Stouthard, 1997

				Assuming uncertainty between proportion with active replication and without
Compensated cirrhosis	0.07 (0.057–0.088)	Generic uncomplicated disease: worry and daily medication	6-10.4<br><br>See Table 6	Murray, 1996<br><br>Age and gender specific
Decompensated cirrhosis	0.163 (0.136–0.194)	Decompensated cirrhosis of the liver	0.34–039	Assuming average waiting time before liver transplantation in the UK and Spain (NHS and Matesanz 2009)
Hepatocellular carcinoma	0.265 (0.222–0.303)	Cancer, diagnosis and primary therapy	0.72–4.48<br><br>See Table 7	Murray, 1996<br><br>Age and gender specific

Table 3. Hepatitis B infected developing symptoms

Age group	Symptomatic hepatitis B
0	10%
1–4	5–15%
5–80+	30–50%

Table 4. Hepatitis B infected developing chronic hepatitis

Age group	Chronic hepatitis B
0	90%
1–4	20–30%
5–80+	1%

Table 5. CFR age distribution for acute hepatitis observed in Estonia, Germany and the Netherlands 2005–2007

Age groups	CFR
0	0.00

1-4	0.00
5-9	0.10
10-14	0.00
15-19	0.00
20-24	0.14
25-29	0.30
30-34	0.53
35-39	1.27
40-44	1.75
45-49	4.56
50-54	5.81
55-59	5.83
60-64	7.90
65-69	11.86
70-74	11.97
75-79	19.77
80-84	15.67
>85	12.54
All ages	100

Table 6. Duration of compensated cirrhosis

	F	M
0–4	10.4	10.3
5–14	10.4	10.4
15–44	10.2	10
45–59	9.3	8.8
60+	6.5	6

Table 7. Duration of HCC

Age group	Duration (years)	
	F	M
0–14	4.48	4.11
15–44	1.45	2.92
45–59	1.91	2.88
60+	0.72	1.56

References

Alter MJ, Mares A, Hadler SC, Maynard JE. The effect of underreporting on the apparent incidence and epidemiology of acute viral hepatitis. Am J Epidemiol. 1987;125(1):133-9.

Bernuau J, Goudeau A, Poynard T. Multivariate analysis of prognostic factors in fulminant hepatitis B. Hepatology, vol.6, no. 4, pp. 648–651, 1986

Bernuau J, Rueff B, Benhamou JP. Fulminant and subfulminant liver failure: definitions and causes. Seminars in Liver Disease, vol. 6, no. 2, pp. 97–106, 1986

Christensen PB, et al. Outbreak of Hepatitis B among injecting drug users in Denmark. J Clinical Virology 2001;22(1):133-141.

Chu CM. (2000), Natural history of chronic hepatitis B virus infection in adults with emphasis on the occurrence of cirrhosis and hepatocellular carcinoma. Journal of Gastroenterology and Hepatology, 15: E25–E30.

D'Amico G, Garcia-Tsao G, Pagliaro L. Natural history and prognostic indicators of survival in cirrhosis: a systematic review of 118 studies. Journal of Hepatology 2006;44(1):217-31.

Edmunds WJ, Medley GF, Nokes DJ, Hall AJ, Whittle HC. The influence of age on the development of the hepatitis B carrier state. Proceedings Biological Sciences, 1993;253(1337):197-201.

Elhouhari HM, Tamimi TIAR, Carey WD. Hepatitis B virus infection: Understanding its epidemiology, course, and diagnosis. Cleveland Clinical Journal of Medicine 2008;75:881-889.

Eisenbach C, Sauer P, Mehrabi A, Stremmel W, Encke J. Prevention of hepatitis B virus recurrence after liver transplantation. Clin Transplant 2006; 20 Suppl 17: 111–116

Fattovich G, Bortolotti F, Donato F. Natural history of chronic hepatitis B: special emphasis on disease progression and prognostic factors. [Review] [143 refs]. Journal of Hepatology. 2008 20071204 [Epub ahead of print];48(2):335-52.

Garfein RS, Vlahov D, Galai N, et al. Viral infections in short-term injection drug users: the prevalence of the hepatitis C, hepatitis B, human immunodeficiency, and human T-lymphotropic viruses. Am J Public Health 1996;86:655–661.

Greten TF, Papendorf F, Bleck JS, Kirchhoff T, Wohlberedt T, Kubicka S, et al. Survival rate in patients with hepatocellular carcinoma: a retrospective analysis of 389 patients. British Journal of Cancer, 2005 May 23;92(10):1862-8.

Haagsma JA, Maertens de Noordhout C, Polinder S, Vos T, Havelaar AH, Cassini A, Devleesschauwer B, Kretzschmar ME, Speybroeck N, Salomon JA. Assessing disability weights based on the responses of 30,660 people from four European countries. Population Health Metrics 2015; 13: 10

Hoofnagle JH, Carithers RL Jr, Shapiro C, Ascher N. Fulminant hepatic failure: summary of a workshop. Hepatology 1995;21:240-252.

Hoofnagle JH, Doo E, Liang TJ, Fleischer R, Lok ASF. Management of Hepatitis B: Summary of a Clinical Research Workshop. Hepatology 2007;45:1056-1075.

Kanwal F, Gralnek IM, Martin P et al. Treatment alternatives for chronic hepatitis B viral infection: a cost-effectiveness analysis. Annals of Internal Medicine. 2005;142:821-31.

Kim WR, Brown RS, Terrault NA, El-Serag H. Burden of liver disease in the United States: summary of a workshop. Hepatology 2002; 36: 227-242

Kwong JC, Ratnasingham S, Campitelli MA, Daneman N, Deeks SL, et al. (2012) The Impact of Infection on Population Health: Results of the Ontario Burden of Infectious Diseases Study. PLoS ONE 7(9): e44103. doi:10.1371/journal.pone.0044103

Levine OS, Vlahov D, Nelson KE. Epidemiology of hepatitis B virus infections among injecting drug users: seroprevalence, risk factors, and viral interactions. Epidemiol Rev 1994;16:418–36

Levine OS, Vlahov D, Koehler J, et al. Seroepidemiology of hepatitis B virus in a population of injecting drug users. Association with drug injection patterns. Am J Epidemiol 1995;142:331–41.

Liaw YF (2009) Natural history of chronic hepatitis B virus infection and long-term outcome under treatment. Liver international Vol. 29; pp 100- 107.

Lok ASF, McMahon (2007) Chronic Hepatitis B. Hepatology Vol. 45; pp 507 - 539

Matesanzemail R, de la Rosa G. Liver transplantation: The Spanish experience. Digestive and Liver Disease Supplements. Volume 3, Issue 4 , Pages 75-81, December 2009.

McMahon BJ, Alward WL, Hall DB, Heyward WL, Bender TR, Francis DP, et al. Acute hepatitis B virus infection: relation of age to the clinical expression of disease and subsequent development of the carrier state. The Journal of Infectious Diseases, 1985 Apr;151(4):599-603.

McMahon BJ (2005). Epidemiology and natural history of Hepatitis B. Seminars in liver disease. Vol. 25. Supplement 1.

Murray CJL, Lopez AD. World Health Organization, World Bank, Harvard School of Public Health. Global health statistics: a compendium of incidence, prevalence and mortality estimates for over 200 conditions. Geneva: World Health Organization; 1996.

Murray CJL, Lopez AD. The Global Burden of Disease - A comprehensive assessment of mortality and disability from diseases, injuries, and risk factors in 1990 and projected to 2020. Cambridge: Harvard School of Public Health on behalf of the World Health Organization and the World Bank; 1996.

National Health System (NHS), UK. h p://www.nhs.uk/condi̭ons/Liver-transplant/Pages/Introduc̭on.aspx. Website visited on 31 July 2014

National Institutes of Health (2008) NIH Consensus Development Conference Statement on Management of Hepatitis B. NIH Consensus and State-of-the-Science Statements Volume 25, Number 2. October 20–22, 2008

Pungpapong S, Kim WR, Poterucha JJ. Natural history of hepatitis B virus infection: an update for clinicians. Mayo Clin Proc 2007; 82:967–975.

Shepard CW, Simard EP, Finelli L, Fiore AE, Bell BP (2006) Hepatitis B virus infection: Epidemiology and vaccination. Epidemiological Reviews; Vol. 28; pp 112-125

Shepherd J, Jones J, Takeda A et al. Adefovir dipivoxil and pegylated interferon alfa-2a for the treatment of chronic hepatitis B: a systematic review and economic evaluation. [Review] [136 refs]. Health Technology Assessment (Winchester, England). 2006;10(28):iii-iiv.

Stouthard M , Essink-Bot M, Bonsel G, Barendregt J, Kramers P. 1997. Disability weights for diseases in the Netherlands. Rotterdam, Department of Public Health, Erasmus University.

Trey C, Davidson CS. The management of fulminant hepatic failure. In: Popper H, Schaffner F, eds. Progress in liver disease. Volume 3. New York: Grune & Stratton, 1970:282-98

Wai CT, Fontana RJ, Polson J, Hussain M, Shakil AO, Han SH, et al. Clinical outcome and virological characteristics of hepatitis B-related acute liver failure in the United States. J Viral Hepat 2005; 12: 192-198

Wilt TJ, Shamliyan T, Shaukat A, Taylor BC, MacDonald R, Yuan J-M, et al. (2008) . Management of Chronic Hepatitis B. Evidence Report/Technology Assessment No. 174. (Prepared by the Minnesota Evidence-based Practice Center under Contract No. 290-02-0009.) AHRQ Publication No. 09-E002. Rockville, MD. Agency for Healthcare Research and Quality. October 2008.

WHO (2002) Hepatitis B; WHO/CDS/CSR/LYO/2002.2:Hepatitis B

WHO (2001) Introduction of Hepatitis B vaccine into childhood immunization services

Yim HJ, Lok ASF. Natural history of chronic hepatitis B virus infection: what we knew in 1981 and what we know in 2005. Hepatology 2006; 43:pp 173–S181.

Hepatitis C

A total of 20–30% of newly infected individuals develop clinical symptoms of acute hepatitis (e.g. anorexia, abdominal discomfort, nausea, vomiting and jaundice) within 2–24 weeks of exposure (CDC, 2011; Wasmuth, 2010; World Health Organization, 2002). In persons who do develop symptoms of acute hepatitis, the illness lasts between two and 12 weeks (Wasmuth, 2010).

In the model, it was assumed that 20–30% of newly infected individuals develop clinical symptoms of acute hepatitis (CDC, 2011).

Rate of developing chronic hepatitis C

Acute hepatitis C develops into chronic infection in 75.6% (67.3–84.9) of all symptomatic and asymptomatic cases over 20 years old, with the infection resolving in the remaining proportion (Alter & Seef, 1994). The chronicity rate is known to be lower in younger individuals. A recent review of the literature by Alter et al. (2000), has estimated that the rate of spontaneous recovery is 29–45% in those aged under 20 years and this was used for the disease model (chronicity rate: 55–69%).

In the early stages of chronic infection there is a small chance of spontaneous remission. The rate of remission of chronic hepatitis C was set at 0.31 (0.26–0.36)% per year in accordance with the findings of Micallef et al. (2006) (in the Burden of Communicable Diseases in Europe toolkit a yearly rate refers to an Annual Transition Probability, ATP, as opposed to the Lifetime Transition Probability, LTP).

In the absence of spontaneous remission or successful antiviral therapy, chronic infections may progress from mild to moderate hepatitis to liver cirrhosis, with a risk of developing life-threatening sequelae such as decompensated liver disease and hepatocellular carcinoma. Progression to severe liver disease can take 20–40 years. However, progression, which is non-linear, is strongly influenced by cofactors including alcohol intake, HIV or HBV coinfection, gender (male) and an older age at infection (Alberti, 2005; Alter & Seeff, 2000; Freeman, 2003; Lauer & Walker, 2001; Poynard, 2001; Thein, 2008).

Given emerging knowledge of the disease, the most appropriate approach to simulating the progression from chronic infection to cirrhosis would be to specify a model with five health stages, representing the METAVIR fibrosis stages F0–F4, linked by multivariate risk functions. A further possibility could be to represent mild and moderate pre-cirrhotic disease stages. However, for the sake of simplicity and in the context of a burden of disease study in which the objective is to compare a broad spectrum of diseases, a single, chronic hepatitis health outcome was applied.

Risk of complications

Compensated cirrhosis (CC)

The risk of HCV-infected persons developing cirrhosis within 20–30 years is estimated in most studies to be within the range of between 5 and 20%, although some studies give estimates of up to 50% (CDC, 2011; Freeman 2001; Freeman 2003; Lauer & Walker, 2001; Poynard, 1997; Poynard, 2001; Thein, 2008; Wasmuth, 2010). Thein et al. predicted via meta-analysis an average 20-year cirrhosis risk of 16% (95% CI: 14%-19%), and a 30-year risk of 41% (95% CI: 36%–45%), which underlines that the progression to cirrhosis is not a linear process (Thein et al. 2008).

The annual risk of progressing to compensated cirrhosis was calculated based on the transitional probabilities between the five METAVIR stages of fibrosis, as estimated by Thein et al. (2008), using random-effect meta-analysis applied to non-clinical studies only. The point estimate for the risk of developing compensated cirrhosis from chronic hepatitis, calculated as the inverse of the summed durations in the first four METAVIR stages (each duration in turn was estimated as 1/probability of leaving the METAVIR stage), was 1.9% per year. The disability duration was calculated at 36.5 years; this is the average time taken for 50% of those with chronic hepatitis to exit the compartment: $1- \exp(-0.019 * 36.5) = 0.5$.

Decompensated cirrhosis (DC)

HCV-associated cirrhosis leads to liver failure and death in about 20–25% of cirrhotic cases. The annual risk of compensated cirrhosis progressing to the decompensated stage (characterised by ascites, bleeding oesophagal varices, or jaundice) is estimated to be 3.9–7% (D'Amico, 2006; Fattovich, 1997; Grieve, 2006; Poynard, 1997; Wasmuth, 2010). In the model, hepatic decompensation was assumed to occur with an annual risk of 3.9 to 12.9 (Dienstag, 2011).

Without transplantation the prognosis is poor. The five-year survival rate with decompensated liver cirrhosis is roughly 50% (Planas, 2004). One report based on a small study population (n=65) estimated the annual mortality rate at 12.9% (Fattovich et al. 1997), but higher values were reported in the systematic review by D'Amico et al. (2006) (20% 1-year mortality from the first stage of DC; 55% from the second DC stage, which is indicated by bleeding varices with or without ascites). The estimated annual risk of death from DC was set to within a range of 13– 38.5% (Fattovich, 1997; Grieve, 2006; D'Amico, 2006); the upper bound was calculated as the mean of the rates for the two DC stages reported by D'Amico et al (2006).

Duration of DC is based on average waiting time for liver transplant in the UK and in Spain which are represented as an average duration (142 days, NHS and 124 days, Matesanz 2009).

Hepatocellular carcinoma (HCC)

In contrast to hepatitis B, development of primary liver cancer, or hepatocellular carcinoma (HCC), is rare in patients with chronic hepatitis C who do not have cirrhosis (Lauer & Walker, 2001; Spengler, 2010; Wasmuth, 2010; WHO, 2002). Once cirrhosis is established, the risk of hepatocellular carcinoma is estimated to be 1–4% per year (Fattovich et al. 1997; Lauer & Walker, 2001). Studies modelling the natural course of hepatitis C have assumed annual risks of around 1.5% (Grieve et al. 2006; Siebert et al. 2003).

HCC is an outcome that can occur after either the compensated or decompensated cirrhosis stages. The annual risk of developing HCC following either CC or DC was set to 3%, based on the estimate by D'Amico et al. (2006).

Studies modelling the natural course of hepatitis C have assumed annual case fatality rates (CFR) due to liver cancer ranging widely from 43– 86% (Grieve, 2006; Siebert, 2003; Wong, 2000). This variation might be a consequence of stage and treatment-specific survival rates, and other underlying conditions including alcohol consumption, diabetes or obesity, where the higher estimate is used to simulate a situation without early diagnosis and effective treatment. In the model, this CFR is set to 48.9%/year, based on the 1-year survival rate (Kwong, 2012).

Other complications

Fulminant hepatic failure due to acute HCV infection is considered to be very rare (CDC, 2011; Lauer & Walker, 2001; Wasmuth, 2010; World Health Organization, 2002) except in cases of HBV coinfection (Chu, 1999). Fulminant liver failure and death was reported to occur in approximately 0.1% (2/1536) of adults with reported (notified) acute hepatitis C (Bianco, 2003). Due to this condition being extremely rare, no health outcome was specified in the outcome tree.

Model input summary

Table 1. Transition probabilities and distributions used in the outcome tree

Health outcome (Health state)	Distribution of health states in health outcome	Transition probability	Source/assumption
Symptomatic infection	20–30%		CDC, 2011
Chronic hepatitis		> 19yr: 75.6% (67.3–84.9)<br>< 20yr: 55–69%	Alter, 1994; Alter, 2000<br>Age dependent
Remission from chronic hepatitis		0.31 (0.26–0.36)%/year	Micallef, 2006 (ATP)
Compensated cirrhosis		1.9%/year	Modelled from Thein, 2008 (ATP)
Decompensated cirrhosis		3.9–12.9%/year	Dienstag, 2011 (ATP)
HCC, following<br>- Compensated cirrhosis<br>- Decompensated cirrhosis		3.0%/year<br>3.0%/year	D'Amico, 2006 (ATP)<br>D'Amico, 2006 (ATP)
CFR, following:<br>- Decompensated cirrhosis<br>- Hepatocellular carcinoma		13–38.5%/year<br>48.9%/year	Fattovich, 1997; Grieve 2006;<br>D'Amico 2006 (ATP)<br>Kwong, 2012 (ATP)

Table 2. Disability weights and duration

Health outcome (Health state)	Disability Weight (DW) (Haagsma, 2015)		Duration	
	DW	Label	In years	Source
Symptomatic infection	0.051 (0.039–0.06)	Infectious disease, acute episode, moderate	0.038–0.23	CDC, 2011; Wasmuth, 2010; World Health Organization, 2002
Chronic hepatitis	0.07 (0.057–0.088)	Generic uncomplicated disease: worry and daily medication	36.5	Modelled from Thein, 2008
Compensated cirrhosis	0.07 (0.057–0.088)	Generic uncomplicated disease: worry and daily medication	6-10.4<br>See table 3	Murray, 1996<br>Age and gender specific
Decompensated cirrhosis	0.163 (0.136–0.194)	Decompensated cirrhosis of the liver	0.34–039	Assuming average waiting time before liver transplantation in UK and Spain (NHS and Matesanz 2009)
Hepatocellular carcinoma	0.265 (0.222–0.303)	Cancer, diagnosis and primary therapy	0.72–4.48<br>See table 4	Murray, 1996<br>Age and gender specific

Table 3. Duration of compensated cirrhosis

Age group	Duration (years)	
	F	M

0-4	10.4	10.3
5-14	10.4	10.4
15-44	10.2	10
45-59	9.3	8.8
60+	6.5	6

Table 4. Duration of hepatocellular carcinoma

Age group	Duration (years)	
	F	M
0-14	4.48	4.11
15-44	1.45	2.92
45-59	1.91	2.88
60+	0.72	1.56

References

Alberti A, Vario A, Ferrari A, Pistis R. (2005). Review article: chronic hepatitis C--natural history and cofactors. Aliment Pharmacol Ther 22 Suppl 2, 74-78.

Alter MJ, Kruszon-Moran D, Nainan OV, McQuillan GM, Gao F, Moyer LA, et al. The prevalence of hepatitis C virus infection in the United States, 1988 through 1994. Engl J Med. 1999 Aug 19; 341(8):556-62.

Alter HJ, Seeff LB. (2000). Recovery, persistence, and sequelae in hepatitis C virus infection: a perspective on long-term outcome. Semin Liver Dis 20, 17-35.

Bianco E, Stroffolini T, Spada E, Szklo A, Marzolini F, Ragni P, et al. (2003). Case fatality rate of acute viral hepatitis in Italy: 1995-2000. An update. Dig Liver Dis 35, 404-408.

Centers for Disease Control and Prevention (CDC). Hepatitis C: Information for Health Professionals. 2011. Available at: http://www.cdc.gov/hepatitis/HCV/index.htm.

Chu CM, Yeh CT, Liaw YF. Fulminant hepatic failure in acute hepatitis C: increased risk in chronic carriers of hepatitis B virus. Gut 1999;45:613- 617.

Crofts N, Jolley D, Kaldor J, et al. Epidemiology of hepatitis C virus infection among injecting drug users in Australia. J Epidemiol Community Health 1997;1:692–697.

D'Amico G, Garcia-Tsao G, Pagliaro L. Natural history and prognostic indicators of survival in cirrhosis: a systematic review of 118 studies. J Hepatol. 2006;44(1):217-31.

Dienstag JL, Ghany MG, Morgan TR, Di Bisceglie AM, Bonkovsky HL, Kim HY, et al. and the HALT-C Trial Group: A prospective study of the rate of progression in compensated, histologically advanced chronic hepatitis C. Hepatology 2011, 54:396–405

ECDC. Surveillance and prevention of hepatitis B and C in Europe. Stockholm: ECDC 2010.

Fattovich G, Giustina G, Degos F, Tremolada F, Diodati G, Almasio P, et al. (1997). Morbidity and mortality in compensated cirrhosis type C: a retrospective follow-up study of 384 patients. Gastroenterology 112, 463-472.

Freeman AJ, Dore GJ, Law MG, Thorpe M, Von Overbeck J, Lloyd AR et al. Estimating progression to cirrhosis in chronic hepatitis C virus infection. Hepatology 2001;34:809-816.

Freeman AJ, Law MG, Kaldor JM, and Dore GJ. Predicting progression to cirrhosis in chronic hepatitis C virus infection. J Viral Hepat 2003;10:285-293.

Grieve R, Roberts J, Wright M, Sweeting M, DeAngelis D, Rosenberg W, et al. Cost effectiveness of interferon alpha or peginterferon alpha with ribavirin for histologically mild chronic hepatitis C. Gut 2006;55:1332-1338.

Hagan H, Snyder N, Hough E, Yu T, McKeirnan S, Boase J, et al. Case-reporting of acute hepatitis B and C among injection drug users. J Urban Health 2002;79(4):579-85.

Haagsma JA, Maertens de Noordhout C, Polinder S, Vos T, Havelaar AH, Cassini A, Devleesschauwer B, Kretzschmar ME, Speybroeck N, Salomon JA. Assessing disability weights based on the responses of 30,660 people from four European countries. Population Health Metrics 2015; 13: 10

Kwong JC, Ratnasingham S, Campitelli MA, Daneman N, Deeks SL, et al. (2012) The Impact of Infection on Population Health: Results of the Ontario Burden of Infectious Diseases Study. PLoS ONE 7(9): e44103. doi:10.1371/journal.pone.0044103

Lauer GM, Walker BD. Hepatitis C virus infection. N Engl J Med. 2001;345:41-52.

Mann AG, Ramsay ME, Brant LJ, Balogun MA, Costella A, Harris HE. Diagnoses of, and deaths from, severe liver disease due to hepatitis C in England between 2000 and 2005 estimated using multiple data sources. Epidemiol Infect. 2009;137(4):513-8.

Matesanzemail R, de la Rosa G. Liver transplantation: The Spanish experience. Digestive and Liver Disease Supplements. Volume 3, Issue 4 , Pages 75-81, December 2009.

McDonald SA, Hutchinson SJ, Mills PR, et al. Diagnosis of hepatitis C virus infection in Scotland's injecting drug user population. Epidemiol. Infect 2010;138:393–402.

Micallef JM, Kaldor JM, Dore GJ (2006) Spontaneous viral clearance following acute hepatitis C infection: a systematic review of longitudinal studies. J Viral Hepat 13: 34–41.

Murray CJL, Lopez AD, World Health Organization., World Bank., Harvard School of Public Health.: Global health statistics: a compendium of incidence, prevalence and mortality estimates for over 200 conditions. Geneva: World Health Organization; 1996.

Murray CJL, Lopez AD: The Global Burden of Disease - A comprehensive assessment of mortality and disability from diseases, injuries, and risk factors in 1990 and projected to 2020. Cambridge: Harvard School of Public Health on behalf of the World Health Organization and the World Bank; 1996.

National Health System (NHS), UK. Available at: http://www.nhs.uk/conditions/Liver-transplant/Pages/Introduction.aspx(accessed 31 July 2014)

Okuda K, Ohtsuki T, Obata H, Tomimatsu M, Okazaki N, Hasegawa H. et al. (1985). Natural history of hepatocellular carcinoma and prognosis in relation to treatment. Study of 850 patients. Cancer 1985;56:918-928.

Planas R, Balleste B, Alvarez MA, Rivera M, Montoliu S, Galeras JA, et al. Natural history of decompensated hepatitis C virus-related cirrhosis. A study of 200 patients. J Hepatol. 2004;40823-830.

Poynard T, Bedossa P, Opolon P. Natural history of liver fibrosis progression in patients with chronic hepatitis C. The OBSVIRC, METAVIR, CLINIVIR, and DOSVIRC groups. Lancet 1997;349:825-832.

Poynard T, Ratziu V, Charlotte F, Goodman Z, McHutchison J, Albrecht J. Rates and risk factors of liver fibrosis progression in patients with chronic hepatitis c. J Hepatol. 2001;34:730-739.

Siebert U, Sroczynski G, Rossol S, Wasem J, Ravens-Sieberer U, Kurth BM, et al. (2003). Cost effectiveness of peginterferon alpha-2b plus ribavirin versus interferon alpha-2b plus ribavirin for initial treatment of chronic hepatitis C. Gut 52, 425-432.

Spengler U. (2010). Hepatocellular Carcinoma - Diagnosis, Prognosis & Therapy. In Hepatology - A Clinical Textbook. S. Mauss, T. Berg, J. Rockstroh, C. Sarrazin, and H. Wedemeyer, eds. (Düsseldorf, Flying Publisher).

Strauss R, Fülöp G, Pfeifer C. Hepatitis C in Austria 1993–2000: reporting bias distort HCV epidemiology in Austria. Euro Surveill. 2003;8(5):pii=412.

Thein HH, Yi Q, Dore GJ, Krahn MD. (2008). Estimation of stage-specific fibrosis progression rates in chronic hepatitis C virus infection: a meta- analysis and meta-regression. Hepatology 48, 418-431.

Wasmuth, J. (2010). Hepatitis C - Epidemiology, transmission and natural history. In Hepatology - A Clinical Textbook. S. Mauss, T. Berg, J. Rockstroh, C. Sarrazin, and H. Wedemeyer, eds. (Düsseldorf, Flying Publisher).

Wong JB, McQuillan GM, McHutchison JG, Poynard T. (2000). Estimating future hepatitis C morbidity, mortality, and costs in the United States. Am J Public Health 90, 1562-1569.

World Health Organization (2002). Hepatitis C. Available at: http://www.who.int/csr/disease/hepatitis/whocdscsrlyo2003/en/index.html. Accessed: February 2011.

Yousuf M, Nakano Y, Tanaka E, Sodeyama T, Kiyosawa K. Persistence of viremia in patients with type-C chronic hepatitis during long-term follow-up. Scand J Gastroenterol. 1992;27:812-816.

HIV

Acquired Immunodeficiency Syndrome (AIDS) is the most severe outcome of an untreated HIV infection. AIDS presents with severe opportunistic infections, malignancies, neurological complications or other HIV-induced disease conditions (Del Rio & Curran, 2005). After infection with HIV, individuals may remain asymptomatic or develop Acute Retroviral Syndrome (ARS) (Del Rio & Curran, 2005). ARS occurs in 50–66% of all recently infected cases (Sterling & Chaisson, 2005). Due to mild and non-specific flu-like symptoms many people do not seek medical advice, and thus are not diagnosed and treated and proceed to a latent stage where they may remain asymptomatic for years before subsequently developing AIDS.

Within the EU, it is estimated that around 8–45% of all HIV infections are undiagnosed and therefore not reported to the health authorities (ECDC, 2014). The overall duration is difficult to estimate because since introduction of Anti-Retroviral Therapy (ART) HIV is increasingly observed as being a chronic disease and individuals receiving treatment have a similar life expectancy to the rest of the population in Europe (Bhaskaran, 2008). Persistent asymptomatic HIV infection is estimated to be on average 17.2 years for long-term non-progressors (Herida, 2006).

Health outcomes/states associated with HIV-infection

HIV is associated with a heterogeneous set of health outcomes/states. In most cases, certain health outcomes/states are caused by subsequent infections with a secondary or tertiary pathogen. HIV compromises the immune status of an individual and thus increases the risk of further additional pathogens causing severe sequelae.

For our study, we considered that in Europe development of AIDS is significantly limited through ART.

HIV/AIDS is a complicated, multi-faceted and systemic disease and for reasons of feasibility, we developed a simplified model which does not differentiate between the CD4 count stages of the disease at the point of diagnosis, even though this is known to affect mortality (Aghaizu, 2013). Moreover, the current model does not take into account transmitted drug resistance, or the issue of co-morbidity (HIV–HCV or HIV–TB) and the consequent need for a specific therapeutic pathway. We assumed that all diagnosed cases are offered treatment and we applied a certain burden to the disease (e.g. side effects).

HIV infection-related deaths are associated with the development of an acquired immunodeficiency syndrome (AIDS) which, after a prolonged latent period, eventually enables opportunistic infections to develop which are generally the cause of death. Therefore, the nature of AIDS itself consists of comorbidities introducing the issue of attributable cause of death. However, we assumed that the severity of the co-infection and the precipitation to death would not have occurred without the primary HIV infection and deaths were therefore attributed entirely to the initial HIV infection. We also did not include the burden associated with HIV-related malignancies or complications linked to long-term antiretroviral therapy (e.g. cardiovascular disease).

Outcome-tree parameters

The main input is ‘persistent HIV infection’ and this is subdivided according to the speed of progression (Qu, 2008). In general, 5–15% of all patients are rapid progressors (RP) and are at risk of developing AIDS within 2–5 years (Qu, 2008). Another 5–15% are long-term non- progressors (LNP) with, on average 17.2 years duration of development (Qu, 2008; Sterling & Chaisson, 2005). The remainder (70–90%) are typical progressors (TP) with an average duration of 8–10 years (Qu, 2008).

The risk of developing early symptomatic AIDS is set at between 4.5% and 7% (Grinsztejn, 2014: 40 observed cases out of 886 in the group with early ART initiation versus 61 out of 877 in the delayed group).

Terminal AIDS has a duration of one month (Kwong, 2010) and the risk of developing terminal AIDS from early symptomatic AIDS is set at 32.09% as this was the case fatality proportion estimated for AIDS in a recent study (Serraino, 2010).

Model input summary

Table 1. Transition probabilities and distributions used in the outcome tree

Health outcome (Health state)	Distribution of health states in health outcome	Transition probability	Source/assumption
Persistent HIV infection			No cure available
(Rapid progressors)	5–15%		Qu, 2008
(Typical progressors)	70–90%		Qu, 2008
(Long-term non-progressors)	5–15%		Qu, 2008; Herida, 2006
AIDS early symptomatic		4.5–7%	Grinsztejn, 2014
AIDS terminal phase		32.09%	Serraino, 2010
CFR from AIDS		100%	

Table 2. Disability weights and duration

	Disability Weight (DW) (Haagsma, 2015)		Duration

Health outcome (Health state)	DW	Label	In years	Source
Persistent HIV infection	0.108 (0.089-0.132)	HIV/AIDS cases, receiving ARV treatment		
(Rapid progressors)			2-5	Qu, 2008
(Typical progressors)			8-10	Qu, 2008
(Long-term non-progressors)			17.2	Qu, 2008; Herida, 2006
Permanent ARV treatment	0.108 (0.089-0.132)	HIV/AIDS cases, receiving ARV treatment	Remaining life expectancy	Assuming ARV treatment has optimal effectiveness and good compliance
AIDS early symptomatic	0.351 (0.299–0.394)	HIV cases, symptomatic, pre-AIDS	5.36	Herida, 2006
AIDS terminal phase	0.574 (0.518–0.635)	AIDS cases, not receiving ARV treatment	0.08	Kwong, 2010

References

Aghaizu A, Brown AE, Nardone A, Gill ON, Delpech VC & contributors. HIV in the United Kingdom 2013 - Report: data to end 2012. November 2013. Public Health England, London. Available at: https://www.gov.uk/government/uploads/system/uploads/attachment_data/file/326601/HIV_annual_report_2013.pdf

Barre-Sinoussi F, Chermann JC, Rey F, Nugeyre MT, Chamaret S, Gruest J, et al. Isolation of a T-lymphotropic retrovirus from a patient at risk for acquired immune deficiency syndrome (AIDS). Science 1983, 220:868-871.

Bhaskaran K, Hamouda O, Sannes M, Boufassa F, Johnson AM, Lambert PC, et al. for the CASCADE Collaboration. Changes in the Risk of Death After HIV Seroconversion Compared With Mortality in the General Population. JAMA. 2008;300(1):51-59. doi:10.1001/jama.300.1.51.

Cameron DW, Simonsen JN, D'Costa LJ, Ronald AR, Maitha GM, Gakinya MN, et al. Female to male transmission of human immunodeficiency virus type 1: risk factors for seroconversion in men. Lancet 1989, 2:403-407.

Cleghorn FR, Reits Jr. MS, Popovic M, Gallo RC. Human Immunodeficiency Viruses. In Principles and Practice of Infectious Diseases. Volume 2. Edited by Mandell GL, Bennet JE, Dolin R. Pennsylvania: Elsevier Inc.; 2005: 2119-2132

HIV/AIDS. Available at: http://www.who.int/topics/hiv_aids/en/

Del Rio C, Curran JW. Epidemiology and Prevention of Acquired Immunodeficiency Virus Infection. In Principles and Practice of Infectious Diseases. Volume 1. Edited by Mandell GL, Bennet JE, Dolin R. Pennsylvania: Elsevier Inc.; 2005: 1477-1506

European Centre for Disease Prevention and Control. Monitoring implementation of the Dublin Declaration on Partnership to Fight HIV/AIDS in Europe and Central Asia: 2014 progress report, in press.

Gallo RC, Sarin PS, Gelmann EP, Robert-Guroff M, Richardson E, Kalyanaraman VS, et al. Isolation of human T-cell leukemia virus in acquired immune deficiency syndrome (AIDS). Science 1983, 220:865-867.

Grinsztejn B, Hosseinipour M, Ribaudo H, Havlir D, Cohen M, et al. (2014). Effects of Early versus Delayed Initiation of Antiretroviral Therapy on HIV Clinical Outcomes: Results from the HPTN 052. The Lancet Infectious Diseases, Volume 14, Issue 4, April 2014, Pages 281-290

Haagsma JA, Maertens de Noordhout C, Polinder S, Vos T, Havelaar AH, Cassini A, Devleesschauwer B, Kretzschmar ME, Speybroeck N, Salomon JA. Assessing disability weights based on the responses of 30,660 people from four European countries. Population Health Metrics 2015; 13: 10

Herida M, Larsen C, Lot F, Laporte A, Desenclos JC, Hamers FF. Cost-effectiveness of HIV post-exposure prophylaxis in France. AIDS 2006, 20:1753-1761.

Kwong JC, Crowcroft NS, Campitelli MA, Ratanasingham S, Daneman N, Deeks SL, et al. Ontario Burden of Infectious Disease Study. In: Ontario Burden of Infectious Disease Study (ed.), Vol. OAHPP/ECES Report. City: Ontario Agency for Health Protection and Promotion and the Institute for Clinical Evaluative Sciences; 2010.

Laga M, Manoka A, Kivuvu M, Malele B, Tuliza M, Nzila N,et al. Non-ulcerative sexually transmitted diseases as risk factors for HIV-1 transmission in women: results from a cohort study. AIDS 1993, 7:95-102.

Merson MH, Piot P. Global Perspectives on Human Immunodeficiency Virus Infection and Acquired Immunodeficiency Syndrome. In Principles and Practice of Infectious Diseases. Volume 1. Edited by Mandell GL, Bennet JE, Dolin R. Pennsylvania: Elsevier Inc.; 2005: 1456-1506

Qu W, Robinson M, Zhang FJ. Factors influencing the natural history of HIV-1 infection. Chin Med J (Engl) 2008, 121:2613-2621.

Serraino D, Bruzzone S, Zucchetta A, Suligoi B, De Paoli A, Pennazza S, et al. Elevated risks of death for diabetes mellitus and cardiovascular diseases in Italian AIDS cases. AIDS Research and Therapy 2010, 7(11)

Sterling TR, Chaisson RE: General Clinical Manifestations of Human Immunodeficiency Virus Infection (Including the Acute Retroviral Syndrome and Oral, Cutaneous, Renal, Ocular and Cardia Diseases). In Principles and Practice of Infectious Diseases. Volume 1. Edited by Mandell GL, Bennet JE, Dolin R. Pennsylvania: Elsevier Inc.; 2005: 1547-1574

Influenza

In most cases influenza infection in humans is uncomplicated and self-limiting within a few days or weeks, but for some patients the disease is fatal. Approximately one third of influenza infections are mild or asymptomatic, to the extent that infected persons do not even see a doctor (Hayward, 2010; Hayward, 2014). Our model assumes a mean duration of five days (Nicholson, 2003).

Wielders et al. (2010) included four different outcomes and their long-term sequelae following acute illness. These were pneumonia, otitis media, acute respiratory distress syndrome (ARDS) and sepsis. The frequency of other post-infectious complications following an influenza infection is low and these were therefore disregarded in the current study. From a clinical perspective, the acute manifestations of the disease often occur in concomitance as complicated cases

Based on information derived from the General Practice Research Database (GPRD), Meier et al. (2000) estimated the number of patients consulting a doctor with symptoms of influenza-like illness (ILI) who developed complications. The percentages were based on subjects who had at least one clinical diagnosis of influenza or influenza-like-illness (ILI) recorded in the GPRD between 1991 and 1996. In addition to the wide range of national case definitions, estimated consultation rates will also vary among countries due to differences in consultation behaviour, estimation procedure (estimation of incidence, given that many surveillance systems are based on sentinel reporting), vaccination coverage (although vaccination has a limited impact on the number of consultations) and obligatory doctor visits for absence from work or school (Harbers, 2005; Meijer et al., 2006). Therefore, doctor consultations were not considered to be indicative of acute complicated influenza disease.

Given very little specific information on the ratio of complicated/uncomplicated acute disease, no distinction was made between these and the variability was accounted for by including all possible manifestations in the disability weight (mild, moderate and severe): 0.051 (0.007–0.125).

Case fatality ratio

Research has shown that clinicians often attribute influenza-related deaths to a pre-existing underlying condition rather than to influenza (Zucs et al., 2005). Therefore, it is difficult to identify true mortality due to influenza only. Distinguishing further between mortality due to influenza with or without complications such as cardiac problems or pneumonia is even more difficult. Therefore in the current study only one category of death was considered, encompassing all causes which, in the model, occur shortly after infection.

For the Netherlands, it was estimated that during the period 1967–1989 the overall impact of influenza on mortality was greater than registered mortality by a factor of 3.6 (Sprenger et al., 1993). Using this multiplication factor for more recent data may overestimate the number of deaths due to influenza, because in many Member States today vaccination coverage is considerably higher than in the period 1967– 1989. In the study by Sprenger et al. almost half of the non-registered influenza deaths were registered as deaths from heart disease, approximately 25% from lung disease and approximately 30% from other diseases (Sprenger et al., 1993). Recently, time series analysis has also been used to estimate mortality attributable to influenza and other respiratory pathogens (van den Wijngaard et al., 2010).

In about 0.1% of all influenza cases the disease will be fatal (Flu.gov, 2012). This includes both uncomplicated and complicated influenza cases.

Approximately 90% of persons with influenza as cause of death were aged ≥ 65 years (Webster, 2013). Therefore, given that the case fatality proportion for influenza is age-dependent, we modelled the age-specific risk according to the observed mortality data in Estonia, Germany and the Netherlands (see Table 3) (CBS, 2009).

Risk of complications

The most vulnerable populations in terms of complications following influenza are children aged under one 1 year and adults over 65 years, pregnant woman, and people of any age with comorbid illnesses (Rothberg et al., 2008).

The most common complications of influenza are secondary bacterial infections, especially otitis media and pneumonia (van Steenbergen, et al., 2006). It is estimated that 0.65% of influenza cases develop otitis media and 0.36% pneumonia (Meier et al., 2000). Secondary

bacterial pneumonia most often complicate the condition 4–14 days after primary seasonal influenza infection (Rothberg et al., 2008). Neurological complications such as encephalopathy (Reye's syndrome), encephalomyelitis, transverse myelitis, aseptic meningitis, focal neurological disorders, and Guillain-Barré syndrome most often appear in small children (Rothberg et al., 2010). The incidence of neurological complications among <5 years was estimated to be 4 per 100 000 (Newland, 2007).

Wielders et al. (2010) assumed that about 1.23% of all influenza cases develop pneumonia. Earlier, van Lier et al. (2007) assumed that this fraction was 0.36%. In most cases the disease will be self-limiting within a few days, and only in a few cases will it be fatal. According to Murray et al. (1996) long-term outcomes of pneumonia in developed countries are very rare and can be disregarded when estimating disease burden.

Wielders et al. (2010) assumed that 0.65% of influenza cases will develop otitis media as a complication of influenza. Most affected persons will fully recover, but 0.006% of otitis media cases will develop deafness as a life-long disability (Murray, 1996). Given the very low risk, we considered this complication as negligible.

A few cases will develop sepsis during an influenza infection, estimated at 0.0097% of all cases (Wielders, 2010). In some cases the disease will be fatal but again, since there was no detailed information available on the percentage, we assumed that fatal cases would be included in the death estimate related to influenza. Long-term disability was estimated to occur in 82% of patients surviving sepsis (Korosec Jagodic, 2006). However, given the fact that sepsis is caused by bacteria giving rise to super-infections possibly related to other factors, the long-term sequelae of sepsis are not considered to be part of the burden of influenza infections.

Acute respiratory distress syndrome (ARDS) and life-long disability

Following Wielders et al. (2010), we assumed that 0.023% of influenza cases will develop ARDS as a complication of influenza. We assumed that the risk of developing ARDS changes according to age (Manzano, 2005). Wielder's study, however, does not consider cases <15 years and in order to account for these, we also included a study on younger populations (Zimmerman, 2009). We combined the ARDS incidence from the two studies, added them together and estimated the age-group risk of developing ARDS (see Table 4).

In a few cases the disease will be fatal. However, having no detailed information on the specific risk, we assumed that fatal cases would be included in the death estimate related to influenza. Around 30–55% (Hopkins, 1999; Mikkelsen, 2012) of patients surviving ARDS will have developed disabilities related to cognitive impairments at one year follow-up. Therefore, in our model, we estimated that 0.007–0.013% of all symptomatic influenza cases will develop cognitive sequelae assumed to be permanent.

Model input summary

Table 1. Transition probabilities used in the outcome tree

Health outcome (Health state)	Distribution of health states in health outcome	Transition probability	Source/assumption
Permanent disability due to ARDS		0.007–0.013% Age dep. (Table 4)	Wielders, 2010; Manzano, 2005; Hopkins, 1999; Mikkelsen, 2012
Fatal cases		0.10% Age dep. (Table 3)	Flu.gov, 2012; observed cases

Table 2. Disability weights and duration

Health outcome (Health state)	Disability Weight (DW) (Haagsma, 2015)		Duration	
	DW	Label	In years	Source
Symptomatic infection	0.051 (0.007–0.125)	Infectious disease, acute episode, from mild to severe	0.014	Nicholson, 2003
Permanent disability due to ARDS	0.056 (0.044–0.067)	Motor plus cognitive impairments, mild	Remaining life expectancy	Hopkins, 1999; Mikkelsen, 2012

Table 3. Age group distribution of 0.1% risk of fatal cases

Age	%
0	0.58
01-04	0.51
05-09	0.24
10-14	0.27
15-19	0.24

20-24	0.33
25-29	0.31
30-34	0.33
35-39	0.75
40-44	1.15
45-49	1.56
50-54	1.53
55-59	2.21
60-64	3.23
65-69	4.54
70-74	5.22
75-79	11.42
80-84	18.72

85+	46.85

Source: based on all reported fatal influenza cases in Estonia, Germany and the Netherlands for the years 2005–2007.

Table 4. Age group distribution of 0.007–0.013% risk of developing ARDS

Age	%
0-14	7.21
15-29	2.59
30-44	7.66
45-59	12.17
60-74	28.73
≥75	41.63

References

CBS (Statistics Netherlands). Causes of death, 2009. Available at: http://statline.cbs.nl/StatWeb/

U.S. Department of Health & Human Services, Flu.gov (US). Community strategy for pandemic influenza mitigation. 2007 Feb.www.flu.gov/professional/community/mitigation.html [cited 12 March 2010]

Haagsma JA, Maertens de Noordhout C, Polinder S, Vos T, Havelaar AH, Cassini A, Devleesschauwer B, Kretzschmar ME, Speybroeck N, Salomon JA. Assessing disability weights based on the responses of 30,660 people from four European countries. Population Health Metrics 2015; 13: 10

Harbers MM. Influenza: are there international differences? In: Public Health Forecast, National Compass Public Health [Available at http://www.rivm.nl/vtv/object_document/o1736n18081.html]. 2005. Bilthoven: National Institute for Public Health and the Environment (RIVM).

Hayward AC et al. The community burden of influenza and Influenza-Like-Illness in England – Early results from the MRC Flu Watch Study. 2010. Personal communication with Professor Hayward.

Hayward AC, Fragaszy EB, Bermingham A, Wang L, Copas A, Edmunds WJ, et al. Comparative community burden and severity of seasonal and pandemic influenza: Results of the Flu Watch cohort study. Lancet Respir. Med. 2014, 2014, doi:10.1016/S2213-2600(14)70034-70037.

Hopkins RO, Weaver LK, Pope D, Orme JF Jr., Bigler ED, Larson-Lohr V. Neuropsychological sequelae and impaired health status in survivors of severe acute respiratory distress syndrome. American Journal of Respiratory and Critical Care Medicine. 1999;160(1):50–56.

Korosec Jagodic H, Jagodic K, Podbregar M. Long-term outcome and quality of life of patients treated in surgical intensive care: a comparison between sepsis and trauma. Crit Care 2006;10: R134.

Manzano F, Yuste E, Colmenero M, Garcia-Horcajadas A, Rivera R, Fernandez-Mondejar E. Granada Respiratory Failure Study Group: Incidence of acute respiratory distress syndrome and its relation to age. J Crit Care. 2005;20:274–80.

Meier CR, Napalkov PN, Wegmuller Y, Jefferson T, Jick H. Population-based study on incidence, risk factors, clinical complications and drug utilisation associated with influenza in the United Kingdom. Eur J Clin Microbiol Infect Dis 2000. 19 (11): 834-42.

Meijer A, Paget WJ, Meerhoff TJ, Brown C, Meuwissen LE, Van Der Velden J. Epidemiological and virological assessment of influenza activity in Europe, during the 2004-2005 winter. Euro Surveill 2006;11(5): 111-8.

Mikkelsen ME, Christie JD, Lanken PN, Biester RC, Thompson BT, Bellamy SL, et al. The adult respiratory distress syndrome cognitive outcomes study: long-term neuropsychological function in survivors of acute lung injury. Am J Respir Crit Care Med. 2012;17:1307–1315. doi: 10.1164/rccm.201111-2025OC.

Murray CJL, Lopez AD (eds.) (1996). Global health statistics: a compendium of incidence, prevalence and mortality estimates for over 200 conditions (Global burden of disease and injuries series; II). Cambridge: Harvard School of Public Health on behalf of the World Health Organization and the World Bank.

Neurologic Complications in Children Hospitalized with Influenza: Characteristics, Incidence, and Risk Factors. Newland JG, Laurich VM, Rosenquist AW, Heydon K, Licht DJ, Keren R et al. The Journal of Pediatrics, 2007; Vol. 150, 3,306–310

Nicholson KG, Wood JM, Zambon M. Influenza. Lancet 2003; 362:1733-45.

Rothberg MB, Haessler SD, Brown RB (2008). Complications of viral influenza. Am J Med 121(4): 258-64.

Rothberg MB, Haessler SD (2010). Complications of seasonal and pandemic influenza. Crit Care Med 38(4 Suppl): e91-7.

Sprenger MJ, Mulder PG, Beyer WE, Van Strik R, Masurel N (1993). Impact of influenza on mortality in relation to age and underlying disease, 1967-1989. Int J Epidemiol 22(2): 334-40.

van den Wijngaard CC, van Asten L, Meijer A, van Pelt W, Nagelkerke NJ, Donker GA, et al (2010). Detection of excess influenza severity: associating respiratory hospitalization and mortality data with reports of influenza-like illness by primary care physicians. Am J Public Health 100(11): 2248-54.

van Lier EA, Havelaar AH (2007). Disease burden of infectious diseases in Europe: a pilot study. Bilthoven: National Institute for Public Health and the Environment (RIVM) (RIVM report 215011001).

van Steenbergen J, Timen EA (eds) (2006). Guidelines Infectious Disease Control Edition 2006. Bilthoven: LCI, Coordinator Infectious Disease Control for the Netherlands.

Webster, Monto, Bracciale, Lamb. Textbook of Influenza. 2nd Edition, 2013. Wiley Blackwell Ed.

Wielders CCH, van Lier EA, van 't Klooster TM, van Gageldonk-Lafeber AB, van den Wijngaard CC, Haagsma JA, et al. The burden of 2009 pandemic influenza A(H1N1) in the Netherlands. Eur J Public Health 2010. http://dx.doi.org/10.1093/eurpub/ckq187

Zimmerman JJ, Akhtar SR, Caldwell E, Rubenfeld GD. Incidence and outcomes of pediatric acute lung injury. Pediatrics. 2009;124:87–95.

Zucs P, Buchholz U, Haas W, Uphoff H. Influenza associated excess mortality in Germany, 1985-2001. Emerg Themes Epidemiol 2005.2:6.

Invasive haemophilus influenza disease

The major disease burden of invasive H. influenzae infection occurs in children under five years (Fogarty, 1995). The most harmful complication is bacteraemia, which is accompanied by a focal infection such as meningitis, pneumonia, or cellulitis in 30–50% of cases (Devarajan, 2009).

Risk of complications

Meningitis is the principal clinical presentation of invasive disease, but bone and joint infections, pneumonia, epiglottitis, cellulitis and septicaemia can also occur. Skin and soft tissue infections may occur in around 6% of patients, followed by a limited number of sequelae (Otero Reigada, 2005). Only the invasive forms are considered as health states in the model.

To estimate the risk of meningitis we used the surveillance data reported in the ECDC Invasive Disease Surveillance report on clinical presentations of the acute symptomatic disease (ECDC, 2013a; ECDC, 2013b). Reported data indicates that meningitis and septicaemia occur together in 0–1% of cases, whereas meningitis alone occurs in 15–18% (15% in 2010, 18% in 2011) of cases, resulting in an overall risk of 15– 18% of developing meningitis. The risk of developing meningitis during the acute phase of the disease is age-specific. Age and gender-specific data were extracted from ECDC's TESSy database on the meningitis complications of IHID for 2010 and 2011 (see Table 4). The risk of developing the long-term sequelae is age and gender-specific.

Long-term sequelae

Bacterial meningitis may cause long-term sequelae and permanent disabilities. To investigate this we extracted the risk of developing these complications after meningitis episodes from Edmond et al. (Edmond, 2010).

Meningitis accounts for various long-term sequelae (each of which is multiplied by the risk of developing meningitis during the acute phase of the disease: 15–18%): cognitive difficulties (0.17–0.20%), seizure disorders (0.23–0.27%), hearing loss (0.48–0.58%), motor deficit (0.33– 0.40%), visual disturbance (0.08–0.09%), behavioural problems (0.32–0.38%), clinical impairments (0.18–0.22%) and multiple impairments (0.39–0.47%) (Edmond, 2010).

Case fatality proportion

The parameters for the case fatality proportion were based on data for EU/EEA countries in 2011, see Table 3 (ECDC, 2013).

Model input summary

Table 1. Transition probabilities used in the outcome tree

Health outcome (Health state)	Distribution of health states in health outcome	Transition probability	Source/assumption
Hearing loss		0.48-0.58%	Edmond, 2010

Cognitive difficulties		0.17-0.20%	Edmond, 2010
Seizure disorder		0.23-0.27%	Edmond, 2010
Motor deficit		0.33-0.40%	Edmond, 2010
Visual disturbance		0.08-0.09%	Edmond, 2010
Behavioural problems		0.32-0.38%	Edmond, 2010
Clinical impairments		0.18-0.22%	Edmond, 2010
Multiple impairments		0.39-0.47%	Edmond, 2010
Fatal cases due to symptomatic infection		See Table 3 (5.4-19.5%)	ECDC, 2013

Table 2. Disability weights and duration

Health outcome (Health state)	Disability Weight (DW) (Haagsma, 2015)		Duration	
	DW	Label	In years	Source
Symptomatic infection	0.655 (0.579-0.727)	Intensive care unit admission	0.019	Tunkel, 2004 Assuming the duration of antimicrobial therapy

Permanent disability following meningitis			Remaining life expectancy	
1. Hearing loss	0.008-0.103	From lowest to highest hearing loss related DWs		
2. Cognitive difficulties	0.044-0.188	From lowest to highest intellectual disability related DWs		
3. Seizure disorder	0.07 (0.057-0.088)	Generic uncomplicated disease: worry and daily medication		
4. Motor deficit	0.011-0.421	From lowest to highest motor impairment related DWs		
5. Visual disturbance	0.004-0.171	From lowest to highest vision impairment related DWs		
6. Behavioural problems	0.088 (0.07-0.108)	Subacute sclerosing panencephalitis – phase 1 (assuming best fitting health state description)		
7. Clinical impairments	0.004-0.421	From lowest to highest DW included in this model		
8. Multiple impairments	0.004-0.421	From lowest to highest DW included in this model		

Table 3. CFR following symptomatic infection

Age	CFR
0	19.5%
1-4	6.5%
5-14	5.7%
15-64	5.4%
≥65	15%

Table 4. Age specific distribution per gender of the 15-18% risk of developing meningitis manifestation during the symptomatic infection (TESSy 2010-2011)

Age group		%

	F	M
0	15.69	17.12
01-04	15.69	18.49
05-09	2.61	5.48
10-14	1.31	2.74
15-19	1.31	2.74
20-24	2.61	4.11
25-29	0.00	0.00
30-34	3.27	1.37
35-39	1.96	4.79
40-44	3.92	7.53
45-49	3.92	8.22
50-54	7.19	2.74
55-59	7.19	2.74
60-64	3.27	4.79
65-69	10.46	3.42
70-74	11.11	5.48
75-79	5.23	4.11
80-84	2.61	1.37
85+	0.65	2.74
Total	100	100

References

Buysse CM, Oranje AP, Zuidema E, Hazelzet JA, Hop WC, Diepstraten AF, et al. Long-term skin scarring and orthopaedic sequelae in survivors of meningococcal septic shock. Arch Dis Child. 2009 May;94(5):381-6.

Devarajan VR. Haemophilus Influenzae Infections. 2009. http://emedicine.medscape.com/article/218271-overview

Edmond K, Clark A, Korczak VS, Sanderson C, Griffiths UK, Rudan I. Global and regional risk of disabling sequelae from bacterial meningitis: a systematic review and meta-analysis. Lancet Infect Dis. 2010 May;10(5):317-28.

Eidelman LA, Putterman D, Putterman C, Sprung CL. The Spectrum of Septic Encephalopathy: Definitions, Etiologies, and Mortalities. JAMA. 1996;275(6):470-473. doi:10.1001/jama.1996.03530300054040.

European Centre for Disease Prevention and Control. Surveillance of invasive bacterial diseases in Europe, 2010. Stockholm: ECDC; 2013.

European Centre for Disease Prevention and Control. Surveillance of invasive bacterial diseases in Europe, 2011. Stockholm: ECDC; 2013.

Fogarty J, Moloney AC and Newell JB. The epidemiology of Haemophilus influenzae type b disease in the Republic of Ireland. Epidemiol. Infect. (1995). 114. 451-463.

Haagsma JA, Maertens de Noordhout C, Polinder S, Vos T, Havelaar AH, Cassini A, Devleesschauwer B, Kretzschmar ME, Speybroeck N, Salomon JA. Assessing disability weights based on the responses of 30,660 people from four European countries. Population Health Metrics 2015; 13: 10

Jit M. The risk of sequelae due to pneumococcal meningitis in high-income countries: a systematic review and meta-analysis. J Infect 2010;61(2):114-24.

Otero Reigada MC, Piqueras Arenas AI, Ferrer Lorente B, Pérez-Tamarit D, Asensi Botet F, Santos Durantez M. Skin and soft tissue infections caused by Haemophilus influenzae type b over a 30 year period. An Pediatr (Barc). 2005 Jul;63(1):29-33.

Tunkel AR, Hartman BJ, Kaplan SL, Kaufman BA, Roos KL, Scheld WM, et al. Practice guidelines for the management of bacterial meningitis. Clin Infect Dis 2004, 39(9):1267–1284.

Ziaja M. Septic encephalopathy. Curr Neurol Neurosci Rep. 2013;13:383

Invasive meningococcal disease (IMD)

As many as 10% of adolescents and adults are asymptomatic transient carriers of N. meningitidis, most strains of which are not pathogenic. In more than 99% of the cases the infection is asymptomatic, but about 1% of the those infected develop acute illness (CDC, 2009). Invasive disease usually requires a seven-day course of antibiotic therapy (Brigham & Sandora, 2009; Tunkel 2004), but may also result in lifelong major sequelae.

Risk of complications

Meningitis is the most common manifestation of invasive disease, and may occur in 47.3% of all patients suffering from N. meningitidis symptomatic infection and in 52.2% of the patients who develop bacteraemia. It always follows hematogenous dissemination, which occurs in 91% of all patients suffering from symptomatic infection. Sepsis occurs in 5–20% of patients with invasive disease (CDC, 2009). Complications are also possible with non-invasive disease; pneumonia occurs in 6% of symptomatic infections, otitis media in 1% of cases and epiglottitis, which is rare, in 0.3% of all manifestations (CDC, 2009).

We decided to use surveillance data reported to TESSy on clinical presentations of the acute symptomatic disease to estimate the risk of meningitis (ECDC, 2013). Reported data indicates that meningitis and septicaemia together occur in 17–18% of cases, whereas meningitis alone occurs in 43–45% of cases, resulting in an overall risk of 60–63% of developing meningitis. The risk of developing meningitis during the acute phase of the disease is age-specific. Age-specific data were extracted for each gender from ECDC's TESSy database on the meningitis complications of IMD for 2010 and 2011 (see Table 4). The risk of developing long-term sequelae is age and gender-specific.

Long term sequelae

Bacterial meningitis may cause long-term sequelae and permanent disabilities. The risk of developing these complications after meningitis episodes was extracted from Edmond et al. (Edmond, 2010).

Meningitis accounts for various long-term sequelae (each of which is multiplied by the risk of developing meningitis during the acute phase of the disease: 60–63%): cognitive difficulties (0.96–1.01%), seizure disorders (0.3–0.35%), hearing loss (1.56–1.64%), motor deficit (0.6– 0.63%), visual disturbance (0.9–0.95%), behavioural problems (0.36–0.38%), clinical impairments (0.12–0.13%) and multiple impairments (0.78-0.82%) (Edmond, 2010).

Case fatality proportion

The parameters for the case fatality ratio were based on data for EU/EEA countries in 2011, see Table 3 (ECDC, 2013).

Model input summary

Table 1. Transition probabilities used in the outcome tree

Health outcome (Health state)		Distribution of health states in health outcome		Transition probability			Source/assumption

Hearing loss		1.56–1.64%	Edmond, 2010
Cognitive difficulties		0.96–1.01%	Edmond, 2010
Seizure disorder		0.3–0.35%	Edmond, 2010
Motor deficit		0.6–0.63%	Edmond, 2010
Visual disturbance		0.9–0.95%	Edmond, 2010
Behavioural problems		0.36–0.38%	Edmond, 2010
Clinical impairments		0.12–0.13%	Edmond, 2010
Multiple impairments		0.78–0.82%	Edmond, 2010
Fatal cases due to symptomatic infection		See Table 3 (6.9-17.1%)	ECDC, 2013

Table 2. Disability weights and duration

Health outcome (Health state)	Disability Weight (DW) (Haagsma, 2015)		Duration	
	DW	Label	In years	Source
Symptomatic infection	0.655 (0.579-0.727)	Intensive care unit admission	0.019	

				Tunkel, 2004 Assuming the duration of antimicrobial therapy
Permanent disability following meningitis:			Remaining life expectancy	
1. Hearing loss	0.008-0.103	From lowest to highest hearing loss related DWs		
2. Cognitive difficulties	0.044-0.188	From lowest to highest intellectual disability related DWs		
3. Seizure disorder	0.07 (0.057-0.088)	Generic uncomplicated disease: worry and daily medication		
4. Motor deficit	0.011-0.421	From lowest to highest motor impairment related DWs		
5. Visual disturbance	0.004-0.171	From lowest to highest vision impairment related DWs		
6. Behavioural problems	0.088 (0.07-0.108)	Subacute sclerosing panencephalitis – phase 1 (assuming best fitting health state description)		
7. Clinical impairments	0.004-0.421	From lowest to highest DW included in this model		
8. Multiple impairments	0.004-0.421	From lowest to highest DW included in this model		

Table 3. CFP following symptomatic infection

Age	CFR
0	7.8%

1-4	6.9%
5-14	5.6%
15-24	9.5%
25-49	8.9%
50-64	7.6%
≥65	17.1%

Table 4. Age specific distribution per gender of the 60-63% risk of developing meningitis manifestation during the symptomatic infection (TESSy 2010-2011)

Age group	%	
	F	M
0	16.22	16.64
01-04	18.19	23.79
05-09	7.13	8.65
10-14	5.90	4.46
15-19	14.53	15.97
20-24	7.21	8.06
25-29	3.93	3.62
30-34	2.62	3.04

35-39	2.05	1.69
40-44	2.54	1.84
45-49	2.81	2.01
50-54	3.47	2.43
55-59	3.28	2.43
60-64	1.97	1.42
65-69	1.88	1.42
70-74	1.97	0.76
75-79	2.05	1.35
80-84	1.31	0.34
85+	0.93	0.08
Total	100	100

References

Brigham KS, Sandora TJ. Neisseria meningitidis: epidemiology, treatment and prevention in adolescents. Curr Opin Pediatr. 2009 Aug;21(4):437-43.

Buysse CM, Oranje AP, Zuidema E, Hazelzet JA, Hop WC, Diepstraten AF, et al. Long-term skin scarring and orthopaedic sequelae in survivors of meningococcal septic shock. Arch Dis Child. 2009 May;94(5):381-6.

Centers for Disease Control and Prevention. Epidemiology and Prevention of Vaccine-Preventable Diseases. Atkinson W, Wolfe S, Hamborsky J, McIntyre L, eds. 11th ed. Washington DC: Public Health Foundation, 2009.

Edmond K, Clark A, Korczak VS, Sanderson C, Griffiths UK, Rudan I. Global and regional risk of disabling sequelae from bacterial meningitis: a systematic review and meta-analysis. Lancet Infect Dis. 2010 May;10(5):317-28.

Eidelman LA, Putterman D, Putterman C, Sprung CL. The Spectrum of Septic Encephalopathy: Definitions, Etiologies, and Mortalities. JAMA. 1996;275(6):470-473. doi:10.1001/jama.1996.03530300054040.

European Centre for Disease Prevention and Control. Surveillance of invasive bacterial diseases in Europe, 2010. Stockholm: ECDC; 2013.

European Centre for Disease Prevention and Control. Surveillance of invasive bacterial diseases in Europe, 2011. Stockholm: ECDC; 2013.

Haagsma JA, Maertens de Noordhout C, Polinder S, Vos T, Havelaar AH, Cassini A, Devleesschauwer B, Kretzschmar ME, Speybroeck N, Salomon JA. Assessing disability weights based on the responses of 30,660 people from four European countries. Population Health Metrics 2015; 13: 10

Jit M. The risk of sequelae due to pneumococcal meningitis in high-income countries: a systematic review and meta-analysis. J Infect 2010;61(2):114-24.

Tunkel AR, Hartman BJ, Kaplan SL, Kaufman BA, Roos KL, Scheld WM, et al. Practice guidelines for the management of bacterial meningitis. Clin Infect Dis 2004, 39(9):1267–1284.

Ziaja M. Septic encephalopathy. Curr Neurol Neurosci Rep. 2013;13:383.

Invasive pneumococcal disease

Despite the large number of serogroups and serotypes known, most cases of invasive pneumococcal disease (IPD) on a global scale are attributed to the 1, 3, 4, 6, 7, 9, 14, 18, 23 (Jefferson, 2006) and 19a serogroups.

Risk of complications

Invasive pneumococcal infection can manifest as meningitis, bacteraemic pneumonia, bacteraemia without a focus, and bacteraemia with a focus other than the lungs or meninges (e.g. endocarditis, osteomyelitis, and arthritis, although rare). Complications, such as pneumonia or otitis media, are also possible with non-invasive forms of infection but are not considered in this study.

Most observed complications of invasive bacterial diseases, including IPD, are related to the meningitis event. The risk of meningitis was estimated using surveillance data reported to TESSy on clinical presentations of the acute symptomatic disease (ECDC, 2013) and it was found that 10% of IPD cases are reported to manifest meningitis. The risk of developing meningitis during the acute phase of the disease is age- specific. Age and gender-specific data were extracted from ECDC's TESSy database on the risk of developing meningitis for IPD cases from 2010 to 2011 (see Table 4). The risk of developing long-term sequelae is age and gender-specific.

Long-term sequelae

Bacterial meningitis may cause long-term sequelae and permanent disabilities. In order to account for these, information was extracted on the risk of developing permanent sequelae from Edmond et al. (Edmond, 2010).

Meningitis can result in various long-term sequelae: cognitive difficulties (4.2%), seizure disorders (2.5%), hearing loss (7.5%), motor deficit (5.8%), visual disturbance (1.1%), behavioural problems (4.6%) multiple (5.7%) and clinical impairments (3.3%) (Edmond, 2010). Therefore, we assumed that 10% of all IPD patients would be at risk of developing long-term sequelae.

Case fatality proportion

The case fatality proportion for invasive pneumococcal disease has been estimated at 18% in a population-based study of 19 000 people (Harboe, 2009); however, important differences were observed between age groups, with a lower (3%) mortality rate observed in children <5 years. The overall lethality rate due to bacteraemia is about 10–20% (CDC, 2009; Rudan, 2009; Lin, 2010; Saldías, 2009) and may be as high as 60% among elderly patients (CDC, 2009).

Overall mortality due to endocarditis is 50%, but it can reach 60–65% in children (Elward, 1990). The case-fatality proportion for pneumococcal meningitis is about 30%, but may be as high as 80% in elderly persons (CDC, 2009; Burckhardt et al. 2010). The parameters for the case fatality proportion were based on data for EU/EEA countries in 2011, see Table 3 (ECDC, 2013).

Model input summary

Table 1. Transition probabilities used in the outcome tree

Health outcome (Health state)	Distribution of health states in health outcome	Transition probability	Source/assumption

Hearing loss		0.75%	Edmond, 2010
Cognitive difficulties		0.42%	Edmond, 2010
Seizure disorder		0.25%	Edmond, 2010
Motor deficit		0.58%	Edmond, 2010
Visual disturbance		0.11%	Edmond, 2010
Behavioural problems		0.46%	Edmond, 2010
Clinical impairments		0.33%	Edmond, 2010
Multiple impairments		0.57%	Edmond, 2010
Fatal cases due to symptomatic infection		See Table 3 (3-24%)	Harboe, 2009

Table 2. Disability weights and duration

Health outcome (Health state)	Disability Weight (DW) (Haagsma, 2015)	Duration

	DW	Label	In years	Source
Symptomatic infection	0.655 (0.579-0.727)	Intensive care unit admission	0.027-0.038	Tunkel, 2004<br>Assuming the duration of antimicrobial therapy
Permanent disability following meningitis			Remaining life expectancy	
1. Hearing loss	0.008-0.103	From lowest to highest hearing loss related DWs		
2. Cognitive difficulties	0.044-0.188	From lowest to highest intellectual disability related DWs		
3. Seizure disorder	0.07 (0.057-0.088)	Generic uncomplicated disease: worry and daily medication		
4. Motor deficit	0.011-0.421	From lowest to highest motor impairment related DWs		
5. Visual disturbance	0.004-0.171	From lowest to highest vision impairment related DWs		
6. Behavioural problems	0.088 (0.07-0.108)	Subacute sclerosing panencephalitis – phase 1 (assuming best fitting health state description)		
7. Clinical impairments	0.004-0.421	From lowest to highest DW included in this model		
8. Multiple impairments	0.004-0.421	From lowest to highest DW included in this model		

Table 3. CFP following symptomatic infection

Age	CFR
0	5.1%
1-4	3%
5-14	7.1%
15-64	8%

≥65	14.3%

Table 4. Age specific distribution per gender of the 60-63% risk of developing meningitis manifestation during the symptomatic infection (TESSy 2010-2011)

Age group	%	
	F	M
0	10.37	11.45
01-04	8.13	8.52
05-09	2.70	3.56
10-14	1.54	2.54
15-19	0.39	1.57
20-24	1.29	1.22
25-29	1.02	2.23
30-34	2.45	3.56
35-39	3.29	5.68
40-44	3.74	5.58
45-49	5.47	6.90
50-54	6.70	7.31
55-59	9.21	7.76
60-64	11.28	9.02

65-69	9.78	7.04
70-74	7.60	6.24
75-79	6.51	4.91
80-84	5.15	2.98
85+	3.36	1.93
Total	100	100

References

Burckhardt I, Burckhardt F, Van Der Linden M, Heeg C, Reinert RR. Risk factor analysis for pneumococcal meningitis in adults with invasive pneumococcal infection. Epidemiol Infect 2010;138(9):1353-8.

Buysse CM, Oranje AP, Zuidema E, Hazelzet JA, Hop WC, Diepstraten AF, et al. Long-term skin scarring and orthopaedic sequelae in survivors of meningococcal septic shock. Arch Dis Child. 2009 May;94(5):381-6.

Centers for Disease Control and Prevention. Epidemiology and Prevention of Vaccine-Preventable Diseases. Atkinson W, Wolfe S, Hamborsky J, McIntyre L, eds. 11th ed. Washington DC: Public Health Foundation, 2009.

Edmond K, Clark A, Korczak VS, Sanderson C, Griffiths UK, Rudan I. Global and regional risk of disabling sequelae from bacterial meningitis: a systematic review and meta-analysis. Lancet Infect Dis. 2010 May;10(5):317-28.

Eidelman LA, Putterman D, Putterman C, Sprung CL. The Spectrum of Septic Encephalopathy: Definitions, Etiologies, and Mortalities. JAMA. 1996;275(6):470-473. doi:10.1001/jama.1996.03530300054040.

Elward K, Hruby N, Christy C. Pneumococcal endocarditis in infants and children: Report of a case and review of the literature. Pediatr Infect Dis J. 1990;9:652.

European Centre for Disease Prevention and Control. Surveillance of invasive bacterial diseases in Europe, 2010. Stockholm: ECDC; 2013.

European Centre for Disease Prevention and Control. Surveillance of invasive bacterial diseases in Europe, 2011. Stockholm: ECDC; 2013.

Haagsma JA, Maertens de Noordhout C, Polinder S, Vos T, Havelaar AH, Cassini A, Devleesschauwer B, Kretzschmar ME, Speybroeck N, Salomon JA. Assessing disability weights based on the responses of 30,660 people from four European countries. Population Health Metrics 2015; 13: 10

Harboe ZB, Thomsen RW, Riis A, et al. Pneumococcal Serotypes and Mortality following Invasive Pneumococcal Disease: A Population-Based Cohort Study. PLoS Med. 2009 May; 6(5): e1000081.

Jefferson T, Ferroni E, Curtale F, et al. Streptococcus pneumoniae in western Europe: serotype distribution and incidence in children less than 2 years old. Lancet Infect Dis 2006;6:405-10.

Jit M. The risk of sequelae due to pneumococcal meningitis in high-income countries: a systematic review and meta-analysis. J Infect 2010;61(2):114-24.

Lin SH, Lai CC, Tan CK, Liao WH, Hsueh PR. Outcomes of hospitalized patients with bacteraemic and non-bacteraemic community-acquired pneumonia caused by Streptococcus pneumoniae. Epidemiol Infect. 2010 Oct 26:1-10. [Epub ahead of print].

Rudan I, Campbell H. The deadly toll of S pneumoniae and H influenzae type b. Lancet 2009; 374(9.693):854-6.

Saldías PF, Viviani GP, Pulgar BD, Valenzuela FF, Paredes ES, Díaz PO. Prognostic factors and mortality in immunocompetent adult patients hospitalized with community-acquired pneumococcal pneumonia. Rev Med Chil 2009; 137(12):1.545-52.

Tunkel AR, Hartman BJ, Kaplan SL, Kaufman BA, Roos KL, Scheld WM, et al: Practice guidelines for the management of bacterial meningitis. Clin Infect Dis 2004, 39(9):1267–1284.

Ziaja M. Septic encephalopathy. Curr Neurol Neurosci Rep. 2013; 13: 383

Legionnaires' disease

Since 2008, the EU case definition focuses solely on Legionnaires' disease, dismissing Pontiac fever cases. Therefore, the present disease outcome tree focuses only on Legionnaires' disease and its sequelae.

Legionnaires' disease is mostly observed in the elderly and conditions associated with immunodeficiency constitute a risk for Legionnaires'.

In rare cases, Legionnaires' disease may also cause extra-pulmonary symptoms, mainly developing cardiac complications (WHO, 2007). Myocarditis, pericarditis, post-cardiomyotomy syndrome or endocarditis are examples of such manifestations although, according to other studies, most of these complication are related to nosocomial infections (Stout, 1997). Extra-pulmonary manifestations are also often observed in immunocompromised patients. For the purpose of this disease model, we focus on community-acquired Legionnaires' cases and extra- pulmonary manifestations are excluded.

Legionnaires' disease causes acute consolidating pneumonia. In most cases, and without testing for the causative agent, pneumonia arising from infection with Legionella pneumophila cannot be distinguished from other types of pneumonia. Symptoms of Legionnaires' disease are an unproductive cough, chest pain, shortness of breath, myalgia and digestive symptoms such as diarrhoea, vomiting and nausea. Patients may also present neurological symptoms such as confusion or deliria (WHO, 2007).

In many cases, the acute phase requires admission to hospital. Studies have shown that in-patient stays in the hospital vary between eight and 13 days (Lettinga, 2002a; von Baum, 2008). However, it may take more than 90 days to recover to the premorbid health state (Lettinga, 2002a) and roentgenographic clearance can take 2–4 months (Edelstein, 2008). For the model the duration of acute Legionnaires' disease is set at 8– 13 days, as stated in one European study (Lettinga, 2002a).

We consider three different health states occurring during the acute phase of the disease, mild (outpatient, uncomplicated cases), moderate (hospitalised, complicated cases not admitted to an intensive-care unit) and severe (complicated cases admitted to an intensive care unit). Studies have shown that hospitalisation is required in 69–74% of Legionnaires' cases (von Baum, 2008; Garcia-Fulgueiras, 2003). We therefore assume that 26–31% of cases will be mild. Moreover, it is shown that 30% of hospitalised cases require a stay in an intensive-care unit (ICU) (Lettinga, 2002b), thus the proportion of complicated cases (not requiring ICU) is set to 46.7–53.2% and those requiring ICU is set to 20.7– 22.2% of all symptomatic infections.

The case-fatality proportion (CFP) differs widely and is associated with the severity level. The CFP for severe cases was found to be higher, ranging from 10 to 30% (Lettinga, 2002b; Benin, 2002; Falco, 1991). In a review conducted by WHO, case-fatality proportions of community- acquired infections ranged from 5 to 10% (WHO, 2007; Benin, 2002; Howden, 2003). The European working group on Legionella infections (EWGLI) suggested a 12% case-fatality in Europe (von Baum, 2008). In our model, CFP for uncomplicated and complicated cases not requiring a stay in an ICU is set at 5–12% and 10–30% for severe cases requiring an ICU.

Risk of complications

Legionnaires' disease is associated with pulmonary (e.g. severe respiratory failure, pulmonary abscess and pleural empyema), cardiac (e.g. acute pericarditis, myocarditis), neuromuscular (e.g. headache, confusion, fatigue) and renal (e.g. acute renal failure, interstitial nephritis) complications. Multi-organ involvement or septic shock are also possible. In the outcome-tree these complications are not treated separately as they are part of the acute phase of Legionnaires' disease.

Studies on the long-term sequelae of Legionnaires' are scarce, however some reported consequences up to two years after the initial infection (Lattimer, 1979). Two studies reported fatigue in 58–81% of cases, concentration problems and memory loss in 6–81%, muscle/joint pain or muscle weakness in 25–79% and post-traumatic stress disorder in 15% (Lattimer, 1979; Lettinga, 2002a). Given the lack of evidence on the causality of Legionnaires' and the long-term consequences, these were not considered.

Model input summary

Table 1. Transition probabilities and distributions used in the outcome tree

Health outcome (Health state)	Distribution of health states in health outcome	Transition probability	Source/assumption
Symptomatic infection			von Baum, 2008; Garcia-Fulgueiras, 2003; Lettinga, 2002b
(Uncomplicated)	26–31%		
(Complicated)	46.7–53.2%		
(Complicated ICU)	20.7–22.2%		
Fatal cases			Lettinga, 2002b; Benin, 2002; Falco, 1991; WHO, 2007; Benin, 2002; Howden, 2003; von Baum, 2008
(Uncomplicated)		5–12%	
(Complicated)		5–12%	
(Complicated ICU)		10–30%	

Table 2. Disability weights and duration

Health outcome (Health state)	Disability Weight (DW) (Haagsma, 2015)		Duration	
	DW	Label	In years	Source
Symptomatic infection		Infectious disease, acute episode, moderate	0.022–0.036	Lettinga, 2002a; von Baum, 2008
(Uncomplicated)	0.051 (0.039–0.06)	Infectious disease, acute episode, severe		
(Complicated)	0.125 (0.104–0.152)	Intensive care unit admission		
(Complicated ICU)	0.655 (0.579–0.727)			

References

Edelstein PH. Detection of antibodies to Legionella. In Manual of Molecular and Clinical Laboratory Immunology. Edited by Detrick B, Hamilton R, Folds J. Washington, DC: ASM Press; 2006: 468-476

Haagsma JA, Maertens de Noordhout C, Polinder S, Vos T, Havelaar AH, Cassini A, Devleesschauwer B, Kretzschmar ME, Speybroeck N, Salomon JA. Assessing disability weights based on the responses of 30,660 people from four European countries. Population Health Metrics 2015; 13: 10

World Health Organization: and the prevention of legionellosis. Geneva; 2007.

Stout JE, Yu VL. Legionellosis. N Engl J Med 1997, 337:682-687.

Lettinga KD, Verbon A, Nieuwkerk PT, Jonkers RE, Gersons BP, Prins JM, et al. Health-related quality of life and post-traumatic stress disorder among survivors of an outbreak of Legionnaires disease. Clin Infect Dis 2002, 35:11-17.

von Baum H, Ewig S, Marre R, Suttorp N, Gonschior S, Welte T, et al. Community-acquired Legionella pneumonia: new insights from the German competence network for community acquired pneumonia. Clin Infect Dis 2008, 46:1356-1364.

Edelstein PH: Legionnaires' Disease: History and Clinical findings. In Legionella: Molecular Microbiology. Edited by Heuner K, Swanson M. Portland, Oregan: Caister Academic Press; 2008: 1-17

Garcia-Fulgueiras A, Navarro C, Fenoll D, Garcia J, Gonzalez-Diego P, Jimenez-Bunuales T, et al. Legionnaires' disease outbreak in Murcia, Spain. Emerg Infect Dis 2003, 9:915-921.

Lettinga KD, Verbon A, Weverling GJ, Schellekens JF, Den Boer JW, Yzerman EP, et al. Legionnaires' disease at a Dutch flower show: prognostic factors and impact of therapy. Emerg Infect Dis 2002, 8:1448-1454.

Benin AL, Benson RF, Arnold KE, Fiore AE, Cook PG, Williams LK, et al. An outbreak of travel-associated Legionnaires disease and Pontiac fever: the need for enhanced surveillance of travel-associated legionellosis in the United States. J Infect Dis 2002, 185:237-243.

Howden BP, Stuart RL, Tallis G, Bailey M, Johnson PD. Treatment and outcome of 104 hospitalized patients with Legionnaires' disease. Intern Med J 2003, 33:484-488.

Falco V, Fernandez de Sevilla T, Alegre J, Ferrer A, Martinez Vazquez JM. Legionella pneumophila. A cause of severe community-acquired pneumonia. CHEST Journal 1991, 100:1007-1011.

Lattimer GL, Rhodes LV, 3rd, Salventi JS, Galgon JP, Stonebraker V, Boley S, et al. The Philadelphia epidemic of Legionnaire's disease: clinical, pulmonary, and serologic findings two years later. Ann Intern Med 1979, 90:522-526.

Listeriosis

Acquired listeriosis

Listeriosis is an infection caused by the gram-positive bacterium Listeria monocytogenes. The infection is generally asymptomatic but can become extremely severe in immunocompromised patients, pregnant women and their foetuses/newborn and elderly. The severity of the disease is related to its invasiveness: if the infection is not invasive, it will generally cause mild or no symptoms and therefore no burden (with the exception of acute gastroenteritis if a person ingests a large amount of bacteria). Therefore, it is not surprising that most notified cases are invasive listeriosis diseases, hence complicated ones. In order to estimate the number of complicated cases we referred to the US Centers for Disease Control's 2012 and 2011 Listeriosis Annual Surveillance Summaries (CDC, 2014), reporting 95–97% of cases as invasive, and we applied this to the proportion of complicated symptomatic cases.

Manifestations of listeriosis are meningitis, septicaemia, pneumonia, and gastroenteritis. Based on reports from enhanced surveillance in the Netherlands (Doorduyn, 2006 a,b) and a Gamma distribution used to express the uncertainty, Kemmeren et al. (Kemmeren, 2006) and Haagsma et al. (Haagsma, 2009) estimated the distribution of these health states for acquired listeriosis. However, from a clinical perspective it is conceivable that most cases present a mixed form of the disease and isolates are available from multiple anatomical sites. We therefore defined symptomatic infections as either complicated (invasive) or uncomplicated.

In order to determine those long-term sequelae which are linked only to the manifestation of meningitis, we looked at enhanced surveillance in a few European countries, however data on the risk of developing meningitis during invasive listeriosis disease was inconsistent. Therefore, we referred to CDC enhanced surveillance in the USA from 2007 to 2012 and estimated that 13–18% of invasive (complicated) symptomatic cases would present with meningitis (CDC, 2014).

In the current model, the age-specific case fatality proportion related to listeriosis is derived from cases of acquired listeriosis notified to TESSy from 2009 to 2013 (see Table 3) by all EEA Member States except Bulgaria and Lithuania because they report only aggregate data. The case fatality proportion is applied to complicated cases only.

Perinatal listeriosis

Perinatal listeriosis encompasses both pregnant women and their foetuses or newborns. Of the pregnant women with listeriosis, around two out of three will present with prodromal influenza-like symptoms such as fever, chills and headache. Three to seven days after the prodromal symptoms, the pregnant woman may abort the foetus or have premature labour (Gellin, 1989). To the mother, listeriosis is rarely life- threatening, however, infection in the first trimester of pregnancy may result in spontaneous abortion and, in later stages, in stillbirth or a critically ill newborn (Farber, 1991a). Newborns may present with an early-onset or a late-onset form of listeriosis. Early-onset listeriosis is defined as a case of symptomatic listeriosis in a newborn that is less than seven days old. Early-onset listeriosis is acquired by the foetus prenatally. Newborns with early-onset listeriosis mostly develop sepsis and meningitis (Farber, 1991b; Mylonakis, 2002). Late-onset listeriosis is defined as symptomatic listeriosis in a newborn during the first eight to 28 days of life. In this case, the unborn child is infected during childbirth when passing through the birth canal. Newborns with late-onset listeriosis are usually born healthy and at full term, but are at higher risk of developing meningitis during their first weeks of life (Farber, 1991a).

In the current study, the disease burden for health outcomes of early- and late-onset listeriosis are combined into one category. Based on data reported to TESSy between 2009 and 2013, the case fatality proportion was set to 18.71%.

Risk of complications

Long-term sequelae due to meningitis may occur, and will therefore be considered in the outcome tree. The frequency of other post-infectious complications following listeriosis is low (Haagsma, 2009) and therefore they have been disregarded in the current study.

According to Aouaj et al. (Aouaj, 2002), 20% of all listeriosis cases in their study are perinatal. Therefore, of the 147 cases analysed for long- term outcomes (Aouaj, 2002), we estimated that there were 118 acquired cases (29 perinatal). The study stated that 15 (12.7%) of the total number of acquired listeriosis cases presenting meningitis developed neurological long-term sequelae.

Given that 13–18% of all acute cases present meningitis, the risk of developing neurological long-term sequelae from all cases of complicated acquired listeriosis is 1.65–2.29%.

Similarly, knowing that seven of the 29 perinatal listeriosis cases (24%) developed long-term neurological sequelae and that all acute cases present meningitis, the risk of developing life-long neurological disabilities from a perinatal listeria infection is 24%.

Model input summary

Table 1. Transition probabilities and distributions used in the outcome tree

Health outcome<br><br>(Health state)	Distribution of health states<br><br>in health outcome	Transition probability	Source/assumption
Acquired listeriosis			
Symptomatic infection<br><br>(Uncomplicated)<br><br>(Complicated)	3–5%<br><br>95–97%		CDC, 2014

Fatal cases		Age dependent (Table 3)	TESSy 2009–2013
Permanent disability following meningitis		1.65–2.29% of complicated cases	Aouaj, 2002; CDC 2014
Perinatal listeriosis			
Fatal cases		18.71%	TESSy 2009–2013
Permanent disability due to meningitis		24%	Aouaj, 2002

Table 2. Disability weights and duration

Health outcome (Health state)	Disability Weight (DW) (Haagsma, 2015)		Duration	
	DW	Label	In years	Source
Acquired listeriosis				
Symptomatic infection (Uncomplicated) (Complicated)	0.149 (0.12–0.182)<br>0.655 (0.579–0.727)	Diarrhoea, moderate<br>Intensive care unit admission	0.02–0.5	Kemmeren, 2006<br>Haagsma, 2009;
Permanent disability following meningitis	0.011–0.421	From lowest to highest motor and cognitive difficulties	Remaining life expectancy	
Perinatal listeriosis				
Symptomatic infection	0.655 (0.579–0.727)	Intensive care unit admission	0.02–0.5	Kemmeren 2006 & Haagsma 2009
Permanent disability due to meningitis	0.011–0.421	From lowest to highest motor and cognitive difficulties	Remaining life expectancy	

Table 3. Age-group acquired listeriosis case fatality proportion based on cases and deaths notified to TESSy (2009– 2013)

Age groups	%
0	11.90
1-4	0.00
5-9	5.88
10-14	20.00
15-19	13.16
20-24	1.75
25-29	4.10
30-34	1.39
35-39	8.40
40-44	12.50
45-49	14.08
50-54	16.59
55-59	13.77

60-64	18.16
65-69	15.65
70-74	15.17
75-79	17.83
80-84	17.35
>85	23.15
All ages	15.74

References

Aouaj Y, Spanjaard L, van Leeuwen N, Dankert J. (2002). Listeria monocytogenes meningitis: serotype distribution and patient characteristics in The Netherlands, 1976-95. Epidemiol Infect, 128(3):405-409.

CDC (2014). Centers for Disease Control and Prevention, National Surveillance of Bacterial Foodborne Illnesses (Enteric Diseases) National Listeria Surveillance. Available at: http://www.cdc.gov/nationalsurveillance/listeria-surveillance.html [accessed 2 October 2014].

Doorduyn Y, de Jager CM, van der Zwaluw WK, Wannet WJ, van der Ende A, Spanjaard L, et al. (2006a). Invasive Listeria monocytogenes infections in the Netherlands, 1995-2003. Eur J Clin Microbiol Infect Dis, 25(7):433-442.

Doorduyn Y, de Jager CM, van der Zwaluw WK, Wannet WJ, van der Ende A, Spanjaard L, et al (2006b). First results of the active surveillance of Listeria monocytogenes infections in the Netherlands reveal higher than expected incidence. Euro Surveill, 11(4):E060420 060424.

Doorduyn Y, De Jager CM, Van der Zwaluw WK, Wannet WJB, Heuvelink AE, Van der Ende A, et al. (2007). Intensieve surveillance van Listeria monocytogenes in Nederland, 2006. Infectieziekten Bulletin, 9:308-314.

Farber JM, Peterkin PI (1991a). Listeria monocytogenes, a food-borne pathogen. Microbiol Rev, 55(3):476-511.

Farber JM, Peterkin PI, Carter AO, Varughese PV, Ashton FE, Ewan EP. (1991b). Neonatal listeriosis due to cross-infection confirmed by isoenzyme typing and DNA fingerprinting. J Infect Dis, 163(4):927-928.

Haagsma JA, van der Zanden BP, Tariq L, van Pelt W, van Duynhoven YTHP, Havelaar AH (2009). Disease burden and costs of selected foodborne pathogens in the Netherlands, 2006. Bilthoven: National Institute of Public Health and Environment. RIVM rapport 330331001. Available online at: www.rivm.nl/bibliotheek/rapporten/330331001.pdf

Haagsma JA, Maertens de Noordhout C, Polinder S, Vos T, Havelaar AH, Cassini A, Devleesschauwer B, Kretzschmar ME, Speybroeck N, Salomon JA. Assessing disability weights based on the responses of 30,660 people from four European countries. Population Health Metrics 2015; 13: 10

Kemmeren JM, Mangen MJJ, van Duynhoven YTHP, Havelaar AH. (2006). Priority setting of foodborne pathogens: disease burden and costs of selected enteric pathogens. Bilthoven: National Institute of Public Health and Environment: RIVM rapport 330080001. Available online at: www.rivm.nl/bibliotheek/rapporten/330080001.pdf

Lorber B (1997). Listeriosis. Clin Infect Dis, 24(1):1-9; quiz 10-11.

McLauchlin J, Mitchell RT, Smerdon WJ, Jewell K. (2004). Listeria monocytogenes and listeriosis: a review of hazard characterisation for use in microbiological risk assessment of foods. Int J Food Microbiol, 92(1):15-33.

Mylonakis E, Paliou M, Hohmann EL, Calderwood SB, Wing EJ. (2002). Listeriosis during pregnancy: a case series and review of 222 cases. Medicine (Baltimore), 81(4):260-269.

Measles

According to the US Centers for Disease Control and Prevention (CDC, 2012), approximately 30% of reported symptomatic measles cases have one or more complications. The most important complications are: otitis media (occurring in approximately 10% of infected cases), encephalitis (0.1% of cases), and post-infectious encephalomyelitis (0.1–0.3% of cases). Other complications of acute measles include pneumonia (5–6% of untreated cases; Kabra, 2008; CDC, 1991) and diarrhoea (8%) (CDC, 2012). Convulsions are also a relatively frequent complication (5% of cases; Miller, 1978). Complications during pregnancy occur in up to 30% of women with severe measles (Atmar, 1992).

Complications occurring during the acute phase of the disease may overlap and cannot be treated as independent. Two health states were therefore used in our model: complicated and uncomplicated. We derived the risk of complications from data reported to TESSy between 2006 and 2013. Given the high number of cases notified to TESSy without information on complications and in order to account for this uncertainty we included two scenarios. We estimated the proportion of cases reported as uncomplicated out of the number of known cases as 57.24% (excluding cases for which complications were reported as unknown or left blank). We then added the uncomplicated cases to the unknown and blank and obtained the total number 88.64% (assuming that all unknown and blank cases were uncomplicated).

In the model, the rare permanent disabilities due to otitis media, encephalitis, post-infectious encephalomyelitis and subacute sclerosing panencephalitis (SSPE) (van Steenbergen, 2006) are treated as distinct sequelae.

Otitis media and permanent disability due to otitis media

The health state otitis media occurs in around one in ten cases of acute measles and can result in permanent hearing loss (CDC, 2011). The probability of developing permanent disability due to otitis media is 0.01% (CDC, 1991) of all cases of otitis media, therefore the overall risk of developing a permanent disability has been set to 0.001%.

Encephalitis and permanent disability due to encephalitis

Encephalitis occurs in approximately 0.1% of acute symptomatic cases (Weissbrich, 2003; Beutels, 2002; Miller, 1957). Long-term sequelae of measles encephalitis are reported to occur in 20–30% of measles-related encephalitis cases (Beutels, 2002; Filia, 2007); therefore the transition probability for the health outcome 'permanent disability due to encephalitis' was set to 0.02-0.033%.

Encephalitis of the delayed type (Barthez Carpentier, 1992) can occur after acute illness in immunocompromised patients and may occur after asymptomatic infection (Kidd, 2003). Because of the specific population affected, and its relative rarity, the outcome tree was not modified accordingly.

Post-infectious encephalomyelitis (PIE) and permanent disability due to PIE

Post-infectious encephalomyelitis occurs in 1–3 per 1 000 infected persons, usually three to ten days after the onset of rash. Higher rates of PIE due to measles occur in adolescents and adults than in school-aged children (Perry & Halsey, 2004). The condition is associated with demyelination and is thought to have an autoimmune basis. A total of 33% of those afflicted with PIE who survive have lifelong neurological sequelae, including severe retardation, motor impairment, blindness and sometimes hemiparesis (Perry & Halsey, 2004). The transition probability in the model for developing the health outcome 'permanent disability due to PIE' was set to the range 0.033–0.1%.

Subacute sclerosing panencephalitis (SSPE)

On average, the symptoms of SSPE begin seven to ten years after measles infection, but they can appear anytime from one month to 27 years after infection (CDC, 2012).

Various estimates are available for the proportion of cases that develop the SSPE health outcome. SSPE is observed at a rate of 1 per 10 000– 20 000 (Weissbrich, 2003; Takasu, 2003; Bellini, 2005; Garg, 2008). In children who have previously had natural measles, the risk of developing SSPE is between 0.6 and 2.2 per 100 000 cases (Hosoya, 2006). Other estimates include: one SSPE case in every 100 000 cases of measles (Rezende, 1989); 4–11 cases of SSPE per 100 000 cases of measles (CDC, 2009); one in every 25 000 measles infections (Miller, 2004); one in 8 000 for children under two years (Miller, 1992; 2004) and a 16-fold greater risk for those infected under one year of age compared with those over five years (Miller, 1992). The risk of developing SSPE is known to be age-specific (Beutels, 2002; Farrington, 1991; Miller, 2004; CDC, 2012).Therefore, transitional probabilities in the model were also specified as age-dependent (see Table 3) (Beutels, 2002). In the model, the duration for this health outcome was specified as one to two years (CDC, 2012). In the model the transition probability from SSPE to death was set to 100%.

Case fatality proportion

Measles is fatal in approximately 0.05–0.1% of cases (Wolfson, 2007; Lozano, 2012). The risk of death is higher among young children and adults (CDC, 2012). According to CDC (CDC, 2012), the most common causes of death are pneumonia in children and acute encephalitis in adults, but due to the lack of specific data for different age groups we applied the same CFP for all the same age groups.

Model input summary

Table 1. Transition probabilities used in the outcome tree

Health outcome (Health state)	Distribution of health states in health outcome	Transition probability	Source/assumption

Symptomatic infection<br>(Complicated)<br>(Uncomplicated)	11.36-42.76%<br>57.24-88.64%		TESSy, 2006–2013
Permanent disability following otitis media		0.001%	CDC, 1991
Permanent disability following encephalitis		0.02–0.033%	Beutels, 2002; Filia, 2007
Permanent disability following PIE		0.033–0.1%	Perry & Halsey, 2004
SSPE		See Table 3	Beutels, 2002
Fatal cases following SSPE		100%	
Fatal cases following symptomatic infection		0.05–0.1%	Wolfson, 2007; Lozano, 2012

Table 2. Disability weights and duration

Health outcome (Health state)	Disability Weight (DW) (Haagsma, 2015)		Duration	
	DW	Label	In years	Source
Symptomatic infection<br>(Complicated)<br>(Uncomplicated)	0.125 (0.104-0.152)<br>0.051 (0.039-0.06)	Infectious disease, acute episode, severe<br>Infectious disease, acute episode, moderate	0.03	Kwong, 2012
Permanent disability due to otitis media	0.008-0.103	From lowest to highest hearing loss related DWs	Remaining life expectancy	
Permanent disability due to encephalitis	0.054-0.425	From lowest to highest Motor plus cognitive impairments related DWs	Remaining life	

			expectancy	
Permanent disability due to PIE	0.054-0.425	From lowest to highest Motor plus cognitive impairments related DWs	Remaining life expectancy	
Latency period before SSPE	0		0.082–27	CDC, 2012
SSPE	0.276 (0.088-0.543)	From Phase 1 to Phase 3 (median is Phase 2) of subacute sclerosing panencephalitis related DWs	1–2	CDC, 2012

Table 3. Transition probabilities subacute sclerosing panencephalitis (SSPE)

Age	%
0-4	0.0081
5-9	0.0011
≥10	0.0010

References

Barthez Carpentier MA, Billard C, Maheut J, Jourdan ML, Degenne D, Ruchoux MM, et al. Acute measles encephalitis of the delayed type: neuroradiological and immunological findings. Eur Neurol. 1992;32(4):235-7.

Bellini WJ, Rota JS, Lowe LE, et al (2005). Subacute sclerosing panencephalitis: More cases of this fatal disease are prevented by measles immunization than previously recognized. J Infect Dis 192:1686–1693.

Beutels P, Van Damme P, Van Casteren V, Gay NJ, De Schrijver K, Meheus A. The difficult quest for data on "vanishing" vaccine-preventable infections in Europe: the case of measles in Flanders (Belgium). Vaccine. 2002 Oct 4;20(29-30):3551-9.

Centers for Disease Control (CDC). Measles: United States, 1990. MMWR Morb Mortal Wkly Rep 1991;40:369–372.

Centers for Disease Control and Prevention. Epidemiology and Prevention of Vaccine-Preventable Diseases. Atkinson W, Wolfe S, Hamborsky J, McIntyre L, eds. 11th ed. Washington DC: Public Health Foundation, 2009

Centers for Disease Control and Prevention (CDC). The Pink Book: Course Textbook - 12th Edition Second Printing (May 2012).

Farrington CP. Subacute sclerosing panencephalitis in England and Wales: transient effects and risk estimates. Stat Med 1991;10:1733–1744;

Filia A, Brenna A, Panà A, Maggio Cavallaro G, Massari M, Ciofi degli Atti LM. Health burden and economic impact of measles-related hospitalizations in Italy in 2002–2003. BMC Public Health. 2007; 7: 169

Garg RK (2008). Subacute sclerosing panencephalitis. J Neurol 255(12):1861–1871.

Haagsma JA, Maertens de Noordhout C, Polinder S, Vos T, Havelaar AH, Cassini A, Devleesschauwer B, Kretzschmar ME, Speybroeck N, Salomon JA. Assessing disability weights based on the responses of 30,660 people from four European countries. Population Health Metrics 2015; 13: 10

Hosoya M. Measles encephalitis: direct viral invasion or autoimmune-mediated inflammation? Intern Med. 2006;45(14):841-2.

Kabra SK, Lodha R, Hilton DJ. Antibiotics for preventing complications in children with measles. Cochrane Database of Systematic Reviews 2008, Issue 3. Art. No.: CD001477. DOI: 10.1002/14651858.CD001477.pub3.

Kwong JC, Ratnasingham S, Campitelli MA, Daneman N, Deeks SL, et al. (2012) The Impact of Infection on Population Health: Results of the Ontario Burden of Infectious Diseases Study. PLoS ONE 7(9): e44103. doi:10.1371/journal.pone.0044103

Lozano R, Naghavi M, Foreman K, Lim S, Shibuya K, Aboyans V, et al. (Dec 15, 2012). Global and regional mortality from 235 causes of death for 20 age groups in 1990 and 2010: a systematic analysis for the Global Burden of Disease Study 2010. Lancet 380 (9859): 2095–128

Miller HG, Stanton JB, Gibbons JL. Acute disseminated encephalomyelitis and related syndromes. British medical journal.1957 Mar 23;1(5020):668-72

Miller CL. Severity of notified measles. British Medical Journal. 1978; 1(6122): 1253-1255.

Miller C, Andrews N, Rush M, Munro H, Jin L, Miller E. The epidemiology of subacute sclerosing panencephalitis in England and Wales 1990-2002. Arch Dis Child. 2004 Dec;89(12):1145-8.

Murray CJL, Lopez AD, editors. The global burden of disease: a comprehensive assessment of mortality and disability from diseases, injuries, and risk factors in 1990 and projected to 2020. Cambridge: Harvard University Press, 1996.

Rezende MA. Nursing care and the neurological sequelae of measles. Revista da Escola de Enfermagem USP. 1989 Apr;23(2):141-8.

Stein CE, et al, The Global Burden of Measles in the Year 2000—A Model that Uses Country-Specific Indicators. Journal of Infectious Diseases 2003; 187(Suppl 1):S8–14.

Takasu T, Mgone JM, Mgone CS, et al (2003). A continuing high incidence of subacute sclerosing panencephalitis (SSPE) in the eastern highlands of Papua New Guinea. Epidemiol Infect 131:887–898.

Tishler M, Abramov AL. Liver involvement in measles infection of young adults. Isr J Med Sci. 1983 Sep;19(9):791-3.

Wolfson LJ, Strebel PM, Gacic-Dobo M, Hoekstra EJ, McFarland JW, et al. (2007) Has the 2005 measles mortality reduction goal been achieved? A natural history modelling study. Lancet 369: 191–200

Zhou F, Reef S, Massoudi M, Papania MJ, Yusuf HR, Bardenheier B, et al. An Economic Analysis of the Current Universal 2-Dose Measles-Mumps- Rubella Vaccination Program in the United States. The Journal of Infectious Diseases. 2004 May 1;189 Suppl 1:S131-45.

Mumps

Mumps is symptomatic in 80% of infections (CDC, 2012), the main symptom being parotitis.

Risk of complications

The principal complications with mumps are orchitis, oorphoritis, meningitis, pancreatitis, and encephalitis.

Epididymo-orchitis occurs in 15–30% of adult men with mumps infection, but it is rare before puberty (Hviid, 2008). Oophoritis (ovarian inflammation), the counterpart of orchitis in females, is associated with pelvic pain and tenderness. It occurs in 5% of post-pubertal females (CDC, 2009).

Mumps meningitis is a benign entity with no significant risk of mortality or long-term sequelae. Even though cerebrospinal fluid pleiocytosis occurs in about half of the patients with mumps, clinical manifestations of meningitis arise in 1–10% of the cases (Hviid, 2008), and long-term morbidity is rare. Encephalitis occurs in 0.1% of acute cases (Hviid, 2008).

Acute pancreatitis, with symptoms of abdominal distention and pain, fever, nausea, and vomiting (Demirci, 2011), occurs in approximately 4% of mumps cases (Vanlioglu & Chua, 2011).

With mumps, the acute complications of symptomatic infections are considered as a single health state (complicated) because they can occur concomitantly.

Of all mumps infections, 40–50% may have only non-specific or primarily respiratory symptoms (CDC, 2012). Therefore, knowing that 20% of infections are asymptomatic, 32–40% of symptomatic cases were considered to be uncomplicated. Durations were set to 7–10 days for the uncomplicated cases and 7–14 days for the complicated ones.

Permanent deafness caused by mumps occurs with an estimated frequency of one in 20 000 cases (0.005%) and in 80% of the cases, hearing loss is monolateral (Hviid, 2008).

Case fatality proportion

Death is very rare in mumps cases and the mortality rate following encephalitis is 1.5%. Therefore, 0.15% was used in the model for the risk of death resulting from all symptomatic infections. More than half of fatalities occur in patients over 19 years (Hviid, 2008; Demirci, 2011). This age distribution also applies to the symptomatic complicated cases (see Table 3).

Model input summary

Table 1. Transition probabilities used in the outcome tree

Health outcome (Health state)		Distribution of health states in health outcome		Transition probability		Source/assumption

Symptomatic infection<br>(Uncomplicated)<br>(Complicated)	32–40%<br>60–68%		CDC, 2012
Permanent disability due to hearing loss		0.005%	Hviid, 2008
Fatal cases		0.15%<br>Age dependent (see Table 3)	Hviid, 2008<br>Assuming 1.5% of encephalitis cases (0.1%) become fatal

Table 2. Disability weights and duration

Health outcome (Health state)	Disability Weight (DW) (Haagsma, 2015)		Duration	
	DW	Label	In years	Source
Symptomatic infection<br>Uncomplicated<br>Complicated	0.051 (0.039-0.06)<br>0.125 (0.104-0.152)	Infectious disease, acute episode, moderate<br>Infectious disease, acute episode, severe	0.019-0.027<br>0.019-0.038	Hviid, 2008
Permanent hearing loss	0.008 (0.005-0.012)	Unilateral hearing loss	Remaining life expectancy	Hviid, 2008

Table 3. Age distribution – case fatality ratio

0-19	50
≥20	50

References

Centers for Disease Control and Prevention. Epidemiology and Prevention of Vaccine-Preventable Diseases. Atkinson W, Wolfe S, Hamborsky J, eds. 12th ed., second printing. Washington DC: Public Health Foundation, 2012.

Demirci CS, Abuhammour W, Quintana EC, Shahidi H, Wilkes G. Mumps. Available from http://emedicine.medscape.com/article/966678- overview [accessed 12 September, 2011].

Haagsma JA, Maertens de Noordhout C, Polinder S, Vos T, Havelaar AH, Cassini A, Devleesschauwer B, Kretzschmar ME, Speybroeck N, Salomon JA. Assessing disability weights based on the responses of 30,660 people from four European countries. Population Health Metrics 2015; 13: 10

Hviid A, Rubin S, Mühlemann K. Mumps. Lancet. 2008 Mar 15;371(9616):932-44.

Mandell GL, Bennet JE, Dolin R. Mandell, Douglas and Bennett's Principles and Practice of Infectious Disease 5th edition. Churchill Livingstone, 1999.

Vanlioglu B, Chua TC. Presentation of mumps infection as acute pancreatitis without parotitis. Pancreas. 2011 Jan;40(1):167-8.

Pertussis

Pertussis is principally toxin-mediated. Toxins paralyse the cilia of the respiratory tract cells, leading to the clinical features and complications of the disease. The clinical course of the illness is divided into three stages. The first one is the catarrhal stage, characterised by coryza, sneezing, low-grade fever and a mild, occasional cough. The cough gradually becomes more severe, and after 1–2 weeks, the paroxysmal stage begins, usually lasting one to six weeks. In the convalescent stage, which lasts two to three weeks, recovery is gradual and the cough becomes less paroxysmal. However, paroxysms often recur for many months after the onset of pertussis (CDC, 2009; Mandell, 1999).

Clinical manifestations of pertussis may be mild in adults and vaccinated children. Around 20% of infected persons develop mild/asymptomatic disease (Rothstein, 2005). Based on this finding, an asymptomatic proportion of 20% was specified in the model.

Risk of complications

The principal complications of pertussis are secondary infections, such as otitis media and pneumonia, neurological complications, such as seizures and encephalopathy. Other possible complications include physical sequelae of paroxysmal cough (e.g. subconjunctival haemorrhages, epistaxis, petechiae, central nervous system haemorrhage, pneumothorax and hernia) (CDC, 2009; Mandell, 1999).

Pneumonia can result from aspiration during whooping and vomiting or from impaired clearance mechanisms. It occurs in 5.2% of all patients (CDC, 2009), in up to 25% of cases reported in infants (Mandell, 1999), in 2–4% of individuals aged 10–19 years, in 2.7–5.5% of those over 20 years and in 5–9% of those over 30 years (Rothstein, 2005).

Approximately 4% of adolescents and adults with symptomatic pertussis infection develop otitis media (De Serres, 2000).

Neurological complications of pertussis are more common among infants. In children 12 months of age or younger with pertussis in the USA (1980–1989), convulsions occurred in 3.0% and encephalopathy in 0.9% of cases. Encephalopathy, febrile and afebrile convulsions occur infrequently in adults with pertussis (CDC, 2009), with encephalopathy observed in 0.1% of cases during the period 1997–2000 (CDC, 2009).

Seizures were reported among 0.8% of all pertussis cases in the period 1997–2000 (CDC, 2009).

Infants with pertussis are at greater risk of complications and permanent sequelae, however complications of pertussis, including serious ones, are not uncommon in adolescents and adults, especially the elderly. Complications occur in up to 23% of patients aged 19–83 years. Complications are more frequent in adults than in adolescents (28% compared to 16%) (CDC, 2009; Mandell, 1999; Rothstein, 2005).

Most complications occurring during the symptomatic acute disease phase overlap with one other. We therefore decided to aggregate all complicated cases into one health state. Risk of complications is reported to be 50% in infants (<1 year), 16% in children and adolescents and 28% in cases 20 years (CDC, 2013).

We assumed that in complete and active surveillance systems, those cases notified represent the complicated cases of pertussis. The United Kingdom has an enhanced surveillance system for pertussis where information is compiled from different sources. We therefore chose to consider the number of cases reported in the UK (2007–2013) as complicated. In order to estimate the proportion of complicated cases, we divided the number of cases reported in the UK by the estimated true incidence of pertussis derived from the literature: 71–507 per 100 000 10 years; 46 per 100 000 <10 years (Wirsing von Konig, 2002; Diez-Domingo, 2004) (see Table 3).

Case fatality proportion

Death from pertussis is rare beyond the age of 10 years, occurring in less than 0.1% of all cases, with older adults being at greater risk than younger adults (Rothstein, 2005). Pneumonia is a leading cause of death, but in a study of 99 patients aged 55–94 years who died of pertussis (Rothstein, 2005), intracranial haemorrhage was the cause of death for two of the four deaths thought to be associated with pertussis. Among patients who died, apnoea, pneumonia, seizures, and encephalopathy were reported for 58% (40 of 69), 54% (39 of 72), 21% (14 of 68), and 12% (7 of 57), respectively (Rothstein, 2005; Farizo, 1992).

‘The case fatality proportion in the United States between 1990 and 1996 was 0.2%. Eighty-four per cent of pertussis-related deaths occur in infants younger than six months of age’ (Ratnapalam, 2005).

In general, we considered that only complicated cases were at risk of dying. We used the CFP reported in the UK for deaths of infants <1 year old because of its comprehensive surveillance system, compiling data from different sources and deemed to be capturing approximately 94% of the cases in recent capture-recapture studies. There were 33 deaths due to pertussis reported to TESSy between 2007 and 2013 out of 1 791 cases. This resulted in a CFP of 1.84% which was applied to complicated cases <1 year.

We chose 0.1% of complicated cases for all other age groups.

Model input summary

Table 1. Transition probabilities used in the outcome tree

Health outcome<br><br>(Health state)	Distribution of health states in health outcome	Transition probability	Source/assumption
Symptomatic infection<br><br>(Complicated)<br><br>(Uncomplicated)	Age dependent (see Table 3)<br><br>Remaining cases		CDC, 2013

Fatal cases		1.84% <1 yr.<br>0.1% ≥ 1 yr.	TESSy<br>Rothstein, 2005

Table 2. Disability weights and duration

Health outcome (Health state)	Disability Weight (DW) (Haagsma, 2015)		Duration	
	DW	Label	In years	Source
Symptomatic infection			0.077–0.211	CDC, 2009; Mandell, 1999
(Complicated)	0.125 (0.104–0.152)	Infectious disease, acute episode, severe		
(Uncomplicated)	0.051 (0.039–0.06)	Infectious disease, acute episode, moderate		

Table 3. Risk of complications

Age	%	
	Estimated from low true incidence	Estimated from high true incidence
0	28.04	
01-04	8.04	
05-09	5.85	
10-14	0.35	2.46
15-19	0.39	2.81
20-24	1.05	7.50
25-29	1.59	11.38
30-34	1.92	13.68
35-39	1.45	10.32
40-44	1.84	13.12
45-49	2.23	15.96
50-54	2.00	14.29
55-59	1.68	11.97

60-64	1.20	8.57
65-69	1.48	10.58
70-74	1.24	8.83
75-79	1.30	9.26
80-84	0.91	6.52
85+	0.54	3.88

References

Centers for Disease Control and Prevention. Epidemiology and Prevention of Vaccine-Preventable Diseases. Atkinson W, Wolfe S, Hamborsky J, McIntyre L, eds. 11th Edition. Washington DC: Public Health Foundation, 2009.

Centers for Disease Control and Prevention. http://www.cdc.gov/pertussis/about/complications.html Last update January 2013 [accessed 25 September 2014].

De Serres G, Shadmani R, Duval B, Boulianne N, Déry P, Douville Fradet M, et al. Morbidity of pertussis in adolescents and adults. J Infect Dis. 2000 Jul;182(1):174-9.

Diez-Domingo J, Ballester A, Baldo JM, Planelles MV, Villarroya JV, Alvarez T et al. Incidence of pertussis in persons < 15 years of age in Valencia, Spain: seroprevalence of antibodies to pertussis toxin (PT) in children, adolescents and adults. Journal of Infection (2004) 49, 242- 247

Farizo KM, Cochi SL, Zell ER, Brink EW, Wassilak SG, Patriarca PA. Epidemiological features of pertussis in the United States, 1980-1989. Clin Infect Dis. 1992 Mar;14(3):708-19.

Haagsma JA, Maertens de Noordhout C, Polinder S, Vos T, Havelaar AH, Cassini A, Devleesschauwer B, Kretzschmar ME, Speybroeck N, Salomon JA. Assessing disability weights based on the responses of 30,660 people from four European countries. Population Health Metrics 2015; 13: 10

Mandell GL, Bennet JE, Dolin R. Mandell, Douglas and Bennett’s Principles and Practice of Infectious Disease 5th edition. Churchill Livingstone, 1999.

Ratnapalan S, Parkin PC, Allen U. Case 1: The deadly danger of pertussis. Paediatr Child Health. 2005 Apr;10(4):221-2

Rothstein E, Edwards K. Health burden of pertussis in adolescents and adults. Pediatr Infect Dis J. 2005 May;24(5 Suppl):S44-7. Wirsing von

Konig CH, Halperin S, Riffelmann M, Guiso N. Pertussis of adults and infants. Lancet Infect Dis 2002; 2: 744-50.

Poliomyelitis

Poliomyelitis is an acute illness which may result from invasion of the gastro-intestinal tract by one of three types of polio virus. It usually affects small children under the age of three years. The virus is transmitted through contaminated food and water, and multiplies in the intestine, from where it can invade the nervous system. Transmission occurs through contact with faeces or pharyngeal secretions of an infected person. The incubation period ranges from three to 21 days, but may be longer. Cases are infectious from about ten days before to seven days after the onset of symptoms; however, carriers and some immuno-compromised persons may shed the virus in faeces for longer than six weeks (Howard, 2005).

Most infections are not clinically apparent; up to 95% of infections are asymptomatic (CDC, 2009).

Risk of complications

Clinical disease may range in severity from minor illness (abortive poliomyelitis), to non-paralytic poliomyelitis (aseptic meningitis) and paralytic poliomyelitis (Feigin, 2009).

Approximately 4–8% of polio infections consist of a non-specific 'minor illness' without clinical or laboratory evidence of central nervous system invasion (CDC, 2009; Feigin, 2009). This clinical presentation is known as abortive poliomyelitis, and is characterised by complete recovery in less than one week (CDC, 2009).

Nonparalytic aseptic meningitis (symptoms of stiffness of the neck, back, and/or legs) which usually follows several days after a prodrome similar to that of a minor illness, occurs in 1–2% of polio infections (CDC, 2009). Increased or abnormal sensations can also occur. Typically these symptoms will last from two to ten days, followed by complete recovery (CDC, 2009).

Less than 1% of all polio infections result in flaccid paralysis (CDC, 2009; Heymann, 2004). Paralytic symptoms generally begin one to ten days after prodromal symptoms and progress for two to three days. Generally, no further paralysis occurs after fever subsides (CDC, 2009). Many patients with paralytic poliomyelitis recover completely and, in most of them, muscle function returns to some degree. Weakness or paralysis 12 months after onset is usually permanent (CDC, 2009).

In acute flaccid paralysis (AFP), the legs are usually more often affected than the muscles of the upper body. However, the polio virus may invade the brain stem, potentially leading to breathing difficulty and even death. Symptoms include headache, gastro-intestinal disturbance, malaise and stiffness of the neck and back, with or without paralysis (American Academy of Pediatrics, 2006; Shibuya & Murray, 2002). Improvements are seen within the first six months (Farbu, 2013; Neumann, 2004). The principal complication is painful, acute, asymmetric paralysis of the arms or the legs, reaching its maximum extent over the course of three to four days and leading to permanent lameness of the affected limbs and breathing difficulties (UK Department of Health, 2006; WHO, 2014).

Given the estimates of symptomatic polio cases, we considered that on average 8.5% of infections are symptomatic (6–11%; CDC, 2011); hence 70.59% of cases on average will be abortive (uncomplicated), 17.65% will be non-paralytic and 11.76 will be paralytic.

According to WHO (WHO, 2014), 1 in 200 infections leads to irreversible paralysis. Given that 1% of all infections has a paralytic form, we considered that 50% of all paralytic forms would develop a permanent disability due to paralysis.

Post-polio syndrome is a long-term sequela that occurs 30–35 years after infection in approximately 25–50% of cases (Jubelt & Drucker, 1999). A slowly progressing condition, it can also occur in patients who have had the non-paralytic form of poliomyelitis. The most common symptoms include slow, progressive muscle weakness, fatigue (both generalised and muscular) and a gradual decrease in the size of muscles (muscle atrophy). Pain from joint degeneration and increasing skeletal deformities such as scoliosis (curvature of the spine) is common and may precede the weakness and muscle atrophy. Some individuals experience only minor symptoms while others develop visible muscle weakness and atrophy. Fatigue is clearly the most prominent manifestation, occurring in up to 80% of patients (Jubelt & Drucker, 1999). Post-polio syndrome is rarely life-threatening (NINDS, 2012).

Case fatality proportion

The case fatality proportion is 5–10% of paralytic forms (WHO, 2014).

Model input summary

Table 1. Transition probabilities used in the outcome tree

Health outcome (Health state)	Distribution of health states in health outcome	Transition probability	Source/assumption
Symptomatic infection		6-11%	
(Uncomplicated)	70.59%		CDC, 2009
(Non-paralytic poliomyelitis)	17.65%		CDC, 2009; Heymann, 2004
(Paralytic poliomyelitis)	11.76%		
Post-polio syndrome		25–50%	Jubelt & Drucker, 1999
Permanent disability following paralytic poliomyelitis		50%	WHO, 2014

Fatal cases following paralytic poliomyelitis		5-10%	WHO, 2014

Table 2. Disability weights and duration

Health outcome (Health state)	Disability Weight (DW) (Haagsma, 2015)		Duration	
	DW	Label	In years	Source
Symptomatic infection				
(Uncomplicated)	0.007 (0.005–0.01)	Infectious disease, acute episode, mild	0.019	CDC, 2009
(Non-paralytic poliomyelitis)	0.051 (0.039–0.06)	Infectious disease, acute episode, moderate	0.005–0.027	CDC, 2009
(Paralytic poliomyelitis)	0.125 (0.104–0.152)	Infectious disease, acute episode, severe	0.011–0.038	CDC, 2009
Permanent disability following paralytic poliomyelitis	0.067 (0.054–0.081)	Spinal cord lesion below neck level (treated)	Remaining life expectancy	
Latency period before PPS	0		30–35	Jubelt & Drucker, 1999
Post-polio syndrome (PPS)	0.344 (0.3–0.391)	Musculoskeletal problems, generalised, moderate	Remaining life expectancy	

References

American Academy of Pediatrics. Red Book: 2006 Report of the Committee on Infectious Diseases. 27th ed. Elk Grove Village. 2006.

Centers for Disease Control and Prevention (CDC). Epidemiology and Prevention of Vaccine-Preventable Diseases. Atkinson W, Wolfe S, Hamborsky J, McIntyre L, eds. 11th ed. Washington DC: Public Health Foundation, 2009.

Department of Health, UK. Immunisation against infectious disease (the Green Book). 3rd ed. London: The Stationery Office. 2006.

Feigin R, Cherry J, Demmler-Harrison GJ, Kaplan S. Feigin and Cherry's Textbook of Pediatric Infectious Diseases. Sixth Edition, 2009.

Farbu E. 2013. Post-polio Syndrome. In: JH Stone, M Blouin, editors. International Encyclopedia of Rehabilitation. Available online: http://cirrie.buffalo.edu/encyclopedia/en/article/138/

Haagsma JA, Maertens de Noordhout C, Polinder S, Vos T, Havelaar AH, Cassini A, Devleesschauwer B, Kretzschmar ME, Speybroeck N, Salomon JA. Assessing disability weights based on the responses of 30,660 people from four European countries. Population Health Metrics 2015; 13: 10

Heymann D, Aylward B. Poliomyelitis. Orphanet Encyclopedia. WHO. 2004.

Howard RS. Poliomyelitis and the post polio syndrome. BMJ 2005;330(7503):1314–1318.

Jubelt B, Drucker J. Poliomyelitis and the Post-Polio Syndrome in Motor Disorders. Philadelphia: Lippincott Williams and Wilkins. 1999. pp. 381.

Neumann D. Polio: its impact on the people of the United States and the emerging profession of physical therapy (PDF).The Journal of Orthopaedic and Sports Physical Therapy 2004; 34 (8): 479–92. PMID 15373011

Post-Polio Syndrome Fact Sheet. NINDS. 2012.

Shibuya K, Murray CJL. Poliomyelitis. In: Stein CE, Murray CJL, Lopez AD (eds). The Global Burden of Disease and Injuries Series, Volume 4: The Global Epidemiology of Infectious Diseases. WHO, 2002.

UNICEF Executive Board Special (2009). Focus Session on Global Health, with particular focus on polio eradication. Available online from: http://www.unicef.org/about/execboard/files/Special_focus_session-Background_SFS_English_8_June_09(1).pdf

World Health Organization (WHO). Poliomyelitis. Available from http://www.who.int/topics/poliomyelitis/en/ [accessed 19 September 2014].

Q fever

Q fever infection becomes symptomatic in 40% of cases (Dijkstra, 2012). Symptomatic infections are divided into two health states: uncomplicated and complicated (more severe cases) and the proportion of complications is based on the hospitalisation rate (2–5%) for Q fever (Maurin & Raoult, 1999; Raoult, 2005).

Around 1–2% of Q fever cases are fatal (ECDC, 2010). This CFR is applied to complicated cases only, based on the US Centers for Disease Control (CDC) Fact Sheet which states that 'the case fatality ratio for hospitalized patients is under 2%' (CDC, 2013).

Chronic Q fever

The transition probability that cases with symptomatic infections will develop chronic Q fever is set to 1.6% (1.5–2%) (van der Hoek, 2011; ECDC, 2010). Due to the lack of evidence, development of chronic Q fever was not associated with asymptomatic Q fever (ECDC, 2010). The average duration of chronic Q fever before developing symptoms is 0.5 years (0.08–1.5 years) (Fenollar, 2001) and this is included in the burden calculation as it reduces the life expectancy of later health outcomes.

Taking the duration of treatment as a proxy for the duration of chronic Q fever, we set the duration to 12–18 months (CDC, 2013) although there are studies recommending life-long treatment which could vary from one year to a person's entire lifespan (Forland, 2012). However, we assume that symptoms due to the infection resolve during the treatment; if symptoms continue, we consider them not to be associated with the Q fever infection but with underlying conditions.

The most common manifestation of chronic Q fever is heart failure, of which a quarter of cases show conduction disorders (Marrie, 2010); other possible manifestations include vascular and pulmonary infections and chronic hepatitis (Maurin & Raoult, 1999). Therefore disability weights describing heart failure were applied.

The case fatality proportion for chronic Q fever has been estimated to be from to 5 to 50%, according to time of diagnosis and onset of treatment (ECDC, 2010).

Post-infectious fatigue syndrome

Follow-up studies after large outbreaks provide some information regarding duration and the probability of developing post-infectious fatigue syndrome. One large cohort following an outbreak in the UK used standard clinical criteria to quantify the proportion of patients developing fatigue after five years (Ayres JG, 1998) and ten years (Wildman, 2002). The first follow-up reported a larger proportion of idiopathic chronic fatigue (ICF) in Q fever cases (42.3%) than in matched controls (26%), with a difference of 16.3%. At the 10-year follow-up point, cases were matched to controls for the presence of comorbidities and hospital attendance, but there was still a higher proportion of ICF (21.6% vs. 5.4%), with a difference of 16.2%. A recent study from a Dutch outbreak indicates the proportion of patients with fatigue after 12 to 26 months to be higher (43.5%) than after five or ten years of follow-up (Morroy, 2011). Therefore, two health states were specified in order to differentiate short-term fatigue (43.5–16.2/16.3% = 27.2/27.3%) from long-term fatigue (16.2–16.3%). The short-term health state consists of clinical cases that recover within 12 to 26 months; severe cases are assumed to recover after 10 years. Regarding the sources of post-infectious fatigue syndrome (PFS), it is surprising that after 10 years the proportion of PFS is reduced to the same extent in controls as in the cases. We therefore considered the bias to be prevalent and decided to exclude PFS from the model.

Model input summary

Table 1. Transition probabilities and distributions used in the outcome tree

Health outcome	Distribution of health states in health outcome	Transition probability			Source/assumption

(Health state)			
Symptomatic infection<br>(Mild)<br>(Severe)	95–98%<br>2–5%		Maurin & Raoult, 1999; Raoult, 2005
Chronic Q fever		1.6% (1.5–2%)	van der Hoek, 2011; ECDC, 2010
Fatal cases following symptomatic infection		1-2% of severe cases	ECDC, 2010
Fatal cases following chronic infection		5-50%	ECDC, 2010

Table 2. Disability weights and duration

Health outcome (Health state)	Disability Weight (DW) (Haagsma, 2015)		Duration	
	DW	Label	In years	Source
Symptomatic infection<br>(Mild)	0.007 (0.005-0.01)<br>0.125 (0.104-0.152)	Infectious disease, acute episode, mild	0.038<br>0.038	Stouthard, 1997<br>Stouthard, 1997

(Severe)		Infectious disease, acute episode, severe		
Latency period (before chronic Q fever)			0.5 (0.08-1.5)	Fenollar, 2001
Chronic Q fever	0.173 (0.14-0.205)	Heart failure, severe	1-1.5	CDC, 2013

References

Ayres JG, Flint N, Smith EG, Tunnicliffe WS, Fletcher TJ, Hammond K, et al. Post-infection fatigue syndrome following Q fever. QJM: Monthly Journal of the Association of Physicians, 1998 Feb;91(2):105-23.

Dijkstra F, van der Hoek W, Wijers N, Schimmer B, Rietveld A, Wijkmans CJ, et al. The 2007–2010 Q fever epidemic in the Netherlands: Characteristics of notified acute Q fever patients and the association with dairy goat farming. FEMS Immunology and Medical Microbiology. 2012;64(1):3-12 (epub 2011/11/10).

ECDC Technical Report – Risk Assessment on Q fever. May 2010. Available at:

http://www.ecdc.europa.eu/en/publications/Publications/1005_TER_Risk_Assessment_Qfever.pdf

Fenollar F, Fournier PE, Carrieri MP, Habib G, Messana T, Raoult D. Risks factors and prevention of Q fever endocarditis. Clinical Infectious Diseases, 2001 Aug 1;33(3):312-6.

Forland F, De Carvalho Gomes H, Nokleby H, Escriva A, Coulombier D, Giesecke J, et al. Applicability of evidence-based practice in public health: risk assessment on Q fever under an ongoing outbreak. Euro Surveill. 2012;17(3):pii=20060. Available at: http://www.eurosurveillance.org/ViewArticle.aspx?ArticleId=20060

Haagsma JA, Maertens de Noordhout C, Polinder S, Vos T, Havelaar AH, Cassini A, Devleesschauwer B, Kretzschmar ME, Speybroeck N, Salomon JA. Assessing disability weights based on the responses of 30,660 people from four European countries. Population Health Metrics 2015; 13: 10

Karakousis PC, Trucksis M, Dumler SJ: Chronic Q fever in United States. J Clin Microbiol 2006;44: 2283–2287.

Marrie TJ, Raoult D, 2010. Coxiella burnetii (Q Fever). In Mandell, Douglas, and Bennett's Principles and Practice of Infectious Diseases [eds] Gerald L. Mandell, John E. Bennett, Raphael Dolin.—7th edition 2010, Chapter 189, 2511-2519.Maurin M, Raoult D. Q fever. Clinical Microbiology Reviews. 1999;12(4):518-53 (epub 1999/10/09).

Morroy G, Peters JB, van Nieuwenhof M, Bor HH, Hautvast JL, van der Hoek W, et al. The health status of Q-fever patients after long-term follow- up. BMC Infectious Disease, 2011 Apr 18;11:97.

Q Fever Fact Sheet: Symptoms, Diagnosis, and Treatment – CDC, Atlanta. 2013. Available at: http://www.cdc.gov/qfever/symptoms/

Raoult D, Marrie T, Mege J. Natural history and pathophysiology of Q fever. Lancet Infectious Diseases, 2005 Apr;5(4):219-26.

Stouthard ME, Essink-Bot ML, Bonsel GJ, Barendregt JJ, Kramers PGN, van de Water HPA, et al. Disability Weights for Diseases in the Netherlands. Rotterdam, the Netherlands: Department of Public Health, Erasmus University Rotterdam, the Netherlands; 1997.

van der Hoek W, Meekelenkamp JC, Leenders AC, Wijers N, Notermans DW, Hukkelhoven CW. Antibodies against Coxiella burnetii and pregnancy outcome during the 2007-2008 Q fever outbreaks in The Netherlands. BMC Infectious Diseases, 2011 Feb 11;11:44.

Rabies

The initial symptoms of rabies resemble those of other systemic viral infections (Anderson, 1984). Two kinds of central nervous system (CNS) presentation can be seen: the furious form in 70% of all cases and the paralytic form in the remainder (WHO, 2013).

The furious form usually lasts around 12 days on average (range 9�17.8 days) (Udow, 2014). The paralytic form has a longer survival period of 22 days on average (range 18�28 days) and generally results in death.

Case fatality proportion

Once the symptomatic disease onset is confirmed the case fatality proportion is considered to be 100%.

Model input summary

Table 1. Transition probabilities used in the outcome tree

Health outcome<br>(Health state)	Distribution of health states in health outcome	Transition probability	Source/assumption
Symptomatic infection<br>(Furious form)<br>(Paralytic form)	 70%<br>30%		WHO, 2013
Fatal cases		100%	WHO, 2013

Table 2. Disability weights and duration

Health outcome<br>(Health state)	Disability Weight (DW) (Haagsma, 2015)		Duration	
	DW	Label	In years	Source
Symptomatic infection	0.655 (0.579-0.727)	Intensive Care Unit admission		

(Furious form)	As above	As above	0.033 (0.025-0.049)	Udow, 2014
(Paralytic form)	As above	As above	0.060 (0.049-0.077)	Udow, 2014

References

Anderson LJ, Nicholson KG, Tauxe RV, Winkler WG. Human rabies in the United States, 1960 to 1979: Epidemiology, diagnosis, and prevention. Ann Intern Med 1984;100:728-735.

Haagsma JA, Maertens de Noordhout C, Polinder S, Vos T, Havelaar AH, Cassini A, Devleesschauwer B, Kretzschmar ME, Speybroeck N, Salomon JA. Assessing disability weights based on the responses of 30,660 people from four European countries. Population Health Metrics 2015; 13: 10

Udow S, Marrie R, Jackson A. Clinical Features of Dog- and Bat-Acquired Rabies in Humans. Clinical Infectious Diseases Volume 57, Issue 5 Pp. 689-696.

WHO. Rabies. Fact Sheet N�99. Available at: http://www.who.int/mediacentre/factsheets/fs099/en/.

Rubella

Acquired rubella

Acquired, or non-congenital rubella usually gives rise to a mild rash and asymptomatic infections are common. The rash usually begins on the face and then progresses from head to foot. It lasts about three days and is occasionally pruritic (CDC, 2009). Since up to 50% of infections may not present with a rash, many cases are not detected or reported (CDC, 2009; Ang, 2010).

Risk of complications

The most relevant complications associated with rubella virus infection include arthritis or arthralgia, thrombocytopenia, and encephalitis (Zhou, 2004). Additional, but rare complications include orchitis, neuritis, bacterial superinfection, a late syndrome of progressive panencephalitis and mild hepatitis (CDC, 2009).

Arthritis/arthralgia

Arthralgia or arthritis may occur in 30–70% of adult women who contract rubella, but it is rare in children and adult males. It rarely develops into chronic arthritis (CDC, 2009; Mandell, 1999; Johnson, 1958). An age-independent range of 30–70% was estimated as the proportion of acute infections with this complication in the model, for females only. In 11 patients with rubella arthritis studied by Yanez et al. (Yanez, 1966), the onset of arthritis occurred one to six days after the beginning of the exanthem and lasted three to 28 days (mean of nine days).

Thrombocytopenic purpura

Hemorrhagic manifestations occur in approximately one case in 3 000 – more frequently in children than in adults – of which thrombocytopenic purpura is the most common (CDC, 2009; White, 1985; Mandell, 1999; Heggie, 1969; Boyer, 1965). Based on this estimated rate of occurrence (1/3 000), the proportion with the complication was estimated as 0.03% in the model.

Acute throbocytopenic purpura is commonly seen in children aged 1–7 years, and is defined as thrombocytopenia that lasts less than six months. In cases where thrombocytopenia persists for more than six months, it is considered chronic. Chronic thrombocytopenia occurs in a very small number of children (Taghizadeh, 2008).

Encephalitis

Encephalitis occurs in one in 5 000-6 000 cases, more frequently in adults (especially in females) than in children (CDC 2009; Mandell, 1999). Notwithstanding this occurrence rate, an age/sex-independent range of 0.01–0.02% was estimated for the proportion of acute cases with this complication in the model. The severity is highly variable. Symptoms in survivors usually resolve within 1–3 weeks without neurological sequelae (Gülen, 2008; Wolinsky, 1994).

Case fatality proportion

The case fatality proportion for thrombocytopenic purpura is 2.6% (Portielje, 2001). For encephalitis the overall lethality rate is 0–50% (CDC, 2009). Therefore in the model, the case fatality proportion following the health state thrombocytopenic purpura was specified with a point estimate of 4%, and the case fatality proportion following the health state encephalitis was set to the range of 20–50%.

Congenital rubella

Symptomatic infection occurs in 100% of infected foetuses between weeks 1 and 11. During weeks 11–20, symptomatic infection occurs in 30% of foetuses. After week 20 no foetus develops any manifestation of Congenital Rubella Syndrome (CRS) (Feigin, 2004). However, occasional foetal damage (deafness only) has been observed after the twentieth week (Mandell, 1999). Up to 50% of affected foetuses may appear healthy at birth and develop central nervous system abnormalities later (Duszak, 2009). Among children with CRS, 13% have one congenital defect, 24% have two defects and 63% have three or more defects (Reef, 2000).

We did not consider any loss of quality of life before birth and therefore the disability weight and duration for the symptomatic infection was set to 0.

Risk of sequelae

Hearing impairment occurs in 60% of children with CRS, heart disease in 45%, microcephaly in 27% (Reef, 2000), cataracts in 16–25% (Bloom, 2005), mental retardation in 13–25% (Lanzieri, 2004; Reef, 2000), and retinopathy in 5% (Reef, 2000). Overall, 20–40% of CRS survivors aged 35 or older have insulin-dependent diabetes (Mandell, 1999; Duszak, 2009) and 5% of survivors aged 13–19 develop some form of thyroid disease. (Duszak, 2009). Panencephalitis is a rare, fatal, late complication. The incidence of other late complications is still unknown (Duszak, 2009).

The case fatality ratio for infants with confirmed CRS is 10% (Reef, 2000).

Model input summary

Table 1. Transition probabilities used in the outcome tree

Health outcome<br><br>(Health state)	Distribution of health states in health outcome	Transition probability	Source/assumption

Acquired			
Symptomatic infection<br>(Arthritis/arthralgia)<br>(Thrombocytopenic purpura)<br>(Encephalitis) (Uncomplicated)	30–70%; females only<br>0.03%<br>0.01–0.02%<br>Remaining cases		CDC 2009, Mandell 1999, Johnson 1958<br>CDC 2009, White 1985<br>CDC 2009, Mandell 1999
Fatal cases following thrombocytopenic purpura		2.6%	Portielje, 2001
Fatal cases following encephalitis		0–50%	CDC, 2009
Congenital			
Permanent disability due to hearing impairment		60%	Reef, 2000
Permanent disability due to congenital heart defects		45%	Reef, 2000
Permanent disability due to microcephaly		27%	Reef, 2000
Permanent disability due to cataract		16–25%	Bloom, 2005
Permanent disability due to mental retardation		13–25%	Lanzieri, 2004; Reef, 2000
Permanent disability due to retinopathy		5%	Reef, 2000
Permanent disability due to insulin-dependent diabetes		20–40%	Mandell, 1999; Duszak, 2009 (aged >35 years)
Permanent disability due to thyroid gland dysfunction		5%	Duszak, 2009 (aged 13–19 years)
Fatal cases		10%	Reef, 2000

Table 2. Disability weights and duration

Health outcome<br>(Health state)	Disability Weight (DW) (Haagsma, 2015)	Duration

	DW	Label	In years	Source/assumption
Symptomatic infection<br>(Uncomplicated)<br>(Arthritis/arthralgia)<br>(Thrombocytopenic purpura)<br>(Encephalitis)	0.007 (0.005–0.01)<br>0.344 (0.3–0.391)<br>0.167 (0.134–0.201)<br>0.41 (0.358–0.47)	Infectious disease, acute episode, mild<br>Musculoskeletal problems, generalised,moderate<br>Thrombocytopenic purpura<br>Encephalopathy - moderate	0.008<br>0.008–0.077<br>0.008–0.5<br>0.019–0.058	CDC, 2009<br>CDC, 2009<br>Yanez, 1996<br>Taghizadeh, 2008 Gülen, 2008; Wolinsky, 1994/without any neurological sequelae
Congenital				
Symptomatic infection	0		0	
Permanent disability due to hearing impairment	0.008–0.103	From lowest to highest hearing loss related DWs	Remaining life expectancy	
Permanent disability due to congenital heart defects	0.052–0.173	From lowest to highest heart failure related DWs	Remaining life expectancy	
Permanent disability due to microcephaly	0.011–0.421		Remaining life expectancy	

		From lowest to highest cognitive difficulties related DWs		
Permanent disability due to cataract	0.004–0.171	From lowest to highest visual impairment related DWs	Remaining life expectancy	
Permanent disability due to mental retardation	0.011–0.421	From lowest to highest cognitive difficulties related DWs	Remaining life expectancy	
Permanent disability due to retinopathy	0.004–0.171	From lowest to highest visual impairment related DWs	Remaining life expectancy	
Latency period before diabetes	0		35	Mandell, 1999; Duszak, 2009
Latency period before thyroid dysfunction	0		13–19	Duszak, 2009
Permanent disability due to insulin-dependent diabetes	0.07 (0.057–0.088)	Generic uncomplicated disease: worry and daily medication	Remaining life expectancy	
Permanent disability due to thyroid gland dysfunction	0.07 (0.057–0.088)	Generic uncomplicated disease: worry and daily medication.	Remaining life expectancy	

References

Ang LW, Chua LT, James L, Goh KT. Epidemiological surveillance and control of rubella in Singapore, 1991-2007. Ann Acad Med Singapore. 2010 Feb;39(2):95-101.

Assaad F, Ljungars-Esteves K. Rubella-world impact. Rev Infect Dis. 1985 Mar-Apr;7 Suppl 1:S29-36.

Boyer WL, Sherman FE, Michaels RH, et al. Purpura in congenital and acquired rubella. N Engl J Med. 1965;273:1362.

Bloom S, Rguig A, Berraho A, Zniber L, Bouazzaoui N, Zaghloul Z, et al. Congenital rubella syndrome burden in Morocco: a rapid retrospective assessment. Lancet. 2005 Jan 8-14;365(9454):135-41.

Centers for Disease Control and Prevention. Epidemiology and Prevention of Vaccine-Preventable Diseases. Atkinson W, Wolfe S, Hamborsky J, McIntyre L, eds. 11th ed. Washington DC: Public Health Foundation, 2009.

Duszak RS. Congenital rubella syndrome--major review. Optometry. 2009 Jan;80(1):36-43.

Feigin RD. Textbook of pediatric infectious diseases, Volume 1. 2004. Elsevier Health Sciences.

Haagsma JA, Maertens de Noordhout C, Polinder S, Vos T, Havelaar AH, Cassini A, Devleesschauwer B, Kretzschmar ME, Speybroeck N, Salomon JA. Assessing disability weights based on the responses of 30,660 people from four European countries. Population Health Metrics 2015; 13: 10

Heggie AD, Robbins FC. Natural rubella acquired after birth. Am J Dis Child. 1969;118:12.

Gülen F, Cagliyan E, Aydinok Y, Ozen S, Yildiz B. A patient with rubella encephalitis and status epilepticus. Minerva Pediatr. 2008 Feb;60(1):141- 4.

Johnson RE, Hall AP. Rubella arthritis. N Engl J Med. 1958;258:743.

Lanzieri TM, Parise MS, Siqueira MM, Fortaleza BM, Segatto TC, Prevots DR. Incidence, clinical features and estimated costs of congenital rubella syndrome after a large rubella outbreak in Recife, Brazil, 1999-2000. Pediatr Infect Dis J. 2004 Dec;23(12):1116-22

Mandell GL, Bennet JE, Dolin R. Mandell, Douglas and Bennett’s Principles and Practice of Infectious Disease - 5th edition. Churchill Livingstone, 1999.

Portielje JE, Westendorp RG, Kluin-Nelemans HC, Brand A. Morbidity and mortality in adults with idiopathic thrombocytopenic purpura. Blood 2001 97:2549–2554.

Reef SE, Plotkin S, Cordero JF, Katz M, Cooper L, Schwartz B, et al. Preparing for elimination of Congenital Rubella Syndrome (CRS): summary of a workshop on CRS elimination in the United States. Clin Infect Dis. 2000 Jul;31(1):85-95

Sherman FE, Michaels RH, Kenny FM. Acute encephalopathy (encephalitis) complicating rubella. JAMA. 1965;192:675.

Steen E, Torp KH. Encephalitis and thrombocytopenic purpura after rubella. Arch Dis Child. 1956;31:470.

Taghizadeh M. An Update on Immune-Mediated Thrombocytopenia. Labmedicine. Volume 39, Number 1, January 2008.

White CC, Koplan JP, Orenstein WA. Benefits, risks and costs of immunization for measles, mumps and rubella. Am J Public Health. 1985 July; 75(7): 739–744.

Wolinsky JS. Rubella. In: Fields BM, Knipe DM, Chanock RM, et al., eds. Virology. 3rd ed. New York: Raven Press, 1994;899-929.

Yanez JE, Thompson GR, Mikkelsen WM, et al. Rubella arthritis. Ann Intern Med 64:772-777, 1966.

Zhou F, Reef S, Massoudi M, Papania MJ, Yusuf HR, Bardenheier B, et al. An Economic Analysis of the Current Universal 2-Dose Measles-Mumps- Rubella Vaccination Program in the United States. J Infect Dis. (2004) 189 Supplement 1: S131-S145.

Salmonellosis

Acute gastroenteritis associated with Salmonella infections in humans is, in most cases, self-limiting within a few days or weeks, but for some patients the disease is fatal. Studies estimated the duration to be 5.58 days for gastroenteritis cases not requiring medical help, 10.65 days for gastroenteritis cases visiting a doctor but not hospitalised and 16.15 days for hospitalised gastroenteritis cases (Kemmeren 2006).

The proportion of mild (uncomplicated), moderate (complicated, doctor) and severe (complicated, doctor) symptomatic infections is set at 83.3%, 15% and 1.7% (Kemmeren 2006; Kwong 2012; redistributing in order to total 100%)

In many reports bacteraemia is highlighted as a possible extra-intestinal complication of salmonellosis (0.03% of laboratory-confirmed cases, Ternhag 2008), although these complicated cases are often considered within the hospitalised proportion of cases (Cressey & Lake 2007; Kemmeren 2006).

The case fatality proportion for symptomatic salmonellosis cases ranged from 0.1% (Kemmeren 2006; Helms 2003) to 0.05% in salmonellosis outbreaks in Austria (Much 2005) and 0.3 for non-typhoid infections in England and Wales (Adak, 2002). These were in line with case fatality proportions observed in cases reported to TESSy between 2009 and 2013 (personal communication).

We chose to estimate the overall case fatality proportion as being within the range 0.05–0.1% and assumed a different age-group distribution of this risk, based on the age-group distribution of deaths reported to TESSy between 2009 and 2013 (see Table 3). This table is based on all TESSy notified cases from EU Member States except Bulgaria, Latvia and Poland which report only aggregate data, and Italy because the outcome was not reported.

Risk of complications

Reactive arthritis (ReA) and Irritable Bowel Syndrome (IBS) are the most frequent sequelae of salmonellosis reported in the literature (Haagsma 2009; Raybourne 2003). The frequency of other post-infectious complications following salmonellosis is extremely low and these were disregarded in the current study.

Reactive arthritis (ReA)

Many studies reported ReA as sequelae of salmonellosis (Keat 1983; Fendler 2001; Raybourne, 2003). A review of the literature, which included mostly cases of salmonellosis occurring during outbreaks, estimated that 8% (2.3–15%) of cases are at risk of developing ReA (Raybourne, 2003), although most of these studies have estimated risk based on laboratory-confirmed cases and duration of diarrhoea is highly correlated with the development of ReA (Yu & Thomson, 1994). In order to account for the considerable uncertainty, the risk of developing ReA from all symptomatic cases is set at 1.31% (0.29–5.43%) (Kemmeren, 2006).

Little is known about the duration of ReA; the average duration is set at between 1.5 months, derived from Hannu et al. (Hannu, 2005) and 222 days (Kemmeren, 2006).

Irritable bowel syndrome (IBS)

In a recent literature review, 8.8% (7.2–10.4%) of symptomatic salmonellosis cases were considered at risk of developing IBS, irrespective of age and gender; the duration was set to five years (Haagsma, 2010). However, the causality is largely debated and the impact of concurrent factors significant. Therefore, IBS is not considered as part of the salmonellosis outcome tree in our study.

Model input summary

Table 1. Transition probabilities and distributions used in the outcome tree

Health outcome (Health state)	Distribution of health states in health outcome	Transition probability	Source/assumption
Symptomatic infection:<br>(Uncomplicated)<br>(Complicated, doctor)<br>(Complicated, hospital)	83.3%<br>15%<br>1.7%		Kemmeren, 2006; Kwong, 2012
Fatal cases following symptomatic infection		0.05–0.1%<br>Age dep. Table 3	Kemmeren, 2006; Much, 2005; TESSy 2009-2013
Reactive arthritis		1.31% (0.29-5.43%)	Kemmeren, 2006

Table 2. Disability weights and duration

Health outcome (Health state)	Disability Weight (DW) (Haagsma, 2015)		Duration
	DW	Label	In years

				Source
Symptomatic infection	0.073 (0.061–0.092)	Diarrhoea, mild	0.015	Kemmeren, 2006
(Uncomplicated)	0.149 (0.12–0.182)	Diarrhoea, moderate	0.029	
(Complicated, doctor)	0.239 (0.202–0.285)	Diarrhoea, severe	0.044	
(Complicated, hospital)				
Reactive arthritis	0.344 (0.3–0.391)	Musculoskeletal problems, generalised, moderate	0.131–0.608	Hannu, 2002

Table 3. Age-group redistribution of CFR (0.05–0.1%)

Age groups	%
0	0.69
1–4	1.72
5–9	1.38
10–14	0.34
15–19	1.03
20–24	0.00
25–29	1.72
30–34	0.34
35–39	1.03
40–44	0.69
45–49	2.07

50–54	3.45
55–59	4.14
60–64	5.17
65–69	9.31
70–74	12.41
75–79	16.55
80–84	18.62
>85	19.31
All ages	100.00

References

Adak GK, Long SM, O'Brien SJ. Trends in indigenous foodborne disease and deaths, England and Wales: 1992 to 2000. Gut. 2002 Dec;51(6):832- 41.

Cressey PJ and Lake R (2007). Risk ranking: Estimates of the burden of foodborne disease for New Zealand. Institute of Environmental Science & Research Limited; Christchurch Science Center.

Fendler CS, Laitko HH, Sorensen C, Gripenberg-Lerche A, Groh J, Uksila K et al. Frequency of triggering bacteria in patients with reactive arthritis and undifferentiated oligoarthritis and the relative importance of the tests used for diagnosis. Ann Rheum Dis 2001;60(4): 337-43.

Food Standards Agency (2000). A Report of the Study of Infectious Intestinal Disease in England. Food Standards Agency London: HMSO.

Haagsma JA, Havelaar AH, Janssen BM, Bonsel GJ. Disability Adjusted Life Years and minimal disease: application of a preference-based relevance criterion to rank enteric pathogens. Popul Health Metr 2008;6:7.

Haagsma JA, van der Zanden BP, Tariq L, van Pelt W, van Duynhoven YTHP, Havelaar AH. Disease burden and costs of selected foodborne pathogens in the Netherlands, 2006. Bilthoven: National Institute of Public Health and Environment. 2009. RIVM rapport 330331001. Available online at: www.rivm.nl/bibliotheek/rapporten/330331001.pdf

Haagsma JA, Siersema PD, De Wit NJ, Havelaar AH. Disease burden of post-infectious irritable bowel syndrome in the Netherlands. Epidemiol Infect 2010;138(11):1650-6.

Haagsma JA, Maertens de Noordhout C, Polinder S, Vos T, Havelaar AH, Cassini A, Devleesschauwer B, Kretzschmar ME, Speybroeck N, Salomon JA. Assessing disability weights based on the responses of 30,660 people from four European countries. Population Health Metrics 2015; 13: 10

Hannu TL, Mattila H, Rautelin P, Pelkonen P, Lahdenne A, Siitonen M et al. Campylobacter-triggered reactive arthritis: a population-based study. Rheumatology 2002;41(3): 312-318.

Havelaar AH, de Wit MAS, van Koningsveld R, van Kempen E (2000a). Health burden in the Netherlands due to infection with thermophilic Campylobacter spp. Epidemiol Infect 2000;125(3): 505-22.

Havelaar AH, de Wit MAS, van Koningsveld R (2000b). Health burden in the Netherlands (1990–1995) due to infection with thermophilic Campylobacter species. Bilthoven, the Netherlands: National Institute for Public Health and the Environment. Report no. 284550004.

Havelaar AH, Ivarsson S, Löfdahl M, Nauta MJ. Estimating the true incidence of campylobacteriosis and salmonellosis in the EU. 2012. [Submitted for publication].

Helms M, Vastrup P, Gerner-Smidt P, Molbak K. Short and long term mortality associated with foodborne bacterial gastrointestinal infections: registry based study. BMJ 2003; 326(7385): 357-60.

Keat A. Reiter's syndrome and reactive arthritis in perspective. N Engl J Med 1983;309(26): 1606-15.

Kemmeren JM, Mangen MJJ, van Duynhoven YTHP, Havelaar AH. Priority setting of foodborne pathogens: disease burden and costs of selected enteric pathogens. Bilthoven: National Institute of Public Health and Environment: RIVM rapport 330080001. 2006. Available online at: www.rivm.nl/bibliotheek/rapporten/330080001.pdf

Mangen MJJ, Havelaar AH, de Wit GA. Campylobacteriosis and sequelae in the Netherlands - Estimating the disease burden and the cost-of- illness. Bilthoven: National Institute for Public Health and the Environment; Report no. 250911004. 2004. Available online at: www.rivm.nl/bibliotheek/rapporten/250911004.pdf

Mangen MJJ, Havelaar AH, Bernsen RAJAM, van Koningsveld R, de Wit GA. The costs of human Campylobacter infections and sequelae in the Netherlands: A DALY and cost-of-illness approach. Acta Agriculturae Scandinavica Section C Food Economics 2005;(1): 35-51.

Melse JM and Kramers PGN (1998). Berekening van de ziektelast in Nederland. Achtergronddocument bij VTV-1997; deel III, hoofdstuk 7. Bilthoven; Report no. 431501028.

Much P, Pichler J, Allerberg F. Food borne infectious outbreaks, Austria 2005." Wien Klin Wochenschr 2007;119 (5-6):150-7.

Raybourne RB, Williams KM, Roberts T and Arthritis Working Group. Food Poisoning: Economic Implications. In: Caballero BL, Trugo P, Finglas (Editors). Encylopaedia of Food Sciences and Nutrition. 10 volume set edition. London: 2003; 2672-82.

Robert Koch Institut. Infektionsepidemiologisches Jahrbuch (various years). Available online at:

www.rki.de/cln_178/nn_205772/DE/Content/Infekt/Jahrbuch/jahrbuch__node.html?__nnn=true

Ternhag A, Törner A, Svensson A, Ekdahl K, Giesecke J. Short- and long-term effects of bacterial gastrointestinal infections. Emerg Infect Dis 2008;14 (1):143-8.

WHO. Global Burden of Disease 2004 Update: Disability weights for diseases and conditions. Geneva: 2004, WHO. Available online at: http://www.who.int/healthinfo/global_burden_disease/GBD2004_DisabilityWeights.pdf

Yu DTY and Thomson GTD. Clinical, epidemiological and pathogenic aspects of reactive arthritis. Food-Microbiology 1994;11(2): 97-108.

Shigellosis

Acute gastroenteritis associated with Shigella spp. infections in humans is, in most cases, self-limiting within days to weeks, but for a few patients the disease may be severe and fatal.

We assume that more complicated cases visit their doctor or are hospitalised and will subsequently be laboratory-tested and reported as confirmed. The proportion of reported cases over the total symptomatic cases is 5.45% (2.18–40%) (Haagsma, 2010).

We assumed a similar duration of symptoms as for salmonellosis: 5.58 days for uncomplicated cases and 10.65–16.15 for complicated ones (Kemmeren, 2006).

On average, patients aged 65 years and over are hospitalised for a greater number of days and are more likely to die of shigellosis than other patients (van Pelt, 2010; Barton Behravesh, 2011). We assumed that only complicated cases lead to fatalities and set the case fatality proportion for complicated cases as 0.06–0.97% (Van Pelt, 2010; Barton Behravesh, 2011). Assuming a different age-group distribution of this risk, we distributed the case fatality proportion based on the age-group distribution of deaths reported to TESSy between 2009 and 2013 (see Table 3). This table is based on all TESSy-notified cases from EU Member States except Bulgaria, Lithuania and Poland, because they report only aggregate data, and Liechtenstein, Luxembourg and Italy which do not report on the death outcome.

Risk of complications

Reactive arthritis (ReA), Post-Infectious Irritable Bowel Syndrome (PI-IBS), Haemolytic Uraemic Syndrome (HUS) and End-stage Renal Disease (ESRD) are possible sequelae of shigellosis.

Asymptomatic cases, which themselves do not have a disease burden for acute illness, might also develop sequelae. However neither the number of asymptomatic cases in the population, nor the percentage of asymptomatic cases that develop sequelae is known and these are therefore not included in the model.

Reactive arthritis (ReA)

The risk of developing ReA has been found to be 6.6% of all laboratory-confirmed cases of shigellosis (Hannu, 2005), 1.2% (Rees, 2004) and 9.8% (Schiellerup, 2008). However, severity of the acute infection and duration of diarrhoea are associated with a higher risk of developing ReA (Townes, 2008; Hannu, 2005; Rees, 2004; Schiellerup, 2008); moreover, these figures relate to laboratory-confirmed cases only. Therefore, we assume that only ‘complicated’ cases have a risk of 6.6% (1.2-9.8%) of developing ReA.

Little is known about the duration of ReA; the average duration is set between 1.5 months (Hannu, 2005) and 222 days (Kemmeren, 2006).

Irritable bowel syndrome (IBS)

In a recent literature review, 8.8% (7.2–10.4%) of symptomatic infections involving foodborne pathogens (salmonellosis, campylobacteriosis and shigellosis) were associated with a risk of developing IBS, irrespective of age and gender. The duration of IBS was set to five years (Haagsma, 2010). However, the causality is largely debated and the impact of concurrent factors significant. Therefore, IBS is not considered as part of the shigellosis outcome tree in our study.

Haemolytic uraemic syndrome (HUS)

>HUS is characterised by haemolytic anaemia (severe anaemia due to increased destruction of red blood cells), thrombocytopenia (reduced platelet count) and impaired kidney function (acute renal failure). Haemolytic anaemia and thrombocytopenia often occur after bloody diarrhoea. Acute renal failure may then follow.

Several studies have associated HUS with shigellosis infections, in particular Shigella dysenteriae type 1, a species which occurs mainly in tropical countries and accounts for approximately 30% of S. dysenteriae isolates in those countries (Mark Taylor, 2008; Chopra, 1997; Bennis, 2006; Kotloff, 1999; Ekdahl, 2005).

In Europe, based on data reported to TESSy, S. dysenteriae accounts for less than 3% of laboratory-confirmed shigellosis cases, whereas S. sonnei is the most common Shigella species (ECDC, 2013 a & b). This means that around 0.9% of the shigellosis cases occurring in Europe, caused by Shigella dysenteriae type 1, are at risk of developing HUS; however, the risk varies according to EU Member State.

The incidence of S. dysenteriae-induced HUS is unknown and it is affected by antibiotic treatment (Bennish, 2006). HUS caused by S. dysenteriae type 1 is often perceived as more severe than HUS caused by enterohaemorrhagic E. coli (EHEC), however this is probably due to the fact that such infections mainly occur in countries with limited access to high-quality healthcare. Though the age range of Shigella-induced- HUS is wider and the 'median time from the onset of diarrhoea to the presentation of HUS' is longer, HUS caused by Shigella and EHEC is very similar (Mark Taylor, 2008). Therefore, we assume that the risk of developing HUS after symptomatic infection with Shigella dysenteriae type 1 is the same as the risk for symptomatic infections with Shiga-toxin producing E. coli O157 (STEC), around 0.94–1.25% (Cressey & Lake, 2007).

Given that 0.9% of shigellosis cases occurring in Europe are caused by Shigella dysenteriae type 1, the overall risk of developing HUS after symptomatic shigellosis is set to 0.008–0.011%.

HUS occurs mainly in children aged one to five years, and less frequently in children over five years. In one study (Havelaar, 2003) 72% of all HUS cases were under 15 years of age, and 28% were older. The distribution of HUS patients admitted to the Paediatric Nephrology Department of University Hospital Nijmegen from 1974–1993 was used for cases under 15 years (Havelaar, 2003). For the current study we distributed the age risk of developing HUS (0.008-0.011%) according to TESSy-notified cases of HUS by age due to VTEC infection from 2009 to 2013 (see Table 4). Cases were from all EU Member States except Bulgaria and Lithuania, because they report only aggregate data, and Italy because it has sentinel surveillance for STEC/VTEC which does not provide sufficient coverage.

Duration of HUS is reported to be seven days (range 3–31 days) and 41% (19/46) of patients were admitted to hospital (McPherson, 2011). Hospitalisation is reported to last 2–4 weeks for HUS patients (Havelaar, 2003).

The case fatality proportion is assumed to be 3.7% (Cressey & Lake, 2007; Oxford Medical Dictionary, 2003; Havelaar, 2003). This low case- fatality might be valid for cases up to 65 years and be as high as 56% for those aged ≥65 years as data from an outbreak in Scotland suggests (Dundas, 1999). For the current study we use age-specific fatality proportions as reported by Havelaar et al. (Havelaar, 2003; see Table 5).

End-stage renal disease (ESRD)

ESRD is one of the most serious outcomes associated with HUS and is the most advanced stage of kidney failure (Oxford Medical Dictionary, 2003). HUS cases may develop ESRD briefly after HUS or after a long latency period. In the current study we assume that 13.4% develop ESRD, of which 2.9% briefly after HUS and 10.5% after a latency period of 20 years (Havelaar, 2004; Cressey & Lake, 2007). We also assume that all cases experiencing ESRD are in dialysis treatment until transplantation occurs.

The case-fatality proportion is based on the assumption that in the first year after starting dialysis mortality ratios are relatively high and differ between age-groups (see Table 6) and that only few fatalities occur after renal transplantation (Havelaar, 2003). Duration of dialysis (time to transplantation) is age-dependent and is applied to the duration of ESRD, see Table 7 (Havelaar, 2003).

Model input summary

Table 1. Transition probabilities used in the outcome tree

Health outcome<br>(Health state)	Distribution of health states in health outcome	Transition probability	Source/assumption
Symptomatic infection<br>(Uncomplicated)<br>(Complicated)	Rem. cases<br>5.45% (2.18–40%)		Haagsma, 2010
Fatal cases following complicated symptomatic infection		0.06–0.97<br>Age dep.Table 3	Van Pelt, 2010; Barton Behravesh, 2011; TESSy 2009–2013
ReA		6.6% (1.2–9.8%)	Hannu, 2005; Rees, 2004; Townes, 2008; Schiellerup, 2008
HUS		0.008–0.011%<br>Age dep.<br>Table 4	Mark Taylor, 2008; Chopra, 1997; Bennis, 2006; Kotloff, 1999; Ekdahl, 2005; ECDC, 2013 a & b; Cressey & Lake, 2007
Latency period before ESRD		10.5%	Havelaar, 2004; Cressey & Lake, 2007
ESRD after HUS		2.9%	Havelaar, 2004; Cressey and Lake, 2007

ESRD after latency period		100%	
Fatal cases following HUS		< 65 years: 3.7%<br>>=65 years: 56%<br>Table 5	Haavelar, 2004; Dundas, 1999
Fatal cases following ESRD		Age dep. & different for dialysis and transplantation<br>See Table 6.	Havelaar, 2003<br>see Table 6
Transplanted		Remaining %	

Table 2. Disability weights and duration

Health outcome (Health state)	Disability Weight (DW) (Haagsma, 2015)		Duration	
	DW	Label	In years	Source/assumption
Symptomatic infection<br>(Uncomplicated)<br>(Complicated)	0.073–0.149<br>0.239 (0.202–0.285)	Diarrhoea, from mild to moderate<br>Diarrhoea, severe	0.015<br>0.029–0.044	Kemmeren, 2006
ReA	0.344 (0.3–0.391)	Musculoskeletal problems, generalised, moderate	0.131-0.608	Estimated from Hannu, 2005; Kemmeren, 2006
HUS	0.108 (0.09–0.132)	Chronic kidney disease (stage IV)	0.019 (0.008-0.085)	McPherson, 2011
ESRD	0.487 (0.432–0.544)	End-stage renal disease, on dialysis	See Table 7	Assuming that all ESRD are in dialysis

Transplanted	0.070 (0.057–0.088)	Generic uncomplicated disease: worry and daily medication	Remaining life expectancy	Assuming no risk of re-transplantation

Table 3. Age group distribution of the case fatality proportion (0.06–0.97%)

Age groups	%
0	0.00
1–4	10.00
5–9	10.00
10–14	0.00
15–19	0.00
20–24	0.00
25–29	0.00
30–34	10.00
35–39	0.00
40–44	10.00
45–49	20.00
50–54	0.00
55–59	0.00
60–64	10.00
65–69	0.00
70–74	0.00
75–79	10.00
80–84	10.00
>85	10.00
All ages	100.00

Table 4. Age-group redistribution of risk of developing HUS (0.008–0.011%) following infection (TESSy 2009– 2013)

Age groups	%
0	5.67
1–4	33.74
5–9	13.09
10–14	6.62

15–19	2.88
20–24	2.27
25–29	3.83
30–34	3.54

35–39	2.88
40–44	3.40
45–49	3.45
50–54	2.36
55–59	2.88
60–64	3.02
65–69	2.27
70–74	3.36
75–79	1.89
80–84	1.65
85+	0.99
All ages	100

Table 5. HUS case-fatality proportion per age group

Age groups	CFR
0–65	3.7%
>65	56%

Table 6. Case-fatality proportions in the first year after starting dialysis and after renal transplantation

Age class	Case-fatality ratio dialysis	Case-fatality ratio renal transplantation
0–14	4.1% (0.9–11.1%)	7% (2.2–16%)
15–44	8.7% (5.8–12.4%)	7% (2.2–16%)
45–64	37% (31–44%)	7% (2.2–16%)
65–74	65% (58–72%)	7% (2.2–16%)
75+	79% (70–87%)	7% (2.2–16%)

Table 7. Age-specific duration of dialysis

Age class	Duration of dialysis
0–14	1.7 (0.2–5.3)
15–44	2.5 (0.2–9.6)
45–64	6.7 (0.5–30)
>65	5 to remaining life expectancy

References

Barton Behravesh C, Jones TF, Vugia DJ, Long C, Marcus R, Smith K et al. Deaths associated with bacterial pathogens transmitted commonly through food: foodborne diseases active surveillance network (FoodNet), 1996-2005. J Infect Dis, 2011. 204(2):263-7.

Bennish ML, Khan WA, Begum M, Bridges EA, Ahmed S, Saha D, et al. Low risk of hemolytic uremic syndrome after early effective antimicrobial therapy for Shigella dysenteriae type 1 infection in Bangladesh. Clin Infect Dis, 2006. 42(3):356-62.

Chopra, M., D. Wilkinson, and S. Stirling, Epidemic shigella dysentery in children in northern KwaZulu-Natal. S Afr Med J, 1997. 87(1):48-51.

Cressey PJ, Lake R. Risk ranking: Estimates of the burden of foodborne disease for New Zealand, 2007, Institute of Environmental Science & Research Limited; Christchurch Science Center: Christchurch.

Dundas S, et al. Effectiveness of therapeutic plasma exchange in the 1996 Lanarkshire Escherichia coli O157:H7 outbreak. Lancet, 1999. 354(9187):1327-30.

Ekdahl, K. and Y. Andersson, The epidemiology of travel-associated shigellosis--regional risks, seasonality and serogroups. Journal of Infection, 2005. 51(3):222-229.

European Centre for Disease Prevention and Control. Annual Epidemiological Report 2012. Reporting on 2010 surveillance data and 2011 epidemic intelligence data. Stockholm: ECDC; 2013.

European Centre for Disease Prevention and Control. Annual Epidemiological Report 2013. Reporting on 2011 surveillance data and 2012 epidemic intelligence data. Stockholm: ECDC; 2013.

Haagsma JA, Siersema PD, De Wit NJ, Havelaar AH. Disease burden of post-infectious irritable bowel syndrome in the Netherlands. Epidemiol Infect, 2010. 138(11):1650-6.

Haagsma JA, Maertens de Noordhout C, Polinder S, Vos T, Havelaar AH, Cassini A, Devleesschauwer B, Kretzschmar ME, Speybroeck N, Salomon JA. Assessing disability weights based on the responses of 30,660 people from four European countries. Population Health Metrics 2015; 13: 10

Hannu T, Mattila L, Siitonen A, Leirisalo-Repo M. Reactive arthritis attributable to Shigella infection: a clinical and epidemiological nationwide study. Annals of the rheumatic diseases, 2005. 64(4):594.

Havelaar AH, Van Duynhoven YT, Nauta MJ, Bouwknegt M, Heuvelink AE, De Wit GA, et al. Disease burden in the Netherlands due to infections with Shiga toxin-producing Escherichia coli O157. Epidemiol Infect, 2004. 132(3):467-84.

Havelaar AH, Mangen MJJ, van Duynhoven YTHP, Havelaar, AH. Disease burden in the Netherlands due to infections with Shiga toxin-producing Escherichia coli O157., in RIVM report 284550008/20032003, National Institute of Public Health and the Environment: Bilthoven, 2003.

Kemmeren J, Mangen MJJ, van Duynhoven YTHP, Havelaar AH. Priority setting of foodborne pathogens: disease burden and costs of selected enteric pathogens. RIVM report. National Institute of Public Health and the Environment: Bilthoven, 2006.

2006. Available at: http://www.rivm.nl/bibliotheek/rapporten/330080001.pdf.

Mark Taylor C. Enterohaemorrhagic Escherichia coli and Shigella dysenteriae type 1-induced haemolytic uraemic syndrome. Pediatric Nephrology, 2008. 23(9):1425-1431.

McPherson M, et al. Economic costs of Shiga toxin-producing Escherichia coli infection in Australia. Foodborne Pathogen Diseases, 2011. 8(1):55-62.

Rees JR, et al., Persistent diarrhea, arthritis, and other complications of enteric infections: a pilot survey based on California FoodNet surveillance, 1998-1999. Clin Infect Dis, 2004. 38 Suppl 3: S311-7.

Schiellerup P, Krogfelt KA, Locht H. A comparison of self-reported joint symptoms following infection with different enteric pathogens: effect of HLA-B27. J Rheumatol, 2008. 35(3): p. 480-7.

Tariq L, Haagsma J, Havelaar A. Cost of Illness and Disease Burden in The Netherlands Due to Infections with Shiga ToxinProducing Escherichia coli O157. Journal of Food Protection&# 174;, 2011. 74(4):545-552.

Townes JM, et al. Reactive arthritis following culture-confirmed infections with bacterial enteric pathogens in Minnesota and Oregon: a population-based study. Ann Rheum Dis, 2008. 67(12): 1689-96.

Van Pelt W, et al. Trends in Gastro-enteritis in Nederland; notitie met betrekking tot 2009 en 2010, in RIVM report project V/210221 December 2010, RIVM: Bilthoven.

STEC/VTEC

The current disease model relies strongly on publications focused around STEC/VTEC O157 infections. Shiga toxin-producing Escherichia coli O157 (STEC/VTEC O157) infection may be asymptomatic, or may result in acute gastroenteritis (GE), and potentially in haemorrhagic colitis: 44.5% of cases had bloody diarrhoea (Michel, 2000). Duration is assumed to be longer than for non-bloody diarrhoea (Havelaar, 2004): median duration of five days and three days for bloody and non-bloody diarrhoea respectively (Cressey & Lake, 2007), which are proposed in the model as a uniform distribution.

There is little information on STEC/VTEC-associated mortality. Study findings range from 0.083% of the total estimated/VTEC O157:H7 (Mead, 1999), 0.03% (Buzby & Roberts, 2009), 0.04% (Walkerton outbreak, one fatal case in 2 321 patients, Bruce-Grey-Owen Sound Health Unit, 2000) and 0.045 (Havelaar, 2004). We therefore assume a uniformly distributed case-fatality proportion of between 0.03% and 0.045% for this study.

Fatal cases occur mainly in elderly people (Bauch, 2007); therefore, we assumed that the case fatality proportion of 0.03–0.045% is distributed across age-groups in accordance with the observed age-group distribution of TESSy-reported deaths between 2009 and 2013 (see Table 3). This table is based on all TESSy-notified cases from EU Member States except Bulgaria and Lithuania, because they report only aggregate data, and Italy because it has sentinel surveillance for STEC/VTEC for which we do not have the coverage.

Risk of complications

STEC/VTEC infection has been associated with post-diarrhoeal haemolytic uremic syndrome (HUS), which may result in death, end-stage renal disease (ESRD) or other sequelae. HUS and ESRD are the most frequently occurring sequelae of STEC and will be considered in the outcome tree. Irritable Bowel Syndrome (IBS) is another frequently occurring sequelae of bacteria-triggered gastroenteritis (Haagsma, 2010; Marshall, 2010; Thabane, 2009) and was considered for inclusion in the outcome tree (see below). The frequency of other post-infectious complications following STEC is low and they were therefore disregarded (Havelaar, 2004; Frenzen, 2005; Cressey & Lake, 2007; Buzby, 2009; McPherson, 2011; Tariq, 2011).

Haemolytic uraemic syndrome (HUS)

Haemolytic Uraemic Syndrome (HUS) is 'a condition in which sudden rapid destruction of red blood cells causes acute renal failure' (Oxford Medical Dictionary, 2003). HUS may occur following a respiratory or gastrointestinal infection, especially by pathogenic Escherichia coli or

Shigella spp.

The risk of developing HUS after STEC/VTEC infection has been found to be 3–7% (McPherson, 2011), 1% (Havelaar, 2004), 0.94–1.25% (Cressey & Lake, 2007) and 1.6% of laboratory-confirmed EHEC infections although authors mention under-estimation due to misclassification (13/820; Ternhag, 2008). In the current study we assume that the probability of developing HUS after a VTEC/STEC symptomatic infection is 0.94–1.25%.

HUS occurs mainly in children between the ages of one and five years, and less frequently in children over five years. In one study, 72% of all HUS cases were under 15 years of age and 28% were older (Havelaar, 2003). Member States report HUS outcomes relating to STEC/VTEC infections and we therefore redistributed the age-group risk of developing HUS (0.94–1.24%) based on the age-group of HUS cases reported to TESSy between 2009 and 2013 (all Member States except Bulgaria, Italy and Lithuania) (see Table 4).

Duration is reported to be seven days (range 3–31 days) and 41% (19/46) of patients were admitted to hospital (McPherson, 2011); hospitalisation is reported to last two to four weeks for HUS patients (Havelaar, 2003).

The case fatality proportion was found to be 3.7% (Cressey & Lake, 2007; Oxford Medical Dictionary, 2003; Havelaar, 2003). This low case- fatality may be valid for cases up to 65 years and then as high as 56% for cases ≥65 years, as indicated by data from an outbreak in Scotland (Dundas, 1999). Other studies assume age-specific fatality rates, as reported by Havelaar et al. (Havelaar, 2003). We estimated the age-group case fatality proportion from HUS based on STEC/VTEC infections notified to TESSy between 2009 and 2013 from all Member States, except Bulgaria, Italy and Lithuania (see Table 5).

End-stage renal disease (ESRD)

ESRD is one of the most serious outcomes associated with HUS and is the most advanced stage of kidney failure (Oxford Medical Dictionary, 2003). HUS cases may develop ESRD briefly after HUS or after a long latency period. In the current study we assume that 13.4% develop ESRD, 2.9% of whom develop it briefly after HUS and 10.5% after a latency period of 20 years (Havelaar, 2004; Cressey & Lake, 2007). We also assume that all cases experiencing ESRD undergo dialysis treatment until transplantation occurs.

The case-fatality proportion is based on the assumption that in the first year after starting dialysis mortality is relatively high and differs among age-groups (see Table 6) and that only few fatalities occur after renal transplantation (Havelaar, 2003). Duration of dialysis (time to transplantation) is age-dependent and is applied to the duration of ESRD – see Table 7 (Havelaar, 2003).

Irritable bowel syndrome (IBS)

In a recent literature review, 8.8% (7.2-10.4%) of symptomatic infections with foodborne pathogens were considered at risk of developing IBS, irrespective of age and gender; the duration was set to 5 years (Haagsma, 2010). However, the causality is largely debated and the impact of concurrent factors significant. Therefore, IBS is not considered as part of the STEC/VTEC outcome tree in our study.

Model input summary

Table 1. Transition probabilities and distributions used in the outcome tree

Health outcome (Health state)	Distribution of health states in health outcome	Transition probability	Source/assumption
Fatal cases following symptomatic infection		0.03-0.045% Age-dependent (Table 3)	Buzby & Roberts, 2009; TESSy 2009-2013
Haemolytic uraemic syndrome (HUS)		0.94-1.25% Age-dependent (Table 4)	Havelaar, 2004; Cressey and Lake, 2007; TESSy 2009-2013
Latency period before ESRD		10.5%	Havelaar, 2004; Cressey and Lake, 2007
ESRD after HUS		2.9%	Havelaar, 2004; Cressey and Lake, 2007
ESRD after latency period		100%	
Fatal cases following HUS		Age-dependent (Table 5)	TESSy 2009-2013
Fatal cases following ESRD		Age-dependent, different for dialysis and transplantation (Table 6)	Havelaar, 2003 see Table 6
Transplanted		Remaining %	

Table 2. Disability weights and duration

Health outcome (Health state)	Disability Weight (DW) (Haagsma, 2015)		Duration	
	DW	Label	In years	Source
Symptomatic infection (Gastroenteritis)	0.149 (0.12-0.182)	Diarrhoea, moderate	0.008-0.014	Havelaar, 2004; Cressey & Lake, 2007
HUS	0.108 (0.09–0.132)	Chronic kidney disease (stage IV)	0.019 (0.008–0.085)	McPherson, 2011
ESRD	0.487 (0.432–0.544)	End-stage renal disease, on dialysis	Age dependent(See Table 7)	Assuming that all ESRD are in dialysis
Transplanted	0.070 (0.057–0.088)	Generic uncomplicated disease: worry and daily medication	Remaining life expectancy	

Table 3. Age-group redistribution of case fatality proportion (0.03–0.045%)

Age groups	%
0	4.30
1-4	9.68
5-9	4.30
10-14	0.00
15-19	0.00
20-24	2.15
25-29	0.00
30-34	0.00
35-39	3.23
40-44	3.23
45-49	2.15
50-54	1.08
55-59	4.30

60-64	8.60
65-69	4.30
70-74	10.75
75-79	10.75
80-84	15.05
>85	16.13
All ages	100.00

Table 4. Age-group redistribution of risk of developing haemolytic uraemic syndrome (0.94–1.25%)

Age	%
0	5.67
1-4	33.74
5-9	13.09
10-14	6.62
15-19	2.88
20-24	2.27
25-29	3.83
30-34	3.54
35-39	2.88
40-44	3.40
45-49	3.45
50-54	2.36
55-59	2.88
60-64	3.02
65-69	2.27
70-74	3.36
75-79	1.89
80-84	1.65
85+	0.99
All ages	100

Table 5. Age-group case fatality proportion from haemolytic uraemic syndrome (TESSy 2009–2013)

Age	%

0	6.06
1-4	2.63
5-9	3.25
10-14	0.00

15-19	0.00
20-24	5.13
25-29	0.00
30-34	0.00
35-39	3.64
40-44	3.28
45-49	3.17
50-54	2.13
55-59	2.00
60-64	4.44
65-69	8.33
70-74	4.62
75-79	17.86
80-84	25.93
85+	28.57
All ages	3.91

Table 6. Case-fatality proportions in the first year after starting dialysis and after renal transplantation

Age class	Case-fatality ratio dialysis	Case-fatality ratio renal transplantation
0–14	4.1% (0.9–11.1%)	7% (2.2–16%)
15–44	8.7% (5.8–12.4%)	7% (2.2–16%)
45–64	37% (31–44%)	7% (2.2–16%)
65–74	65% (58–72%)	7% (2.2–16%)
75+	79% (70–87%)	7% (2.2–16%)

Table 7 .Age specific duration of dialysis

Age class	Duration of dialysis
0-14	1.7 (0.2-5.3)
15-44	2.5 (0.2-9.6)
45-64	6.7 (0.5-30)

>65	5 years to remaining life expectancy

References

Bauch CT, Anonychuk AM, Pham BZ, Gilca V, Duval B, Krahn MD (2007). Cost-utility of universal hepatitis A vaccination in Canada. Vaccine 25(51): 8536-48.

Buzby JC, Roberts T (2009). The economics of enteric infections: human foodborne disease costs. Gastroenterology 136: 1851-1862.

Bruce-Grey-Owen Sound Health Unit (2000). The investigative report of the Walkerton outbreak of waterborne gastro-enteritis. Owen Sound, Ont. Canada: Bruce-Grey-Owen Sound Health Unit. Cressey PJ, Lake R (2007). Risk ranking: Estimates of the burden of foodborne disease for New Zealand. Institute of Environmental Science & Research Limited; Christchurch Science Center.

Dundas S, Murphy J, Soutar RL, Jones GA, Hutchinson SJ, Todd WT. Effectiveness of therapeutic plasma exchange in the 1996 Lanarkshire Escherichia coli O157:H7 outbreak. Lancet. 1999;354: 1327–30

Frenzen PD, Drake A, Angulo FJ (2005). Economic cost of illness due to Escherichia coli O157 infections in the United States. J Food Prot 68 (12): 2623-30.

Haagsma J.A., P.D. Siersema, N.J. De Wit, A.H. Havelaar (2010). Disease burden of post-infectious irritable bowel syndrome in the Netherlands. Epidemiol Infect 138(11): 1650-6.

Haagsma JA, Maertens de Noordhout C, Polinder S, Vos T, Havelaar AH, Cassini A, Devleesschauwer B, Kretzschmar ME, Speybroeck N, Salomon JA. Assessing disability weights based on the responses of 30,660 people from four European countries. Population Health Metrics 2015; 13: 10

Havelaar AH, van Duynhoven YT, Nauta MJ, Bouwknegt M, Heuvelink AE, de Wit GA, et al. (2003) Disease burden in The Netherlands due to infections with Shiga toxin-producing Escherichia coli O157. Epidemiol Infect 132(3): 467-84.

Marshall JK, Thabane M, Garg AX, Clark WF, Moayyedi P, Collins SM. Walkerton Health Study Investigators (2010). Eight year prognosis of post- infectious irritable bowel syndrome following waterborne bacterial dysentery. Gut;59: 605-611.

McPherson M, Kirk MD, Raupach J, Combs B, Butler JR. (2011) Economic costs of Shiga toxin-producing Escherichia coli infection in Australia. Foodborne Pathog Dis 8 (1): 55-62.

Mead PS, Slutsker L, Dietz V, McCaig LF, Bresee JS, Shapiro C et al. (1999). Food-related illness and death in the United States. Emerg Infect Dis 5(5): 607-625.

Michel P, Wilson JB, Martin SW, Clarke RC, McEwen SA, Gyles CL (2000). Estimation of the under-reporting rate for surveillance of Escherichia coli O157:H7 cases in Ontario, Canada. Epidemiol Infect 125: 35-45.

Oxford Medical Dictionary (2003). Oxford, Oxford University Press; 3rd edition.

Tariq L, Haagsma J, Havelaar A (2011). Cost of Illness and Disease Burden in The Netherlands Due to Infections with Shiga Toxin-Producing Escherichia coli O157. J Food Prot 74 (4): 545-52.

Ternhag A, Törner A, Svensson A, Ekdahl K, Giesecke J (2008). Short- and long-term effects of bacterial gastrointestinal infections. Emerg Infect Dis 14 (1): 143-8.

Thabane M, Simunovic M, Akhtar-Danesh N, Marshall JK (2009). Development and Validation of a Risk Score for Post-Infectious Irritable Bowel Syndrome. Am J Gastroenterol; 104:2267–2274.

Syphilis

Syphilis is a complex, systemic disease caused by the spirochaete Treponema pallidum (T. pallidum), a gram-negative bacterium. Syphilis is preventable and curable with effective and inexpensive antibiotics. The only known natural hosts are humans, and the pathogen is not able to survive outside its host due to limited metabolic capacities to synthetise its own bio-nutrients. Syphilis spirochetes, like other treponemas, cannot be cultivated in vitro. The primary mode of syphilis transmission is by sexual contact (acquired syphilis). Vertical transmission from infected mother to child is possible (congenital syphilis), either in utero (transfer across the placenta) or through contact with an active genital lesion during delivery (Singh, 1999). Untreated syphilis can adversely affect pregnancy outcomes, resulting in spontaneous abortion, stillbirth, premature delivery, or perinatal death. Prematurity and low birth weight have been observed in 10 to 40% of infants born to untreated mothers (Salojee, 2004). The rate of infection through sexual intercourse with an infected partner has been estimated at about 50% (Ficarra & Carlos, 2009).

In Europe and other high-income countries, the transmission via blood or blood products is rare because of the low incidence rates of the disease and improved blood screening and blood donor testing for syphilis (Tramont, 2005).

Only 50% of those infected with T. pallidum will develop symptoms (RKI, 2003). Primary syphilis lasts from two weeks to six months (Baughn & Musher, 2005). Secondary syphilis may last two to eight weeks (Zetola, 2007). Early latent disease is diagnosed less than one year after infection (WHO, 2003; MMWR, 2010). Late latent syphilis infection is diagnosed after more than one year (WHO, 2003; MMWR, 2010).

Health outcomes and states associated with syphilis infection in adults

The incubation period for primary syphilis is on average three weeks (10–90 days) and depends on bacterial load, the immune status of the infected person and the existence of other co-morbid conditions (e.g. HIV/AIDS) (Weir & Fisman, 2002; Krause, 2006). Acquired syphilis is divided into primary, secondary, latent and tertiary syphilis. The disease can also be divided into early and late syphilis. Early syphilis implies the primary, secondary and early latent stages. Late syphilis refers to late latent syphilis and tertiary syphilis (Hook, 1992).

Primary syphilis is characterised by an ulcer and/or chancre at the site of infection or inoculation. This primary lesion appears about three weeks after exposure as an indurated, painless ulcer and may not be clinically evident (i.e. it may be in the rectum or the cervix). Invasion of the bloodstream precedes the initial lesion. In 50% of cases, the chancre is accompanied by regional lymphadenopathy (a firm, non-tender satellite lymph node) (Genc, 2000). After three to six weeks the chancre begins to involute, but may persist in the secondary stage in 15–30% of those infected (Zetola, 2007; Krause, 2006; Parish, 2000).

After 2–12 weeks on average (sometimes 12 months) the untreated infection may progress to secondary syphilis caused by the haematogenic spread and lymphatic dissemination of T. pallidum in the body. The time at which the secondary lesions manifest depends on the bacterial load of the treponeme and the immune response of the host (Baughn, 2005). This stage is characterised by skin rash, condylomata lata (5–22% of patients), mucocutaneous lesions, alopecia (5–7% of patients), and generalised lymphadenopathy (Ficarra & Carlos, 2009). A patient with secondary syphilis may have one, several or all of the signs of the secondary stage. Since each of the signs may also be associated with other diseases, none are specific to syphilis. Neurological involvement in secondary syphilis (known as syphilitic meningitis) can occur, especially in HIV co-infected patients (Marra, 2004). The manifestations of secondary syphilis last two to eight weeks and then may resolve, even without treatment (Zetola, 2007).

After resolution of the secondary manifestations, around one-third of untreated patients will enter into a latent phase. The latent or asymptomatic stage of syphilis is defined as the period from disappearance of the secondary manifestations until therapeutic cure or development of late sequelae. An infection without any clinical symptoms lasting less than one year is referred to as early latent syphilis, whereas an infection of more than one year's duration without clinical evidence of treponemal infection is referred to as late latent syphilis (WHO, 2003). The definitions of duration may vary across countries. The early latent period corresponds to the highest risk of transmission.

Tertiary syphilis may appear after a long period of untreated syphilis (5–20 years after initial infection) and its manifestations can include gummas (late benign syphilis), cardiovascular symptoms and neurosyphilis (Hutto, 2001). In developed countries gummas and cardiovascular symptoms are rarely seen and most of the late sequelae are associated with neuro-syphilis. The timescale for development of neuro-syphilis may vary from a period of one or two years to more than 30 years after primary syphilis, and may involve 5–10% of untreated patients (Gjestland, 1955). It is characterised by the involvement of the central nervous system which leads to a number of different syndromes, included in the health outcome 'neuro-syphilis' in our model. In two thirds of patients the infection will not progress to late complications (Mindel, 2000).

Health outcomes and states associated with congenital syphilis infection

Postnatal manifestations of congenital syphilis are divided into early and late stages. Clinical manifestations occurring within the first two years after birth (<2 years) are categorised as early congenital syphilis. Clinical manifestations which occur later than two years after birth are late congenital syphilis (Parish, 2000). For the underlying model, and due to scarce data, only congenital syphilis was included, with no distinction between early or late.

Outcome tree parameters

Due to the high complexity of syphilis outcomes and for reasons of feasibility, the outcome tree for the adult population was split into symptomatic and asymptomatic infections at the first level of disaggregation. The natural course of syphilis was subdivided into the three main disease states: primary, secondary and neuro-syphilis. The focus was on neuro-syphilis because other forms of late syphilis sequelae are very rare in developed countries.

The percentage of asymptomatic cases was estimated at 50% (RKI 2003, Singh, 1999; Ficarra, 2009; Genc, 2000; Parish, 2000). Gerbase and colleagues presented treatment rates of 85% for both primary and secondary symptomatic syphilis cases in regions with established market economies (Gerbase, 2000). As a result of high cure rates (up to 100%), it was estimated that about 85% of all primary syphilis cases are treated and subsequently cured. The remaining 15% of untreated symptomatic cases have a 30–50% possibility of developing secondary syphilis, resulting in a probability of 4.5–7.5% that they will develop secondary syphilis, after having had primary syphilis (Singh, 1999; Weir & Fisman, 2002; Krause, 2006; Gerbase, 2000; Golden, 2003). In asymptomatic primary syphilis the primary chancre is not visible and will generally go unnoticed, meaning that it is less likely to be treated, hence the greater risk of progression to secondary syphilis (30–50%).

Furthermore, 85% of symptomatic secondary syphilis cases are treated and again, as a result of the high cure rates (around 100%), the remaining 15% of untreated cases have a probability of 5–12% of developing neuro-syphilis. Thus, the proportion of people developing neuro- syphilis from preceding secondary syphilis was set at 0.75–1.88% (Tramont, 2005; Zetola, 2007; Gerbase, 2000; Goldmeier & Guallar, 2003).

The probability of dying due to syphilis before reaching the late (tertiary) phase of the disease is very low and there is little evidence of a case fatality ratio associated with syphilis in general, or neurosyphilis in particular, within Europe. We assumed that neurosyphilis in Europe is successfully treated; although with a possibility of developing permanent disabilities for which it was impossible to define the impact due to lack of data. Antibiotic treatment is highly effective and is therefore not associated with a case fatality ratio.

For infants the main outcome is congenital infection with a probability of 20% (2–64%) for an infected child (Singh, 1999; Salojee, 2004; Genc, 2000; Gerbase, 2000). In total, 1% of all children with congenital infection die (Gerbase, 2000).

Model input summary

Table 1. Transition probabilities and distributions used in the outcome tree

Health outcome<br>(Health state)	Transition probability	Source/assumption
Acquired		
Primary syphilis from infection	50%	RKI, 2003
Secondary syphilis from asymptomatic infection	30–50%	Singh, 1999; Weir & Fisman, 2002; Gerbase, 2000; Golden 2003
Secondary syphilis from symptomatic infection	4.5–7.5%	Singh, 1999; Weir & Fisman, 2002; Gerbase, 2000; Golden 2003
Neuro-syphilis	0.75–1.88%	Tramont, 2005; Zetola, 2007; Krause, 2006; Weir&Fisman, 2002; Gerbase, 2000; Golden, 2003; Goldmeier, 2003
Fatal cases due to neurosyphilis	0%	Assuming all cases are identified and treated, and no treatment failure
Congenital		
Symptomatic infection	20% (2–64%)	Singh, 1999; Saloojee, 2004; Genc & Ledger, 2000

Fatal cases due to congenital infection	1%	Genc & Ledger, 2000

Table 2. Disability weights and duration

Health outcome (Health state)	Disability Weight (DW) (Haagsma, 2015)		Duration	
	DW	Label	In years	Source
Acquired				
Primary syphilis	0.007 (0.005-0.01)	Infectious disease, acute episode, mild	0.121-0.5	Baughn & Musher, 2005
Latency period (from primary to secondary)	0		0.23 (0.038-1)	Baughn, 2005
Secondary syphilis	0.125 (0.104-0.152)	Infectious disease, acute episode, severe	0.038-0.153	Zetola, 2007
Latency period (from secondary to neurosyphilis)	0		4.77-19.77	Hutto, 2001
Neurosyphilis	0.407 (0.36-0.46)	Motor plus cognitive impairments, severe	0.027-0.038	Workowski, 2010 Assuming 10–14 days of treatment
Congenital				
Symptomatic infection	0.125 (0.104-0.152)	Infectious disease, acute episode, severe	3	Kwong, 2010

References

Baughn RE, Musher DM. Secondary syphilitic lesions. Clin Microbiol Rev 2005, 18:205-216.

Bremer, V, Marcus U, Hamouda O (2012). Syphilis on the rise again in Germany--results from surveillance data for 2011. Euro Surveill 17(29).

[SW1] Doherty L, Fenton KA, Jones J, Paine TC, Higgins SP, Williams D, et al. Syphilis: old problem, new strategy. BMJ 2002, 325:153-156.

Ficarra G, Carlos R. Syphilis: the renaissance of an old disease with oral implications. Head Neck Pathol 2009, 3:195-206.

Genc M, Ledger WJ. Syphilis in pregnancy. Sex Transm Infect 2000, 76:73-79.

Gerbase A, Stein C, Levison J, Htun Y. Global burden of sexually transmitted diseases (excluding HIV) in the year 2000. In: Global burden of sexually transmitted diseases (excluding HIV) in the year 2000. Geneva; World Health Organization; 2000.

Gjestland T. The Oslo study of untreated syphilis; an epidemiologic investigation of the natural course of the syphilitic infection based upon a re-study of the Boeck-Bruusgaard material. Acta Derm Venereol Suppl (Stockh) 1955, 35:3-368; Annex I-LVI.

Golden MR, Marra CM, Holmes KK. Update on syphilis: resurgence of an old problem. JAMA 2003, 290:1510-1514.

Goldmeier D, Guallar C. Syphilis: an update. Clin Med 2003, 3:209-211.

Haagsma JA, Maertens de Noordhout C, Polinder S, Vos T, Havelaar AH, Cassini A, Devleesschauwer B, Kretzschmar ME, Speybroeck N, Salomon JA. Assessing disability weights based on the responses of 30,660 people from four European countries. Population Health Metrics 2015; 13: 10

Hook EW, 3rd, Marra CM. Acquired syphilis in adults. N Engl J Med 1992, 326:1060-1069.

Hutto B. Syphilis in clinical psychiatry: a review. Psychosomatics 2001, 42:453-460.

Krause W. [Syphilis]. Urologe A 2006, 45:1494-1500.

Kwong JC, Crowcroft NS, Campitelli MA, Ratanasingham S, Daneman N, Deeks SL, et al. Ontario Burden of Infectious Disease Study. OAHPP/ECES Report. City: Ontario Agency for Health Protection and Promotion and the Institute for Clinical Evaluative Sciences; 2010.

Limb M. New scheme aims to eradicate congenital syphilis in three years. BMJ 2012, 344:e1616.

Marra C. Neurosyphilis. Curr Neurol Neurosci Rep 2004, 4:435-440.

Mindel A, Estcourt C. Syphilis. In: Sexually transmitted diseases: Vaccines, Prevention and Control. Edited by Stanberry LR, Bernstein DI: Academic Press, San Diego, San Francisco, New York, Boston, London, Sydney, Tokyo; 2000

Murray CJL, Lopez AD, World Health Organization, World Bank, Harvard School of Public Health. Global health statistics: a compendium of incidence, prevalence and mortality estimates for over 200 conditions. Geneva: World Health Organization; 1996.

Murray CJL, Lopez AD. The Global Burden of Disease - A comprehensive assessment of mortality and disability from diseases, injuries, and risk factors in 1990 and projected to 2020. Cambridge: Harvard School of Public Health on behalf of the World Health Organization and the World Bank; 1996.

Parish JL. Treponemal infections in the pediatric population. Clin Dermatol 2000, 18:687-700.

Robert Koch Institut. RKI-Ratgeber Infektionskrankheiten - Merkblätter für Ärzte: Syphilis (Lues). Epidemiologisches Bulletin 2003, 30:229-233.

Saloojee H, Velaphi S, Goga Y, Afadapa N, Steen R, Lincetto O. The prevention and management of congenital syphilis: an overview and recommendations. Bull World Health Organ 2004, 82:424-430.

Singh AE, Romanowski B. Syphilis: review with emphasis on clinical, epidemiologic, and some biologic features. Clin Microbiol Rev 1999, 12:187- 209.

Tramont EC. Treponema pallidum (Syphilis). In: Principles and practice of infectious diseases. Volume Volume 2, 6th Edition. Edited by Mandel GL, Bennet JE, Dolin R. Pennsylvania: Elsevier Inc. 2005: 2768-2785

Weir E, Fisman D. Syphilis: have we dropped the ball? CMAJ 2002, 167:1267-1268.

Workowski KA, Berman SM; Centers for Disease Control and Prevention. Sexually transmitted diseases treatment guidelines, 2010. MMWR Recomm Rep. 2010;59(RR-12):1-110.

World Health Organization. Dept. of Reproductive Health and Research. Guidelines for the management of sexually transmitted infections. Geneva: World Health Organization; 2003.

Zetola NM, Engelman J, Jensen TP, Klausner JD. Syphilis in the United States: an update for clinicians with an emphasis on HIV coinfection. Mayo Clin Proc 2007, 82:1091-1102.

Tetanus

Tetanus is an acute and often fatal disease induced by the tetanospasmin, an exotoxin produced by Clostridium tetani, a gram-positive anaerobic bacillus (Bleck, 2005; CDC, 2012). C. tetani is sensitive to heat and not viable under aerobic conditions (CDC, 2012). In contrast, the spores of C. tetani are resistant to heat and antiseptics and are widely present in soil and in the intestines and faeces of animals (e.g. horses, sheep and dogs). Tetanus is primarily contracted via contaminated wounds and is not contagious. Effective vaccination programmes significantly reduced the burden of tetanus. Globally around 800 000 to 1 000 000 people die of tetanus each year (Dietz, 1996). Around 90% of all deaths occur in developing countries which are largely affected by tetanus and especially neonatal and maternal tetanus. In developed countries, high-risk groups, such as unvaccinated persons and injecting drug users, are prone to infection with C. tetani (CDC, 2012). The proportion of asymptomatic/subclinical infections is unknown but it can be assumed that cases of tetanus are symptomatic in nearly 100% of those infected. The first symptoms of tetanus appear after an average incubation period of eight days (range: 3–21 days) (CDC, 2012). The duration of the symptomatic disease for generalised, localised and cephalic tetanus is two to three weeks (CDC, 2012).

Health outcomes/states associated with tetanus infection

The clinical features of acute tetanus infections can be subdivided into three health states that are observed in developed countries. A fourth type, tetanus neonatorum is a specific form of generalised tetanus that affects neonates and is mostly observed in the developing world with a high case fatality of up to 90% (Roper, 2007). As neonatal tetanus has been eliminated in Europe this health outcome is not considered in our outcome tree and model.

The distribution of the three health states is set according to the observed risk of developing the different forms of acute infection in USA (Bardenheier, 1998): 81% were generalised; 13% localised and 6% cephalic.

Localised tetanus

Localised tetanus is an uncommon health state of tetanus. Localised tetanus appears as a persistent contraction of muscles in the injured area, commonly preceding generalised tetanus, and lasts around two to three 3 weeks (CDC, 2012).

Generalised tetanus

The most common health state of tetanus infection is generalised tetanus. The probability of developing generalised tetanus after initial infection is around 80% (CDC, 2012; Bardenheier, 1998; Guilfoile, 2008). The symptoms of generalised tetanus are trismus or lockjaw in the early stages, developing into stiffness of the neck, difficulty in swallowing and rigidity of abdominal muscles. Further, unspecific symptoms such as elevated temperature, sweating, elevated blood pressure, and episodic rapid heart rate may occur. Generalised tetanus can last for 3-4 weeks and full recovery may take several months (CDC, 2012).

Cephalic tetanus

Cephalic tetanus is another uncommon health state involving the cranial nerves. The same duration has been assumed for this health state as for localised tetanus: 2–3 weeks.

Further complications and case fatality proportion

In cases of cephalic tetanus otitis media may occur (CDC, 2012). Long-term sequelae/disabilities from tetanus are not reported in the literature.

The overall mortality rate of tetanus ranges from 28/100 000 in developing countries to 0.1/100 000 in developed countries such as the USA. The case fatality proportion ranges between 5 and 55% (Guilfoile, 2008; Brook, 2004; Cook, 2001; Farrar, 2000; Kanchanapongkul, 2001; Miranda-Filho Dde 2004; Saltoglu, 2004; Sanford, 1995; Thwaites, 2004; Trujillo, 1987). Mortality from tetanus is clearly dependent on age, immune status and vaccination. People over 60 years of age or unvaccinated persons have an elevated lethality of 18 and 22%, respectively. In the model, the mortality rate following symptomatic cases was set at 11% (CDC, 2012; Bardenheier, 1998).

Model input summary

Table 1. Transition probabilities used in the outcome tree

Health outcome (Health state)	Distribution of health states in health outcome	Transition probability	Source/assumption
Symptomatic infection<br>(Localised tetanus)<br>(Generalised tetanus)<br>(Cephalic tetanus)	 13%<br>81%<br>6%		Bardenheier, 1998
Fatal cases		11%	CDC, 2012<br>Bardenheier, 1998

Table 2. Disability weights and duration

Health outcome (Health state)	Disability Weight (DW) (Haagsma, 2015)		Duration	
	DW	Label	In years	Source
Symptomatic infection (Generalised tetanus)	0.421 (0.377-0.477)	Motor impairment, severe	0.06-0.08	CDC, 2012
(Localised tetanus)	0.011 (0.008-0.014)	Motor impairment, mild	0.04-0.06	CDC, 2012
(Cephalic tetanus)	0.053 (0.042-0.064)	Motor impairment, moderate	0.04-0.06	CDC, 2012 Assumed same as for localised

References

Bardenheier B, Prevots DR, Khetsuriani N, Wharton M. Tetanus surveillance - United States, 1995-1997. MMWR CDC Surveill Summ 1998, 47:1- 13.

Bleck TP. Clostridium tetani (Tetanus). In Principles and practice of infectious diseases. Volume 2. Edited by Mandell GL, Bennett JE, Dolin R. Pennsylvania: Elsevier Inc.; 2005:2817–2822

Brook I. Tetanus in children. Pediatr Emerg Care 2004, 20:48-51.

Centers for Disease Control and Prevention: Tetanus. In Epidemiology and Prevention of Vaccine-Preventable Diseases. Edited by Atkinson W, Wolfe S, Hamborsky J. Washington DC: Public Health Foundation; 2012:291-300

Cook TM, Protheroe RT, Handel JM. Tetanus: a review of the literature. Br J Anaesth 2001, 87:477-487.

Dietz V, Milstien JB, van Loon F, Cochi S, Bennett J: Performance and potency of tetanus toxoid: implications for eliminating neonatal tetanus. Bull World Health Organ 1996, 74:619-628.

Farrar JJ, Yen LM, Cook T, Fairweather N, Binh N, Parry J, et al. Tetanus. J Neurol Neurosurg Psychiatry 2000, 69:292-301.

Guilfoile PG. Tetanus. New York: Infobase Publishing; Chelsea House; 2008.

Haagsma JA, Maertens de Noordhout C, Polinder S, Vos T, Havelaar AH, Cassini A, Devleesschauwer B, Kretzschmar ME, Speybroeck N, Salomon JA. Assessing disability weights based on the responses of 30,660 people from four European countries. Population Health Metrics 2015; 13: 10

Hsu SS, Groleau G. Tetanus in the emergency department: a current review. J Emerg Med 2001, 20:357-365.

Kanchanapongkul J. Tetanus in adults: a review of 85 cases at Chon Buri Hospital. J Med Assoc Thai 2001, 84:494-499.

Miranda-Filho Dde B, Ximenes RA, Barone AA, Vaz VL, Vieira AG, Albuquerque VM. Randomised controlled trial of tetanus treatment with anti- tetanus immunoglobulin by the intrathecal or intramuscular route. BMJ 2004, 328:615.

Murray CJL, Lopez AD. World Health Organization., World Bank., Harvard School of Public Health.: Global health statistics : a compendium of incidence, prevalence and mortality estimates for over 200 conditions. Geneva: World Health Organization; 1996.

Roper MH, Vandelaer JH, Gasse FL. Maternal and neonatal tetanus. Lancet 2007, 370:1947-1959.

Saltoglu N, Tasova Y, Midikli D, Burgut R, Dundar IH. Prognostic factors affecting deaths from adult tetanus. Clin Microbiol Infect 2004, 10:229- 233.

Sanford JP. Tetanus: forgotten but not gone. Waltham, MA, : Massachusetts Medical Society; 1995.

Thwaites CL, Yen LM, Nga NT, Parry J, Binh NT, Loan HT et al. Impact of improved vaccination programme and intensive care facilities on incidence and outcome of tetanus in southern Vietnam, 1993-2002. Trans R Soc Trop Med Hyg 2004, 98:671-677.

Trujillo MH, Castillo A, Espana J, Manzo A, Zerpa R. Impact of intensive care management on the prognosis of tetanus. Analysis of 641 cases. Chest 1987, 92:63-65.

Tick-borne encephalitis (TBE)

Most cases of tick-borne encephalitis (TBE) in Europe involve a biphasic presentation of the disease with fever during the first phase and neurological disorders during the second phase (Gubler, 2007). Severity of tick-borne encephalitis increases with age. TBE in children (<14 years) usually runs a more benign course (Mickiene, 2002; Kaiser, 1999). The proportion of asymptomatic cases is 66–80% (Gustafson, 1992). To calculate the burden of disease we assume that asymptomatic patients do not develop sequelae and are not included in the burden estimation.

The subtype considered is the Central European encephalitis subtype (Western tick-borne encephalitis virus) which is the dominant one in Europe. Another subtype does occur, the Russian spring-summer encephalitis subtype, however this occurs less in EU Member States and is not considered in the outcome tree.

The symptomatic infection (viraemic phase) begins after an average incubation period of eight days (range 4–28 days) (Kaiser, 1999). Symptoms of this first phase include fever, muscle pain, fatigue and headache (Gunther, 1997; Kaiser, 1999), normally lasting for five (2–7) days (Gubler, 2007).

Meningoencephalitic phase

After a symptom-free period, usually less than two weeks, a meningoencephalitic second phase occurs in 20–30% of symptomatic patients (Gustafson, 1990; 1992; Kiffner, 2010). The duration of the meningoencephalitic phase is set to 15 days (10–70) (Kaiser, 1999). The case fatality proportion of the meningoencephalitic phase is set to 0.75% (Mickiene, 2002).

Paralysis and residual paresis

Following the meningoencephalitic phase there is a latency period of six days (range 1–17 days), after which paralysis occurs in an estimated 11% of patients (Gunther, 1997). The duration is set to 3–10 days (Kaiser, 1999). Overall, 56% of paralytic patients are at risk of developing lifelong residual paresis (partial loss of or impaired movement) (Gunther, 1997).

Post-encephalitic TBE syndrome

A long-term post-encephalitic TBE syndrome, with symptoms including cognitive or neuropsychiatric complaints, balance disorders, headache, dysphasia, hearing defects and spinal paralysis, has been reported in 39–46% of meningoencephalitic patients (Gunther, 1997; Mickiene, 2002). The duration of post-encephalitic TBE syndrome is set to one year ('Post TBE syndrome existed after 1 year in more than one third of the patients' Gunther, 1997).

Lifelong chronic sequelae can persist in 35.7% (Haglund & Gunther, 2003) to 38.8% of post-encephalitic syndrome patients (Gunther, 1997: 'persisting symptoms at 12 months in 33/85 patients'). Males are affected twice as much as females and 12% of patients with post-encephalitic TBE syndrome were under 14 years of age (Kaiser, 1999). However, the association between gender, age and severity still needs more research and is not considered in the outcome tree.

Model input summary

Table 1. Transition probabilities used in the outcome tree

Health outcome (Health state)	Distribution of health states in health outcome	Transition probability	Source/assumption
Symptomatic infection		20–34%	Gustafson, 1992
Meningoencephalitic phase		20–30%	Gustafson, 1990, 1992; Kiffner, 2010
Paralysis		11%	Kaiser, 1999; Gunther, 1997
Residual paresis		56%	Gunther, 1997
Post-encephalitic TBE syndrome		39–46%	Gunther 1997; Mickiene, 2002
Chronic post-encephalitic TBE syndrome		35.7–38.8%	Haglund & Gunther, 2003<br>Gunther, 1997
Fatal cases following meningoencephalitic phase		0.75%	Mickiene, 2002

Table 2. Disability weights and duration

Health outcome (Health state)	Disability Weight (DW) (Haagsma, 2015)		Duration	
	DW	Label	In years	Source

Symptomatic infection	0.051 (0.039-0.06)	Infectious disease, acute episode, moderate	0.014 (0.005-0.019)	Gubler, 2007
Meningoencephalitic phase	0.447 (0.391-0.501)	Encephalopathy - severe	0.041 (0.027-0.192)	Kaiser, 1999
Paralysis	0.526 (0.469-0.586)	Spinal cord lesion at neck level (treated)	0.0137	Kaiser, 1999
Residual paresis	0.056 (0.044-0.067)	Motor plus cognitive impairments, mild	Remaining life expectancy	Remaining life expectancy
Post-encephalitic TBE syndrome	0.202 (0.167-0.242)	Motor plus cognitive impairments, moderate	1	Gunther, 1997
Chronic post-encephalitic TBE syndrome	0.056 (0.044-0.067)	Motor plus cognitive impairments, mild	Remaining life expectancy	Remaining life expectancy

References

Gubler JD, Kuno G, Markoff L. 2007. Flaviviruses. In: Knipe, D.M., Howley, P.M. (Eds.), Fields Virology 5th ed., vol. 1. Lippincott Williams & Wilkins, London, New York, Tokyo, pp. 1043–1125.

Gustafson R, Svenungsson B, Forsgren M, Gardulf A, Granström M. Two-year survey of the incidence of Lyme borreliosis and tick-borne encephalitis in a high-risk population in Sweden. European Journal of Clinical Microbiology and Infectious Diseases, 1992 Oct;11(10):894-900. PMID: 1486884

Gustafson R, Svenungsson B, Gardulf A, Stiernstedt G, Forsgren M. Prevalence of tick-borne encephalitis and Lyme borreliosis in a defined Swedish population. Scandinavian Journal of Infectious Diseases, 1990;22(3):297-306. PMID: 2371545

Günther G, Haglund M, Lindquist L, Forsgren M, Sköldenberg B. Tick-bone encephalitis in Sweden in relation to aseptic meningo-encephalitis of other etiology: a prospective study of clinical course and outcome. Journal of Neurology, 1997 Apr;244(4):230-8. PMID: 9112591

Haglund M, Günther G. Tick-borne encephalitis--pathogenesis, clinical course and long-term follow-up. Vaccine, 2003 Apr 1;21 Suppl 1:S11-8. PMID: 12628810

Haagsma JA, Maertens de Noordhout C, Polinder S, Vos T, Havelaar AH, Cassini A, Devleesschauwer B, Kretzschmar ME, Speybroeck N, Salomon JA. Assessing disability weights based on the responses of 30,660 people from four European countries. Population Health Metrics 2015; 13: 10

Kaiser R. The clinical and epidemiological profile of tick-borne encephalitis in southern Germany 1994-98: a prospective study of 656 patients. Brain 1999; 122 (Pt 11): 2067-78. PMID: 10545392

Kiffner C, Zucchini W, Schomaker P, Vor T, Hagedorn P, Niedrig M, et al. Determinants of tick-borne encephalitis in counties of southern Germany, 2001-2008. International Journal of Health Geography, 2010 Aug 13;9:42. PMID: 20707897

Mickiene A, Laiskonis A, Gunther G, et al. Tick-borne encephalitis in an area of high endemicity in Lithuania: disease severity and long-term prognosis. Clin Infect Dis 2002; 35 (6): 650-8 PMID: 12203160

Toxoplasmosis

Acquired toxoplasmosis

In Europe, most cases of acquired toxoplasmosis are asymptomatic and self-limiting (Rorman, 2006). Acquired toxoplasmosis will lead to symptomatic illness in approximately 10–20% of infected cases (Montoya, 2000). It is estimated that 4.67% (0–15.3%) of symptomatic cases will manifest more severe symptoms and approximately 2% (0–4.67%) are at risk of developing life-long sequelae relative to chorioretinitis. However, it is unclear if this risk is attributable mainly to more severe, symptomatic infections or all infections (Kemmeren, 2006). All other symptomatic cases will manifest minor symptoms, such as fever and lymphoadenopathy (Rorman, 2006; Anand, 2012).

Mortality due to acquired toxoplasmosis is extremely rare and occurs in immunocompromised patients. It has therefore been decided to exclude fatal cases from the outcome tree of acquired toxoplasmosis.

Toxoplasmosis may also play a role in the development of psychiatric disorders, such as schizophrenia and bipolar depression (Torrey, 2003; Henriquez, 2009; Brown, 2010). However, insight into causality is still insufficient and these sequelae are not included in the model.

Congenital toxoplasmosis

Vertical transmission from a recently infected pregnant woman to her foetus may lead to congenital toxoplasmosis. Infections occurring during the first and second trimester of pregnancy may result in foetal loss (1.5–1.7% of seroconverting pregnant women, Havelaar 2007) or stillbirth (although neither of these are included in the present burden estimation) and symptoms in newborn infants are generally more severe.

However, if the infection occurs in the third trimester the disease manifestation is generally subclinical. When present, symptoms vary from a triad including chorioretinitis, intracranial calcification and hydrocephalus to abnormalities of the central nervous system. These complications may lead to life-long sequelae, including subclinical congenital toxoplasmosis which could increase the risk of developing chorioretinitis later in life. Death can occur in a small proportion of infections. Other symptoms are very rare and have not been considered in this model.

Several studies have described clinical manifestations and follow-up of newborns infected with toxoplasmosis: 89% of children were asymptomatic at birth (16% of them developed chorioretinitis later in life) (Berrebi, 2010), 85% had no clinical findings at birth (Lebech, 1999) and 74.5% were asymptomatic at birth (Schmidt, 2006). Therefore, the proportion of asymptomatic infections out of the total congenital toxoplasmosis infections is 11–25%.

Asymptomatic congenital toxoplasmosis-infected infants have a 2% (1–3%) per year risk of developing chorioretinitis at a later age. The studies followed cases of asymptomatic congenital toxoplasmosis for 10–14 years (Havelaar, 2007).

Based on an extensive literature review, Havelaar et al. (Haavelar, 2007) estimated the risk of developing permanent disabilities related to congenital toxoplasmosis infections. We applied the same estimates to our model for all infections: 13% (12–15%) will develop permanent disabilities due to complications related to chorioretinitis, 11% (8–12%) to intracranial calcification, 3% (1-6%) to the central nervous system and 2% (1–3%) to hydrocephalus.

Model input summary

Table 1. Percentages used in the outcome tree

Health outcome<br><br>(Health state)	Distribution of health<br><br>states in health outcome	Transition probability	Source/assumption

Acquired toxoplasmosis			
Symptomatic infections:<br>(Uncomplicated)<br>(Complicated)	 Remaining cases<br>4.67% (0–15.3%)	10–20%	Kemmeren, 2006
Chorioretinitis following symptomatic infection		2% (0–4.67%)	Kemmeren, 2006
Congenital toxoplasmosis			
Symptomatic infections:<br>(Asymptomatic)<br>(Symptomatic)	75–89%<br>Remaining cases		Berrebi, 2010<br>Lebech, 1999<br>Schmidt, 2006
Permanent disability due to chorioretinitis after the first year following asymptomatic infection		2% (1-3%) per year (ATP) for 10–14 years	Havelaar, 2007<br>Starting one year after infection up to the age of 10–14 years<br>ATP: Annual Transition Probability

Permanent disability due to chorioretinitis within first year		13% (12–15%)	Havelaar, 2007
Permanent disability due to intracranial calcification		11% (8–12%)	Havelaar, 2007
Permanent disability due to hydrocephalus		2% (1–3%)	Havelaar, 2007
Permanent disability due to CNS abnormalities		3% (1–6%)	Havelaar, 2007
Fatal cases		0.7% (0.4–1.2%)	Havelaar, 2007

Table 2. Disability weights and duration

Health outcome (Health state)	Disability Weight (DW) (Haagsma, 2015)		Duration	
	DW	Label	In years	Source
Acquired toxoplasmosis				
Acquired toxoplasmosis (Uncomplicated)<br>(Complicated)	0.007 (0.005–0.01)<br>0.125 (0.104–0.152)	Infectious disease, acute episode, mild<br>Infectious disease, acute episode, severe	0.04	Kemmeren, 2006
Congenital toxoplasmosis				
Congenital toxoplasmosis (Asymptomatic)<br>(Symptomatic)	0<br>0.125 (0.104–0.152)	Infectious disease, acute episode, mild<br>Infectious disease, acute episode, severe	1<br>0.167	Assuming chorioretinitis starts after one year<br>Melse, 2000
Permanent disability due to chorioretinitis following asymptomatic infections	0.015 (0.011–0.019)	Conjunctivitis without corneal scar	rem life exp.	Havelaar, 2007
Permanent disability due to	0.015 (0.011–0.019)	Conjunctivitis without corneal	rem life exp.	Havelaar, 2007

chorioretinitis following symptomatic infections		scar		
Permanent disability due to intracranial calcification	0.044–0.087	Intellectual disability/mental retardation, from mild to moderate	rem life exp.	Havelaar, 2007
Permanent disability due to hydrocephalus	0.044–0.188	Intellectual disability/mental retardation, from mild to severe	rem life exp.	Havelaar, 2007
Permanent disability due to CNS abnormalities	0.056–0.407	Motor plus cognitive impairments, from mild to severe	rem life exp.	Havelaar, 2007
Permanent disability due to chorioretinitis	0.015 (0.011–0.019)	Conjunctivitis without corneal scar	rem life exp.	Kemmeren, 2006

References

Anand R, Jones CW, Ricks JH, Sofarelli TA, Hale DC. Acute primary toxoplasmosis in travelers returning from endemic countries. J Travel Med. 2012; 19(1): 57-60.

Berrebi A, Assouline C, Bessieres MH, Lathiere M, Cassaing S, Minville V, et al. Long-term outcome of children with congenital toxoplasmosis. American journal of obstetrics and gynecology. 2010; 203(6): 552 e1-6.

Brown AS, Derkits EJ. Prenatal infection and schizophrenia: a review of epidemiologic and translational studies. Am J Psychiatry. 2010; 167(3): 261-80.

Burnett AJ, Shortt SG, Isaac-Renton J, King A, Werker D, Bowie WR. Multiple cases of acquired toxoplasmosis retinitis presenting in an outbreak. Ophthalmology. 1998; 105(6): 1032-7.

Haagsma JA, Maertens de Noordhout C, Polinder S, Vos T, Havelaar AH, Cassini A, Devleesschauwer B, Kretzschmar ME, Speybroeck N, Salomon JA. Assessing disability weights based on the responses of 30,660 people from four European countries. Population Health Metrics 2015; 13: 10

Havelaar AH, Kemmeren JM, Kortbeek LM. Disease burden of congenital toxoplasmosis. Clin Infect Dis. 2007; 44(11): 1467-74.

Henriquez SA, Brett R, Alexander J, Pratt J, Roberts CW. Neuropsychiatric disease and Toxoplasma gondii infection. Neuroimmunomodulation. 2009; 16(2): 122-33.

Kemmeren JM, Mangen, MJJ, van Duynhoven YTHP, Havelaar AH. Priority setting of foodborne pathogens: disease burden and costs of selected enteric pathogens. Bilthoven: National Institute of Public Health and Environment; 2006. Report No.: RIVM rapport 330080001.

Lebech M, Andersen O, Christensen NC, Hertel J, Nielsen HE, Peitersen B, et al. Feasibility of neonatal screening for toxoplasma infection in the absence of prenatal treatment. Danish Congenital Toxoplasmosis Study Group. Lancet. 1999; 353(9167): 1834-7.

Melse JM, Essink-Bot ML, Kramers PG, Hoeymans N. A national burden of disease calculation: Dutch disability-adjusted life-years. Dutch Burden of Disease Group. Am J Public Health. 2000; 90(8): 1241-7.

Montoya J, Remingtons J. Toxoplasma gondii. In: Mandell G, Douglas, Benett J, editors. Principles and Practice of Infectious Diseases. Philadelphia: Churchill Livingstone; 2000.

Rorman E, Zamir CS, Rilkis I, Ben-David H. Congenital toxoplasmosis--prenatal aspects of Toxoplasma gondii infection. Reprod Toxicol. 2006; 21(4): 458-72.

Schmidt DR, Hogh B, Andersen O, Fuchs J, Fledelius H, Petersen E. The national neonatal screening programme for congenital toxoplasmosis in Denmark: results from the initial four years, 1999-2002. Archives of disease in childhood. 2006; 91(8): 661-5.

Torrey EF, Yolken RH. Toxoplasma gondii and schizophrenia. Emerg Infect Dis. 2003; 9(11): 1375-80.

Tuberculosis

Tuberculosis (TB) is an infectious disease caused by the Mycobacterium tuberculosis. The term �tuberculosis� is also used for other similar diseases caused by M. bovis and M. africanum (Fitzgerald, 2005; Comstock, 1998). However, for the purposes of the disease report, outcome tree and model presented here, only those infections caused by M. tuberculosis complex are considered.

Tuberculosis bacteria are transmitted via droplets � by coughing, sneezing or talking � and mostly affect the lungs of humans, although they can also result in a systemic disease, affecting virtually all organs (Fitzgerald, 2005). The course �of TB can be split into several phases. The first phase after infection, �primary TB�, is observed in a minority of patients. The majority of infected (asymptomatic) persons proceed to a �latent� stage, lasting from months to several years or even for the rest of their life. Due to endogenous or exogenous reactivation, people may develop active TB after a certain time spent in the latent stage of the disease.

According to published literature only 5�10% of all infected individuals develop symptoms of active (primary) TB (cough, fever, lethargy, and weight loss) in their lifetime (Castillo-Chavez & Feng, 1997; Gideon & Flynn, 2011, Lin & Flynn, 2010, North & Jung, 2004).

Health outcomes and health states associated with tuberculosis infection

The main health outcomes associated with TB infection are active (primary) TB, MDR (multidrug-resistant) TB and XDR (extensively drug- resistant) TB. After initial infection with M. tuberculosis, an immuno-competent person is generally able to stop the replication and spread of bacilli and thus does not develop any symptoms. Primary TB can be split in pulmonary TB (the majority of cases) and extra-pulmonary TB, affecting different sites of the human organism. Given the complexity of the disease course, all TB cases are considered in the model, with a focus on the distinction between drug-susceptible (DS TB), MDR and XDR TB and their relative case fatality proportions (CFP), irrespective of the site of infection.

Of all laboratory-confirmed TB cases notified to ECDC/WHO between 2009 and 2013, on average 4.5% were multidrug-resistant and 14.6% of these cases were extensively drug resistant (ECDC/WHO, 2015). Therefore, in our model of all symptomatic infections 4.5% are considered to be MDR TB and 0.64% are considered to be XDR TB. However, it should be noted that these proportions vary widely across countries and users are advised to tailor them according to the epidemiology of the population under study.

Transition probabilities

In a cost-effectiveness analysis performed by Tseng and colleagues the authors used various assumptions on the progression of TB. Their model estimates the risk of active TB to be about 5% within the first two years of TB infection. Spontaneous resolution without treatment was set to 25%. Cure rates of TB with treatment and cure rates of MDR TB with treatment were 62.4% and 68.6% respectively (Tseng, 2011).

Tiemersma and colleagues estimated CFP and assessed durations of untreated pulmonary TB in HIV-negative patients and stated an overall case-fatality proportion of 30.7% in the first year of follow-up. The highest proportions were observed shortly after diagnosis. The 5-year and 10-year averages for case fatalities were 58% and 73% respectively (Tiemersma, 2011). In their review they also included the study conducted by Berg, estimating sex- and age-specific 10-year mortality rates. For men aged 15�29, 30�49 and >50 years, the 10-year mortality rates were 66%, 70% and 94% respectively. For women aged 15�29, 30�49 and >50 years, 10-year mortality rates were 70%, 69% and 92% respectively (Berg, 1951). Assuming that detected TB cases are treated in Europe, the case fatality proportions cited above overestimate current TB mortality patterns. Duration of pulmonary TB and TB is difficult to estimate due to difficulties in establishing onset of disease; based on estimates from prevalence and incidence studies an average duration of three years was suggested (Tiemersma, 2011).

A cost-effectiveness analysis using Markov models estimated active TB progression rates from underlying latent TB on the basis of disease duration and age-dependent case-fatality rates. Base case rates for developing active TB from latent TB within 1�2 years, 3�5 years and 6�7 years of exposure were estimated at 0.74%, 0.31% (0�2.5%), and 0.16% respectively. Age-specific death rates for people aged 35, 50 and 70 years were 1%, 5% and 10% respectively (Pisu, 2009).

Based on an international TB network, the US Centers for Disease Control and Prevention (CDC) and the World Health Organization (WHO) estimated that in 2004, of 17 960 TB isolates, 20% were MDR and 2% XDR. In population-based trials in the US, Latvia and South Korea 4%, 19% and 15% of all MDR TB cases were XDR in 2004. The studies in the US and Latvia also provided additional information on the progression of MDR and XDR TB in 2004. In the US study 55% of MDR patients completed treatment/were cured and 25% died during treatment. With regard to XDR, 31% completed treatment/were cured and 23% died. Results from Latvia show the percentage of completed treatment/cases cured of MDR TB to be 69% and that of deaths/failures to be 17%. For XDR 61% completed treatment/were cured and 17% died/or had failed treatment (CDC, 2006).

Jaquet and colleagues estimated the impact of DOTS[*] in Haiti and therefore conducted a cost-effectiveness analysis with probability estimates and outcome features of TB taken from literature. For reactivation of latent TB they estimated a probability of 0.1% per year for infection present for more than two years. Within two years of a new TB infection they estimated a base case rate of 5% (2�15%) for developing TB. Cure rates of treated smear positive (drug-sensitive) TB were estimated at 62.4%. For MDR TB, authors assumed a cure rate of 48% (base case; range 48�73%) and the proportion of deaths to be 12% (base case; range 12�26%) (Jacquet, 2006).

Outcome tree parameters

Given the changes in TB epidemiology in Europe during recent decades, the situation has not been sufficiently stable to enable incidence of infection to be estimated from active TB case data. It was therefore decided not to consider latent TB in the model.

Duration of symptomatic TB is set to 0.2�2 years, irrespective of whether it is active, MDR or XDR TB (WHO, 2014).

The case fatality proportion for active TB cases is estimated to be 43% in cases not on TB treatment (Corbett, 2003; Tiemersma, 2011) and 3% in cases on TB treatment (Straetemans, 2011). Given that the estimated incidence of active TB (non-MDR or XDR) in EU/EEA is 10% higher than the notification rate (ECDC/WHO, 2015) and, assuming that all notified cases are being treated, the CFP of active TB (non-MDR or XDR) cases was set at 7%.

The case fatality proportion for MDR TB was set at 12.8% (2.3�23.3%) (Straetemans, 2011). Given the lack of evidence on the case fatality ratio for XDR TB, we used the treatment outcome result category �Died�, notified in the EU/EEA, as a proxy for estimating the XDR TB case fatality proportion and set the value at 27% (ECDC/WHO, 2015).

Model input summary

Table 1. Transition probabilities and distributions used in the outcome tree

Health outcome<br>(Health state)	Distribution of health states in health outcome	Transition probability	Source/assumption
Active TB<br>(Remaining active cases)<br>(MDR, non-XDR)<br>(XDR)	94.86%<br>4.5%<br>0.64%		ECDC/WHO, 2015
Fatal cases following remaining active cases		7%	Modelled based on Corbett, 2003; Tiemersma, 2011; Straetemans, 2011
Fatal cases following MDR TB		12.8% (2.3�23.3%)	Straetemans, 2011
Fatal cases following XDR TB		27%	ECDC/WHO, 2015

Table 2. Disability weights and duration

Health outcome<br>(Health state)	Disability Weight (DW) (Haagsma, 2015)		Duration	
	DW	Label	In years	Source
Active TB<br>(Remaining active cases)<br>(MDR, non-XDR)<br>(XDR)	0.308 (0.264�0.353)<br>0.308 (0.264�0.353)<br>0.308 (0.264�0.353)	Tuberculosis, not HIV infected<br>Tuberculosis, not HIV infected<br>Tuberculosis, not HIV infected	2<br>2<br>2	WHO, 2013<br>WHO, 2013<br>WHO, 2013

References

Berg G. Statistik �ber die Tuberkulosemortalit�t in Kriegszeiten. Beitr�ge zur Klinik der Tuberkulose 1951, 106:1-9.

Castillo-Chavez C, Feng Z. To treat or not to treat: the case of tuberculosis. J Math Biol 1997, 35:629-656.

Centers for Disease Control and Prevention: Emergence of Mycobacterium tuberculosis with extensive resistance to second-line drugs - worldwide, 2000�2004. MMWR Morb Mortal Wkly Rep 2006, 55:301-305.

Comstock G, O'Brian R. Tuberculosis. In Bacterial Infections in Humans - Epidemiology and Control. Volume 3rd edition. Edited by Evans A, Brachman P. New York: Plenum Publishing Corporation; 1998: 777-804

Corbett EL, Watt CJ, Walker N, Maher D, Williams BG, et al. (2003) The growing burden of tuberculosis: global trends and interactions with the HIV epidemic. Arch Intern Med 163: 1009-1021.

Dye C, Scheele S, Dolin P, Pathania V, Raviglione M (for the WHO Global Surveillance Monitoring Project). Global burden of tuberculosis: Estimated incidence, prevalence, and mortality by country. JAMA 1999, 282:677-686.

European Centre for Disease Prevention and Control/WHO Regional Office for Europe. Tuberculosis surveillance and monitoring in Europe 2015.

European Centre for Disease Prevention and Control, WHO Regional Office for Europe: Tuberculosis surveillance and monitoring in Europe. In: Tuberculosis surveillance and monitoring in Europe. Stockholm: European Centre for Disease Prevention and Control; 2012.

Fitzgerald D, Haas D. Mycobacterium tuberculosis. In: Principles and Practice of Infectious Diseases. Sixth edition. Edited by Mandell G, Bennett J, Dolin R. Philadelphia: Elsevier Churchill Livingstone; 2005: 2852-2886

Gideon HP, Flynn JL. Latent tuberculosis: what the host "sees"? Immunol Res 2011, 50:202-212.

Global Tuberculosis Report 2013. 1.Tuberculosis � epidemiology. 2.Tuberculosis, Pulmonary � prevention and control. 3.Tuberculosis � economics. 4.Tuberculosis, Multidrug-Resistant. 5. Annual reports. World Health Organization. ISBN 978 92 4 156465 6. Available at: http://apps.who.int/iris/bitstream/10665/91355/1/9789241564656_eng.pdf

Global Tuberculosis Report 2014. 1.Tuberculosis � epidemiology. 2.Tuberculosis, Pulmonary � prevention and control. 3.Tuberculosis � economics. 4.Tuberculosis, Multidrug-Resistant. 5. Annual reports. World Health Organization. ISBN 978 92 4 156480 9. Available at: http://www.who.int/tb/publications/global_report/gtbr14_main_text.pdf?ua=1

Haagsma JA, Maertens de Noordhout C, Polinder S, Vos T, Havelaar AH, Cassini A, Devleesschauwer B, Kretzschmar ME, Speybroeck N, Salomon JA. Assessing disability weights based on the responses of 30,660 people from four European countries. Population Health Metrics 2015; 13: 10

Jacquet V, Morose W, Schwartzman K, Oxlade O, Barr G, Grimard F, et al. Impact of DOTS expansion on tuberculosis related outcomes and costs in Haiti. BMC Public Health 2006, 6:209.

Jassal M, Bishai WR. Extensively drug-resistant tuberculosis. Lancet Infect Dis 2009, 9:19-30.

Lin PL, Flynn JL. Understanding Latent Tuberculosis: A Moving Target. The Journal of Immunology 2010, 185:15-22.

Murray CJL, Lopez AD, World Health Organization, World Bank, Harvard School of Public Health. The global burden of disease: a comprehensive assessment of mortality and disability from diseases, injuries, and risk factors in 1990 and projected to 2020. Geneva: World Health Organization; 1996.

North RJ, Jung YJ. Immunity to tuberculosis. Annu Rev Immunol 2004, 22:599-623.

Pisu M, Gerald J, Shamiyeh JE, Bailey WC, Gerald LB. Targeted tuberculosis contact investigation saves money without sacrificing health. J Public Health Manag Pract 2009, 15:319-327.

Raviglione M. Tuberculosis - the essentials. New York: Informa Health Care; 2010.

Straetemans M, Glaziou P, Bierrenbach AL, Sismanidis C, van der Werf MJ. Assessing tuberculosis case fatality ratio: a meta-analysis. PLoS One 2011, 6:e20755.

Tiemersma EW, van der Werf MJ, Borgdorff MW, Williams BG, Nagelkerke NJ. Natural history of tuberculosis: duration and fatality of untreated pulmonary tuberculosis in HIV negative patients: a systematic review. PLoS One 2011, 6:e17601.

Tseng CL, Oxlade O, Menzies D, Aspler A, Schwartzman K. Cost-effectiveness of novel vaccines for tuberculosis control: a decision analysis study. BMC Public Health 2011, 11:55.

World Health Organization: Global tuberculosis control: epidemiology, planning, financing. WHO report 2009. Geneva: World Health Organization; 2009.

Variant Creutzfeldt-Jakob disease (vCJD)

The initial symptoms of variant Creutzfeldt-Jakob disease (vCJD) are usually psychiatric, most frequently depression, anxiety and withdrawal (Henry & Knight, 2002; Will & Ward, 2004). After a median of six months, neurological features develop, including cognitive impairment, ataxia and involuntary movements. The clinical course is progressive with the development of dementia and diffuse cortical deficits.

Death occurs after a median of 14 months from the onset of symptoms (range 6–39 months) and is often due to an intercurrent infection (Will & Ward, 2004). However, Henry and Knight stated that the disease is fatal after a median of 13 months and a range of 6–39 months (Henry & Knight, 2002).

In the study by Hilton (Hilton, 2006) the mean age at death for vCJD is 26 years and 29 years with a range of 12–74 years (Will & Ward, 2004; Smiths, 2004). This is in line with the overall median age of 28 at death for all vCJD diagnoses in the UK during the period January 1994– December 2009, with a range from 14 to 75 (Andrews, 2010). During the epidemic, the median age of onset did not change over time, suggesting an important age-related risk. This could be due to an age-dependent susceptibility, age-related exposure or both (Hilton, 2006). There is no significant difference in deaths between males and females (56% male, p=0.12).

Precise estimates of the length and variability of the incubation period for vCJD are difficult to obtain since they require knowledge of the time of infection, whereas exposure may have occurred over several years. Ghani assumes that the incubation period is approximately 15–18 years (Ghani, 2002), whereas Collinge concludes that the incubation period would be at least 11 years (Collinge, 1999).

Although a peak has passed, it is possible that there will be future peaks, possibly in other genetic groups. To date, all cases of vCJD have been genotyped as methionine homozygous at codon 129 of the PrP gene (about 40% of the population). If the other 60% of the population is not completely resistant to infection, the disease in these individuals is associated with a longer incubation period, therefore epidemics in this group may still occur (Smith, 2004). Kaski et al. reported the first suspected clinical case of vCJD in an individual heterozygous for methionine/valine (Kaski, 2009).

There is also the possibility of ongoing person-to-person transmission, as seen with three cases of vCJD infection following transfusion of packed red blood cells from asymptomatic donors who subsequently died from vCJD (Ironside, 2010). Furthermore, Peden et al. described a vCJD infection in the first known asymptomatic patient (Millar, 2010; Peden, 2010). The patient died from unrelated pathology with no evidence of neurological diseases. The infection was detected in a study of autopsy and biopsy materials from 17 neurologically asymptomatic patients with haemophilia, considered to be at increased risk of vCJD. The most likely route of infection was receipt of UK plasma products.

Finally, Smith assumes that the ascertainment of vCJD cases in young adults is nearly complete. In the absence of a reliable, minimally invasive, diagnostic test, the possibility remains that cases in the elderly are being missed due to the small proportion of those dying with dementia that are subject to post-mortem examination (Smiths, 2004).

Model input summary

Table 1. Transition probabilities and distributions used in the outcome tree

Health outcome<br><br>(Health state)	Percent of health<br><br>outcome in health state	Transition probability	Source/assumption
Fatal cases following symptomatic infection		100%	

Table 2. Disability weights and duration

Health outcome (Health state)	Disability Weight (DW) (Haagsma, 2015)		Duration	
	DW	Label	In years	Source
Symptomatic infection	0.407 (0.36–0.46)	Motor plus cognitive impairments, severe.	1.151 (0.5–3.205)	Will & Ward, 2004

References

Ang LW, Chua LT, James L, Goh KT. Epidemiological surveillance and control of rubella in Singapore, 1991-2007. Ann Acad Med Singapore. 2010 Feb;39(2):95-101.

Andrews NJ. Incidence of variant Creutzfeldt-Jakob disease diagnoses and deaths in the UK, January 1994 - December 2009. 2010 [updated 22 June 2010]; Available from: http://www.cjd.ed.ac.uk/cjdq64jun10.pdf.

Collinge J. Variant Creutzfeldt-Jakob disease. Lancet. 1999 Jul 24;354(9175):317-23.

Ghani AC. The epidemiology of variant Creutzfeldt-Jakob disease in Europe. Microbes Infect. 2002 Mar;4(3):385-93.

Haagsma JA, Maertens de Noordhout C, Polinder S, Vos T, Havelaar AH, Cassini A, Devleesschauwer B, Kretzschmar ME, Speybroeck N, Salomon JA. Assessing disability weights based on the responses of 30,660 people from four European countries. Population Health Metrics 2015; 13: 10

Henry C, Knight R. Clinical features of variant Creutzfeldt-Jakob disease. Rev Med Virol. 2002 May-Jun;12(3):143-50.

Hilton DA. Pathogenesis and prevalence of variant Creutzfeldt-Jakob disease. J Pathol. 2006 Jan;208(2):134-41.

Ironside JW. Variant Creutzfeldt-Jakob disease. Haemophilia. 2010 Jul;16 Suppl 5:175-80.

Kaski D, Mead S, Hyare H, Cooper S, Jampana R, Overell J, et al. Variant CJD in an individual heterozygous for PRNP codon 129. Lancet. 2009 Dec 19;374(9707):2128.

Millar CM, Connor N, Dolan G, Lee CA, Makris M, Wilde J, et al. Risk reduction strategies for variant Creutzfeldt-Jakob disease transmission by UK plasma products and their impact on patients with inherited bleeding disorders. Haemophilia. 2010 Mar;16(2):305-15.

Peden A, McCardle L, Head MW, Love S, Ward HJ, Cousens SN, et al. Variant CJD infection in the spleen of a neurologically asymptomatic UK adult patient with haemophilia. Haemophilia. 2010 Mar;16(2):296-304.

Smith PG, Cousens SN, d' Huillard Aignaux JN, Ward HJ, Will RG. The epidemiology of variant Creutzfeldt-Jakob disease. Curr Top Microbiol Immunol. 2004;284:161-91.

Will RG, Ward HJ. Clinical features of variant Creutzfeldt-Jakob disease. Curr Top Microbiol Immunol. 2004;284:121-32.

Campylobacteriosis

Acute gastroenteritis associated with Campylobacter infections in humans is in most cases self-limiting after a few days to weeks, but for some patients the disease may be fatal. When available, information on duration of illness mainly relates to cases having requested medical help. These cases are often the most severe cases of longer duration. For example, the overall mean duration of illness due to Campylobacter

infection observed in the GP[*] case control component of the IID study in England and Wales was 9.34 days, whereas it was only 6.52 days for all cases observed in the community component (Adak, 2002). About 47% of the community component cases would have visited their GP (Food Standards Agency, 2000). Based on the IID study, it is has been assumed that gastroenteritis caused by campylobacteriosis would last 3.22 days (no medical help), 9.72 days (visiting GP) and 14.39 days (hospitalised) (Mangen, 2004; Mangen, 2005). In our current model we chose to apply 3.22–9.72 days for all uncomplicated cases and 14.39 days for the complicated ones.

Bacteraemia is highlighted in many reports as a possible extra-intestinal complication of campylobacteriosis. For example, Skirrow et al. (Skirrow, 1993) estimated a bacteraemia incidence of 1.5 per 1 000 reported campylobacteriosis cases, whereas Ternhag et al. (Ternhag, 2008) reported an absolute risk of bacteraemia/sepsis of 0.02% for laboratory-confirmed campylobacteriosis cases.

Assuming that GP visits represent an indication of moderate diarrhoea and that the proportion of hospitalised cases represents severe diarrhoea, we divided cases into the following groups: uncomplicated (mild diarrhoea) 75.5%, complicated (GP, moderate diarrhoea) 23.5% and complicated hospitalised (severe diarrhoea) cases 1% (Kemmeren, 2006; Kwong, 2012; redistributing to total 100%).

Estimates of campylobacteriosis case fatality proportions range from 0.001% to 0.05%: 0.05% (Mead, 1999), 0.024% of all foodborne campylobacteriosis cases in the IID study (Adak, 2002), 2–6% of the hospitalised cases (Buzby, 1996; corresponding to 0.012–0.036% of all cases, considering that 0.6% of cases are hospitalised according to Mangen et al. 2004), 1.3 fatal cases per year, corresponding to 0.001% of the estimated 123 000 Campylobacter cases (Cressey & Lake, 2007), 0.038% of all symptomatic cases (Mangen, 2004).

We chose to estimate the overall case fatality proportion as being within the range 0.001–0.05% and assumed a different age-group distribution of this risk based on the age-group distribution of reported deaths to TESSy between 2009 and 2013 (see Table 3). This table is based on all TESSy notified cases from EU Member States except Bulgaria, reporting only aggregate data, Greece, Portugal and Liechtenstein which do not report.

Risk of complications

Reactive arthritis (ReA), irritable bowel syndrome (IBS) (but not inflammatory bowel disease due to lack of confirmation of a biological link and limited evidence) and Guillain-Barré syndrome (GBS) may be associated with campylobacteriosis.

Reactive arthritis (ReA)

ReA is a significant long-term sequelae following campylobacteriosis (Keat, 1983; Johnsen, 1983; Hannu, 2002). A retrospective study carried out in Finland found that 7.4% (45/609) of laboratory-confirmed campylobacteriosis cases fulfilled the criteria for ReA (Hannu, 2002), which is similar to that found by another study: 8.1% (3/37) (Johnsen, 1983). A further study reported a 2.6% (9 of 350) frequency of ReA in patients contacting a municipal health centre following an outbreak of C. jejuni (Hannu, 2004) and 16% of laboratory-confirmed cases self-reported having had ReA (Locht & Krogfeld, 2002), although self-reporting might be prone to overestimation (Hannu, 2002). Other studies including clinical testing report a 2.8% and a 2.4% risk of developing rheumatological symptoms (Rees, 2004; Kosunen, 1980). In order to account for the large uncertainty, the risk of developing ReA from all symptomatic cases is 1.7% (0.73–4.4%) (Kemmeren, 2006).

Little is known about the duration of ReA; the average duration is between 1.5 months derived from Hannu et al. (Hannu, 2005) and 222 days (Kemmeren, 2006).

Irritable Bowel Syndrome (IBS)

In a recent literature review, 8.8% (7.2–10.4%) of symptomatic campylobacteriosis symptomatic cases were considered at risk of developing IBS, irrespective of age and gender; the duration was set to five years (Haagsma, 2010). However, the causality is largely debated and the impact of concurrent factors significant. Therefore, IBS is not considered as part of the campylobacteriosis outcome tree in our study.

Guillain-Barré syndrome (GBS)

GBS is a neurological disease frequently preceded by an acute infectious illness, mainly upper respiratory infections and gastrointestinal infections. The functional status of patients with GBS is scored on a seven-point disability scale (F-score), ranking from 0 (healthy) to 6 (death). GBS-patients with an F-score at nadir of < 3 (able to walk unaided at nadir) are considered to be mildly affected. GBS patients with an F-score of ≥ 3 (unable to walk unaided at nadir) are considered to be severely affected (van Koningsveld, 2001). Paralysis from GBS is generally reversible over time, but some patients are bedridden for life and others die prematurely.

Incidence is estimated at 0.8–2.0 or 0.4–4 cases per 100 000 persons year (van Koningsveld, 2001; Mc Grogan, 2009; Hughes & Rees, 1997) in Europe and North America. A systematic review of the literature and metanalysis estimated an age-specific GBS rate per 100 000 person years of $\exp[-12.0771 + 0.01813(\text{age in years})] \times 100\,000$ (Sejvar, 2011).

Studies show that 14–36% of GBS patients previously had a Campylobacter infection (Jacobs, 1998); 33–50% of GBS patients had increased levels of Campylobacter spp. (Mishu, 1993). A more recent systematic literature review estimated that 31% of the 2 502 GBS cases studied were attributable to Campylobacter infection (Poropatich, 2010).

Research has found that about 0.022% of laboratory-confirmed campylobacteriosis cases would develop GBS (13/57,425) (Ternhag, 2008), resulting for all symptomatic cases in a 0.0015% risk of developing GBS; in Sweden one GBS case per 3 285 Campylobacter jejuni infections (95% C.I.: 1.729 – 7.210) resulting in a risk of 0.03% (McCarthy & Giesecke, 2001); in the USA one per 1 058 campylobacteriosis cases (0.09% risk; Allos, 1997). Studies estimating the burden of campylobacteriosis assumed a 0.075% and 0.023% risk of developing GBS (Mangen, 2004 and 2005; Cressy & Lake, 2007). Given the large diversity found in the literature, the risk of developing GBS following a symptomatic Campylobacter infection is set to 0.0015–0.09%.

Males were more commonly affected by GBS in almost all studies (Sedano, 1994; Hughes & Rees, 1997; Nachamkin, 1998; Nagpal, 1999; van Koningsveld, 2000; Sejvar, 2011). However, these differences might be based on environmental factors as well as biological factors (van Koningsveld, 2000) and therefore it is difficult to speculate about the origin of this gender difference and the cause and determinants of GBS and therefore we do not distinguish in risk between genders.

Havelaar et al. (Havelaar 2000 a,b) estimated the proportion of mild and severe GBS cases after Campylobacter infections to be 17% and 83%, respectively. Age plays a role (van Koningsveld et al., 2000; Sejvar et al., 2011), we therefore assume that the age-group-specific distribution of the risk of developing a mild GBS is 17% and a severe GBS is 83% – see Table 4 and 5 (Havelaar 2000a, b). A total of 69% of mild GBS cases are under the age of 50, whereas for severe GBS cases this is only 48%.

The clinical course of GBS is highly variable. Very limited information is available for mildly affected patients. About 50% of the patients recover fully after six months, and the others have an F-score of 1. Most will recover after one year and the remainder will only suffer from minor symptoms (Havelaar, 2000a). We therefore assumed that mild cases will recover fully after one year.

There is a high heterogeneity among the severely-affected GBS patients: 60% of patients are reported to have an F-score of 4 when hospitalised, and approximately 20% of the patients had an F-score of 5 at nadir (Van der Meché, 1992). All patients recovered from intensive care, but after six months, 17% of them still had an F-score of 3 or 4. In a follow-up study the residual symptoms were evaluated up to six years after onset (Bernsen, 1997): only 25% recovered fully, whereas 44% of patients continued to suffer from minor symptoms (F-score=1) and 31% had functional limitations (F-score 2-4). Given that there had been no significant improvement since the acute phase, we assume that 17–31% of severely affected GBS patients would have permanent sequelae; this risk is distributed by age groups, see Table 6 (Havelaar 2000 a;b).

The case fatality rate for GBS ranges from 2–5% (Havelaar, 2000a) to 3.4% in a retrospective study (Van Koningsveld, 2000). However, generally only the severe cases are at risk of dying, therefore the risk is only estimated for these cases (CFR/83% severe cases x 100): 4.1% (2.41–6.02). The case fatality rate is age-dependent (Havelaar, 2000a) and strictly linked to the risk of developing permanent disabilities due to GBS; therefore, we apply the same age-group distribution as the risk of dying, see Table 6).

Model input summary

Table 1. Transition probabilities used in the outcome tree

Health outcome (Health state)	Distribution of health states in health outcome	Transition probability	Source/assumption
Symptomatic infection<br>(Uncomplicated)<br>(Complicated, GP)<br>(Complicated, hosp)	 76%<br>23%<br>1%		Kemmeren, 2006; Kwong, 2012
Fatal cases following symptomatic infection		0.001–0.05%<br>Age dep. Table 3	Adak, 2002; Cressey & Lake, 2007; Mangen, 2005; Mead, 1999; TESSy 2009-2013
Reactive arthritis		1.7% (0.73–4.4%)	Kemmeren, 2006

Guillain-Barré syndrome<br><br>(Mild)<br><br><br><br>(Severe)	 <br>17%<br><br>Age dep. Table 4<br><br><br>83%<br><br>Age dep. Table 5	0.0015–0.09%	Allos, 1987; Ternhag, 2008; Havelaar 2000a, b
Fatal cases following severe GBS		4.1% (2.41–6.02%)<br><br>Age dep. Table 6	Koningsveld, 2001; Havelaar, 2000a<br><br>Assuming only severe cases are fatal
Permanent disability following GBS		17–31%<br><br><br>Age dep. Table 6	Havelaar, 2000a, b<br><br><br>Assuming only severe cases

Table 2. Disability weights and duration

Health outcome (Health state)	Disability Weight (DW) (Haagsma, 2015)		Duration	
	DW	Label	In years	Source/assumption

Symptomatic infection<br><br>(Uncomplicated)<br>(Complicated, GP)<br>(Complicated, hosp)	 <br><br>0.073 (0.061–0.092)<br>0.149 (0.12–0.182)<br>0.239 (0.202–0.285)	 <br><br>Diarrhoea, mild<br>Diarrhoea, moderate<br>Diarrhoea, severe	 <br><br>0.009<br>0.027<br>0.039	Food Standard Agency, 2000; Mangen, 2004, 2005
Reactive arthritis	0.344 (0.3-0.391)	Musculoskeletal problems, generalized, moderate	0.131-0.608	Hannu, 2002; Kemmeren, 2006
Guillain-Barré syndrome<br>(Mild)<br>(Severe)	 <br>0.053 (0.042-0.064)<br><br>0.520 (0.465-0.581)	 <br>Motor impairment, moderate<br><br>Spinal cord lesion at neck level (treated)	 <br>1<br>1	Havelaar, 2000a, b
Permanent disability following GBS	0.421 (0.377-0.477)	Motor impairment, severe	Remaining life expectancy	Van der Meché, 1992; Bernsen, 1997

Table 3. Age-group distribution of the case fatality rate (0.001–0.05%)

Age groups	%
0	0.54
1-4	1.09
5-9	3.26
10-14	1.63
15-19	0.54
20-24	4.35
25-29	5.98
30-34	1.63
35-39	3.26
40-44	3.80
45-49	3.80
50-54	5.43

55-59	5.98
60-64	5.98
65-69	8.15
70-74	6.52
75-79	11.96
80-84	11.96
>85	14.13
All ages	100.00

Table 4. Age distribution mild GBS

Age	%
0	0.63
01-04	5.02
05-09	2.51
10-14	1.25
15-19	6.27
20-24	6.90

25-29	10.04
30-34	9.41
35-39	9.41
40-44	8.78
45-49	8.78
50-54	5.17
55-59	4.82
60-64	4.13
65-69	5.51
70-74	5.17
75-79	4.13
80-84	0.69
85+	1.38
Total	100

Table 5. Age distribution – severe GBS

Age	%
0	0.44
01-04	3.49
05-09	1.75
10-14	0.87
15-19	4.36
20-24	4.80
25-29	6.98
30-34	6.55
35-39	6.55
40-44	6.11
45-49	6.11
50-54	8.67
55-59	8.09
60-64	6.93
65-69	9.24
70-74	8.67
75-79	6.93

80-84	1.16
85+	2.31

Total	100

Table 6. Age distribution permanent GBS and case fatality rate

Age	%
0	0.00
01-04	0.00
05-09	0.00
10-14	0.00
15-19	0.00
20-24	1.56
25-29	1.56
30-34	1.56
35-39	1.56
40-44	2.08
45-49	2.08
50-54	2.08
55-59	6.25
60-64	6.25
65-69	6.25
70-74	18.75
75-79	25.00
80-84	18.75
85+	6.25
Total	100.00

References

Adak GK, Long SM, O'Brien SJ (2002). Trends in indigenous foodborne disease and deaths, England and Wales: 1992 to 2000. Gut 2002. 51:832- 841.

Allos BM. (1997). Association between Campylobacter infection and Guillain-Barré syndrome. J Infect Dis 1997.176(Suppl 2): S125-S128.

Bernsen RA, Jacobs HM, de Jager AE, van der Meché FGA (1997). Residual health status after Guillain-Barré syndrome. J. Neurol Neurosurg Psychiatry 1997. 62:637-640.

Buzby JC, Roberts T, Lin JCT, MacDonald JM (1996). Bacterial Foodborne Disease – Health care costs & Productivity losses. Washington, USA: Economic Research service, USDA. Agricultural Economic Report No 741.

Cressey P J and Lake R (2007). Risk ranking: Estimates of the burden of foodborne disease for New Zealand. Institute of Environmental Science & Research Limited; Christchurch Science Center.

Food Standards Agency (2000). A Report of the Study of Infectious Intestinal Disease in England. Food Standards Agency London: HMSO.

Haagsma JA, Havelaar AH, Janssen BM, Bonsel GJ. Disability Adjusted Life Years and minimal disease: application of a preference-based relevance criterion to rank enteric pathogens. Popul Health Metr 2008;6:7.

Haagsma JA, van der Zanden BP, Tariq L, van Pelt W, van Duynhoven YTHP, Havelaar AH (2009). Disease burden and costs of selected foodborne pathogens in the Netherlands, 2006. Bilthoven: National Institute of Public Health and Environment. RIVM rapport 330331001. Available online at: www.rivm.nl/bibliotheek/rapporten/330331001.pdf

Haagsma JA, Siersema PD, De Wit NJ, Havelaar AH. Disease burden of post-infectious irritable bowel syndrome in the Netherlands. Epidemiol Infect 2010.138(11):1650-6.

Haagsma JA, Maertens de Noordhout C, Polinder S, Vos T, Havelaar AH, Cassini A, Devleesschauwer B, Kretzschmar ME, Speybroeck N, Salomon JA. Assessing disability weights based on the responses of 30,660 people from four European countries. Population Health Metrics 2015; 13: 10

Hannu TL, Mattila H, Rautelin P, Pelkonen P, Lahdenne A, Siitonen M et al. Campylobacter-triggered reactive arthritis: a population-based study. Rheumatology 2002. 41(3):312-318.

Hannu TL, Kauppi M, Tuomala M, Laaksonen I, Klemets P, Kuusi M. Reactive arthritis following an outbreak of Campylobacter jejuni infection. J Rheumatol 2004. 31(3):528-30.

Havelaar AH, de Wit MAS, van Koningsveld R, van Kempen E (2000a). Health burden in the Netherlands due to infection with thermophilic Campylobacter spp. Epidemiol Infect 2000. 125(3):505-22.

Havelaar AH, de Wit MAS, van Koningsveld R (2000b). Health burden in the Netherlands (1990-1995) due to infection with thermophilic Campylobacter species. Bilthoven, the Netherlands: National Institute for Public Health and the Environment. Report no. 284550004.

Havelaar AH, Haagsma JA, Mangen MJJ, Kemmeren JM, Verhoef LPB, Vijgen SMC, et al. (2012a). Disease burden of foodborne pathogens in the Netherlands, 2009. International Journal of Microbiology in press.

Havelaar AH, Ivarsson S, Löfdahl M, Nauta MJ (2012b). Estimating the true incidence of campylobacteriosis and salmonellosis in the EU. Submitted.

Helms M, Simonsen J, Molbak K. Foodborne bacterial infection and hospitalization: a registry-based study. Clin Infect Dis 2006.42(4):498-506.

Hughes RAC and Rees JH (1997). Clinical and epidemiologic features of Guillain-Barré syndrome. Journal of infectious diseases 1997. 176 (suppl 2): S92-S98.

Jacobs BC, Rothbarth PH, van der Meché FG, Herbrink P, Schmitz PI, de Klerk MA, et al. (1998). The spectrum of antecedent infections in Guillain-Barré syndrome: a case-control study. Neurology 1998. 51(4):1110-1115.

Jess T, Simonsen J, Nielsen NM, Jørgensen KT, Bager P, Ethelberg S et al. (2011). Enteric Salmonella or Campylobacter infections and the risk of inflammatory bowel disease. Gut 2011;60(3):318-324.

Johnsen K, Ostensen M, Melbeye AC, Melby K (1983). HLA-B27-negative arthritis related to Campylobacter jejuni enteritis in three children and two adults. Acta Med Scand 1983. 214(2): 165-168.

Kemmeren JM, Mangen MJJ, van Duynhoven YTHP, Havelaar AH. (2006). Priority setting of foodborne pathogens: disease burden and costs of selected enteric pathogens. Bilthoven: National Institute of Public Health and Environment: RIVM rapport 330080001. Available online at: www.rivm.nl/bibliotheek/rapporten/330080001.pdf

Keat A. (1983). Reiter's syndrome and reactive arthritis in perspective. N Engl J Med 1983. 309(26):1606-15.

Kosunen TU, Kauranen O, Martio J, Pitkanen T, Ponka A, Hortling L, et al. (1980). Reactive arthritis after campylobacter jejuni enteritis in patients with HLA-B27. Lancet 1980. 1 (8181):1312-3.

Kwong JC, Ratnasingham S, Campitelli MA, Daneman N, Deeks SL, et al. (2012) The Impact of Infection on Population Health: Results of the Ontario Burden of Infectious Diseases Study. PLoS ONE 7(9): e44103. doi:10.1371/journal.pone.0044103

Lake RJ, Cressey PJ, Campbell DM, Oakley E (2010). Risk ranking for foodborne microbial hazards in New Zealand: burden of disease estimates. Risk Analysis 2010. 30(5):743-52.

Locht H, Krogfelt KA (2002). Comparison of rheumatological and gastrointestinal symptoms after infection with Campylobacter jejuni/coli and enterotoxigenic Escherichia coli. Ann Rheum Dis 2002.61(5): 448-52.

Mangen MJJ, Havelaar AH, de Wit GA. (2004). Campylobacteriosis and sequelae in the Netherlands - Estimating the disease burden and the cost-of-illness. Bilthoven: National Institute for Public Health and the Environment; Report nr. 250911004. Available online at: www.rivm.nl/bibliotheek/rapporten/250911004.pdf

Mangen MJJ, Havelaar AH, Bernsen RAJAM, van Koningsveld R, de Wit GA (2005). The costs of human Campylobacter infections and sequelae in the Netherlands: A DALY and cost-of-illness approach. 2005. Acta Agriculturae Scandinavica Section C Food Economics 2005. 2(1):35-51.

McCarthy N, Giesecke J (2001). Incidence of Guillain-Barre syndrome following infection with Campylobacter jejuni. Am J Epidemiol 2001. 153 (6): 610-614.

McGrogan A, Madle GC, Seaman HE, de Vries CS (2009). The epidemiology of Guillain-Barré syndrome worldwide. A systematic literature review. Neuroepidemiology 2009. 32(2):150-163.

Mead PS, Slutsker L, Dietz V, McCaig LF, Bresee JF, Shapiro C, et al. (1999). Food-related illness and death in the United States. Emerg Infect Dis 1999.5(5): 607-625.

Melse JM, Kramers PGN (1998). Berekening van de ziektelast in Nederland. Achtergronddocument bij VTV-1997; deel III, hoofdstuk 7. Bilthoven; Report nr. 431501028.

Mishu B, Ilyas AA, Koski CL, Vriesendorp F, Cook SD, Mithen FA, et al. (1993). Serological evidence of previous Campylobacter jejuni infection in patients with the Guillain-Barré Syndrome. Ann Intern Med 1993. 118(12): 947-953.

Nachamkin I, Allos BM, Ho T. (1998). Campylobacter species and Guillain-Barré syndrome. Clin Microbiol Rev 1998. 11(3): 555-567.

Nagpal S, Benstead T, Shumak K, Rock G, Brown M, Anderson DR (1999). Treatment of Guillain-Barré syndrome: a cost-effectiveness analysis. J. Clin Apheresis 1999. 14(3):107-113.

Poropatich KO, Walker CL, Black RE (2010). Quantifying the association between Campylobacter infection and Guillain-Barré syndrome: a systematic review. J. Health Popul Nutr; 2010. 28(6):545-52.

Sedano MJ, Calleja J, Canga E, Berciano J (1994). Guillain-Barré syndrome in Cantabria, Spain. An epidemiological and clinical study. Acta Neurol Scand 89(4): 287-292.

Sejvar JJ, Baughman AL, Wise M, Morgan OW (2011). Population incidence of Guillain-Barré syndrome: a systematic review and meta-analysis. Neuroepidemiology; 2011 36(2):123-133.

Skirrow MB, Jones DM, Sutcliffe E, Benjamin J (1993). Campylobacter bacteraemia in England and Wales, 191-91. Epidemiology and Infection 110 (3): 49-57.

Tam CC, Rodrigues IC, Viviani L, Dodds JP, Evans MR, Hunter PR et al. Longitudinal study of infectious intestinal disease in the UK (IID2 study): incidence in the community and presenting to general practice. Gut. 2012 Jan;61(1):69-77

Ternhag A, Törner A, Svensson A, Ekdahl K, Giesecke J (2008). Short- and long-term effects of bacterial gastrointestinal infections. Emerg Infect Dis 2008.14 (1):143-8.

Van der Meché FGA, Schmitz PIM and Dutch Guillain-Barré Study Group (1992). A randomized trial comparing intravenous immune globulin and plasma exchange in Guillain-Barré syndrome.N Engl. J. Med. 1992 326:1123-1129.

Van Koningsveld R, van Doorn PA, Schmitz PIM, Ang CW, van der Meché FGA (2000). Mild forms of Guillain-Barré syndrome in an epidemiological survey in the Netherlands. Neurology 54: 620-625.

Van Koningsveld R. (2001). Epidemiological and clinical aspects of the Guillain-Barré syndrome. Rotterdam, the Netherlands: Erasmus University Rotterdam. PhD-Thesis.

WHO (2004). Global Burden of Disease 2004 Update: Disability weights for diseases and conditions. Geneva: WHO. Available online at: http://www.who.int/healthinfo/global_burden_disease/GBD2004_DisabilityWeights.pdf

Yu DTY, Thomson GTD (1994). Clinical, epidemiological and pathogenic aspects of reactive arthritis. Food-Microbiology 1994. 11(2):97-108.

Chlamydia

Chlamydia trachomatis is a bacterium that causes a sexually transmitted infection (STI). WHO estimates a global annual incidence of about 90 million cases. Chlamydia trachomatis affects both women and men and can cause severe harm to the reproductive system of women. Additionally, children born to infected mothers are at high risk of developing severe complications (e.g. ophthalmia neonatorum, pneumonia). C. trachomatis has various serovars with different transmission modes and consequences. Serovars A, B, Ba and C, often transmitted by close eye- to-eye contact, cause ocular trachoma and are responsible for about 7–9 million cases of blindness (Stamm, 2005). Serovars D–K, responsible for genital infections, are associated with various adverse health outcomes in both men and women (Carey & Beagley, 2010). Serovars L1, L2 and L3 cause Lymphogranuloma venerum, a systemic STI mainly observed in the high-risk group of men having sex with men (MSM) (Martin- Iguacel, 2010). For the current outcome trees only serovars D–K responsible for genital infection are taken into consideration.

C. trachomatis mostly affects the young and sexually-active population with a female-male sex ratio of 1:0.7 (in tested individuals) (ECDC, 2014a). The genito-urinary infections present different disease patterns in the female and male hosts.

The asymptomatic infection poses serious threats to the health of the population as asymptomatic carriers represent a pool for new infections, and asymptomatic infections are associated with the risk of developing severe sequelae.

Rates of asymptomatic cases reported in literature vary widely. More than 50% of the infections due to C. trachomatis in males do not produce any symptoms or present a mild symptomatic illness (van de Laar & Morre, 2007). In a study of male army recruits, 85.6% of men testing positive for Chlamydia reported no symptoms (Cecil, 2001). Comparable rates were also reported by McKay and colleagues, with 88% of infected men being asymptomatic (McKay, 2003). Long-term sequelae due to chronic asymptomatic infections in men are still under discussion, but the pool of asymptomatic C. trachomatis carriers poses a serious threat to women's health due to continuous transmission and re-infection. Gaydos and Quinn refer to a percentage of asymptomatic male cases above 50%, in line with the above-mentioned estimates (Gaydos & Quinn, 2012).

Genital infections in women may present with short-term acute symptoms of cervicitis and urethritis (Stamm, 2005). Women also face a high number of asymptomatic infections. In total, 70–90% of all female and 50–88% of all male chlamydial infections do not present any symptoms (Stamm, 2005; Gaydos, 1998; Kalwij, 2010). Quinn and colleagues noted that around 79% of women with a Chlamydia infection attending a STI clinic were asymptomatic (Quinn, 1996). Clinical textbooks report a range of 70–90% of female cases being asymptomatic (Stamm, 2005; Gaydos & Quinn, 2012).

For the model we decided to use a range of 70–90% for the asymptomatic proportion (Stamm, 2005; Gaydos & Quinn, 2012) for female and 50– 88% for male cases (Stamm, 2005; Gaydos, 1998; Kalwij, 2010).

Health outcomes associated with chlamydial infection

Genital infection in men

Urethritis: with an incubation period of 7–14 days, urethritis causes symptoms of dysuria and urethral discharge (Stamm, 2005). We selected a range of 12–50% of infected men to represent symptomatic cases developing non-gonococcal urethritis (NGU) (Carey & Beagley, 2010; McKay, 2003).

Epididymitis: epididymitis is an acute inflammation of the epididymis (Carey & Beagley, 2010). The symptoms are oligospermia during the acute phase, swollen epididymis (and/or testicles), and dysuria. Fever and chills may occur in some cases. The association between epididymitis and future (in)fertility is an ongoing debate in research with no clear indication (Stamm, 2005).

Proctitis and proctocolitis: this clinical picture is most common in the MSM community. The classic symptoms are rectal pruritus, -pain and - bleeding. Fever often accompanies the initial proctitis and proctocolitis (Stamm, 2005; Carey & Beagley, 2010). This health outcome was not considered in the model due to lack of information.

Reactive arthritis: a further clinical picture is sexually-acquired reactive arthritis occurring as an acute aseptic arthritis or presenting as Reiter's syndrome. Reiter's syndrome includes symptoms of arthritis, conjunctivitis, urethritis and skin lesions (Stamm, 2005; Keat, 1983).

Genital infection in men can also include chronic pelvic pain. However, due to lack of information we decided not to include it in the model (Haggerty, 2010).

Genital infection in women

Urethritis/cervicitis

The acute form of C. trachomatis infection in women is urethritis and/or cervicitis. The majority of cases of both urethritis and cervicitis are asymptomatic, but can lead to severe sequelae (Low, 2007).

Pelvic inflammatory disease (PID)

Both symptomatic and asymptomatic infections can lead to serious consequences. Pelvic inflammatory disease is a commonly reported health outcome of a chlamydial infection. The literature shows very heterogeneous patterns regarding the transition probabilities from acute infection to PID. Carey and Beagley state that 12–50% of women infected with C. trachomatis develop PID (Carey & Beagley, 2010). In other literature the risk of PID after lower genital tract infection with Chlamydia varied from 0 to 30% (Risser & Risser, 2007) and from 0 to 72% (Boeke, 2005). Cates and Wasserheit reported that 40% of women with an untreated C. trachomatis infection develop PID (Cates & Wasserheit, 1991). Van Valkengoed and colleagues reported that complications of Chlamydia trachomatis infections are overestimated in the literature. They found five Cost Effectiveness Analyses (CEA) using decision trees to estimate the effect of screening programmes (Van Valkengoed, 2004). In these studies the estimates of the probability of developing PID after infection varied from 25 to 80%. ECDC has undertaken a systematic literature review and found a risk of developing PID from chlamydial infections of 9% (4–19%) (ECDC, 2014b).

Acute PID with pelvic pain, lasting for about 15 days, and silent PID with no or mild symptoms can cause severe long-term sequelae (Carey & Beagley, 2010; Westrom, 1980).

The estimated risk of tubal infertility as a sequelae of PID varies between 10–20% (Carey & Beagley, 2010; Lan, 1995; Land, 2010). Land and colleagues estimated the risk of tubal infertility after asymptomatic Chlamydia infection to be around 0.07% (Land, 2010). The risk of tubal infertility was found to be dependent on the course of infection (mild vs. severe) and the frequencies of re-infection (e.g. after three episodes of PID the risk is five-fold compared to a single episode.) ECDC's systematic review found that 16% of women with PID will develop infertility (ECDC, 2014b), which applies to women of reproductive age.

In total, 7–9% of pregnant women develop ectopic pregnancy after PID (Lan, 1995). Around 15% of women with previous PID develop chronic pelvic pain (Rogstad, 2008). Tubo-ovarian abscesses (tubal pathology) incur a risk of 7–16% for women who have previously had PID (Kottmann, 1995). The risk of cervical neoplasia is still under debate due to the fact that most cervical neoplasia are due to human papilloma virus (HPV) (Stamm, 2005).

Based on registration data from Amsterdam it was estimated that 0.07% and 0.02% of women exposed to chlamydia infection develop ectopic pregnancy and tubal factor infertility, respectively (Van Valkengoed, 2004).

Perinatal infections

Perinatal chlamydia may complicate as conjunctivitis (ophthalmia neonatorum) and neonatal pneumonia. We considered the ONBoID study for the input parameters which estimated that 15% of cases would develop ophthalmia neonatorum and 16% neonatal pneumonia (Kwong 2012). Assuming that in EU/EEA Member States all notified cases will have had symptoms, we used the same proportion: 48.39% are affected by ophthalmia and 51.61% will present pneumonia.

Outcome-tree parameters

Male outcome tree

For the male outcome tree a minimum of 50% and maximum of 88% was estimated as the percentage of asymptomatic cases (Carey & Beagley, 2010; McKay, 2003). The probability of developing epididymitis from symptomatic infections (10%) was taken from the World Health Organization STD Burden of Disease Study by Gerbase and colleagues (Gerbase, 2000). For asymptomatic infections a probability of 1–4% was taken from the cost effectiveness analysis of Welte and colleagues (Welte, 2001). Data on sexually acquired reactive arthritis (1% of symptomatic urethritis) and the resulting Reiters' syndrome (33% of reactive arthritis) were taken from a clinical text book (Stamm, 2005).

Female outcome tree

For the percentage of asymptomatic cases a range of 70–90% was included in the model (Stamm, 2005; Gaydos & Quinn, 2012; Gaydos, 1998; Kalwij, 2010; Stamm, 1999).

For the development of PID, estimates are included from the systematic review conducted by ECDC for the minimum (4%) (Van Valkengoed, 2004), maximum (19%) and most likely values (9%) (ECDC, 2014b).

The probability of developing ectopic pregnancy and tubal infertility after chlamydia infection is set to 0.07% and 0.02% respectively (Van Valkengoed, 2004). The probability of dying due to ectopic pregnancy was set to 0.038%, based on the study from Goldner (Goldner, 1993).

The risk of moving from PID to chronic pelvic pain was set at 18–75% and from PID to tubo-ovarian abscess at 0.8% (ECDC, 2014b; Ness, 2002, Soper 2010).

We decided to set the case fatality proportion for abscesses that have not ruptured to zero. Current mortality proportions for patients with ruptured abscesses are not reported in the literature; data from the 1960s suggested a mortality proportion ranging from 1.7 to 3.7 percent (Pedowitz, 2004; Paik, 2006). Due to the fact that these figures come from old studies and that diagnostics and treatment have significantly improved, we decided not to include the risk of dying from tubo-ovarian abscess.

Model input summary

Table 1. Transition probabilities and distributions used in the outcome tree

Health outcome (Health state)	Distribution of health states within health outcome	Transition probability	Source/assumption
Men			
Symptomatic infection		12–50%	Carey & Beagley, 2010; McKay, 2003
Epididymitis following symptomatic infection		10%	Gerbase, 2000
Reactive arthritis		1%	Stamm, 2005
(Mild)	67%		Stamm, 2005
(Severe)	33%		Stamm, 2005
Epididymitis following asymptomatic infection		1–4%	Gerbase, 2000; Welte, 2001

Women<br><br>Symptomatic infection		10–30%	Stamm, 1999; Stamm, 2005; Gaydos & Quinn, 2012; Gaydos, 1998; Kalwij, 2010
Pelvic inflammatory disease (PID)		9% (4–19%)	ECDC, 2014b
Tubo-ovarian abscess from PID		0.8%	Ness, 2002
Chronic pelvic pain after PID		18–75%	ECDC, 2014b; Soper 2010
Ectopic pregnancy		0.07%<br><br>Age dep. See Table 4	van Valkengoed, 2004<br><br>Female reproductive age 15-49
Tubal Infertility		0.02%<br><br>Age dep. See Table 4	Land, 2010; ECDC, 2014b<br><br>Female reproductive age 15-49
Fatal cases following ectopic pregnancy		0.038%	Goldner, 1993
Perinatal			
Symptomatic infection<br><br>(Neonatal pneumonia) (Ophthalmia neonatorum)	48.39%<br><br>51.61%		Kwong, 2012<br><br>Assuming that all reported cases have symptoms, we used the same proportion

Table 2. Disability weights and duration

Health outcome<br>(Health state)	Disability Weight (DW) (Haagsma, 2015)		Duration	
	DW	ECDC European Disability Weight Project (2014)	In years	Source

Men				
Symptomatic infection	0.007 (0.005–0.01)	Infectious disease, acute episode, mild	0.02	Trojian, 2009
Epididymitis	0.176 (0.143–0.208)	Epididymo-orchitis	0.04	Murray, 1996
Reactive arthritis<br>(Mild)<br><br><br><br>(Severe)	 0.344 (0.3-0.391)<br><br><br><br>0.518 (0.457–0.576)	 Musculoskeletal problems, generalised, moderate<br>Musculoskeletal problems, generalised, severe	 0.13–0.28<br><br><br>0.41	 Özgül, 2006; Hannu, 2002<br><br><br><br>Miehle, 2003
Women				
Symptomatic infection	0.007 (0.005–0.01)	Infectious disease, acute episode, mild	0.03	Murray, 1996
Pelvic inflammatory disease (PID)	0.018–0.310	Abdominopelvic problem, mild to severe	0.04	Westrom, 1980
Tubo-ovarian abscess	0.31 (0.262–0.355)	Abdominopelvic problem, severe	0.01	Goharkhay, 2007; Teisala, 1990
Chronic pelvic pain	0.018–0.123	Abdominopelvic problem, mild to moderate	2.8	Sharma, 2011
Ectopic pregnancy	0.31 (0.262–0.355)	Abdominopelvic problem, severe	0.08	Murray, 1996
Tubal infertility	0.007 (0.005–0.01)	Infertility, secondary	See Table 3	Female reproductive age 15-49 (See Table 4)

Perinatal				
Neonatal pneumonia	0.125 (0.104–0.152)	Infectious disease, acute episode, severe	0.038	Zar, 2005<br><br>Assuming two weeks of treatment
Ophthalmia neonatorum	0.015 (0.011–0.019)	Conjunctivitis without corneal scar	0.038	American Academy of Pediatrics, 2012.<br><br>Assuming two weeks of treatment

Table 3. Duration of tubal infertility (female outcome tree)

Age	Duration in years
15–19	32
20–24	27
25–29	22
30–34	17
35–39	12
40–44	7
45–49	2

Table 4. Age-group risk (only reproductive age)

Age	%
0–14	0
15–49	100
≥50	0

References

American Academy of Pediatrics. Chlamydia trachomatis. In: Pickering LK, editor. Red book: 2012. Report of the Committee on Infectious Diseases. Elk Grove Village, IL: American Academy of Pediatrics; 2012. pp. 276–81.

Boeke AJ, van Bergen JE, Morre SA, van Everdingen JJ: [The risk of pelvic inflammatory disease associated with urogenital infection with Chlamydia trachomatis; literature review]. Ned Tijdschr Geneeskd 2005, 149:878-884.

Carey AJ, Beagley KW: Chlamydia trachomatis, a hidden epidemic: effects on female reproduction and options for treatment. Am J Reprod Immunol 2010, 63:576-586.

Cates W Jr. Wasserheit JN: Genital chlamydial infections: epidemiology and reproductive sequelae. Am J Obstet Gynecol 1991, 164:1771-1781.

Cecil JA, Howell MR, Tawes JJ, Gaydos JC, McKee KT, Quinn TC, et al. Features of Chlamydia trachomatis and Neisseria gonorrhoeae Infection in Male Army Recruits. Journal of Infectious Diseases 2001, 184:1216-1219.

European Centre for Disease Prevention and Control. Sexually transmitted infections in Europe 2012: ECDC; 2014a.

European Centre for Disease Prevention and Control. Chlamydia control in Europe: literature review. Stockholm: ECDC; 2014b.

Gaydos CA, Howell MR, Pare B, Clark KL, Ellis DA, Hendrix RM, et al. Chlamydia trachomatis Infections in Female Military Recruits. New England Journal of Medicine 1998, 339:739-744.

Gaydos CA, Quinn TC. Chlamydial infections. In Harrison's Principles of Internal Medicine. Volume 2. 18th Edition. Edited by Longo D, Fauci A, Kasper D, Hauser S, Jameson J, Loscalzo J. New York: McGraw Hill; 2012: 1421-1432

Gerbase A, Stein C, Levison J, Htun Y: Global burden of sexually transmitted diseases (excluding HIV) in the year 2000. In: Global burden of sexually transmitted diseases (excluding HIV) in the year 2000 (Ed.). World Health Organization; 2000.

Goldner TE, Lawson HW, Xia Z, Atrash HK. Surveillance for ectopic pregnancy—United States, 1970–1989. MMWR CDC Surveill Summ. 1993;42:73–85.

Goharkhay N, Verma U, Maggiorotto F: Comparison of CT- or ultrasound-guided drainage with concomitant intravenous antibiotics vs. intravenous antibiotics alone in the management of tubo-ovarian abscesses. Ultrasound Obstet Gynecol 2007, 29:65-69.

Haggerty CL, Gottlieb SL, Taylor BD, Low N, Xu F, Ness RB. Risk of sequelae after Chlamydia trachomatis genital infection in women. J Infect Dis 2010, 201 Suppl 2:S134-155.

Haagsma JA, Maertens de Noordhout C, Polinder S, Vos T, Havelaar AH, Cassini A, Devleesschauwer B, Kretzschmar ME, Speybroeck N, Salomon JA. Assessing disability weights based on the responses of 30,660 people from four European countries. Population Health Metrics 2015; 13: 10

Hammerschlag MR, Chandler JW, Alexander ER, English M, Koutsky L. Longitudinal studies on chlamydial infections in the first year of life. The Pediatric Infectious Disease Journal 1982, 1:395-401.

Hammerschlag MR. Chlamydial infections. J Pediatr 1989, 114:727-734.

Hannu TL, Mattila H, Rautelin P, Pelkonen P, Lahdenne A, Siitonen M et al. (2002) Campylobacter-triggered reactive arthritis: a population- based study. Rheumatology 41(3): 312-318.

Kalwij S, Macintosh M, Baraitser P. Screening and treatment of Chlamydia trachomatis infections. BMJ 2010, 340.

Keat A, Thomas BJ, Taylor-Robinson D. Chlamydial infection in the aetiology of arthritis. Br Med Bull 1983, 39:168-174.

Kottmann LM. Pelvic inflammatory disease: clinical overview. J Obstet Gynecol Neonatal Nurs 1995, 24:759-767.

Kwong JC, Ratnasingham S, Campitelli MA, Daneman N, Deeks SL, et al. (2012) The Impact of Infection on Population Health: Results of the Ontario Burden of Infectious Diseases Study. PLoS ONE 7(9): e44103. doi:10.1371/journal.pone.0044103

Lan J, van den Brule AJ, Hemrika DJ, Risse EK, Walboomers JM, Schipper ME, et al. Chlamydia trachomatis and ectopic pregnancy: retrospective analysis of salpingectomy specimens, endometrial biopsies, and cervical smears. J Clin Pathol 1995, 48:815-819.

Land JA, Van Bergen JE, Morre SA, Postma MJ. Epidemiology of Chlamydia trachomatis infection in women and the cost-effectiveness of screening. Hum Reprod Update 2010, 16:189-204.

Low N, McCarthy A, Macleod J, Salisbury C, Campbell R, Roberts TE, et al. Epidemiological, social, diagnostic and economic evaluation of population screening for genital chlamydial infection. Health Technol Assess 2007, 11:iii-iv, ix-xii, 1-165.

Martin-Iguacel R, Llibre JM, Nielsen H, Heras E, Matas L, Lugo R, et al. Lymphogranuloma venereum proctocolitis: a silent endemic disease in men who have sex with men in industrialised countries. Eur J Clin Microbiol Infect Dis 2010, 29:917-925.

McKay L, Clery H, Carrick-Anderson K, Hollis S, Scott G. Genital Chlamydia trachomatis infection in a subgroup of young men in the UK. Lancet 2003, 361:1792.

Miehle W. Medikamentöse Therapie rheumatischer Erkrankungen. Stuttgart: Georg Thieme Verlag; 2003.

Murray CJL, Lopez AD. The Global Burden of Disease - A comprehensive assessment of mortality and disability from diseases, injuries and risk factors in 1990 and projected to 2020. Cambridge: Harvard School of Public Health on behalf of the World Health Organization and the World Bank; 1996.

Ness RB, Soper DE, Holley RL, Peipert J, Randall H, Sweet RL, Sondheimer SJ, Hendrix SL, Amortegui A, Trucco G, Songer T, Lave JR, Hillier SL, Bass DC, Kelsey SF (2002) Effectiveness of inpatient and outpatient treatment strategies for women with pelvic inflammatory disease: results from the Pelvic Inflammatory Disease Evaluation and Clinical Health (PEACH) Randomized Trial. Am J Obstet Gynecol 186: 929-937.

Özgül A, et al. (2006). Clinical presentations of chlamydial and non-chlamydial reactive arthritis. Rheumatology International 26(10): 879-885.

Paik CK, Waetjen LE, Xing G, et al. Hospitalizations for pelvic inflammatory disease and tuboovarian abscess. Obstet Gynecol 2006; 107:611.

Pedowitz P, Bloomfield Rd. Ruptured adnexal abcess (tuboovarian) with generalized peritonitis. Am J Obstet Gynecol 1964; 88:721.

Peipert JF. Clinical practice. Genital chlamydial infections. N Engl J Med 2003, 349:2424-2430.

Quinn TC, Gaydos C, Shepherd M, Bobo L, Hook EW, Viscidi R, et al. Epidemiological and microbiological correlates of Chlamydia trachomatis infection in sexual partnerships. JAMA: Journal of the American Medical Association 1996, 276:1737-1742.

Risser WL, Risser JM. The incidence of pelvic inflammatory disease in untreated women infected with Chlamydia trachomatis: a structured review. Int J STD AIDS 2007, 18:727-731.

Rogstad K. Complications in the female and their management. In: International handbook of Chlamydia. 3rd Edition. Edited by Moss T. Haslemere, UK: Alden Press; 2008: 111-121

Salomon JA, Haagsma JA, Davis A, Maertens de Noordhout C, Polinder S, Havelaar AH, et al. Disability weights for the Global Burden of Disease 2013 study. The Lancet Global Health. [Accepted, in press.]

Sharma D, Dahiya K, Duhan N, Bansal R. Diagnostic laparoscopy in chronic pelvic pain. Arch Gynecol Obstet 2011, 283:295-297.

Soper DE. Pelvic inflammatory disease. Obstetrics and Gynecology, vol. 116, no. 2, part 1, pp. 419–428, 2010.

Stamm WE, Jones RB, Batteiger BE. Chlamydia trachomatis (Trachoma, Prinatal Infections, Lymphogranuloma Venerum and other genital Infections). In: Principles and practice of infectious diseases. Volume 2, 6th Edition. Edited by Mandel GL, Bennet JE, Dolin R. Pennsylvania: Elsevier Inc.; 2005: 2239-2255

Stamm WE. Chlamydia trachomatis infections of the adult. In: Sexually transmitted diseases. 3rd Edition. Edited by Holmes KK, Sparling PF, Mardh PA. New York: McGraw Hill; 1999:407–422

Trojian TH, Lishnak TS, Heiman D. Epididymitis and orchitis: an overview. Am Fam Physician 2009, 79:583-587.

Teisala K, Heinonen PK, Punnonen R. Transvaginal ultrasound in the diagnosis and treatment of tubo-ovarian abscess. Br J Obstet Gynaecol 1990, 97:178-180.

van de Laar MJ, Morre SA. Chlamydia: a major challenge for public health. Euro Surveill 2007, 12:E1-2.

van Valkengoed IG, Morre SA, van den Brule AJ, Meijer CJ, Bouter LM, Boeke AJ. Overestimation of complication rates in evaluations of Chlamydia trachomatis screening programmes--implications for cost-effectiveness analyses. Int J Epidemiol 2004, 33:416-425.

Welte R, Jager H, Postma MJ. Cost-effectiveness of screening for genital Chlamydia trachomatis. Expert Rev Pharmacoecon Outcomes Res 2001, 1:145-156.

Westrom L. Incidence, prevalence, and trends of acute pelvic inflammatory disease and its consequences in industrialized countries. Am J Obstet Gynecol 1980, 138:880-892.

Zar HJ. Neonatal chlamydial infections: prevention and treatment. Pediatric Drugs, vol. 7, no. 2, pp. 103–110, 2005

Cryptosporidiosis

Acute gastroenteritis associated with cryptosporidiosis in humans is in most cases self-limiting and symptoms disappear within a few days or weeks, but in very small number of cases the disease can be fatal.

We assumed that only a small proportion of cases (0.150%) experience the disease as more severe and complicated (Vijgen, 2007).

The average duration of the uncomplicated, mild disease is 3.5 days and 7–18.4 days for the complicated form (Vijgen, 2007).

The case fatality proportion was found to be 0.0042% (Vijgen, 2007), in line with 0.005% found in other studies (Mead, 1999). Mortality from acute gastroenteritis was assumed to be age-dependent and was redistributed according to the age-group-distributed cryptosporidiosis and giardiasis case fatality proportion reported to TESSy between 2009 and 2013 (see Table 3). This table is based on all TESSy notified cases from EEA Member States except Bulgaria, Poland (reporting only aggregate data), Austria, Czech Republic, Iceland, Luxembourg, Malta, Norway, Romania, Slovenia and Slovakia (because the very low incidence reported seems to indicate low sensitivity of the surveillance system).

Cryptosporidiosis can become chronic in immunocompromised persons, especially those with AIDS (Caccio and Pozio, 2006; Call, 2000; Pozio, 1997). However, several studies showed that AIDS-related cryptosporidiosis can be cured following successful antiretroviral therapy (Miao, 2000; Maggi, 2000; Foudraine, 1998).

Model input summary

Table 1. Transition probabilities and distributions used in the outcome tree

Health outcome (Health state)	Distribution of health states in health outcome	Transition probability	Source/assumption
Symptomatic infection			Vijgen, 2007
Uncomplicated)	99.85%		
Complicated)	0.15%		
Fatal cases following symptomatic infection		0.0042%<br>Age dependent (Table 3)	Vijgen, 2007;<br>TESSy 2009–2013

Table 2. Disability weights and duration

Health outcome (Health state)	Disability Weight (DW) (Haagsma, 2015)			Duration	

	DW	Label	In years		Source
Symptomatic infection					Vijgen, 2007
(Moderate)	0.073 (0.061–0.092)	Diarrhoea, mild	0.01		
(Severe)	0.239 (0.202–0.285)	Diarrhoea, severe	0.019–0.05		

Table 3. Age-group redistribution of case fatality proportion due to cryptosporidiosis (0.0042%)

Age groups	%
0	12.50
1–4	6.25
5–9	6.25
10–14	0.00
15–19	0.00
20–24	0.00
25–29	0.00
30–34	6.25

35–39	0.00
40–44	0.00
45–49	6.25
50–54	12.50
55–59	6.25
60–64	6.25
65–69	6.25
70–74	6.25
75–79	18.75
80–84	6.25
>85	0.00
All ages	100.00

References

Caccio SM, Pozio E (2006). Advances in the epidemiology, diagnosis and treatment of cryptosporidiosis. Expert Rev Anti Infect Ther. 4(3):429- 443.

Call SA, Heudebert G, Saag M, Wilcox CM (2000). The changing etiology of chronic diarrhea in HIV-infected patients with CD4 cell counts less than 200 cells/mm3. Am J Gastroenterol , 95(11):3142-3146.

Foudraine NA, Weverling GJ, van Gool T, Roos MT, de Wolf F, Koopmans PP, et al. (1998). Improvement of chronic diarrhoea in patients with advanced HIV-1 infection during potent antiretroviral therapy. Aids, 12(1):35-41.

Haagsma JA, Maertens de Noordhout C, Polinder S, Vos T, Havelaar AH, Cassini A, Devleesschauwer B, Kretzschmar ME, Speybroeck N, Salomon JA. Assessing disability weights based on the responses of 30,660 people from four European countries. Population Health Metrics 2015; 13: 10

Maggi P, Larocca AM, Quarto M, Serio G, Brandonisio O, Angarano G, et al. (2000) Effect of antiretroviral therapy on cryptosporidiosis and microsporidiosis in patients infected with human immunodeficiency virus type 1. Eur J Clin Microbiol Infect Dis, 19(3):213-217.

Mead PS, Slutsker L, Dietz V, McCaig LF, Bresee JS, Shapiro C, et al. (1999) Food-related illness and death in the United States. Emerg Infect Dis, 5(5):607-625.

Miao YM, Awad-El-Kariem FM, Franzen C, Ellis DS, Muller A, Counihan HM, et al. (2000) Eradication of cryptosporidia and microsporidia following successful antiretroviral therapy. J Acquir Immune Defic Syndr, 25(2):124-129.

Pozio E, Rezza G, Boschini A, Pezzotti P, Tamburrini A, Rossi P, et al. (1997) Clinical cryptosporidiosis and human immunodeficiency virus (HIV)- induced immunosuppression: findings from a longitudinal study of HIV-positive and HIV-negative former injection drug users. J Infect Dis, 176(4):969-975.

Vijgen SMC, Mangen MJM, Kortbeek LM, Van Duynhoven YTHP, Havelaar AH (2007). Disease burden and related costs of cryptosporidiosis and giardiasis in the Netherlands. 58 pp. Bilthoven.

Diphtheria

Thanks to vaccination, respiratory diphtheria has almost disappeared from many European countries. In total, 85% of patients suffer from subclinical disease or turn into asymptomatic carriers (Vitek, 1998) and only an estimated 15% of infections lead to a symptomatic case. The duration of acute illness was based on the Ontario Burden of Infectious Disease Study [AC1] (‘the Ontario Study’) [SW2] and set at 12 days (Kwong, 2012).

Risk of complications

Systemic toxicity (a toxic form of the disease with swelling of the neck) occurs in 8.1% of all diphtheria patients and may lead to complications such as myocarditis, neuropathies and renal failure (Rakhmanova, 1996). The more frequent complications of acute illness are myocarditis and polyneuropathies/nerve palsies. Other complications, such as sepsis, septic arthritis, pneumonia, otitis media, splenic and hepatic abscesses and rhinitis, were not included in the outcome tree because they are either extremely rare or mild.

Our model is based on the assumption that 8.1% of symptomatic patients would have a complicated form of the disease (Rakhmanova, 1996).

Permanent disability following myocarditis (arrhythmias)

Assuming that myocarditis represents 66.6% of the complicated diphtheria cases (Jayashree, 2006) and that 0.25% (Mandell, 1999) of these will develop permanent conduction defects (arrhythmias), the transition probability of patients with complications developing permanent cardiac disability is 0.17%.

Case fatality ratio

The US Centers for Disease Control and Prevention (US CDC) have reported a case-fatality proportion (CFP) of 5–10% for diphtheria, with higher death rates (up to 20%) among persons under five and over 40 years. The case fatality proportion has changed very little over the last 50 years (CDC, 2009).

In the model, the CFP associated with uncomplicated disease is 1% and with complicated disease 25.7% (Rakhmanova, 1996).

Model input summary

Table 1. Transition probabilities used in the outcome tree

Health outcome (Health state)	Distribution of health states in health outcome	Transition probability	Source/assumption
Symptomatic infection<br>(Uncomplicated)<br>(Complicated)	 91.9%<br>8.1%		Rakhmanova, 1996
Permanent disability (arrhythmias) following complicated symptomatic infection		0.17%	Jayashree, 2006; Mandell, 1999

Fatal cases following uncomplicated symptomatic infection		1%	Rakhmanova, 1996
Fatal cases following complicated symptomatic infection		25.7%	Rakhmanova, 1996

Table 2. Disability weights and duration

Health outcome (Health state)	Disability Weight (DW) (Haagsma, 2015)		Duration	
	DW	Label	In years	Source
Symptomatic infection (Uncomplicated)	0.051 (0.039-0.06)	Infectious disease, acute episode, moderate	0.003	Kwong 2012
(Complicated)	0.125 (0.104-0.152)	Infectious disease, acute episode, severe		
Permanent disability (arrhythmias) following complicated symptomatic infection	0.295 (0.258-0.343)	Cardiac conduction disorders and cardiac dysrhythmias	Remaining life expectancy	

References

Centers for Disease Control and Prevention. Epidemiology and Prevention of Vaccine-Preventable Diseases. Atkinson W, Wolfe S, Hamborsky J, McIntyre L, eds. 11th ed. Washington DC: Public Health Foundation, 2009.

Demirci CS, Abuhammour W. Diphtheria. Available from: http://emedicine.medscape.com/article/963334-clinical#showall Accessed: 1 September 2011.

Hadfield TL, McEvoy P, Polotsky Y, Tzinserling VA, Yakovlev AA. The pathology of diphtheria. J Infect Dis. 2000;181(SUPPL. 1):S116-20

Haagsma JA, Maertens de Noordhout C, Polinder S, Vos T, Havelaar AH, Cassini A, Devleesschauwer B, Kretzschmar ME, Speybroeck N, Salomon JA. Assessing disability weights based on the responses of 30,660 people from four European countries. Population Health Metrics 2015; 13: 10

Jayashree M, Shruthi N, Singhi S. Predictors of outcome in patients with diphtheria receiving intensive care. Indian Pediatr. 2006;43(2):155-60.

Kwong JC, Ratnasingham S, Campitelli MA, Daneman N, Deeks SL, et al. (2012) The Impact of Infection on Population Health: Results of the Ontario Burden of Infectious Diseases Study. PLoS ONE 7(9): e44103. doi:10.1371/journal.pone.0044103

Lodha R, Dash NR, Kapil A, Kabra SK. Diphtheria in urban slums in north India. Lancet, 2000 Jan 15;355(9199):204. PMID: 10675127

Mandell GL, Bennet JE, Dolin R. Mandell, Douglas and Bennett's principles and practice of infectious disease 5th edition. Churchill Livingstone, 1999.

Merck Manual available at http://www.merckmanuals.com/professional/infectious_diseases/gram-positive_bacilli/diphtheria.html?qt=diphtheria&alt=sh#v1005972 (last update May 2013)

Rakhmanova AG, Nosikova E, Tanasijchuk T, Saikku J. Diphtheria Outbreak in St. Petersburg: Clinical Characteristics of 1,860 Adult Patients. Scand J Infect Dis 1996; 28: 37-40.

Solders G, Nennesmo I, Perssont A, Diphtheritic neuropathy, an analysis based on muscle and nerve biopsy and repeated neurophysiological and autonomic function tests. Journal of Neurology, Neurosurgery, and Psychiatry 1989;52:876-880

Vitek CR, Wharton M. Diphtheria in the former soviet union: Reemergence of a pandemic disease. Emerging Infectious Diseases. 1998;4(4):539-50.

WHO (2004). Global Burden of Disease 2004 Update: Disability weights for diseases and conditions. Geneva: WHO. Available online at: http://www.who.int/healthinfo/global_burden_disease/

GBD2004_DisabilityWeights.pdf

Giardiasis

Acute gastroenteritis associated with giardia in humans is in most cases self-limiting within a few weeks (Wolfe, 2000). Vijgen et al. (Vijgen, 2007) assumed for their disease burden estimates a mean duration of 10 days for gastroenteritis cases not requiring medical help or requiring a visit to the doctor. Severe hospitalised gastroenteritis cases were assumed to last for 30 days.

We assumed that the proportion of more severe cases requiring hospitalisation would be 0.265% (360 cases requiring hospitalisation out of an estimated 136 000 incident cases) (Vijgen, 2007). Moreover, the study presents an age-specific risk of hospitalisation which we applied to the 'severe' health state of the symptomatic infection outcome (see Table 3).

The Dutch Association of Parasitology is not aware of fatal cases of giardia (Vijgen, 2007). Additionally, studies by Adak et al. (Adak, 2002) and Levy et al. (Levy, 1998) have not reported fatal cases.

However, a small number of deaths associated with giardiasis were reported to TESSy: nine cases between 2009 and 2013, resulting in 0.014% of notified cases. The CFP is applied to all symptomatic cases and re-distributed according to the age-group observed deaths for giardiasis and cryptosporidiosis notified between 2009 and 2013 from all Member States, with the exception of Denmark, France, Greece, Italy, Liechtenstein, the Netherlands and Portugal, because they do not report (see Table 4). Data from Bulgaria and Poland were also excluded because they only report aggregate data. It is important to note that the CFP will increase in case multipliers adjusting for under-estimation are applied to the incidence inputted in the toolkit and this should be taken into account.

Risk of complications

Apart from Irritable Bowel Syndrome (IBS) as a possible sequela of giardia, no other sequelae could be identified. However, given the fact that few studies expressed a statistical link between IBS and giardia (1–2%) (Nygard, 2006; Hanevik, 2009; Haagsma, 2010), IBS was not included as a possible complication.

Model input summary

Table 1. Transition probabilities used in the outcome tree

Health outcome<br><br>(Health state)	Distribution of health states in health outcome	Transition probability	Source/assumption
Symptomatic infection<br>(Uncomplicated)<br>(Complicated)	 <br><br>99.735%<br>0.265%<br>Age dep. (Table 3)		
Fatal cases following		0.014%	TESSy 2009-2013

symptomatic infection		Age dependent (Table 4)	

Table 2. Disability weights and duration

Health outcome (Health state)	Disability Weight (DW) (Haagsma, 2015)		Duration	
	DW	Label	In years	Source/assumption
Symptomatic infection				Vijgen, 2007
(Moderate)	0.149 (0.12–0.182)	Diarrhoea, moderate	0.027	
(Severe)	0.239 (0.202-0.285)	Diarrhoea, severe	0.082	

Table 3. Age distribution of severe cases

Age class	%
0–4	27
5–9	27
10–14	3
15–64	34
≥65	8

Table 4. Age-group redistribution of CFR (applied only to complicated cases)

Age groups	%
0	12.50
1–4	6.25
5–9	6.25
10–14	0.00
15–19	0.00
20–24	0.00
25–29	0.00
30–34	6.25
35–39	0.00
40–44	0.00
45–49	6.25
50–54	12.50
55–59	6.25
60–64	6.25
65–69	6.25
70–74	6.25
75–79	18.75
80–84	6.25
>85	0.00
All ages	100.00

References

Adak GK, Long SM, O'Brien SJ. Trends in indigenous foodborne disease and deaths, England and Wales: 1992 to 2000. Gut, 2002. 51(6):832-841.

Food Standards Agency. A Report of the Study of Infectious Intestinal Disease in England. Food Standards Agency London, 2000. HMSO.

Haagsma JA, Siersema PD, De Wit NJ, Havelaar AH. Disease burden of post-infectious irritable bowel syndrome in the Netherlands. Epidemiol Infect 2010. 138(11):1650-6.

Haagsma JA, Maertens de Noordhout C, Polinder S, Vos T, Havelaar AH, Cassini A, Devleesschauwer B, Kretzschmar ME, Speybroeck N, Salomon JA. Assessing disability weights based on the responses of 30,660 people from four European countries. Population Health Metrics 2015; 13: 10

Hanevik K, Dizdar V, Langeland N, Hausken T. Development of functional gastrointestinal disorders after Giardia lamblia infection. BMC Gastroenterol, 2009. 9:27.

Lengerich EJ, Addiss DG, Juranek DD. Severe giardiasis in the United States. Clin Infect Dis, 1994.18(5):760-763.

Levy DA, Bens MS, Craun GF, Calderon RL, Herwaldt BL. Surveillance for waterborne-disease outbreaks – United States, 1995–96. MMWR CDC Surveill Summ, 1998. 47(5):1-34.

Nygard K, Schimmer B, Sobstad O, Walde A, Tveit I, Langeland N. A large community outbreak of waterborne giardiasis-delayed detection in a non-endemic urban area. BMC Public Health, 2006. 6:141.

Vijgen SMC, Mangen MJM, Kortbeek LM, Van Duynhoven YTHP, Havelaar AH. Disease burden and related costs of cryptosporidiosis and giardiasis in the Netherlands. 58 pp. Bilthoven. 2007. National Institute of Public Health and the Environment.

Wolfe MS. Giardiasis. Clin Microbiol Rev, 1992. 5(1):93-100.

Gonorrhoea

Gonorrhoea is the second most commonly reported sexually transmitted disease (STD) in the United States of America (Skolnik & Neil, 2008). Neisseria gonorrhoeae is almost exclusively transmitted by sexual contact and perinatally (from mother to child during labour) (Handsfield & Sparling, 2005). The bacteria affect the mucous membranes of the urethra and the cervix. Less frequently, mucous membranes of the rectum, oropharynx and conjunctivae are also involved during infection. N. gonorrhoeae primarily infects columnar and cuboidal epithelium. Gonorrhoeal infections in women may lead to pelvic inflammatory disease (PID) and may be a cause of female infertility. Further complications resulting from infection with N. gonorrhoeae are epididymitis, ophthalmitis, ectopic pregnancy and disseminated gonococcal infection (DGI). Untreated infections mostly resolve spontaneously over time (several weeks or months) but can lead to serious sequelae associated with adverse effects on health. Even though the duration of disease is hard to estimate, mean duration is assumed to be several days for men and less than two weeks for women. The incubation period is short and re-infection is common (Handsfield & Sparling, 2005).

The true number of gonorrhoea cases is largely affected by under-estimation due to high percentages of asymptomatic cases and diagnosed cases not being reported to the surveillance system. It was estimated that the true number of new infections is twice as high as the reported number (CDC, 2002). Brunham and Embree reported that gonorrhoea is posing serious threats in Africa, Latin America, Asia and eastern Europe (Brunham & Embree, 1992). In 2008, WHO estimated that there were around 46.8 million cases of STDs in the European Region, with 3.4 million cases being due to N. gonorrhoeae (WHO, 2012).

About 40–80% of women are asymptomatically infected (De Maio & Zenilman, 1998; Nelson, 2007). For men symptomatic rates of up to 95- 99% were observed for genital infection (De Maio & Zenilman, 1998; Nelson, 2007; Stamm, 2005).

Health outcomes and health states associated with gonococcal infection

Infection with N. gonorrhoeae results in different clinical pictures in women, men and infants. In our study, we only considered disease models which reflect genital infection; pharyngeal and rectal infections are not considered to be the cause of significant short or long-term sequelae and therefore do not contribute to the burden of gonorrhoea.

Infections in men

An uncomplicated infection presents as an acute urethritis, infection in the pharynx or rectum are likely to be asymptomatic. In 2013, 36% of reported gonorrhoea cases were detected at these sites. In most cases (95–99%) the disease has a symptomatic course with typical signs of dysuria and urethral discharge (De Maio & Zenilman, 1998; Nelson, 2007; Stamm, 2005). In a few cases the infection remains asymptomatic and is neither recognised nor diagnosed (Sherrard, 1996). These infections pose a serious problem as they provide a pool of further transmissible infections. In most cases gonococcal urethritis resolves spontaneously over several weeks but may also trigger sequelae (Handsfield & Sparling, 2005).

The most common sequela of gonococcal infections in men is the acute epididymitis (Stamm, 2005; Trojian, 2009). The symptoms associated with epididymitis are oligospermia during the acute phase, swollen epididymis (and/or testicles), and dysuria. The association between epididymitis and future infertility is an ongoing debate in research with no clear evidence (Stamm, 2005). Uncommon complications are penile oedema, penile lymphangitis, periurethral abscess, acute prostatitis, seminal vasculitis and Tyson's or Cowper's gland infections (Handsfield & Sparling, 2005). Due to their rare occurrence they are not considered in the outcome tree.

Infections in women

Uncomplicated infections in women mostly affect the endocervix and N. gonorrhoeae are also recovered from the urethra, rectum or occasionally from the periurethral (Skene's) glands and the ducts of Bartholin's glands. Many women with gonococcal infections only develop minor symptoms or are entirely asymptomatic and thus do not seek medical advice and are consequently not reported to the surveillance system.

A major complication resulting in remarkable disease burden is pelvic inflammatory disease (PID) (Handsfield & Sparling, 2005; De Maio & Zenilman, 1998). Studies report 10–40% of infected women developing PID (Handsfield, 1974; McCormack, 1977; Westrom, 1980; Westrom, 1992). In a cost effectiveness analysis, Bernstein and colleagues estimated a base case scenario of 30% (range 10–40%) of infected women developing PID (Bernstein, 2006). Women with

PID have an increased risk of developing infertility in the future (Handsfield & Sparling, 2005; De Maio & Zenilman, 1998; Westrom, 1980; Westrom, 1992; Ross, 2002). The study of Weström (1992) and colleagues reported a 10% probability of infected women developing tubal infertility. The risk of infertility is linked to number and severity of PID episodes. Ross reported 15–20% and 50–80% of infected women developing tubal infertility after one and three or more PID episodes, respectively. PID itself is also a cause of further (long-term) sequelae such as chronic pelvic pain, ectopic pregnancy and perihepatitis. Pelvic pain occurs in 20% of cases and ectopic pregnancy in 9.1% of PID cases (Handsfield & Sparling, 2005; Westrom, 1980). Infections with N. gonorrhoeae during pregnancy can result in spontaneous abortion, premature labour, early rupture of fetal membranes and perinatal infant mortality (Handsfield & Sparling, 2005). The cost effectiveness study by Bernstein and colleagues estimated transition probabilities from PID to chronic pelvic pain, ectopic pregnancy and tubal factor infertility of 18% (range 15–30), 7.8% (range 7.8–9.1%), and 15% (range 9–18%), respectively (Bernstein, 2006).

Sequelae reported for both sexes

As a result of bacteraemic dissemination, disseminated gonococcal infection (DGI) can occur in 0.5–3% of people infected with N. gonorrhoeae. This may cause infective arthritis and also be the cause of endocarditis and meningitis in very rare cases (Holmes, 2007).

Gonococcal infections in infants

Infants born to infected mothers can suffer from gonococcal conjunctivitis (ophthalmia neonatorum). Gonococcal conjunctivitis affects 30–35% of children born to infected mothers and is a major problem in many developing countries causing blindness (De Maio & Zenilman, 1998; Nelson, 2007). Ophthalmia neonatorum can lead to corneal scars, resulting in low-vision or complete blindness. Effective treatment is available which has led to very low numbers of sequelae resulting from ophthalmia neonatorum in the developed world (Darling, 2010; Schaller & Klauss, 2001). Consequently, we did not consider corneal-scar-related ‘low-vision’ or ‘blindness’ in our model.

Infected infants may have a low birth weight; some studies relate low birth weight to gonococcal infections (15% from Gerbase, 2000), however the attribution of this condition to the infection is extremely difficult in a developed country setting. Therefore, we decided to discard this relationship.

Case fatality proportion

Fatal cases resulting from gonococcal infections are extremely rare and mainly result from endocarditis, meningitis and DGI. Estimating the mortality of PID is complicated due to the lack of standardised case definitions, inconsistent reporting practices and unclear aetiology (percentage of fatal cases attributable to gonococcal PID) (De Meaio & Zenilman, 1998).

Outcome tree parameters

Male outcome-tree

The proportion of infections in men who develop symptoms is set at 95–99% (De Maio & Zenilman, 1998; Trojian, 2009, Nelson, 2007). The probability of developing DGI (which is part of the initial symptomatic phase of the disease) is set at 0.5–3% (Holmes, 2007), whereas the probability of developing epididymitis is set to 3% (1–5%) (Bernstein, 2006). Debate is currently ongoing as to whether asymptomatic cases also develop epididymitis, however, due to lack of a proven association, this was not taken into account.

Female outcome-tree

Information on the proportion of symptomatic (20–60%) and asymptomatic (40–80%) gonococcal infections were taken from reviews, clinical text books and a study conducted by Weström (Handsfield & Sparling, 2005; De Maio & Zenilman, 1998; Nelson, 2007; Westrom, 1992). Information on PID as a major sequela were obtained from reviews, clinical text books and a cost effectiveness analysis which provided an estimate that 30% (10–40%) of women were symptomatically infected (Bernstein, 2006). The probabilities of developing an ectopic pregnancy (7.8-9.1%), chronic pelvic pain (18%, range 15–30%) or tubal infertility (15%, range 9–18%) were taken from Bernstein`s cost-effectiveness study (Bernstein, 2006). Case fatality proportions from ectopic pregnancies were estimated at 0.038% (Goldner, 1993). The probability of developing a tubo-ovarian abscess is set at 0.8% (Ness, 2002). However, diagnosis and treatment have significantly improved it was therefore decided not to include a case fatality event for tubo-ovarian abscess.

Congenital outcome-tree

The burden studies on STDs by Gerbase and colleagues and Nelson et al. report 30–35% of cases developing ophthalmia neonatorum (Nelson, 2007; Gerbase, 2000).

Assuming that in EU/EEA Member States all notified cases will have had symptoms, in our model all cases of symptomatic infant gonococcal infections manifest as ophthalmia neonatorum and will represent the only health state included in the model.

Model input summary

Table 1. Transition probabilities and distributions used in the outcome tree

Health outcome	Distribution of health states in health	Transition probability	Source/assumption

(health state)	outcome		
Men			
Symptomatic infection<br>(Urethritis)<br>- Uncomplicated<br>- Complicated	 <br><br>97–99.5%<br>0.5–3%	 95–99%	 De Maio & Zenilman, 1998; Nelson, 2007; Stamm, 2005<br>Holmes, 2007
Epididymitis from symptomatic		3% (1–5%)	Bernstein, 2006
Women			
Symptomatic infection<br>(Cervicitis)<br>- Uncomplicated<br>- Complicated	 <br><br>97–99.5%<br>0.5–3%	 20–60%	 Handsfield & Sparling, 2005; De Maio & Zenilman, 1998; Nelson, 2007;<br>Westrom, 1992;<br>Holmes, 2007
Pelvic Inflammatory Disease (PID) from symptomatic and asymptomatic		30% (10–40%)	Bernstein, 2006
Ectopic pregnancy		7.8–9.1%<br>Age dep.<br>See Table 4	Bernstein, 2006<br>Female reproductive age 15-49
Tubo-ovarian abscess		0.8%	Ness, 2002
Chronic pelvic pain syndrome		18% (15–30%)	Bernstein, 2006

Tubal infertility		15% (9–18%)<br>Age dep.<br>See Table 4	Bernstein, 2006<br>Female reproductive age 15-49
Fatal cases due to ectopic pregnancy		0.038%	Goldner, 1993
Congenital			
Symptomatic infection<br>(Ophthalmia neonatorum)		100%	

Table 2. Disability weights and duration

Health outcome (health state)	Disability Weight (DW) (Haagsma, 2015)		Duration	
	DW	Label	In years	Source
Men				
Uncomplicated<br>Complicated	0.051 (0.039-0.06)<br>0.125 (0.104-0.152)	Infectious disease, acute episode, moderate<br>Infectious disease, acute episode, severe	0.02<br>0.02	Trojian, 2009<br>Trojian, 2009
Epididymitis	0.176 (0.143-0.208)	Epididymo-orchitis	0.08	Trojian, 2009
Women				
Uncomplicated<br>Complicated	0.051 (0.039-0.06)<br>0.125 (0.104-0.152)	Infectious disease, acute episode, moderate<br>Infectious disease, acute episode, severe	0.03<br>0.03	Murray, 1996<br>Murray, 1996
Pelvic Inflammatory Disease (PID)	0.123 (0.1-0.15)	Abdominopelvic problem, moderate	0.07	De Maio & Zenilman, 1998
Tubo-ovarian abscess	0.31 (0.262-0.355)	Abdominopelvic problem, severe	0.01	Goharkhay, 2007; Teisala, 1990
Chronic pelvic pain	0.123 (0.1-0.15)	Abdominopelvic problem, moderate	2.8	Sharma, 2011
Ectopic pregnancy	0.31 (0.262-0.355)	Abdominopelvic problem, severe	0.08	Murray, 1996
Tubal infertility	0.007 (0.005-0.01)	Infertility, secondary	See Table 3	Female reproductive age 15–49 years<br>See Table 4

Congenital				
Symptomatic infection (Ophthalmia neonatorum)	0.015 (0.011-0.019)	Conjunctivitis without corneal scar	0.038	American Academy of Pediatrics, 2012. Assuming 2 weeks of treatment

Table 3. Duration of tubal infertility

Age	Duration in years
15–19	32
20–24	27
25–29	22
30–34	17
35–39	12

40–44	7
45–49	2

Table 4. Age group risk (only reproductive age)

Age	%
0–14	0
15–49	100
≥50	0

References

Adelman D, Casale T, Corren J. Manual of Allergy and Immunology. Philadelphia, USA: Lippincott, Williams and Wilkins; 2002.

American Academy of Pediatrics. Chlamydia trachomatis. In: Pickering LK, editor. Red book: 2012. Report of the Committee on Infectious Diseases. Elk Grove Village, IL: American Academy of Pediatrics; 2012:276–81.

American Social Health Association. Sexually Transmitted Diseases in America: How Many Cases and at What Cost? In: Sexually Transmitted Disease in America: How Many Cases and at What Cost? (Ed.). City; 1998:1-27.

Barr J, Danielsson D. Septic gonococcal dermatitis. Br Med J 1971, 1:482-485.

Bernstein KT, Mehta SD, Rompalo AM, Erbelding EJ. Cost-effectiveness of screening strategies for gonorrhea among females in private sector care. Obstet Gynecol 2006, 107:813-821.

Brunham R, Embree J. Sexually transmitted diseases: Current and future dimensions of the problem of the Third World. In: Reproductive Tract Infections Global Impact on Priorities for Women's Reproductive Health. Edited by Germanie A, Holmes K, Piot P, Wasserheit N. New York: Plenum Press; 1992: 35-38

Centers for Disease Control and Prevention. Sexually Transmitted Disease Surveillance, 2002. Atlanta: US Department of Health and Human Services; 2003.

Darling EK, McDonald H. A Meta-analysis of the Efficacy of Ocular Prophylactic Agents Used for the Prevention of Gonococcal and Chlamydial Ophthalmia Neonatorum. Journal of Midwifery & Women's Health 2010, 55:319-327.

DeMaio J, Zenilman J. Gonococcal Infections. In: Bacterial Infections of Humans: Epidemiology and Control. Edited by Evans A, Brachman P. New York: Plenum Medical Book Company; 1998: 285-304

Gerbase A, Stein C, Levison J, Htun Y. Global burden of sexually transmitted diseases (excluding HIV) in the year 2000. In: Global burden of sexually transmitted diseases (excluding HIV) in the year 2000 (Ed.) City: World Health Organization; 2000.

Goharkhay N, Verma U, Maggiorotto F. Comparison of CT- or ultrasound-guided drainage with concomitant intravenous antibiotics vs. intravenous antibiotics alone in the management of tubo-ovarian abscesses. Ultrasound Obstet Gynecol 2007, 29:65-69.

Goldner TE, Lawson HW, Xia Z, Atrash HK. Surveillance for ectopic pregnancy—United States, 1970–1989. MMWR CDC Surveill Summ. 1993;42:73–85.

Haagsma JA, Maertens de Noordhout C, Polinder S, Vos T, Havelaar AH, Cassini A, Devleesschauwer B, Kretzschmar ME, Speybroeck N, Salomon JA. Assessing disability weights based on the responses of 30,660 people from four European countries. Population Health Metrics 2015; 13: 10

Handsfield HH, Lipman TO, Harnisch JP, Tronca E, Holmes KK. Asymptomatic gonorrhea in men. Diagnosis, natural course, prevalence and significance. N Engl J Med 1974, 290:117-123.

Handsfield HH, Sparling RF. Neisseria gonorrhoeae. In Principles and Practice of Infectious Diseases. Volume Volume 2; 6th Edition. Edited by Mandel GL, Bennet JE, Dolin R. Peensylvania: Elsevier Inc.; 2005: 2514-2529

Holmes KK et al. Sexually Transmitted Diseases. 4th Edition 2007. McGraw-Hill Companies: New York. ISBN: 978-0071417488

Marra F, Marra CA, Patrick DM. Cost effectiveness analysis of azithromycin and doxycycline for Chlamydia trachomatis infection in women: A Canadian perspective. Can J Infect Dis 1997, 8:202-208.

McCormack WM, Stumacher RJ, Johnson K, Donner A. Clinical spectrum of gonococcal infection in women. Lancet 1977, 1:1182-1185.

Murray CJL, Lopez AD, World Health Organization, World Bank, Harvard School of Public Health. Global health statistics: a compendium of incidence, prevalence and mortality estimates for over 200 conditions. Geneva: World Health Organization; 1996.

Murray CJL, Lopez AD. The Global Burden of Disease - A comprehensive assessment of mortality and disability from diseases, injuries, and risk factors in 1990 and projected to 2020. Cambridge: Harvard School of Public Health on behalf of the World Health Organization and the World Bank; 1996.

Nelson AL. Gonorrheal Infections. In: Sexually Transmitted Infections - Current Clinical Practice. Edited by Nelson AL, Woodward J, Wysocki S: Humana Press; 2007: 153-182

Ness RB, Soper DE, Holley RL, Peipert J, Randall H, Sweet RL, et al. Effectiveness of inpatient and outpatient treatment strategies for women with pelvic inflammatory disease: results from the Pelvic Inflammatory Disease Evaluation and Clinical Health (PEACH) Randomized Trial. Am J Obstet Gynecol 2002. 186: 929-937.

Newman LM, Moran JS, Workowski KA. Update on the management of gonorrhea in adults in the United States. Clin Infect Dis 2007, 44 Suppl 3:S84-101.

Ross JD. An update on pelvic inflammatory disease. Sex Transm Infect 2002, 78:18-19.

Rowley J, Berkely S (1998). Sexually Transmitted Diseases. In: Health Dimensions of Sex and Reproduction. The Global Burden of Disease and Injuries Series, Volume 3. Harvard University Press, Cambridge.

Schaller UC, Klauss V. Is Crede's prophylaxis for ophthalmia neonatorum still valid? Bull World Health Organ 2001, 79:262-263.

Sharma D, Dahiya K, Duhan N, Bansal R. Diagnostic laparoscopy in chronic pelvic pain. Arch Gynecol Obstet 2011, 283:295-297.

Sharp JT. Editorial: Gonococcal infections and arthritis. Arthritis Rheum 1974, 17:511-512.

Sherrard J, Barlow D. Gonorrhoea in men: clinical and diagnostic aspects. Genitourin Med. 1996;72:422–6.

Skolnik NS, Neil S. Sexually Transmitted Diseases. In: Essential Infectious Disease Topics in Primary Care - Current Clinical Practice. Edited by Albert RH: Humana Press; 2008: 105-166

Spencer SE, Bash MC. Extragenital manifestations of Neisseria gonorrhoeae. Curr Infect Dis Rep 2006, 8:132-138.

Stamm WE, Jones RB, Batteiger BE. Chlamydia trachomatis (Trachoma, prinatal infections, Lymphogranuloma venerum and other genital infections). In Principles and Practice of Infectious Diseases. Volume 2, 6th Edition. Edited by Mandel GL, Bennet JE, Dolin R. Pennsylvania: Elsevier Inc.; 2005: 2239-2255

Teisala K, Heinonen PK, Punnonen R. Transvaginal ultrasound in the diagnosis and treatment of tubo-ovarian abscess. Br J Obstet Gynaecol 1990, 97:178-180.

Trojian TH, Lishnak TS, Heiman D. Epididymitis and orchitis: an overview. Am Fam Physician 2009, 79:583-587.

Westrom L, Joesoef R, Reynolds G, Hagdu A, Thompson SE. Pelvic inflammatory disease and fertility. A cohort study of 1,844 women with laparoscopically verified disease and 657 control women with normal laparoscopic results. Sex Transm Dis 1992, 19:185-192.

Westrom L. Incidence, prevalence, and trends of acute pelvic inflammatory disease and its consequences in industrialized countries. Am J Obstet Gynecol 1980, 138:880-892.

Wise CM, Morris CR, Wasilauskas BL, Salzer WL. Gonococcal arthritis in an era of increasing penicillin resistance. Presentations and outcomes in 41 recent cases (1985-1991). Arch Intern Med 1994, 154:2690-2695.

World Health Organization. Global incidence and prevalence of selected curable sexually transmitted infections - 2008. Geneva: World Health Organization; 2012.

Hepatitis A

Hepatitis A virus (HAV) infections range from asymptomatic health state to fulminant hepatitis (Jeong & Lee, 2010). Hepatitis A symptomatic infections depend strongly on the age: approximately 30% of infected children develop symptoms (Jeong & Lee, 2010; Ciocca, 2000), whereas, according to literature, this is 70–80% for adults (Jeong & Lee, 2010; Ciocca, 2000; Cuthbert, 2001). The manifestation of HAV infection in young children generally includes mild flu-like, but anicteric symptoms (Gingrich, 1983), whereas in adults frequently reported symptoms are jaundice, dark urine, fatigue, loss of appetite, abdominal pain and light-coloured stool lasting for several weeks (Koff, 1992).

Not only severity, also duration is related to the age of the patient. Symptoms in young children last for one to two weeks (Gingrich, 1983). According to Koff, around 80% of adults are ill for up to eight weeks (Koff, 1992). Haagsma et al. assumed that symptomatic HAV cases not requiring medical help would have symptoms for 14 days, and symptomatic HAV cases requiring any kind of medical help would have symptoms for 30 days (Haagsma, 2009). Havelaar et al. assumed that hospitalised HAV cases would have symptoms for up to 0.3 years(Haavelar, 2012). According to the US Centers for Disease Control and Prevention, clinical illness usually does not last longer than two months, although 10–15% of persons have prolonged or relapsing signs of symptoms for up to six months (CDC, 2012).

The case fatality proportions are reported to be 0.1% (Mead, 1999), 1% of hospitalised HAV cases (Arteaga Rodriguez, 2010) and 0.3% (Bauch, 2007; Fiore, 2004).

Fatal cases occur mainly in elderly people (Bauch, 2007; Jacobs, 2004; Jacobs, 2000). In the following table we have summarised the rates of mortality attributable to HAV as used in various cost-effectiveness analyses (Bauch, 2007; Jacobs, 2004; Jacobs, 2000).

Table 1. Deaths among symptomatic patients per 10 000 stratified for age classes

Age classes (in years)	Sources		
	Bauch 2007	Jacobs 2004	Jacobs 2000
	30	-	
5-14	18	-	
15-19	18	-	18 (6-30)
20-29	18	18	18 (6-30)
30-39	21	21	21 (10-32)
40-49	59	36	36 (23-49)

50-59	59	81	81 (70-92)
60-69	272	149	149 (146-152)
70-79	272	283	283 (154-310)
>80	272	283	385 (356-414)

We chose to consider the overall case fatality proportion to be within the range 0.1–0.3% and assumed a different age-group distribution of this risk based on the age-group distribution of fatal cases reported to TESSy between 2009 and 2013 (see Table 4). This table is based on all TESSy notified cases from EU Member States except Bulgaria, Lithuania, Latvia and Poland, because they report only aggregate data, and Liechtenstein which does not report.

Risk of complications

Fulminant hepatitis is a rare complication of hepatitis (Jeong & Lee, 2010). According to Bauch et al. (Bauch, 2007), the probability of fulminant infection in hospitalised HAV cases is 0.011%. Jacobs et al. (Jacobs, 2004) assumed that the probability of liver transplantation would be 0.02% for symptomatic HAV cases in 25 to 29-year olds, increasing slightly with age to 0.08% for symptomatic HAV cases in 70-year olds. According to Jeong and Lee (Jeong & Lee, 2010), a liver transplantation may be necessary, however HAV-related fulminant hepatitis does resolve spontaneously on a more frequent basis than fulminant hepatitis of other aetiologies. Given the low incidence, and the resulting negligible burden, fulminant hepatitis was not considered as a separate health outcome in the current study.

In a current review (Jeong & Lee, 2010), rare atypical clinical manifestations and extra-hepatic manifestations are listed. Atypical clinical manifestations occasionally reported are: relapsing hepatitis, prolonged cholestasis, and complicated cases with acute kidney injury. Rarely reported extra-hepatic manifestations are autoimmune haemolytic anaemia, aplastic anaemia, pure red cell aplasia, pleural or pericardial effusion, acute reactive arthritis, acute pancreatitis, acalculous cholecystitis, mononeuritis, and Guillain-Barré syndrome. None of these manifestations were considered in a recent disease burden study (Havelaar, 2012), nor in cost-effectiveness studies evaluating HAV vaccination programmes (Bauch, 2007; Jacobs, 2000, 2004).

Model input summary

Table 2. Transition probabilities used in the outcome tree

Health outcome (Health state)	Distribution of health states in health outcome	Transition probability	Source/assumption
Fatal cases		0.1–0.3%. Age-dependent (Table 4)	Mead 1999, Bauch 2007, Fiore 2004

Table 3. Disability weights and duration

Health outcome (Health state)	Disability Weight (DW) (Haagsma, 2015)		Duration	
	DW	Label	In years	Source/assumption
Symptomatic infection	0.125 (0.104–0.152)	Infectious disease, acute episode, severe	0–9 years: 0.019–0.038 $\geq$ 10 years: 0.082 (0.038–0.5). See Table 5.	CDC 2012; Haagsma 2009, age-dependent

Table 4. Age-group redistribution of case fatality proportion (0.1–0.3%)

Age groups	%
0	0.00
1-4	0.00
5-9	0.00
10-14	0.00
15-19	0.00
20-24	10.00
25-29	0.00
30-34	0.00
35-39	0.00

40-44	10.00
45-49	0.00
50-54	10.00
55-59	20.00
60-64	10.00
65-69	0.00
70-74	20.00
75-79	20.00
80-84	0.00
>85	0.00
All ages	100.00

Table 5. Duration of symptomatic disease by age group

Age	%
0-9	0.019–0.038
$\geq$ 10	0.082 (0.038–0.5)

References

Allen CJ, Ferson MJ. Notification of infectious diseases by general practitioners: a quantitative and qualitative study. Med J Aust 2000, 172:325- 328.

Arteaga-Rodriguez A, Carrasco-Garrido P, de Andres AL, de Miguel AG, Santos J, Jimenez-Garcia R. Changes in the epidemiology of hepatitis A in Spain (2005-2008): trends of acute hepatitis A hospitalizations, comorbidities, and costs associated with the hospitalization. Eur J Gastroenterol Hepatol 2010, 22:1284-1289.

Bauch CT, Anonychuk AM, Pham BZ, Gilca V, Duval B, Krahn MD. Cost-utility of universal hepatitis A vaccination in Canada. Vaccine 2007, 25:8536-8548.

Centers for Disease Control and Prevention. Epidemiology and Prevention of Vaccine-Preventable Diseases. Atkinson W, Wolfe S, Hamborsky J, eds. 12th ed. Washington DC: Public Health Foundation, 2012.

Ciocca M. Clinical course and consequences of hepatitis A infection. Vaccine 2000, 18 Suppl 1:S71-74.

Cuthbert JA: Hepatitis A: old and new. Clin Microbiol Rev 2001, 14:38-58.

Fiore AE. Hepatitis A transmitted by food. Clin Infect Dis 2004, 38:705-715.

Gingrich GA, Hadler SC, Elder HA, Ash KO. Serologic investigation of an outbreak of hepatitis A in a rural day-care center. Am J Public Health 1983, 73:1190-1193.

Haagsma JA, Janssen MF, Bonsel GJ. Comparing generic and disease specific health state valuations by a laymen panel. In EuroQol proceedings; Oslo, Norway. 2005

Haagsma JA, van der Zanden BP, Tariq L, van Pelt W, van Duynhoven YTHP, Havelaar AH. Disease burden and costs of selected foodborne pathogens in the Netherlands, 2006. vol. RIVM rapport 330331001. Bilthoven: National Institute of Public Health and Environment; 2009.

Haagsma JA, Maertens de Noordhout C, Polinder S, Vos T, Havelaar AH, Cassini A, Devleesschauwer B, Kretzschmar ME, Speybroeck N, Salomon JA. Assessing disability weights based on the responses of 30,660 people from four European countries. Population Health Metrics 2015; 13: 10

Havelaar AH, Haagsma J, Mangen MJJ, Kemmeren JM, Verhoef L, Vijgen SMC, et al. Disease burden of foodborne pathogens in the Netherlands, 2009. International Journal of Food Microbiology 2012 156:231-238.

Havelaar AH, Melse JM. Quantifying public health risks in the WHO Guidelines for Drinking-Water Quality. Bilthoven, the Netherlands: National Institute for Public Health and the Environment; 2003.

Jacobs RJ, Gibson GA, Meyerhoff AS: Cost-effectiveness of hepatitis A-B vaccine versus hepatitis B vaccine for healthcare and public safety workers in the western United States. Infect Control Hosp Epidemiol 2004, 25:563-569.

Jacobs RJ, Margolis HS, Coleman PJ. The cost-effectiveness of adolescent hepatitis A vaccination in states with the highest disease rates. Arch Pediatr Adolesc Med 2000, 154:763-770.

Jeong SH, Lee HS. Hepatitis A: clinical manifestations and management. Intervirology 2010, 53:15-19.

Koff RS. Clinical manifestations and diagnosis of hepatitis A virus infection. Vaccine 1992, 10 Suppl 1:S15-17.

Matin N, Grant A, Granerod J, Crowcroft N. Hepatitis A surveillance in England--how many cases are not reported and does it really matter? Epidemiol Infect 2006, 134:1299-1302.

Mead PS, Slutsker L, Dietz V, McCaig LF, Bresee JS, Shapiro C, et al. Food-related illness and death in the United States. Emerg Infect Dis 1999, 5:607-625.

Van de Laar MJW, Wijgergangs LM, Rijlaarsdam J, de Wit A. Vaccineren tegen hepatitis A: een verkenning van kosten. Infectieziektebulletin 2000, 11:262-266.

WHO. Global Burden of Disease 2004 Update: Disability weights for diseases and conditions. Geneva: WHO; 2004.

Hepatitis B

Hepatitis B is caused by the hepatitis B virus (HBV) which affects the liver and can cause both acute and chronic infections. Many patients present no symptoms during the initial infection.

The following estimates have been calculated for the proportion of infected individuals who develop symptoms:

- 30–50% of those adults infected develop acute icteric hepatitis (McMahon et al. 1985)

- Over 90 percent of perinatal HBV infections are asymptomatic, while the typical manifestations of acute hepatitis are noted in 5–15 percent of newly-infected young children (1–5 years of age) and in 33–50 percent of older children, adolescents, and adults (Shepard et al. 2006).

We therefore assumed that the range for the symptomatic proportion of new infections was age-dependent (see Table 1). The duration of acute illness has been estimated at six weeks (Kwong, 2012).

Chronicity rate

There is much evidence of age-related variation in the development rate for chronic HBV infection after acute infection. For example:

- The likelihood of developing chronic HBV infection is higher in individuals infected perinatally (90%) or during childhood (20–30%), when the immune system is thought to be immature, compared with immunocompetent subjects infected during adulthood (<1%)' (Fattovich, 2008)

- The overall chronicity rate for HBV has been estimated at 5–10%, although it is higher in those who were infected perinatally (90%) or during childhood (20%) (Yim & Lok, 2005)

- More than 90% of infected infants, 25–50% of children infected between and 5 years, and 6–10% of acutely infected older children and adults develop chronic infection (Shepard et al. 2006)

- About 30% of children aged 1–5 years and 5% of adults develop chronic hepatitis B infection (Pungpadong et al. 2007).

- Nearly all persons infected perinatally and up to 50% of children infected between the ages of 1–5 years develop chronic hepatitis (NIH, 2008)

- 5% of adults with acute infection develop chronic hepatitis B (Wilt et al. 2008)

- 5-10% of adult patients do not clear the virus and either progress to become asymptomatic carriers or develop chronic hepatitis (WHO 2002)

- The chronicity rate is approximately 90% for infants in the first year of life, 30% for children infected between the ages of 1 and 4 years and <5% for healthy adults (Edmunds et al. 1993).

In the model, we adopted the age-dependent chronicity rates reported above by Fattovich et al. presented in the results of a systematic review of the literature (2008).

The duration of the chronic carrier stage varies according to the presence or absence of active viral replication, estimated at 4.5 years in the case of active viral replication and 33.24 years in the case of no active replication (Stouthard, 1997). Information on the proportion of chronic hepatitis cases with active viral replication to those without active replication is not available and we chose to set the duration as uncertain, between 4.5 and 33.24 years.

Risk of complications

Fulminant liver failure

Fulminant liver failure occurs in approximately 0.5 to 1.0% of adults with reported acute hepatitis B but rarely in infants and children (Pappas, 1995; Hoofnagle et al. 1995). In the model we specified a range (0.5–1.0%) for this transition probability for all age groups as we were unable to locate specific values for infants and children. However, we modelled the age-specific probability of the case fatality rate based on the observed rates, hence a zero probability of children dying of acute hepatitis (see Table 5).

The case fatality rate (CFR) among patients who develop fulminant liver failure is approximately 20–33% (Bernua et al. 1986; Wai et al. 2005) and this figure was chosen for our model. There were no recent specific European studies stating the frequency and impact of orthotopic liver transplantation (OLT) (Steinmuller et al. 2002) and new antiviral medications (Eisenbach, 2006).

The duration of fulminant liver failure, estimated based on the time from onset of symptoms to encephalopathy, is one to 56 days (Trey and Davidson 1970).

Compensated cirrhosis (CC)

According to Chu (2000), on average, 2.1% of people with chronic HBV infection develop compensated cirrhosis annually. This does not take into account variations due to other effects such as alcohol consumption, diabetes and obesity (in the BCoDE toolkit the yearly rate refers to an Annual Transition Probability, ATP, as opposed to the Lifetime Transition Probability, LTP). However, it is important to consider that individuals who have a severe acute exacerbation complicated by subacute hepatic failure or who have recurrent episodes of acute exacerbations with bridging hepatic necrosis are more likely to develop cirrhosis (Chu, 2000)

Decompensated cirrhosis (DC)

According to a systematic review undertaken by D'Amico et al. (2006. The review undertaken by Fattovich et al. (2008) estimated an annual probability of 3–4% for Europe which we chose for our model.

The 20–57% case fatality rate for DC was estimated based on the review by D`Amico et al. (20% from the first of two DC stages, characterised by ascites with or without non-bleeding esophageal varices; 57% from the second of two DC stages, characterised by bleeding varices, with or without ascites).

The duration of DC is based on the average waiting time for liver transplants in EU countries which publish their data online (UK and Spain): between 124 and 142 days (NHS, 2014; Matesanz 2009).

Hepatocellular carcinoma (HCC)

The annual rate of developing HCC is 0.1% in asymptomatic HBsAg individuals, and between 0.3 and 1% in patients with chronic hepatitis B, but this rate increases to 2–10% in patients with compensated cirrhosis (Fattovich, 2008; Yim & Lok, 2005; Pungpadong, 2007; Chu, 2000; D'Amico, 2006). Chu and Liaw (2006) and Fattovich (2008) estimated the CC to HCC transition probability to range between 1.5 and 2.2%/year for Europe.

For the model, we adopted Fattovich`s (2008) estimate stemming from an extensive systematic literature review of 0.3% (0.12–0.41) per year to develop HCC from chronic hepatitis B infection and 2.2% (1.71–2.71) per year for the development of HCC from compensated cirrhosis.

In a European setting, Shepherd`s (2006) cost-effectiveness analysis set the annual case fatality rate for HCC to 56%, while Kanwal (2005) set it to 43.3% (20–60). We chose the latter range for our model as it includes Shepherd`s assumption.

Model input summary

Table 1. Transition probabilities and distributions used in the outcome tree

Health outcome<br>(Health state)	Distribution of health states within health outcome	Transition probability	Source/assumption
Symptomatic infection		10–50%<br>See Table 3	Age-dependent<br>McMahon, 1985; Shepard, 2006
Chronic hepatitis		1–90%<br>See Table 4	Age-dependent<br>Fattovich, 2008
Fulminant liver failure		0.5–1%	Pappas, 1995; Hoofnagle et al. 1995
Fatal cases due to liver failure		20-33.3%<br>See Table 5	Bernau et al. 1986 ; Wait et al. 2005<br>Assuming different age-specific probabilities based on observed mortality

Compensated cirrhosis		2.1%/year	Chu, 2000 (ATP)
Decompensated cirrhosis		3-4%/year	Fattovich, 2008 (ATP)
HCC, following<br>- Chronic hepatitis<br>- Compensated cirrhosis		0.3% (0.12–0.41)/year<br>2.2% (1.71–2.71)/year	Fattovich, 2008 (ATP<br>Fattovich, 2008 (ATP
CFR, following:<br>- DC<br>- HCC		20-57%/year<br>43.3% (20-60)/year	D'Amico, 2006 (ATP)<br>Kanwal, 2005 (ATP)

Table 2. Disability weights and duration

Health outcome (Health state)	Disability Weight (DW) (Haagsma, 2015)			Duration
	DW	Source: ECDC European Disability Weight Project (2014)	In years	Source/assumption
Symptomatic infection	0.051 (0.039–0.06)	Infectious disease, acute episode, moderate	0.115	Kwong 2012
Fulminant liver failure	0.515 (0.459–0.572)	Terminal phase, with medication (for cancers, end-stage kidney/liver disease)	0.003–0.153	Trey, 1970
Chronic hepatitis	0.07 (0.057–0.088)	Generic, uncomplicated disease: worry and daily medication	4.5–33.24	Stouthard, 1997

				Assuming uncertainty between proportion with active replication and without
Compensated cirrhosis	0.07 (0.057–0.088)	Generic uncomplicated disease: worry and daily medication	6-10.4<br><br>See Table 6	Murray, 1996<br><br>Age and gender specific
Decompensated cirrhosis	0.163 (0.136–0.194)	Decompensated cirrhosis of the liver	0.34–039	Assuming average waiting time before liver transplantation in the UK and Spain (NHS and Matesanz 2009)
Hepatocellular carcinoma	0.265 (0.222–0.303)	Cancer, diagnosis and primary therapy	0.72–4.48<br><br>See Table 7	Murray, 1996<br><br>Age and gender specific

Table 3. Hepatitis B infected developing symptoms

Age group	Symptomatic hepatitis B
0	10%
1–4	5–15%
5–80+	30–50%

Table 4. Hepatitis B infected developing chronic hepatitis

Age group	Chronic hepatitis B
0	90%
1–4	20–30%
5–80+	1%

Table 5. CFR age distribution for acute hepatitis observed in Estonia, Germany and the Netherlands 2005–2007

Age groups	CFR
0	0.00

1-4	0.00
5-9	0.10
10-14	0.00
15-19	0.00
20-24	0.14
25-29	0.30
30-34	0.53
35-39	1.27
40-44	1.75
45-49	4.56
50-54	5.81
55-59	5.83
60-64	7.90
65-69	11.86
70-74	11.97
75-79	19.77
80-84	15.67
>85	12.54
All ages	100

Table 6. Duration of compensated cirrhosis

	F	M
0–4	10.4	10.3
5–14	10.4	10.4
15–44	10.2	10
45–59	9.3	8.8
60+	6.5	6

Table 7. Duration of HCC

Age group	Duration (years)	
	F	M
0–14	4.48	4.11
15–44	1.45	2.92
45–59	1.91	2.88
60+	0.72	1.56

References

Alter MJ, Mares A, Hadler SC, Maynard JE. The effect of underreporting on the apparent incidence and epidemiology of acute viral hepatitis. Am J Epidemiol. 1987;125(1):133-9.

Bernuau J, Goudeau A, Poynard T. Multivariate analysis of prognostic factors in fulminant hepatitis B. Hepatology, vol.6, no. 4, pp. 648–651, 1986

Bernuau J, Rueff B, Benhamou JP. Fulminant and subfulminant liver failure: definitions and causes. Seminars in Liver Disease, vol. 6, no. 2, pp. 97–106, 1986

Christensen PB, et al. Outbreak of Hepatitis B among injecting drug users in Denmark. J Clinical Virology 2001;22(1):133-141.

Chu CM. (2000), Natural history of chronic hepatitis B virus infection in adults with emphasis on the occurrence of cirrhosis and hepatocellular carcinoma. Journal of Gastroenterology and Hepatology, 15: E25–E30.

D'Amico G, Garcia-Tsao G, Pagliaro L. Natural history and prognostic indicators of survival in cirrhosis: a systematic review of 118 studies. Journal of Hepatology 2006;44(1):217-31.

Edmunds WJ, Medley GF, Nokes DJ, Hall AJ, Whittle HC. The influence of age on the development of the hepatitis B carrier state. Proceedings Biological Sciences, 1993;253(1337):197-201.

Elhouhari HM, Tamimi TIAR, Carey WD. Hepatitis B virus infection: Understanding its epidemiology, course, and diagnosis. Cleveland Clinical Journal of Medicine 2008;75:881-889.

Eisenbach C, Sauer P, Mehrabi A, Stremmel W, Encke J. Prevention of hepatitis B virus recurrence after liver transplantation. Clin Transplant 2006; 20 Suppl 17: 111–116

Fattovich G, Bortolotti F, Donato F. Natural history of chronic hepatitis B: special emphasis on disease progression and prognostic factors. [Review] [143 refs]. Journal of Hepatology. 2008 20071204 [Epub ahead of print];48(2):335-52.

Garfein RS, Vlahov D, Galai N, et al. Viral infections in short-term injection drug users: the prevalence of the hepatitis C, hepatitis B, human immunodeficiency, and human T-lymphotropic viruses. Am J Public Health 1996;86:655–661.

Greten TF, Papendorf F, Bleck JS, Kirchhoff T, Wohlberedt T, Kubicka S, et al. Survival rate in patients with hepatocellular carcinoma: a retrospective analysis of 389 patients. British Journal of Cancer, 2005 May 23;92(10):1862-8.

Haagsma JA, Maertens de Noordhout C, Polinder S, Vos T, Havelaar AH, Cassini A, Devleesschauwer B, Kretzschmar ME, Speybroeck N, Salomon JA. Assessing disability weights based on the responses of 30,660 people from four European countries. Population Health Metrics 2015; 13: 10

Hoofnagle JH, Carithers RL Jr, Shapiro C, Ascher N. Fulminant hepatic failure: summary of a workshop. Hepatology 1995;21:240-252.

Hoofnagle JH, Doo E, Liang TJ, Fleischer R, Lok ASF. Management of Hepatitis B: Summary of a Clinical Research Workshop. Hepatology 2007;45:1056-1075.

Kanwal F, Gralnek IM, Martin P et al. Treatment alternatives for chronic hepatitis B viral infection: a cost-effectiveness analysis. Annals of Internal Medicine. 2005;142:821-31.

Kim WR, Brown RS, Terrault NA, El-Serag H. Burden of liver disease in the United States: summary of a workshop. Hepatology 2002; 36: 227-242

Kwong JC, Ratnasingham S, Campitelli MA, Daneman N, Deeks SL, et al. (2012) The Impact of Infection on Population Health: Results of the Ontario Burden of Infectious Diseases Study. PLoS ONE 7(9): e44103. doi:10.1371/journal.pone.0044103

Levine OS, Vlahov D, Nelson KE. Epidemiology of hepatitis B virus infections among injecting drug users: seroprevalence, risk factors, and viral interactions. Epidemiol Rev 1994;16:418–36

Levine OS, Vlahov D, Koehler J, et al. Seroepidemiology of hepatitis B virus in a population of injecting drug users. Association with drug injection patterns. Am J Epidemiol 1995;142:331–41.

Liaw YF (2009) Natural history of chronic hepatitis B virus infection and long-term outcome under treatment. Liver international Vol. 29; pp 100- 107.

Lok ASF, McMahon (2007) Chronic Hepatitis B. Hepatology Vol. 45; pp 507 - 539

Matesanzemail R, de la Rosa G. Liver transplantation: The Spanish experience. Digestive and Liver Disease Supplements. Volume 3, Issue 4 , Pages 75-81, December 2009.

McMahon BJ, Alward WL, Hall DB, Heyward WL, Bender TR, Francis DP, et al. Acute hepatitis B virus infection: relation of age to the clinical expression of disease and subsequent development of the carrier state. The Journal of Infectious Diseases, 1985 Apr;151(4):599-603.

McMahon BJ (2005). Epidemiology and natural history of Hepatitis B. Seminars in liver disease. Vol. 25. Supplement 1.

Murray CJL, Lopez AD. World Health Organization, World Bank, Harvard School of Public Health. Global health statistics: a compendium of incidence, prevalence and mortality estimates for over 200 conditions. Geneva: World Health Organization; 1996.

Murray CJL, Lopez AD. The Global Burden of Disease - A comprehensive assessment of mortality and disability from diseases, injuries, and risk factors in 1990 and projected to 2020. Cambridge: Harvard School of Public Health on behalf of the World Health Organization and the World Bank; 1996.

National Health System (NHS), UK. h p://www.nhs.uk/condi̭ons/Liver-transplant/Pages/Introduc̭on.aspx. Website visited on 31 July 2014

National Institutes of Health (2008) NIH Consensus Development Conference Statement on Management of Hepatitis B. NIH Consensus and State-of-the-Science Statements Volume 25, Number 2. October 20–22, 2008

Pungpapong S, Kim WR, Poterucha JJ. Natural history of hepatitis B virus infection: an update for clinicians. Mayo Clin Proc 2007; 82:967–975.

Shepard CW, Simard EP, Finelli L, Fiore AE, Bell BP (2006) Hepatitis B virus infection: Epidemiology and vaccination. Epidemiological Reviews; Vol. 28; pp 112-125

Shepherd J, Jones J, Takeda A et al. Adefovir dipivoxil and pegylated interferon alfa-2a for the treatment of chronic hepatitis B: a systematic review and economic evaluation. [Review] [136 refs]. Health Technology Assessment (Winchester, England). 2006;10(28):iii-iiv.

Stouthard M , Essink-Bot M, Bonsel G, Barendregt J, Kramers P. 1997. Disability weights for diseases in the Netherlands. Rotterdam, Department of Public Health, Erasmus University.

Trey C, Davidson CS. The management of fulminant hepatic failure. In: Popper H, Schaffner F, eds. Progress in liver disease. Volume 3. New York: Grune & Stratton, 1970:282-98

Wai CT, Fontana RJ, Polson J, Hussain M, Shakil AO, Han SH, et al. Clinical outcome and virological characteristics of hepatitis B-related acute liver failure in the United States. J Viral Hepat 2005; 12: 192-198

Wilt TJ, Shamliyan T, Shaukat A, Taylor BC, MacDonald R, Yuan J-M, et al. (2008) . Management of Chronic Hepatitis B. Evidence Report/Technology Assessment No. 174. (Prepared by the Minnesota Evidence-based Practice Center under Contract No. 290-02-0009.) AHRQ Publication No. 09-E002. Rockville, MD. Agency for Healthcare Research and Quality. October 2008.

WHO (2002) Hepatitis B; WHO/CDS/CSR/LYO/2002.2:Hepatitis B

WHO (2001) Introduction of Hepatitis B vaccine into childhood immunization services

Yim HJ, Lok ASF. Natural history of chronic hepatitis B virus infection: what we knew in 1981 and what we know in 2005. Hepatology 2006; 43:pp 173–S181.

Hepatitis C

A total of 20–30% of newly infected individuals develop clinical symptoms of acute hepatitis (e.g. anorexia, abdominal discomfort, nausea, vomiting and jaundice) within 2–24 weeks of exposure (CDC, 2011; Wasmuth, 2010; World Health Organization, 2002). In persons who do develop symptoms of acute hepatitis, the illness lasts between two and 12 weeks (Wasmuth, 2010).

In the model, it was assumed that 20–30% of newly infected individuals develop clinical symptoms of acute hepatitis (CDC, 2011).

Rate of developing chronic hepatitis C

Acute hepatitis C develops into chronic infection in 75.6% (67.3–84.9) of all symptomatic and asymptomatic cases over 20 years old, with the infection resolving in the remaining proportion (Alter & Seef, 1994). The chronicity rate is known to be lower in younger individuals. A recent review of the literature by Alter et al. (2000), has estimated that the rate of spontaneous recovery is 29–45% in those aged under 20 years and this was used for the disease model (chronicity rate: 55–69%).

In the early stages of chronic infection there is a small chance of spontaneous remission. The rate of remission of chronic hepatitis C was set at 0.31 (0.26–0.36)% per year in accordance with the findings of Micallef et al. (2006) (in the Burden of Communicable Diseases in Europe toolkit a yearly rate refers to an Annual Transition Probability, ATP, as opposed to the Lifetime Transition Probability, LTP).

In the absence of spontaneous remission or successful antiviral therapy, chronic infections may progress from mild to moderate hepatitis to liver cirrhosis, with a risk of developing life-threatening sequelae such as decompensated liver disease and hepatocellular carcinoma. Progression to severe liver disease can take 20–40 years. However, progression, which is non-linear, is strongly influenced by cofactors including alcohol intake, HIV or HBV coinfection, gender (male) and an older age at infection (Alberti, 2005; Alter & Seeff, 2000; Freeman, 2003; Lauer & Walker, 2001; Poynard, 2001; Thein, 2008).

Given emerging knowledge of the disease, the most appropriate approach to simulating the progression from chronic infection to cirrhosis would be to specify a model with five health stages, representing the METAVIR fibrosis stages F0–F4, linked by multivariate risk functions. A further possibility could be to represent mild and moderate pre-cirrhotic disease stages. However, for the sake of simplicity and in the context of a burden of disease study in which the objective is to compare a broad spectrum of diseases, a single, chronic hepatitis health outcome was applied.

Risk of complications

Compensated cirrhosis (CC)

The risk of HCV-infected persons developing cirrhosis within 20–30 years is estimated in most studies to be within the range of between 5 and 20%, although some studies give estimates of up to 50% (CDC, 2011; Freeman 2001; Freeman 2003; Lauer & Walker, 2001; Poynard, 1997; Poynard, 2001; Thein, 2008; Wasmuth, 2010). Thein et al. predicted via meta-analysis an average 20-year cirrhosis risk of 16% (95% CI: 14%-19%), and a 30-year risk of 41% (95% CI: 36%–45%), which underlines that the progression to cirrhosis is not a linear process (Thein et al. 2008).

The annual risk of progressing to compensated cirrhosis was calculated based on the transitional probabilities between the five METAVIR stages of fibrosis, as estimated by Thein et al. (2008), using random-effect meta-analysis applied to non-clinical studies only. The point estimate for the risk of developing compensated cirrhosis from chronic hepatitis, calculated as the inverse of the summed durations in the first four METAVIR stages (each duration in turn was estimated as 1/probability of leaving the METAVIR stage), was 1.9% per year. The disability duration was calculated at 36.5 years; this is the average time taken for 50% of those with chronic hepatitis to exit the compartment: $1- \exp(-0.019 * 36.5) = 0.5$.

Decompensated cirrhosis (DC)

HCV-associated cirrhosis leads to liver failure and death in about 20–25% of cirrhotic cases. The annual risk of compensated cirrhosis progressing to the decompensated stage (characterised by ascites, bleeding oesophagal varices, or jaundice) is estimated to be 3.9–7% (D'Amico, 2006; Fattovich, 1997; Grieve, 2006; Poynard, 1997; Wasmuth, 2010). In the model, hepatic decompensation was assumed to occur with an annual risk of 3.9 to 12.9 (Dienstag, 2011).

Without transplantation the prognosis is poor. The five-year survival rate with decompensated liver cirrhosis is roughly 50% (Planas, 2004). One report based on a small study population (n=65) estimated the annual mortality rate at 12.9% (Fattovich et al. 1997), but higher values were reported in the systematic review by D'Amico et al. (2006) (20% 1-year mortality from the first stage of DC; 55% from the second DC stage, which is indicated by bleeding varices with or without ascites). The estimated annual risk of death from DC was set to within a range of 13– 38.5% (Fattovich, 1997; Grieve, 2006; D'Amico, 2006); the upper bound was calculated as the mean of the rates for the two DC stages reported by D'Amico et al (2006).

Duration of DC is based on average waiting time for liver transplant in the UK and in Spain which are represented as an average duration (142 days, NHS and 124 days, Matesanz 2009).

Hepatocellular carcinoma (HCC)

In contrast to hepatitis B, development of primary liver cancer, or hepatocellular carcinoma (HCC), is rare in patients with chronic hepatitis C who do not have cirrhosis (Lauer & Walker, 2001; Spengler, 2010; Wasmuth, 2010; WHO, 2002). Once cirrhosis is established, the risk of hepatocellular carcinoma is estimated to be 1–4% per year (Fattovich et al. 1997; Lauer & Walker, 2001). Studies modelling the natural course of hepatitis C have assumed annual risks of around 1.5% (Grieve et al. 2006; Siebert et al. 2003).

HCC is an outcome that can occur after either the compensated or decompensated cirrhosis stages. The annual risk of developing HCC following either CC or DC was set to 3%, based on the estimate by D'Amico et al. (2006).

Studies modelling the natural course of hepatitis C have assumed annual case fatality rates (CFR) due to liver cancer ranging widely from 43– 86% (Grieve, 2006; Siebert, 2003; Wong, 2000). This variation might be a consequence of stage and treatment-specific survival rates, and other underlying conditions including alcohol consumption, diabetes or obesity, where the higher estimate is used to simulate a situation without early diagnosis and effective treatment. In the model, this CFR is set to 48.9%/year, based on the 1-year survival rate (Kwong, 2012).

Other complications

Fulminant hepatic failure due to acute HCV infection is considered to be very rare (CDC, 2011; Lauer & Walker, 2001; Wasmuth, 2010; World Health Organization, 2002) except in cases of HBV coinfection (Chu, 1999). Fulminant liver failure and death was reported to occur in approximately 0.1% (2/1536) of adults with reported (notified) acute hepatitis C (Bianco, 2003). Due to this condition being extremely rare, no health outcome was specified in the outcome tree.

Model input summary

Table 1. Transition probabilities and distributions used in the outcome tree

Health outcome (Health state)	Distribution of health states in health outcome	Transition probability	Source/assumption
Symptomatic infection	20–30%		CDC, 2011
Chronic hepatitis		> 19yr: 75.6% (67.3–84.9)<br>< 20yr: 55–69%	Alter, 1994; Alter, 2000<br>Age dependent
Remission from chronic hepatitis		0.31 (0.26–0.36)%/year	Micallef, 2006 (ATP)
Compensated cirrhosis		1.9%/year	Modelled from Thein, 2008 (ATP)
Decompensated cirrhosis		3.9–12.9%/year	Dienstag, 2011 (ATP)
HCC, following<br>- Compensated cirrhosis<br>- Decompensated cirrhosis		3.0%/year<br>3.0%/year	D'Amico, 2006 (ATP)<br>D'Amico, 2006 (ATP)
CFR, following:<br>- Decompensated cirrhosis<br>- Hepatocellular carcinoma		13–38.5%/year<br>48.9%/year	Fattovich, 1997; Grieve 2006;<br>D'Amico 2006 (ATP)<br>Kwong, 2012 (ATP)

Table 2. Disability weights and duration

Health outcome (Health state)	Disability Weight (DW) (Haagsma, 2015)		Duration	
	DW	Label	In years	Source
Symptomatic infection	0.051 (0.039–0.06)	Infectious disease, acute episode, moderate	0.038–0.23	CDC, 2011; Wasmuth, 2010; World Health Organization, 2002
Chronic hepatitis	0.07 (0.057–0.088)	Generic uncomplicated disease: worry and daily medication	36.5	Modelled from Thein, 2008
Compensated cirrhosis	0.07 (0.057–0.088)	Generic uncomplicated disease: worry and daily medication	6-10.4<br>See table 3	Murray, 1996<br>Age and gender specific
Decompensated cirrhosis	0.163 (0.136–0.194)	Decompensated cirrhosis of the liver	0.34–039	Assuming average waiting time before liver transplantation in UK and Spain (NHS and Matesanz 2009)
Hepatocellular carcinoma	0.265 (0.222–0.303)	Cancer, diagnosis and primary therapy	0.72–4.48<br>See table 4	Murray, 1996<br>Age and gender specific

Table 3. Duration of compensated cirrhosis

Age group	Duration (years)	
	F	M

0-4	10.4	10.3
5-14	10.4	10.4
15-44	10.2	10
45-59	9.3	8.8
60+	6.5	6

Table 4. Duration of hepatocellular carcinoma

Age group	Duration (years)	
	F	M
0-14	4.48	4.11
15-44	1.45	2.92
45-59	1.91	2.88
60+	0.72	1.56

References

Alberti A, Vario A, Ferrari A, Pistis R. (2005). Review article: chronic hepatitis C--natural history and cofactors. Aliment Pharmacol Ther 22 Suppl 2, 74-78.

Alter MJ, Kruszon-Moran D, Nainan OV, McQuillan GM, Gao F, Moyer LA, et al. The prevalence of hepatitis C virus infection in the United States, 1988 through 1994. Engl J Med. 1999 Aug 19; 341(8):556-62.

Alter HJ, Seeff LB. (2000). Recovery, persistence, and sequelae in hepatitis C virus infection: a perspective on long-term outcome. Semin Liver Dis 20, 17-35.

Bianco E, Stroffolini T, Spada E, Szklo A, Marzolini F, Ragni P, et al. (2003). Case fatality rate of acute viral hepatitis in Italy: 1995-2000. An update. Dig Liver Dis 35, 404-408.

Centers for Disease Control and Prevention (CDC). Hepatitis C: Information for Health Professionals. 2011. Available at: http://www.cdc.gov/hepatitis/HCV/index.htm.

Chu CM, Yeh CT, Liaw YF. Fulminant hepatic failure in acute hepatitis C: increased risk in chronic carriers of hepatitis B virus. Gut 1999;45:613- 617.

Crofts N, Jolley D, Kaldor J, et al. Epidemiology of hepatitis C virus infection among injecting drug users in Australia. J Epidemiol Community Health 1997;1:692–697.

D'Amico G, Garcia-Tsao G, Pagliaro L. Natural history and prognostic indicators of survival in cirrhosis: a systematic review of 118 studies. J Hepatol. 2006;44(1):217-31.

Dienstag JL, Ghany MG, Morgan TR, Di Bisceglie AM, Bonkovsky HL, Kim HY, et al. and the HALT-C Trial Group: A prospective study of the rate of progression in compensated, histologically advanced chronic hepatitis C. Hepatology 2011, 54:396–405

ECDC. Surveillance and prevention of hepatitis B and C in Europe. Stockholm: ECDC 2010.

Fattovich G, Giustina G, Degos F, Tremolada F, Diodati G, Almasio P, et al. (1997). Morbidity and mortality in compensated cirrhosis type C: a retrospective follow-up study of 384 patients. Gastroenterology 112, 463-472.

Freeman AJ, Dore GJ, Law MG, Thorpe M, Von Overbeck J, Lloyd AR et al. Estimating progression to cirrhosis in chronic hepatitis C virus infection. Hepatology 2001;34:809-816.

Freeman AJ, Law MG, Kaldor JM, and Dore GJ. Predicting progression to cirrhosis in chronic hepatitis C virus infection. J Viral Hepat 2003;10:285-293.

Grieve R, Roberts J, Wright M, Sweeting M, DeAngelis D, Rosenberg W, et al. Cost effectiveness of interferon alpha or peginterferon alpha with ribavirin for histologically mild chronic hepatitis C. Gut 2006;55:1332-1338.

Hagan H, Snyder N, Hough E, Yu T, McKeirnan S, Boase J, et al. Case-reporting of acute hepatitis B and C among injection drug users. J Urban Health 2002;79(4):579-85.

Haagsma JA, Maertens de Noordhout C, Polinder S, Vos T, Havelaar AH, Cassini A, Devleesschauwer B, Kretzschmar ME, Speybroeck N, Salomon JA. Assessing disability weights based on the responses of 30,660 people from four European countries. Population Health Metrics 2015; 13: 10

Kwong JC, Ratnasingham S, Campitelli MA, Daneman N, Deeks SL, et al. (2012) The Impact of Infection on Population Health: Results of the Ontario Burden of Infectious Diseases Study. PLoS ONE 7(9): e44103. doi:10.1371/journal.pone.0044103

Lauer GM, Walker BD. Hepatitis C virus infection. N Engl J Med. 2001;345:41-52.

Mann AG, Ramsay ME, Brant LJ, Balogun MA, Costella A, Harris HE. Diagnoses of, and deaths from, severe liver disease due to hepatitis C in England between 2000 and 2005 estimated using multiple data sources. Epidemiol Infect. 2009;137(4):513-8.

Matesanzemail R, de la Rosa G. Liver transplantation: The Spanish experience. Digestive and Liver Disease Supplements. Volume 3, Issue 4 , Pages 75-81, December 2009.

McDonald SA, Hutchinson SJ, Mills PR, et al. Diagnosis of hepatitis C virus infection in Scotland's injecting drug user population. Epidemiol. Infect 2010;138:393–402.

Micallef JM, Kaldor JM, Dore GJ (2006) Spontaneous viral clearance following acute hepatitis C infection: a systematic review of longitudinal studies. J Viral Hepat 13: 34–41.

Murray CJL, Lopez AD, World Health Organization., World Bank., Harvard School of Public Health.: Global health statistics: a compendium of incidence, prevalence and mortality estimates for over 200 conditions. Geneva: World Health Organization; 1996.

Murray CJL, Lopez AD: The Global Burden of Disease - A comprehensive assessment of mortality and disability from diseases, injuries, and risk factors in 1990 and projected to 2020. Cambridge: Harvard School of Public Health on behalf of the World Health Organization and the World Bank; 1996.

National Health System (NHS), UK. Available at: http://www.nhs.uk/conditions/Liver-transplant/Pages/Introduction.aspx(accessed 31 July 2014)

Okuda K, Ohtsuki T, Obata H, Tomimatsu M, Okazaki N, Hasegawa H. et al. (1985). Natural history of hepatocellular carcinoma and prognosis in relation to treatment. Study of 850 patients. Cancer 1985;56:918-928.

Planas R, Balleste B, Alvarez MA, Rivera M, Montoliu S, Galeras JA, et al. Natural history of decompensated hepatitis C virus-related cirrhosis. A study of 200 patients. J Hepatol. 2004;40823-830.

Poynard T, Bedossa P, Opolon P. Natural history of liver fibrosis progression in patients with chronic hepatitis C. The OBSVIRC, METAVIR, CLINIVIR, and DOSVIRC groups. Lancet 1997;349:825-832.

Poynard T, Ratziu V, Charlotte F, Goodman Z, McHutchison J, Albrecht J. Rates and risk factors of liver fibrosis progression in patients with chronic hepatitis c. J Hepatol. 2001;34:730-739.

Siebert U, Sroczynski G, Rossol S, Wasem J, Ravens-Sieberer U, Kurth BM, et al. (2003). Cost effectiveness of peginterferon alpha-2b plus ribavirin versus interferon alpha-2b plus ribavirin for initial treatment of chronic hepatitis C. Gut 52, 425-432.

Spengler U. (2010). Hepatocellular Carcinoma - Diagnosis, Prognosis & Therapy. In Hepatology - A Clinical Textbook. S. Mauss, T. Berg, J. Rockstroh, C. Sarrazin, and H. Wedemeyer, eds. (Düsseldorf, Flying Publisher).

Strauss R, Fülöp G, Pfeifer C. Hepatitis C in Austria 1993–2000: reporting bias distort HCV epidemiology in Austria. Euro Surveill. 2003;8(5):pii=412.

Thein HH, Yi Q, Dore GJ, Krahn MD. (2008). Estimation of stage-specific fibrosis progression rates in chronic hepatitis C virus infection: a meta- analysis and meta-regression. Hepatology 48, 418-431.

Wasmuth, J. (2010). Hepatitis C - Epidemiology, transmission and natural history. In Hepatology - A Clinical Textbook. S. Mauss, T. Berg, J. Rockstroh, C. Sarrazin, and H. Wedemeyer, eds. (Düsseldorf, Flying Publisher).

Wong JB, McQuillan GM, McHutchison JG, Poynard T. (2000). Estimating future hepatitis C morbidity, mortality, and costs in the United States. Am J Public Health 90, 1562-1569.

World Health Organization (2002). Hepatitis C. Available at: http://www.who.int/csr/disease/hepatitis/whocdscsrlyo2003/en/index.html. Accessed: February 2011.

Yousuf M, Nakano Y, Tanaka E, Sodeyama T, Kiyosawa K. Persistence of viremia in patients with type-C chronic hepatitis during long-term follow-up. Scand J Gastroenterol. 1992;27:812-816.

HIV

Acquired Immunodeficiency Syndrome (AIDS) is the most severe outcome of an untreated HIV infection. AIDS presents with severe opportunistic infections, malignancies, neurological complications or other HIV-induced disease conditions (Del Rio & Curran, 2005). After infection with HIV, individuals may remain asymptomatic or develop Acute Retroviral Syndrome (ARS) (Del Rio & Curran, 2005). ARS occurs in 50–66% of all recently infected cases (Sterling & Chaisson, 2005). Due to mild and non-specific flu-like symptoms many people do not seek medical advice, and thus are not diagnosed and treated and proceed to a latent stage where they may remain asymptomatic for years before subsequently developing AIDS.

Within the EU, it is estimated that around 8–45% of all HIV infections are undiagnosed and therefore not reported to the health authorities (ECDC, 2014). The overall duration is difficult to estimate because since introduction of Anti-Retroviral Therapy (ART) HIV is increasingly observed as being a chronic disease and individuals receiving treatment have a similar life expectancy to the rest of the population in Europe (Bhaskaran, 2008). Persistent asymptomatic HIV infection is estimated to be on average 17.2 years for long-term non-progressors (Herida, 2006).

Health outcomes/states associated with HIV-infection

HIV is associated with a heterogeneous set of health outcomes/states. In most cases, certain health outcomes/states are caused by subsequent infections with a secondary or tertiary pathogen. HIV compromises the immune status of an individual and thus increases the risk of further additional pathogens causing severe sequelae.

For our study, we considered that in Europe development of AIDS is significantly limited through ART.

HIV/AIDS is a complicated, multi-faceted and systemic disease and for reasons of feasibility, we developed a simplified model which does not differentiate between the CD4 count stages of the disease at the point of diagnosis, even though this is known to affect mortality (Aghaizu, 2013). Moreover, the current model does not take into account transmitted drug resistance, or the issue of co-morbidity (HIV–HCV or HIV–TB) and the consequent need for a specific therapeutic pathway. We assumed that all diagnosed cases are offered treatment and we applied a certain burden to the disease (e.g. side effects).

HIV infection-related deaths are associated with the development of an acquired immunodeficiency syndrome (AIDS) which, after a prolonged latent period, eventually enables opportunistic infections to develop which are generally the cause of death. Therefore, the nature of AIDS itself consists of comorbidities introducing the issue of attributable cause of death. However, we assumed that the severity of the co-infection and the precipitation to death would not have occurred without the primary HIV infection and deaths were therefore attributed entirely to the initial HIV infection. We also did not include the burden associated with HIV-related malignancies or complications linked to long-term antiretroviral therapy (e.g. cardiovascular disease).

Outcome-tree parameters

The main input is 'persistent HIV infection' and this is subdivided according to the speed of progression (Qu, 2008). In general, 5–15% of all patients are rapid progressors (RP) and are at risk of developing AIDS within 2–5 years (Qu, 2008). Another 5–15% are long-term non- progressors (LNP) with, on average 17.2 years duration of development (Qu, 2008; Sterling & Chaisson, 2005). The remainder (70–90%) are typical progressors (TP) with an average duration of 8–10 years (Qu, 2008).

The risk of developing early symptomatic AIDS is set at between 4.5% and 7% (Grinsztejn, 2014: 40 observed cases out of 886 in the group with early ART initiation versus 61 out of 877 in the delayed group).

Terminal AIDS has a duration of one month (Kwong, 2010) and the risk of developing terminal AIDS from early symptomatic AIDS is set at 32.09% as this was the case fatality proportion estimated for AIDS in a recent study (Serraino, 2010).

Model input summary

Table 1. Transition probabilities and distributions used in the outcome tree

Health outcome (Health state)	Distribution of health states in health outcome	Transition probability	Source/assumption
Persistent HIV infection			No cure available
(Rapid progressors)	5–15%		Qu, 2008
(Typical progressors)	70–90%		Qu, 2008
(Long-term non-progressors)	5–15%		Qu, 2008; Herida, 2006
AIDS early symptomatic		4.5–7%	Grinsztejn, 2014
AIDS terminal phase		32.09%	Serraino, 2010
CFR from AIDS		100%	

Table 2. Disability weights and duration

	Disability Weight (DW) (Haagsma, 2015)		Duration

Health outcome (Health state)	DW	Label	In years	Source
Persistent HIV infection	0.108 (0.089-0.132)	HIV/AIDS cases, receiving ARV treatment		
(Rapid progressors)			2-5	Qu, 2008
(Typical progressors)			8-10	Qu, 2008
(Long-term non-progressors)			17.2	Qu, 2008; Herida, 2006
Permanent ARV treatment	0.108 (0.089-0.132)	HIV/AIDS cases, receiving ARV treatment	Remaining life expectancy	Assuming ARV treatment has optimal effectiveness and good compliance
AIDS early symptomatic	0.351 (0.299–0.394)	HIV cases, symptomatic, pre-AIDS	5.36	Herida, 2006
AIDS terminal phase	0.574 (0.518–0.635)	AIDS cases, not receiving ARV treatment	0.08	Kwong, 2010

References

Aghaizu A, Brown AE, Nardone A, Gill ON, Delpech VC & contributors. HIV in the United Kingdom 2013 - Report: data to end 2012. November 2013. Public Health England, London. Available at: https://www.gov.uk/government/uploads/system/uploads/attachment_data/file/326601/HIV_annual_report_2013.pdf

Barre-Sinoussi F, Chermann JC, Rey F, Nugeyre MT, Chamaret S, Gruest J, et al. Isolation of a T-lymphotropic retrovirus from a patient at risk for acquired immune deficiency syndrome (AIDS). Science 1983, 220:868-871.

Bhaskaran K, Hamouda O, Sannes M, Boufassa F, Johnson AM, Lambert PC, et al. for the CASCADE Collaboration. Changes in the Risk of Death After HIV Seroconversion Compared With Mortality in the General Population. JAMA. 2008;300(1):51-59. doi:10.1001/jama.300.1.51.

Cameron DW, Simonsen JN, D'Costa LJ, Ronald AR, Maitha GM, Gakinya MN, et al. Female to male transmission of human immunodeficiency virus type 1: risk factors for seroconversion in men. Lancet 1989, 2:403-407.

Cleghorn FR, Reits Jr. MS, Popovic M, Gallo RC. Human Immunodeficiency Viruses. In Principles and Practice of Infectious Diseases. Volume 2. Edited by Mandell GL, Bennet JE, Dolin R. Pennsylvania: Elsevier Inc.; 2005: 2119-2132

HIV/AIDS. Available at: http://www.who.int/topics/hiv_aids/en/

Del Rio C, Curran JW. Epidemiology and Prevention of Acquired Immunodeficiency Virus Infection. In Principles and Practice of Infectious Diseases. Volume 1. Edited by Mandell GL, Bennet JE, Dolin R. Pennsylvania: Elsevier Inc.; 2005: 1477-1506

European Centre for Disease Prevention and Control. Monitoring implementation of the Dublin Declaration on Partnership to Fight HIV/AIDS in Europe and Central Asia: 2014 progress report, in press.

Gallo RC, Sarin PS, Gelmann EP, Robert-Guroff M, Richardson E, Kalyanaraman VS, et al. Isolation of human T-cell leukemia virus in acquired immune deficiency syndrome (AIDS). Science 1983, 220:865-867.

Grinsztejn B, Hosseinipour M, Ribaudo H, Havlir D, Cohen M, et al. (2014). Effects of Early versus Delayed Initiation of Antiretroviral Therapy on HIV Clinical Outcomes: Results from the HPTN 052. The Lancet Infectious Diseases, Volume 14, Issue 4, April 2014, Pages 281-290

Haagsma JA, Maertens de Noordhout C, Polinder S, Vos T, Havelaar AH, Cassini A, Devleesschauwer B, Kretzschmar ME, Speybroeck N, Salomon JA. Assessing disability weights based on the responses of 30,660 people from four European countries. Population Health Metrics 2015; 13: 10

Herida M, Larsen C, Lot F, Laporte A, Desenclos JC, Hamers FF. Cost-effectiveness of HIV post-exposure prophylaxis in France. AIDS 2006, 20:1753-1761.

Kwong JC, Crowcroft NS, Campitelli MA, Ratanasingham S, Daneman N, Deeks SL, et al. Ontario Burden of Infectious Disease Study. In: Ontario Burden of Infectious Disease Study (ed.), Vol. OAHPP/ECES Report. City: Ontario Agency for Health Protection and Promotion and the Institute for Clinical Evaluative Sciences; 2010.

Laga M, Manoka A, Kivuvu M, Malele B, Tuliza M, Nzila N,et al. Non-ulcerative sexually transmitted diseases as risk factors for HIV-1 transmission in women: results from a cohort study. AIDS 1993, 7:95-102.

Merson MH, Piot P. Global Perspectives on Human Immunodeficiency Virus Infection and Acquired Immunodeficiency Syndrome. In Principles and Practice of Infectious Diseases. Volume 1. Edited by Mandell GL, Bennet JE, Dolin R. Pennsylvania: Elsevier Inc.; 2005: 1456-1506

Qu W, Robinson M, Zhang FJ. Factors influencing the natural history of HIV-1 infection. Chin Med J (Engl) 2008, 121:2613-2621.

Serraino D, Bruzzone S, Zucchetta A, Suligoi B, De Paoli A, Pennazza S, et al. Elevated risks of death for diabetes mellitus and cardiovascular diseases in Italian AIDS cases. AIDS Research and Therapy 2010, 7(11)

Sterling TR, Chaisson RE: General Clinical Manifestations of Human Immunodeficiency Virus Infection (Including the Acute Retroviral Syndrome and Oral, Cutaneous, Renal, Ocular and Cardia Diseases). In Principles and Practice of Infectious Diseases. Volume 1. Edited by Mandell GL, Bennet JE, Dolin R. Pennsylvania: Elsevier Inc.; 2005: 1547-1574

Influenza

In most cases influenza infection in humans is uncomplicated and self-limiting within a few days or weeks, but for some patients the disease is fatal. Approximately one third of influenza infections are mild or asymptomatic, to the extent that infected persons do not even see a doctor (Hayward, 2010; Hayward, 2014). Our model assumes a mean duration of five days (Nicholson, 2003).

Wielders et al. (2010) included four different outcomes and their long-term sequelae following acute illness. These were pneumonia, otitis media, acute respiratory distress syndrome (ARDS) and sepsis. The frequency of other post-infectious complications following an influenza infection is low and these were therefore disregarded in the current study. From a clinical perspective, the acute manifestations of the disease often occur in concomitance as complicated cases

Based on information derived from the General Practice Research Database (GPRD), Meier et al. (2000) estimated the number of patients consulting a doctor with symptoms of influenza-like illness (ILI) who developed complications. The percentages were based on subjects who had at least one clinical diagnosis of influenza or influenza-like-illness (ILI) recorded in the GPRD between 1991 and 1996. In addition to the wide range of national case definitions, estimated consultation rates will also vary among countries due to differences in consultation behaviour, estimation procedure (estimation of incidence, given that many surveillance systems are based on sentinel reporting), vaccination coverage (although vaccination has a limited impact on the number of consultations) and obligatory doctor visits for absence from work or school (Harbers, 2005; Meijer et al., 2006). Therefore, doctor consultations were not considered to be indicative of acute complicated influenza disease.

Given very little specific information on the ratio of complicated/uncomplicated acute disease, no distinction was made between these and the variability was accounted for by including all possible manifestations in the disability weight (mild, moderate and severe): 0.051 (0.007–0.125).

Case fatality ratio

Research has shown that clinicians often attribute influenza-related deaths to a pre-existing underlying condition rather than to influenza (Zucs et al., 2005). Therefore, it is difficult to identify true mortality due to influenza only. Distinguishing further between mortality due to influenza with or without complications such as cardiac problems or pneumonia is even more difficult. Therefore in the current study only one category of death was considered, encompassing all causes which, in the model, occur shortly after infection.

For the Netherlands, it was estimated that during the period 1967–1989 the overall impact of influenza on mortality was greater than registered mortality by a factor of 3.6 (Sprenger et al., 1993). Using this multiplication factor for more recent data may overestimate the number of deaths due to influenza, because in many Member States today vaccination coverage is considerably higher than in the period 1967– 1989. In the study by Sprenger et al. almost half of the non-registered influenza deaths were registered as deaths from heart disease, approximately 25% from lung disease and approximately 30% from other diseases (Sprenger et al., 1993). Recently, time series analysis has also been used to estimate mortality attributable to influenza and other respiratory pathogens (van den Wijngaard et al., 2010).

In about 0.1% of all influenza cases the disease will be fatal (Flu.gov, 2012). This includes both uncomplicated and complicated influenza cases.

Approximately 90% of persons with influenza as cause of death were aged ≥ 65 years (Webster, 2013). Therefore, given that the case fatality proportion for influenza is age-dependent, we modelled the age-specific risk according to the observed mortality data in Estonia, Germany and the Netherlands (see Table 3) (CBS, 2009).

Risk of complications

The most vulnerable populations in terms of complications following influenza are children aged under one 1 year and adults over 65 years, pregnant woman, and people of any age with comorbid illnesses (Rothberg et al., 2008).

The most common complications of influenza are secondary bacterial infections, especially otitis media and pneumonia (van Steenbergen, et al., 2006). It is estimated that 0.65% of influenza cases develop otitis media and 0.36% pneumonia (Meier et al., 2000). Secondary

bacterial pneumonia most often complicate the condition 4–14 days after primary seasonal influenza infection (Rothberg et al., 2008). Neurological complications such as encephalopathy (Reye's syndrome), encephalomyelitis, transverse myelitis, aseptic meningitis, focal neurological disorders, and Guillain-Barré syndrome most often appear in small children (Rothberg et al., 2010). The incidence of neurological complications among <5 years was estimated to be 4 per 100 000 (Newland, 2007).

Wielders et al. (2010) assumed that about 1.23% of all influenza cases develop pneumonia. Earlier, van Lier et al. (2007) assumed that this fraction was 0.36%. In most cases the disease will be self-limiting within a few days, and only in a few cases will it be fatal. According to Murray et al. (1996) long-term outcomes of pneumonia in developed countries are very rare and can be disregarded when estimating disease burden.

Wielders et al. (2010) assumed that 0.65% of influenza cases will develop otitis media as a complication of influenza. Most affected persons will fully recover, but 0.006% of otitis media cases will develop deafness as a life-long disability (Murray, 1996). Given the very low risk, we considered this complication as negligible.

A few cases will develop sepsis during an influenza infection, estimated at 0.0097% of all cases (Wielders, 2010). In some cases the disease will be fatal but again, since there was no detailed information available on the percentage, we assumed that fatal cases would be included in the death estimate related to influenza. Long-term disability was estimated to occur in 82% of patients surviving sepsis (Korosec Jagodic, 2006). However, given the fact that sepsis is caused by bacteria giving rise to super-infections possibly related to other factors, the long-term sequelae of sepsis are not considered to be part of the burden of influenza infections.

Acute respiratory distress syndrome (ARDS) and life-long disability

Following Wielders et al. (2010), we assumed that 0.023% of influenza cases will develop ARDS as a complication of influenza. We assumed that the risk of developing ARDS changes according to age (Manzano, 2005). Wielder's study, however, does not consider cases <15 years and in order to account for these, we also included a study on younger populations (Zimmerman, 2009). We combined the ARDS incidence from the two studies, added them together and estimated the age-group risk of developing ARDS (see Table 4).

In a few cases the disease will be fatal. However, having no detailed information on the specific risk, we assumed that fatal cases would be included in the death estimate related to influenza. Around 30–55% (Hopkins, 1999; Mikkelsen, 2012) of patients surviving ARDS will have developed disabilities related to cognitive impairments at one year follow-up. Therefore, in our model, we estimated that 0.007–0.013% of all symptomatic influenza cases will develop cognitive sequelae assumed to be permanent.

Model input summary

Table 1. Transition probabilities used in the outcome tree

Health outcome (Health state)	Distribution of health states in health outcome	Transition probability	Source/assumption
Permanent disability due to ARDS		0.007–0.013% Age dep. (Table 4)	Wielders, 2010; Manzano, 2005; Hopkins, 1999; Mikkelsen, 2012
Fatal cases		0.10% Age dep. (Table 3)	Flu.gov, 2012; observed cases

Table 2. Disability weights and duration

Health outcome (Health state)	Disability Weight (DW) (Haagsma, 2015)		Duration	
	DW	Label	In years	Source
Symptomatic infection	0.051 (0.007–0.125)	Infectious disease, acute episode, from mild to severe	0.014	Nicholson, 2003
Permanent disability due to ARDS	0.056 (0.044–0.067)	Motor plus cognitive impairments, mild	Remaining life expectancy	Hopkins, 1999; Mikkelsen, 2012

Table 3. Age group distribution of 0.1% risk of fatal cases

Age	%
0	0.58
01-04	0.51
05-09	0.24
10-14	0.27
15-19	0.24

20-24	0.33
25-29	0.31
30-34	0.33
35-39	0.75
40-44	1.15
45-49	1.56
50-54	1.53
55-59	2.21
60-64	3.23
65-69	4.54
70-74	5.22
75-79	11.42
80-84	18.72

85+	46.85

Source: based on all reported fatal influenza cases in Estonia, Germany and the Netherlands for the years 2005–2007.

Table 4. Age group distribution of 0.007–0.013% risk of developing ARDS

Age	%
0-14	7.21
15-29	2.59
30-44	7.66
45-59	12.17
60-74	28.73
≥75	41.63

References

CBS (Statistics Netherlands). Causes of death, 2009. Available at: http://statline.cbs.nl/StatWeb/

U.S. Department of Health & Human Services, Flu.gov (US). Community strategy for pandemic influenza mitigation. 2007 Feb.www.flu.gov/professional/community/mitigation.html [cited 12 March 2010]

Haagsma JA, Maertens de Noordhout C, Polinder S, Vos T, Havelaar AH, Cassini A, Devleesschauwer B, Kretzschmar ME, Speybroeck N, Salomon JA. Assessing disability weights based on the responses of 30,660 people from four European countries. Population Health Metrics 2015; 13: 10

Harbers MM. Influenza: are there international differences? In: Public Health Forecast, National Compass Public Health [Available at http://www.rivm.nl/vtv/object_document/o1736n18081.html]. 2005. Bilthoven: National Institute for Public Health and the Environment (RIVM).

Hayward AC et al. The community burden of influenza and Influenza-Like-Illness in England – Early results from the MRC Flu Watch Study. 2010. Personal communication with Professor Hayward.

Hayward AC, Fragaszy EB, Bermingham A, Wang L, Copas A, Edmunds WJ, et al. Comparative community burden and severity of seasonal and pandemic influenza: Results of the Flu Watch cohort study. Lancet Respir. Med. 2014, 2014, doi:10.1016/S2213-2600(14)70034-70037.

Hopkins RO, Weaver LK, Pope D, Orme JF Jr., Bigler ED, Larson-Lohr V. Neuropsychological sequelae and impaired health status in survivors of severe acute respiratory distress syndrome. American Journal of Respiratory and Critical Care Medicine. 1999;160(1):50–56.

Korosec Jagodic H, Jagodic K, Podbregar M. Long-term outcome and quality of life of patients treated in surgical intensive care: a comparison between sepsis and trauma. Crit Care 2006;10: R134.

Manzano F, Yuste E, Colmenero M, Garcia-Horcajadas A, Rivera R, Fernandez-Mondejar E. Granada Respiratory Failure Study Group: Incidence of acute respiratory distress syndrome and its relation to age. J Crit Care. 2005;20:274–80.

Meier CR, Napalkov PN, Wegmuller Y, Jefferson T, Jick H. Population-based study on incidence, risk factors, clinical complications and drug utilisation associated with influenza in the United Kingdom. Eur J Clin Microbiol Infect Dis 2000. 19 (11): 834-42.

Meijer A, Paget WJ, Meerhoff TJ, Brown C, Meuwissen LE, Van Der Velden J. Epidemiological and virological assessment of influenza activity in Europe, during the 2004-2005 winter. Euro Surveill 2006;11(5): 111-8.

Mikkelsen ME, Christie JD, Lanken PN, Biester RC, Thompson BT, Bellamy SL, et al. The adult respiratory distress syndrome cognitive outcomes study: long-term neuropsychological function in survivors of acute lung injury. Am J Respir Crit Care Med. 2012;17:1307–1315. doi: 10.1164/rccm.201111-2025OC.

Murray CJL, Lopez AD (eds.) (1996). Global health statistics: a compendium of incidence, prevalence and mortality estimates for over 200 conditions (Global burden of disease and injuries series; II). Cambridge: Harvard School of Public Health on behalf of the World Health Organization and the World Bank.

Neurologic Complications in Children Hospitalized with Influenza: Characteristics, Incidence, and Risk Factors. Newland JG, Laurich VM, Rosenquist AW, Heydon K, Licht DJ, Keren R et al. The Journal of Pediatrics, 2007; Vol. 150, 3,306–310

Nicholson KG, Wood JM, Zambon M. Influenza. Lancet 2003; 362:1733-45.

Rothberg MB, Haessler SD, Brown RB (2008). Complications of viral influenza. Am J Med 121(4): 258-64.

Rothberg MB, Haessler SD (2010). Complications of seasonal and pandemic influenza. Crit Care Med 38(4 Suppl): e91-7.

Sprenger MJ, Mulder PG, Beyer WE, Van Strik R, Masurel N (1993). Impact of influenza on mortality in relation to age and underlying disease, 1967-1989. Int J Epidemiol 22(2): 334-40.

van den Wijngaard CC, van Asten L, Meijer A, van Pelt W, Nagelkerke NJ, Donker GA, et al (2010). Detection of excess influenza severity: associating respiratory hospitalization and mortality data with reports of influenza-like illness by primary care physicians. Am J Public Health 100(11): 2248-54.

van Lier EA, Havelaar AH (2007). Disease burden of infectious diseases in Europe: a pilot study. Bilthoven: National Institute for Public Health and the Environment (RIVM) (RIVM report 215011001).

van Steenbergen J, Timen EA (eds) (2006). Guidelines Infectious Disease Control Edition 2006. Bilthoven: LCI, Coordinator Infectious Disease Control for the Netherlands.

Webster, Monto, Bracciale, Lamb. Textbook of Influenza. 2nd Edition, 2013. Wiley Blackwell Ed.

Wielders CCH, van Lier EA, van 't Klooster TM, van Gageldonk-Lafeber AB, van den Wijngaard CC, Haagsma JA, et al. The burden of 2009 pandemic influenza A(H1N1) in the Netherlands. Eur J Public Health 2010. http://dx.doi.org/10.1093/eurpub/ckq187

Zimmerman JJ, Akhtar SR, Caldwell E, Rubenfeld GD. Incidence and outcomes of pediatric acute lung injury. Pediatrics. 2009;124:87–95.

Zucs P, Buchholz U, Haas W, Uphoff H. Influenza associated excess mortality in Germany, 1985-2001. Emerg Themes Epidemiol 2005.2:6.

Invasive haemophilus influenza disease

The major disease burden of invasive H. influenzae infection occurs in children under five years (Fogarty, 1995). The most harmful complication is bacteraemia, which is accompanied by a focal infection such as meningitis, pneumonia, or cellulitis in 30–50% of cases (Devarajan, 2009).

Risk of complications

Meningitis is the principal clinical presentation of invasive disease, but bone and joint infections, pneumonia, epiglottitis, cellulitis and septicaemia can also occur. Skin and soft tissue infections may occur in around 6% of patients, followed by a limited number of sequelae (Otero Reigada, 2005). Only the invasive forms are considered as health states in the model.

To estimate the risk of meningitis we used the surveillance data reported in the ECDC Invasive Disease Surveillance report on clinical presentations of the acute symptomatic disease (ECDC, 2013a; ECDC, 2013b). Reported data indicates that meningitis and septicaemia occur together in 0–1% of cases, whereas meningitis alone occurs in 15–18% (15% in 2010, 18% in 2011) of cases, resulting in an overall risk of 15– 18% of developing meningitis. The risk of developing meningitis during the acute phase of the disease is age-specific. Age and gender-specific data were extracted from ECDC's TESSy database on the meningitis complications of IHID for 2010 and 2011 (see Table 4). The risk of developing the long-term sequelae is age and gender-specific.

Long-term sequelae

Bacterial meningitis may cause long-term sequelae and permanent disabilities. To investigate this we extracted the risk of developing these complications after meningitis episodes from Edmond et al. (Edmond, 2010).

Meningitis accounts for various long-term sequelae (each of which is multiplied by the risk of developing meningitis during the acute phase of the disease: 15–18%): cognitive difficulties (0.17–0.20%), seizure disorders (0.23–0.27%), hearing loss (0.48–0.58%), motor deficit (0.33– 0.40%), visual disturbance (0.08–0.09%), behavioural problems (0.32–0.38%), clinical impairments (0.18–0.22%) and multiple impairments (0.39–0.47%) (Edmond, 2010).

Case fatality proportion

The parameters for the case fatality proportion were based on data for EU/EEA countries in 2011, see Table 3 (ECDC, 2013).

Model input summary

Table 1. Transition probabilities used in the outcome tree

Health outcome<br>(Health state)	Distribution of health states in health outcome	Transition probability	Source/assumption
Hearing loss		0.48-0.58%	Edmond, 2010

Cognitive difficulties		0.17-0.20%	Edmond, 2010
Seizure disorder		0.23-0.27%	Edmond, 2010
Motor deficit		0.33-0.40%	Edmond, 2010
Visual disturbance		0.08-0.09%	Edmond, 2010
Behavioural problems		0.32-0.38%	Edmond, 2010
Clinical impairments		0.18-0.22%	Edmond, 2010
Multiple impairments		0.39-0.47%	Edmond, 2010
Fatal cases due to symptomatic infection		See Table 3 (5.4-19.5%)	ECDC, 2013

Table 2. Disability weights and duration

Health outcome (Health state)	Disability Weight (DW) (Haagsma, 2015)		Duration	
	DW	Label	In years	Source
Symptomatic infection	0.655 (0.579-0.727)	Intensive care unit admission	0.019	Tunkel, 2004 Assuming the duration of antimicrobial therapy

Permanent disability following meningitis			Remaining life expectancy	
1. Hearing loss	0.008-0.103	From lowest to highest hearing loss related DWs		
2. Cognitive difficulties	0.044-0.188	From lowest to highest intellectual disability related DWs		
3. Seizure disorder	0.07 (0.057-0.088)	Generic uncomplicated disease: worry and daily medication		
4. Motor deficit	0.011-0.421	From lowest to highest motor impairment related DWs		
5. Visual disturbance	0.004-0.171	From lowest to highest vision impairment related DWs		
6. Behavioural problems	0.088 (0.07-0.108)	Subacute sclerosing panencephalitis – phase 1 (assuming best fitting health state description)		
7. Clinical impairments	0.004-0.421	From lowest to highest DW included in this model		
8. Multiple impairments	0.004-0.421	From lowest to highest DW included in this model		

Table 3. CFR following symptomatic infection

Age	CFR
0	19.5%
1-4	6.5%
5-14	5.7%
15-64	5.4%
≥65	15%

Table 4. Age specific distribution per gender of the 15-18% risk of developing meningitis manifestation during the symptomatic infection (TESSy 2010-2011)

Age group		%

	F	M
0	15.69	17.12
01-04	15.69	18.49
05-09	2.61	5.48
10-14	1.31	2.74
15-19	1.31	2.74
20-24	2.61	4.11
25-29	0.00	0.00
30-34	3.27	1.37
35-39	1.96	4.79
40-44	3.92	7.53
45-49	3.92	8.22
50-54	7.19	2.74
55-59	7.19	2.74
60-64	3.27	4.79
65-69	10.46	3.42
70-74	11.11	5.48
75-79	5.23	4.11
80-84	2.61	1.37
85+	0.65	2.74
Total	100	100

References

Buysse CM, Oranje AP, Zuidema E, Hazelzet JA, Hop WC, Diepstraten AF, et al. Long-term skin scarring and orthopaedic sequelae in survivors of meningococcal septic shock. Arch Dis Child. 2009 May;94(5):381-6.

Devarajan VR. Haemophilus Influenzae Infections. 2009. http://emedicine.medscape.com/article/218271-overview

Edmond K, Clark A, Korczak VS, Sanderson C, Griffiths UK, Rudan I. Global and regional risk of disabling sequelae from bacterial meningitis: a systematic review and meta-analysis. Lancet Infect Dis. 2010 May;10(5):317-28.

Eidelman LA, Putterman D, Putterman C, Sprung CL. The Spectrum of Septic Encephalopathy: Definitions, Etiologies, and Mortalities. JAMA. 1996;275(6):470-473. doi:10.1001/jama.1996.03530300054040.

European Centre for Disease Prevention and Control. Surveillance of invasive bacterial diseases in Europe, 2010. Stockholm: ECDC; 2013.

European Centre for Disease Prevention and Control. Surveillance of invasive bacterial diseases in Europe, 2011. Stockholm: ECDC; 2013.

Fogarty J, Moloney AC and Newell JB. The epidemiology of Haemophilus influenzae type b disease in the Republic of Ireland. Epidemiol. Infect. (1995). 114. 451-463.

Haagsma JA, Maertens de Noordhout C, Polinder S, Vos T, Havelaar AH, Cassini A, Devleesschauwer B, Kretzschmar ME, Speybroeck N, Salomon JA. Assessing disability weights based on the responses of 30,660 people from four European countries. Population Health Metrics 2015; 13: 10

Jit M. The risk of sequelae due to pneumococcal meningitis in high-income countries: a systematic review and meta-analysis. J Infect 2010;61(2):114-24.

Otero Reigada MC, Piqueras Arenas AI, Ferrer Lorente B, Pérez-Tamarit D, Asensi Botet F, Santos Durantez M. Skin and soft tissue infections caused by Haemophilus influenzae type b over a 30 year period. An Pediatr (Barc). 2005 Jul;63(1):29-33.

Tunkel AR, Hartman BJ, Kaplan SL, Kaufman BA, Roos KL, Scheld WM, et al. Practice guidelines for the management of bacterial meningitis. Clin Infect Dis 2004, 39(9):1267–1284.

Ziaja M. Septic encephalopathy. Curr Neurol Neurosci Rep. 2013;13:383

Invasive meningococcal disease (IMD)

As many as 10% of adolescents and adults are asymptomatic transient carriers of N. meningitidis, most strains of which are not pathogenic. In more than 99% of the cases the infection is asymptomatic, but about 1% of the those infected develop acute illness (CDC, 2009). Invasive disease usually requires a seven-day course of antibiotic therapy (Brigham & Sandora, 2009; Tunkel 2004), but may also result in lifelong major sequelae.

Risk of complications

Meningitis is the most common manifestation of invasive disease, and may occur in 47.3% of all patients suffering from N. meningitidis symptomatic infection and in 52.2% of the patients who develop bacteraemia. It always follows hematogenous dissemination, which occurs in 91% of all patients suffering from symptomatic infection. Sepsis occurs in 5–20% of patients with invasive disease (CDC, 2009). Complications are also possible with non-invasive disease; pneumonia occurs in 6% of symptomatic infections, otitis media in 1% of cases and epiglottitis, which is rare, in 0.3% of all manifestations (CDC, 2009).

We decided to use surveillance data reported to TESSy on clinical presentations of the acute symptomatic disease to estimate the risk of meningitis (ECDC, 2013). Reported data indicates that meningitis and septicaemia together occur in 17–18% of cases, whereas meningitis alone occurs in 43–45% of cases, resulting in an overall risk of 60–63% of developing meningitis. The risk of developing meningitis during the acute phase of the disease is age-specific. Age-specific data were extracted for each gender from ECDC's TESSy database on the meningitis complications of IMD for 2010 and 2011 (see Table 4). The risk of developing long-term sequelae is age and gender-specific.

Long term sequelae

Bacterial meningitis may cause long-term sequelae and permanent disabilities. The risk of developing these complications after meningitis episodes was extracted from Edmond et al. (Edmond, 2010).

Meningitis accounts for various long-term sequelae (each of which is multiplied by the risk of developing meningitis during the acute phase of the disease: 60–63%): cognitive difficulties (0.96–1.01%), seizure disorders (0.3–0.35%), hearing loss (1.56–1.64%), motor deficit (0.6– 0.63%), visual disturbance (0.9–0.95%), behavioural problems (0.36–0.38%), clinical impairments (0.12–0.13%) and multiple impairments (0.78-0.82%) (Edmond, 2010).

Case fatality proportion

The parameters for the case fatality ratio were based on data for EU/EEA countries in 2011, see Table 3 (ECDC, 2013).

Model input summary

Table 1. Transition probabilities used in the outcome tree

Health outcome (Health state)		Distribution of health states in health outcome		Transition probability		Source/assumption

Hearing loss		1.56–1.64%	Edmond, 2010
Cognitive difficulties		0.96–1.01%	Edmond, 2010
Seizure disorder		0.3–0.35%	Edmond, 2010
Motor deficit		0.6–0.63%	Edmond, 2010
Visual disturbance		0.9–0.95%	Edmond, 2010
Behavioural problems		0.36–0.38%	Edmond, 2010
Clinical impairments		0.12–0.13%	Edmond, 2010
Multiple impairments		0.78–0.82%	Edmond, 2010
Fatal cases due to symptomatic infection		See Table 3 (6.9-17.1%)	ECDC, 2013

Table 2. Disability weights and duration

Health outcome (Health state)	Disability Weight (DW) (Haagsma, 2015)		Duration	
	DW	Label	In years	Source
Symptomatic infection	0.655 (0.579-0.727)	Intensive care unit admission	0.019	

				Tunkel, 2004 Assuming the duration of antimicrobial therapy
Permanent disability following meningitis:			Remaining life expectancy	
1. Hearing loss	0.008-0.103	From lowest to highest hearing loss related DWs		
2. Cognitive difficulties	0.044-0.188	From lowest to highest intellectual disability related DWs		
3. Seizure disorder	0.07 (0.057-0.088)	Generic uncomplicated disease: worry and daily medication		
4. Motor deficit	0.011-0.421	From lowest to highest motor impairment related DWs		
5. Visual disturbance	0.004-0.171	From lowest to highest vision impairment related DWs		
6. Behavioural problems	0.088 (0.07-0.108)	Subacute sclerosing panencephalitis – phase 1 (assuming best fitting health state description)		
7. Clinical impairments	0.004-0.421	From lowest to highest DW included in this model		
8. Multiple impairments	0.004-0.421	From lowest to highest DW included in this model		

Table 3. CFP following symptomatic infection

Age	CFR
0	7.8%

1-4	6.9%
5-14	5.6%
15-24	9.5%
25-49	8.9%
50-64	7.6%
≥65	17.1%

Table 4. Age specific distribution per gender of the 60-63% risk of developing meningitis manifestation during the symptomatic infection (TESSy 2010-2011)

Age group	%	
	F	M
0	16.22	16.64
01-04	18.19	23.79
05-09	7.13	8.65
10-14	5.90	4.46
15-19	14.53	15.97
20-24	7.21	8.06
25-29	3.93	3.62
30-34	2.62	3.04

35-39	2.05	1.69
40-44	2.54	1.84
45-49	2.81	2.01
50-54	3.47	2.43
55-59	3.28	2.43
60-64	1.97	1.42
65-69	1.88	1.42
70-74	1.97	0.76
75-79	2.05	1.35
80-84	1.31	0.34
85+	0.93	0.08
Total	100	100

References

Brigham KS, Sandora TJ. Neisseria meningitidis: epidemiology, treatment and prevention in adolescents. Curr Opin Pediatr. 2009 Aug;21(4):437-43.

Buysse CM, Oranje AP, Zuidema E, Hazelzet JA, Hop WC, Diepstraten AF, et al. Long-term skin scarring and orthopaedic sequelae in survivors of meningococcal septic shock. Arch Dis Child. 2009 May;94(5):381-6.

Centers for Disease Control and Prevention. Epidemiology and Prevention of Vaccine-Preventable Diseases. Atkinson W, Wolfe S, Hamborsky J, McIntyre L, eds. 11th ed. Washington DC: Public Health Foundation, 2009.

Edmond K, Clark A, Korczak VS, Sanderson C, Griffiths UK, Rudan I. Global and regional risk of disabling sequelae from bacterial meningitis: a systematic review and meta-analysis. Lancet Infect Dis. 2010 May;10(5):317-28.

Eidelman LA, Putterman D, Putterman C, Sprung CL. The Spectrum of Septic Encephalopathy: Definitions, Etiologies, and Mortalities. JAMA. 1996;275(6):470-473. doi:10.1001/jama.1996.03530300054040.

European Centre for Disease Prevention and Control. Surveillance of invasive bacterial diseases in Europe, 2010. Stockholm: ECDC; 2013.

European Centre for Disease Prevention and Control. Surveillance of invasive bacterial diseases in Europe, 2011. Stockholm: ECDC; 2013.

Haagsma JA, Maertens de Noordhout C, Polinder S, Vos T, Havelaar AH, Cassini A, Devleesschauwer B, Kretzschmar ME, Speybroeck N, Salomon JA. Assessing disability weights based on the responses of 30,660 people from four European countries. Population Health Metrics 2015; 13: 10

Jit M. The risk of sequelae due to pneumococcal meningitis in high-income countries: a systematic review and meta-analysis. J Infect 2010;61(2):114-24.

Tunkel AR, Hartman BJ, Kaplan SL, Kaufman BA, Roos KL, Scheld WM, et al. Practice guidelines for the management of bacterial meningitis. Clin Infect Dis 2004, 39(9):1267–1284.

Ziaja M. Septic encephalopathy. Curr Neurol Neurosci Rep. 2013;13:383.

Invasive pneumococcal disease

Despite the large number of serogroups and serotypes known, most cases of invasive pneumococcal disease (IPD) on a global scale are attributed to the 1, 3, 4, 6, 7, 9, 14, 18, 23 (Jefferson, 2006) and 19a serogroups.

Risk of complications

Invasive pneumococcal infection can manifest as meningitis, bacteraemic pneumonia, bacteraemia without a focus, and bacteraemia with a focus other than the lungs or meninges (e.g. endocarditis, osteomyelitis, and arthritis, although rare). Complications, such as pneumonia or otitis media, are also possible with non-invasive forms of infection but are not considered in this study.

Most observed complications of invasive bacterial diseases, including IPD, are related to the meningitis event. The risk of meningitis was estimated using surveillance data reported to TESSy on clinical presentations of the acute symptomatic disease (ECDC, 2013) and it was found that 10% of IPD cases are reported to manifest meningitis. The risk of developing meningitis during the acute phase of the disease is age- specific. Age and gender-specific data were extracted from ECDC's TESSy database on the risk of developing meningitis for IPD cases from 2010 to 2011 (see Table 4). The risk of developing long-term sequelae is age and gender-specific.

Long-term sequelae

Bacterial meningitis may cause long-term sequelae and permanent disabilities. In order to account for these, information was extracted on the risk of developing permanent sequelae from Edmond et al. (Edmond, 2010).

Meningitis can result in various long-term sequelae: cognitive difficulties (4.2%), seizure disorders (2.5%), hearing loss (7.5%), motor deficit (5.8%), visual disturbance (1.1%), behavioural problems (4.6%) multiple (5.7%) and clinical impairments (3.3%) (Edmond, 2010). Therefore, we assumed that 10% of all IPD patients would be at risk of developing long-term sequelae.

Case fatality proportion

The case fatality proportion for invasive pneumococcal disease has been estimated at 18% in a population-based study of 19 000 people (Harboe, 2009); however, important differences were observed between age groups, with a lower (3%) mortality rate observed in children <5 years. The overall lethality rate due to bacteraemia is about 10–20% (CDC, 2009; Rudan, 2009; Lin, 2010; Saldías, 2009) and may be as high as 60% among elderly patients (CDC, 2009).

Overall mortality due to endocarditis is 50%, but it can reach 60–65% in children (Elward, 1990). The case-fatality proportion for pneumococcal meningitis is about 30%, but may be as high as 80% in elderly persons (CDC, 2009; Burckhardt et al. 2010). The parameters for the case fatality proportion were based on data for EU/EEA countries in 2011, see Table 3 (ECDC, 2013).

Model input summary

Table 1. Transition probabilities used in the outcome tree

Health outcome<br><br>(Health state)	Distribution of health states in health outcome	Transition probability	Source/assumption

Hearing loss		0.75%	Edmond, 2010
Cognitive difficulties		0.42%	Edmond, 2010
Seizure disorder		0.25%	Edmond, 2010
Motor deficit		0.58%	Edmond, 2010
Visual disturbance		0.11%	Edmond, 2010
Behavioural problems		0.46%	Edmond, 2010
Clinical impairments		0.33%	Edmond, 2010
Multiple impairments		0.57%	Edmond, 2010
Fatal cases due to symptomatic infection		See Table 3 (3-24%)	Harboe, 2009

Table 2. Disability weights and duration

Health outcome (Health state)	Disability Weight (DW) (Haagsma, 2015)	Duration

	DW	Label	In years	Source
Symptomatic infection	0.655 (0.579-0.727)	Intensive care unit admission	0.027-0.038	Tunkel, 2004<br>Assuming the duration of antimicrobial therapy
Permanent disability following meningitis			Remaining life expectancy	
1. Hearing loss	0.008-0.103	From lowest to highest hearing loss related DWs		
2. Cognitive difficulties	0.044-0.188	From lowest to highest intellectual disability related DWs		
3. Seizure disorder	0.07 (0.057-0.088)	Generic uncomplicated disease: worry and daily medication		
4. Motor deficit	0.011-0.421	From lowest to highest motor impairment related DWs		
5. Visual disturbance	0.004-0.171	From lowest to highest vision impairment related DWs		
6. Behavioural problems	0.088 (0.07-0.108)	Subacute sclerosing panencephalitis – phase 1 (assuming best fitting health state description)		
7. Clinical impairments	0.004-0.421	From lowest to highest DW included in this model		
8. Multiple impairments	0.004-0.421	From lowest to highest DW included in this model		

Table 3. CFP following symptomatic infection

Age	CFR
0	5.1%
1-4	3%
5-14	7.1%
15-64	8%

≥65	14.3%

Table 4. Age specific distribution per gender of the 60-63% risk of developing meningitis manifestation during the symptomatic infection (TESSy 2010-2011)

Age group	%	
	F	M
0	10.37	11.45
01-04	8.13	8.52
05-09	2.70	3.56
10-14	1.54	2.54
15-19	0.39	1.57
20-24	1.29	1.22
25-29	1.02	2.23
30-34	2.45	3.56
35-39	3.29	5.68
40-44	3.74	5.58
45-49	5.47	6.90
50-54	6.70	7.31
55-59	9.21	7.76
60-64	11.28	9.02

65-69	9.78	7.04
70-74	7.60	6.24
75-79	6.51	4.91
80-84	5.15	2.98
85+	3.36	1.93
Total	100	100

References

Burckhardt I, Burckhardt F, Van Der Linden M, Heeg C, Reinert RR. Risk factor analysis for pneumococcal meningitis in adults with invasive pneumococcal infection. Epidemiol Infect 2010;138(9):1353-8.

Buysse CM, Oranje AP, Zuidema E, Hazelzet JA, Hop WC, Diepstraten AF, et al. Long-term skin scarring and orthopaedic sequelae in survivors of meningococcal septic shock. Arch Dis Child. 2009 May;94(5):381-6.

Centers for Disease Control and Prevention. Epidemiology and Prevention of Vaccine-Preventable Diseases. Atkinson W, Wolfe S, Hamborsky J, McIntyre L, eds. 11th ed. Washington DC: Public Health Foundation, 2009.

Edmond K, Clark A, Korczak VS, Sanderson C, Griffiths UK, Rudan I. Global and regional risk of disabling sequelae from bacterial meningitis: a systematic review and meta-analysis. Lancet Infect Dis. 2010 May;10(5):317-28.

Eidelman LA, Putterman D, Putterman C, Sprung CL. The Spectrum of Septic Encephalopathy: Definitions, Etiologies, and Mortalities. JAMA. 1996;275(6):470-473. doi:10.1001/jama.1996.03530300054040.

Elward K, Hruby N, Christy C. Pneumococcal endocarditis in infants and children: Report of a case and review of the literature. Pediatr Infect Dis J. 1990;9:652.

European Centre for Disease Prevention and Control. Surveillance of invasive bacterial diseases in Europe, 2010. Stockholm: ECDC; 2013.

European Centre for Disease Prevention and Control. Surveillance of invasive bacterial diseases in Europe, 2011. Stockholm: ECDC; 2013.

Haagsma JA, Maertens de Noordhout C, Polinder S, Vos T, Havelaar AH, Cassini A, Devleesschauwer B, Kretzschmar ME, Speybroeck N, Salomon JA. Assessing disability weights based on the responses of 30,660 people from four European countries. Population Health Metrics 2015; 13: 10

Harboe ZB, Thomsen RW, Riis A, et al. Pneumococcal Serotypes and Mortality following Invasive Pneumococcal Disease: A Population-Based Cohort Study. PLoS Med. 2009 May; 6(5): e1000081.

Jefferson T, Ferroni E, Curtale F, et al. Streptococcus pneumoniae in western Europe: serotype distribution and incidence in children less than 2 years old. Lancet Infect Dis 2006;6:405-10.

Jit M. The risk of sequelae due to pneumococcal meningitis in high-income countries: a systematic review and meta-analysis. J Infect 2010;61(2):114-24.

Lin SH, Lai CC, Tan CK, Liao WH, Hsueh PR. Outcomes of hospitalized patients with bacteraemic and non-bacteraemic community-acquired pneumonia caused by Streptococcus pneumoniae. Epidemiol Infect. 2010 Oct 26:1-10. [Epub ahead of print].

Rudan I, Campbell H. The deadly toll of S pneumoniae and H influenzae type b. Lancet 2009; 374(9.693):854-6.

Saldías PF, Viviani GP, Pulgar BD, Valenzuela FF, Paredes ES, Díaz PO. Prognostic factors and mortality in immunocompetent adult patients hospitalized with community-acquired pneumococcal pneumonia. Rev Med Chil 2009; 137(12):1.545-52.

Tunkel AR, Hartman BJ, Kaplan SL, Kaufman BA, Roos KL, Scheld WM, et al: Practice guidelines for the management of bacterial meningitis. Clin Infect Dis 2004, 39(9):1267–1284.

Ziaja M. Septic encephalopathy. Curr Neurol Neurosci Rep. 2013; 13: 383

Legionnaires' disease

Since 2008, the EU case definition focuses solely on Legionnaires' disease, dismissing Pontiac fever cases. Therefore, the present disease outcome tree focuses only on Legionnaires' disease and its sequelae.

Legionnaires' disease is mostly observed in the elderly and conditions associated with immunodeficiency constitute a risk for Legionnaires'.

In rare cases, Legionnaires' disease may also cause extra-pulmonary symptoms, mainly developing cardiac complications (WHO, 2007). Myocarditis, pericarditis, post-cardiomyotomy syndrome or endocarditis are examples of such manifestations although, according to other studies, most of these complication are related to nosocomial infections (Stout, 1997). Extra-pulmonary manifestations are also often observed in immunocompromised patients. For the purpose of this disease model, we focus on community-acquired Legionnaires' cases and extra- pulmonary manifestations are excluded.

Legionnaires' disease causes acute consolidating pneumonia. In most cases, and without testing for the causative agent, pneumonia arising from infection with Legionella pneumophila cannot be distinguished from other types of pneumonia. Symptoms of Legionnaires' disease are an unproductive cough, chest pain, shortness of breath, myalgia and digestive symptoms such as diarrhoea, vomiting and nausea. Patients may also present neurological symptoms such as confusion or deliria (WHO, 2007).

In many cases, the acute phase requires admission to hospital. Studies have shown that in-patient stays in the hospital vary between eight and 13 days (Lettinga, 2002a; von Baum, 2008). However, it may take more than 90 days to recover to the premorbid health state (Lettinga, 2002a) and roentgenographic clearance can take 2–4 months (Edelstein, 2008). For the model the duration of acute Legionnaires' disease is set at 8– 13 days, as stated in one European study (Lettinga, 2002a).

We consider three different health states occurring during the acute phase of the disease, mild (outpatient, uncomplicated cases), moderate (hospitalised, complicated cases not admitted to an intensive-care unit) and severe (complicated cases admitted to an intensive care unit). Studies have shown that hospitalisation is required in 69–74% of Legionnaires' cases (von Baum, 2008; Garcia-Fulgueiras, 2003). We therefore assume that 26–31% of cases will be mild. Moreover, it is shown that 30% of hospitalised cases require a stay in an intensive-care unit (ICU) (Lettinga, 2002b), thus the proportion of complicated cases (not requiring ICU) is set to 46.7–53.2% and those requiring ICU is set to 20.7– 22.2% of all symptomatic infections.

The case-fatality proportion (CFP) differs widely and is associated with the severity level. The CFP for severe cases was found to be higher, ranging from 10 to 30% (Lettinga, 2002b; Benin, 2002; Falco, 1991). In a review conducted by WHO, case-fatality proportions of community- acquired infections ranged from 5 to 10% (WHO, 2007; Benin, 2002; Howden, 2003). The European working group on Legionella infections (EWGLI) suggested a 12% case-fatality in Europe (von Baum, 2008). In our model, CFP for uncomplicated and complicated cases not requiring a stay in an ICU is set at 5–12% and 10–30% for severe cases requiring an ICU.

Risk of complications

Legionnaires' disease is associated with pulmonary (e.g. severe respiratory failure, pulmonary abscess and pleural empyema), cardiac (e.g. acute pericarditis, myocarditis), neuromuscular (e.g. headache, confusion, fatigue) and renal (e.g. acute renal failure, interstitial nephritis) complications. Multi-organ involvement or septic shock are also possible. In the outcome-tree these complications are not treated separately as they are part of the acute phase of Legionnaires' disease.

Studies on the long-term sequelae of Legionnaires' are scarce, however some reported consequences up to two years after the initial infection (Lattimer, 1979). Two studies reported fatigue in 58–81% of cases, concentration problems and memory loss in 6–81%, muscle/joint pain or muscle weakness in 25–79% and post-traumatic stress disorder in 15% (Lattimer, 1979; Lettinga, 2002a). Given the lack of evidence on the causality of Legionnaires' and the long-term consequences, these were not considered.

Model input summary

Table 1. Transition probabilities and distributions used in the outcome tree

Health outcome<br>(Health state)	Distribution of health states in health outcome	Transition probability	Source/assumption
Symptomatic infection<br>(Uncomplicated)<br>(Complicated)<br>(Complicated ICU)	26–31%<br>46.7–53.2%<br>20.7–22.2%		von Baum, 2008; Garcia-Fulgueiras, 2003; Lettinga, 2002b
Fatal cases<br>(Uncomplicated)<br>(Complicated)<br>(Complicated ICU)		5–12%<br>5–12%<br>10–30%	Lettinga, 2002b; Benin, 2002; Falco, 1991; WHO, 2007; Benin, 2002; Howden, 2003; von Baum, 2008

Table 2. Disability weights and duration

Health outcome (Health state)	Disability Weight (DW) (Haagsma, 2015)		Duration	
	DW	Label	In years	Source
Symptomatic infection		Infectious disease, acute episode, moderate	0.022–0.036	Lettinga, 2002a; von Baum, 2008
(Uncomplicated)	0.051 (0.039–0.06)	Infectious disease, acute episode, severe		
(Complicated)	0.125 (0.104–0.152)	Intensive care unit admission		
(Complicated ICU)	0.655 (0.579–0.727)			

References

Edelstein PH. Detection of antibodies to Legionella. In Manual of Molecular and Clinical Laboratory Immunology. Edited by Detrick B, Hamilton R, Folds J. Washington, DC: ASM Press; 2006: 468-476

Haagsma JA, Maertens de Noordhout C, Polinder S, Vos T, Havelaar AH, Cassini A, Devleesschauwer B, Kretzschmar ME, Speybroeck N, Salomon JA. Assessing disability weights based on the responses of 30,660 people from four European countries. Population Health Metrics 2015; 13: 10

World Health Organization: and the prevention of legionellosis. Geneva; 2007.

Stout JE, Yu VL. Legionellosis. N Engl J Med 1997, 337:682-687.

Lettinga KD, Verbon A, Nieuwkerk PT, Jonkers RE, Gersons BP, Prins JM, et al. Health-related quality of life and post-traumatic stress disorder among survivors of an outbreak of Legionnaires disease. Clin Infect Dis 2002, 35:11-17.

von Baum H, Ewig S, Marre R, Suttorp N, Gonschior S, Welte T, et al. Community-acquired Legionella pneumonia: new insights from the German competence network for community acquired pneumonia. Clin Infect Dis 2008, 46:1356-1364.

Edelstein PH: Legionnaires' Disease: History and Clinical findings. In Legionella: Molecular Microbiology. Edited by Heuner K, Swanson M. Portland, Oregan: Caister Academic Press; 2008: 1-17

Garcia-Fulgueiras A, Navarro C, Fenoll D, Garcia J, Gonzalez-Diego P, Jimenez-Bunuales T, et al. Legionnaires' disease outbreak in Murcia, Spain. Emerg Infect Dis 2003, 9:915-921.

Lettinga KD, Verbon A, Weverling GJ, Schellekens JF, Den Boer JW, Yzerman EP, et al. Legionnaires' disease at a Dutch flower show: prognostic factors and impact of therapy. Emerg Infect Dis 2002, 8:1448-1454.

Benin AL, Benson RF, Arnold KE, Fiore AE, Cook PG, Williams LK, et al. An outbreak of travel-associated Legionnaires disease and Pontiac fever: the need for enhanced surveillance of travel-associated legionellosis in the United States. J Infect Dis 2002, 185:237-243.

Howden BP, Stuart RL, Tallis G, Bailey M, Johnson PD. Treatment and outcome of 104 hospitalized patients with Legionnaires' disease. Intern Med J 2003, 33:484-488.

Falco V, Fernandez de Sevilla T, Alegre J, Ferrer A, Martinez Vazquez JM. Legionella pneumophila. A cause of severe community-acquired pneumonia. CHEST Journal 1991, 100:1007-1011.

Lattimer GL, Rhodes LV, 3rd, Salventi JS, Galgon JP, Stonebraker V, Boley S, et al. The Philadelphia epidemic of Legionnaire's disease: clinical, pulmonary, and serologic findings two years later. Ann Intern Med 1979, 90:522-526.

Listeriosis

Acquired listeriosis

Listeriosis is an infection caused by the gram-positive bacterium Listeria monocytogenes. The infection is generally asymptomatic but can become extremely severe in immunocompromised patients, pregnant women and their foetuses/newborn and elderly. The severity of the disease is related to its invasiveness: if the infection is not invasive, it will generally cause mild or no symptoms and therefore no burden (with the exception of acute gastroenteritis if a person ingests a large amount of bacteria). Therefore, it is not surprising that most notified cases are invasive listeriosis diseases, hence complicated ones. In order to estimate the number of complicated cases we referred to the US Centers for Disease Control's 2012 and 2011 Listeriosis Annual Surveillance Summaries (CDC, 2014), reporting 95–97% of cases as invasive, and we applied this to the proportion of complicated symptomatic cases.

Manifestations of listeriosis are meningitis, septicaemia, pneumonia, and gastroenteritis. Based on reports from enhanced surveillance in the Netherlands (Doorduyn, 2006 a,b) and a Gamma distribution used to express the uncertainty, Kemmeren et al. (Kemmeren, 2006) and Haagsma et al. (Haagsma, 2009) estimated the distribution of these health states for acquired listeriosis. However, from a clinical perspective it is conceivable that most cases present a mixed form of the disease and isolates are available from multiple anatomical sites. We therefore defined symptomatic infections as either complicated (invasive) or uncomplicated.

In order to determine those long-term sequelae which are linked only to the manifestation of meningitis, we looked at enhanced surveillance in a few European countries, however data on the risk of developing meningitis during invasive listeriosis disease was inconsistent. Therefore, we referred to CDC enhanced surveillance in the USA from 2007 to 2012 and estimated that 13–18% of invasive (complicated) symptomatic cases would present with meningitis (CDC, 2014).

In the current model, the age-specific case fatality proportion related to listeriosis is derived from cases of acquired listeriosis notified to TESSy from 2009 to 2013 (see Table 3) by all EEA Member States except Bulgaria and Lithuania because they report only aggregate data. The case fatality proportion is applied to complicated cases only.

Perinatal listeriosis

Perinatal listeriosis encompasses both pregnant women and their foetuses or newborns. Of the pregnant women with listeriosis, around two out of three will present with prodromal influenza-like symptoms such as fever, chills and headache. Three to seven days after the prodromal symptoms, the pregnant woman may abort the foetus or have premature labour (Gellin, 1989). To the mother, listeriosis is rarely life- threatening, however, infection in the first trimester of pregnancy may result in spontaneous abortion and, in later stages, in stillbirth or a critically ill newborn (Farber, 1991a). Newborns may present with an early-onset or a late-onset form of listeriosis. Early-onset listeriosis is defined as a case of symptomatic listeriosis in a newborn that is less than seven days old. Early-onset listeriosis is acquired by the foetus prenatally. Newborns with early-onset listeriosis mostly develop sepsis and meningitis (Farber, 1991b; Mylonakis, 2002). Late-onset listeriosis is defined as symptomatic listeriosis in a newborn during the first eight to 28 days of life. In this case, the unborn child is infected during childbirth when passing through the birth canal. Newborns with late-onset listeriosis are usually born healthy and at full term, but are at higher risk of developing meningitis during their first weeks of life (Farber, 1991a).

In the current study, the disease burden for health outcomes of early- and late-onset listeriosis are combined into one category. Based on data reported to TESSy between 2009 and 2013, the case fatality proportion was set to 18.71%.

Risk of complications

Long-term sequelae due to meningitis may occur, and will therefore be considered in the outcome tree. The frequency of other post-infectious complications following listeriosis is low (Haagsma, 2009) and therefore they have been disregarded in the current study.

According to Aouaj et al. (Aouaj, 2002), 20% of all listeriosis cases in their study are perinatal. Therefore, of the 147 cases analysed for long- term outcomes (Aouaj, 2002), we estimated that there were 118 acquired cases (29 perinatal). The study stated that 15 (12.7%) of the total number of acquired listeriosis cases presenting meningitis developed neurological long-term sequelae.

Given that 13–18% of all acute cases present meningitis, the risk of developing neurological long-term sequelae from all cases of complicated acquired listeriosis is 1.65–2.29%.

Similarly, knowing that seven of the 29 perinatal listeriosis cases (24%) developed long-term neurological sequelae and that all acute cases present meningitis, the risk of developing life-long neurological disabilities from a perinatal listeria infection is 24%.

Model input summary

Table 1. Transition probabilities and distributions used in the outcome tree

Health outcome<br><br>(Health state)	Distribution of health states<br><br>in health outcome	Transition probability	Source/assumption
Acquired listeriosis			
Symptomatic infection<br><br>(Uncomplicated)<br><br>(Complicated)	3–5%<br><br>95–97%		CDC, 2014

Fatal cases		Age dependent<br>(Table 3)	TESSy 2009–2013
Permanent disability following meningitis		1.65–2.29% of complicated cases	Aouaj, 2002; CDC 2014
Perinatal listeriosis			
Fatal cases		18.71%	TESSy 2009–2013
Permanent disability due to meningitis		24%	Aouaj, 2002

Table 2. Disability weights and duration

Health outcome (Health state)	Disability Weight (DW) (Haagsma, 2015)		Duration	
	DW	Label	In years	Source
Acquired listeriosis				
Symptomatic infection<br>(Uncomplicated)<br>(Complicated)	0.149 (0.12–0.182)<br>0.655 (0.579–0.727)	Diarrhoea, moderate<br>Intensive care unit admission	0.02–0.5	Kemmeren, 2006<br>Haagsma, 2009;
Permanent disability following meningitis	0.011–0.421	From lowest to highest motor and cognitive difficulties	Remaining life expectancy	
Perinatal listeriosis				
Symptomatic infection	0.655 (0.579–0.727)	Intensive care unit admission	0.02–0.5	Kemmeren 2006 & Haagsma 2009
Permanent disability due to meningitis	0.011–0.421	From lowest to highest motor and cognitive difficulties	Remaining life expectancy	

Table 3. Age-group acquired listeriosis case fatality proportion based on cases and deaths notified to TESSy (2009– 2013)

Age groups	%
0	11.90
1-4	0.00
5-9	5.88
10-14	20.00
15-19	13.16
20-24	1.75
25-29	4.10
30-34	1.39
35-39	8.40
40-44	12.50
45-49	14.08
50-54	16.59
55-59	13.77

60-64	18.16
65-69	15.65
70-74	15.17
75-79	17.83
80-84	17.35
>85	23.15
All ages	15.74

References

Aouaj Y, Spanjaard L, van Leeuwen N, Dankert J. (2002). Listeria monocytogenes meningitis: serotype distribution and patient characteristics in The Netherlands, 1976-95. Epidemiol Infect, 128(3):405-409.

CDC (2014). Centers for Disease Control and Prevention, National Surveillance of Bacterial Foodborne Illnesses (Enteric Diseases) National Listeria Surveillance. Available at: http://www.cdc.gov/nationalsurveillance/listeria-surveillance.html [accessed 2 October 2014].

Doorduyn Y, de Jager CM, van der Zwaluw WK, Wannet WJ, van der Ende A, Spanjaard L, et al. (2006a). Invasive Listeria monocytogenes infections in the Netherlands, 1995-2003. Eur J Clin Microbiol Infect Dis, 25(7):433-442.

Doorduyn Y, de Jager CM, van der Zwaluw WK, Wannet WJ, van der Ende A, Spanjaard L, et al (2006b). First results of the active surveillance of Listeria monocytogenes infections in the Netherlands reveal higher than expected incidence. Euro Surveill, 11(4):E060420 060424.

Doorduyn Y, De Jager CM, Van der Zwaluw WK, Wannet WJB, Heuvelink AE, Van der Ende A, et al. (2007). Intensieve surveillance van Listeria monocytogenes in Nederland, 2006. Infectieziekten Bulletin, 9:308-314.

Farber JM, Peterkin PI (1991a). Listeria monocytogenes, a food-borne pathogen. Microbiol Rev, 55(3):476-511.

Farber JM, Peterkin PI, Carter AO, Varughese PV, Ashton FE, Ewan EP. (1991b). Neonatal listeriosis due to cross-infection confirmed by isoenzyme typing and DNA fingerprinting. J Infect Dis, 163(4):927-928.

Haagsma JA, van der Zanden BP, Tariq L, van Pelt W, van Duynhoven YTHP, Havelaar AH (2009). Disease burden and costs of selected foodborne pathogens in the Netherlands, 2006. Bilthoven: National Institute of Public Health and Environment. RIVM rapport 330331001. Available online at: www.rivm.nl/bibliotheek/rapporten/330331001.pdf

Haagsma JA, Maertens de Noordhout C, Polinder S, Vos T, Havelaar AH, Cassini A, Devleesschauwer B, Kretzschmar ME, Speybroeck N, Salomon JA. Assessing disability weights based on the responses of 30,660 people from four European countries. Population Health Metrics 2015; 13: 10

Kemmeren JM, Mangen MJJ, van Duynhoven YTHP, Havelaar AH. (2006). Priority setting of foodborne pathogens: disease burden and costs of selected enteric pathogens. Bilthoven: National Institute of Public Health and Environment: RIVM rapport 330080001. Available online at: www.rivm.nl/bibliotheek/rapporten/330080001.pdf

Lorber B (1997). Listeriosis. Clin Infect Dis, 24(1):1-9; quiz 10-11.

McLauchlin J, Mitchell RT, Smerdon WJ, Jewell K. (2004). Listeria monocytogenes and listeriosis: a review of hazard characterisation for use in microbiological risk assessment of foods. Int J Food Microbiol, 92(1):15-33.

Mylonakis E, Paliou M, Hohmann EL, Calderwood SB, Wing EJ. (2002). Listeriosis during pregnancy: a case series and review of 222 cases. Medicine (Baltimore), 81(4):260-269.

Measles

According to the US Centers for Disease Control and Prevention (CDC, 2012), approximately 30% of reported symptomatic measles cases have one or more complications. The most important complications are: otitis media (occurring in approximately 10% of infected cases), encephalitis (0.1% of cases), and post-infectious encephalomyelitis (0.1–0.3% of cases). Other complications of acute measles include pneumonia (5–6% of untreated cases; Kabra, 2008; CDC, 1991) and diarrhoea (8%) (CDC, 2012). Convulsions are also a relatively frequent complication (5% of cases; Miller, 1978). Complications during pregnancy occur in up to 30% of women with severe measles (Atmar, 1992).

Complications occurring during the acute phase of the disease may overlap and cannot be treated as independent. Two health states were therefore used in our model: complicated and uncomplicated. We derived the risk of complications from data reported to TESSy between 2006 and 2013. Given the high number of cases notified to TESSy without information on complications and in order to account for this uncertainty we included two scenarios. We estimated the proportion of cases reported as uncomplicated out of the number of known cases as 57.24% (excluding cases for which complications were reported as unknown or left blank). We then added the uncomplicated cases to the unknown and blank and obtained the total number 88.64% (assuming that all unknown and blank cases were uncomplicated).

In the model, the rare permanent disabilities due to otitis media, encephalitis, post-infectious encephalomyelitis and subacute sclerosing panencephalitis (SSPE) (van Steenbergen, 2006) are treated as distinct sequelae.

Otitis media and permanent disability due to otitis media

The health state otitis media occurs in around one in ten cases of acute measles and can result in permanent hearing loss (CDC, 2011). The probability of developing permanent disability due to otitis media is 0.01% (CDC, 1991) of all cases of otitis media, therefore the overall risk of developing a permanent disability has been set to 0.001%.

Encephalitis and permanent disability due to encephalitis

Encephalitis occurs in approximately 0.1% of acute symptomatic cases (Weissbrich, 2003; Beutels, 2002; Miller, 1957). Long-term sequelae of measles encephalitis are reported to occur in 20–30% of measles-related encephalitis cases (Beutels, 2002; Filia, 2007); therefore the transition probability for the health outcome 'permanent disability due to encephalitis' was set to 0.02-0.033%.

Encephalitis of the delayed type (Barthez Carpentier, 1992) can occur after acute illness in immunocompromised patients and may occur after asymptomatic infection (Kidd, 2003). Because of the specific population affected, and its relative rarity, the outcome tree was not modified accordingly.

Post-infectious encephalomyelitis (PIE) and permanent disability due to PIE

Post-infectious encephalomyelitis occurs in 1–3 per 1 000 infected persons, usually three to ten days after the onset of rash. Higher rates of PIE due to measles occur in adolescents and adults than in school-aged children (Perry & Halsey, 2004). The condition is associated with demyelination and is thought to have an autoimmune basis. A total of 33% of those afflicted with PIE who survive have lifelong neurological sequelae, including severe retardation, motor impairment, blindness and sometimes hemiparesis (Perry & Halsey, 2004). The transition probability in the model for developing the health outcome 'permanent disability due to PIE' was set to the range 0.033–0.1%.

Subacute sclerosing panencephalitis (SSPE)

On average, the symptoms of SSPE begin seven to ten years after measles infection, but they can appear anytime from one month to 27 years after infection (CDC, 2012).

Various estimates are available for the proportion of cases that develop the SSPE health outcome. SSPE is observed at a rate of 1 per 10 000– 20 000 (Weissbrich, 2003; Takasu, 2003; Bellini, 2005; Garg, 2008). In children who have previously had natural measles, the risk of developing SSPE is between 0.6 and 2.2 per 100 000 cases (Hosoya, 2006). Other estimates include: one SSPE case in every 100 000 cases of measles (Rezende, 1989); 4–11 cases of SSPE per 100 000 cases of measles (CDC, 2009); one in every 25 000 measles infections (Miller, 2004); one in 8 000 for children under two years (Miller, 1992; 2004) and a 16-fold greater risk for those infected under one year of age compared with those over five years (Miller, 1992). The risk of developing SSPE is known to be age-specific (Beutels, 2002; Farrington, 1991; Miller, 2004; CDC, 2012).Therefore, transitional probabilities in the model were also specified as age-dependent (see Table 3) (Beutels, 2002). In the model, the duration for this health outcome was specified as one to two years (CDC, 2012). In the model the transition probability from SSPE to death was set to 100%.

Case fatality proportion

Measles is fatal in approximately 0.05–0.1% of cases (Wolfson, 2007; Lozano, 2012). The risk of death is higher among young children and adults (CDC, 2012). According to CDC (CDC, 2012), the most common causes of death are pneumonia in children and acute encephalitis in adults, but due to the lack of specific data for different age groups we applied the same CFP for all the same age groups.

Model input summary

Table 1. Transition probabilities used in the outcome tree

Health outcome (Health state)	Distribution of health states in health outcome	Transition probability	Source/assumption

Symptomatic infection<br><br>(Complicated)<br>(Uncomplicated)	 <br>11.36-42.76%<br>57.24-88.64%		TESSy, 2006–2013
Permanent disability following otitis media		0.001%	CDC, 1991
Permanent disability following encephalitis		0.02–0.033%	Beutels, 2002; Filia, 2007
Permanent disability following PIE		0.033–0.1%	Perry & Halsey, 2004
SSPE		See Table 3	Beutels, 2002
Fatal cases following SSPE		100%	
Fatal cases following symptomatic infection		0.05–0.1%	Wolfson, 2007; Lozano, 2012

Table 2. Disability weights and duration

Health outcome<br>(Health state)	Disability Weight (DW) (Haagsma, 2015)		Duration	
	DW	Label	In years	Source
Symptomatic infection<br>(Complicated)<br>(Uncomplicated)	 <br>0.125 (0.104-0.152)<br>0.051 (0.039-0.06)	 <br>Infectious disease, acute episode, severe<br>Infectious disease, acute episode, moderate	0.03	Kwong, 2012
Permanent disability due to otitis media	0.008-0.103	From lowest to highest hearing loss related DWs	Remaining life expectancy	
Permanent disability due to encephalitis	0.054-0.425	From lowest to highest Motor plus cognitive impairments related DWs	Remaining life	

			expectancy	
Permanent disability due to PIE	0.054-0.425	From lowest to highest Motor plus cognitive impairments related DWs	Remaining life expectancy	
Latency period before SSPE	0		0.082–27	CDC, 2012
SSPE	0.276 (0.088-0.543)	From Phase 1 to Phase 3 (median is Phase 2) of subacute sclerosing panencephalitis related DWs	1–2	CDC, 2012

Table 3. Transition probabilities subacute sclerosing panencephalitis (SSPE)

Age	%
0-4	0.0081
5-9	0.0011
≥10	0.0010

References

Barthez Carpentier MA, Billard C, Maheut J, Jourdan ML, Degenne D, Ruchoux MM, et al. Acute measles encephalitis of the delayed type: neuroradiological and immunological findings. Eur Neurol. 1992;32(4):235-7.

Bellini WJ, Rota JS, Lowe LE, et al (2005). Subacute sclerosing panencephalitis: More cases of this fatal disease are prevented by measles immunization than previously recognized. J Infect Dis 192:1686–1693.

Beutels P, Van Damme P, Van Casteren V, Gay NJ, De Schrijver K, Meheus A. The difficult quest for data on "vanishing" vaccine-preventable infections in Europe: the case of measles in Flanders (Belgium). Vaccine. 2002 Oct 4;20(29-30):3551-9.

Centers for Disease Control (CDC). Measles: United States, 1990. MMWR Morb Mortal Wkly Rep 1991;40:369–372.

Centers for Disease Control and Prevention. Epidemiology and Prevention of Vaccine-Preventable Diseases. Atkinson W, Wolfe S, Hamborsky J, McIntyre L, eds. 11th ed. Washington DC: Public Health Foundation, 2009

Centers for Disease Control and Prevention (CDC). The Pink Book: Course Textbook - 12th Edition Second Printing (May 2012).

Farrington CP. Subacute sclerosing panencephalitis in England and Wales: transient effects and risk estimates. Stat Med 1991;10:1733–1744;

Filia A, Brenna A, Panà A, Maggio Cavallaro G, Massari M, Ciofi degli Atti LM. Health burden and economic impact of measles-related hospitalizations in Italy in 2002–2003. BMC Public Health. 2007; 7: 169

Garg RK (2008). Subacute sclerosing panencephalitis. J Neurol 255(12):1861–1871.

Haagsma JA, Maertens de Noordhout C, Polinder S, Vos T, Havelaar AH, Cassini A, Devleesschauwer B, Kretzschmar ME, Speybroeck N, Salomon JA. Assessing disability weights based on the responses of 30,660 people from four European countries. Population Health Metrics 2015; 13: 10

Hosoya M. Measles encephalitis: direct viral invasion or autoimmune-mediated inflammation? Intern Med. 2006;45(14):841-2.

Kabra SK, Lodha R, Hilton DJ. Antibiotics for preventing complications in children with measles. Cochrane Database of Systematic Reviews 2008, Issue 3. Art. No.: CD001477. DOI: 10.1002/14651858.CD001477.pub3.

Kwong JC, Ratnasingham S, Campitelli MA, Daneman N, Deeks SL, et al. (2012) The Impact of Infection on Population Health: Results of the Ontario Burden of Infectious Diseases Study. PLoS ONE 7(9): e44103. doi:10.1371/journal.pone.0044103

Lozano R, Naghavi M, Foreman K, Lim S, Shibuya K, Aboyans V, et al. (Dec 15, 2012). Global and regional mortality from 235 causes of death for 20 age groups in 1990 and 2010: a systematic analysis for the Global Burden of Disease Study 2010. Lancet 380 (9859): 2095–128

Miller HG, Stanton JB, Gibbons JL. Acute disseminated encephalomyelitis and related syndromes. British medical journal.1957 Mar 23;1(5020):668-72

Miller CL. Severity of notified measles. British Medical Journal. 1978; 1(6122): 1253-1255.

Miller C, Andrews N, Rush M, Munro H, Jin L, Miller E. The epidemiology of subacute sclerosing panencephalitis in England and Wales 1990-2002. Arch Dis Child. 2004 Dec;89(12):1145-8.

Murray CJL, Lopez AD, editors. The global burden of disease: a comprehensive assessment of mortality and disability from diseases, injuries, and risk factors in 1990 and projected to 2020. Cambridge: Harvard University Press, 1996.

Rezende MA. Nursing care and the neurological sequelae of measles. Revista da Escola de Enfermagem USP. 1989 Apr;23(2):141-8.

Stein CE, et al, The Global Burden of Measles in the Year 2000—A Model that Uses Country-Specific Indicators. Journal of Infectious Diseases 2003; 187(Suppl 1):S8–14.

Takasu T, Mgone JM, Mgone CS, et al (2003). A continuing high incidence of subacute sclerosing panencephalitis (SSPE) in the eastern highlands of Papua New Guinea. Epidemiol Infect 131:887–898.

Tishler M, Abramov AL. Liver involvement in measles infection of young adults. Isr J Med Sci. 1983 Sep;19(9):791-3.

Wolfson LJ, Strebel PM, Gacic-Dobo M, Hoekstra EJ, McFarland JW, et al. (2007) Has the 2005 measles mortality reduction goal been achieved? A natural history modelling study. Lancet 369: 191–200

Zhou F, Reef S, Massoudi M, Papania MJ, Yusuf HR, Bardenheier B, et al. An Economic Analysis of the Current Universal 2-Dose Measles-Mumps- Rubella Vaccination Program in the United States. The Journal of Infectious Diseases. 2004 May 1;189 Suppl 1:S131-45.

Mumps

Mumps is symptomatic in 80% of infections (CDC, 2012), the main symptom being parotitis.

Risk of complications

The principal complications with mumps are orchitis, oorphoritis, meningitis, pancreatitis, and encephalitis.

Epididymo-orchitis occurs in 15–30% of adult men with mumps infection, but it is rare before puberty (Hviid, 2008). Oophoritis (ovarian inflammation), the counterpart of orchitis in females, is associated with pelvic pain and tenderness. It occurs in 5% of post-pubertal females (CDC, 2009).

Mumps meningitis is a benign entity with no significant risk of mortality or long-term sequelae. Even though cerebrospinal fluid pleiocytosis occurs in about half of the patients with mumps, clinical manifestations of meningitis arise in 1–10% of the cases (Hviid, 2008), and long-term morbidity is rare. Encephalitis occurs in 0.1% of acute cases (Hviid, 2008).

Acute pancreatitis, with symptoms of abdominal distention and pain, fever, nausea, and vomiting (Demirci, 2011), occurs in approximately 4% of mumps cases (Vanlioglu & Chua, 2011).

With mumps, the acute complications of symptomatic infections are considered as a single health state (complicated) because they can occur concomitantly.

Of all mumps infections, 40–50% may have only non-specific or primarily respiratory symptoms (CDC, 2012). Therefore, knowing that 20% of infections are asymptomatic, 32–40% of symptomatic cases were considered to be uncomplicated. Durations were set to 7–10 days for the uncomplicated cases and 7–14 days for the complicated ones.

Permanent deafness caused by mumps occurs with an estimated frequency of one in 20 000 cases (0.005%) and in 80% of the cases, hearing loss is monolateral (Hviid, 2008).

Case fatality proportion

Death is very rare in mumps cases and the mortality rate following encephalitis is 1.5%. Therefore, 0.15% was used in the model for the risk of death resulting from all symptomatic infections. More than half of fatalities occur in patients over 19 years (Hviid, 2008; Demirci, 2011). This age distribution also applies to the symptomatic complicated cases (see Table 3).

Model input summary

Table 1. Transition probabilities used in the outcome tree

Health outcome<br><br>(Health state)		Distribution of health states<br>in health outcome	Transition probability	Source/assumption

Symptomatic infection<br>(Uncomplicated)<br>(Complicated)	32–40%<br>60–68%		CDC, 2012
Permanent disability due to hearing loss		0.005%	Hviid, 2008
Fatal cases		0.15%<br>Age dependent<br>(see Table 3)	Hviid, 2008<br>Assuming 1.5% of encephalitis cases (0.1%) become fatal

Table 2. Disability weights and duration

Health outcome (Health state)	Disability Weight (DW) (Haagsma, 2015)		Duration	
	DW	Label	In years	Source
Symptomatic infection<br>Uncomplicated<br>Complicated	0.051 (0.039-0.06)<br>0.125 (0.104-0.152)	Infectious disease, acute episode, moderate<br>Infectious disease, acute episode, severe	0.019-0.027<br>0.019-0.038	Hviid, 2008
Permanent hearing loss	0.008 (0.005-0.012)	Unilateral hearing loss	Remaining life expectancy	Hviid, 2008

Table 3. Age distribution – case fatality ratio

0-19	50
≥20	50

References

Centers for Disease Control and Prevention. Epidemiology and Prevention of Vaccine-Preventable Diseases. Atkinson W, Wolfe S, Hamborsky J, eds. 12th ed., second printing. Washington DC: Public Health Foundation, 2012.

Demirci CS, Abuhammour W, Quintana EC, Shahidi H, Wilkes G. Mumps. Available from http://emedicine.medscape.com/article/966678- overview [accessed 12 September, 2011].

Haagsma JA, Maertens de Noordhout C, Polinder S, Vos T, Havelaar AH, Cassini A, Devleesschauwer B, Kretzschmar ME, Speybroeck N, Salomon JA. Assessing disability weights based on the responses of 30,660 people from four European countries. Population Health Metrics 2015; 13: 10

Hviid A, Rubin S, Mühlemann K. Mumps. Lancet. 2008 Mar 15;371(9616):932-44.

Mandell GL, Bennet JE, Dolin R. Mandell, Douglas and Bennett’s Principles and Practice of Infectious Disease 5th edition. Churchill Livingstone, 1999.

Vanlioglu B, Chua TC. Presentation of mumps infection as acute pancreatitis without parotitis. Pancreas. 2011 Jan;40(1):167-8.

Pertussis

Pertussis is principally toxin-mediated. Toxins paralyse the cilia of the respiratory tract cells, leading to the clinical features and complications of the disease. The clinical course of the illness is divided into three stages. The first one is the catarrhal stage, characterised by coryza, sneezing, low-grade fever and a mild, occasional cough. The cough gradually becomes more severe, and after 1–2 weeks, the paroxysmal stage begins, usually lasting one to six weeks. In the convalescent stage, which lasts two to three weeks, recovery is gradual and the cough becomes less paroxysmal. However, paroxysms often recur for many months after the onset of pertussis (CDC, 2009; Mandell, 1999).

Clinical manifestations of pertussis may be mild in adults and vaccinated children. Around 20% of infected persons develop mild/asymptomatic disease (Rothstein, 2005). Based on this finding, an asymptomatic proportion of 20% was specified in the model.

Risk of complications

The principal complications of pertussis are secondary infections, such as otitis media and pneumonia, neurological complications, such as seizures and encephalopathy. Other possible complications include physical sequelae of paroxysmal cough (e.g. subconjunctival haemorrhages, epistaxis, petechiae, central nervous system haemorrhage, pneumothorax and hernia) (CDC, 2009; Mandell, 1999).

Pneumonia can result from aspiration during whooping and vomiting or from impaired clearance mechanisms. It occurs in 5.2% of all patients (CDC, 2009), in up to 25% of cases reported in infants (Mandell, 1999), in 2–4% of individuals aged 10–19 years, in 2.7–5.5% of those over 20 years and in 5–9% of those over 30 years (Rothstein, 2005).

Approximately 4% of adolescents and adults with symptomatic pertussis infection develop otitis media (De Serres, 2000).

Neurological complications of pertussis are more common among infants. In children 12 months of age or younger with pertussis in the USA (1980–1989), convulsions occurred in 3.0% and encephalopathy in 0.9% of cases. Encephalopathy, febrile and afebrile convulsions occur infrequently in adults with pertussis (CDC, 2009), with encephalopathy observed in 0.1% of cases during the period 1997–2000 (CDC, 2009).

Seizures were reported among 0.8% of all pertussis cases in the period 1997–2000 (CDC, 2009).

Infants with pertussis are at greater risk of complications and permanent sequelae, however complications of pertussis, including serious ones, are not uncommon in adolescents and adults, especially the elderly. Complications occur in up to 23% of patients aged 19–83 years. Complications are more frequent in adults than in adolescents (28% compared to 16%) (CDC, 2009; Mandell, 1999; Rothstein, 2005).

Most complications occurring during the symptomatic acute disease phase overlap with one other. We therefore decided to aggregate all complicated cases into one health state. Risk of complications is reported to be 50% in infants (<1 year), 16% in children and adolescents and 28% in cases 20 years (CDC, 2013).

We assumed that in complete and active surveillance systems, those cases notified represent the complicated cases of pertussis. The United Kingdom has an enhanced surveillance system for pertussis where information is compiled from different sources. We therefore chose to consider the number of cases reported in the UK (2007–2013) as complicated. In order to estimate the proportion of complicated cases, we divided the number of cases reported in the UK by the estimated true incidence of pertussis derived from the literature: 71–507 per 100 000 10 years; 46 per 100 000 <10 years (Wirsing von Konig, 2002; Diez-Domingo, 2004) (see Table 3).

Case fatality proportion

Death from pertussis is rare beyond the age of 10 years, occurring in less than 0.1% of all cases, with older adults being at greater risk than younger adults (Rothstein, 2005). Pneumonia is a leading cause of death, but in a study of 99 patients aged 55–94 years who died of pertussis (Rothstein, 2005), intracranial haemorrhage was the cause of death for two of the four deaths thought to be associated with pertussis. Among patients who died, apnoea, pneumonia, seizures, and encephalopathy were reported for 58% (40 of 69), 54% (39 of 72), 21% (14 of 68), and 12% (7 of 57), respectively (Rothstein, 2005; Farizo, 1992).

‘The case fatality proportion in the United States between 1990 and 1996 was 0.2%. Eighty-four per cent of pertussis-related deaths occur in infants younger than six months of age’ (Ratnapalam, 2005).

In general, we considered that only complicated cases were at risk of dying. We used the CFP reported in the UK for deaths of infants <1 year old because of its comprehensive surveillance system, compiling data from different sources and deemed to be capturing approximately 94% of the cases in recent capture-recapture studies. There were 33 deaths due to pertussis reported to TESSy between 2007 and 2013 out of 1 791 cases. This resulted in a CFP of 1.84% which was applied to complicated cases <1 year.

We chose 0.1% of complicated cases for all other age groups.

Model input summary

Table 1. Transition probabilities used in the outcome tree

Health outcome<br><br>(Health state)	Distribution of health states in health outcome	Transition probability	Source/assumption
Symptomatic infection<br><br>(Complicated)<br><br>(Uncomplicated)	Age dependent (see Table 3)<br><br>Remaining cases		CDC, 2013

Fatal cases		1.84% <1 yr.<br>0.1% ≥ 1 yr.	TESSy<br>Rothstein, 2005

Table 2. Disability weights and duration

Health outcome (Health state)	Disability Weight (DW) (Haagsma, 2015)		Duration	
	DW	Label	In years	Source
Symptomatic infection			0.077–0.211	CDC, 2009; Mandell, 1999
(Complicated)	0.125 (0.104–0.152)	Infectious disease, acute episode, severe		
(Uncomplicated)	0.051 (0.039–0.06)	Infectious disease, acute episode, moderate		

Table 3. Risk of complications

Age	%	
	Estimated from low true incidence	Estimated from high true incidence
0	28.04	
01-04	8.04	
05-09	5.85	
10-14	0.35	2.46
15-19	0.39	2.81
20-24	1.05	7.50
25-29	1.59	11.38
30-34	1.92	13.68
35-39	1.45	10.32
40-44	1.84	13.12
45-49	2.23	15.96
50-54	2.00	14.29
55-59	1.68	11.97

60-64	1.20	8.57
65-69	1.48	10.58
70-74	1.24	8.83
75-79	1.30	9.26
80-84	0.91	6.52
85+	0.54	3.88

References

Centers for Disease Control and Prevention. Epidemiology and Prevention of Vaccine-Preventable Diseases. Atkinson W, Wolfe S, Hamborsky J, McIntyre L, eds. 11th Edition. Washington DC: Public Health Foundation, 2009.

Centers for Disease Control and Prevention. http://www.cdc.gov/pertussis/about/complications.html Last update January 2013 [accessed 25 September 2014].

De Serres G, Shadmani R, Duval B, Boulianne N, Déry P, Douville Fradet M, et al. Morbidity of pertussis in adolescents and adults. J Infect Dis. 2000 Jul;182(1):174-9.

Diez-Domingo J, Ballester A, Baldo JM, Planelles MV, Villarroya JV, Alvarez T et al. Incidence of pertussis in persons < 15 years of age in Valencia, Spain: seroprevalence of antibodies to pertussis toxin (PT) in children, adolescents and adults. Journal of Infection (2004) 49, 242- 247

Farizo KM, Cochi SL, Zell ER, Brink EW, Wassilak SG, Patriarca PA. Epidemiological features of pertussis in the United States, 1980-1989. Clin Infect Dis. 1992 Mar;14(3):708-19.

Haagsma JA, Maertens de Noordhout C, Polinder S, Vos T, Havelaar AH, Cassini A, Devleesschauwer B, Kretzschmar ME, Speybroeck N, Salomon JA. Assessing disability weights based on the responses of 30,660 people from four European countries. Population Health Metrics 2015; 13: 10

Mandell GL, Bennet JE, Dolin R. Mandell, Douglas and Bennett's Principles and Practice of Infectious Disease 5th edition. Churchill Livingstone, 1999.

Ratnapalan S, Parkin PC, Allen U. Case 1: The deadly danger of pertussis. Paediatr Child Health. 2005 Apr;10(4):221-2

Rothstein E, Edwards K. Health burden of pertussis in adolescents and adults. Pediatr Infect Dis J. 2005 May;24(5 Suppl):S44-7. Wirsing von Konig CH, Halperin S, Riffelmann M, Guiso N. Pertussis of adults and infants. Lancet Infect Dis 2002; 2: 744-50.

Poliomyelitis

Poliomyelitis is an acute illness which may result from invasion of the gastro-intestinal tract by one of three types of polio virus. It usually affects small children under the age of three years. The virus is transmitted through contaminated food and water, and multiplies in the intestine, from where it can invade the nervous system. Transmission occurs through contact with faeces or pharyngeal secretions of an infected person. The incubation period ranges from three to 21 days, but may be longer. Cases are infectious from about ten days before to seven days after the onset of symptoms; however, carriers and some immuno-compromised persons may shed the virus in faeces for longer than six weeks (Howard, 2005).

Most infections are not clinically apparent; up to 95% of infections are asymptomatic (CDC, 2009).

Risk of complications

Clinical disease may range in severity from minor illness (abortive poliomyelitis), to non-paralytic poliomyelitis (aseptic meningitis) and paralytic poliomyelitis (Feigin, 2009).

Approximately 4–8% of polio infections consist of a non-specific 'minor illness' without clinical or laboratory evidence of central nervous system invasion (CDC, 2009; Feigin, 2009). This clinical presentation is known as abortive poliomyelitis, and is characterised by complete recovery in less than one week (CDC, 2009).

Nonparalytic aseptic meningitis (symptoms of stiffness of the neck, back, and/or legs) which usually follows several days after a prodrome similar to that of a minor illness, occurs in 1–2% of polio infections (CDC, 2009). Increased or abnormal sensations can also occur. Typically these symptoms will last from two to ten days, followed by complete recovery (CDC, 2009).

Less than 1% of all polio infections result in flaccid paralysis (CDC, 2009; Heymann, 2004). Paralytic symptoms generally begin one to ten days after prodromal symptoms and progress for two to three days. Generally, no further paralysis occurs after fever subsides (CDC, 2009). Many patients with paralytic poliomyelitis recover completely and, in most of them, muscle function returns to some degree. Weakness or paralysis 12 months after onset is usually permanent (CDC, 2009).

In acute flaccid paralysis (AFP), the legs are usually more often affected than the muscles of the upper body. However, the polio virus may invade the brain stem, potentially leading to breathing difficulty and even death. Symptoms include headache, gastro-intestinal disturbance, malaise and stiffness of the neck and back, with or without paralysis (American Academy of Pediatrics, 2006; Shibuya & Murray, 2002). Improvements are seen within the first six months (Farbu, 2013; Neumann, 2004). The principal complication is painful, acute, asymmetric paralysis of the arms or the legs, reaching its maximum extent over the course of three to four days and leading to permanent lameness of the affected limbs and breathing difficulties (UK Department of Health, 2006; WHO, 2014).

Given the estimates of symptomatic polio cases, we considered that on average 8.5% of infections are symptomatic (6–11%; CDC, 2011); hence 70.59% of cases on average will be abortive (uncomplicated), 17.65% will be non-paralytic and 11.76 will be paralytic.

According to WHO (WHO, 2014), 1 in 200 infections leads to irreversible paralysis. Given that 1% of all infections has a paralytic form, we considered that 50% of all paralytic forms would develop a permanent disability due to paralysis.

Post-polio syndrome is a long-term sequela that occurs 30–35 years after infection in approximately 25–50% of cases (Jubelt & Drucker, 1999). A slowly progressing condition, it can also occur in patients who have had the non-paralytic form of poliomyelitis. The most common symptoms include slow, progressive muscle weakness, fatigue (both generalised and muscular) and a gradual decrease in the size of muscles (muscle atrophy). Pain from joint degeneration and increasing skeletal deformities such as scoliosis (curvature of the spine) is common and may precede the weakness and muscle atrophy. Some individuals experience only minor symptoms while others develop visible muscle weakness and atrophy. Fatigue is clearly the most prominent manifestation, occurring in up to 80% of patients (Jubelt & Drucker, 1999). Post-polio syndrome is rarely life-threatening (NINDS, 2012).

Case fatality proportion

The case fatality proportion is 5–10% of paralytic forms (WHO, 2014).

Model input summary

Table 1. Transition probabilities used in the outcome tree

Health outcome (Health state)	Distribution of health states in health outcome	Transition probability	Source/assumption
Symptomatic infection		6-11%	
(Uncomplicated) (Non-paralytic poliomyelitis)	70.59%		CDC, 2009
(Paralytic poliomyelitis)	17.65%		CDC, 2009; Heymann, 2004
	11.76%		
Post-polio syndrome		25–50%	Jubelt & Drucker, 1999
Permanent disability following paralytic poliomyelitis		50%	WHO, 2014

Fatal cases following paralytic poliomyelitis		5-10%	WHO, 2014

Table 2. Disability weights and duration

Health outcome (Health state)	Disability Weight (DW) (Haagsma, 2015)		Duration	
	DW	Label	In years	Source
Symptomatic infection<br>(Uncomplicated)<br>(Non-paralytic poliomyelitis)<br>(Paralytic poliomyelitis)	0.007 (0.005–0.01)<br>0.051 (0.039–0.06)<br>0.125 (0.104–0.152)	Infectious disease, acute episode, mild<br>Infectious disease, acute episode, moderate<br>Infectious disease, acute episode, severe	0.019<br>0.005–0.027<br>0.011–0.038	CDC, 2009<br>CDC, 2009<br>CDC, 2009
Permanent disability following paralytic poliomyelitis	0.067 (0.054–0.081)	Spinal cord lesion below neck level (treated)	Remaining life expectancy	
Latency period before PPS	0		30–35	Jubelt & Drucker, 1999
Post-polio syndrome (PPS)	0.344 (0.3–0.391)	Musculoskeletal problems, generalised, moderate	Remaining life expectancy	

References

American Academy of Pediatrics. Red Book: 2006 Report of the Committee on Infectious Diseases. 27th ed. Elk Grove Village. 2006.

Centers for Disease Control and Prevention (CDC). Epidemiology and Prevention of Vaccine-Preventable Diseases. Atkinson W, Wolfe S, Hamborsky J, McIntyre L, eds. 11th ed. Washington DC: Public Health Foundation, 2009.

Department of Health, UK. Immunisation against infectious disease (the Green Book). 3rd ed. London: The Stationery Office. 2006.

Feigin R, Cherry J, Demmler-Harrison GJ, Kaplan S. Feigin and Cherry's Textbook of Pediatric Infectious Diseases. Sixth Edition, 2009.

Farbu E. 2013. Post-polio Syndrome. In: JH Stone, M Blouin, editors. International Encyclopedia of Rehabilitation. Available online: http://cirrie.buffalo.edu/encyclopedia/en/article/138/

Haagsma JA, Maertens de Noordhout C, Polinder S, Vos T, Havelaar AH, Cassini A, Devleesschauwer B, Kretzschmar ME, Speybroeck N, Salomon JA. Assessing disability weights based on the responses of 30,660 people from four European countries. Population Health Metrics 2015; 13: 10

Heymann D, Aylward B. Poliomyelitis. Orphanet Encyclopedia. WHO. 2004.

Howard RS. Poliomyelitis and the post polio syndrome. BMJ 2005;330(7503):1314–1318.

Jubelt B, Drucker J. Poliomyelitis and the Post-Polio Syndrome in Motor Disorders. Philadelphia: Lippincott Williams and Wilkins. 1999. pp. 381.

Neumann D. Polio: its impact on the people of the United States and the emerging profession of physical therapy (PDF).The Journal of Orthopaedic and Sports Physical Therapy 2004; 34 (8): 479–92. PMID 15373011

Post-Polio Syndrome Fact Sheet. NINDS. 2012.

Shibuya K, Murray CJL. Poliomyelitis. In: Stein CE, Murray CJL, Lopez AD (eds). The Global Burden of Disease and Injuries Series, Volume 4: The Global Epidemiology of Infectious Diseases. WHO, 2002.

UNICEF Executive Board Special (2009). Focus Session on Global Health, with particular focus on polio eradication. Available online from: http://www.unicef.org/about/execboard/files/Special_focus_session-Background_SFS_English_8_June_09(1).pdf

World Health Organization (WHO). Poliomyelitis. Available from http://www.who.int/topics/poliomyelitis/en/ [accessed 19 September 2014].

Q fever

Q fever infection becomes symptomatic in 40% of cases (Dijkstra, 2012). Symptomatic infections are divided into two health states: uncomplicated and complicated (more severe cases) and the proportion of complications is based on the hospitalisation rate (2–5%) for Q fever (Maurin & Raoult, 1999; Raoult, 2005).

Around 1–2% of Q fever cases are fatal (ECDC, 2010). This CFR is applied to complicated cases only, based on the US Centers for Disease Control (CDC) Fact Sheet which states that 'the case fatality ratio for hospitalized patients is under 2%' (CDC, 2013).

Chronic Q fever

The transition probability that cases with symptomatic infections will develop chronic Q fever is set to 1.6% (1.5–2%) (van der Hoek, 2011; ECDC, 2010). Due to the lack of evidence, development of chronic Q fever was not associated with asymptomatic Q fever (ECDC, 2010). The average duration of chronic Q fever before developing symptoms is 0.5 years (0.08–1.5 years) (Fenollar, 2001) and this is included in the burden calculation as it reduces the life expectancy of later health outcomes.

Taking the duration of treatment as a proxy for the duration of chronic Q fever, we set the duration to 12–18 months (CDC, 2013) although there are studies recommending life-long treatment which could vary from one year to a person's entire lifespan (Forland, 2012). However, we assume that symptoms due to the infection resolve during the treatment; if symptoms continue, we consider them not to be associated with the Q fever infection but with underlying conditions.

The most common manifestation of chronic Q fever is heart failure, of which a quarter of cases show conduction disorders (Marrie, 2010); other possible manifestations include vascular and pulmonary infections and chronic hepatitis (Maurin & Raoult, 1999). Therefore disability weights describing heart failure were applied.

The case fatality proportion for chronic Q fever has been estimated to be from to 5 to 50%, according to time of diagnosis and onset of treatment (ECDC, 2010).

Post-infectious fatigue syndrome

Follow-up studies after large outbreaks provide some information regarding duration and the probability of developing post-infectious fatigue syndrome. One large cohort following an outbreak in the UK used standard clinical criteria to quantify the proportion of patients developing fatigue after five years (Ayres JG, 1998) and ten years (Wildman, 2002). The first follow-up reported a larger proportion of idiopathic chronic fatigue (ICF) in Q fever cases (42.3%) than in matched controls (26%), with a difference of 16.3%. At the 10-year follow-up point, cases were matched to controls for the presence of comorbidities and hospital attendance, but there was still a higher proportion of ICF (21.6% vs. 5.4%), with a difference of 16.2%. A recent study from a Dutch outbreak indicates the proportion of patients with fatigue after 12 to 26 months to be higher (43.5%) than after five or ten years of follow-up (Morroy, 2011). Therefore, two health states were specified in order to differentiate short-term fatigue (43.5–16.2/16.3% = 27.2/27.3%) from long-term fatigue (16.2–16.3%). The short-term health state consists of clinical cases that recover within 12 to 26 months; severe cases are assumed to recover after 10 years. Regarding the sources of post-infectious fatigue syndrome (PFS), it is surprising that after 10 years the proportion of PFS is reduced to the same extent in controls as in the cases. We therefore considered the bias to be prevalent and decided to exclude PFS from the model.

Model input summary

Table 1. Transition probabilities and distributions used in the outcome tree

Health outcome	Distribution of health states in health outcome	Transition probability			Source/assumption

(Health state)			
Symptomatic infection<br>(Mild)<br>(Severe)	95–98%<br>2–5%		Maurin & Raoult, 1999; Raoult, 2005
Chronic Q fever		1.6% (1.5–2%)	van der Hoek, 2011; ECDC, 2010
Fatal cases following symptomatic infection		1-2% of severe cases	ECDC, 2010
Fatal cases following chronic infection		5-50%	ECDC, 2010

Table 2. Disability weights and duration

Health outcome (Health state)	Disability Weight (DW) (Haagsma, 2015)		Duration	
	DW	Label	In years	Source
Symptomatic infection<br>(Mild)	0.007 (0.005-0.01)<br>0.125 (0.104-0.152)	Infectious disease, acute episode, mild	0.038<br>0.038	Stouthard, 1997<br>Stouthard, 1997

(Severe)		Infectious disease, acute episode, severe		
Latency period (before chronic Q fever)			0.5 (0.08-1.5)	Fenollar, 2001
Chronic Q fever	0.173 (0.14-0.205)	Heart failure, severe	1-1.5	CDC, 2013

References

Ayres JG, Flint N, Smith EG, Tunnicliffe WS, Fletcher TJ, Hammond K, et al. Post-infection fatigue syndrome following Q fever. QJM: Monthly Journal of the Association of Physicians, 1998 Feb;91(2):105-23.

Dijkstra F, van der Hoek W, Wijers N, Schimmer B, Rietveld A, Wijkmans CJ, et al. The 2007–2010 Q fever epidemic in the Netherlands: Characteristics of notified acute Q fever patients and the association with dairy goat farming. FEMS Immunology and Medical Microbiology. 2012;64(1):3-12 (epub 2011/11/10).

ECDC Technical Report – Risk Assessment on Q fever. May 2010. Available at:

http://www.ecdc.europa.eu/en/publications/Publications/1005_TER_Risk_Assessment_Qfever.pdf

Fenollar F, Fournier PE, Carrieri MP, Habib G, Messana T, Raoult D. Risks factors and prevention of Q fever endocarditis. Clinical Infectious Diseases, 2001 Aug 1;33(3):312-6.

Forland F, De Carvalho Gomes H, Nokleby H, Escriva A, Coulombier D, Giesecke J, et al. Applicability of evidence-based practice in public health: risk assessment on Q fever under an ongoing outbreak. Euro Surveill. 2012;17(3):pii=20060. Available at: http://www.eurosurveillance.org/ViewArticle.aspx?ArticleId=20060

Haagsma JA, Maertens de Noordhout C, Polinder S, Vos T, Havelaar AH, Cassini A, Devleesschauwer B, Kretzschmar ME, Speybroeck N, Salomon JA. Assessing disability weights based on the responses of 30,660 people from four European countries. Population Health Metrics 2015; 13: 10

Karakousis PC, Trucksis M, Dumler SJ: Chronic Q fever in United States. J Clin Microbiol 2006;44: 2283–2287.

Marrie TJ, Raoult D, 2010. Coxiella burnetii (Q Fever). In Mandell, Douglas, and Bennett's Principles and Practice of Infectious Diseases [eds] Gerald L. Mandell, John E. Bennett, Raphael Dolin.—7th edition 2010, Chapter 189, 2511-2519.Maurin M, Raoult D. Q fever. Clinical Microbiology Reviews. 1999;12(4):518-53 (epub 1999/10/09).

Morroy G, Peters JB, van Nieuwenhof M, Bor HH, Hautvast JL, van der Hoek W, et al. The health status of Q-fever patients after long-term follow- up. BMC Infectious Disease, 2011 Apr 18;11:97.

Q Fever Fact Sheet: Symptoms, Diagnosis, and Treatment – CDC, Atlanta. 2013. Available at: http://www.cdc.gov/qfever/symptoms/

Raoult D, Marrie T, Mege J. Natural history and pathophysiology of Q fever. Lancet Infectious Diseases, 2005 Apr;5(4):219-26.

Stouthard ME, Essink-Bot ML, Bonsel GJ, Barendregt JJ, Kramers PGN, van de Water HPA, et al. Disability Weights for Diseases in the Netherlands. Rotterdam, the Netherlands: Department of Public Health, Erasmus University Rotterdam, the Netherlands; 1997.

van der Hoek W, Meekelenkamp JC, Leenders AC, Wijers N, Notermans DW, Hukkelhoven CW. Antibodies against Coxiella burnetii and pregnancy outcome during the 2007-2008 Q fever outbreaks in The Netherlands. BMC Infectious Diseases, 2011 Feb 11;11:44.

Rabies

The initial symptoms of rabies resemble those of other systemic viral infections (Anderson, 1984). Two kinds of central nervous system (CNS) presentation can be seen: the furious form in 70% of all cases and the paralytic form in the remainder (WHO, 2013).

The furious form usually lasts around 12 days on average (range 9�17.8 days) (Udow, 2014). The paralytic form has a longer survival period of 22 days on average (range 18�28 days) and generally results in death.

Case fatality proportion

Once the symptomatic disease onset is confirmed the case fatality proportion is considered to be 100%.

Model input summary

Table 1. Transition probabilities used in the outcome tree

Health outcome<br><br>(Health state)	Distribution of health states<br><br>in health outcome	Transition probability	Source/assumption
Symptomatic infection<br><br>(Furious form)<br><br>(Paralytic form)	 <br>70%<br><br>30%		WHO, 2013
Fatal cases		100%	WHO, 2013

Table 2. Disability weights and duration

Health outcome<br><br>(Health state)	Disability Weight (DW) (Haagsma, 2015)		Duration		
	DW	Label	In years		Source
Symptomatic infection	0.655 (0.579-0.727)	Intensive Care Unit admission			

(Furious form)	As above	As above	0.033 (0.025-0.049)	Udow, 2014
(Paralytic form)	As above	As above	0.060 (0.049-0.077)	Udow, 2014

References

Anderson LJ, Nicholson KG, Tauxe RV, Winkler WG. Human rabies in the United States, 1960 to 1979: Epidemiology, diagnosis, and prevention. Ann Intern Med 1984;100:728-735.

Haagsma JA, Maertens de Noordhout C, Polinder S, Vos T, Havelaar AH, Cassini A, Devleesschauwer B, Kretzschmar ME, Speybroeck N, Salomon JA. Assessing disability weights based on the responses of 30,660 people from four European countries. Population Health Metrics 2015; 13: 10

Udow S, Marrie R, Jackson A. Clinical Features of Dog- and Bat-Acquired Rabies in Humans. Clinical Infectious Diseases Volume 57, Issue 5 Pp. 689-696.

WHO. Rabies. Fact Sheet N�99. Available at: http://www.who.int/mediacentre/factsheets/fs099/en/.

Rubella

Acquired rubella

Acquired, or non-congenital rubella usually gives rise to a mild rash and asymptomatic infections are common. The rash usually begins on the face and then progresses from head to foot. It lasts about three days and is occasionally pruritic (CDC, 2009). Since up to 50% of infections may not present with a rash, many cases are not detected or reported (CDC, 2009; Ang, 2010).

Risk of complications

The most relevant complications associated with rubella virus infection include arthritis or arthralgia, thrombocytopenia, and encephalitis (Zhou, 2004). Additional, but rare complications include orchitis, neuritis, bacterial superinfection, a late syndrome of progressive panencephalitis and mild hepatitis (CDC, 2009).

Arthritis/arthralgia

Arthralgia or arthritis may occur in 30–70% of adult women who contract rubella, but it is rare in children and adult males. It rarely develops into chronic arthritis (CDC, 2009; Mandell, 1999; Johnson, 1958). An age-independent range of 30–70% was estimated as the proportion of acute infections with this complication in the model, for females only. In 11 patients with rubella arthritis studied by Yanez et al. (Yanez, 1966), the onset of arthritis occurred one to six days after the beginning of the exanthem and lasted three to 28 days (mean of nine days).

Thrombocytopenic purpura

Hemorrhagic manifestations occur in approximately one case in 3 000 – more frequently in children than in adults – of which thrombocytopenic purpura is the most common (CDC, 2009; White, 1985; Mandell, 1999; Heggie, 1969; Boyer, 1965). Based on this estimated rate of occurrence (1/3 000), the proportion with the complication was estimated as 0.03% in the model.

Acute throbocytopenic purpura is commonly seen in children aged 1–7 years, and is defined as thrombocytopenia that lasts less than six months. In cases where thrombocytopenia persists for more than six months, it is considered chronic. Chronic thrombocytopenia occurs in a very small number of children (Taghizadeh, 2008).

Encephalitis

Encephalitis occurs in one in 5 000-6 000 cases, more frequently in adults (especially in females) than in children (CDC 2009; Mandell, 1999). Notwithstanding this occurrence rate, an age/sex-independent range of 0.01–0.02% was estimated for the proportion of acute cases with this complication in the model. The severity is highly variable. Symptoms in survivors usually resolve within 1–3 weeks without neurological sequelae (Gülen, 2008; Wolinsky, 1994).

Case fatality proportion

The case fatality proportion for thrombocytopenic purpura is 2.6% (Portielje, 2001). For encephalitis the overall lethality rate is 0–50% (CDC, 2009). Therefore in the model, the case fatality proportion following the health state thrombocytopenic purpura was specified with a point estimate of 4%, and the case fatality proportion following the health state encephalitis was set to the range of 20–50%.

Congenital rubella

Symptomatic infection occurs in 100% of infected foetuses between weeks 1 and 11. During weeks 11–20, symptomatic infection occurs in 30% of foetuses. After week 20 no foetus develops any manifestation of Congenital Rubella Syndrome (CRS) (Feigin, 2004). However, occasional foetal damage (deafness only) has been observed after the twentieth week (Mandell, 1999). Up to 50% of affected foetuses may appear healthy at birth and develop central nervous system abnormalities later (Duszak, 2009). Among children with CRS, 13% have one congenital defect, 24% have two defects and 63% have three or more defects (Reef, 2000).

We did not consider any loss of quality of life before birth and therefore the disability weight and duration for the symptomatic infection was set to 0.

Risk of sequelae

Hearing impairment occurs in 60% of children with CRS, heart disease in 45%, microcephaly in 27% (Reef, 2000), cataracts in 16–25% (Bloom, 2005), mental retardation in 13–25% (Lanzieri, 2004; Reef, 2000), and retinopathy in 5% (Reef, 2000). Overall, 20–40% of CRS survivors aged 35 or older have insulin-dependent diabetes (Mandell, 1999; Duszak, 2009) and 5% of survivors aged 13–19 develop some form of thyroid disease. (Duszak, 2009). Panencephalitis is a rare, fatal, late complication. The incidence of other late complications is still unknown (Duszak, 2009).

The case fatality ratio for infants with confirmed CRS is 10% (Reef, 2000).

Model input summary

Table 1. Transition probabilities used in the outcome tree

Health outcome<br><br>(Health state)	Distribution of health states in health outcome	Transition probability	Source/assumption

Acquired			
Symptomatic infection<br>(Arthritis/arthralgia)<br>(Thrombocytopenic purpura)<br>(Encephalitis) (Uncomplicated)	30–70%; females only<br>0.03%<br>0.01–0.02%<br>Remaining cases		CDC 2009, Mandell 1999, Johnson 1958<br>CDC 2009, White 1985<br>CDC 2009, Mandell 1999
Fatal cases following thrombocytopenic purpura		2.6%	Portielje, 2001
Fatal cases following encephalitis		0–50%	CDC, 2009
Congenital			
Permanent disability due to hearing impairment		60%	Reef, 2000
Permanent disability due to congenital heart defects		45%	Reef, 2000
Permanent disability due to microcephaly		27%	Reef, 2000
Permanent disability due to cataract		16–25%	Bloom, 2005
Permanent disability due to mental retardation		13–25%	Lanzieri, 2004; Reef, 2000
Permanent disability due to retinopathy		5%	Reef, 2000
Permanent disability due to insulin-dependent diabetes		20–40%	Mandell, 1999; Duszak, 2009 (aged >35 years)
Permanent disability due to thyroid gland dysfunction		5%	Duszak, 2009 (aged 13–19 years)
Fatal cases		10%	Reef, 2000

Table 2. Disability weights and duration

Health outcome (Health state)	Disability Weight (DW) (Haagsma, 2015)		Duration

	DW	Label	In years	Source/assumption
Symptomatic infection<br>(Uncomplicated)<br>(Arthritis/arthralgia)<br>(Thrombocytopenic purpura)<br>(Encephalitis)	0.007 (0.005–0.01)<br>0.344 (0.3–0.391)<br>0.167 (0.134–0.201)<br>0.41 (0.358–0.47)	Infectious disease, acute episode, mild<br>Musculoskeletal problems, generalised,moderate<br>Thrombocytopenic purpura<br>Encephalopathy - moderate	0.008<br>0.008–0.077<br>0.008–0.5<br>0.019–0.058	CDC, 2009<br>CDC, 2009<br>Yanez, 1996<br>Taghizadeh, 2008 Gülen, 2008; Wolinsky, 1994/without any neurological sequelae
Congenital				
Symptomatic infection	0		0	
Permanent disability due to hearing impairment	0.008–0.103	From lowest to highest hearing loss related DWs	Remaining life expectancy	
Permanent disability due to congenital heart defects	0.052–0.173	From lowest to highest heart failure related DWs	Remaining life expectancy	
Permanent disability due to microcephaly	0.011–0.421		Remaining life expectancy	

		From lowest to highest cognitive difficulties related DWs		
Permanent disability due to cataract	0.004–0.171	From lowest to highest visual impairment related DWs	Remaining life expectancy	
Permanent disability due to mental retardation	0.011–0.421	From lowest to highest cognitive difficulties related DWs	Remaining life expectancy	
Permanent disability due to retinopathy	0.004–0.171	From lowest to highest visual impairment related DWs	Remaining life expectancy	
Latency period before diabetes	0		35	Mandell, 1999; Duszak, 2009
Latency period before thyroid dysfunction	0		13–19	Duszak, 2009
Permanent disability due to insulin-dependent diabetes	0.07 (0.057–0.088)	Generic uncomplicated disease: worry and daily medication	Remaining life expectancy	
Permanent disability due to thyroid gland dysfunction	0.07 (0.057–0.088)	Generic uncomplicated disease: worry and daily medication.	Remaining life expectancy	

References

Ang LW, Chua LT, James L, Goh KT. Epidemiological surveillance and control of rubella in Singapore, 1991-2007. Ann Acad Med Singapore. 2010 Feb;39(2):95-101.

Assaad F, Ljungars-Esteves K. Rubella-world impact. Rev Infect Dis. 1985 Mar-Apr;7 Suppl 1:S29-36.

Boyer WL, Sherman FE, Michaels RH, et al. Purpura in congenital and acquired rubella. N Engl J Med. 1965;273:1362.

Bloom S, Rguig A, Berraho A, Zniber L, Bouazzaoui N, Zaghloul Z, et al. Congenital rubella syndrome burden in Morocco: a rapid retrospective assessment. Lancet. 2005 Jan 8-14;365(9454):135-41.

Centers for Disease Control and Prevention. Epidemiology and Prevention of Vaccine-Preventable Diseases. Atkinson W, Wolfe S, Hamborsky J, McIntyre L, eds. 11th ed. Washington DC: Public Health Foundation, 2009.

Duszak RS. Congenital rubella syndrome--major review. Optometry. 2009 Jan;80(1):36-43.

Feigin RD. Textbook of pediatric infectious diseases, Volume 1. 2004. Elsevier Health Sciences.

Haagsma JA, Maertens de Noordhout C, Polinder S, Vos T, Havelaar AH, Cassini A, Devleesschauwer B, Kretzschmar ME, Speybroeck N, Salomon JA. Assessing disability weights based on the responses of 30,660 people from four European countries. Population Health Metrics 2015; 13: 10

Heggie AD, Robbins FC. Natural rubella acquired after birth. Am J Dis Child. 1969;118:12.

Gülen F, Cagliyan E, Aydinok Y, Ozen S, Yildiz B. A patient with rubella encephalitis and status epilepticus. Minerva Pediatr. 2008 Feb;60(1):141- 4.

Johnson RE, Hall AP. Rubella arthritis. N Engl J Med. 1958;258:743.

Lanzieri TM, Parise MS, Siqueira MM, Fortaleza BM, Segatto TC, Prevots DR. Incidence, clinical features and estimated costs of congenital rubella syndrome after a large rubella outbreak in Recife, Brazil, 1999-2000. Pediatr Infect Dis J. 2004 Dec;23(12):1116-22

Mandell GL, Bennet JE, Dolin R. Mandell, Douglas and Bennett's Principles and Practice of Infectious Disease - 5th edition. Churchill Livingstone, 1999.

Portielje JE, Westendorp RG, Kluin-Nelemans HC, Brand A. Morbidity and mortality in adults with idiopathic thrombocytopenic purpura. Blood 2001 97:2549–2554.

Reef SE, Plotkin S, Cordero JF, Katz M, Cooper L, Schwartz B, et al. Preparing for elimination of Congenital Rubella Syndrome (CRS): summary of a workshop on CRS elimination in the United States. Clin Infect Dis. 2000 Jul;31(1):85-95

Sherman FE, Michaels RH, Kenny FM. Acute encephalopathy (encephalitis) complicating rubella. JAMA. 1965;192:675.

Steen E, Torp KH. Encephalitis and thrombocytopenic purpura after rubella. Arch Dis Child. 1956;31:470.

Taghizadeh M. An Update on Immune-Mediated Thrombocytopenia. Labmedicine. Volume 39, Number 1, January 2008.

White CC, Koplan JP, Orenstein WA. Benefits, risks and costs of immunization for measles, mumps and rubella. Am J Public Health. 1985 July; 75(7): 739–744.

Wolinsky JS. Rubella. In: Fields BM, Knipe DM, Chanock RM, et al., eds. Virology. 3rd ed. New York: Raven Press, 1994;899-929.

Yanez JE, Thompson GR, Mikkelsen WM, et al. Rubella arthritis. Ann Intern Med 64:772-777, 1966.

Zhou F, Reef S, Massoudi M, Papania MJ, Yusuf HR, Bardenheier B, et al. An Economic Analysis of the Current Universal 2-Dose Measles-Mumps- Rubella Vaccination Program in the United States. J Infect Dis. (2004) 189 Supplement 1: S131-S145.

Salmonellosis

Acute gastroenteritis associated with Salmonella infections in humans is, in most cases, self-limiting within a few days or weeks, but for some patients the disease is fatal. Studies estimated the duration to be 5.58 days for gastroenteritis cases not requiring medical help, 10.65 days for gastroenteritis cases visiting a doctor but not hospitalised and 16.15 days for hospitalised gastroenteritis cases (Kemmeren 2006).

The proportion of mild (uncomplicated), moderate (complicated, doctor) and severe (complicated, doctor) symptomatic infections is set at 83.3%, 15% and 1.7% (Kemmeren 2006; Kwong 2012; redistributing in order to total 100%)

In many reports bacteraemia is highlighted as a possible extra-intestinal complication of salmonellosis (0.03% of laboratory-confirmed cases, Ternhag 2008), although these complicated cases are often considered within the hospitalised proportion of cases (Cressey & Lake 2007; Kemmeren 2006).

The case fatality proportion for symptomatic salmonellosis cases ranged from 0.1% (Kemmeren 2006; Helms 2003) to 0.05% in salmonellosis outbreaks in Austria (Much 2005) and 0.3 for non-typhoid infections in England and Wales (Adak, 2002). These were in line with case fatality proportions observed in cases reported to TESSy between 2009 and 2013 (personal communication).

We chose to estimate the overall case fatality proportion as being within the range 0.05–0.1% and assumed a different age-group distribution of this risk, based on the age-group distribution of deaths reported to TESSy between 2009 and 2013 (see Table 3). This table is based on all TESSy notified cases from EU Member States except Bulgaria, Latvia and Poland which report only aggregate data, and Italy because the outcome was not reported.

Risk of complications

Reactive arthritis (ReA) and Irritable Bowel Syndrome (IBS) are the most frequent sequelae of salmonellosis reported in the literature (Haagsma 2009; Raybourne 2003). The frequency of other post-infectious complications following salmonellosis is extremely low and these were disregarded in the current study.

Reactive arthritis (ReA)

Many studies reported ReA as sequelae of salmonellosis (Keat 1983; Fendler 2001; Raybourne, 2003). A review of the literature, which included mostly cases of salmonellosis occurring during outbreaks, estimated that 8% (2.3–15%) of cases are at risk of developing ReA (Raybourne, 2003), although most of these studies have estimated risk based on laboratory-confirmed cases and duration of diarrhoea is highly correlated with the development of ReA (Yu & Thomson, 1994). In order to account for the considerable uncertainty, the risk of developing ReA from all symptomatic cases is set at 1.31% (0.29–5.43%) (Kemmeren, 2006).

Little is known about the duration of ReA; the average duration is set at between 1.5 months, derived from Hannu et al. (Hannu, 2005) and 222 days (Kemmeren, 2006).

Irritable bowel syndrome (IBS)

In a recent literature review, 8.8% (7.2–10.4%) of symptomatic salmonellosis cases were considered at risk of developing IBS, irrespective of age and gender; the duration was set to five years (Haagsma, 2010). However, the causality is largely debated and the impact of concurrent factors significant. Therefore, IBS is not considered as part of the salmonellosis outcome tree in our study.

Model input summary

Table 1. Transition probabilities and distributions used in the outcome tree

Health outcome (Health state)	Distribution of health states in health outcome	Transition probability	Source/assumption
Symptomatic infection:<br>(Uncomplicated)<br>(Complicated, doctor)<br>(Complicated, hospital)	83.3%<br>15%<br>1.7%		Kemmeren, 2006; Kwong, 2012
Fatal cases following symptomatic infection		0.05–0.1%<br>Age dep. Table 3	Kemmeren, 2006; Much, 2005; TESSy 2009-2013
Reactive arthritis		1.31% (0.29-5.43%)	Kemmeren, 2006

Table 2. Disability weights and duration

Health outcome (Health state)	Disability Weight (DW) (Haagsma, 2015)		Duration
	DW	Label	In years

					Source
Symptomatic infection<br>(Uncomplicated)<br>(Complicated, doctor)<br>(Complicated, hospital)	0.073	(0.061–0.092)<br>0.149 (0.12–0.182)<br>0.239 (0.202–0.285)	Diarrhoea, mild<br>Diarrhoea, moderate<br>Diarrhoea, severe	0.015<br>0.029<br>0.044	Kemmeren, 2006
Reactive arthritis	0.344	(0.3–0.391)	Musculoskeletal problems, generalised, moderate	0.131–0.608	Hannu, 2002

Table 3. Age-group redistribution of CFR (0.05–0.1%)

Age groups	%
0	0.69
1–4	1.72
5–9	1.38
10–14	0.34
15–19	1.03
20–24	0.00
25–29	1.72
30–34	0.34
35–39	1.03
40–44	0.69
45–49	2.07

50–54	3.45
55–59	4.14
60–64	5.17
65–69	9.31
70–74	12.41
75–79	16.55
80–84	18.62
>85	19.31
All ages	100.00

References

Adak GK, Long SM, O'Brien SJ. Trends in indigenous foodborne disease and deaths, England and Wales: 1992 to 2000. Gut. 2002 Dec;51(6):832- 41.

Cressey PJ and Lake R (2007). Risk ranking: Estimates of the burden of foodborne disease for New Zealand. Institute of Environmental Science & Research Limited; Christchurch Science Center.

Fendler CS, Laitko HH, Sorensen C, Gripenberg-Lerche A, Groh J, Uksila K et al. Frequency of triggering bacteria in patients with reactive arthritis and undifferentiated oligoarthritis and the relative importance of the tests used for diagnosis. Ann Rheum Dis 2001;60(4): 337-43.

Food Standards Agency (2000). A Report of the Study of Infectious Intestinal Disease in England. Food Standards Agency London: HMSO.

Haagsma JA, Havelaar AH, Janssen BM, Bonsel GJ. Disability Adjusted Life Years and minimal disease: application of a preference-based relevance criterion to rank enteric pathogens. Popul Health Metr 2008;6:7.

Haagsma JA, van der Zanden BP, Tariq L, van Pelt W, van Duynhoven YTHP, Havelaar AH. Disease burden and costs of selected foodborne pathogens in the Netherlands, 2006. Bilthoven: National Institute of Public Health and Environment. 2009. RIVM rapport 330331001. Available online at: www.rivm.nl/bibliotheek/rapporten/330331001.pdf

Haagsma JA, Siersema PD, De Wit NJ, Havelaar AH. Disease burden of post-infectious irritable bowel syndrome in the Netherlands. Epidemiol Infect 2010;138(11):1650-6.

Haagsma JA, Maertens de Noordhout C, Polinder S, Vos T, Havelaar AH, Cassini A, Devleesschauwer B, Kretzschmar ME, Speybroeck N, Salomon JA. Assessing disability weights based on the responses of 30,660 people from four European countries. Population Health Metrics 2015; 13: 10

Hannu TL, Mattila H, Rautelin P, Pelkonen P, Lahdenne A, Siitonen M et al. Campylobacter-triggered reactive arthritis: a population-based study. Rheumatology 2002;41(3): 312-318.

Havelaar AH, de Wit MAS, van Koningsveld R, van Kempen E (2000a). Health burden in the Netherlands due to infection with thermophilic Campylobacter spp. Epidemiol Infect 2000;125(3): 505-22.

Havelaar AH, de Wit MAS, van Koningsveld R (2000b). Health burden in the Netherlands (1990–1995) due to infection with thermophilic Campylobacter species. Bilthoven, the Netherlands: National Institute for Public Health and the Environment. Report no. 284550004.

Havelaar AH, Ivarsson S, Löfdahl M, Nauta MJ. Estimating the true incidence of campylobacteriosis and salmonellosis in the EU. 2012. [Submitted for publication].

Helms M, Vastrup P, Gerner-Smidt P, Molbak K. Short and long term mortality associated with foodborne bacterial gastrointestinal infections: registry based study. BMJ 2003; 326(7385): 357-60.

Keat A. Reiter's syndrome and reactive arthritis in perspective. N Engl J Med 1983;309(26): 1606-15.

Kemmeren JM, Mangen MJJ, van Duynhoven YTHP, Havelaar AH. Priority setting of foodborne pathogens: disease burden and costs of selected enteric pathogens. Bilthoven: National Institute of Public Health and Environment: RIVM rapport 330080001. 2006. Available online at: www.rivm.nl/bibliotheek/rapporten/330080001.pdf

Mangen MJJ, Havelaar AH, de Wit GA. Campylobacteriosis and sequelae in the Netherlands - Estimating the disease burden and the cost-of- illness. Bilthoven: National Institute for Public Health and the Environment; Report no. 250911004. 2004. Available online at: www.rivm.nl/bibliotheek/rapporten/250911004.pdf

Mangen MJJ, Havelaar AH, Bernsen RAJAM, van Koningsveld R, de Wit GA. The costs of human Campylobacter infections and sequelae in the Netherlands: A DALY and cost-of-illness approach. Acta Agriculturae Scandinavica Section C Food Economics 2005;(1): 35-51.

Melse JM and Kramers PGN (1998). Berekening van de ziektelast in Nederland. Achtergronddocument bij VTV-1997; deel III, hoofdstuk 7. Bilthoven; Report no. 431501028.

Much P, Pichler J, Allerberg F. Food borne infectious outbreaks, Austria 2005." Wien Klin Wochenschr 2007;119 (5-6):150-7.

Raybourne RB, Williams KM, Roberts T and Arthritis Working Group. Food Poisoning: Economic Implications. In: Caballero BL, Trugo P, Finglas (Editors). Encylopaedia of Food Sciences and Nutrition. 10 volume set edition. London: 2003; 2672-82.

Robert Koch Institut. Infektionsepidemiologisches Jahrbuch (various years). Available online at:

www.rki.de/cln_178/nn_205772/DE/Content/Infekt/Jahrbuch/jahrbuch__node.html?__nnn=true

Ternhag A, Törner A, Svensson A, Ekdahl K, Giesecke J. Short- and long-term effects of bacterial gastrointestinal infections. Emerg Infect Dis 2008;14 (1):143-8.

WHO. Global Burden of Disease 2004 Update: Disability weights for diseases and conditions. Geneva: 2004, WHO. Available online at: http://www.who.int/healthinfo/global_burden_disease/GBD2004_DisabilityWeights.pdf

Yu DTY and Thomson GTD. Clinical, epidemiological and pathogenic aspects of reactive arthritis. Food-Microbiology 1994;11(2): 97-108.

Shigellosis

Acute gastroenteritis associated with Shigella spp. infections in humans is, in most cases, self-limiting within days to weeks, but for a few patients the disease may be severe and fatal.

We assume that more complicated cases visit their doctor or are hospitalised and will subsequently be laboratory-tested and reported as confirmed. The proportion of reported cases over the total symptomatic cases is 5.45% (2.18–40%) (Haagsma, 2010).

We assumed a similar duration of symptoms as for salmonellosis: 5.58 days for uncomplicated cases and 10.65–16.15 for complicated ones (Kemmeren, 2006).

On average, patients aged 65 years and over are hospitalised for a greater number of days and are more likely to die of shigellosis than other patients (van Pelt, 2010; Barton Behravesh, 2011). We assumed that only complicated cases lead to fatalities and set the case fatality proportion for complicated cases as 0.06–0.97% (Van Pelt, 2010; Barton Behravesh, 2011). Assuming a different age-group distribution of this risk, we distributed the case fatality proportion based on the age-group distribution of deaths reported to TESSy between 2009 and 2013 (see Table 3). This table is based on all TESSy-notified cases from EU Member States except Bulgaria, Lithuania and Poland, because they report only aggregate data, and Liechtenstein, Luxembourg and Italy which do not report on the death outcome.

Risk of complications

Reactive arthritis (ReA), Post-Infectious Irritable Bowel Syndrome (PI-IBS), Haemolytic Uraemic Syndrome (HUS) and End-stage Renal Disease (ESRD) are possible sequelae of shigellosis.

Asymptomatic cases, which themselves do not have a disease burden for acute illness, might also develop sequelae. However neither the number of asymptomatic cases in the population, nor the percentage of asymptomatic cases that develop sequelae is known and these are therefore not included in the model.

Reactive arthritis (ReA)

The risk of developing ReA has been found to be 6.6% of all laboratory-confirmed cases of shigellosis (Hannu, 2005), 1.2% (Rees, 2004) and 9.8% (Schiellerup, 2008). However, severity of the acute infection and duration of diarrhoea are associated with a higher risk of developing ReA (Townes, 2008; Hannu, 2005; Rees, 2004; Schiellerup, 2008); moreover, these figures relate to laboratory-confirmed cases only. Therefore, we assume that only 'complicated' cases have a risk of 6.6% (1.2-9.8%) of developing ReA.

Little is known about the duration of ReA; the average duration is set between 1.5 months (Hannu, 2005) and 222 days (Kemmeren, 2006).

Irritable bowel syndrome (IBS)

In a recent literature review, 8.8% (7.2–10.4%) of symptomatic infections involving foodborne pathogens (salmonellosis, campylobacteriosis and shigellosis) were associated with a risk of developing IBS, irrespective of age and gender. The duration of IBS was set to five years (Haagsma, 2010). However, the causality is largely debated and the impact of concurrent factors significant. Therefore, IBS is not considered as part of the shigellosis outcome tree in our study.

Haemolytic uraemic syndrome (HUS)

>HUS is characterised by haemolytic anaemia (severe anaemia due to increased destruction of red blood cells), thrombocytopenia (reduced platelet count) and impaired kidney function (acute renal failure). Haemolytic anaemia and thrombocytopenia often occur after bloody diarrhoea. Acute renal failure may then follow.

Several studies have associated HUS with shigellosis infections, in particular Shigella dysenteriae type 1, a species which occurs mainly in tropical countries and accounts for approximately 30% of S. dysenteriae isolates in those countries (Mark Taylor, 2008; Chopra, 1997; Bennis, 2006; Kotloff, 1999; Ekdahl, 2005).

In Europe, based on data reported to TESSy, S. dysenteriae accounts for less than 3% of laboratory-confirmed shigellosis cases, whereas S. sonnei is the most common Shigella species (ECDC, 2013 a & b). This means that around 0.9% of the shigellosis cases occurring in Europe, caused by Shigella dysenteriae type 1, are at risk of developing HUS; however, the risk varies according to EU Member State.

The incidence of S. dysenteriae-induced HUS is unknown and it is affected by antibiotic treatment (Bennish, 2006). HUS caused by S. dysenteriae type 1 is often perceived as more severe than HUS caused by enterohaemorrhagic E. coli (EHEC), however this is probably due to the fact that such infections mainly occur in countries with limited access to high-quality healthcare. Though the age range of Shigella-induced- HUS is wider and the 'median time from the onset of diarrhoea to the presentation of HUS' is longer, HUS caused by Shigella and EHEC is very similar (Mark Taylor, 2008). Therefore, we assume that the risk of developing HUS after symptomatic infection with Shigella dysenteriae type 1 is the same as the risk for symptomatic infections with Shiga-toxin producing E. coli O157 (STEC), around 0.94–1.25% (Cressey & Lake, 2007).

Given that 0.9% of shigellosis cases occurring in Europe are caused by Shigella dysenteriae type 1, the overall risk of developing HUS after symptomatic shigellosis is set to 0.008–0.011%.

HUS occurs mainly in children aged one to five years, and less frequently in children over five years. In one study (Havelaar, 2003) 72% of all HUS cases were under 15 years of age, and 28% were older. The distribution of HUS patients admitted to the Paediatric Nephrology Department of University Hospital Nijmegen from 1974–1993 was used for cases under 15 years (Havelaar, 2003). For the current study we distributed the age risk of developing HUS (0.008-0.011%) according to TESSy-notified cases of HUS by age due to VTEC infection from 2009 to 2013 (see Table 4). Cases were from all EU Member States except Bulgaria and Lithuania, because they report only aggregate data, and Italy because it has sentinel surveillance for STEC/VTEC which does not provide sufficient coverage.

Duration of HUS is reported to be seven days (range 3–31 days) and 41% (19/46) of patients were admitted to hospital (McPherson, 2011). Hospitalisation is reported to last 2–4 weeks for HUS patients (Havelaar, 2003).

The case fatality proportion is assumed to be 3.7% (Cressey & Lake, 2007; Oxford Medical Dictionary, 2003; Havelaar, 2003). This low case- fatality might be valid for cases up to 65 years and be as high as 56% for those aged ≥65 years as data from an outbreak in Scotland suggests (Dundas, 1999). For the current study we use age-specific fatality proportions as reported by Havelaar et al. (Havelaar, 2003; see Table 5).

End-stage renal disease (ESRD)

ESRD is one of the most serious outcomes associated with HUS and is the most advanced stage of kidney failure (Oxford Medical Dictionary, 2003). HUS cases may develop ESRD briefly after HUS or after a long latency period. In the current study we assume that 13.4% develop ESRD, of which 2.9% briefly after HUS and 10.5% after a latency period of 20 years (Havelaar, 2004; Cressey & Lake, 2007). We also assume that all cases experiencing ESRD are in dialysis treatment until transplantation occurs.

The case-fatality proportion is based on the assumption that in the first year after starting dialysis mortality ratios are relatively high and differ between age-groups (see Table 6) and that only few fatalities occur after renal transplantation (Havelaar, 2003). Duration of dialysis (time to transplantation) is age-dependent and is applied to the duration of ESRD, see Table 7 (Havelaar, 2003).

Model input summary

Table 1. Transition probabilities used in the outcome tree

Health outcome<br>(Health state)	Distribution of health states in health outcome	Transition probability	Source/assumption
Symptomatic infection<br>(Uncomplicated)<br>(Complicated)	Rem. cases<br>5.45% (2.18–40%)		Haagsma, 2010
Fatal cases following complicated symptomatic infection		0.06–0.97<br>Age dep.Table 3	Van Pelt, 2010; Barton Behravesh, 2011; TESSy 2009–2013
ReA		6.6% (1.2–9.8%)	Hannu, 2005; Rees, 2004; Townes, 2008; Schiellerup, 2008
HUS		0.008–0.011%<br>Age dep.<br>Table 4	Mark Taylor, 2008; Chopra, 1997; Bennis, 2006; Kotloff, 1999; Ekdahl, 2005; ECDC, 2013 a & b; Cressey & Lake, 2007
Latency period before ESRD		10.5%	Havelaar, 2004; Cressey & Lake, 2007
ESRD after HUS		2.9%	Havelaar, 2004; Cressey and Lake, 2007

ESRD after latency period		100%	
Fatal cases following HUS		< 65 years: 3.7%<br>>=65 years: 56%<br>Table 5	Haavelar, 2004; Dundas, 1999
Fatal cases following ESRD		Age dep. & different for dialysis and transplantation<br>See Table 6.	Havelaar, 2003<br>see Table 6
Transplanted		Remaining %	

Table 2. Disability weights and duration

Health outcome<br>(Health state)	Disability Weight (DW) (Haagsma, 2015)		Duration	
	DW	Label	In years	Source/assumption
Symptomatic infection<br>(Uncomplicated)<br>(Complicated)	0.073–0.149<br>0.239 (0.202–0.285)	Diarrhoea, from mild to moderate<br>Diarrhoea, severe	0.015<br>0.029–0.044	Kemmeren, 2006
ReA	0.344 (0.3–0.391)	Musculoskeletal problems, generalised, moderate	0.131-0.608	Estimated from Hannu, 2005; Kemmeren, 2006
HUS	0.108 (0.09–0.132)	Chronic kidney disease (stage IV)	0.019 (0.008-0.085)	McPherson, 2011
ESRD	0.487 (0.432–0.544)	End-stage renal disease, on dialysis	See Table 7	Assuming that all ESRD are in dialysis

Transplanted	0.070 (0.057–0.088)	Generic uncomplicated disease: worry and daily medication	Remaining life expectancy	Assuming no risk of re-transplantation

Table 3. Age group distribution of the case fatality proportion (0.06–0.97%)

Age groups	%
0	0.00
1–4	10.00
5–9	10.00
10–14	0.00
15–19	0.00
20–24	0.00
25–29	0.00
30–34	10.00
35–39	0.00
40–44	10.00
45–49	20.00
50–54	0.00
55–59	0.00
60–64	10.00
65–69	0.00
70–74	0.00
75–79	10.00
80–84	10.00
>85	10.00
All ages	100.00

Table 4. Age-group redistribution of risk of developing HUS (0.008–0.011%) following infection (TESSy 2009– 2013)

Age groups	%
0	5.67
1–4	33.74
5–9	13.09
10–14	6.62

15–19	2.88
20–24	2.27
25–29	3.83
30–34	3.54

35–39	2.88
40–44	3.40
45–49	3.45
50–54	2.36
55–59	2.88
60–64	3.02
65–69	2.27
70–74	3.36
75–79	1.89
80–84	1.65
85+	0.99
All ages	100

Table 5. HUS case-fatality proportion per age group

Age groups	CFR
0–65	3.7%
>65	56%

Table 6. Case-fatality proportions in the first year after starting dialysis and after renal transplantation

Age class	Case-fatality ratio dialysis	Case-fatality ratio renal transplantation
0–14	4.1% (0.9–11.1%)	7% (2.2–16%)
15–44	8.7% (5.8–12.4%)	7% (2.2–16%)
45–64	37% (31–44%)	7% (2.2–16%)
65–74	65% (58–72%)	7% (2.2–16%)
75+	79% (70–87%)	7% (2.2–16%)

Table 7. Age-specific duration of dialysis

Age class	Duration of dialysis
0–14	1.7 (0.2–5.3)
15–44	2.5 (0.2–9.6)
45–64	6.7 (0.5–30)
>65	5 to remaining life expectancy

References

Barton Behravesh C, Jones TF, Vugia DJ, Long C, Marcus R, Smith K et al. Deaths associated with bacterial pathogens transmitted commonly through food: foodborne diseases active surveillance network (FoodNet), 1996-2005. J Infect Dis, 2011. 204(2):263-7.

Bennish ML, Khan WA, Begum M, Bridges EA, Ahmed S, Saha D, et al. Low risk of hemolytic uremic syndrome after early effective antimicrobial therapy for Shigella dysenteriae type 1 infection in Bangladesh. Clin Infect Dis, 2006. 42(3):356-62.

Chopra, M., D. Wilkinson, and S. Stirling, Epidemic shigella dysentery in children in northern KwaZulu-Natal. S Afr Med J, 1997. 87(1):48-51.

Cressey PJ, Lake R. Risk ranking: Estimates of the burden of foodborne disease for New Zealand, 2007, Institute of Environmental Science & Research Limited; Christchurch Science Center: Christchurch.

Dundas S, et al. Effectiveness of therapeutic plasma exchange in the 1996 Lanarkshire Escherichia coli O157:H7 outbreak. Lancet, 1999. 354(9187):1327-30.

Ekdahl, K. and Y. Andersson, The epidemiology of travel-associated shigellosis--regional risks, seasonality and serogroups. Journal of Infection, 2005. 51(3):222-229.

European Centre for Disease Prevention and Control. Annual Epidemiological Report 2012. Reporting on 2010 surveillance data and 2011 epidemic intelligence data. Stockholm: ECDC; 2013.

European Centre for Disease Prevention and Control. Annual Epidemiological Report 2013. Reporting on 2011 surveillance data and 2012 epidemic intelligence data. Stockholm: ECDC; 2013.

Haagsma JA, Siersema PD, De Wit NJ, Havelaar AH. Disease burden of post-infectious irritable bowel syndrome in the Netherlands. Epidemiol Infect, 2010. 138(11):1650-6.

Haagsma JA, Maertens de Noordhout C, Polinder S, Vos T, Havelaar AH, Cassini A, Devleesschauwer B, Kretzschmar ME, Speybroeck N, Salomon JA. Assessing disability weights based on the responses of 30,660 people from four European countries. Population Health Metrics 2015; 13: 10

Hannu T, Mattila L, Siitonen A, Leirisalo-Repo M. Reactive arthritis attributable to Shigella infection: a clinical and epidemiological nationwide study. Annals of the rheumatic diseases, 2005. 64(4):594.

Havelaar AH, Van Duynhoven YT, Nauta MJ, Bouwknegt M, Heuvelink AE, De Wit GA, et al. Disease burden in the Netherlands due to infections with Shiga toxin-producing Escherichia coli O157. Epidemiol Infect, 2004. 132(3):467-84.

Havelaar AH, Mangen MJJ, van Duynhoven YTHP, Havelaar, AH. Disease burden in the Netherlands due to infections with Shiga toxin-producing Escherichia coli O157., in RIVM report 284550008/20032003, National Institute of Public Health and the Environment: Bilthoven, 2003.

Kemmeren J, Mangen MJJ, van Duynhoven YTHP, Havelaar AH. Priority setting of foodborne pathogens: disease burden and costs of selected enteric pathogens. RIVM report. National Institute of Public Health and the Environment: Bilthoven, 2006.

2006. Available at: http://www.rivm.nl/bibliotheek/rapporten/330080001.pdf.

Mark Taylor C. Enterohaemorrhagic Escherichia coli and Shigella dysenteriae type 1-induced haemolytic uraemic syndrome. Pediatric Nephrology, 2008. 23(9):1425-1431.

McPherson M, et al. Economic costs of Shiga toxin-producing Escherichia coli infection in Australia. Foodborne Pathogen Diseases, 2011. 8(1):55-62.

Rees JR, et al., Persistent diarrhea, arthritis, and other complications of enteric infections: a pilot survey based on California FoodNet surveillance, 1998-1999. Clin Infect Dis, 2004. 38 Suppl 3: S311-7.

Schiellerup P, Krogfelt KA, Locht H. A comparison of self-reported joint symptoms following infection with different enteric pathogens: effect of HLA-B27. J Rheumatol, 2008. 35(3): p. 480-7.

Tariq L, Haagsma J, Havelaar A. Cost of Illness and Disease Burden in The Netherlands Due to Infections with Shiga ToxinProducing Escherichia coli O157. Journal of Food Protection&# 174;, 2011. 74(4):545-552.

Townes JM, et al. Reactive arthritis following culture-confirmed infections with bacterial enteric pathogens in Minnesota and Oregon: a population-based study. Ann Rheum Dis, 2008. 67(12): 1689-96.

Van Pelt W, et al. Trends in Gastro-enteritis in Nederland; notitie met betrekking tot 2009 en 2010, in RIVM report project V/210221 December 2010, RIVM: Bilthoven.

STEC/VTEC

The current disease model relies strongly on publications focused around STEC/VTEC O157 infections. Shiga toxin-producing Escherichia coli O157 (STEC/VTEC O157) infection may be asymptomatic, or may result in acute gastroenteritis (GE), and potentially in haemorrhagic colitis: 44.5% of cases had bloody diarrhoea (Michel, 2000). Duration is assumed to be longer than for non-bloody diarrhoea (Havelaar, 2004): median duration of five days and three days for bloody and non-bloody diarrhoea respectively (Cressey & Lake, 2007), which are proposed in the model as a uniform distribution.

There is little information on STEC/VTEC-associated mortality. Study findings range from 0.083% of the total estimated/VTEC O157:H7 (Mead, 1999), 0.03% (Buzby & Roberts, 2009), 0.04% (Walkerton outbreak, one fatal case in 2 321 patients, Bruce-Grey-Owen Sound Health Unit, 2000) and 0.045 (Havelaar, 2004). We therefore assume a uniformly distributed case-fatality proportion of between 0.03% and 0.045% for this study.

Fatal cases occur mainly in elderly people (Bauch, 2007); therefore, we assumed that the case fatality proportion of 0.03–0.045% is distributed across age-groups in accordance with the observed age-group distribution of TESSy-reported deaths between 2009 and 2013 (see Table 3). This table is based on all TESSy-notified cases from EU Member States except Bulgaria and Lithuania, because they report only aggregate data, and Italy because it has sentinel surveillance for STEC/VTEC for which we do not have the coverage.

Risk of complications

STEC/VTEC infection has been associated with post-diarrhoeal haemolytic uremic syndrome (HUS), which may result in death, end-stage renal disease (ESRD) or other sequelae. HUS and ESRD are the most frequently occurring sequelae of STEC and will be considered in the outcome tree. Irritable Bowel Syndrome (IBS) is another frequently occurring sequelae of bacteria-triggered gastroenteritis (Haagsma, 2010; Marshall, 2010; Thabane, 2009) and was considered for inclusion in the outcome tree (see below). The frequency of other post-infectious complications following STEC is low and they were therefore disregarded (Havelaar, 2004; Frenzen, 2005; Cressey & Lake, 2007; Buzby, 2009; McPherson, 2011; Tariq, 2011).

Haemolytic uraemic syndrome (HUS)

Haemolytic Uraemic Syndrome (HUS) is 'a condition in which sudden rapid destruction of red blood cells causes acute renal failure' (Oxford Medical Dictionary, 2003). HUS may occur following a respiratory or gastrointestinal infection, especially by pathogenic Escherichia coli or

Shigella spp.

The risk of developing HUS after STEC/VTEC infection has been found to be 3–7% (McPherson, 2011), 1% (Havelaar, 2004), 0.94–1.25% (Cressey & Lake, 2007) and 1.6% of laboratory-confirmed EHEC infections although authors mention under-estimation due to misclassification (13/820; Ternhag, 2008). In the current study we assume that the probability of developing HUS after a VTEC/STEC symptomatic infection is 0.94–1.25%.

HUS occurs mainly in children between the ages of one and five years, and less frequently in children over five years. In one study, 72% of all HUS cases were under 15 years of age and 28% were older (Havelaar, 2003). Member States report HUS outcomes relating to STEC/VTEC infections and we therefore redistributed the age-group risk of developing HUS (0.94–1.24%) based on the age-group of HUS cases reported to TESSy between 2009 and 2013 (all Member States except Bulgaria, Italy and Lithuania) (see Table 4).

Duration is reported to be seven days (range 3–31 days) and 41% (19/46) of patients were admitted to hospital (McPherson, 2011); hospitalisation is reported to last two to four weeks for HUS patients (Havelaar, 2003).

The case fatality proportion was found to be 3.7% (Cressey & Lake, 2007; Oxford Medical Dictionary, 2003; Havelaar, 2003). This low case- fatality may be valid for cases up to 65 years and then as high as 56% for cases ≥65 years, as indicated by data from an outbreak in Scotland (Dundas, 1999). Other studies assume age-specific fatality rates, as reported by Havelaar et al. (Havelaar, 2003). We estimated the age-group case fatality proportion from HUS based on STEC/VTEC infections notified to TESSy between 2009 and 2013 from all Member States, except Bulgaria, Italy and Lithuania (see Table 5).

End-stage renal disease (ESRD)

ESRD is one of the most serious outcomes associated with HUS and is the most advanced stage of kidney failure (Oxford Medical Dictionary, 2003). HUS cases may develop ESRD briefly after HUS or after a long latency period. In the current study we assume that 13.4% develop ESRD, 2.9% of whom develop it briefly after HUS and 10.5% after a latency period of 20 years (Havelaar, 2004; Cressey & Lake, 2007). We also assume that all cases experiencing ESRD undergo dialysis treatment until transplantation occurs.

The case-fatality proportion is based on the assumption that in the first year after starting dialysis mortality is relatively high and differs among age-groups (see Table 6) and that only few fatalities occur after renal transplantation (Havelaar, 2003). Duration of dialysis (time to transplantation) is age-dependent and is applied to the duration of ESRD – see Table 7 (Havelaar, 2003).

Irritable bowel syndrome (IBS)

In a recent literature review, 8.8% (7.2-10.4%) of symptomatic infections with foodborne pathogens were considered at risk of developing IBS, irrespective of age and gender; the duration was set to 5 years (Haagsma, 2010). However, the causality is largely debated and the impact of concurrent factors significant. Therefore, IBS is not considered as part of the STEC/VTEC outcome tree in our study.

Model input summary

Table 1. Transition probabilities and distributions used in the outcome tree

Health outcome (Health state)	Distribution of health states in health outcome	Transition probability	Source/assumption
Fatal cases following symptomatic infection		0.03-0.045% Age-dependent (Table 3)	Buzby & Roberts, 2009; TESSy 2009-2013
Haemolytic uraemic syndrome (HUS)		0.94-1.25% Age-dependent (Table 4)	Havelaar, 2004; Cressey and Lake, 2007; TESSy 2009-2013
Latency period before ESRD		10.5%	Havelaar, 2004; Cressey and Lake, 2007
ESRD after HUS		2.9%	Havelaar, 2004; Cressey and Lake, 2007
ESRD after latency period		100%	
Fatal cases following HUS		Age-dependent (Table 5)	TESSy 2009-2013
Fatal cases following ESRD		Age-dependent, different for dialysis and transplantation (Table 6)	Havelaar, 2003 see Table 6
Transplanted		Remaining %	

Table 2. Disability weights and duration

Health outcome (Health state)	Disability Weight (DW) (Haagsma, 2015)		Duration	
	DW	Label	In years	Source
Symptomatic infection (Gastroenteritis)	0.149 (0.12-0.182)	Diarrhoea, moderate	0.008-0.014	Havelaar, 2004; Cressey & Lake, 2007
HUS	0.108 (0.09–0.132)	Chronic kidney disease (stage IV)	0.019 (0.008–0.085)	McPherson, 2011
ESRD	0.487 (0.432–0.544)	End-stage renal disease, on dialysis	Age dependent(See Table 7)	Assuming that all ESRD are in dialysis
Transplanted	0.070 (0.057–0.088)	Generic uncomplicated disease: worry and daily medication	Remaining life expectancy	

Table 3. Age-group redistribution of case fatality proportion (0.03–0.045%)

Age groups	%
0	4.30
1-4	9.68
5-9	4.30
10-14	0.00
15-19	0.00
20-24	2.15
25-29	0.00
30-34	0.00
35-39	3.23
40-44	3.23
45-49	2.15
50-54	1.08
55-59	4.30

60-64	8.60
65-69	4.30
70-74	10.75
75-79	10.75
80-84	15.05
>85	16.13
All ages	100.00

Table 4. Age-group redistribution of risk of developing haemolytic uraemic syndrome (0.94–1.25%)

Age	%
0	5.67
1-4	33.74
5-9	13.09
10-14	6.62
15-19	2.88
20-24	2.27
25-29	3.83
30-34	3.54
35-39	2.88
40-44	3.40
45-49	3.45
50-54	2.36
55-59	2.88
60-64	3.02
65-69	2.27
70-74	3.36
75-79	1.89
80-84	1.65
85+	0.99
All ages	100

Table 5. Age-group case fatality proportion from haemolytic uraemic syndrome (TESSy 2009–2013)

Age	%

0	6.06
1-4	2.63
5-9	3.25
10-14	0.00

15-19	0.00
20-24	5.13
25-29	0.00
30-34	0.00
35-39	3.64
40-44	3.28
45-49	3.17
50-54	2.13
55-59	2.00
60-64	4.44
65-69	8.33
70-74	4.62
75-79	17.86
80-84	25.93
85+	28.57
All ages	3.91

Table 6. Case-fatality proportions in the first year after starting dialysis and after renal transplantation

Age class	Case-fatality ratio dialysis	Case-fatality ratio renal transplantation
0–14	4.1% (0.9–11.1%)	7% (2.2–16%)
15–44	8.7% (5.8–12.4%)	7% (2.2–16%)
45–64	37% (31–44%)	7% (2.2–16%)
65–74	65% (58–72%)	7% (2.2–16%)
75+	79% (70–87%)	7% (2.2–16%)

Table 7 .Age specific duration of dialysis

Age class	Duration of dialysis
0-14	1.7 (0.2-5.3)
15-44	2.5 (0.2-9.6)
45-64	6.7 (0.5-30)

>65	5 years to remaining life expectancy

References

Bauch CT, Anonychuk AM, Pham BZ, Gilca V, Duval B, Krahn MD (2007). Cost-utility of universal hepatitis A vaccination in Canada. Vaccine 25(51): 8536-48.

Buzby JC, Roberts T (2009). The economics of enteric infections: human foodborne disease costs. Gastroenterology 136: 1851-1862.

Bruce-Grey-Owen Sound Health Unit (2000). The investigative report of the Walkerton outbreak of waterborne gastro-enteritis. Owen Sound, Ont. Canada: Bruce-Grey-Owen Sound Health Unit. Cressey PJ, Lake R (2007). Risk ranking: Estimates of the burden of foodborne disease for New Zealand. Institute of Environmental Science & Research Limited; Christchurch Science Center.

Dundas S, Murphy J, Soutar RL, Jones GA, Hutchinson SJ, Todd WT. Effectiveness of therapeutic plasma exchange in the 1996 Lanarkshire Escherichia coli O157:H7 outbreak. Lancet. 1999;354: 1327–30

Frenzen PD, Drake A, Angulo FJ (2005). Economic cost of illness due to Escherichia coli O157 infections in the United States. J Food Prot 68 (12): 2623-30.

Haagsma J.A., P.D. Siersema, N.J. De Wit, A.H. Havelaar (2010). Disease burden of post-infectious irritable bowel syndrome in the Netherlands. Epidemiol Infect 138(11): 1650-6.

Haagsma JA, Maertens de Noordhout C, Polinder S, Vos T, Havelaar AH, Cassini A, Devleesschauwer B, Kretzschmar ME, Speybroeck N, Salomon JA. Assessing disability weights based on the responses of 30,660 people from four European countries. Population Health Metrics 2015; 13: 10

Havelaar AH, van Duynhoven YT, Nauta MJ, Bouwknegt M, Heuvelink AE, de Wit GA, et al. (2003) Disease burden in The Netherlands due to infections with Shiga toxin-producing Escherichia coli O157. Epidemiol Infect 132(3): 467-84.

Marshall JK, Thabane M, Garg AX, Clark WF, Moayyedi P, Collins SM. Walkerton Health Study Investigators (2010). Eight year prognosis of post- infectious irritable bowel syndrome following waterborne bacterial dysentery. Gut;59: 605-611.

McPherson M, Kirk MD, Raupach J, Combs B, Butler JR. (2011) Economic costs of Shiga toxin-producing Escherichia coli infection in Australia. Foodborne Pathog Dis 8 (1): 55-62.

Mead PS, Slutsker L, Dietz V, McCaig LF, Bresee JS, Shapiro C et al. (1999). Food-related illness and death in the United States. Emerg Infect Dis 5(5): 607-625.

Michel P, Wilson JB, Martin SW, Clarke RC, McEwen SA, Gyles CL (2000). Estimation of the under-reporting rate for surveillance of Escherichia coli O157:H7 cases in Ontario, Canada. Epidemiol Infect 125: 35-45.

Oxford Medical Dictionary (2003). Oxford, Oxford University Press; 3rd edition.

Tariq L, Haagsma J, Havelaar A (2011). Cost of Illness and Disease Burden in The Netherlands Due to Infections with Shiga Toxin-Producing Escherichia coli O157. J Food Prot 74 (4): 545-52.

Ternhag A, Törner A, Svensson A, Ekdahl K, Giesecke J (2008). Short- and long-term effects of bacterial gastrointestinal infections. Emerg Infect Dis 14 (1): 143-8.

Thabane M, Simunovic M, Akhtar-Danesh N, Marshall JK (2009). Development and Validation of a Risk Score for Post-Infectious Irritable Bowel Syndrome. Am J Gastroenterol; 104:2267–2274.

Syphilis

Syphilis is a complex, systemic disease caused by the spirochaete Treponema pallidum (T. pallidum), a gram-negative bacterium. Syphilis is preventable and curable with effective and inexpensive antibiotics. The only known natural hosts are humans, and the pathogen is not able to survive outside its host due to limited metabolic capacities to synthetise its own bio-nutrients. Syphilis spirochetes, like other treponemas, cannot be cultivated in vitro. The primary mode of syphilis transmission is by sexual contact (acquired syphilis). Vertical transmission from infected mother to child is possible (congenital syphilis), either in utero (transfer across the placenta) or through contact with an active genital lesion during delivery (Singh, 1999). Untreated syphilis can adversely affect pregnancy outcomes, resulting in spontaneous abortion, stillbirth, premature delivery, or perinatal death. Prematurity and low birth weight have been observed in 10 to 40% of infants born to untreated mothers (Salojee, 2004). The rate of infection through sexual intercourse with an infected partner has been estimated at about 50% (Ficarra & Carlos, 2009).

In Europe and other high-income countries, the transmission via blood or blood products is rare because of the low incidence rates of the disease and improved blood screening and blood donor testing for syphilis (Tramont, 2005).

Only 50% of those infected with T. pallidum will develop symptoms (RKI, 2003). Primary syphilis lasts from two weeks to six months (Baughn & Musher, 2005). Secondary syphilis may last two to eight weeks (Zetola, 2007). Early latent disease is diagnosed less than one year after infection (WHO, 2003; MMWR, 2010). Late latent syphilis infection is diagnosed after more than one year (WHO, 2003; MMWR, 2010).

Health outcomes and states associated with syphilis infection in adults

The incubation period for primary syphilis is on average three weeks (10–90 days) and depends on bacterial load, the immune status of the infected person and the existence of other co-morbid conditions (e.g. HIV/AIDS) (Weir & Fisman, 2002; Krause, 2006). Acquired syphilis is divided into primary, secondary, latent and tertiary syphilis. The disease can also be divided into early and late syphilis. Early syphilis implies the primary, secondary and early latent stages. Late syphilis refers to late latent syphilis and tertiary syphilis (Hook, 1992).

Primary syphilis is characterised by an ulcer and/or chancre at the site of infection or inoculation. This primary lesion appears about three weeks after exposure as an indurated, painless ulcer and may not be clinically evident (i.e. it may be in the rectum or the cervix). Invasion of the bloodstream precedes the initial lesion. In 50% of cases, the chancre is accompanied by regional lymphadenopathy (a firm, non-tender satellite lymph node) (Genc, 2000). After three to six weeks the chancre begins to involute, but may persist in the secondary stage in 15–30% of those infected (Zetola, 2007; Krause, 2006; Parish, 2000).

After 2–12 weeks on average (sometimes 12 months) the untreated infection may progress to secondary syphilis caused by the haematogenic spread and lymphatic dissemination of T. pallidum in the body. The time at which the secondary lesions manifest depends on the bacterial load of the treponeme and the immune response of the host (Baughn, 2005). This stage is characterised by skin rash, condylomata lata (5–22% of patients), mucocutaneous lesions, alopecia (5–7% of patients), and generalised lymphadenopathy (Ficarra & Carlos, 2009). A patient with secondary syphilis may have one, several or all of the signs of the secondary stage. Since each of the signs may also be associated with other diseases, none are specific to syphilis. Neurological involvement in secondary syphilis (known as syphilitic meningitis) can occur, especially in HIV co-infected patients (Marra, 2004). The manifestations of secondary syphilis last two to eight weeks and then may resolve, even without treatment (Zetola, 2007).

After resolution of the secondary manifestations, around one-third of untreated patients will enter into a latent phase. The latent or asymptomatic stage of syphilis is defined as the period from disappearance of the secondary manifestations until therapeutic cure or development of late sequelae. An infection without any clinical symptoms lasting less than one year is referred to as early latent syphilis, whereas an infection of more than one year's duration without clinical evidence of treponemal infection is referred to as late latent syphilis (WHO, 2003). The definitions of duration may vary across countries. The early latent period corresponds to the highest risk of transmission.

Tertiary syphilis may appear after a long period of untreated syphilis (5–20 years after initial infection) and its manifestations can include gummas (late benign syphilis), cardiovascular symptoms and neurosyphilis (Hutto, 2001). In developed countries gummas and cardiovascular symptoms are rarely seen and most of the late sequelae are associated with neuro-syphilis. The timescale for development of neuro-syphilis may vary from a period of one or two years to more than 30 years after primary syphilis, and may involve 5–10% of untreated patients (Gjestland, 1955). It is characterised by the involvement of the central nervous system which leads to a number of different syndromes, included in the health outcome 'neuro-syphilis' in our model. In two thirds of patients the infection will not progress to late complications (Mindel, 2000).

Health outcomes and states associated with congenital syphilis infection

Postnatal manifestations of congenital syphilis are divided into early and late stages. Clinical manifestations occurring within the first two years after birth (<2 years) are categorised as early congenital syphilis. Clinical manifestations which occur later than two years after birth are late congenital syphilis (Parish, 2000). For the underlying model, and due to scarce data, only congenital syphilis was included, with no distinction between early or late.

Outcome tree parameters

Due to the high complexity of syphilis outcomes and for reasons of feasibility, the outcome tree for the adult population was split into symptomatic and asymptomatic infections at the first level of disaggregation. The natural course of syphilis was subdivided into the three main disease states: primary, secondary and neuro-syphilis. The focus was on neuro-syphilis because other forms of late syphilis sequelae are very rare in developed countries.

The percentage of asymptomatic cases was estimated at 50% (RKI 2003, Singh, 1999; Ficarra, 2009; Genc, 2000; Parish, 2000). Gerbase and colleagues presented treatment rates of 85% for both primary and secondary symptomatic syphilis cases in regions with established market economies (Gerbase, 2000). As a result of high cure rates (up to 100%), it was estimated that about 85% of all primary syphilis cases are treated and subsequently cured. The remaining 15% of untreated symptomatic cases have a 30–50% possibility of developing secondary syphilis, resulting in a probability of 4.5–7.5% that they will develop secondary syphilis, after having had primary syphilis (Singh, 1999; Weir & Fisman, 2002; Krause, 2006; Gerbase, 2000; Golden, 2003). In asymptomatic primary syphilis the primary chancre is not visible and will generally go unnoticed, meaning that it is less likely to be treated, hence the greater risk of progression to secondary syphilis (30–50%).

Furthermore, 85% of symptomatic secondary syphilis cases are treated and again, as a result of the high cure rates (around 100%), the remaining 15% of untreated cases have a probability of 5–12% of developing neuro-syphilis. Thus, the proportion of people developing neuro- syphilis from preceding secondary syphilis was set at 0.75–1.88% (Tramont, 2005; Zetola, 2007; Gerbase, 2000; Goldmeier & Guallar, 2003).

The probability of dying due to syphilis before reaching the late (tertiary) phase of the disease is very low and there is little evidence of a case fatality ratio associated with syphilis in general, or neurosyphilis in particular, within Europe. We assumed that neurosyphilis in Europe is successfully treated; although with a possibility of developing permanent disabilities for which it was impossible to define the impact due to lack of data. Antibiotic treatment is highly effective and is therefore not associated with a case fatality ratio.

For infants the main outcome is congenital infection with a probability of 20% (2–64%) for an infected child (Singh, 1999; Salojee, 2004; Genc, 2000; Gerbase, 2000). In total, 1% of all children with congenital infection die (Gerbase, 2000).

Model input summary

Table 1. Transition probabilities and distributions used in the outcome tree

Health outcome (Health state)	Transition probability	Source/assumption
Acquired		
Primary syphilis from infection	50%	RKI, 2003
Secondary syphilis from asymptomatic infection	30–50%	Singh, 1999; Weir & Fisman, 2002; Gerbase, 2000; Golden 2003
Secondary syphilis from symptomatic infection	4.5–7.5%	Singh, 1999; Weir & Fisman, 2002; Gerbase, 2000; Golden 2003
Neuro-syphilis	0.75–1.88%	Tramont, 2005; Zetola, 2007; Krause, 2006; Weir&Fisman, 2002; Gerbase, 2000; Golden, 2003; Goldmeier, 2003
Fatal cases due to neurosyphilis	0%	Assuming all cases are identified and treated, and no treatment failure
Congenital		
Symptomatic infection	20% (2–64%)	Singh, 1999; Saloojee, 2004; Genc & Ledger, 2000

Fatal cases due to congenital infection	1%	Genc & Ledger, 2000

Table 2. Disability weights and duration

Health outcome (Health state)	Disability Weight (DW) (Haagsma, 2015)		Duration	
	DW	Label	In years	Source
Acquired				
Primary syphilis	0.007 (0.005-0.01)	Infectious disease, acute episode, mild	0.121-0.5	Baughn & Musher, 2005
Latency period (from primary to secondary)	0		0.23 (0.038-1)	Baughn, 2005
Secondary syphilis	0.125 (0.104-0.152)	Infectious disease, acute episode, severe	0.038-0.153	Zetola, 2007
Latency period (from secondary to neurosyphilis)	0		4.77-19.77	Hutto, 2001
Neurosyphilis	0.407 (0.36-0.46)	Motor plus cognitive impairments, severe	0.027-0.038	Workowski, 2010 Assuming 10–14 days of treatment
Congenital				
Symptomatic infection	0.125 (0.104-0.152)	Infectious disease, acute episode, severe	3	Kwong, 2010

References

Baughn RE, Musher DM. Secondary syphilitic lesions. Clin Microbiol Rev 2005, 18:205-216.

Bremer, V, Marcus U, Hamouda O (2012). Syphilis on the rise again in Germany--results from surveillance data for 2011. Euro Surveill 17(29).

[SW1] Doherty L, Fenton KA, Jones J, Paine TC, Higgins SP, Williams D, et al. Syphilis: old problem, new strategy. BMJ 2002, 325:153-156.

Ficarra G, Carlos R. Syphilis: the renaissance of an old disease with oral implications. Head Neck Pathol 2009, 3:195-206.

Genc M, Ledger WJ. Syphilis in pregnancy. Sex Transm Infect 2000, 76:73-79.

Gerbase A, Stein C, Levison J, Htun Y. Global burden of sexually transmitted diseases (excluding HIV) in the year 2000. In: Global burden of sexually transmitted diseases (excluding HIV) in the year 2000. Geneva; World Health Organization; 2000.

Gjestland T. The Oslo study of untreated syphilis; an epidemiologic investigation of the natural course of the syphilitic infection based upon a re-study of the Boeck-Bruusgaard material. Acta Derm Venereol Suppl (Stockh) 1955, 35:3-368; Annex I-LVI.

Golden MR, Marra CM, Holmes KK. Update on syphilis: resurgence of an old problem. JAMA 2003, 290:1510-1514.

Goldmeier D, Guallar C. Syphilis: an update. Clin Med 2003, 3:209-211.

Haagsma JA, Maertens de Noordhout C, Polinder S, Vos T, Havelaar AH, Cassini A, Devleesschauwer B, Kretzschmar ME, Speybroeck N, Salomon JA. Assessing disability weights based on the responses of 30,660 people from four European countries. Population Health Metrics 2015; 13: 10

Hook EW, 3rd, Marra CM. Acquired syphilis in adults. N Engl J Med 1992, 326:1060-1069.

Hutto B. Syphilis in clinical psychiatry: a review. Psychosomatics 2001, 42:453-460.

Krause W. [Syphilis]. Urologe A 2006, 45:1494-1500.

Kwong JC, Crowcroft NS, Campitelli MA, Ratanasingham S, Daneman N, Deeks SL, et al. Ontario Burden of Infectious Disease Study. OAHPP/ECES Report. City: Ontario Agency for Health Protection and Promotion and the Institute for Clinical Evaluative Sciences; 2010.

Limb M. New scheme aims to eradicate congenital syphilis in three years. BMJ 2012, 344:e1616.

Marra C. Neurosyphilis. Curr Neurol Neurosci Rep 2004, 4:435-440.

Mindel A, Estcourt C. Syphilis. In: Sexually transmitted diseases: Vaccines, Prevention and Control. Edited by Stanberry LR, Bernstein DI: Academic Press, San Diego, San Francisco, New York, Boston, London, Sydney, Tokyo; 2000

Murray CJL, Lopez AD, World Health Organization, World Bank, Harvard School of Public Health. Global health statistics: a compendium of incidence, prevalence and mortality estimates for over 200 conditions. Geneva: World Health Organization; 1996.

Murray CJL, Lopez AD. The Global Burden of Disease - A comprehensive assessment of mortality and disability from diseases, injuries, and risk factors in 1990 and projected to 2020. Cambridge: Harvard School of Public Health on behalf of the World Health Organization and the World Bank; 1996.

Parish JL. Treponemal infections in the pediatric population. Clin Dermatol 2000, 18:687-700.

Robert Koch Institut. RKI-Ratgeber Infektionskrankheiten - Merkblätter für Ärzte: Syphilis (Lues). Epidemiologisches Bulletin 2003, 30:229-233.

Saloojee H, Velaphi S, Goga Y, Afadapa N, Steen R, Lincetto O. The prevention and management of congenital syphilis: an overview and recommendations. Bull World Health Organ 2004, 82:424-430.

Singh AE, Romanowski B. Syphilis: review with emphasis on clinical, epidemiologic, and some biologic features. Clin Microbiol Rev 1999, 12:187- 209.

Tramont EC. Treponema pallidum (Syphilis). In: Principles and practice of infectious diseases. Volume Volume 2, 6th Edition. Edited by Mandel GL, Bennet JE, Dolin R. Pennsylvania: Elsevier Inc. 2005: 2768-2785

Weir E, Fisman D. Syphilis: have we dropped the ball? CMAJ 2002, 167:1267-1268.

Workowski KA, Berman SM; Centers for Disease Control and Prevention. Sexually transmitted diseases treatment guidelines, 2010. MMWR Recomm Rep. 2010;59(RR-12):1-110.

World Health Organization. Dept. of Reproductive Health and Research. Guidelines for the management of sexually transmitted infections. Geneva: World Health Organization; 2003.

Zetola NM, Engelman J, Jensen TP, Klausner JD. Syphilis in the United States: an update for clinicians with an emphasis on HIV coinfection. Mayo Clin Proc 2007, 82:1091-1102.

Tetanus

Tetanus is an acute and often fatal disease induced by the tetanospasmin, an exotoxin produced by Clostridium tetani, a gram-positive anaerobic bacillus (Bleck, 2005; CDC, 2012). C. tetani is sensitive to heat and not viable under aerobic conditions (CDC, 2012). In contrast, the spores of C. tetani are resistant to heat and antiseptics and are widely present in soil and in the intestines and faeces of animals (e.g. horses, sheep and dogs). Tetanus is primarily contracted via contaminated wounds and is not contagious. Effective vaccination programmes significantly reduced the burden of tetanus. Globally around 800 000 to 1 000 000 people die of tetanus each year (Dietz, 1996). Around 90% of all deaths occur in developing countries which are largely affected by tetanus and especially neonatal and maternal tetanus. In developed countries, high-risk groups, such as unvaccinated persons and injecting drug users, are prone to infection with C. tetani (CDC, 2012). The proportion of asymptomatic/subclinical infections is unknown but it can be assumed that cases of tetanus are symptomatic in nearly 100% of those infected. The first symptoms of tetanus appear after an average incubation period of eight days (range: 3–21 days) (CDC, 2012). The duration of the symptomatic disease for generalised, localised and cephalic tetanus is two to three weeks (CDC, 2012).

Health outcomes/states associated with tetanus infection

The clinical features of acute tetanus infections can be subdivided into three health states that are observed in developed countries. A fourth type, tetanus neonatorum is a specific form of generalised tetanus that affects neonates and is mostly observed in the developing world with a high case fatality of up to 90% (Roper, 2007). As neonatal tetanus has been eliminated in Europe this health outcome is not considered in our outcome tree and model.

The distribution of the three health states is set according to the observed risk of developing the different forms of acute infection in USA (Bardenheier, 1998): 81% were generalised; 13% localised and 6% cephalic.

Localised tetanus

Localised tetanus is an uncommon health state of tetanus. Localised tetanus appears as a persistent contraction of muscles in the injured area, commonly preceding generalised tetanus, and lasts around two to three 3 weeks (CDC, 2012).

Generalised tetanus

The most common health state of tetanus infection is generalised tetanus. The probability of developing generalised tetanus after initial infection is around 80% (CDC, 2012; Bardenheier, 1998; Guilfoile, 2008). The symptoms of generalised tetanus are trismus or lockjaw in the early stages, developing into stiffness of the neck, difficulty in swallowing and rigidity of abdominal muscles. Further, unspecific symptoms such as elevated temperature, sweating, elevated blood pressure, and episodic rapid heart rate may occur. Generalised tetanus can last for 3-4 weeks and full recovery may take several months (CDC, 2012).

Cephalic tetanus

Cephalic tetanus is another uncommon health state involving the cranial nerves. The same duration has been assumed for this health state as for localised tetanus: 2–3 weeks.

Further complications and case fatality proportion

In cases of cephalic tetanus otitis media may occur (CDC, 2012). Long-term sequelae/disabilities from tetanus are not reported in the literature.

The overall mortality rate of tetanus ranges from 28/100 000 in developing countries to 0.1/100 000 in developed countries such as the USA. The case fatality proportion ranges between 5 and 55% (Guilfoile, 2008; Brook, 2004; Cook, 2001; Farrar, 2000; Kanchanapongkul, 2001; Miranda-Filho Dde 2004; Saltoglu, 2004; Sanford, 1995; Thwaites, 2004; Trujillo, 1987). Mortality from tetanus is clearly dependent on age, immune status and vaccination. People over 60 years of age or unvaccinated persons have an elevated lethality of 18 and 22%, respectively. In the model, the mortality rate following symptomatic cases was set at 11% (CDC, 2012; Bardenheier, 1998).

Model input summary

Table 1. Transition probabilities used in the outcome tree

Health outcome<br>(Health state)	Distribution of health states in health outcome	Transition probability	Source/assumption
Symptomatic infection<br>(Localised tetanus)<br>(Generalised tetanus)<br>(Cephalic tetanus)	 13%<br>81%<br>6%		Bardenheier, 1998
Fatal cases		11%	CDC, 2012<br>Bardenheier, 1998

Table 2. Disability weights and duration

Health outcome (Health state)	Disability Weight (DW) (Haagsma, 2015)		Duration	
	DW	Label	In years	Source
Symptomatic infection (Generalised tetanus)	0.421 (0.377-0.477)	Motor impairment, severe	0.06-0.08	CDC, 2012
(Localised tetanus)	0.011 (0.008-0.014)	Motor impairment, mild	0.04-0.06	CDC, 2012
(Cephalic tetanus)	0.053 (0.042-0.064)	Motor impairment, moderate	0.04-0.06	CDC, 2012 Assumed same as for localised

References

Bardenheier B, Prevots DR, Khetsuriani N, Wharton M. Tetanus surveillance - United States, 1995-1997. MMWR CDC Surveill Summ 1998, 47:1- 13.

Bleck TP. Clostridium tetani (Tetanus). In Principles and practice of infectious diseases. Volume 2. Edited by Mandell GL, Bennett JE, Dolin R. Pennsylvania: Elsevier Inc.; 2005:2817–2822

Brook I. Tetanus in children. Pediatr Emerg Care 2004, 20:48-51.

Centers for Disease Control and Prevention: Tetanus. In Epidemiology and Prevention of Vaccine-Preventable Diseases. Edited by Atkinson W, Wolfe S, Hamborsky J. Washington DC: Public Health Foundation; 2012:291-300

Cook TM, Protheroe RT, Handel JM. Tetanus: a review of the literature. Br J Anaesth 2001, 87:477-487.

Dietz V, Milstien JB, van Loon F, Cochi S, Bennett J: Performance and potency of tetanus toxoid: implications for eliminating neonatal tetanus. Bull World Health Organ 1996, 74:619-628.

Farrar JJ, Yen LM, Cook T, Fairweather N, Binh N, Parry J, et al. Tetanus. J Neurol Neurosurg Psychiatry 2000, 69:292-301.

Guilfoile PG. Tetanus. New York: Infobase Publishing; Chelsea House; 2008.

Haagsma JA, Maertens de Noordhout C, Polinder S, Vos T, Havelaar AH, Cassini A, Devleesschauwer B, Kretzschmar ME, Speybroeck N, Salomon JA. Assessing disability weights based on the responses of 30,660 people from four European countries. Population Health Metrics 2015; 13: 10

Hsu SS, Groleau G. Tetanus in the emergency department: a current review. J Emerg Med 2001, 20:357-365.

Kanchanapongkul J. Tetanus in adults: a review of 85 cases at Chon Buri Hospital. J Med Assoc Thai 2001, 84:494-499.

Miranda-Filho Dde B, Ximenes RA, Barone AA, Vaz VL, Vieira AG, Albuquerque VM. Randomised controlled trial of tetanus treatment with anti- tetanus immunoglobulin by the intrathecal or intramuscular route. BMJ 2004, 328:615.

Murray CJL, Lopez AD. World Health Organization., World Bank., Harvard School of Public Health.: Global health statistics : a compendium of incidence, prevalence and mortality estimates for over 200 conditions. Geneva: World Health Organization; 1996.

Roper MH, Vandelaer JH, Gasse FL. Maternal and neonatal tetanus. Lancet 2007, 370:1947-1959.

Saltoglu N, Tasova Y, Midikli D, Burgut R, Dundar IH. Prognostic factors affecting deaths from adult tetanus. Clin Microbiol Infect 2004, 10:229- 233.

Sanford JP. Tetanus: forgotten but not gone. Waltham, MA, : Massachusetts Medical Society; 1995.

Thwaites CL, Yen LM, Nga NT, Parry J, Binh NT, Loan HT et al. Impact of improved vaccination programme and intensive care facilities on incidence and outcome of tetanus in southern Vietnam, 1993-2002. Trans R Soc Trop Med Hyg 2004, 98:671-677.

Trujillo MH, Castillo A, Espana J, Manzo A, Zerpa R. Impact of intensive care management on the prognosis of tetanus. Analysis of 641 cases. Chest 1987, 92:63-65.

Tick-borne encephalitis (TBE)

Most cases of tick-borne encephalitis (TBE) in Europe involve a biphasic presentation of the disease with fever during the first phase and neurological disorders during the second phase (Gubler, 2007). Severity of tick-borne encephalitis increases with age. TBE in children (<14 years) usually runs a more benign course (Mickiene, 2002; Kaiser, 1999). The proportion of asymptomatic cases is 66–80% (Gustafson, 1992). To calculate the burden of disease we assume that asymptomatic patients do not develop sequelae and are not included in the burden estimation.

The subtype considered is the Central European encephalitis subtype (Western tick-borne encephalitis virus) which is the dominant one in Europe. Another subtype does occur, the Russian spring-summer encephalitis subtype, however this occurs less in EU Member States and is not considered in the outcome tree.

The symptomatic infection (viraemic phase) begins after an average incubation period of eight days (range 4–28 days) (Kaiser, 1999). Symptoms of this first phase include fever, muscle pain, fatigue and headache (Gunther, 1997; Kaiser, 1999), normally lasting for five (2–7) days (Gubler, 2007).

Meningoencephalitic phase

After a symptom-free period, usually less than two weeks, a meningoencephalitic second phase occurs in 20–30% of symptomatic patients (Gustafson, 1990; 1992; Kiffner, 2010). The duration of the meningoencephalitic phase is set to 15 days (10–70) (Kaiser, 1999). The case fatality proportion of the meningoencephalitic phase is set to 0.75% (Mickiene, 2002).

Paralysis and residual paresis

Following the meningoencephalitic phase there is a latency period of six days (range 1–17 days), after which paralysis occurs in an estimated 11% of patients (Gunther, 1997). The duration is set to 3–10 days (Kaiser, 1999). Overall, 56% of paralytic patients are at risk of developing lifelong residual paresis (partial loss of or impaired movement) (Gunther, 1997).

Post-encephalitic TBE syndrome

A long-term post-encephalitic TBE syndrome, with symptoms including cognitive or neuropsychiatric complaints, balance disorders, headache, dysphasia, hearing defects and spinal paralysis, has been reported in 39–46% of meningoencephalitic patients (Gunther, 1997; Mickiene, 2002). The duration of post-encephalitic TBE syndrome is set to one year ('Post TBE syndrome existed after 1 year in more than one third of the patients' Gunther, 1997).

Lifelong chronic sequelae can persist in 35.7% (Haglund & Gunther, 2003) to 38.8% of post-encephalitic syndrome patients (Gunther, 1997: 'persisting symptoms at 12 months in 33/85 patients'). Males are affected twice as much as females and 12% of patients with post-encephalitic TBE syndrome were under 14 years of age (Kaiser, 1999). However, the association between gender, age and severity still needs more research and is not considered in the outcome tree.

Model input summary

Table 1. Transition probabilities used in the outcome tree

Health outcome (Health state)	Distribution of health states in health outcome	Transition probability	Source/assumption
Symptomatic infection		20–34%	Gustafson, 1992
Meningoencephalitic phase		20–30%	Gustafson, 1990, 1992; Kiffner, 2010
Paralysis		11%	Kaiser, 1999; Gunther, 1997
Residual paresis		56%	Gunther, 1997
Post-encephalitic TBE syndrome		39–46%	Gunther 1997; Mickiene, 2002
Chronic post-encephalitic TBE syndrome		35.7–38.8%	Haglund & Gunther, 2003<br>Gunther, 1997
Fatal cases following meningoencephalitic phase		0.75%	Mickiene, 2002

Table 2. Disability weights and duration

Health outcome (Health state)	Disability Weight (DW) (Haagsma, 2015)		Duration	
	DW	Label	In years	Source

Symptomatic infection	0.051 (0.039-0.06)	Infectious disease, acute episode, moderate	0.014 (0.005-0.019)	Gubler, 2007
Meningoencephalitic phase	0.447 (0.391-0.501)	Encephalopathy - severe	0.041 (0.027-0.192)	Kaiser, 1999
Paralysis	0.526 (0.469-0.586)	Spinal cord lesion at neck level (treated)	0.0137	Kaiser, 1999
Residual paresis	0.056 (0.044-0.067)	Motor plus cognitive impairments, mild	Remaining life expectancy	Remaining life expectancy
Post-encephalitic TBE syndrome	0.202 (0.167-0.242)	Motor plus cognitive impairments, moderate	1	Gunther, 1997
Chronic post-encephalitic TBE syndrome	0.056 (0.044-0.067)	Motor plus cognitive impairments, mild	Remaining life expectancy	Remaining life expectancy

References

Gubler JD, Kuno G, Markoff L. 2007. Flaviviruses. In: Knipe, D.M., Howley, P.M. (Eds.), Fields Virology 5th ed., vol. 1. Lippincott Williams & Wilkins, London, New York, Tokyo, pp. 1043–1125.

Gustafson R, Svenungsson B, Forsgren M, Gardulf A, Granström M. Two-year survey of the incidence of Lyme borreliosis and tick-borne encephalitis in a high-risk population in Sweden. European Journal of Clinical Microbiology and Infectious Diseases, 1992 Oct;11(10):894-900. PMID: 1486884

Gustafson R, Svenungsson B, Gardulf A, Stiernstedt G, Forsgren M. Prevalence of tick-borne encephalitis and Lyme borreliosis in a defined Swedish population. Scandinavian Journal of Infectious Diseases, 1990;22(3):297-306. PMID: 2371545

Günther G, Haglund M, Lindquist L, Forsgren M, Sköldenberg B. Tick-bone encephalitis in Sweden in relation to aseptic meningo-encephalitis of other etiology: a prospective study of clinical course and outcome. Journal of Neurology, 1997 Apr;244(4):230-8. PMID: 9112591

Haglund M, Günther G. Tick-borne encephalitis--pathogenesis, clinical course and long-term follow-up. Vaccine, 2003 Apr 1;21 Suppl 1:S11-8. PMID: 12628810

Haagsma JA, Maertens de Noordhout C, Polinder S, Vos T, Havelaar AH, Cassini A, Devleesschauwer B, Kretzschmar ME, Speybroeck N, Salomon JA. Assessing disability weights based on the responses of 30,660 people from four European countries. Population Health Metrics 2015; 13: 10

Kaiser R. The clinical and epidemiological profile of tick-borne encephalitis in southern Germany 1994-98: a prospective study of 656 patients. Brain 1999; 122 (Pt 11): 2067-78. PMID: 10545392

Kiffner C, Zucchini W, Schomaker P, Vor T, Hagedorn P, Niedrig M, et al. Determinants of tick-borne encephalitis in counties of southern Germany, 2001-2008. International Journal of Health Geography, 2010 Aug 13;9:42. PMID: 20707897

Mickiene A, Laiskonis A, Gunther G, et al. Tick-borne encephalitis in an area of high endemicity in Lithuania: disease severity and long-term prognosis. Clin Infect Dis 2002; 35 (6): 650-8 PMID: 12203160

Toxoplasmosis

Acquired toxoplasmosis

In Europe, most cases of acquired toxoplasmosis are asymptomatic and self-limiting (Rorman, 2006). Acquired toxoplasmosis will lead to symptomatic illness in approximately 10–20% of infected cases (Montoya, 2000). It is estimated that 4.67% (0–15.3%) of symptomatic cases will manifest more severe symptoms and approximately 2% (0–4.67%) are at risk of developing life-long sequelae relative to chorioretinitis. However, it is unclear if this risk is attributable mainly to more severe, symptomatic infections or all infections (Kemmeren, 2006). All other symptomatic cases will manifest minor symptoms, such as fever and lymphoadenopathy (Rorman, 2006; Anand, 2012).

Mortality due to acquired toxoplasmosis is extremely rare and occurs in immunocompromised patients. It has therefore been decided to exclude fatal cases from the outcome tree of acquired toxoplasmosis.

Toxoplasmosis may also play a role in the development of psychiatric disorders, such as schizophrenia and bipolar depression (Torrey, 2003; Henriquez, 2009; Brown, 2010). However, insight into causality is still insufficient and these sequelae are not included in the model.

Congenital toxoplasmosis

Vertical transmission from a recently infected pregnant woman to her foetus may lead to congenital toxoplasmosis. Infections occurring during the first and second trimester of pregnancy may result in foetal loss (1.5–1.7% of seroconverting pregnant women, Havelaar 2007) or stillbirth (although neither of these are included in the present burden estimation) and symptoms in newborn infants are generally more severe.

However, if the infection occurs in the third trimester the disease manifestation is generally subclinical. When present, symptoms vary from a triad including chorioretinitis, intracranial calcification and hydrocephalus to abnormalities of the central nervous system. These complications may lead to life-long sequelae, including subclinical congenital toxoplasmosis which could increase the risk of developing chorioretinitis later in life. Death can occur in a small proportion of infections. Other symptoms are very rare and have not been considered in this model.

Several studies have described clinical manifestations and follow-up of newborns infected with toxoplasmosis: 89% of children were asymptomatic at birth (16% of them developed chorioretinitis later in life) (Berrebi, 2010), 85% had no clinical findings at birth (Lebech, 1999) and 74.5% were asymptomatic at birth (Schmidt, 2006). Therefore, the proportion of asymptomatic infections out of the total congenital toxoplasmosis infections is 11–25%.

Asymptomatic congenital toxoplasmosis-infected infants have a 2% (1–3%) per year risk of developing chorioretinitis at a later age. The studies followed cases of asymptomatic congenital toxoplasmosis for 10–14 years (Havelaar, 2007).

Based on an extensive literature review, Havelaar et al. (Haavelar, 2007) estimated the risk of developing permanent disabilities related to congenital toxoplasmosis infections. We applied the same estimates to our model for all infections: 13% (12–15%) will develop permanent disabilities due to complications related to chorioretinitis, 11% (8–12%) to intracranial calcification, 3% (1-6%) to the central nervous system and 2% (1–3%) to hydrocephalus.

Model input summary

Table 1. Percentages used in the outcome tree

Health outcome<br>(Health state)	Distribution of health<br>states in health outcome	Transition probability	Source/assumption

Acquired toxoplasmosis			
Symptomatic infections:<br>(Uncomplicated)<br>(Complicated)	 Remaining cases<br>4.67% (0–15.3%)	10–20%	Kemmeren, 2006
Chorioretinitis following symptomatic infection		2% (0–4.67%)	Kemmeren, 2006
Congenital toxoplasmosis			
Symptomatic infections:<br>(Asymptomatic)<br>(Symptomatic)	75–89%<br>Remaining cases		Berrebi, 2010<br>Lebech, 1999<br>Schmidt, 2006
Permanent disability due to chorioretinitis after the first year following asymptomatic infection		2% (1-3%) per year (ATP) for 10–14 years	Havelaar, 2007<br>Starting one year after infection up to the age of 10–14 years<br>ATP: Annual Transition Probability

Permanent disability due to chorioretinitis within first year		13% (12–15%)	Havelaar, 2007
Permanent disability due to intracranial calcification		11% (8–12%)	Havelaar, 2007
Permanent disability due to hydrocephalus		2% (1–3%)	Havelaar, 2007
Permanent disability due to CNS abnormalities		3% (1–6%)	Havelaar, 2007
Fatal cases		0.7% (0.4–1.2%)	Havelaar, 2007

Table 2. Disability weights and duration

Health outcome (Health state)	Disability Weight (DW) (Haagsma, 2015)		Duration	
	DW	Label	In years	Source
Acquired toxoplasmosis				
Acquired toxoplasmosis (Uncomplicated) (Complicated)	0.007 (0.005–0.01) 0.125 (0.104–0.152)	Infectious disease, acute episode, mild Infectious disease, acute episode, severe	0.04	Kemmeren, 2006
Congenital toxoplasmosis				
Congenital toxoplasmosis (Asymptomatic) (Symptomatic)	0 0.125 (0.104–0.152)	Infectious disease, acute episode, mild Infectious disease, acute episode, severe	1 0.167	Assuming chorioretinitis starts after one year Melse, 2000
Permanent disability due to chorioretinitis following asymptomatic infections	0.015 (0.011–0.019)	Conjunctivitis without corneal scar	rem life exp.	Havelaar, 2007
Permanent disability due to	0.015 (0.011–0.019)	Conjunctivitis without corneal	rem life exp.	Havelaar, 2007

chorioretinitis following symptomatic infections		scar		
Permanent disability due to intracranial calcification	0.044–0.087	Intellectual disability/mental retardation, from mild to moderate	rem life exp.	Havelaar, 2007
Permanent disability due to hydrocephalus	0.044–0.188	Intellectual disability/mental retardation, from mild to severe	rem life exp.	Havelaar, 2007
Permanent disability due to CNS abnormalities	0.056–0.407	Motor plus cognitive impairments, from mild to severe	rem life exp.	Havelaar, 2007
Permanent disability due to chorioretinitis	0.015 (0.011–0.019)	Conjunctivitis without corneal scar	rem life exp.	Kemmeren, 2006

References

Anand R, Jones CW, Ricks JH, Sofarelli TA, Hale DC. Acute primary toxoplasmosis in travelers returning from endemic countries. J Travel Med. 2012; 19(1): 57-60.

Berrebi A, Assouline C, Bessieres MH, Lathiere M, Cassaing S, Minville V, et al. Long-term outcome of children with congenital toxoplasmosis. American journal of obstetrics and gynecology. 2010; 203(6): 552 e1-6.

Brown AS, Derkits EJ. Prenatal infection and schizophrenia: a review of epidemiologic and translational studies. Am J Psychiatry. 2010; 167(3): 261-80.

Burnett AJ, Shortt SG, Isaac-Renton J, King A, Werker D, Bowie WR. Multiple cases of acquired toxoplasmosis retinitis presenting in an outbreak. Ophthalmology. 1998; 105(6): 1032-7.

Haagsma JA, Maertens de Noordhout C, Polinder S, Vos T, Havelaar AH, Cassini A, Devleesschauwer B, Kretzschmar ME, Speybroeck N, Salomon JA. Assessing disability weights based on the responses of 30,660 people from four European countries. Population Health Metrics 2015; 13: 10

Havelaar AH, Kemmeren JM, Kortbeek LM. Disease burden of congenital toxoplasmosis. Clin Infect Dis. 2007; 44(11): 1467-74.

Henriquez SA, Brett R, Alexander J, Pratt J, Roberts CW. Neuropsychiatric disease and Toxoplasma gondii infection. Neuroimmunomodulation. 2009; 16(2): 122-33.

Kemmeren JM, Mangen, MJJ, van Duynhoven YTHP, Havelaar AH. Priority setting of foodborne pathogens: disease burden and costs of selected enteric pathogens. Bilthoven: National Institute of Public Health and Environment; 2006. Report No.: RIVM rapport 330080001.

Lebech M, Andersen O, Christensen NC, Hertel J, Nielsen HE, Peitersen B, et al. Feasibility of neonatal screening for toxoplasma infection in the absence of prenatal treatment. Danish Congenital Toxoplasmosis Study Group. Lancet. 1999; 353(9167): 1834-7.

Melse JM, Essink-Bot ML, Kramers PG, Hoeymans N. A national burden of disease calculation: Dutch disability-adjusted life-years. Dutch Burden of Disease Group. Am J Public Health. 2000; 90(8): 1241-7.

Montoya J, Remingtons J. Toxoplasma gondii. In: Mandell G, Douglas, Benett J, editors. Principles and Practice of Infectious Diseases. Philadelphia: Churchill Livingstone; 2000.

Rorman E, Zamir CS, Rilkis I, Ben-David H. Congenital toxoplasmosis--prenatal aspects of Toxoplasma gondii infection. Reprod Toxicol. 2006; 21(4): 458-72.

Schmidt DR, Hogh B, Andersen O, Fuchs J, Fledelius H, Petersen E. The national neonatal screening programme for congenital toxoplasmosis in Denmark: results from the initial four years, 1999-2002. Archives of disease in childhood. 2006; 91(8): 661-5.

Torrey EF, Yolken RH. Toxoplasma gondii and schizophrenia. Emerg Infect Dis. 2003; 9(11): 1375-80.

Tuberculosis

Tuberculosis (TB) is an infectious disease caused by the Mycobacterium tuberculosis. The term �tuberculosis� is also used for other similar diseases caused by M. bovis and M. africanum (Fitzgerald, 2005; Comstock, 1998). However, for the purposes of the disease report, outcome tree and model presented here, only those infections caused by M. tuberculosis complex are considered.

Tuberculosis bacteria are transmitted via droplets � by coughing, sneezing or talking � and mostly affect the lungs of humans, although they can also result in a systemic disease, affecting virtually all organs (Fitzgerald, 2005). The course �of TB can be split into several phases. The first phase after infection, �primary TB�, is observed in a minority of patients. The majority of infected (asymptomatic) persons proceed to a �latent� stage, lasting from months to several years or even for the rest of their life. Due to endogenous or exogenous reactivation, people may develop active TB after a certain time spent in the latent stage of the disease.

According to published literature only 5�10% of all infected individuals develop symptoms of active (primary) TB (cough, fever, lethargy, and weight loss) in their lifetime (Castillo-Chavez & Feng, 1997; Gideon & Flynn, 2011, Lin & Flynn, 2010, North & Jung, 2004).

Health outcomes and health states associated with tuberculosis infection

The main health outcomes associated with TB infection are active (primary) TB, MDR (multidrug-resistant) TB and XDR (extensively drug- resistant) TB. After initial infection with M. tuberculosis, an immuno-competent person is generally able to stop the replication and spread of bacilli and thus does not develop any symptoms. Primary TB can be split in pulmonary TB (the majority of cases) and extra-pulmonary TB, affecting different sites of the human organism. Given the complexity of the disease course, all TB cases are considered in the model, with a focus on the distinction between drug-susceptible (DS TB), MDR and XDR TB and their relative case fatality proportions (CFP), irrespective of the site of infection.

Of all laboratory-confirmed TB cases notified to ECDC/WHO between 2009 and 2013, on average 4.5% were multidrug-resistant and 14.6% of these cases were extensively drug resistant (ECDC/WHO, 2015). Therefore, in our model of all symptomatic infections 4.5% are considered to be MDR TB and 0.64% are considered to be XDR TB. However, it should be noted that these proportions vary widely across countries and users are advised to tailor them according to the epidemiology of the population under study.

Transition probabilities

In a cost-effectiveness analysis performed by Tseng and colleagues the authors used various assumptions on the progression of TB. Their model estimates the risk of active TB to be about 5% within the first two years of TB infection. Spontaneous resolution without treatment was set to 25%. Cure rates of TB with treatment and cure rates of MDR TB with treatment were 62.4% and 68.6% respectively (Tseng, 2011).

Tiemersma and colleagues estimated CFP and assessed durations of untreated pulmonary TB in HIV-negative patients and stated an overall case-fatality proportion of 30.7% in the first year of follow-up. The highest proportions were observed shortly after diagnosis. The 5-year and 10-year averages for case fatalities were 58% and 73% respectively (Tiemersma, 2011). In their review they also included the study conducted by Berg, estimating sex- and age-specific 10-year mortality rates. For men aged 15�29, 30�49 and >50 years, the 10-year mortality rates were 66%, 70% and 94% respectively. For women aged 15�29, 30�49 and >50 years, 10-year mortality rates were 70%, 69% and 92% respectively (Berg, 1951). Assuming that detected TB cases are treated in Europe, the case fatality proportions cited above overestimate current TB mortality patterns. Duration of pulmonary TB and TB is difficult to estimate due to difficulties in establishing onset of disease; based on estimates from prevalence and incidence studies an average duration of three years was suggested (Tiemersma, 2011).

A cost-effectiveness analysis using Markov models estimated active TB progression rates from underlying latent TB on the basis of disease duration and age-dependent case-fatality rates. Base case rates for developing active TB from latent TB within 1�2 years, 3�5 years and 6�7 years of exposure were estimated at 0.74%, 0.31% (0�2.5%), and 0.16% respectively. Age-specific death rates for people aged 35, 50 and 70 years were 1%, 5% and 10% respectively (Pisu, 2009).

Based on an international TB network, the US Centers for Disease Control and Prevention (CDC) and the World Health Organization (WHO) estimated that in 2004, of 17 960 TB isolates, 20% were MDR and 2% XDR. In population-based trials in the US, Latvia and South Korea 4%, 19% and 15% of all MDR TB cases were XDR in 2004. The studies in the US and Latvia also provided additional information on the progression of MDR and XDR TB in 2004. In the US study 55% of MDR patients completed treatment/were cured and 25% died during treatment. With regard to XDR, 31% completed treatment/were cured and 23% died. Results from Latvia show the percentage of completed treatment/cases cured of MDR TB to be 69% and that of deaths/failures to be 17%. For XDR 61% completed treatment/were cured and 17% died/or had failed treatment (CDC, 2006).

Jaquet and colleagues estimated the impact of DOTS[*] in Haiti and therefore conducted a cost-effectiveness analysis with probability estimates and outcome features of TB taken from literature. For reactivation of latent TB they estimated a probability of 0.1% per year for infection present for more than two years. Within two years of a new TB infection they estimated a base case rate of 5% (2�15%) for developing TB. Cure rates of treated smear positive (drug-sensitive) TB were estimated at 62.4%. For MDR TB, authors assumed a cure rate of 48% (base case; range 48�73%) and the proportion of deaths to be 12% (base case; range 12�26%) (Jacquet, 2006).

Outcome tree parameters

Given the changes in TB epidemiology in Europe during recent decades, the situation has not been sufficiently stable to enable incidence of infection to be estimated from active TB case data. It was therefore decided not to consider latent TB in the model.

Duration of symptomatic TB is set to 0.2�2 years, irrespective of whether it is active, MDR or XDR TB (WHO, 2014).

The case fatality proportion for active TB cases is estimated to be 43% in cases not on TB treatment (Corbett, 2003; Tiemersma, 2011) and 3% in cases on TB treatment (Straetemans, 2011). Given that the estimated incidence of active TB (non-MDR or XDR) in EU/EEA is 10% higher than the notification rate (ECDC/WHO, 2015) and, assuming that all notified cases are being treated, the CFP of active TB (non-MDR or XDR) cases was set at 7%.

The case fatality proportion for MDR TB was set at 12.8% (2.3�23.3%) (Straetemans, 2011). Given the lack of evidence on the case fatality ratio for XDR TB, we used the treatment outcome result category �Died�, notified in the EU/EEA, as a proxy for estimating the XDR TB case fatality proportion and set the value at 27% (ECDC/WHO, 2015).

Model input summary

Table 1. Transition probabilities and distributions used in the outcome tree

Health outcome<br>(Health state)	Distribution of health states in health outcome	Transition probability	Source/assumption
Active TB<br>(Remaining active cases)<br>(MDR, non-XDR)<br>(XDR)	94.86%<br>4.5%<br>0.64%		ECDC/WHO, 2015
Fatal cases following remaining active cases		7%	Modelled based on Corbett, 2003; Tiemersma, 2011; Straetemans, 2011
Fatal cases following MDR TB		12.8% (2.3�23.3%)	Straetemans, 2011
Fatal cases following XDR TB		27%	ECDC/WHO, 2015

Table 2. Disability weights and duration

Health outcome<br>(Health state)	Disability Weight (DW) (Haagsma, 2015)		Duration	
	DW	Label	In years	Source
Active TB<br>(Remaining active cases)<br>(MDR, non-XDR)<br>(XDR)	0.308 (0.264�0.353)<br>0.308 (0.264�0.353)<br>0.308 (0.264�0.353)	Tuberculosis, not HIV infected<br>Tuberculosis, not HIV infected<br>Tuberculosis, not HIV infected	2<br>2<br>2	WHO, 2013<br>WHO, 2013<br>WHO, 2013

References

Berg G. Statistik �ber die Tuberkulosemortalit�t in Kriegszeiten. Beitr�ge zur Klinik der Tuberkulose 1951, 106:1-9.

Castillo-Chavez C, Feng Z. To treat or not to treat: the case of tuberculosis. J Math Biol 1997, 35:629-656.

Centers for Disease Control and Prevention: Emergence of Mycobacterium tuberculosis with extensive resistance to second-line drugs - worldwide, 2000�2004. MMWR Morb Mortal Wkly Rep 2006, 55:301-305.

Comstock G, O'Brian R. Tuberculosis. In Bacterial Infections in Humans - Epidemiology and Control. Volume 3rd edition. Edited by Evans A, Brachman P. New York: Plenum Publishing Corporation; 1998: 777-804

Corbett EL, Watt CJ, Walker N, Maher D, Williams BG, et al. (2003) The growing burden of tuberculosis: global trends and interactions with the HIV epidemic. Arch Intern Med 163: 1009-1021.

Dye C, Scheele S, Dolin P, Pathania V, Raviglione M (for the WHO Global Surveillance Monitoring Project). Global burden of tuberculosis: Estimated incidence, prevalence, and mortality by country. JAMA 1999, 282:677-686.

European Centre for Disease Prevention and Control/WHO Regional Office for Europe. Tuberculosis surveillance and monitoring in Europe 2015.

European Centre for Disease Prevention and Control, WHO Regional Office for Europe: Tuberculosis surveillance and monitoring in Europe. In: Tuberculosis surveillance and monitoring in Europe. Stockholm: European Centre for Disease Prevention and Control; 2012.

Fitzgerald D, Haas D. Mycobacterium tuberculosis. In: Principles and Practice of Infectious Diseases. Sixth edition. Edited by Mandell G, Bennett J, Dolin R. Philadelphia: Elsevier Churchill Livingstone; 2005: 2852-2886

Gideon HP, Flynn JL. Latent tuberculosis: what the host "sees"? Immunol Res 2011, 50:202-212.

Global Tuberculosis Report 2013. 1.Tuberculosis � epidemiology. 2.Tuberculosis, Pulmonary � prevention and control. 3.Tuberculosis � economics. 4.Tuberculosis, Multidrug-Resistant. 5. Annual reports. World Health Organization. ISBN 978 92 4 156465 6. Available at: http://apps.who.int/iris/bitstream/10665/91355/1/9789241564656_eng.pdf

Global Tuberculosis Report 2014. 1.Tuberculosis � epidemiology. 2.Tuberculosis, Pulmonary � prevention and control. 3.Tuberculosis � economics. 4.Tuberculosis, Multidrug-Resistant. 5. Annual reports. World Health Organization. ISBN 978 92 4 156480 9. Available at: http://www.who.int/tb/publications/global_report/gtbr14_main_text.pdf?ua=1

Haagsma JA, Maertens de Noordhout C, Polinder S, Vos T, Havelaar AH, Cassini A, Devleesschauwer B, Kretzschmar ME, Speybroeck N, Salomon JA. Assessing disability weights based on the responses of 30,660 people from four European countries. Population Health Metrics 2015; 13: 10

Jacquet V, Morose W, Schwartzman K, Oxlade O, Barr G, Grimard F, et al. Impact of DOTS expansion on tuberculosis related outcomes and costs in Haiti. BMC Public Health 2006, 6:209.

Jassal M, Bishai WR. Extensively drug-resistant tuberculosis. Lancet Infect Dis 2009, 9:19-30.

Lin PL, Flynn JL. Understanding Latent Tuberculosis: A Moving Target. The Journal of Immunology 2010, 185:15-22.

Murray CJL, Lopez AD, World Health Organization, World Bank, Harvard School of Public Health. The global burden of disease: a comprehensive assessment of mortality and disability from diseases, injuries, and risk factors in 1990 and projected to 2020. Geneva: World Health Organization; 1996.

North RJ, Jung YJ. Immunity to tuberculosis. Annu Rev Immunol 2004, 22:599-623.

Pisu M, Gerald J, Shamiyeh JE, Bailey WC, Gerald LB. Targeted tuberculosis contact investigation saves money without sacrificing health. J Public Health Manag Pract 2009, 15:319-327.

Raviglione M. Tuberculosis - the essentials. New York: Informa Health Care; 2010.

Straetemans M, Glaziou P, Bierrenbach AL, Sismanidis C, van der Werf MJ. Assessing tuberculosis case fatality ratio: a meta-analysis. PLoS One 2011, 6:e20755.

Tiemersma EW, van der Werf MJ, Borgdorff MW, Williams BG, Nagelkerke NJ. Natural history of tuberculosis: duration and fatality of untreated pulmonary tuberculosis in HIV negative patients: a systematic review. PLoS One 2011, 6:e17601.

Tseng CL, Oxlade O, Menzies D, Aspler A, Schwartzman K. Cost-effectiveness of novel vaccines for tuberculosis control: a decision analysis study. BMC Public Health 2011, 11:55.

World Health Organization: Global tuberculosis control: epidemiology, planning, financing. WHO report 2009. Geneva: World Health Organization; 2009.

Variant Creutzfeldt-Jakob disease (vCJD)

The initial symptoms of variant Creutzfeldt-Jakob disease (vCJD) are usually psychiatric, most frequently depression, anxiety and withdrawal (Henry & Knight, 2002; Will & Ward, 2004). After a median of six months, neurological features develop, including cognitive impairment, ataxia and involuntary movements. The clinical course is progressive with the development of dementia and diffuse cortical deficits.

Death occurs after a median of 14 months from the onset of symptoms (range 6–39 months) and is often due to an intercurrent infection (Will & Ward, 2004). However, Henry and Knight stated that the disease is fatal after a median of 13 months and a range of 6–39 months (Henry & Knight, 2002).

In the study by Hilton (Hilton, 2006) the mean age at death for vCJD is 26 years and 29 years with a range of 12–74 years (Will & Ward, 2004; Smiths, 2004). This is in line with the overall median age of 28 at death for all vCJD diagnoses in the UK during the period January 1994– December 2009, with a range from 14 to 75 (Andrews, 2010). During the epidemic, the median age of onset did not change over time, suggesting an important age-related risk. This could be due to an age-dependent susceptibility, age-related exposure or both (Hilton, 2006). There is no significant difference in deaths between males and females (56% male, p=0.12).

Precise estimates of the length and variability of the incubation period for vCJD are difficult to obtain since they require knowledge of the time of infection, whereas exposure may have occurred over several years. Ghani assumes that the incubation period is approximately 15–18 years (Ghani, 2002), whereas Collinge concludes that the incubation period would be at least 11 years (Collinge, 1999).

Although a peak has passed, it is possible that there will be future peaks, possibly in other genetic groups. To date, all cases of vCJD have been genotyped as methionine homozygous at codon 129 of the PrP gene (about 40% of the population). If the other 60% of the population is not completely resistant to infection, the disease in these individuals is associated with a longer incubation period, therefore epidemics in this group may still occur (Smith, 2004). Kaski et al. reported the first suspected clinical case of vCJD in an individual heterozygous for methionine/valine (Kaski, 2009).

There is also the possibility of ongoing person-to-person transmission, as seen with three cases of vCJD infection following transfusion of packed red blood cells from asymptomatic donors who subsequently died from vCJD (Ironside, 2010). Furthermore, Peden et al. described a vCJD infection in the first known asymptomatic patient (Millar, 2010; Peden, 2010). The patient died from unrelated pathology with no evidence of neurological diseases. The infection was detected in a study of autopsy and biopsy materials from 17 neurologically asymptomatic patients with haemophilia, considered to be at increased risk of vCJD. The most likely route of infection was receipt of UK plasma products.

Finally, Smith assumes that the ascertainment of vCJD cases in young adults is nearly complete. In the absence of a reliable, minimally invasive, diagnostic test, the possibility remains that cases in the elderly are being missed due to the small proportion of those dying with dementia that are subject to post-mortem examination (Smiths, 2004).

Model input summary

Table 1. Transition probabilities and distributions used in the outcome tree

Health outcome<br><br>(Health state)	Percent of health outcome in health state	Transition probability	Source/assumption
Fatal cases following symptomatic infection		100%	

Table 2. Disability weights and duration

Health outcome (Health state)	Disability Weight (DW) (Haagsma, 2015)		Duration	
	DW	Label	In years	Source
Symptomatic infection	0.407 (0.36–0.46)	Motor plus cognitive impairments, severe.	1.151 (0.5–3.205)	Will & Ward, 2004

References

Ang LW, Chua LT, James L, Goh KT. Epidemiological surveillance and control of rubella in Singapore, 1991-2007. Ann Acad Med Singapore. 2010 Feb;39(2):95-101.

Andrews NJ. Incidence of variant Creutzfeldt-Jakob disease diagnoses and deaths in the UK, January 1994 - December 2009. 2010 [updated 22 June 2010]; Available from: http://www.cjd.ed.ac.uk/cjdq64jun10.pdf.

Collinge J. Variant Creutzfeldt-Jakob disease. Lancet. 1999 Jul 24;354(9175):317-23.

Ghani AC. The epidemiology of variant Creutzfeldt-Jakob disease in Europe. Microbes Infect. 2002 Mar;4(3):385-93.

Haagsma JA, Maertens de Noordhout C, Polinder S, Vos T, Havelaar AH, Cassini A, Devleesschauwer B, Kretzschmar ME, Speybroeck N, Salomon JA. Assessing disability weights based on the responses of 30,660 people from four European countries. Population Health Metrics 2015; 13: 10

Henry C, Knight R. Clinical features of variant Creutzfeldt-Jakob disease. Rev Med Virol. 2002 May-Jun;12(3):143-50.

Hilton DA. Pathogenesis and prevalence of variant Creutzfeldt-Jakob disease. J Pathol. 2006 Jan;208(2):134-41.

Ironside JW. Variant Creutzfeldt-Jakob disease. Haemophilia. 2010 Jul;16 Suppl 5:175-80.

Kaski D, Mead S, Hyare H, Cooper S, Jampana R, Overell J, et al. Variant CJD in an individual heterozygous for PRNP codon 129. Lancet. 2009 Dec 19;374(9707):2128.

Millar CM, Connor N, Dolan G, Lee CA, Makris M, Wilde J, et al. Risk reduction strategies for variant Creutzfeldt-Jakob disease transmission by UK plasma products and their impact on patients with inherited bleeding disorders. Haemophilia. 2010 Mar;16(2):305-15.

Peden A, McCardle L, Head MW, Love S, Ward HJ, Cousens SN, et al. Variant CJD infection in the spleen of a neurologically asymptomatic UK adult patient with haemophilia. Haemophilia. 2010 Mar;16(2):296-304.

Smith PG, Cousens SN, d' Huillard Aignaux JN, Ward HJ, Will RG. The epidemiology of variant Creutzfeldt-Jakob disease. Curr Top Microbiol Immunol. 2004;284:161-91.

Will RG, Ward HJ. Clinical features of variant Creutzfeldt-Jakob disease. Curr Top Microbiol Immunol. 2004;284:121-32.
